# Photoinduced C(sp^3^)–H sulfination empowers the direct and chemoselective introduction of the sulfonyl group†

**DOI:** 10.1039/d1sc04245a

**Published:** 2021-09-28

**Authors:** Shengfei Jin, Graham C. Haug, Ramon Trevino, Viet D. Nguyen, Hadi D. Arman, Oleg V. Larionov

**Affiliations:** Department of Chemistry, The University of Texas at San Antonio One UTSA Circle San Antonio TX 78249 USA oleg.larionov@utsa.edu

## Abstract

Direct installation of the sulfinate group by the functionalization of unreactive aliphatic C–H bonds can provide access to most classes of organosulfur compounds, because of the central position of sulfinates as sulfonyl group linchpins. Despite the importance of the sulfonyl group in synthesis, medicine, and materials science, a direct C(sp^3^)–H sulfination reaction that can convert abundant aliphatic C–H bonds to sulfinates has remained elusive, due to the reactivity of sulfinates that are incompatible with typical oxidation-driven C–H functionalization approaches. We report herein a photoinduced C(sp^3^)–H sulfination reaction that is mediated by sodium metabisulfite and enables access to a variety of sulfinates. The reaction proceeds with high chemoselectivity and moderate to good regioselectivity, affording only monosulfination products and can be used for a solvent-controlled regiodivergent distal C(sp^3^)–H functionalization.

## Abstract 2

The photoinduced C–H sulfination of abundant aliphatic C–H bonds provides direct access to all major classes of organosulfur compounds *via* the intermediacy of synthetically versatile sulfinate salts.

## Graphical abstract

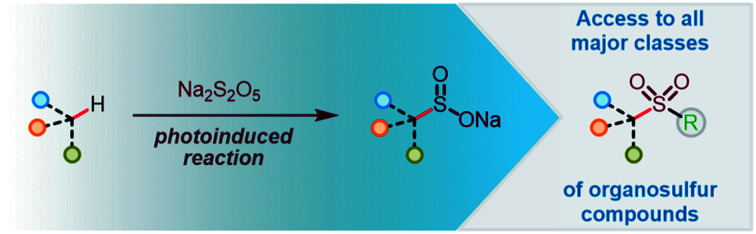

## Introduction

**Fig. 1:**
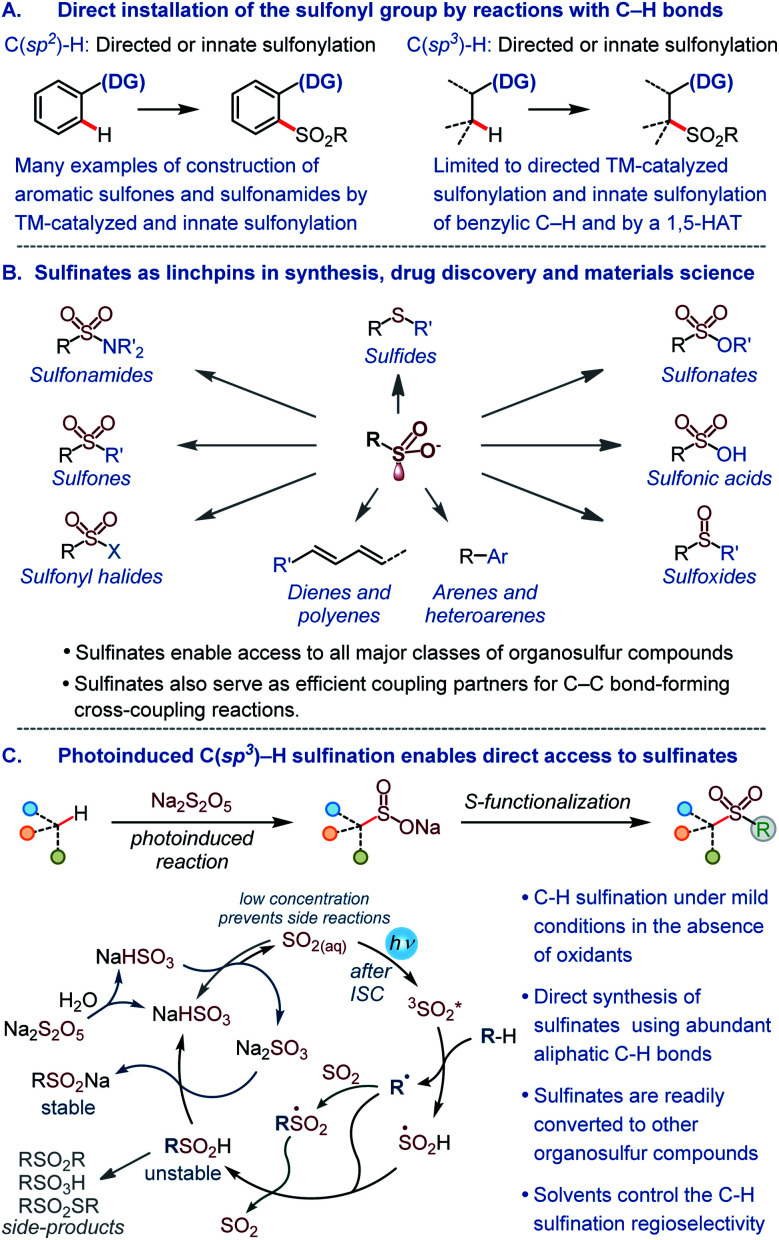
(A) Overview of methods for the direct installation of the sulfonyl group by reactions with C–H bonds. (B) Key synthetic roles of sulfinates. (C) The photoinduced C(sp^3^)–H sulfination.

The sulfonyl group is one of the most important functional groups in organic synthesis,^1^ materials science,^2^ and medicinal chemistry.^3^ However, methods are lacking for the direct installation of the sulfonyl group by harnessing the abundant aliphatic C–H bonds with high potential for a rapid build-up of the structural diversity. The introduction of the sulfonyl group into organic molecules by the C–S bond formation is instead typically accomplished in a stepwise fashion *via* pregenerated reactive intermediates,^4,5^ while the direct installation of the sulfonyl group by reactions with C–H bonds remains underdeveloped (Fig. 1A). Currently available methods for the introduction of the sulfonyl group by means of C–H functionalization largely comprise sulfonylations (*e.g.*, reactions producing sulfones and sulfonamides) of aromatic substrates proceeding by transition metal-catalyzed pathways that either require a directing group or exploit the innate reactivity of the aromatic ring.^6^ The scope of the reactions that engage aliphatic C–H bonds remains limited, and only few examples of C(sp^3^)–H sulfonylation have been described to date.^7^ Importantly, no C(sp^3^)–H sulfination, *i.e.*, a reaction that can enable direct access to sulfinate salts by the functionalization of aliphatic C–H bonds is currently available. Sulfinates have recently emerged as highly versatile synthetic intermediates that can be used to access all major classes of organosulfur compounds^1*a*,4^ and as coupling partners in new regio- and stereoselective C–C bond-forming cross-coupling reactions (Fig. 1B).^5^

Given the central position of the sulfonyl group in medicine and the growing recognition of the importance of increasing the fraction of saturated residues (Fsp^3^) in compounds that enter screening as a way of improving success rates of drug discovery campaigns,^8^ new methods are required that selectively install the sulfonyl group by the functionalization of C(sp^3^)–H bonds. In addition, to be broadly synthetically useful these methods should enable facile access to a variety of classes of organosulfur compounds. Due to the synthetic versatility of sulfinates, C–H sulfination will satisfy these requirements. However, sulfinates are generally incompatible with the oxidative conditions that are required for transition metal-catalyzed C–H functionalization, and a broad scope synthesis of sulfinates directly from C–H bonds has remained elusive.

Recent studies of photoinduced transformations have resulted in the development of new and efficient methods, enabling construction of a variety of carbon–carbon and carbon–heteroatom bonds under mild conditions and in the absence of precious and toxic metals that are typically required to effect the bond formation.^9^

Sulfur dioxide is known to produce sulfonyl group-containing mixtures of products that include sulfinic acids in a photoinduced gas-phase reaction with C_1_–C_4_ alkanes. The reaction proceeds *via* the excitation of sulfur dioxide that undergoes intersystem crossing (ISC) to the reactive triplet state (Fig. 1C). The triplet sulfur dioxide then abstracts a hydrogen atom from the substrate generating alkyl and hydroxysulfinyl (S(O)OH) radicals. Subsequent radical combination or trapping of the alkyl radical by sulfur dioxide and hydrogen abstraction from S(O)OH produce the sulfinic acid product (Fig. 1) that is prone to decomposition under the reaction conditions.^10^ Despite the significant synthetic potential, the reaction has not found synthetic applications because of the harsh gas phase conditions, the use of pressurized toxic sulfur dioxide gas and mercury vapors, formation of many by-products, low yields (typically ≤10%), and a narrow substrate scope.

Due to the instability of sulfurous acid (H_2_SO_3_) in aqueous solutions, sulfite salts (*e.g.*, sodium hydrogen sulfite) exist in an equilibrium with dissolved sulfur dioxide.^11^ We hypothesized that the quantities of sulfur dioxide that are present in the solution (∼0.003M in a 1M solution of NaHSO_3_) will be sufficient to effect the photoinduced C(sp^3^)–H sulfination, producing stable sulfinate salts. Sulfur dioxide is well soluble in organic solvents, and we expected biphasic solvent mixtures also to be suitable for reactions with water-insoluble organic substrates. Given the mild conditions, the absence of reactive oxidants and transition metals, the low concentration of the highly reactive photoexcited sulfur dioxide, and the *in situ* formation of stable sulfinates, thus obviating the isolation of unstable sulfinic acids, it was also expected that the method would solve the challenges that have hitherto prevented the development of a broadly useful photoinduced C(sp^3^)–H sulfination.

We report herein an efficient photoinduced direct sulfination of aliphatic C–H bonds, producing sulfinates that serve as versatile synthetic linchpins and provide access to other key classes of organosulfur compounds. Remarkably, and in contrast to other radical C–H functionalizations, the reaction produces only monosulfination products, while the regioselectivity of the C–H sulfination can be controlled by the solvent, enabling for the first time a regiodivergent sulfination of distal C–H bonds.

## Results and discussion

**Scheme 1:**
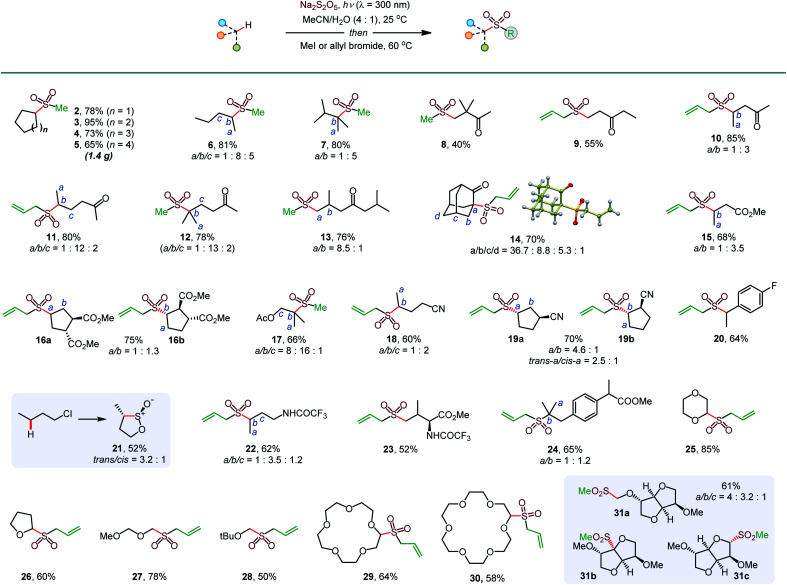
Scope of the photoinduced C–H sulfination. ^*a*^Dichloromethane as a solvent. ^*b*^HFIP as a solvent. ^*c*^1M HCl was used instead of water. ^*d*^Trifluoroethanol as a solvent.

Initial optimization studies with cyclohexane revealed that a clean C–H sulfination can be achieved in the presence of sodium metabisulfite in aqueous acetonitrile under UV-B light (*λ* = 300 nm), producing sulfinate salt **1** in 89% yield (Table 1). Sodium metabisulfite is a bench stable and inexpensive reagent that is used as a pharmaceutical and food preservative. In aqueous solutions sodium metabisulfite rapidly hydrolyzes to sodium hydrogen sulfite. Sodium metabisulfite has one of the highest SO_2_ equivalent contents (65.4%) and is one of the most atom-economical sulfur dioxide precursors.^11*a*,12^ Shorter- (254 nm) or longer- (350 nm) wavelength light afforded the product in lower yields. Other solvents (*e.g.*, dichloromethane, hexafluoroisopropanol (HFIP), entries 5 and 6) were less suitable for the C–H sulfination of a hydrocarbon substrate but could be used for other types of reactants (*vide infra*). Sodium bisulfite also mediated the sulfination, albeit with a lower yield than the freshly prepared solution from metabisulfite, while no reaction was observed with sodium sulfite (entries 7 and 8). The influence of structural and electronic effects on the photoinduced C–H sulfination was examined next with a variety of substrates and using methyl and allyl sulfone products as readouts to facilitate the analysis (Scheme 1). Cycloalkanes of various ring sizes **2–5** reacted smoothly, including on a gram scale (*e.g.*, **5**). Acyclic substrates **6** and **7** were equally suitable, providing an opportunity to study the selectivity of the hydrogen abstraction by triplet sulfur dioxide. The *k*(2°) : *k*(1°) and *k*(3°) : *k*(1°) ratios were 15 : 1 and 30 : 1 respectively, indicating that the hydrogen abstraction selectivity of triplet sulfur dioxide is comparable to that of the *tert*-butoxy radical that is commonly used in synthetic radical chemistry.^13^ β-Sulfonylketones **8** and **9** were readily formed as sole products, highlighting the deactivating effect of the carbonyl group. Other solvents, in particular, hexafluoroisopropanol (HFIP), and pH adjustment had a beneficial effect on the reaction efficiency with deactivated substrates. The C–H sulfination proceeded with β-selectivity for sulfone **10** and substantial γ-selectivity for sulfones **11–13**. Notably, the observed γ-selectivity could be attributed to the selectivity-modulating effect of HFIP (*vide infra*) that was also previously observed for other photoinduced regiodivergent transformations.^9*d*^ Unexpectedly, 2-adamantanone reacted with high selectivity at the α position (**14**). This selectivity is unprecedented, as it stands in contrast to the distal (*d*) selectivity observed with other HAT-inducing radicals and the distal selectivity observed for other ketones in the present system. Given the presence of hydrogen-bonded sulfur dioxide^11*b*,*c*^ and the conformational rigidity of 2-adamantanone, the α selectivity can be facilitated by hydrogen bonding of the carbonyl group with triplet sulfur dioxide with water or protic solvent as a hydrogen bonding linchpin, indicating that directing group-enabled functionalization of specific C–H bonds may be possible with conformationally constrained substrates using the sulfination reaction. Similarly, β-sulfonylesters **15** and **16b** were produced as major products. Notably, β-isomer **16b** was produced as a single diastereomer, pointing to the stereoselectivity of the sulfination step. An ester alkyl group can also be sulfinylated (**17**), with the carboxylic group exerting a deactivating effect on the proximal C–H bonds. The strongly deactivating character of the nitrile group resulted in a higher γ-selectivity (**18** and **19**) with the *trans* preference for the distal C–H sulfination in **19a**, despite the remote position and the small size of the nitrile group. Benzylic C–H bonds can also be readily sulfonylated (**20**). Interestingly, the reaction can be used to access the sultine framework in a *trans*-selective fashion (**21**). Sultines have significant potential as emerging structural units for drug discovery,**3** yet they remain underexplored, as few methods are available for their selective and efficient construction.^14^*N*-Protected amines and amino acids can also serve as suitable substrates, favoring functionalization in the γ-position (**22** and **23**). In addition, functionalization of the remote side chain in ibuprofen highlighted the synthetic potential for the late-stage diversification of medicinal targets (**24**). In contrast to the electron-deficient substrates, the functionalization of cyclic alkyl ethers afforded α-sulfones as sole products (**25–30**). Notably, facile sulfination of crown ethers (**29** and **30**) provides a straightforward approach to appending functionalized side chains that can be used for conjugation and grafting in materials science applications.^15^ In another demonstration of the facility of the C–H sulfination-enabled structural diversification, isosorbide methyl ether that is used in drug delivery applications,^16^ was readily converted to a set of sulfonyl derivatives **31a** and **31b,c**, formed as single diastereomers. Importantly, only monosulfination was observed in all cases, in contrast to other radical processes, *e.g.*, halogenation, that tend to suffer from polyhalogenation.

**Table 1:** Reaction conditions for the direct photoinduced C(sp^3^)–H sulfination^a^

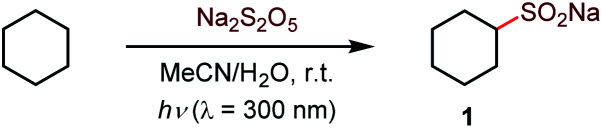		
Entry	Change from the optimal conditions	Yield^b^, %
1	No change	89 (82)^c^
2	No light	0
3	*λ* = 254 nm	38
4	*λ* = 350 nm	35
5	CH_2_Cl_2_ instead of MeCN	82
6	HFIP instead of MeCN	58
7	NaHSO_3_ instead of Na_2_S_2_O_5_	66^d^
8	Na_2_SO_3_ instead of Na_2_S_2_O_5_	0

a Reaction conditions: cyclohexane (0.5 mmol), Na_2_S_2_O_5_ (0.6 mmol), MeCN/H_2_O (4 : 1, 5 mL), *hν* (*λ* = 300 nm), 25 °C.

b Determined by ^1^H NMR with lactic acid as the internal standard.

c Isolated yield.

d 2.4 equiv. of NaHSO_3_.

**Scheme 2:**
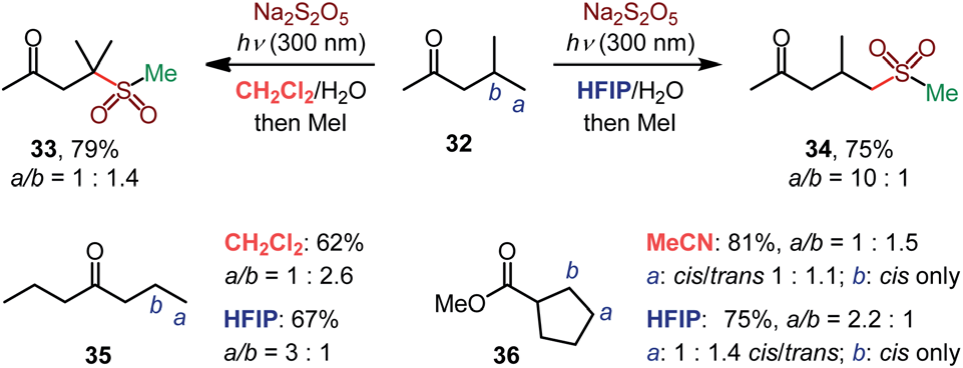
Solvent-induced regiodivergent C–H sulfination.

The high γ-selectivity observed for products **11–13**, **18**, **19**, **22**, and **23** in HFIP indicated that the regioselectivity of the C–H sulfination is controlled by the solvent. Indeed, while β-selective sulfination of the tertiary position in ketone **32** was observed in dichloromethane, the selectivity switched in favor of the distal primary γ position in HFIP (**34**, Scheme 2), enabling solvent-controlled regiodivergent C–H functionalization in the absence of directing groups and catalysts that are typically required to achieve regiodivergent distal C–H functionalizations.^17^

The solvent controlled regioselectivity was also observed for substrates **35** and **36**, with the selectivity shifting from β in aprotic solvents (dichloromethane and acetonitrile) in favor of the distal γ-sulfination in HFIP. Notably, the β-sulfination of ester **36** proceeded with exclusive *cis*-selectivity both in acetonitrile and HFIP.

**Scheme 3:**
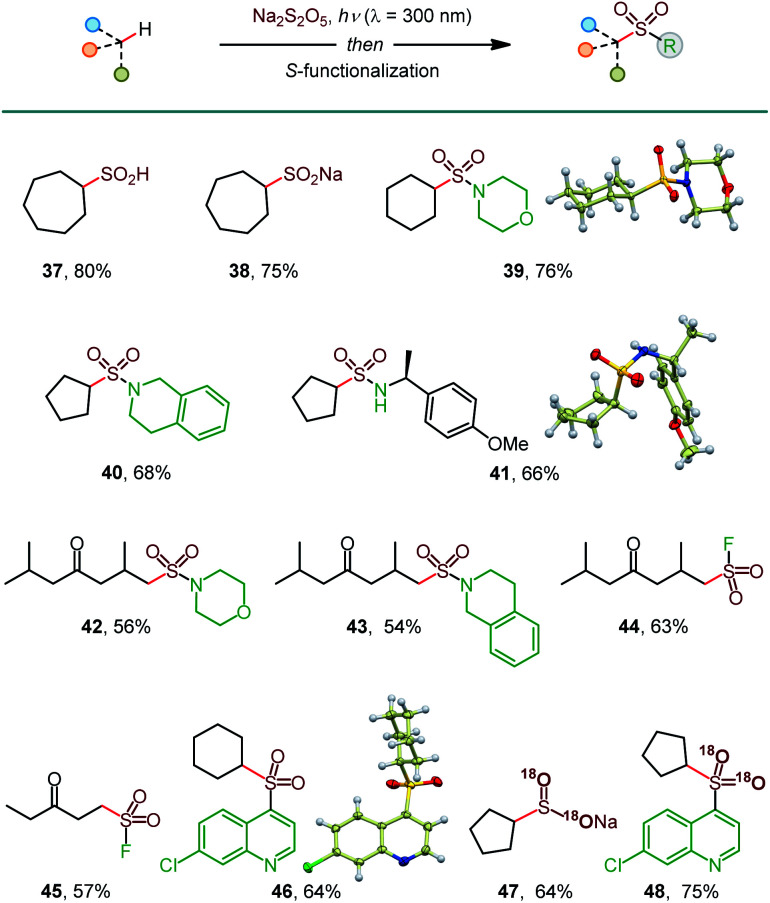
Construction of diverse S-functionalized C–H sulfination products. See Table 1 for the reaction conditions for the C–H sulfination; Dichloromethane was used for **37–41**, **45**, and **46**; HFIP was used for **42–44**; MeCN was used for **47** and **48**. Reaction conditions for the S-functionalization: sulfonamides: amine, I_2_, DCE, 16 h; sulfonyl fluorides: Selectfluor, dioxane, 4 h; sulfone: 4,7-dichloroquinoline, Na_2_S_2_O_8_, 2 h. ^18^O-Labeled products were prepared using H_2_^18^O in the C–H sulfination step.

In addition to sulfinates (*e.g.*, **37**, Scheme 3), other classes of sulfonyl compounds can also be accessed using the photoinduced C–H sulfination. For example, aliphatic sulfinic acids are typically difficult to access, due to facile disproportionation and oxidation, but can be readily synthesized using the reported method following aqueous work-up (**38**). Furthermore, a simple post-sulfination S–N coupling affords sulfonamides that play important roles in medicinal and synthetic chemistry (**39–43**). Sulfonyl fluorides have recently emerged as versatile probes with applications in chemical biology and materials science.^18^ Pleasingly, the photoinduced C–H functionalization of two ketone substrates in conjunction with the Selectfluor-induced sulfinate fluorination afforded sulfonyl fluorides **44** and **45**. Notably, sulfonamides **42** and **43** and sulfonyl fluorides **44** and **45** were formed as single regioisomers with no polysulfination by-products, highlighting the efficiency of the reaction and the excellent HFIP-induced regiocontrol, enabling for the first time exclusive distal C–H amidosulfonation and fluorosulfonation. Sulfones are also readily accessible (**46**) by a metal-free, persulfate-mediated coupling reaction.^4*e*^

Isotopically labeled compounds, *e.g.*, ^18^O-labeled sulfonyl-containing small molecule probes, play increasingly important roles in drug discovery.^19^ However, the installation of the ^18^O-labeled sulfonyl groups remains challenging, because sulfonyl compounds do not undergo a facile ^16^O/^18^O exchange.^20^ Given the mildly acidic (pH 3.9) medium of the C–H sulfination reaction and the propensity of sulfinic acids to undergo an ^16^O/^18^O exchange,^20^ we hypothesized that ^18^O-labeled sulfinate products can be readily accessed, if the C–H sulfination reaction is carried out in the presence of H_2_O^18^. Subsequent S-functionalization can then deliver a variety of ^18^O-labeled sulfonyl compounds that typically do not undergo the ^16^O/^18^O exchange. Indeed, ^18^O-labeled sulfinate salt **47** and sulfone **48** were readily produced with 95% ^18^O isotopic purity, following the simple protocol. These results further highlight the broad synthetic potential of the photoinduced C–H sulfination reaction.

**Fig. 2:**
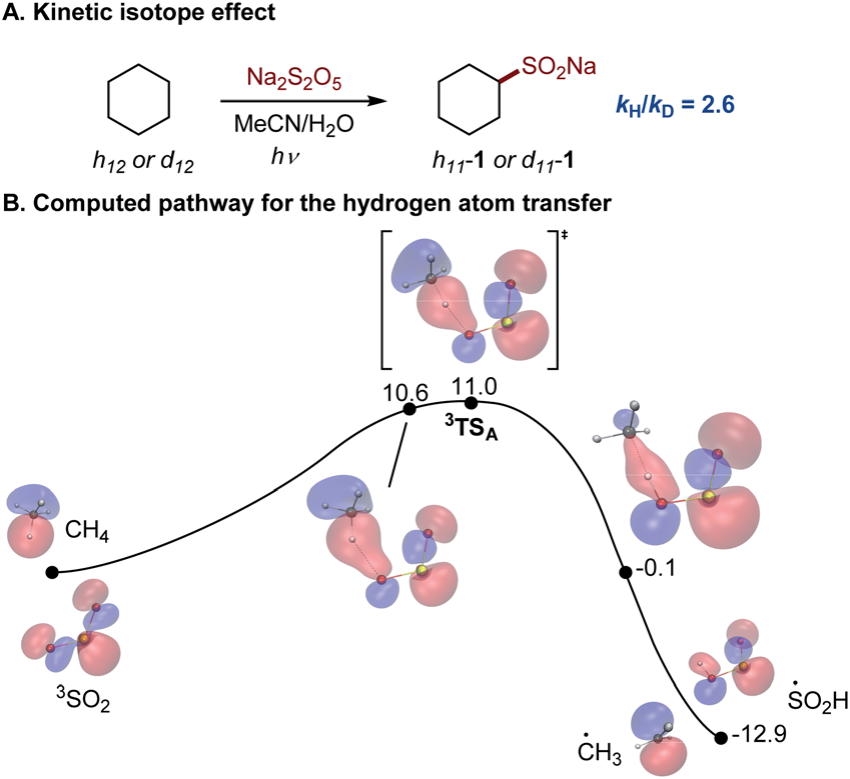
(A) Kinetic isotope effect for the photoinduced C–H sulfination. (B) Computed Gibbs free energy profile for the hydrogen atom transfer reaction of methane with triplet sulfur dioxide with the developing doubly occupied σ-bonding molecular orbital, Δ*G*, kcal mol^−1^. *r*(O–H) = 1.79 Å (10.6 kcal mol^−1^), 1.56 Å (^3^TS_A_), and 1.00 Å (−0.1 kcal mol^−1^).

The photoinduced C–H sulfination exhibits several remarkable features, *e.g.*, a high preference for monosulfination that contrasts other radical processes, and a protic solvent-induced distal γ-functionalization whose understanding is impeded by a lack of mechanistic knowledge of the triplet sulfur dioxide-mediated hydrogen atom transfer from C–H substrates. To gain insights into the mechanism of the C–H sulfination that accounts for the observed selectivity, computational studies were carried out. The C–H sulfination of cyclohexane proceeded with a relatively small kinetic isotope effect of *k*_H_/*k*_D_ = 2.6 (Fig. 2A, see also S1†), pointing to a significantly asymmetrical transition state^21^ in the hydrogen atom transfer step. Indeed, computational studies of the reaction with methane as the C–H substrate show that the hydrogen abstraction by triplet sulfur dioxide proceeds exergonically *via* an early transition state (Fig. 2B) with the interaction of the lowest SOMO-1 of SO_2_ with the σ orbital of the C–H substrate, forming a doubly occupied σ bonding orbital and an antibonding SOMO-1 in the transition state ^3^TS_A_. The activation barrier has a relatively small enthalpic contribution (Δ*G*^≠^ = 11.0 kcal mol^−1^, and Δ*H*^≠^ = 1.7 kcal mol^−1^), as was previously observed for other reactive oxygen-centered radical-mediated hydrogen atom transfers.^13^

**Fig. 3:**
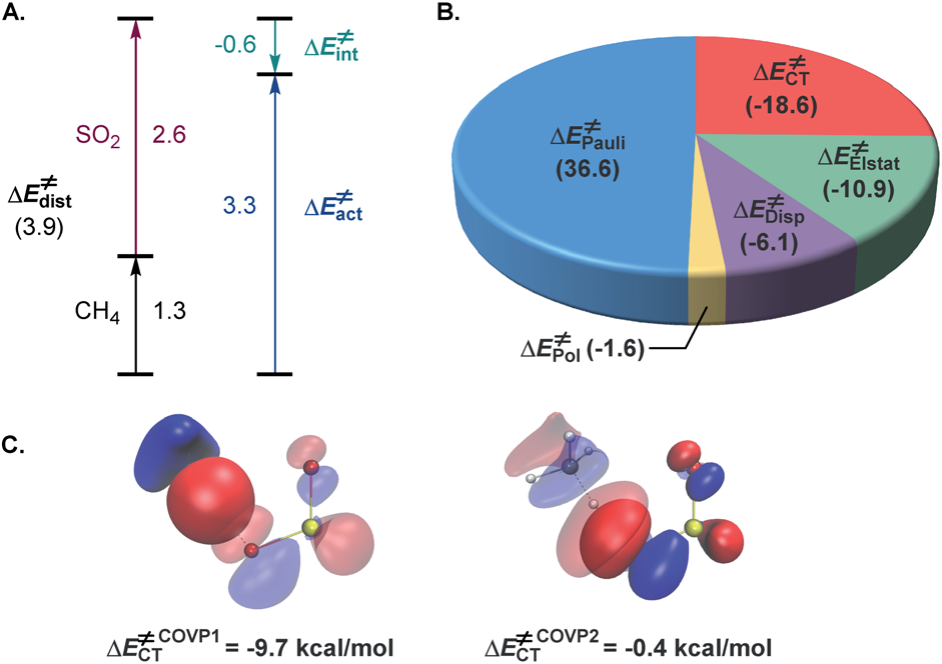
(A) Activation strain model analysis of the hydrogen atom transfer transition state ^3^TS_A_, kcal mol^−1^. (B) Energy decomposition analysis of ^3^TS_A_, kcal mol^−1^. (C) The most significant complementary occupied-virtual pair (COVP) for ^3^TS_A_.

The activation strain model (ASM)^22^ analysis further indicates that the distortion energy is higher for the sulfur dioxide fragment than for the C–H substrate, while both the overall distortion energy and the interaction energy remain relatively small (Fig. 3A).

Further insights into the electronic effects influencing the interaction energy of the transition state were derived from the second-generation energy decomposition analysis based on absolutely localized molecular orbitals (ALMO-EDA2).^23^ Pauli repulsion was the major contributor to the interaction energy, however, charge transfer and dispersion also played significant roles, nearly compensating for the Pauli repulsion, in combination with the electrostatic component (Fig. 3B). The complementary occupied-virtual pair (COVP)^24^ analysis indicated that the most significant charge transfer takes place between a σ orbital of the C–H substrate and the lowest SOMO-1 of the triplet sulfur dioxide in the beta space with substantially smaller α-SOMO-1→σ_C–H_* contribution in the alpha space (Fig. 3C), pointing to the electrophilic character of triplet sulfur dioxide, and is further corroborated by the second order perturbation theory (SOPT) analysis^25^ (see the ESI†). These results underscore the delicate balance of the various stabilizing and destabilizing effects that contribute to the low-barrier hydrogen atom transfer and clarify interfragment interactions that enable the process.

**Fig. 4:**
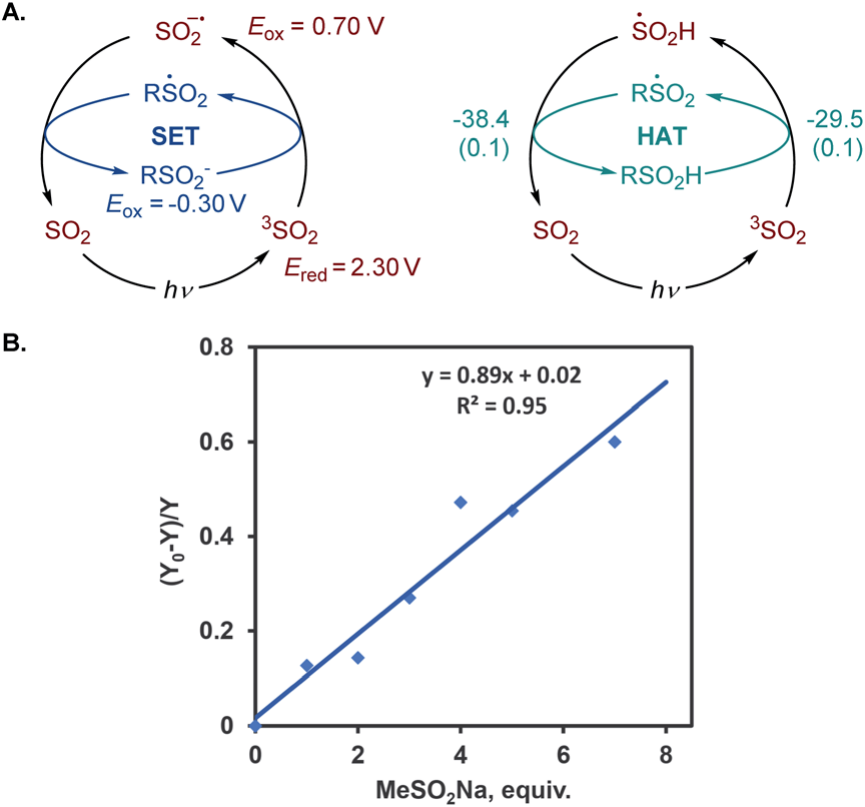
(A) SET and HAT pathways for the deactivation of triplet sulfur dioxide with sulfinates and the inhibitory effect of the added sulfinate salt on the photoinduced C–H sulfination of cyclohexane. Reduction (*E*_red_) and oxidation (*E*_ox_) potentials *vs.* SCE in MeCN and for R = Me. For the HAT process, values reported are Δ*G* (Δ*G*^≠^), kcal mol^−1^, for R = Me. (B) The inhibitory effect of the added sulfinate salt on the photoinduced C–H sulfination of cyclohexane, and (*Y*_0_ − *Y*)/*Y* is the relative change in the yield of product **1** as a function of the added sulfinate.

**Fig. 5:**
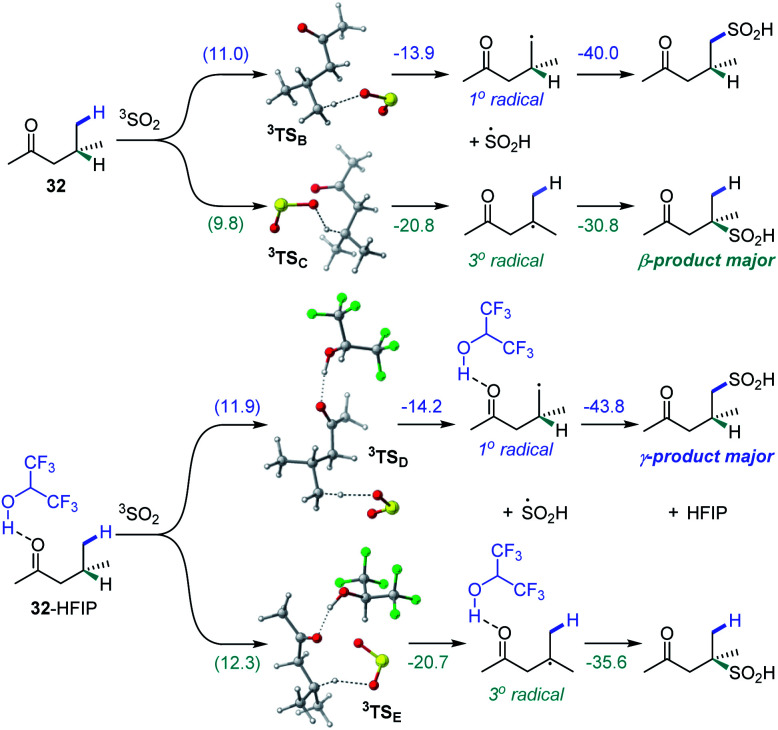
Computed energy profiles of the β- and γ-C–H sulfination pathways for ketone **32** in dichloromethane and for hydrogen bond complex HFIP-**32** in HFIP, Δ*G* (Δ*G*^≠^), kcal mol^−1^.

We further proceeded with the investigation of the high preference for monosulfination observed for all substrates even in the presence of a large excess of sodium metabisulfite (*e.g.*, 4–12 equiv.). We hypothesized that triplet sulfur dioxide can engage in an unproductive single electron transfer/back electron transfer (SET/BET) process with the sulfinate product. Indeed, triplet sulfur dioxide is a strong oxidant with a calculated reduction potential *E*_red_ = 2.30 V *vs.* SCE that can readily oxidize sulfinate salts (*e.g.*, *E*_ox_ = −0.30 V *vs.* SCE for CH_3_SO_2_NBu_4_), producing the corresponding sulfonyl radical and sulfur dioxide anion radical.^26^ The latter (*E*_ox_ = 0.70 V *vs.* SCE) can reduce the sulfonyl radical to sulfinate by a back electron transfer, resulting in net deactivation of photoexcited sulfur dioxide by the C–H sulfination product (Fig. 4A), thus preventing the installation of additional sulfonyl groups and leading to exclusive monosulfination. This conclusion is supported by the observation of the inhibitory effect of the added sulfinate on the reaction performance (Fig. 4B), underscoring the autoinhibitory role of the sulfinate products. Additionally, since the O–H bond in sulfinic acids is substantially weaker (BDE ∼78 kcal mol^−1^)^27^ than C–H bonds, the deactivation of photoexcited sulfur dioxide can also readily proceed *via* a hydrogen atom abstraction by triplet SO_2_ that is followed by back-HAT (Fig. 4A). Both steps are near barrierless and exergonic, indicating that monosulfination can also be effected by the HAT pathway with sulfinic acids present in the acid–base equilibrium. We next explored the origin of the solvent-induced divergence in the β/γ-regioselectivity that is observed in dichloromethane and HFIP. Given the strong hydrogen bond donor ability of HFIP (*α* = 1.96)^28^ and the effects of hydrogen bonding and polar medium on HAT processes,^29^ we hypothesized that hydrogen bonding interactions of HFIP with the carbonyl group substrate amplified by the high polarity (*E*_T_(30) = 65.3)^28^ and very low nucleophilicity (*N*_OTs_ = −4.23)^28^ of the HFIP solvent medium result in the deactivation of the proximal C–H positions in favor of the distal γ-C–H functionalization. Computational studies with ketone **32** as the substrate revealed that the C–H sulfination proceeds with β-selectivity in dichloromethane (Δ*G*^≠^ = 11.0 kcal mol^−1^ for ^3^TS_B_, Δ*G*^≠^ = 9.8 kcal mol^−1^ for ^3^TS_C_, and ΔΔ*G*^≠^_3°/1°_ = −1.2 kcal mol^−1^) in line with the experimental observations (ΔΔ*G*^≠^_3°/1°_ = −1.3 kcal mol^−1^, Fig. 5). Both the HAT step and the subsequent cross-termination of the alkyl and hydroxysulfinyl radicals were substantially exergonic, resulting in an overall thermodynamically favorable C–H functionalization process facilitated by the high triplet energy of sulfur dioxide (73.4 kcal mol^−1^).^30^ Interestingly, when the C–H sulfination of the HFIP-32 hydrogen bond complex was studied with HFIP as a solvent, the regioselectivity inverted in favor of the γ-functionalization (Δ*G*^≠^ = 11.9 kcal mol^−1^ for ^3^TS_D_, Δ*G*^≠^ = 12.3 kcal mol^−1^ for ^3^TS_E_, and ΔΔ*G*^≠^_3°/1°_ = 0.4 kcal mol^−1^) in agreement with the experiment (ΔΔ*G*^≠^_3°/1°_ = 0.3 kcal mol^−1^). Notably, both the β (3°) and the γ (1°) HAT pathways suffered from higher barriers in HFIP, however, the β HAT pathway was more sensitive to the deactivating effect of the HFIP ligation, due to the proximity of the carbonyl group. These results indicate that HFIP-mediated hydrogen-bonding interactions can be successfully used to modulate the regioselectivity of synthetically important radical C(sp^3^)–H functionalization reactions.

## Conclusions

In conclusion, we have developed a direct photoinduced C–H sulfination of abundant C(sp^*3*^)–H bonds mediated by sodium metabisulfite in aqueous organic solvent mixtures. The reaction proceeds under ambient and mild conditions and does not require pressurized toxic sulfur dioxide gas. Remarkably, only monosulfination products are formed, and a clean conversion to sulfinates is achieved without disproportionation and oxidation by-products that typically complicate sulfinic acid synthesis. In addition to high chemoselectivity, the reaction allows for the functionalization of distal C–H positions with moderate to good regioselectivity and with solvent effects playing a key role in establishing the regiocontrol. The new method enables a simple and direct conversion of aliphatic C–H bonds to other classes of organosulfur compounds, including sulfonamides, sulfonyl fluorides, and sulfones, and can be used for the facile introduction of ^18^O-labeled sulfonyl groups.

## Data availability

Experimental and computational data associated with this work are provided in the accompanying ESI.†

## Author contributions

SJ and VDN carried out the experiments, GCH and RT performed the computational studies. HDA performed the X-ray crystallography studies. OVL conceived the project, wrote the manuscript, and co-wrote and edited the ESI. SJ, VDN, GC, and RT co-wrote the ESI and contributed to writing the manuscript.

## Conflicts of interest

There are no conflicts to declare.

## Supplementary Material

SC-012-D1SC04245A-s001

SC-012-D1SC04245A-s002
