# Supplementary material for: Photoinduced C(sp3)–H sulfination empowers the direct and chemoselective introduction of the sulfonyl group
Source: Chem Sci. 2021 Sep 28;12(41):13914–21. doi: 10.1039/d1sc04245a (PMC8549786; doi:10.1039/d1sc04245a)
Supplement: SC-012-D1SC04245A-s001 [file SC-012-D1SC04245A-s001.pdf]

Electronic Supplementary Material (ESI) for Chemical Science.
This journal is © The Royal Society of Chemistry 2021

Supporting Information

Photoinduced C(sp^3)–H Sulfination Empowers a Direct, Chemo- and Regioselective Introduction of the Sulfonyl Group

Shengfei Jin,[§] Graham C. Haug,[†] Ramon Trevino,[†] Viet D. Nguyen,[†] Hadi D. Arman, and Oleg V. Larionov*

Department of Chemistry, The University of Texas at San Antonio, San Antonio, Texas 78249, United States

oleg.larionov@utsa.edu

Contents

Materials and experimental details S1
General procedures S3
C–H Sulfination products S5
Sulfonyl derivatives S39
Computational data S53
X-ray crystallographic data S142
NMR spectroscopic data S150
References S288

Mateials and experimental details

Materials: Acetonitrile and dichloromethane were dried over 3 Å molecular sieves. Deionized water was thoroughly degassed prior to use. *N*-Butyl-2,2,2-trifluoro-acetamide, [1] methyl (2,2,2-trifluoroacetyl)-D-valinate, [2] methyl 2-(4-isobutylphenyl) propanoate[3] were prepared as described elsewhere. All other chemicals were obtained from commercial sources and used without further purification.

[†] These authors contributed equally to this work.
[§] Current address: Wuya College of Innovation, Shenyang Pharmaceutical University, Shenyang, Liaoning 110016, P. R. China

Experimental equipment: The photochemical reactions were conducted in quartz test-tubes (typically 6 or 10 mL capacity with GL14 and GL16 screw caps, Quartz Scientific, Inc.) in a Rayonet RPR-100 photochemical reactor equipped with 16 Rayonet RPR-300 or Ushio 8W T5 UV-C lamps with the fan on. Given the higher molar absorptivity of sulfur dioxide at 300 nm, RPR-300 were more efficient light sources. The efficiency of the lamps was found to decrease over time, and new lamps gave the best results. The chamber temperature was 25 °C. The reaction test-tubes were placed ~2 cm from the UV lamps on a stirplate. For heterogenious reactions, efficient stirring was key to achieving high yields, and rare-earth stirbars in combination with a high and stable stirring rate (2500 rpm) served best to prevent a loss in yields due to poor mixing. Reaction mixtures should be thoroughly deoxygenated to prevent side reactons. Cyclic voltammetry (CV) measurements were performed on a CHI 650D potentiostat using a three-electrode cell with a glassy-carbon working electrode, a Ag|AgCl (1M KCl) reference electrode and a Pt counter electrode. CV was conducted at a scan rate of 100 mV s^{-1} for tetrabutylammonium methanesulfinate (0.4mM) in anhydrous degassed acetonitrile with tetrabutylammonium hexafluorophosphate (0.2M) as an electrolyte. Inflection-point potentials were used to characterize irreversible redox processes, since they were shown to provide the best approximation of standard electrochemical potentials for irreversible redox systems.[4]

Purification: Purification was carried out by means of flash chromatography. Thin layer chromatography was carried out on silica gel-coated glass plates (Merck Kieselgel 60 F254). Plates were visualized under ultraviolet light (254 nm) and using a potassium permanganate stain.

Characterization: ^{1}H, ^{13}C, and ^{19}F NMR spectra were recorded at 500 MHz or 300 MHz (^{1}H), 125 MHz or 75 MHz (^{13}C), 470 (^{19}F) MHz on an Agilent Inova 500 or 300, and Bruker AVANCE III 500 instruments in $CDCl_3$ or other specified deuterated solvents with and without tetramethylsilane (TMS) as an internal standard at 25 °C, unless specified

Go back to table of contents

otherwise. Chemical shifts (δ) are reported in parts per million (ppm) from tetramethylsilane (^{1}H and ^{13}C) and $CFCl_3$ (^{19}F). Coupling constants (*J*) are in Hz. Proton multiplicity is assigned using the following abbreviations: singlet (s), doublet (d), triplet (t), quartet (q), quintet (quint.), septet (sept.), multiplet (m), broad (br).

Infrared measurements were carried out neat on a Bruker Vector 22 FT-IR spectrometer fitted with a Specac diamond attenuated total reflectance (ATR) module.

Experimental Procedures

General procedure for the photoinduced C–H sulfination (GP1)

Acetonitrile or dichloromethane or hexafluoroisopropanol (HFIP) (3–5 mL) and $Na_2S_2O_5$ (0.2–3 mmol) were placed in a quartz test-tube equipped with a stirbar. Argon was bubbled through a glass pipet reaching to the bottom of the test-tube while vigorous stirring was maintained for 5 min. The C–H substrate (0.2–1 mmol) and water (0.75–1 mL) were then added, and the test-tube was sealed with a rubber septum. The solution was stirred for 5 min, and the septum on the quartz test-tube was additionally secured with Parafilm® tape to minimize exposure of the solution to air. The reaction mixture was irradiated with vigorous stirring at 25 °C for the specified time in a Rayonet RPR-100 photochemical reactor.

General procedure for the photoinduced C–H sulfination (GP2)

Acetonitrile (5 mL) or HFIP (3mL) or trifluoroethanol (TFE) (3 mL) and $Na_2S_2O_5$ (1.5–3 mmol) were placed in a quartz test-tube equipped with a stirbar. Argon was bubbled through a glass pipet reaching to the bottom of the test-tube while vigorous stirring was maintained for 5 min. The C–H substrate (0.2–0.3 mmol) and 12M aqueous HCl (0.1–0.25 mL) were then added, and the test-tube was sealed with a rubber septum. The solution was stirred for 5 min, and the septum on the quartz test-tube was additionally secured with Parafilm® tape to minimize exposure of the solution to air. The reaction mixture was

Go back to table of contents

irradiated with vigorous stirring at 25 °C for the specified time in a Rayonet RPR-100 photochemical reactor.

General procedure for the alkylation of sulfinate salts obtained by GP1 (GP3)

A pressure tube equipped with a stirbar was purged with argon, and the reaction mixture obtained in GP1 was quickly transferred into the flask. Iodomethane (2–5 mmol) or allyl bromide (2–3 mmol) was added, and the reaction mixture was stirred at 60 °C for 4 h. The reaction mixture was extracted with EtOAc (3 × 15 mL). The organic phases were combined, dried over anhydrous Na_2SO_4 and concentrated under reduced pressure. The crude product was concentrated and purified by flash chromatography on silica gel or neutral aluminum (EtOAc/hexane) to give the desired sulfone.

General procedure for the alkylation of sulfinate salts obtained by GP2 (GP4)

A pressure tube equipped with a stirbar was purged with argon, and the reaction mixture obtained in GP2 was quickly transferred into the flask. *N*,*N*-Diisopropylethylamine (3 mmol), iodomethane or allyl bromide (2–3 mmol) were added, and the reaction mixture was stirred at 60 °C for 4 h. The reaction mixture was extracted with EtOAc (3 × 15 mL). The organic phases were combined, dried over anhydrous Na_2SO_4 and concentrated under reduced pressure. The crude product was purified by flash chromatography on silica gel (EtOAc/hexane) to give the desired sulfone.

Quantum yield measurement

The photon flux of the photochemical setup was determined using the azoxybenzene chemical actinometer system.[5] Incident photon flux: 3.04 μmol photons per second. The direct photoinduced C–H sulfination of cyclohexane was carried out as described in GP1. Yield was determined by ^{1}H NMR spectroscopy, using lactic acid as an internal standard. $\Phi = 0.0051$.

Go back to table of contents

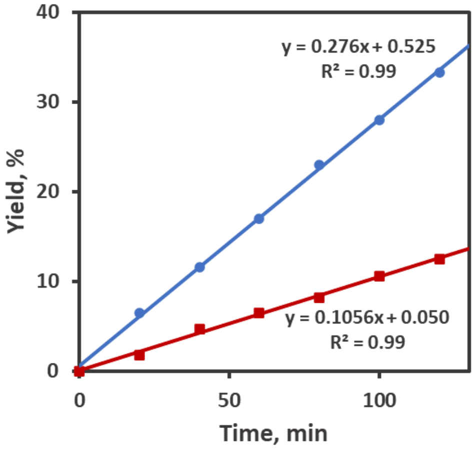

Figure S1. Kinetic isotope effect in the C–H sulfination of cyclohexane. Parallel reactions were performed as described in GP1 with cyclohexane (-●-) and *d*12-cyclohexane (-■-).

C–H Sulfination products

Photoinduced C–H sulfination of cyclohexane: sodium cyclohexanesulfinate (1)[6]

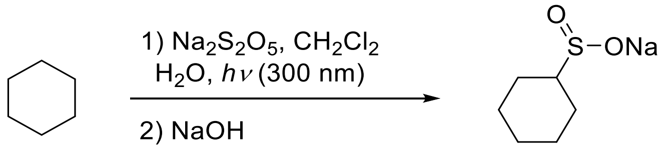

1

According to GP1, a stirred mixture of acetonitrile (4 mL) and $Na_2S_2O_5$ (114 mg, 0.6 mmol) was degassed with Ar for 5 min in a quartz test-tube. Cyclohexane (42 mg, 0.5 mmol) and water (1 mL, degassed prior to use) were then added. The solution was stirred vigorously and irradiated at 25 °C for 36 h in a Rayonet RPR-100 photochemical reactor., 1 M NaOH in methanol solution (1 mL) was added, and the reaction mixture was stirred at room temperature for 10 min. The reaction mixture was concentrated under nitrogen to dryness. The crude product was purified by flash chromatography on silica gel (MeOH/DCM, 1 : 10 v/v) to give sulfinate **1** (70 mg, 82%) as a white solid.

Go back to table of contents

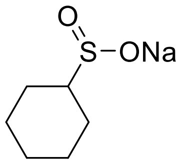

m.p. >250 °C. – ^{1}H NMR (500 MHz, CD_3OD): 1.98–1.75 (5 H, m), 1.65 (1 H, d, J = 11.8 Hz), 1.37–1.12 (5 H, m) ppm. – ^{13}C NMR (125 MHz, CD_3OD): 67.6, 27.2, 26.8, 26.2 ppm. – IR: 3325, 2919, 1650, 1452, 982, cm^{-1}.

Photoinduced C–H sulfination of cyclopentane: sulfone 2[7]

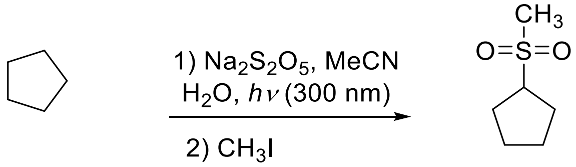

According to GP1, a stirred mixture of MeCN (4.25 mL) and $Na_2S_2O_5$ (114 mg, 0.6 mmol) was degassed with Ar for 5 min in a quartz test-tube. Cyclopentane (35 mg, 0.5 mmol) and water (0.75 mL, degassed prior to use) were then added. The solution was stirred vigorously and irradiated at 25 °C for 16 h in a Rayonet RPR-100 photochemical reactor. The reaction mixture was transferred into a pressure tube, iodomethane (0.3 mL, 5 mmol) and MeOH (1 mL) were added, and the reaction mixture was stirred at 60 °C for 4 h according to GP3. The reaction mixture was then extracted with EtOAc (3 × 15 mL). The organic phases were combined, dried over anhydrous Na_2SO_4 and concentrated under reduced pressure to give sulfone **2** (58 mg, 78%) as a colorless oil.

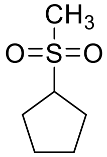

^{1}H NMR (300 MHz, C_6D_6): 2.86 – 2.64 (1 H, m), 2.27–2.09 (3 H, m), 1.94–1.74 (2 H, m), 1.60–1.32 (4 H, m), 1.29–1.11 (2 H, m) ppm. – ^{13}C NMR (75 MHz, C_6D_6): 62.5, 38.8, 27.0, 26.1 ppm. – IR: 3594, 2959, 2873, 1449, 1289, 1124, 966, 763, 596, 538 cm^{-1}.

Go back to table of contents

Photoinduced C–H sulfination of cyclohexane: (methylsulfonyl)cyclohexane (3)[8]

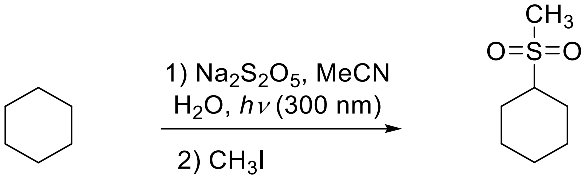

3

According to GP1, a stirred mixture of MeCN (4.25 mL) and $Na_2S_2O_5$ (114 mg, 0.6 mmol) was degassed with Ar for 5 min in a quartz test-tube. Cyclohexane (42 mg, 0.5 mmol) and water (0.75 mL, degassed prior to use) were then added. The solution was stirred vigorously and irradiated at 25 °C for 16 h in a Rayonet RPR-100 photochemical reactor. The reaction mixture was transferred into a pressure tube, iodomethane (0.3 mL, 5 mmol) was added, and the reaction mixture was stirred at 60 °C for 4 h according to GP3. The reaction mixture was then extracted with EtOAc (3 × 15 mL). The organic phases were combined, dried over anhydrous Na_2SO_4 and concentrated under reduced pressure to give sulfone **3** (77 mg, 95%) as a colorless oil.

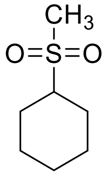

^{1}H NMR (500 MHz, $CDCl_3$): 2.84–2.60 (4 H, m,), 2.10 (2 H, d, *J* = 12.0 Hz), 1.84 (2 H, d, *J* = 12.7 Hz), 1.65 (1 H, d, *J* = 12.5 Hz), 1.49–1.31 (2 H, m), 1.31–1.01 (3 H, m) ppm. – ^{13}C NMR (125 MHz, $CDCl_3$): 62.3, 37.2, 25.4, 25.0 ppm. – IR: 3530, 2932, 2858, 1639, 1453, 1416, 1295, 1266, 1129, 1111, 961, 895, 864, 763, 646, 602, 545 cm^{-1}.

Photoinduced C–H sulfination of cycloheptane: (methylsulfonyl)cycloheptane (4)

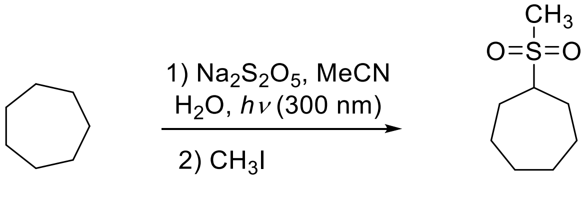

4

According to GP1, a stirred mixture of MeCN (4.25 mL) and $Na_2S_2O_5$ (114 mg, 0.6 mmol) was degassed with Ar for 5 min in a quartz test-tube. Cycloheptane (49 mg, 0.5 mmol) and water (0.75 mL, degassed prior to use) were then added. The solution was stirred

Go back to table of contents

vigorously and irradiated at 25 °C for 16 h in a Rayonet RPR-100 photochemical reactor. The reaction mixture was transferred into a pressure tube, iodomethane (0.3 mL, 5 mmol) and MeOH (1 mL) were added, and the reaction mixture was stirred at 60 °C for 4 h according to GPXX. The reaction mixture was then extracted with EtOAc (3 × 15 mL). The organic phases were combined, dried over anhydrous Na_2SO_4 and concentrated under reduced pressure to give sulfone **4** (64 mg, 73%) as a colorless oil.

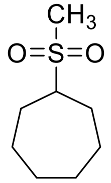

^{1}H NMR (300 MHz, C_6D_6): 2.64–2.39 (1 H, m), 2.23 (3 H, s), 2.11–1.90 (2 H, m), 1.60–1.37 (4 H, m), 1.36–0.91 (6 H, m) ppm. – ^{13}C NMR (75 MHz, C_6D_6): 64.0, 37.0, 28.3, 27.4, 26.0 ppm. – IR: 2926, 2857, 1709, 1463, 1363, 1281, 1131, 963, 766, 653, 594, 538, 519 cm^{-1}. – HRMS calcd for $C_8H_{17}O_2S$: 177.0944, found 177.0945 [M+H$^+$].

Photoinduced C–H sulfination of cyclooctane: (methylsulfonyl)cyclooctane (5)

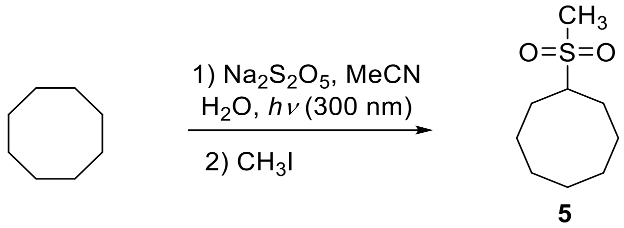

According to GP1, a stirred mixture of MeCN (4.25 mL) and $Na_2S_2O_5$ (114 mg, 0.6 mmol) was degassed with Ar for 5 min in a quartz test-tube. Cyclooctane (56 mg, 0.5 mmol) and water (0.75 mL, degassed prior to use) were then added. The solution was stirred vigorously and irradiated at 25 °C for 16 h in a Rayonet RPR-100 photochemical reactor. The reaction mixture was transferred into a pressure tube, iodomethane (0.3 mL, 5 mmol) and MeOH (1 mL) were added, and the reaction mixture was stirred at 60 °C for 4 h according to GP3. The reaction mixture was then extracted with EtOAc (3 × 15 mL). The organic phases were combined, dried over anhydrous Na_2SO_4 and concentrated under reduced pressure to give sulfone **5** (62 mg, 65%) as a colorless oil.

Go back to table of contents

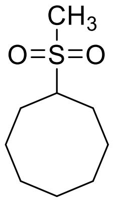

^{1}H NMR (300 MHz, C_6D_6): 2.75–2.50 (1 H, m), 2.21 (3 H, s), 2.11–1.87 (2 H, m), 1.58–1.01 (12 H, m) ppm. – ^{13}C NMR (75 MHz, C_6D_6): 62.3, 37.2, 25.4, 25.0 ppm. – IR: 2927, 1748, 1292, 1135, 651, 538 cm^{-1}. – HRMS calcd for $C_9H_{19}O_2S$: 191.11, found 191.1099 [M+H$^+$].

Photoinduced C–H sulfination of pentane: sulfone 6a-c

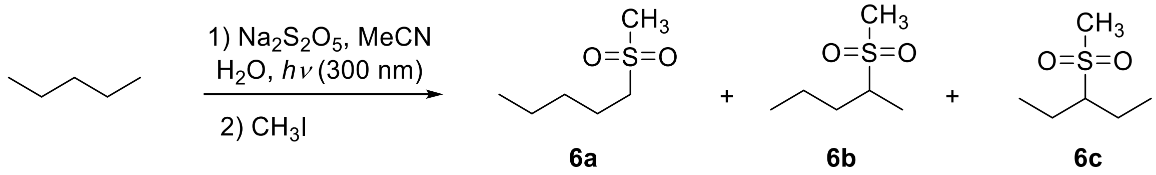

According to GP1, a stirred mixture of acetonitrile (4.25 mL) and $Na_2S_2O_5$ (95 mg, 0.5 mmol) was degassed with Ar for 5 min in a quartz test-tube. Pentane (36 mg, 0.5 mmol) and water (0.75 mL, degassed prior to use) were then added. The solution was stirred vigorously and irradiated at 25 °C for 16 h in a Rayonet RPR-100 photochemical reactor. The reaction mixture was then transferred into a pressure tube, iodomethane (0.3 mL, 5 mmol) and MeOH (1 mL) were added, and the reaction mixture was stirred at 60 °C for 4 h according to GP3. The reaction mixture was then extracted with EtOAc (3 × 15 mL). The organic phases were combined, dried over anhydrous Na_2SO_4 and concentrated under reduced pressure to give three regioisomers (61 mg, 81%, 1 : 8 : 5 ratio of isomers **6a-c**) as a colorless oil.

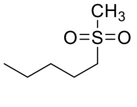

6a: ^{1}H NMR (300 MHz, C_6D_6): 3.01–2.91 (2 H, m), 2.88 (3 H, s), 1.76–0.98 (6 H, m), 0.40 (3 H, t, J = 9.0 Hz) ppm.

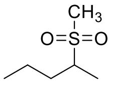

6b: ^{1}H NMR (300 MHz, C_6D_6): 3.01–2.91 (1 H, m), 2.78 (3 H, s), 1.76–0.98 (4 H, m), 0.99 (3 H, d, J = 8.7 Hz), 0.46 (3 H, t, J = 8.9 Hz) ppm.

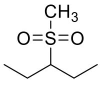

6c: ^{1}H NMR (300 MHz, C_6D_6): 2.80 (3 H, s), 2.62 (1 H, tt, J = 9.0, 6.1 Hz), 1.76–0.98 (4 H, m), 0.62 (6 H, t, J = 9.4 Hz) ppm.

Go back to table of contents

^{13}C NMR (75 MHz, C_6D_6, mixture of regioisomers): 66.3, 59.0, 54.8, 40.5, 38.6, 37.2, 31.2, 30.5, 22.2, 20.7, 19.9, 13.8, 13.2, 11.4 ppm. – IR: 2933, 2876, 1468, 1293, 1134, 1120, 958, 768, 635, 533 cm^{-1}. – HRMS calcd for $C_6H_{15}O_2S$: 151.0787, found 151.0787 [M+H$^+$].

Photoinduced C–H sulfination of 2,3-dimethylbutane: sulfone 7a,b

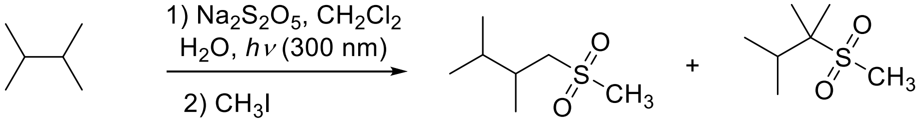

7a **7b**

According to GP1, a stirred mixture of dichloromethane (3 mL) and $Na_2S_2O_5$ (190 mg, 1 mmol) was degassed with Ar for 5 min in a quartz test-tube. 2,3-Dimethylbutane (56 mg, 0.5 mmol) and water (1.5 mL, degassed prior to use) were then added. The solution was stirred vigorously and irradiated at 25 °C for 16 h in a Rayonet RPR-100 photochemical reactor. The reaction mixture was transferred into a pressure tube, iodomethane (0.6 mL, 10 mmol) and MeOH (1 mL) were added, and the reaction mixture was stirred at 60 °C for 4 h according to GP3. The reaction mixture was then extracted with EtOAc (3 × 15 mL). The organic phases were combined, dried over anhydrous Na_2SO_4 and concentrated under reduced pressure to give two regioisomers (131 mg, 80%, 5 : 1 ratio of **6b** and **6a**) as a colorless oil.

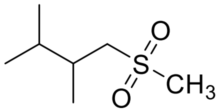

7a: ^1H NMR (300 MHz, C_6D_6): 2.54 (1 H, dd, J = 13.9, 3.5 Hz), 2.34–2.25 (1 H, m), 2.21 (3 H, s), 1.52–1.36 (1 H, m), 1.09–1.00 (1 H, m), 0.91 (3 H, d, J = 6.8 Hz), 0.62 (6 H, dd, J = 10.0, 6.9 Hz) ppm.

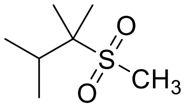

7b: ^1H NMR (300 MHz, C_6D_6): 2.75–2.50 (1 H, m), 2.21 (3 H, s), 2.11–1.87 (2 H, m), 1.58–1.01 (12 H, m) ppm.

^{13}C NMR (75 MHz, C_6D_6, mixture of regioisomers): 64.6, 58.6, 41.3, 35.8, 33.7, 32.5, 32.1, 19.3, 19.0, 18.8, 17.9, 16.0 ppm. – IR: 2965, 1470, 1385, 1285, 1135, 1110, 958, 776, 587 cm^{-1}. – HRMS calcd for $C_7H_{17}O_2S$: 165.0944, found 165.0944 [M+H$^+$].

Go back to table of contents

Photoinduced C–H sulfination of Pinacolone: 3,3-dimethyl-4-(methylsulfonyl)butan-2-one (8)

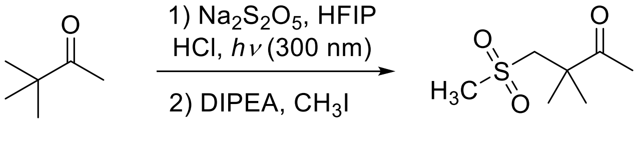

According to GP2, a stirred mixture of hexafluoroisopropanol (3 mL) and $Na_2S_2O_5$ (570 mg, 3 mmol) was degassed with Ar for 5 min in a quartz test-tube. Pinacolone (20 mg, 0.2 mmol) and 12M aqueous HCl (0.25 mL) were then added. The solution was stirred vigorously and irradiated at 25 °C for 16 h in a Rayonet RPR-100 photochemical reactor. The reaction mixture was transferred into a pressure tube, *N,N*-diisopropylethylamine (0.5 mL, 3 mmol) and iodomethane (0.1 mL, 2 mmol) were added, and the reaction mixture was stirred at 60 °C for 4 h according to GP4. The reaction mixture was then extracted with EtOAc (3 × 15 mL). The organic phases were combined, dried over anhydrous Na_2SO_4 and concentrated under reduced pressure. The crude product was purified by flash chromatography on silica gel (EtOAc/hexane, 1 : 5 v/v) to give sulfone **8** (14 mg, 40%) as a colorless oil.

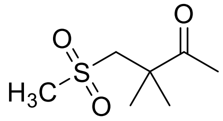

^{1}H NMR (300 MHz, $CDCl_3$): 3.41 (s, 2H), 2.94 (s, 3H), 2.25 (s, 3H), 1.41 (s, 6H) ppm. – ^{13}C NMR (75 MHz, $CDCl_3$): 211.4, 63.1, 47.0, 44.4, 25.6, 25.4 ppm. – IR: 2929, 1708, 1365, 1304, 1217, 1134, 763, 596, 519 cm^{-1}. – HRMS calcd for C7H15O3S: 179.0736, found 179.0739 [M+H$^+$].

Photoinduced C–H sulfination of pentan-3-one: 1-(allylsulfonyl)pentan-3-one (9)

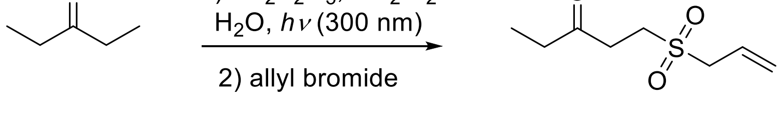

Go back to table of contents

According to GP1, a stirred mixture of dichloromethane (3 mL) and $Na_2S_2O_5$ (152 mg, 0.8 mmol) was degassed with Ar for 5 min in a quartz test-tube. Pentan-3-one (17 mg, 0.2 mmol) and water (0.75 mL, degassed prior to use) were then added. The solution was stirred vigorously and irradiated at 25 °C for 16 h in a Rayonet RPR-100 photochemical reactor. The reaction mixture was transferred into a pressure tube, allyl bromide (0.2 mL, 2 mmol) was added, and the reaction mixture was stirred at 60 °C for 4 h according to GP3. The reaction mixture was then extracted with EtOAc (3 × 15 mL). The organic phases were combined, dried over anhydrous Na_2SO_4 and concentrated under reduced pressure. The crude product was purified by flash chromatography on silica gel (EtOAc/hexane, 1 : 5 v/v) to give sulfone **9** (21 mg, 55%) as a colorless oil.

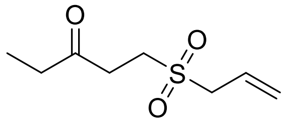

^{1}H NMR (500 MHz, $CDCl_3$): 5.94 (1 H, ddt, *J* = 17.6, 10.3, 7.4 Hz), 5.55–5.38 (2 H, m), 3.74 (2 H, d, *J* = 7.4 Hz), 3.27 (2 H, t, *J* = 7.3 Hz), 2.97 (2 H, t, *J* = 7.3 Hz), 2.52 (2 H, q, *J* = 7.3 Hz), 1.09 (3 H, t, *J* = 7.3 Hz) ppm. – ^{13}C NMR (125 MHz, $CDCl_3$): 207.2, 125.2, 124.9, 58.8, 45.8, 36.2, 33.8, 7.8 ppm. – IR: 3337, 2935, 1716, 1640, 1460, 1416, 1370, 1320, 1276, 1244, 1127, 989, 925, 795, 665, 631, 530 cm^{-1}. – HRMS calcd for $C_8H_{15}O_3S$: 191.0736, found 191.0737 [M+H$^+$].

Photoinduced C–H sulfination of pentan-2-one: sulfone 10a,b

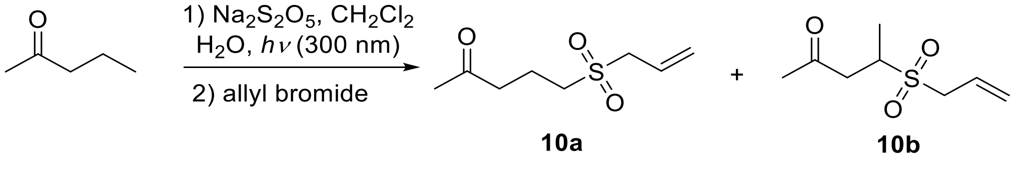

According to GP1, a stirred mixture of dichloromethane (3 mL) and $Na_2S_2O_5$ (152 mg, 0.8 mmol) was degassed with Ar for 5 min in a quartz test-tube. Pentan-2-one (17 mg, 0.2 mmol) and water (1.5 mL, degassed prior to use) were then added. The solution was stirred vigorously and irradiated at 25 °C for 16 h in a Rayonet RPR-100 photochemical

Go back to table of contents

reactor. The reaction mixture was transferred into a pressure tube, allyl bromide (0.2 mL, 2 mmol) was added, and the reaction mixture was stirred at 60 °C for 4 h according to GP3. The reaction mixture was then extracted with EtOAc (3 × 15 mL). The organic phases were combined, dried over anhydrous Na_2SO_4 and concentrated under reduced pressure. The crude product was purified by flash chromatography on silica gel (EtOAc/hexane, 1 : 5 v/v) to give two separable regioisomers (32 mg, 85%, 1 : 3 ratio of sulfones **10a** and **10b**) as a colorless oil.

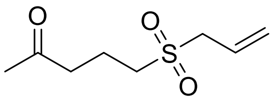

10a: ^{1}H NMR (500 MHz, $CDCl_3$): 6.02–5.83 (1 H, m), 5.56–5.38 (2 H, m), 3.70 (2 H, d, *J* = 7.4 Hz), 2.99 (2 H, t, *J* = 6.7 Hz), 2.68 (2 H, t, *J* = 6.7 Hz), 2.15 (3 H, s), 2.13–2.01 (2 H, m) ppm. – ^{13}C NMR (125 MHz, $CDCl_3$): 207.2, 125.1, 124.9, 57.8, 50.1, 41.2, 30.1, 16.3 ppm. – IR: 3583, 3004, 1708, 1421, 1358, 1220, 1092, 900, 678, 627, 528 cm^{-1}. – HRMS calcd for $C_8H_{15}O_3S$: 191.0736, found 191.074 [M+H$^+$].

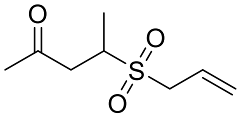

10b: ^{1}H NMR (500 MHz, $CDCl_3$): 6.00–5.82 (1 H, m), 5.52–5.41 (2 H, m), 3.79–3.62 (3 H, m), 3.19 (1 H, dd, *J* = 18.3, 3.9 Hz), 2.60 1 H, (dd, *J* = 18.3, 8.8 Hz), 2.20 (3 H, s), 1.35 (3 H, d, *J* = 6.9 Hz) ppm. – ^{13}C NMR (125 MHz, $CDCl_3$): 204.2, 124.9, 124.6, 55.6, 51.6, 41.8, 30.5, 14.5 ppm. – IR: 3003, 2357, 1709, 1421, 1358, 1220, 1092, 901, 685, 629, 529 cm^{-1}. – HRMS calcd for $C_8H_{15}O_3S$: 191.0736, found 191.0738 [M+H$^+$].

Photoinduced C–H sulfination of hexan-2-one: sulfone 11a-c

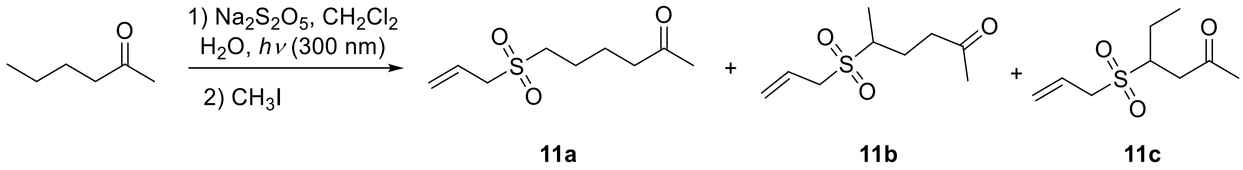

According to GP1, a stirred mixture of dichloromethane (3 mL) and $Na_2S_2O_5$ (114 mg, 0.6 mmol) was degassed with Ar for 5 min in a quartz test-tube. Hexan-2-one (20 mg, 0.2 mmol) and water (0.75 mL, degassed prior to use) were then added. The solution was stirred vigorously and irradiated at 25 °C for 16 h in a Rayonet RPR-100 photochemical reactor. The reaction mixture was transferred into a pressure tube, allyl-bromide (0.2 mL,

Go back to table of contents

2 mmol) was added, and the reaction mixture was stirred at 60 °C for 4 h according to GP3. The reaction mixture was then extracted with EtOAc (3 × 15 mL). The organic phases were combined, dried over anhydrous Na_2SO_4 and concentrated under reduced pressure. The crude product was purified by flash chromatography on silica gel (EtOAc/hexane, 1 : 5 v/v) to give three separable regioisomers (33 mg, 80%, 1: 12 : 2 ratio of isomers **11a**, **11b**, and **11c**) as a colorless oil.

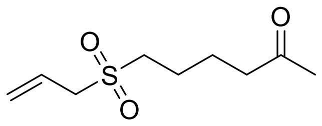

11a: ^{1}H NMR (500 MHz, $CDCl_3$): 6.02–5.81 (1 H, m), 5.58–5.37 (2 H, m), 3.70 (2 H, d, *J* = 7.4 Hz), 3.03–2.85 (2 H, m), 2.49 (2 H, t, *J* = 7.0 Hz), 2.14 (3 H, s), 1.87–1.78 (2 H, m), 1.74–1.66 (2 H, m) ppm. – ^{13}C NMR (125 MHz, $CDCl_3$): 207.8, 77.4, 77.2, 76.9, 57.9, 51.1, 42.8, 30.1, 22.5, 21.4 ppm. – IR: 3004, 1709, 1421, 1358, 1220, 1092, 920, 735, 647, 625, 528 cm^{-1}. – HRMS calcd for $C_9H_{17}O_3S$: 205.0893, found 205.0894 [M+H$^+$].

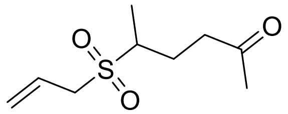

11b: ^{1}H NMR (500 MHz, $CDCl_3$): 5.89 (1 H, ddt, *J* = 17.5, 10.4, 7.4 Hz), 5.51 – 5.34 (2 H, m), 3.71 (2 H, d, *J* = 7.3 Hz), 3.19 – 3.00 (1 H, m), 2.73 (1 H, dt, *J* = 18.3, 6.9 Hz), 2.58 (1 H, dt, *J* = 18.3, 7.2 Hz), 2.24 – 2.15 (1 H, m), 2.13 (3 H, s), 1.82 (1 H, td, *J* = 14.2, 7.5 Hz), 1.32 (3 H, d, *J* = 7.0 Hz) ppm. – ^{13}C NMR (125 MHz, $CDCl_3$): 207.3, 124.7, 124.6, 55.16, 54.9, 39.7, 30.1, 23.3, 13.2 ppm. – IR: 3599, 3005, 1708, 1421, 1359, 1221, 1133, 1092, 920, 734, 625, 528 cm^{-1}. – HRMS calcd for $C_9H_{17}O_3S$: 205.0893, found 205.0896 [M+H$^+$].

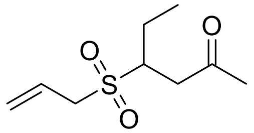

11c: ^{1}H NMR (500 MHz, CDCl3): 5.97–5.83 (1 H, m), 5.47 (2 H, ddd, *J* = 18.2, 13.6, 0.8 Hz), 3.70 (2 H, d, *J* = 7.3 Hz), 3.68–3.60 (1 H, m), 3.16 (1 H, dd, *J* = 18.5, 6.0 Hz), 2.62 (1 H, dd, *J* = 18.5, 5.8 Hz), 2.23 (3 H, s), 1.97 (1 H, ddd, *J* = 14.4, 7.5, 4.7 Hz), 1.72–1.60 (1 H, m), 0.99 (3 H, t, *J* = 7.5 Hz) ppm. – ^{13}C NMR (125 MHz, $CDCl_3$): 204.5, 124.9, 124.7, 57.0, 40.7, 30.3, 22.2, 11.1 ppm. – IR: 3005, 1709, 1419, 1359, 1220, 1092, 820, 735, 648, 626, 528 cm^{-1}. – HRMS calcd for $C_9H_{17}O_3S$: 205.0893, found 205.0895 [M+H$^+$].

Go back to table of contents

Photoinduced C–H sulfination of 5-methylhexan-2-one: sulfone 12a-c

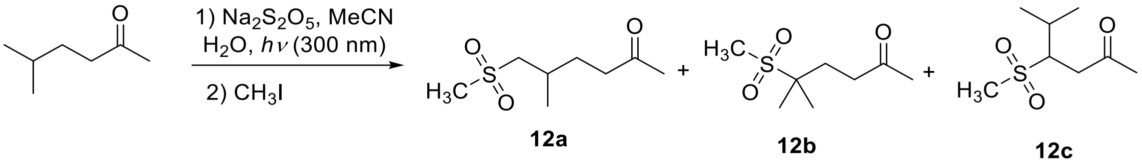

According to GP1, a stirred mixture of MeCN (3 mL) and $Na_2S_2O_5$ (57 mg, 0.3 mmol) was degassed with Ar for 5 min in a quartz test-tube. 5-Methylhexan-2-one (23 mg, 0.2 mmol) and water (0.75 mL, degassed prior to use) were then added. The solution was stirred vigorously and irradiated at 25 °C for 16 h in a Rayonet RPR-100 photochemical reactor. The reaction mixture was transferred into a pressure tube, iodomethane (0.1 mL, 2 mmol) was added, and the reaction mixture was stirred at 60 °C for 4 h according to GP3. The reaction mixture was then extracted with EtOAc (3 × 15 mL). The organic phases were combined, dried over anhydrous Na_2SO_4 and concentrated under reduced pressure. The crude product was purified by flash chromatography on silica gel (EtOAc/hexane, 1 : 5 v/v) to give three separable regioisomers (30 mg, 78%, 1: 13 : 2 ratio of isomers **12a**, **12b**, and **12c**) as a colorless oil.

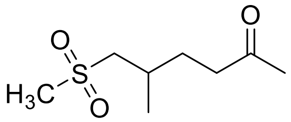

12a: ^{1}H NMR (500 MHz, $CDCl_3$): 3.00 (1 H, dd, *J* = 14.0, 5.1 Hz), 2.92 (3 H, s), 2.88 (1 H, dd, *J* = 14.1, 7.5 Hz), 2.58 – 2.42 (2 H, m), 2.26 – 2.18 (1 H, m), 2.16 (2 H, s), 1.84 (1 H, ddd, *J* = 14.2, 8.6, 6.1 Hz), 1.64 – 1.53 (2 H, m), 1.14 (3 H, d, *J* = 6.7 Hz) ppm. – ^{13}C NMR (125 MHz, $CDCl_3$): 208.2, 61.0, 42.0, 40.7, 30.4, 30.1, 28.1, 20.1 ppm. – IR: 3005, 1709, 1419, 1359, 1220, 1092, 919, 735, 629, 529 cm^{-1}. – HRMS calcd for $C_8H_{17}O_3S$: 193.0893, found 193.0894 [M+H$^+$].

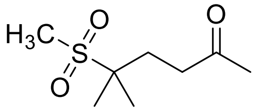

12b: ^{1}H NMR (500 MHz, $CDCl_3$): 2.72 (3 H, s), 2.57 (2 H, t, , *J* = 10.0 Hz), 2.08 (3 H, s), 1.94 (2 H, t, , *J* = 10.0 Hz), 1.27 (6 H, s) ppm. – ^{13}C NMR (125 MHz, $CDCl_3$): 207.1, 60.6, 38.0, 34.6, 30.0, 29.2, 21.0 ppm. – IR: 2935, 1713, 1417,

Go back to table of contents

1369, 1282, 1173, 1109, 958, 631, 533 cm^{-1}. – HRMS calcd for $C_8H_{17}O_3S$: 193.0893, found 193.0894 [M+H$^+$].

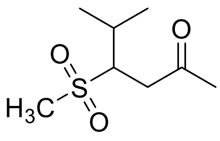

12c: ^{1}H NMR (500 MHz, $CDCl_3$): 3.64–3.57 (1 H, m), 3.10 (1 H, dd, *J* = 19.0, 7.7 Hz), 2.81 (3 H, s), 2.62 (1 H, dd, *J* = 19.0, 3.4 Hz), 2.53 (1 H, dtd, *J* = 13.7, 6.9, 3.0 Hz), 2.24 (3 H, s), 0.98 (6 H, dd, *J* = 16.4, 6.9 Hz) ppm. – ^{13}C NMR (125 MHz, $CDCl_3$): 205.1, 62.8, 41.6, 37.5, 30.2, 26.8, 21.4, 17.7 ppm. – IR: 2967, 1717, 1365, 1295, 1123, 803, 753, 630, 530 cm^{-1}. – HRMS calcd for $C_8H_{17}O_3S$: 193.0893, found 193.0894 [M+H$^+$].

Photoinduced C–H sulfination of 2,6-dimethylheptan-4-one: sulfone 13a,b

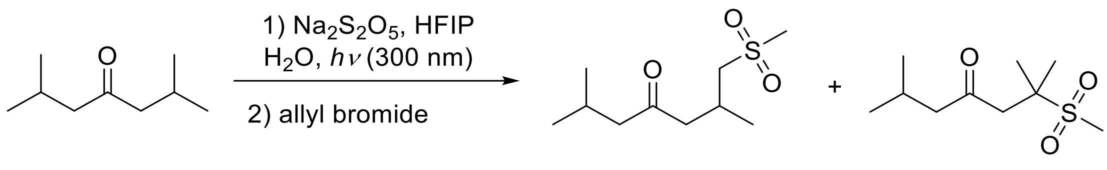

13a **13b**

According to GP1, a stirred mixture of hexafluoroisopropanol (3 mL) and $Na_2S_2O_5$ (76 mg, 0.4 mmol) was degassed with Ar for 5 min in a quartz test-tube. 2,6-Dimethylheptan-4-one (28 mg, 0.2 mmol) and water (0.75 mL, degassed prior to use) were then added. The solution was stirred vigorously and irradiated at 25 °C for 16 h in a Rayonet RPR-100 photochemical reactor. The reaction mixture was transferred into a pressure tube, iodomethane (0.1 mL, 2 mmol) was added, and the reaction mixture was stirred at 60 °C for 4 h according to GP3. The reaction mixture was then extracted with EtOAc (3 × 15 mL). The organic phases were combined, dried over anhydrous Na_2SO_4 and concentrated under reduced pressure. The crude product was purified by flash chromatography on silica gel (EtOAc/hexane, 1 : 5 v/v) to give two separable regioisomers (33 mg, 76%, 1 : 8.5 ratio of sulfones **13a** and **13b**) as a colorless oil.

Go back to table of contents

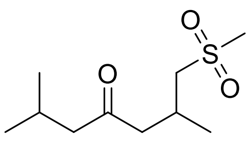

13a: ^{1}H NMR (500 MHz, $CDCl_3$): 3.09 (1 H, dd, *J* = 14.2, 5.5 Hz), 2.93 (3 H, s), 2.87 (1 H, dd, *J* = 14.1, 6.7 Hz), 2.71–2.58 (2 H, m), 2.47 (1 H, dd, *J* = 17.1, 5.1 Hz), 2.25 (2 H, d, *J* = 7.0 Hz), 2.10 (1 H, dp, *J* = 13.5, 6.6 Hz), 1.15 (3 H, d, *J* = 6.7 Hz), 0.88 (6 H, d, *J* = 6.7 Hz) ppm. – ^{13}C NMR (125 MHz, $CDCl_3$): 209.3, 59.7, 52.2, 48.7, 41.5, 24.8, 24.7, 22.6, 20.5 ppm. – IR: 3020, 1708, 1306, 1214, 1133, 908, 748, 670, 650, 633, 531 cm^{-1}. – HRMS calcd for $C_{10}H_{21}O_3S$: 221.1206, found 221.1208 [M+H$^+$].

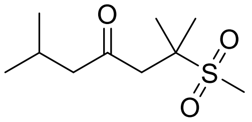

13b: ^{1}H NMR 2.86 (2 H, s), 2.82 (3 H, s), 2.32 (2 H, d, *J* = 6.9 Hz), 2.12 (1 H, dt, *J* = 13.5, 6.7 Hz), 1.54 (6 H, s), 0.91 (6 H, d, *J* = 6.7 Hz) ppm. – ^{13}C NMR (125 MHz, $CDCl_3$): 206.9, 61.3, 53.6, 44.8, 34.4, 24.6, 22.4, 20.4 ppm. – IR: 3019, 1214, 908, 749, 670, 651, 632, 535 cm^{-1}. – HRMS calcd for $C_{10}H_{21}O_3S$: 221.1206, found 221.1208 [M+H$^+$].

Photoinduced C–H sulfination of adamantan-2-one: sulfone 14a-d

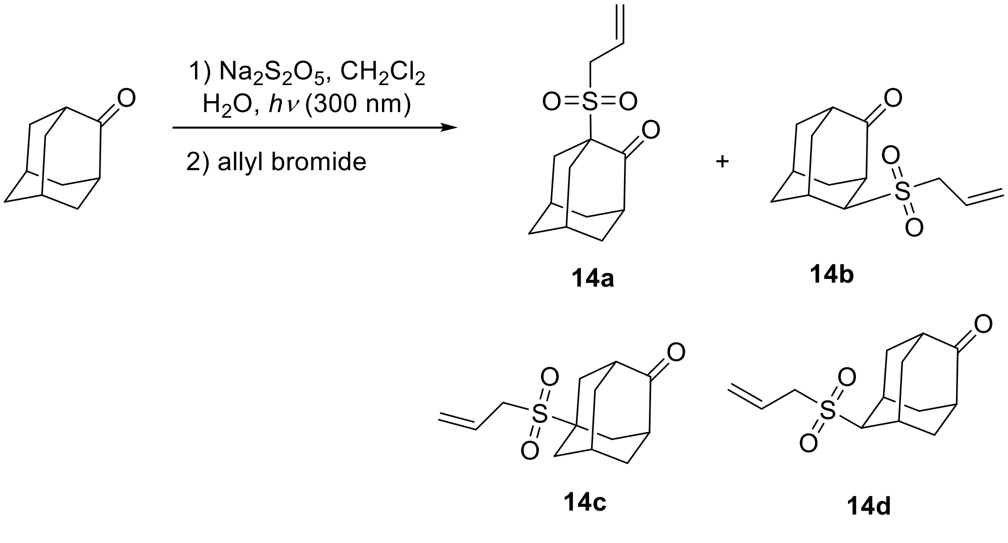

Go back to table of contents

According to GP1, a stirred mixture of dichloromethane (3 mL) and $Na_2S_2O_5$ (190 mg, 1 mmol) was degassed with Ar for 5 min in a quartz test-tube. Adamantan-2-one (30 mg, 0.2 mmol) and water (0.75 mL, degassed prior to use) were then added. The solution was stirred vigorously and irradiated at 25 °C for 16 h in a Rayonet RPR-100 photochemical reactor. The reaction mixture was transferred into a pressure tube, allyl bromide (0.2 mL, 2 mmol) were added, and the reaction mixture was stirred at 60 °C for 4 h according to GP3. The reaction mixture was then extracted with EtOAc (3 × 15 mL). The organic phases were combined, dried over anhydrous Na_2SO_4 and concentrated under reduced pressure. The crude product was purified by flash chromatography on silica gel (EtOAc/hexane, 1:3 v/v) to give four separable regioisomers (35 mg, 70%, 36.7 : 8.8 : 5.3 : 1 ratio of sulfones **14a**, **14b**, **14c**, and **14d**) as a colorless solid (**14a**) and colorless liquids (**14b-c**).

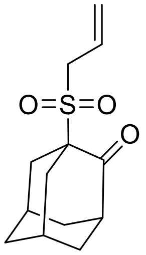

14a: m.p. 78–80 ºC. – ^{1}H NMR (500 MHz, $CDCl_3$): 6.02–5.86 (1 H, m), 5.50–5.42 (2 H, m), 4.02 (2 H, d, *J* = 7.4 Hz), 2.71 (1 H, s), 2.46 (2 H, d, *J* = 12.6 Hz), 2.37–2.27 (4 H, m), 2.10–1.97 (4 H, m), 1.93 (2 H, s) ppm. – ^{13}C NMR (125 MHz, $CDCl_3$): 208.7, 124.9, 123.8, 72.2, 55.3, 47.7, 38.3, 37.7, 34.7, 27.7 ppm. – IR: 2922, 2852, 1737, 1721, 1454, 1365, 1216, 1139, 629, 535 cm^{-1}. – HRMS calcd for $C_{13}H_{18}O_3S$: 255.1049, found 255.1051 [M+H$^+$].

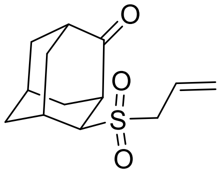

14b: ^{1}H NMR (500 MHz, $CDCl_3$): 5.95 (1 H, dddd, *J* = 16.9, 10.1, 8.2, 6.6 Hz,), 5.47 (2 H, ddd, *J* = 18.2, 13.6, 0.8 Hz), 3.76 (2 H, ddd, *J* = 20.8, 14.2, 7.4 Hz), 3.60 (1 H, d, *J* = 2.0 Hz), 2.97 (1 H, s), 2.80–2.63 (3 H, m), 2.24–1.90 (8 H, m) ppm. – ^{13}C NMR (125 MHz, $CDCl_3$): 212.2, 125.3, 124.7, 67.1, 56.8, 46.6, 45.9, 40.7, 40.3, 38.0, 33.5, 27.2, 26.9 ppm. – IR: 2926, 2857, 1726, 1454, 1318, 1216, 1131, 627 cm^{-1}. – HRMS calcd for $C_{13}H_{18}O_3S$: 255.1049, found 215.1049 [M+H$^+$].

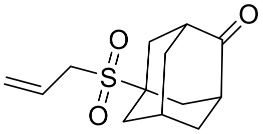

14c: ^{1}H NMR (500 MHz, $CDCl_3$): 5.94 (1 H, td, *J* = 17.1, 7.3 Hz), 5.54–5.42 (2 H, m), 3.71 (2 H, d, *J* = 7.1 Hz), 2.72 (2 H, s), 2.45–2.25 (8 H, m), 2.13–1.97 (4 H, m) ppm. – ^{13}C NMR (125 MHz, $CDCl_3$): 214.0,

125.0, 124.0, 60.3, 51.8, 45.5, 37.9, 36.7, 34.2, 27.7 ppm. – IR: 2920, 2855, 1725, 1456, 1365, 1289, 1216, 1135, 634 cm^{-1}. – HRMS calcd for $C_{13}H_{18}O_3S$: 255.1049, found 255.1052 [M+H$^+$].

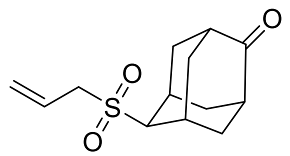

14d: ^{1}H NMR (500 MHz, $CDCl_3$): 6.08–5.84 (1 H, m), 5.49 (2 H, dd, *J* = 26.3, 13.7 Hz), 3.80 (2 H, d, *J* = 7.4 Hz), 3.37 (1 H, s), 2.84 (2 H, d, *J* = 13.7 Hz), 2.64–2.52 (4 H, m), 2.19 (2 H, d, *J* = 13.7 Hz), 2.07–1.99 (2 H, m), 1.97–1.84 (2 H, m) ppm. – ^{13}C NMR (125 MHz, $CDCl_3$): 215.4, 125.7, 124.6, 62.4, 57.4, 45.6, 45.4, 39.9, 33.4, 27.4 ppm. – IR: 2918, 2853, 1713, 1363, 1223, 914, 732, 648 cm^{-1}. – HRMS calcd for $C_{13}H_{18}O_3S$: 255.1049, found 255.1050 [M+H$^+$].

Photoinduced C–H sulfination of methyl butyrate: sulfone 15a,b

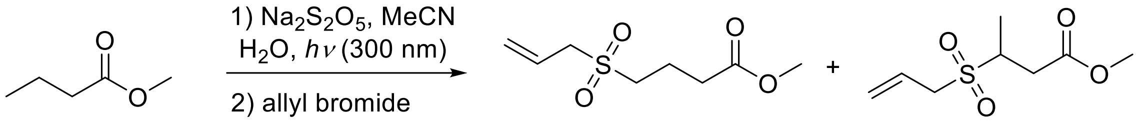

According to GP1, a stirred mixture of MeCN (3 mL) and $Na_2S_2O_5$ (38 mg, 0.2 mmol) was degassed with Ar for 5 min in a quartz test-tube. Methyl butyrate (20 mg, 0.2 mmol) and water (0.75 mL, degassed prior to use) were then added. The solution was stirred vigorously and irradiated at 25 °C for 16 h in a Rayonet RPR-100 photochemical reactor. The reaction mixture was transferred into a pressure tube, allyl bromide (0.2 mL, 2 mmol) was added, and the reaction mixture was stirred at 60 °C for 4 h according to GP3. The reaction mixture was then extracted with EtOAc (3 × 15 mL). The organic phases were combined, dried over anhydrous Na_2SO_4 and concentrated under reduced pressure. The crude product was purified by flash chromatography on silica gel (EtOAc/hexane, 1 : 5 v/v) to give two separable regioisomers (28 mg, 68%, 1 : 3.5 ratio of isomers **15a** and **15b**) as a colorless oil.

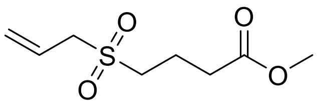

15a: ^{1}H NMR (500 MHz, $CDCl_3$): 5.94 (1 H, ddt, *J* = 17.4, 10.1, 7.4 Hz), 5.48 (2 H, dd, *J* = 17.3, 13.8 Hz), 3.72 (2 H, d, *J* = 7.5 Hz), 3.69 (3 H, s), 3.11–3.00 (2 H, m), 2.53 (2 H, t, *J* = 7.0 Hz), 2.18–2.08 (2 H, m) ppm. – ^{13}C NMR

Go back to table of contents

(125 MHz, $CDCl_3$): 172.7, 125.1, 124.9, 57.9, 52.0, 50.2, 32.2, 17.6 ppm. – IR: 2920, 1732, 1438, 1312, 1124, 891, 711, 631, 534 cm^{-1}. – HRMS calcd for $C_8H_{15}O_4S$: 207.0686, found 207.0687 [$M+H^+$].

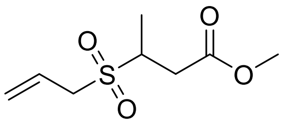

15b: ^1H NMR (500 MHz, $CDCl_3$): 5.93 (1 H, ddt, *J* = 17.4, 10.1, 7.4 Hz), 5.49 (2 H, dd, *J* = 18.7, 13.7 Hz), 3.84 – 3.66 (5 H, m), 3.60 (1 H, ddd, *J* = 9.4, 6.9, 4.4 Hz), 3.06 (1 H, dd, *J* = 16.7, 4.3 Hz), 2.50 (1 H, dd, *J* = 16.7, 9.3 Hz), 1.43 (3 H, d, *J* = 6.9 Hz) ppm. – ^{13}C NMR (125 MHz, $CDCl_3$): 170.8, 77.4, 77.2, 76.9, 55.6, 52.8, 52.5, 33.7, 14.2 ppm. – IR: 3504, 3005, 1706, 1422, 1362, 1223, 1134, 1092, 657, 615, 526 cm^{-1}. – HRMS calcd for $C_8H_{15}O_4S$: 207.0686, found 207.0687 [$M+H^+$].

Photoinduced C–H sulfination of ethyl cyclobutanecarboxylate: sulfone 16a,b

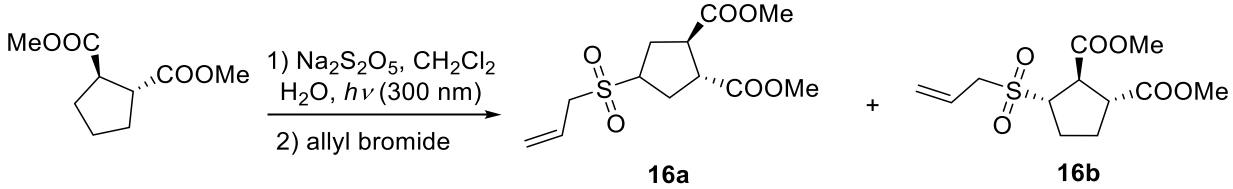

According to GP1, a stirred mixture of dichloromethane (4 mL) and $Na_2S_2O_5$ (380 mg, 2 mmol) was degassed with Ar for 5 min in a quartz test-tube. Ethyl cyclobutanecarboxylate (37 mg, 0.2 mmol) and water (1.5 mL, degassed prior to use) were then added. The solution was stirred vigorously and irradiated at 25 °C for 16 h in a Rayonet RPR-100 photochemical reactor. The reaction mixture was transferred into a pressure tube, allyl bromide (0.2 mL, 2 mmol) was added, and the reaction mixture was stirred at 60 °C for 4 h according to GP3. The reaction mixture was then extracted with EtOAc (3 × 15 mL). The organic phases were combined, dried over anhydrous Na_2SO_4 and concentrated under reduced pressure. The crude product was purified by flash chromatography on silica gel (EtOAc/hexane, 1 : 5 v/v) to give two separable regioisomers (43 mg, 75%, 1 : 1.3 ratio of sulfones **16a** and **16b**) as a colorless oil.

Go back to table of contents

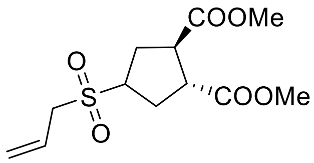

16a: ^{1}H NMR (500 MHz, $CDCl_3$): 5.96–5.81 (1 H, m), 5.46 (2 H, dd, *J* = 21.6, 13.6 Hz), 3.74–3.66 (8 H, m), 3.66–3.56 (1 H, m), 3.36 (1 H, dd, *J* = 16.6, 8.6 Hz), 3.19 (1 H, dd, *J* = 18.3, 8.5 Hz), 2.57–2.29 (3 H, m), 2.24 (1 H, dt, *J* = 14.2, 8.8 Hz) ppm. – ^{13}C NMR (125 MHz, $CDCl_3$): 173.8, 172.6, 124.8, 58.1, 56.9, 52.5, 52.5, 47.0, 45.9, 30.0, 29.7 ppm. – IR: 3019, 1732, 1214, 1133, 908, 750, 733, 670, 651, 632, 534 cm^{-1}. – HRMS calcd for $C_{12}H_{19}O_6S$: 291.0897, found 291.0909 [M+H+].

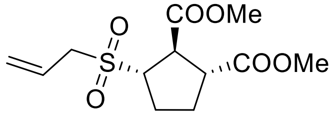

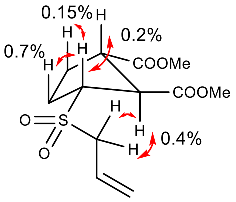

16b: ^{1}H NMR (500 MHz, $CDCl_3$): 5.89 (1 H, dq, *J* = 10.0, 7.4 Hz), 5.44 (2 H, dd, *J* = 19.7, 13.8 Hz), 3.92–3.81 (1 H, m), 3.82–3.62 (9 H, m), 3.10 (1 H, q, *J* = 8.3 Hz), 2.36 (1 H, dd, *J* = 5.0, 1.4 Hz), 2.14–2.00 (3 H, m) ppm. – ^{13}C NMR (125 MHz, $CDCl_3$): 172.8, 172.6, 124.9, 124.6, 62.3, 56.8, 52.9, 52.5, 49.0, 47.8, 29.8, 26.7 ppm. – IR: 2954, 1728, 1640, 1437, 1640, 1437, 1377, 1291, 1265, 1223, 1199, 1173, 1128, 1083, 997, 939, 878, 792, 633, 530 cm^{-1}. – HRMS calcd for $C_{12}H_{19}O_6S$: 291.0897, found 291.0905 [M+H+].

Photoinduced C–H sulfination of isobutyl acetate: sulfone 17a-c

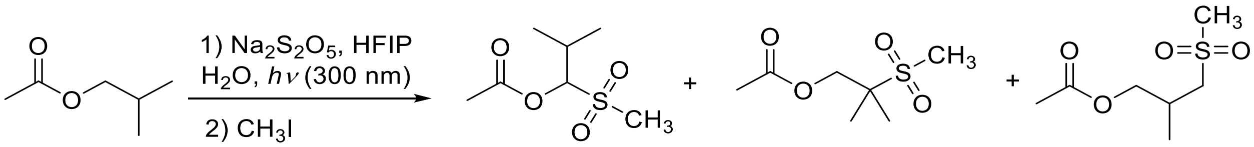

17a **17b** **17c**

According to GP1, a stirred mixture of hexafluoroisopropanol (3 mL) and $Na_2S_2O_5$ (57 mg, 0.3 mmol) was degassed with Ar for 5 min in a quartz test-tube. Isobutyl acetate (23 mg, 0.2 mmol) and water (0.75 mL, degassed prior to use) were then added. The solution was stirred vigorously and irradiated at 25 °C for 16 h in a Rayonet RPR-100 photochemical reactor. The reaction mixture was transferred into a pressure tube, iodomethane (0.1 mL, 2 mmol) was added, and the reaction mixture was stirred at 60 °C for 4 h according to GP3. The reaction mixture was then extracted with EtOAc (3 × 15 mL). The organic phases

Go back to table of contents

were combined, dried over anhydrous Na_2SO_4 and concentrated under reduced pressure to give regioisomers **17a-c** (26 mg, 66%, 16 : 8 : 1 ratio of isomers **17b**, **17c**, and **17a**) as a colorless oil.

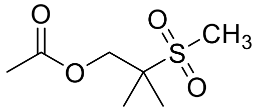

^{1}H NMR (300 MHz, C_6D_6): 4.09 (2 H, s), 2.34 (3 H, s), 1.59 (3 H, d, J = 3.1 Hz), 1.03 (6 H, s) ppm.

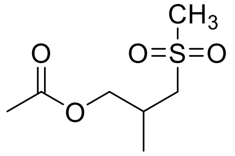

^{1}H NMR (300 MHz, C6D6): 3.88–3.71 (2 H, m), 2.71–2.55 (1 H, m), 2.46–2.36 (1 H, m), 2.22 (3 H, s), 1.67 (3 H, s), 1.39–1.22 (1 H, m), 0.88 (3 H, dd, J = 11.9, 5.0 Hz) ppm.

^{13}C NMR (75 MHz, C_6D_6, mixture of isomers): 170.0, 169.3, 169.1, 88.5, 67.5, 66.8, 60.8, 57.3, 41.3, 39.2, 37.2, 28.5, 28.0, 20.4, 20.2, 19.8, 19.7, 18.3, 17.3, 17.0.ppm. – IR: 3529, 2934, 1743, 1709, 1645, 1368, 1291, 1231, 1117, 1046, 957, 764, 645, 597, 528 cm^{-1}. – HRMS calcd for $C_7H_{15}O_4S$: 195.0686, found 195.0685 [M+H$^+$].

Photoinduced C–H sulfination of pentanenitrile: sulfone 18a,b

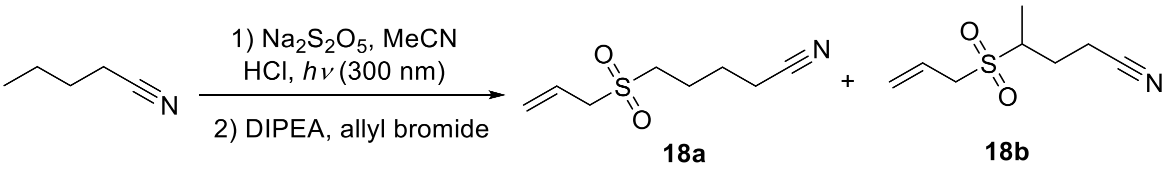

According to GP2, a stirred mixture of MeCN (5 mL) and $Na_2S_2O_5$ (570 mg, 3 mmol) was degassed with Ar for 5 min in a quartz test-tube. Pentanenitrile (21 mg, 0.25 mmol) and 12M aqueous HCl (0.25 mL) were then added. The solution was stirred vigorously and irradiated at 25 °C for 16 h in a Rayonet RPR-100 photochemical reactor. The reaction mixture was transferred into a pressure tube, *N,N*-diisopropylethylamine (0.5 mL, 3 mmol) and allyl bromide (0.2 mL, 2 mmol) were added, and the reaction mixture was stirred at 60 °C for 4 h according to GP4. The reaction mixture was then extracted with EtOAc (3 × 15 mL). The organic phases were combined, dried over anhydrous Na_2SO_4 and concentrated under reduced pressure. The crude product was purified by flash

Go back to table of contents

chromatography on silica gel (EtOAc/hexane, 1 : 5 v/v) to give two separable regioisomers (28 mg, 60%, 2 : 1 ratio of isomers **18b** and **18a**) as colorless oils.

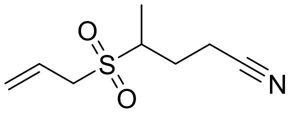

^{1}H NMR (500 MHz, $CDCl_3$): 6.04 – 5.83 (1 H, m), 5.51 (2 H, ddd, *J* = 17.9, 13.6, 0.6 Hz), 3.75 (2 H, qd, *J* = 14.2, 7.4 Hz), 3.24 (1 H, dd, *J* = 13.2, 6.9 Hz), 2.71 (1 H, dt, *J* = 17.1, 7.1 Hz), 2.54 (1 H, dt, *J* = 17.1, 7.3 Hz), 2.41 – 2.29 (1 H, m), 1.94 (1 H, dt, *J* = 14.4, 7.1 Hz), 1.42 (3 H, d, *J* = 6.9 Hz) ppm. – ^{13}C NMR (125 MHz, $CDCl_3$): 125.2, 124.5, 118.5, 55.7, 54.0, 25.1, 15.1, 13.7 ppm. – IR: 3565, 2922, 2244, 1706, 1640, 1423, 1361, 1310, 1222, 1132, 1093, 994, 941, 631, 529 cm^{-1}. – HRMS calcd for $C_8H_{14}NO_2S$: 188.074, found 188.0741 [M+H$^+$].

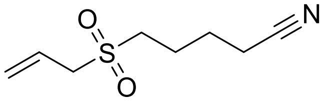

^{1}H NMR (500 MHz, $CDCl_3$): 5.95 (1 H, ddt, *J* = 14.9, 10.1, 7.4 Hz), 5.50 (2 H, dd, *J* = 22.9, 13.6 Hz), 3.73 (2 H, d, *J* = 7.4 Hz), 3.01 (2 H, t, *J* = 7.5 Hz), 2.43 (2 H, t, *J* = 7.0 Hz), 2.02 (2 H, dt, *J* = 15.3, 7.6 Hz), 1.87 (2 H, dt, *J* = 14.4, 7.0 Hz) ppm. – ^{13}C NMR (125 MHz, $CDCl_3$): 125.1, 125.1, 118.9, 58.2, 50.0, 24.3, 21.1, 17.1 ppm. – IR: 3004, 1708, 1422, 1360, 1221, 1127, 1092, 900, 630, 529 cm^{-1}. – HRMS calcd for $C_8H_{14}NO_2S$: 188.074, found 188.0741 [M+H$^+$].

Photoinduced C–H sulfination of cyclopentanecarbonitrile: sulfone 19a-c

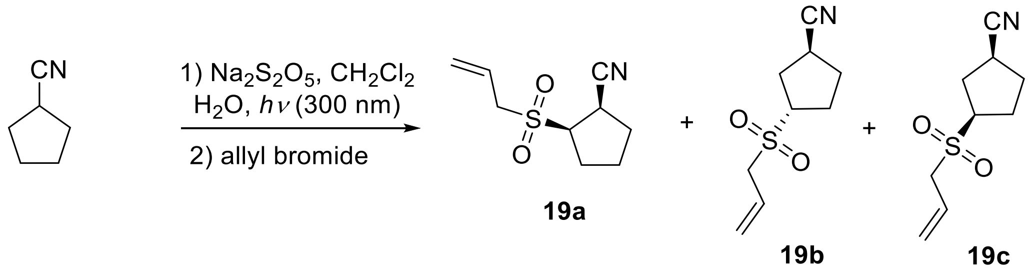

According to GP1, a stirred mixture of dichloromethane (4 mL) and $Na_2S_2O_5$ (380 mg, 2 mmol) was degassed with Ar for 5 min in a quartz test-tube. Cyclopentanecarbonitrile (19 mg, 0.2 mmol) and water (1.5 mL, degassed prior to use) were then added. The solution was stirred vigorously and irradiated at 25 °C for 16 h in a Rayonet RPR-100 photochemical reactor. The reaction mixture was transferred into a pressure tube, allyl

Go back to table of contents

bromide (0.2 mL, 2 mmol) was added, and the reaction mixture was stirred at 60 °C for 4 h according to GP3. The reaction mixture was then extracted with EtOAc (3 × 15 mL). The organic phases were combined, dried over anhydrous Na_2SO_4 and concentrated under reduced pressure. The crude product was purified by flash chromatography on silica gel (EtOAc/hexane, 1 : 5 v/v) to give three separable regioisomers (28 mg, 70%, 1 : 3.3 : 1.3 ratio of sulfones **19a**, *trans*-**19b**, and *cis*-**19b**) as a colorless oil.

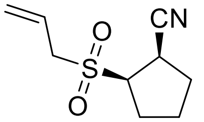

19a: ^{1}H NMR (500 MHz, $CDCl_3$): 5.95 (1 H, ddt, *J* = 17.4, 10.1, 7.4 Hz), 5.61–5.52 (2 H, m), 3.88–3.67 (3 H, m), 3.32 (1 H, dd, *J* = 15.1, 7.2 Hz), 2.38–2.10 (3 H, m), 2.05 (1 H, td, *J* = 14.3, 7.2 Hz), 1.91 (2 H, p, *J* = 7.0 Hz) ppm. – ^{13}C NMR (125 MHz, $CDCl_3$): 125.8, 124.3, 120.5, 62.7, 57.5, 32.5, 30.0, 26.6, 25.4 ppm. – IR: 3006, 1709, 1421, 1359, 1220, 1092, 902, 756, 665, 627, 529 cm^{-1}. – HRMS calcd for $C_9H_{14}NO_2S$: 200.074, found 200.0744 [M+H$^+$].

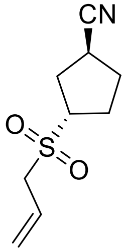

trans-**19b**: ^{1}H NMR (500 MHz, $CDCl_3$): 5.92 (1 H, ddt, *J* = 17.4, 10.1, 7.4 Hz), 5.63–5.35 (2 H, m), 3.72 (2 H, d, *J* = 8.0 Hz), 3.70–3.64 (1 H, m), 3.12–3.01 (1 H, m), 2.55 (1 H, ddd, *J* = 14.0, 7.9, 5.9 Hz), 2.34 – 2.19 (4 H, m), 2.07–1.96 (1 H, m) ppm. – ^{13}C NMR (125 MHz, $CDCl_3$): 125.0, 124.8, 121.2, 57.9, 56.9, 31.4, 31.0, 28.6, 25.8 ppm. – IR: 3019, 1710, 1362, 1215, 909, 751, 670, 632, 531 cm^{-1}. – HRMS calcd for $C_9H_{14}NO_2S$: 200.074, found 200.0741 [M+H$^+$].

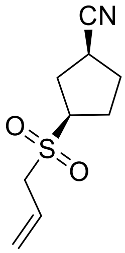

cis-**19b**: ^{1}H NMR (500 MHz, $CDCl_3$): 5.93 (1 H, ddt, *J* = 17.4, 10.1, 7.4 Hz), 5.48 (2 H, ddd, *J* = 18.1, 13.6, 0.8 Hz), 3.72 (2 H, d, *J* = 7.4 Hz), 3.58–3.46 (1 H, m), 2.82 (1 H, ddd, *J* = 15.1, 8.6, 4.9 Hz), 2.55–2.44 (1 H, m), 2.42–2.31 (2 H, m), 2.24–1.95 (3 H, m). – ^{13}C NMR (125 MHz, $CDCl_3$): 124.9, 124.8, 120.5, 58.3, 57.0, 31.2, 30.7, 28.5, 26.0 ppm. – IR: 3019, 1709, 1418, 1360, 1220, 1091, 912, 751, 734, 665, 648, 623, 529 cm^{-1}. – HRMS HMRS calcd for $C_9H_{14}NO_2S$: 200.074, found 200.0743 [M+H$^+$].

Go back to table of contents

Photoinduced C–H sulfination of 1-ethyl-4-fluorobenzene: 1-(1-(allylsulfonyl)ethyl)-4-fluorobenzene (20)

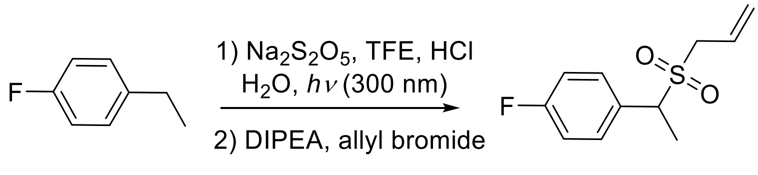

According to GP2, a stirred mixture of trifluoroethanol (3 mL) and $Na_2S_2O_5$ (285 mg, 1.5 mmol) was degassed with Ar for 5 min in a quartz test-tube. 1-Ethyl-4-fluorobenzene (29 mg, 0.2 mmol), water (1.5 mL, degassed prior to use) and 12M aqueous HCl (0.13 mL) were then added. The solution was stirred vigorously and irradiated at 25 °C for 16 h in a Rayonet RPR-100 photochemical reactor. The reaction mixture was transferred into a pressure tube, *N,N*-diisopropylethylamine (0.5 mL, 3 mmol) and allyl bromide (0.2 mL, 2 mmol) were added, and the reaction mixture was stirred at 60 °C for 4 h according to GP4. The reaction mixture was then extracted with EtOAc (3 × 15 mL). The organic phases were combined, dried over anhydrous Na_2SO_4 and concentrated under reduced pressure. The crude product was purified by flash chromatography on silica gel (EtOAc/hexane, 1 : 5 v/v) to give sulfone **20** (29 mg, 64%) as a colorless oil.

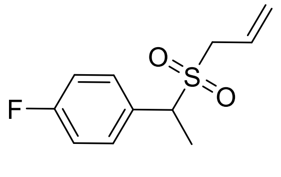

^{1}H NMR (500 MHz, $CDCl_3$): 7.42 (2 H, dd, *J* = 7.5, 5.3 Hz), 7.09 (1 H, t, *J* = 8.3 Hz), 5.97 – 5.70 (1 H, m), 5.46 (1 H, d, *J* = 10.0 Hz), 5.32 (1 H, d, *J* = 17.1 Hz), 4.24 (1 H, dd, *J* = 13.7, 6.7 Hz), 3.50 (2 H, d, *J* = 4.7 Hz), 1.75 (3 H, d, *J* = 7.0 Hz) ppm. – ^{13}C NMR (125 MHz, $CDCl_3$): 164.2, 162.2, 131.1, 131.1, 130.1, 130.0, 124.8, 124.7, 116.2, 116.1, 61.2, 55.1, 14.3 ppm. – ^{19}F NMR (471 MHz, $CDCl_3$): –112.0 ppm. – IR: 3003, 1709, 1421, 1359, 1220, 1092, 901, 684, 629, 529 cm^{-1}. – HRMS calcd for $C_{11}H_{14}FO_2S$: 229.0693, found 229.0696 [M+H$^+$].

Go back to table of contents

Photoinduced C–H sulfination of 1-chlorobutane: sultine 21a,b[9]

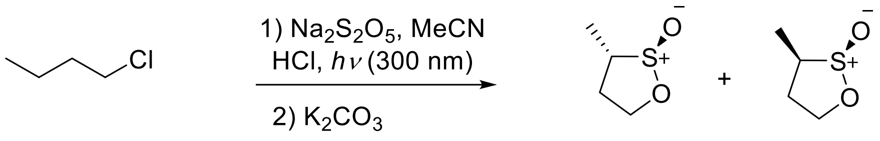

According to GP2, a stirred mixture of MeCN (5 mL) and $Na_2S_2O_5$ (570 mg, 3 mmol) was degassed with Ar for 5 min in a quartz test-tube. 1-Chlorobutane (28 mg, 0.3 mmol) and 12M aqueous HCl (0.25 mL) were then added. The solution was stirred vigorously and irradiated at 25 °C for 16 h in a Rayonet RPR-100 photochemical reactor. The reaction mixture was transferred into a pressure tube, potassium carbonate (828 mg, 5 mmol) was added, and the reaction mixture was stirred at 80 °C for 8 h. The reaction mixture was then filtered. The organic phases was dried over anhydrous Na_2SO_4 and concentrated under reduced pressure. The crude product was purified by flash chromatography on silica gel (EtOAc/hexane, 1 : 5 v/v) to give two separable isomers (18 mg, 52%, 1 : 3.2 ratio of γ-sultines **21b** and **21a** as a colorless oil.

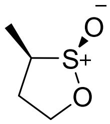

21b: ^{1}H NMR (500 MHz, $CDCl_3$): 4.85–4.74 (1 H, m), 4.51 (2 H, dd, *J* = 15.7, 8.3 Hz), 3.40–3.29 (1 H, m), 2.75 (1 H, dq, *J* = 12.9, 8.3 Hz), 1.95–1.82 (1 H, m), 1.22 (3 H, d, *J* = 7.4 Hz) ppm. – ^{13}C NMR (125 MHz, $CDCl_3$): 74.9, 66.8, 30.3, 13.3 ppm. – IR: 3004, 2359, 1709, 1421, 1358, 1220, 1092, 901, 685, 628, 528 cm^{-1}.

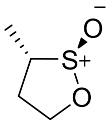

21a: ^{1}H NMR (500 MHz, $CDCl_3$): 4.77 (1 H, td, *J* = 8.6, 2.5 Hz), 4.32 (2 H, ddd, *J* = 10.0, 8.6, 7.0 Hz), 3.14–2.99 (1 H, m), 2.33–2.11 (2 H, m), 1.40 (3 H, d, *J* = 6.7 Hz) ppm. – ^{13}C NMR (125 MHz, $CDCl_3$): 75.4, 62.8, 29.9, 11.3 ppm. – IR: , 2931, 2858, 1455, 1318, 1261, 1173, 1120, 1072, 1021, 952, 927, 865, 766, 715, 664, 646, 631, 614, 593, 584, 561, 555, 536, 523 cm^{-1}.

Go back to table of contents

Photoinduced C–H sulfination of *N*-butyl-2,2,2-trifluoroacetamide: sulfone 22a-c

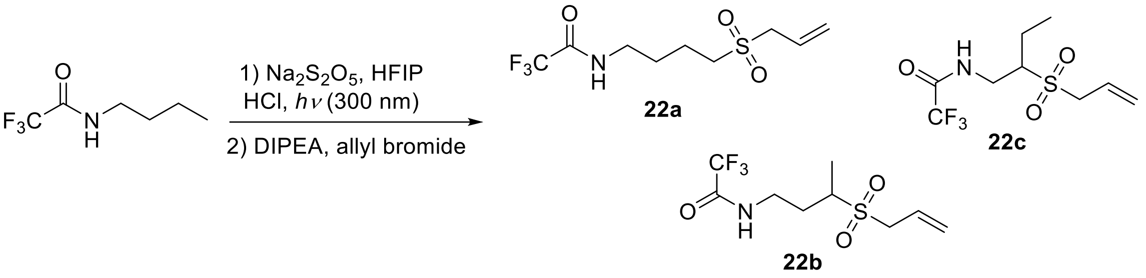

According to GP2, a stirred mixture of hexafluoroisopropanol (5 mL) and $Na_2S_2O_5$ (570 mg, 3 mmol) was degassed with Ar for 5 min in a quartz test-tube. *N*-butyl-2,2,2-trifluoroacetamide (51 mg, 0.3 mmol) and 12M aqueous HCl (0.25 mL) were then added. The solution was stirred vigorously and irradiated at 25 °C for 16 h in a Rayonet RPR-100 photochemical reactor. The reaction mixture was transferred into a pressure tube, *N,N*-diisopropylethylamine (0.5 mL, 3 mmol) and allyl bromide (0.26 mL, 3 mmol) were added, and the reaction mixture was stirred at 60 °C for 4 h according to GP4. The reaction mixture was then extracted with EtOAc (3 × 15 mL). The organic phases were combined, dried over anhydrous Na_2SO_4 and concentrated under reduced pressure. The crude product was purified by flash chromatography on silica gel (EtOAc/hexane, 1 : 5 v/v) to give three separable isomers (51 mg, 62%, 1 : 2.9 : 1.2 ratio of sulfones **22a**, **22b**, and **22c**) as a colorless oil.

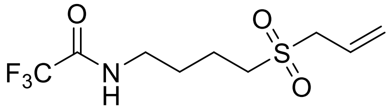

22a: ^1H NMR (500 MHz, $CDCl_3$): 6.66 (1 H, s), 6.01 – 5.83 (1 H, m), 5.62 – 5.33 (2 H, m), 3.72 (2 H, d, *J* = 7.4 Hz), 3.42 (2 H, q, *J* = 6.5 Hz), 3.01 (2 H, t, *J* = 7.5 Hz), 1.94 – 1.85 (2 H, m), 1.84 – 1.73 (2 H, m) ppm. – ^{13}C NMR (125 MHz, $CDCl_3$): 157.8, 157.5, 125.0, 119.7, 117.1, 114.8, 112.6, 100.1, 58.3, 50.3, 39.2, 27.8, 19.0 ppm. – ^{19}F NMR (471 MHz, $CDCl_3$): –75.9 ppm. – IR: 3003, 1709, 1422, 1358, 1220, 1092, 900, 685, 629, 528 cm^{-1}. – HRMS calcd for $C_9H_{15}F_3NO_3S$: 274.0719, found 274.0723 [$M+H^+$].

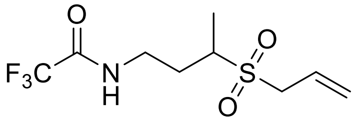

22b: ^1H NMR (500 MHz, $CDCl_3$): 6.93 (1 H, s), 6.04 – 5.79 (1 H, m), 5.50 (2 H, dd, *J* = 21.6, 13.6 Hz), 3.75 (2 H, qd, *J* = 14.2, 7.4

Go back to table of contents

Hz), 3.66 – 3.45 (2 H, m), 3.14 (1 H, dd, *J* = 13.4, 6.7 Hz), 2.24 (1 H, dt, *J* = 21.1, 6.6 Hz), 1.96 (1 H, td, *J* = 13.2, 6.6 Hz), 1.44 (3 H, d, *J* = 6.9 Hz) ppm. – ^{13}C NMR (125 MHz, $CDCl_3$): 158.2, 157.9, 157.6, 157.3, 125.1, 124.5, 119.3, 117.0, 114.8, 112.5, 55.4, 53.7, 37.1, 28.4, 14.4. ppm. – ^{19}F NMR (471 MHz, $CDCl_3$): –75.9 ppm. – IR: 3004, 1709, 1421, 1358, 1220, 1092, 901, 683, 627, 528 cm^{-1}. – HRMS calcd for $C_9H_{15}F_3NO_3S$: 274.0719, found 274.0722 [M+H$^+$].

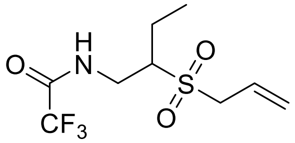

22c: ^{1}H NMR (500 MHz, $CDCl_3$): 7.32 (1 H, s), 6.07–5.79 (1 H, m), 5.52 (2 H, dd, *J* = 33.9, 13.8 Hz), 3.91 (ddd, *J* = 15.0, 6.0, 2.2 Hz, 1H), 3.85–3.68 (3 H, m), 3.13–2.99 (1 H, m), 2.04–1.89 (1 H, m), 1.79–1.62 (1 H, m), 1.12 (3 H, t, *J* = 7.5 Hz) ppm. – ^{13}C NMR (125 MHz, $CDCl_3$): 157.7, 157.4, 125.7, 124.2, 119.2, 116.9, 114.6, 112.4, 61.0, 56.8, 36.2, 19.7, 11.1 ppm. – ^{19}F NMR (471 MHz, $CDCl_3$): –76.1 ppm. – IR: 3586, 3004, 2364, 1708, 1421, 1358, 1220, 1092, 920, 734, 667, 626, 529 cm^{-1}. – HRMS calcd for $C_9H_{15}F_3NO_3S$: 274.0719, found 274.0718 [M+H$^+$].

Photoinduced C–H sulfination of methyl (2,2,2-trifluoroacetyl)-D-valinate: methyl *C*^{4}-(allylsulfonyl)(2,2,2-trifluoroacetyl)-L-valinate (23)

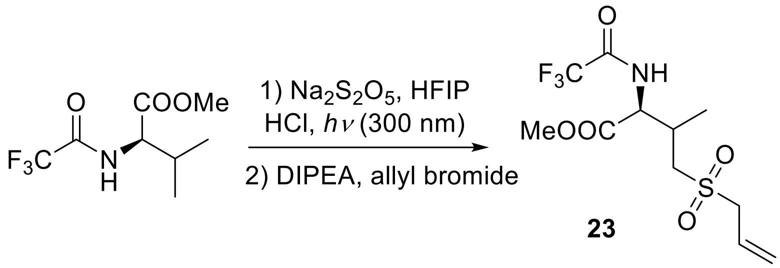

According to GP2, a stirred mixture of hexafluoroisopropanol (5 mL) and $Na_2S_2O_5$ (570 mg, 3 mmol) was degassed with Ar for 5 min in a quartz test-tube. Methyl (2,2,2-trifluoroacetyl)-D-valinate (45 mg, 0.2 mmol) and 12M aqueous HCl (0.25 mL) were then added. The solution was stirred vigorously and irradiated at 25 °C for 24 h in a Rayonet RPR-100 photochemical reactor. The reaction mixture was transferred into a pressure tube, *N,N*-diisopropylethylamine (0.5 mL, 3 mmol) and allyl bromide (0.2 mL, 2 mmol) were added, and the reaction mixture was stirred at 60 °C for 4 h according to GP4. The

Go back to table of contents

reaction mixture was then extracted with EtOAc (3 × 15 mL). The organic phases were combined, dried over anhydrous Na_2SO_4 and concentrated under reduced pressure. The crude product was purified by flash chromatography on silica gel (EtOAc/hexane, 1 : 5 v/v) to give the product (34 mg, 52%) as a colorless oil.

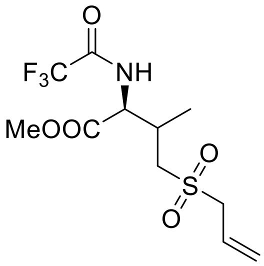

^1H NMR (500 MHz, $CDCl_3$): 7.34 (1 H, s), 6.00–5.83 (1 H, m), 5.60–5.41 (2 H, m), 4.75 (1 H, ddd, J = 12.3, 7.7, 3.9 Hz), 3.86–3.68 (5 H, m), 3.26 (1 H, ddd, J = 76.7, 14.2, 5.3 Hz), 3.04–2.66 (2 H, m), 1.18 (3 H, dd, J = 6.9, 4.2 Hz) ppm. – ^{13}C NMR (125 MHz, $CDCl_3$): 169.8, 169.6, 157.9, 157.6, 157.6, 157.2, 125.5, 125.4, 125.1, 124.9, 124.8, 123.7, 118.6, 116.9, 116.8, 114.6, 114.5, 112.3, 59.3, 58.4, 56.3, 56.29, 53.7, 53.5, 53.4, 31.0, 30.9, 17.0, 16.2 ppm.– ^{19}F NMR (471 MHz, $CDCl_3$): –75.7 ppm. – IR: 2957, 1737, 1465, 1373, 1242, 1047, 914, 758, 735, 632, 609, 534 cm^{-1}. – HRMS calcd for $C_{11}H_{17}F_3NO_5S$: 332.0774, found 332.0781 [M+H$^+$].

Photoinduced C–H sulfination of methyl 2-(4-isobutylphenyl)propanoate: sulfone 24a,b

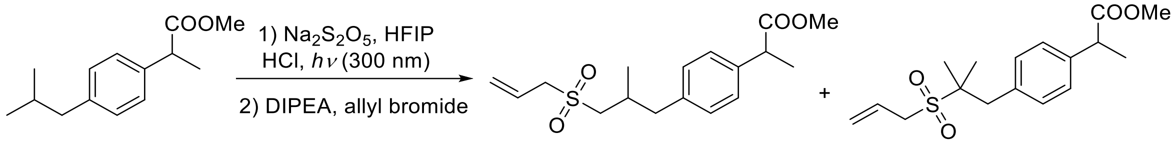

According to GP1, a stirred mixture of hexafluoroisopropanol (4.5 mL) and $Na_2S_2O_5$ (570 mg, 3 mmol) was degassed with Ar for 5 min in a quartz test-tube. Methyl 2-(4-isobutylphenyl)propanoate (66 mg, 0.3 mmol) and 12M aqueous HCl (0.25 mL) were then added. The solution was stirred vigorously and irradiated at 25 °C for 16 h in a Rayonet RPR-100 photochemical reactor. The reaction mixture was transferred into a pressure tube, *N,N*-diisopropylethylamine (0.5 mL, 3 mmol) and allyl bromide (0.26 mL, 3 mmol) were added, and the reaction mixture was stirred at 60 °C for 4 h according to GP3. The reaction mixture was then extracted with EtOAc (3 × 15 mL). The organic phases were combined, dried over anhydrous Na_2SO_4 and concentrated under reduced pressure. The

Go back to table of contents

crude product was purified by flash chromatography on silica gel (EtOAc/hexane, 1:3 v/v) to give inseparable product (63 mg, 65%, 1:1.2 ratio of isomers **24a** and **24b**) as a colorless oil.

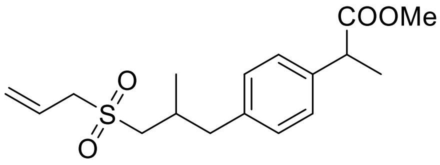

24a: ^{1}H NMR (500 MHz, $CDCl_3$): 7.26–7.22 (2 H, m), 7.17–7.09 (2 H, m), 5.81 (1 H, ddd, *J* = 24.6, 9.8, 7.5 Hz), 5.31 (2 H, dd, *J* = 59.3, 13.6 Hz), 3.74–3.69 (1 H, m), 3.66 (3 H, s), 3.61 (2 H, d, *J* = 7.4 Hz), 3.00 (1 H, dd, *J* = 14.0, 4.5 Hz), 2.77–2.63 (3 H, m), 2.46 (1 H, dt, *J* = 19.0, 6.8 Hz), 1.49 (3 H, d, *J* = 7.1 Hz), 1.17 (3 H, d, *J* = 6.7 Hz) ppm.

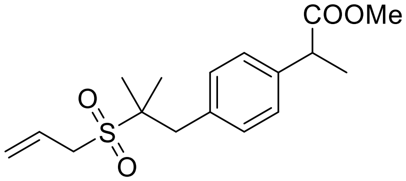

24b: ^{1}H NMR (500 MHz, $CDCl_3$): 7.27–7.21 (2 H, m), 7.18–7.08 (2 H, m), 5.99 (1 H, ddt, *J* = 17.3, 10.1, 7.2 Hz), 5.47 (2 H, dd, *J* = 29.3, 13.6 Hz), 3.71 (2 H, d, *J* = 6.5 Hz), 3.77–3.65 (1 H, m), 3.67 (3 H, s), 3.07 (2 H, s), 1.50 (3 H, d, *J* = 7.1 Hz), 1.34 (6 H, s) ppm.
^{13}C NMR (125 MHz, $CDCl_3$, mixture of regioisomers): 175.1, 175.0, 139.7, 139.0, 137.9, 133.8, 131.4, 129.7, 127.8, 127.6, 125.3, 124.6, 124.5, 64.0, 58.9, 56.2, 52.5, 52.2, 52.18, 45.2, 42.6, 40.2, 30.4, 20.7, 20.3, 18.7 ppm. – IR: 2922, 1732, 1512, 1455, 1290, 1208, 1162, 1106, 936, 645, 619 cm^{-1}. – HRMS calcd for $C_{17}H_{24}O_4S$: 325.1468, found 325.1467 [M+H^{+}].

Photoinduced C–H sulfination of 1,4-dioxane: 2-(allylsulfonyl)-1,4-dioxane (25)

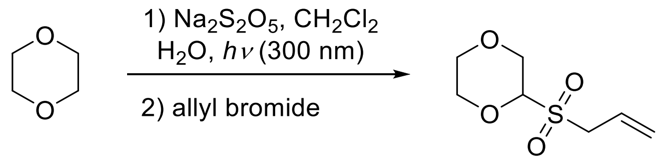

25

According to GP1, a stirred mixture of dichloromethane (3 mL) and $Na_2S_2O_5$ (76 mg, 0.4 mmol) was degassed with Ar for 5 min in a quartz test-tube. 1,4-Dioxane (18 mg, 0.2 mmol) and water (0.75 mL, degassed prior to use) were then added. The solution was stirred vigorously and irradiated at 25 °C for 16 h in a Rayonet RPR-100 photochemical

reactor. The reaction mixture was transferred into a pressure tube, allyl bromide (0.2 mL, 2 mmol) were added, and the reaction mixture was stirred at 60 °C for 4 h according to GP3. The reaction mixture was then extracted with EtOAc (3 × 15 mL). The organic phases were combined, dried over anhydrous Na_2SO_4 and concentrated under reduced pressure. The crude product was purified by flash chromatography on neutral aluminum (EtOAc/hexane, 1:3 v/v) to give sulfone **25** (33 mg, 85%) as a colorless oil.

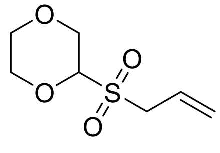

^{1}H NMR (500 MHz, $CDCl_3$): 5.92–5.79 (1 H, m), 5.49–5.38 (2 H, m), 4.56 (1 H, dd, J = 6.5, 3.7 Hz), 4.23–4.16 (1 H, m), 4.03 (2 H, ddd, J = 16.0, 12.3, 5.1 Hz), 3.90 (1 H, dd, J = 14.1, 8.5 Hz), 3.75–3.62 (4 H, m) ppm. – ^{13}C NMR (125 MHz, $CDCl_3$): 125.2, 124.1, 83.4, 66.0, 65.5, 62.4, 54.7 ppm. – IR: 2957, 2925, 2873, 1743, 1458, 1373, 1237, 1113, 1047, 916, 848, 736, 632, 608, 537 cm^{-1}. – HRMS calcd for $C_7H_{12}NaO_4S$: 215.0349, found 215.0354 [M+Na$^+$].

Photoinduced C–H sulfination of tetrahydrofuran: 2-(allylsulfonyl)tetrahydrofuran (26)

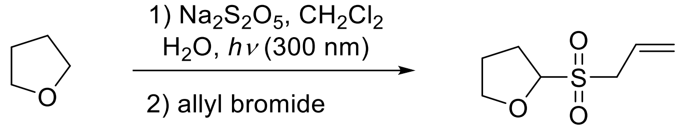

26

According to GP1, a stirred mixture of dichloromethane (3 mL) and $Na_2S_2O_5$ (76 mg, 0.4 mmol) was degassed with Ar for 5 min in a quartz test-tube. Tetrahydrofuran (14 mg, 0.2 mmol) and water (0.75 mL, degassed prior to use) were then added. The solution was stirred vigorously and irradiated at 25 °C for 16 h in a Rayonet RPR-100 photochemical reactor. The reaction mixture was transferred into a pressure tube, allyl bromide (0.2 mL, 2 mmol) was added, and the reaction mixture was stirred at 60 °C for 4 h according to GPXX. The reaction mixture was then extracted with EtOAc (3 × 15 mL). The organic phases were combined, dried over anhydrous Na_2SO_4 and concentrated under reduced

pressure. The crude product was purified by flash chromatography on neutral aluminum (EtOAc/hexane, 1:3 v/v) to give sulfone **26** (21 mg, 60%) as a colorless oil.

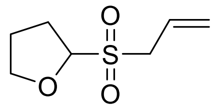

^{1}H NMR (500 MHz, $CDCl_3$): 5.99 – 5.80 (1 H, m), 5.53 – 5.40 (2 H, m), 4.95 (1 H, dd, *J* = 7.9, 3.9 Hz), 4.15 (1 H, dd, *J* = 15.0, 7.2 Hz), 4.05 (1 H, dt, *J* = 13.2, 6.7 Hz), 3.93 (1 H, dd, *J* = 14.0, 8.6 Hz), 3.69 (1 H, dd, *J* = 13.9, 6.2 Hz), 2.67 – 2.47 (1 H, m), 2.31 – 2.13 (2 H, m), 2.02 – 1.88 (1 H, m) ppm. – ^{13}C NMR (125 MHz, $CDCl_3$): 124.9, 124.6, 89.9, 71.2, 54.2, 25.2, 24.6 ppm. – IR: 1739, 1716, 1364, 1218, 758, 749, 710, 697, 630, 589, 555 cm^{-1}. – HRMS calcd for $C_7H_{12}NaO_3S$: 199.0399, found 199.0401 [M+Na^+].

Photoinduced C–H sulfination of dimethoxymethane: 3-(((methoxymethoxy)methyl)sulfonyl)prop-1-ene (27)

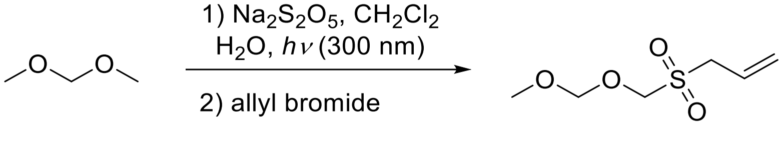

27

According to GP1, a stirred mixture of dichloromethane (3 mL) and $Na_2S_2O_5$ (152 mg, 0.8 mmol) was degassed with Ar for 5 min in a quartz test-tube. Dimethoxymethane (16 mg, 0.2 mmol) and water (0.75 mL, degassed prior to use) were then added. The solution was stirred vigorously and irradiated at 25 °C for 16 h in a Rayonet RPR-100 photochemical reactor. The reaction mixture was transferred into a pressure tube, allyl bromide (0.2 mL, 2 mmol) were added, and the reaction mixture was stirred at 60 °C for 4 h according to GPXX. The reaction mixture was then extracted with EtOAc (3 × 15 mL). The organic phases were combined, dried over anhydrous Na_2SO_4 and concentrated under reduced pressure. The crude product was purified by flash chromatography on neutral aluminum (EtOAc/hexane, 1:2 v/v) to give sulfone **27** (28 mg, 78%) as a colorless oil.

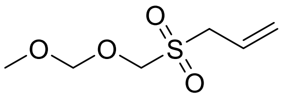

^{1}H NMR (500 MHz, $CDCl_3$): 5.63 (1 H, ddt, *J* = 16.9, 10.3, 7.4 Hz), 4.99 (2 H, dd, *J* = 12.1, 5.8 Hz), 4.52 (2 H, s), 4.12 (2 H, s), 3.31 (2 H,

Go back to table of contents

d, J = 7.4 Hz), 2.98 (3 H, s) ppm. – ^{13}C NMR (125 MHz, $CDCl_3$): 125.4, 123.8, 97.0, 77.2, 55.8, 54.7 ppm. – IR: 2970, 2279, 1712, 1422, 1361, 1219, 1092, 1048, 814, 639, 608, 521 cm^{-1}. – HRMS calcd for $C_6H_{12}NaO_4S$: 203.0349, found 203.0348 [$M+Na^+$].

Photoinduced C–H sulfination of methyl *tert*-butyl ether: 3-((*tert*-butoxymethyl)sulfonyl)prop-1-ene (28)

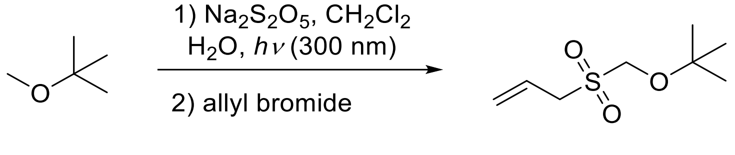

28

According to GP1, a stirred mixture of dichloromethane (3 mL) and $Na_2S_2O_5$ (38 mg, 0.2 mmol) was degassed with Ar for 5 min in a quartz test-tube. Methyl *tert*-butyl ether (17 mg, 0.2 mmol) and water (0.75 mL, degassed prior to use) were then added. The solution was stirred vigorously and irradiated at 25 °C for 16 h in a Rayonet RPR-100 photochemical reactor. The reaction mixture was transferred into a pressure tube, allyl bromide (0.2 mL, 2 mmol) were added, and the reaction mixture was stirred at 60 °C for 4 h according to GP3. The reaction mixture was then extracted with EtOAc (3 × 15 mL). The organic phases were combined, dried over anhydrous Na_2SO_4 and concentrated under reduced pressure. The crude product was purified by flash chromatography on neutral aluminum (EtOAc/hexane, 1:2 v/v) to give sulfone **28** (19 mg, 50%) as a colorless oil.

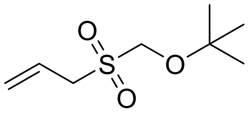

^1H NMR (500 MHz, $CDCl_3$): 5.71 (1 H, ddt, J = 17.4, 10.0, 7.4 Hz), 5.03 (2 H, dd, J = 21.6, 5.6 Hz), 4.03 (2 H, s), 3.38 (2 H, d, J = 7.4 Hz), 0.90 (9 H, s) ppm. – ^{13}C NMR (125 MHz, $CDCl_3$): 125.7, 123.6, 77.0, 75.6, 54.3, 27.3 ppm. – IR: 3017, 2280, 1712, 1419, 1361, 1330, 1219, 1093, 813, 753, 665, 632, 601, 529 cm^{-1}. – HRMS calcd for $C_8H_{16}NaO_3S$: 215.0712, found 215.0713 [$M+Na^+$].

Go back to table of contents

Photoinduced C–H sulfination of 15-crown-5: 2-(allylsulfonyl)-1,4,7,10,13-pentaoxacyclopentadecane (29)

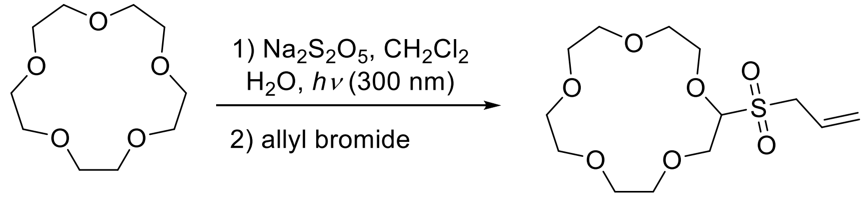

29

According to GP1, a stirred mixture of dichloromethane (3 mL) and $Na_2S_2O_5$ (114 mg, 0.6 mmol) was degassed with Ar for 5 min in a quartz test-tube. 15-Crown-5 (44 mg, 0.2 mmol) and water (0.75 mL, degassed prior to use) were then added. The solution was stirred vigorously and irradiated at 25 °C for 16 h in a Rayonet RPR-100 photochemical reactor. The reaction mixture was transferred into a pressure tube, allyl bromide (0.2 mL, 2 mmol) were added, and the reaction mixture was stirred at 60 °C for 4 h according to GP3. The reaction mixture was then extracted with EtOAc (3 × 15 mL). The organic phases were combined, dried over anhydrous Na_2SO_4 and concentrated under reduced pressure. The crude product was purified by flash chromatography on neutral aluminum (EtOAc/hexane, 1:1 v/v) to give sulfone **29** (41 mg, 64%) as a colorless oil.

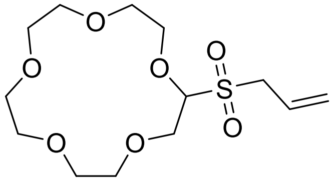

^1H NMR (500 MHz, C_6D_6): 5.88 (1 H, ddt, *J* = 17.3, 10.1, 7.4 Hz), 5.22–5.03 (2 H, m), 4.54 (1 H, t, *J* = 4.8 Hz), 4.08–3.99 (2 H, m), 3.99–3.92 (1 H, m), 3.90–3.79 (1 H, m), 3.73 (1 H, dd, *J* = 13.8, 7.1 Hz), 3.61 (1 H, dd, *J* = 13.8, 7.7 Hz), 3.45–3.16 (14 H, m) ppm. – ^{13}C NMR (125 MHz, C_6D_6): 125.1, 123.8, 94.6, 73.1, 71.0, 70.9, 70.88, 70.8, 70.7, 70.64, 69.62, 55.8 ppm. – IR: 2983, 1737, 1446, 1372, 1234, 1096, 1044, 938, 847, 636, 607 cm^{-1}. – HRMS calcd for $C_{13}H_{24}NaO_7S$: 347.1135, found 347.1134 $[M+Na^+]$.

Go back to table of contents

Photoinduced C–H sulfination of 18-crown-6: 2-(allylsulfonyl)-1,4,7,10,13,16-hexaoxacyclooctadecane (30)

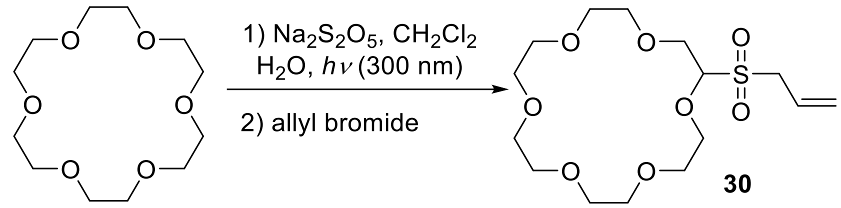

According to GP1, a stirred mixture of dichloromethane (3 mL) and $Na_2S_2O_5$ (114 mg, 0.6 mmol) was degassed with Ar for 5 min in a quartz test-tube. 18-Crown-6 (53 mg, 0.2 mmol) and water (0.75 mL, degassed prior to use) were then added. The solution was stirred vigorously and irradiated at 25 °C for 16 h in a Rayonet RPR-100 photochemical reactor. The reaction mixture was transferred into a pressure tube, allyl bromide (0.2 mL, 2 mmol) were added, and the reaction mixture was stirred at 60 °C for 4 h according to GP3. The reaction mixture was then extracted with EtOAc (3 × 15 mL). The organic phases were combined, dried over anhydrous Na_2SO_4 and concentrated under reduced pressure. The crude product was purified by flash chromatography on neutral aluminum (EtOAc/hexane, 1:1 v/v) to give sulfone **42** (43 mg, 58%) as a colorless oil.

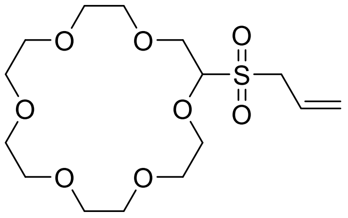

^{1}H NMR (500 MHz, C_6D_6): 5.97 (1, ddt, *J* = 17.4, 10.1, 7.4 Hz), 5.29–5.15 (2 H, m), 4.52 (1 H, t, *J* = 3.9 Hz), 4.15–4.01 (2 H, m), 4.01–3.91 (2 H, m), 3.90–3.73 (2 H, m), 3.49–3.16 (18 H, m) ppm. – ^{13}C NMR (125 MHz, C_6D_6): 125.1, 124.0, 94.8, 72.4, 71.3, 71.0, 70.97, 70.93, 70.85, 70.81, 70.76, 69.79, 56.0 ppm. – IR: 3335, 2944, 2831, 1707, 1449, 1230, 1111, 1021, 816, 736, 610, 536 cm^{-1}. – HRMS calcd for $C_{15}H_{28}NaO_8S$: 391.1397, found 391.1399 [M+Na$^+$].

Go back to table of contents

Photoinduced C–H sulfination of (3*R*,3a*R*,6*R*,6a*R*)-3,6-dimethoxyhexahydrofuro[3,2-b]furan: sulfone 31a-c

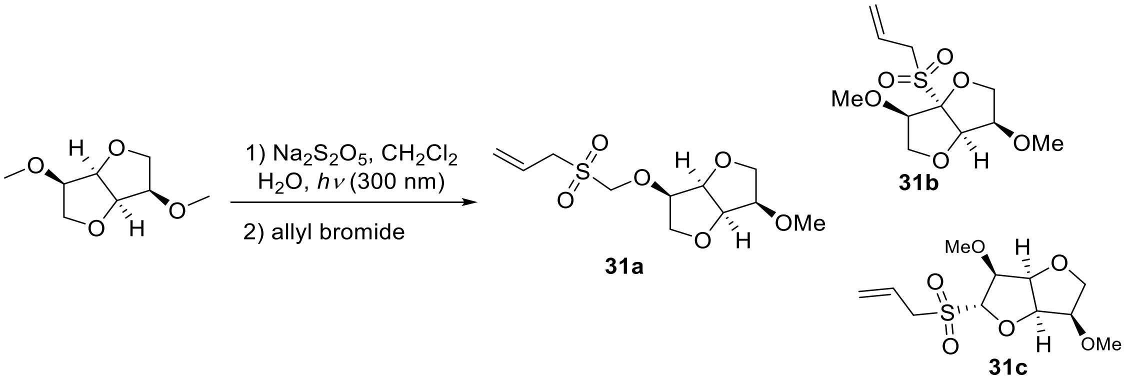

According to GP1, a stirred mixture of dichloromethane (3 mL) and $Na_2S_2O_5$ (190 mg, 1 mmol) was degassed with Ar for 5 min in a quartz test-tube. (3*R*,3a*R*,6*R*,6a*R*)-3,6-Dimethoxyhexahydrofuro[3,2-b]furan (52 mg, 0.3 mmol) and water (0.75 mL, degassed prior to use) were then added. The solution was stirred vigorously and irradiated at 25 °C for 16 h in a Rayonet RPR-100 photochemical reactor. The reaction mixture was transferred into a pressure tube, allyl bromide (0.26 mL, 3 mmol) were added, and the reaction mixture was stirred at 60 °C for 4 h according to GP3. The reaction mixture was then extracted with EtOAc (3 × 15 mL). The organic phases were combined, dried over anhydrous Na_2SO_4 and concentrated under reduced pressure. The crude product was purified by flash chromatography on silica gel (EtOAc/hexane, 1:3 v/v) to give three separable regioisomers (51 mg, 61%, 4:3.2:1 ratio of sulfones **31a**, **31b**, and **31c**) as a colorless oil.

Go back to table of contents

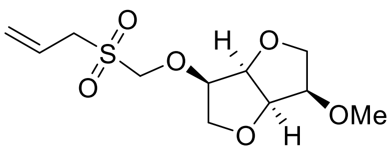

31a: $[\alpha]_D^{21}$ = –5.0 (*c* 0.2, $CDCl_3$). – ^1H NMR (500 MHz, C_6D_6): 5.62 (1 H, ddt, *J* = 17.4, 10.1, 7.4 Hz), 5.06–4.91 (2 H, m), 4.17–4.12 (4 H, m), 3.95 (1 H, t, *J* = 4.9 Hz), 3.67 (4 H, dtd, *J* = 11.6, 8.5, 6.2 Hz), 3.47–3.24 (3 H, m), 3.15 (3 H, s) ppm. – ^{13}C NMR (125 MHz, C_6D_6): 125.2, 124.1, 82.2, 81.6, 81.4, 80.8, 80.4, 71.5, 71.4, 57.7, 54.3 ppm. – IR: 2930, 1295, 1241, 1221, 1143, 1120, 1091, 1040, 940, 878, 802, 744, 630, 597, 534 cm^{-1}. – HRMS calcd for $C_{11}H_{19}O_6S$: 279.0897, found 279.0897 [M+H$^+$].

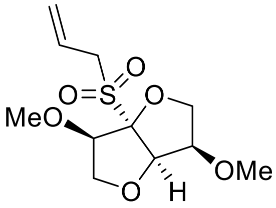

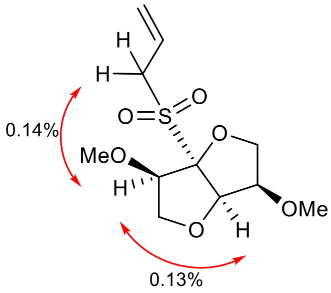

31b: $[\alpha]_D^{21}$ = +93.9 (*c* 0.14, $CDCl_3$). – ^1H NMR (500 MHz, C_6D_6): 5.86 (1 H, ddt, *J* = 17.4, 10.1, 7.4 Hz), 5.15–5.05 (3 H, m), 4.29 (1 H, dd, *J* = 7.3, 6.5 Hz), 4.21 (1 H, dd, *J* = 9.1, 6.3 Hz), 3.97 (1 H, dd, *J* = 9.1, 5.6 Hz), 3.89 (1 H, dd, *J* = 8.5, 6.2 Hz), 3.83–3.76 (2 H, m), 3.70 (1 H, q, *J* = 5.8 Hz), 3.59 (1 H, dd, *J* = 13.8, 7.5 Hz), 3.06 (3 H, s), 2.86 (3 H, s) ppm. – ^{13}C NMR (125 MHz, C_6D_6): 124.5, 123.8, 105.9, 82.1, 81.4, 81.2, 75.9, 72.3, 58.3, 58.2, 54.2 ppm.– IR: 2925, 1459, 1327, 1204, 1085, 631, 537 cm^{-1}. – HRMS calcd for $C_{11}H_{19}O_6S$: 279.0897, found 279.0898 [M+H$^+$].

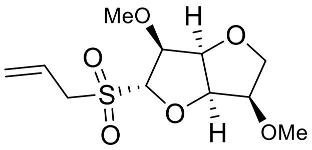

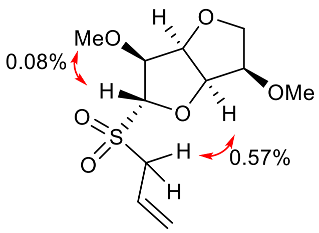

31c: $[\alpha]_D^{21}$ = +100 (*c* 0.03, $CDCl_3$). – ^1H NMR (500 MHz, C_6D_6): 5.67–5.53 (1 H, m), 5.05–4.88 (3 H, m), 4.63 (1 H, t, *J* = 4.3 Hz), 4.49–4.40 (1 H, m), 4.37–4.27 (1 H, m), 3.78 (2 H, dt, *J* = 17.5, 8.2 Hz), 3.44 (1 H, dd, *J* = 14.0, 8.5 Hz), 3.34–3.19 (5 H, m), 3.02 (3 H, s) ppm. – ^{13}C NMR (125 MHz, C_6D_6): 124.6, 124.4, 95.3, 83.4, 82.0, 81.3, 80.8, 69.8, 58.8, 57.7, 54.5 ppm.– IR: 2923, 1712, 1463, 1315, 1223, 1145, 1092, 995, 733, 631, 535 cm^{-1}. – HRMS calcd for $C_{11}H_{19}O_6S$: 279.0897, found 279.0897 [M+H$^+$].

Go back to table of contents

Photoinduced C–H sulfination of methylpentan-2-one: sulfone 33-34

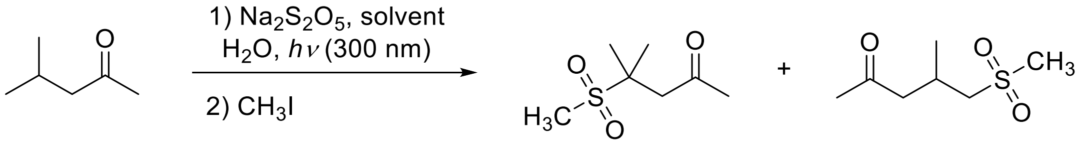

33 **34**

In dichloromethane: According to GP1, a stirred mixture of dichloromethane (4 mL) and $Na_2S_2O_5$ (114 mg, 0.6 mmol) was degassed with Ar for 5 min in a quartz test-tube. 4-Methylpentan-2-one (30 mg, 0.2 mmol) and water (0.5 mL, degassed prior to use) were then added. The solution was stirred vigorously and irradiated at 25 °C for 16 h in a Rayonet RPR-100 photochemical reactor. The reaction mixture was transferred into a pressure tube, iodomethane (0.1 mL, 2 mmol) was added, and the reaction mixture was stirred at 60 °C for 4 h according to GP3. The reaction mixture was then extracted with EtOAc (3 × 15 mL). The organic phases were combined, dried over anhydrous Na_2SO_4 and concentrated under reduced pressure. The crude product was purified by flash chromatography on silica gel (EtOAc/hexane, 1 : 5 v/v) to give two separable regioisomers (28 mg, 79%, 1.4 : 1 ratio of sulfones **33** and **34**) as a colorless oil.

In HFIP: According to GP1, a stirred mixture of hexafluoroisopropanol (3 mL) and $Na_2S_2O_5$ (48 mg, 0.25 mmol) was degassed with Ar for 5 min in a quartz test-tube. 4-Methylpentan-2-one (30 mg, 0.2 mmol) and water (0.75 mL, degassed prior to use) were then added. The solution was stirred vigorously and irradiated at 25 °C for 16 h in a Rayonet RPR-100 photochemical reactor. The reaction mixture was transferred into a pressure tube, iodomethane (0.1 mL, 2 mmol) was added, and the reaction mixture was stirred at 60 °C for 4 h according to GP3. The reaction mixture was then extracted with EtOAc (3 × 15 mL). The organic phases were combined, dried over anhydrous Na_2SO_4 and concentrated under reduced pressure. The crude product was purified by flash chromatography on silica gel (EtOAc/hexane, 1:3 v/v) to give two separable regioisomers (27 mg, 75%, 1 : 10 ratio of sulfones **33** and **34**) as a colorless oil.

Go back to table of contents

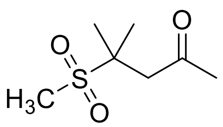

33: ^{1}H NMR (500 MHz, $CDCl_3$): 2.92 (2 H, s), 2.83 (3 H, s), 2.21 (3 H, s), 1.55 (6 H, s) ppm. – ^{13}C NMR (125 MHz, $CDCl_3$): 204.8, 61.4, 45.3, 34.5, 32.2, 20.5 ppm. – IR: 3004, 1708, 1420, 1359, 1220, 1092, 902, 625, 528 cm^{-1}. – HRMS calcd for $C_7H_{15}O_3S$: 179.0736, found 179.074 [M+H$^+$].

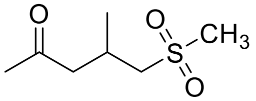

34: ^{1}H NMR (500 MHz, $CDCl_3$): 3.09 (1 H, dd, *J* = 14.1, 6.0 Hz), 2.93 (3 H, s), 2.89 (1 H, dd, *J* = 14.2, 6.7 Hz), 2.75 (1 H, dd, *J* = 17.7, 6.6 Hz), 2.63 (1 H, dq, *J* = 12.8, 6.4 Hz), 2.53 (1 H, dd, *J* = 17.7, 5.7 Hz), 2.13 (3 H, s), 1.16 (3 H, d, *J* = 6.8 Hz) ppm. – ^{13}C NMR (125 MHz, $CDCl_3$): 207.2, 59.6, 49.0, 41.5, 30.5, 24.8, 20.5 ppm. – IR: 3999, 2928, 1706, 1415, 1366, 1291, 1223, 1190, 1128, 967, 851, 637, 603, 525 cm^{-1}. – HRMS calcd for $C_7H_{15}O_3S$: 179.0736, found 179.0738 [M+H$^+$].

Photoinduced C–H sulfination of heptan-4-one: sulfones S1-S2

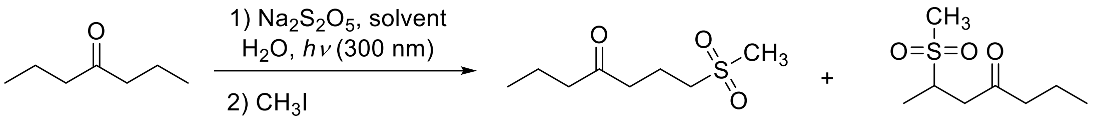

In dichloromethane: According to GP1, a stirred mixture of dichloromethane (3 mL) and $Na_2S_2O_5$ (114 mg, 0.6 mmol) was degassed with Ar for 5 min in a quartz test-tube. Heptan-4-one (23 mg, 0.2 mmol) and water (0.5 mL, degassed prior to use) were then added. The solution was stirred vigorously and irradiated at 25 °C for 16 h in a Rayonet RPR-100 photochemical reactor. The reaction mixture was transferred into a pressure tube, iodomethane (0.1 mL, 2 mmol) was added, and the reaction mixture was stirred at 60 °C for 4 h according to GPXX. The reaction mixture was then extracted with EtOAc (3 × 15 mL). The organic phases were combined, dried over anhydrous Na_2SO_4 and concentrated under reduced pressure. The crude product was purified by flash chromatography on silica gel (EtOAc/hexane, 1 : 5 v/v) to give two separable isomers (24 mg, 62%, 1 : 2.6 ratio of sulfone **S1** and **S2**) as a colorless oil.

In HFIP: According to GP1, a stirred mixture of hexafluoro-2-propanol (3 mL) and $Na_2S_2O_5$ (114 mg, 0.6 mmol) was degassed with Ar for 5 min in a quartz test-tube. Heptan-4-one (23 mg, 0.2 mmol) and water (0.75 mL, degassed prior to use) were then added. The solution was stirred vigorously and irradiated at 25 °C for 16 h in a Rayonet RPR-100 photochemical reactor. The reaction mixture was transferred into a pressure tube, iodomethane (0.1 mL, 2 mmol) was

Go back to table of contents

added, and the reaction mixture was stirred at 60 °C for 4 h. The reaction mixture was then extracted with EtOAc (3 × 15 mL). The organic phases were combined, dried over anhydrous Na_2SO_4 and concentrated under reduced pressure. The crude product was purified by flash chromatography on silica gel (EtOAc/hexane, 1 : 5 v/v) to give two separable isomers (26 mg, 67%, 3 : 1 ratio of sulfone **S1** and **S2**) as a colorless oil.

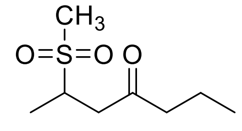

^{1}H NMR (500 MHz, $CDCl_3$): 3.59 (1H, dqd, *J* = 13.7, 6.9, 4.1 Hz), 3.15 (1H, dd, *J* = 18.0, 3.9 Hz), 2.84 (3H, s), 2.57 (1H, dd, *J* = 18.1, 8.7 Hz), 2.52–2.34 (2H, m), 1.65–1.55 (2H, m), 1.37 (3H, d, *J* = 6.9 Hz), 0.90 (3H, t, *J* = 7.4 Hz) ppm. – ^{13}C NMR (125 MHz, $CDCl_3$): 206.6, 54.4, 45.2, 41.4, 38.4, 17.2, 14.6, 13.7 ppm. – IR: 3003, 2253, 1709, 1419, 1358, 1220, 1092, 919, 732, 647, 622, 528 cm^{-1}. – HRMS calcd for $C_8H_{17}O_3S$: 193.0893, found 193.0895 [M+H$^+$].

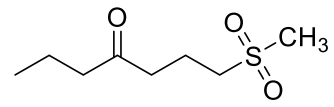

^{1}H NMR (500 MHz, $CDCl_3$): 3.03 (2H, t, *J* = 7.5 Hz), 2.90 (3H, s), 2.64 (2H, t, *J* = 6.7 Hz), 2.37 (2H, t, *J* = 7.3 Hz), 2.13–2.01 (2H, m), 1.63–1.51 (2H, m), 0.89 (3H, t, *J* = 7.4 Hz) ppm. – ^{13}C NMR (125 MHz, $CDCl_3$): 209.6, 53.6, 44.8, 40.6, 40.2, 17.3, 16.8, 13.8 ppm. – IR: 3005, 1709, 1418, 1358, 1220, 1092, 920, 734, 648, 623, 528 cm^{-1}. – HRMS calcd for $C_8H_{17}O_3S$: 193.0893, found 193.0896 [M+H$^+$].

Photoinduced C–H sulfination of methyl cyclopentanecarboxylate: sulfones S3-S5

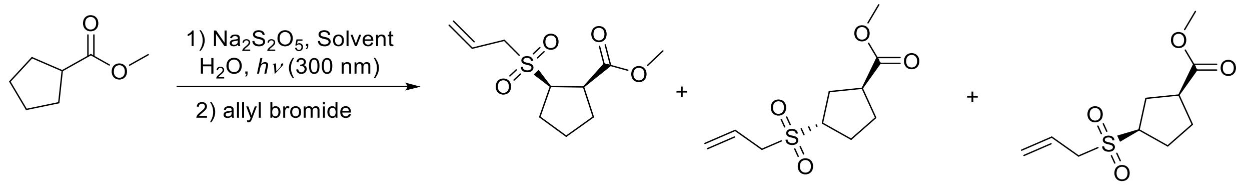

S3 **S4** **S5**

In MeCN: According to GP1, a stirred mixture of MeCN (3 mL) and $Na_2S_2O_5$ (57 mg, 0.3 mmol) was degassed with Ar for 5 min in a quartz test-tube. Ethyl cyclopentanecarboxylate (26 mg, 0.2 mmol) and water (0.75 mL, degassed prior to use) were then added. The solution was stirred vigorously and irradiated at 25 °C for 16 h in a Rayonet RPR-100 photochemical reactor. The reaction mixture was transferred into a pressure tube, allyl bromide (0.2 mL, 2 mmol) was added, and the reaction mixture was stirred at 60 °C for 4 h. The reaction mixture was then extracted with EtOAc (3 × 15 mL). The organic phases were combined, dried over anhydrous Na_2SO_4 and concentrated under reduced pressure. The crude product was purified by flash chromatography

Go back to table of contents

on silica gel (EtOAc/hexane, 1 : 5 v/v) to give three separable isomers (37 mg, 75%, 3 : 1 : 1 ratio of isomers **S3**, **S4**, **S5**) as a colorless oil.

In HFIP: According to GP1, a stirred mixture of hexafluoro-2-propanol (3 mL) and $Na_2S_2O_5$ (57 mg, 0.3 mmol) was degassed with Ar for 5 min in a quartz test-tube. Ethyl cyclopentanecarboxylate (26 mg, 0.2 mmol) and water (0.75 mL, degassed prior to use) were then added. The solution was stirred vigorously and irradiated at 25 °C for 16 h in a Rayonet RPR-100 photochemical reactor. The reaction mixture was transferred into a pressure tube, allyl bromide (0.2 mL, 2 mmol) was added, and the reaction mixture was stirred at 60 °C for 4 h. The reaction mixture was then extracted with EtOAc (3 × 15 mL). The organic phases were combined, dried over anhydrous Na_2SO_4 and concentrated under reduced pressure. The crude product was purified by flash chromatography on silica gel (EtOAc/hexane, 1 : 5 v/v) to give three separable isomers (37 mg, 75%, 1.1 : 1.4 : 1 ratio of isomers **S3**, **S4**, **S5**) as a colorless oil.

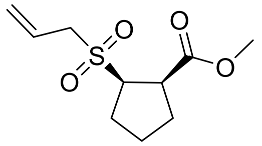

^{1}H NMR (500 MHz, $CDCl_3$): 5.95–5.77 (1H, m), 5.47–5.31 (2H, m), 3.89 (1H, dt, *J* = 9.2, 6.4 Hz), 3.74–3.64 (5H, m), 3.30 (1H, dt, *J* = 8.6, 6.5 Hz), 2.20–2.01 (3H, m), 1.90–1.66 (3H, m) ppm. – ^{13}C NMR (125 MHz, $CDCl_3$): 174.2, 124.7, 124.6, 62.1, 57.0, 52.5, 44.7, 31.8, 27.4, 25.9 ppm. – IR: 2954, 2875, 1731, 1639, 1437, 1370, 1305, 1227, 1198, 1177, 1130, 1083, 1032, 995, 938, 879, 768, 631, 535 cm^{-1}. – HRMS calcd for $C_{10}H_{17}O_4S$: 233.0842, found 233.0847 [M+H$^+$].

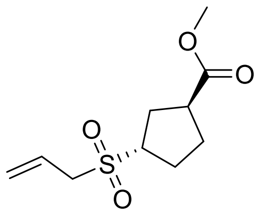

^{1}H NMR (500 MHz, $CDCl_3$): 5.99–5.80 (1H, m), 5.52–5.36 (2H, m), 3.73–3.56 (6H, m), 3.05–2.98 (1H, m), 2.35–2.18 (2H, m), 2.19–2.05 (3H, m), 1.92–1.82 (1H, m) ppm. – ^{13}C NMR (125 MHz, $CDCl_3$): 175.2, 125.0, 124.6, 58.8, 56.8, 52.1, 43.4, 29.9, 29.8, 26.1 ppm. – IR: 3003, 1708, 1422, 1359, 1311, 1221, 1131, 1092, 901, 630, 529 cm^{-1}. – HRMS calcd for $C_{10}H_{17}O_4S$: 233.0842, found 233.0849 [M+H$^+$].

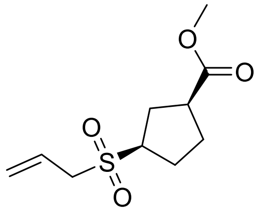

^{1}H NMR (500 MHz, $CDCl_3$): 5.96–5.82 (1H, m), 5.48–5.37 (2H, m), 3.70–3.61 (5H, m), 3.52–3.41 (1H, m), 2.88–2.73 (1H, m), 2.30 (2H, t, *J* = 8.9 Hz), 2.24–2.15 (1H, m), 2.04–1.91 (3H, m) ppm. – ^{13}C NMR (125 MHz, $CDCl_3$): 174.0, 125.0, 124.4, 59.0, 56.6, 52.0, 43.9, 30.0, 28.9, 25.7 ppm. – IR: 2954, 1731, 1713, 1640, 1437, 1398, 1363, 1291, 1243, 1221, 1172, 1129, 1087, 1029, 995, 913, 756, 734, 631, 593, 532 cm^{-1}. – HRMS HMRS calcd for $C_{10}H_{17}O_4S$: 233.0842, found 233.0847 [M+H$^+$].

Go back to table of contents

Sulfonyl derivatves

Photoinduced C–H sulfination of cycloheptane: cycloheptanesulfinic acid (37)

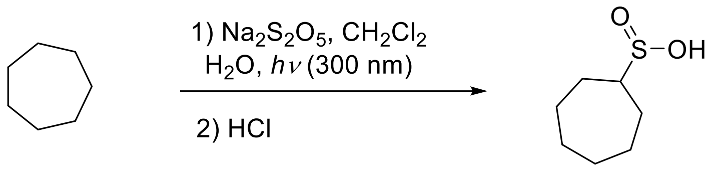

37

According to GP1, a stirred mixture of dichloromethane (3 mL) and $Na_2S_2O_5$ (190 mg, 1 mmol) was degassed with Ar for 5 min in a quartz test-tube. Cycloheptane (49 mg, 0.5 mmol) and water (0.75 mL, degassed prior to use) were then added. The solution was stirred vigorously and irradiated at 25 °C for 16 h in a Rayonet RPR-100 photochemical reactor., 12M aqueous HCl (0.1 mL) was added, and the reaction mixture was stirred at room temperature for 10 min. The reaction mixture was concentrated under nitrogen to dryness. EtOAc (15 mL) was added to the flask and the flask was sonicated, filtered through celite, the organic phases were concentrated under nitrogen to give sulfinic acid **37** (64 mg, 80%) as a colorless oil.

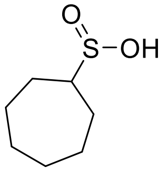

^{1}H NMR (300 MHz, CD_3CN): 8.50 (1 H, s), 2.66–2.49 (1 H, m), 2.16–1.97 (2 H, m), 1.88–1.74 (2 H, m), 1.70–1.46 (8 H, m) ppm. – ^{13}C NMR (75 MHz, CD_3CN): 66.0, 29.2, 27.0, 26.3 ppm. – IR: 3336, 2927, 1025, 632, 534 cm^{-1}. – HRMS calcd for $C_7H_{15}O_2S$: 163.0787, found 163.0785 [M+H$^+$].

Photoinduced C–H sulfination of cycloheptane: sodium cycloheptanesulfinate (38)

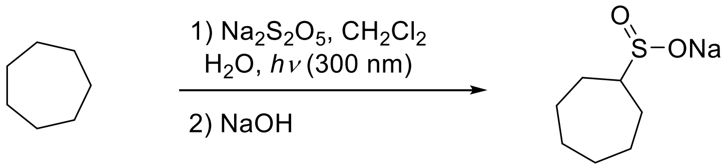

38

According to GP1, a stirred mixture of dichloromethane (3 mL) and $Na_2S_2O_5$ (190 mg, 1 mmol) was degassed with Ar for 5 min in a quartz test-tube. Cycloheptane (49 mg, 0.5

Go back to table of contents

mmol) and water (0.75 mL, degassed prior to use) were then added. The solution was stirred vigorously and irradiated at 25 °C for 16 h in a Rayonet RPR-100 photochemical reactor., 1M NaOH in methanol solution (1 mL) was added, and the reaction mixture was stirred at room temperature for 10 min. The reaction mixture was concentrated under nitrogen to dryness. The crude product was purified by flash chromatography on silica gel (MeOH/DCM, 1:10 v/v) to give sulfinate **38** (69 mg, 75%) as a white solid.

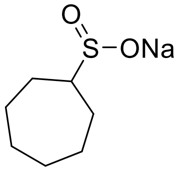

m.p. >250 °C. – ^{1}H NMR (300 MHz, CD_3OD): 2.06–1.88 (3 H, m), 1.83–1.68 (2 H, m), 1.66–1.25 (8 H, m) ppm. – ^{13}C NMR (75 MHz, CD_3OD): 68.2, 29.4, 27.5, 26.9 ppm. – IR: 3327, 2926, 1646, 1458, 953, 814, 632, 534 cm^{-1}. – HRMS calcd for $C_7H_{15}O_2S$: 163.0787, found 163.0787 [M+H$^+$].

Photoinduced C–H sulfination of cyclohexane: 4-(cyclohexylsulfonyl)morpholine (39)

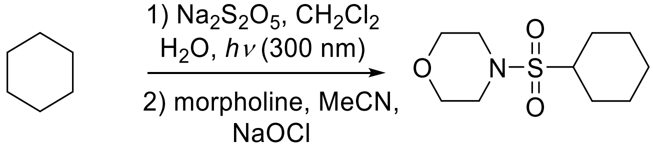

39

According to GP1, a stirred mixture of dichloromethane (3 mL) and $Na_2S_2O_5$ (190 mg, 1 mmol) was degassed with Ar for 5 min in a quartz test-tube. Cyclohexane (42 mg, 0.5 mmol) and water (0.75 mL, degassed prior to use) were then added. The solution was stirred vigorously and irradiated at 25 °C for 16 h in a Rayonet RPR-100 photochemical reactor. The reaction mixture was concentrated under nitrogen to remove the dichloromethane, 3 mL MeCN was added. Morpholine (131 mg, 1.5 mmol) and a 1.41 M NaOCl solution (1.06 mL) were added dropwise at 0 °C, and the reaction mixture was stirred at room temperature for 8 h. The reaction mixture was then extracted with EtOAc (3 × 15 mL). The organic phases were combined, dried over anhydrous Na_2SO_4 and concentrated under reduced pressure. The crude product was purified by flash

Go back to table of contents

chromatography on silica gel (EtOAc/hexane, 1 : 5 v/v) to give sulfonamide **39** (88 mg, 76%) as a white solid.

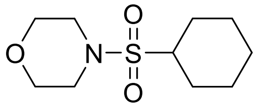

m.p. 66–68 °C. – ^{1}H NMR (500 MHz, $CDCl_3$): 3.87–3.48 (4 H, m), 3.33–3.15 (4 H, m), 2.96–2.67 (1 H, m), 2.06 (2 H, d, J = 12.0 Hz), 1.83 (2 H, d, J = 13.2 Hz), 1.65 (1 H, d, J = 12.2 Hz), 1.45 (2 H, qd, J = 12.4, 2.6 Hz), 1.30–1.06 (3 H, m) ppm. – ^{13}C NMR (125 MHz, $CDCl_3$): 67.02, 61.23, 46.35, 26.54, 25.16, 25.12. ppm. – IR: 3003, 1709, 1421, 1358, 1261, 1220, 1147, 1116, 1092, 953, 901, 676, 627, 577, 528 cm^{-1}. – HRMS calcd for $C_{10}H_{20}NO_3S$: 234.1158, found 234.1161 [M+H$^+$].

Photoinduced C–H sulfination of cyclopentane: 2-(cyclopentylsulfonyl)-1,2,3,4-tetrahydroisoquinoline (40)

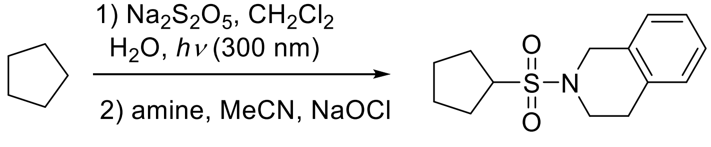

40

According to GP1, a stirred mixture of dichloromethane (3 mL) and $Na_2S_2O_5$ (190 mg, 1 mmol) was degassed with Ar for 5 min in a quartz test-tube. Cyclopentane (35 mg, 0.5 mmol) and water (0.75 mL, degassed prior to use) were then added. The solution was stirred vigorously and irradiated at 25 °C for 16 h in a Rayonet RPR-100 photochemical reactor. The reaction mixture was concentrated under nitrogen to remove the dichloromethane, 3 mL MeCN was added. 1,2,3,4-Tetrahydroisoquinoline (133 mg, 1 mmol) and a 1.41 M NaOCl solution (1.06 mL) were added dropwise at 0 °C, and the reaction mixture was stirred at room temperature for 8 h. The reaction mixture was then extracted with EtOAc (3 × 15 mL). The organic phases were combined, dried over anhydrous Na_2SO_4 and concentrated under reduced pressure. The crude product was purified by flash chromatography on silica gel (EtOAc/hexane, 1 : 5 v/v) to give sulfonamide **40** (90 mg, 68%) as a white solid.

Go back to table of contents

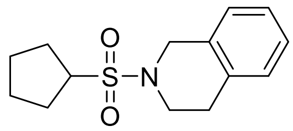

m.p. 55–57 ºC. – ^{1}H NMR (500 MHz, $CDCl_3$): 7.20–7.09 (3 H, m), 7.09–7.01 (1 H, m), 4.51 (s, 2H), 3.61 (2 H, t, *J* = 5.9 Hz), 3.52 (1 H, p, *J* = 8.1 Hz), 2.93 (2 H, t, *J* = 5.8 Hz), 2.12–1.88 (4 H, m), 1.84–1.72 (2 H, m), 1.66–1.52 (2 H, m) ppm. – ^{13}C NMR (125 MHz, $CDCl_3$): 133.5, 132.4, 129.1, 126.8, 126.4, 126.2, 60.9, 47.5, 43.7, 29.4, 28.1, 25.7. ppm. – IR: 2954, 2868, 1497, 1452, 1318, 1141, 1022, 1101, 954, 759 cm^{-1}. – HRMS calcd for $C_{14}H_{19}NO_2S$: 266.1209, found 266.1207 [M+H$^+$].

Photoinduced C–H sulfination of cyclopentane: (*R*)-*N*-(1-(4-methoxyphenyl)ethyl)cyclopentanesulfonamide (41)

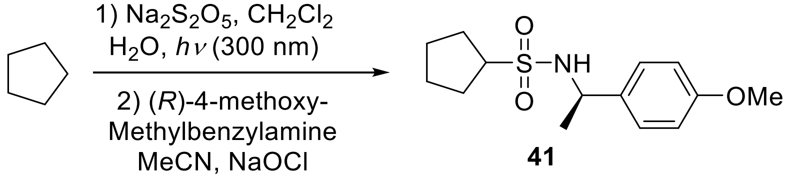

According to GP1, a stirred mixture of dichloromethane (3 mL) and $Na_2S_2O_5$ (190 mg, 1 mmol) was degassed with Ar for 5 min in a quartz test-tube. Cyclopentane (35 mg, 0.5 mmol) and H_2O (0.75 mL, degassed prior to use) were then added. The solution was stirred vigorously and irradiated at 25 °C for 16 h in a Rayonet RPR-100 photochemical reactor. The reaction mixture was concentrated under nitrogen to remove the dichloromethane, 3 mL MeCN was added. (*R*)-(+)-4-methoxy-α-methylbenzylamine (151 mg, 1 mmol) and 1.41 M NaOCl solution (1.06 mL) were added dropwise at 0 °C, and the reaction mixture was stirred at room temperature for 8 h. The reaction mixture was then extracted with EtOAc (3 × 15 mL). The organic phases were combined, dried over anhydrous Na_2SO_4 and concentrated under reduced pressure. The crude product was purified by flash chromatography on silica gel (EtOAc/hexane, 1:5 v/v) to give sulfonamide **41** (93.5 mg, 66%) as a white solid.

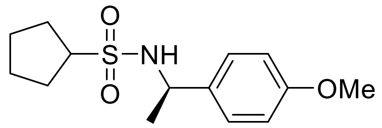

m.p. 112–114 °C. – ^{1}H NMR (500 MHz, $CDCl_3$): 7.25 (2H, d, *J* = 8.6 Hz), 6.88 (2H, d, *J* = 8.6 Hz), 4.75–4.39 (2H, m), 3.81 (3H,

Go back to table of contents

s), 3.14–2.92 (1H, m), 2.05–1.92 (1H, m), 1.87 (2H, dt, J = 13.3, 6.8 Hz), 1.76–1.62 (3H, m), 1.58–1.41 (5H, m) ppm. – ^{13}C NMR (125 MHz, $CDCl_3$): 159.2, 135.3, 127.5, 114.2, 62.4, 55.4, 53.3, 28.4, 27.8, 26.2, 25.9, 24.6. ppm. – IR: 3274, 2957, 2870, 1612, 1514, 1449, 1305, 1246, 1145, 601 cm^{-1}. – HRMS calcd for $C_{14}H_{21}NaNO_3S$: 306.1134, found 306.1136 [M+Na$^+$].

Photoinduced C–H sulfination of 2,6-dimethyl-4-heptanone: 2,6-dimethyl-1-(morpholinosulfonyl)heptan-4-one (42)

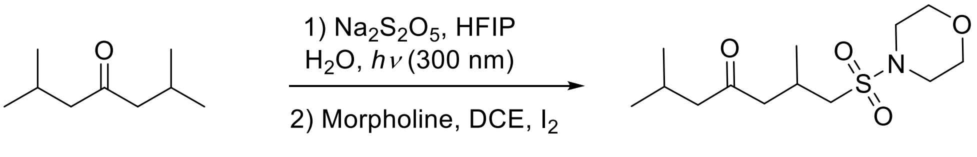

42

According to GP1, a stirred mixture of hexafluoroisopropanol (3 mL) and $Na_2S_2O_5$ (114 mg, 0.6 mmol) was degassed with Ar for 5 min in a quartz test-tube. 2,6-Dimethyl-4-heptanone (28 mg, 0.2 mmol) and water (0.75 mL, degassed prior to use) were then added. The solution was stirred vigorously and irradiated at 25 °C for 16 h in a Rayonet RPR-100 photochemical reactor. The reaction mixture was concentrated under nitrogen to dryness, 5 mL 1,2-dichloroethane was added. Morpholine (9 mg, 0.1 mmol) and iodine (42 mg, 0.4 mmol) were added, and the reaction mixture was stirred under air at room temperature for 16 h. The reaction mixture was then washed with saturated sodium thiosulfate (10 mL) and extracted with EtOAc (3 × 15 mL). The organic phases were combined, dried over anhydrous Na_2SO_4 and concentrated under reduced pressure. The crude product was purified by flash chromatography on silica gel (EtOAc/hexane, 1 : 5 v/v) to give sulfonamide **42** (16 mg, 56%) as a colorless oil.

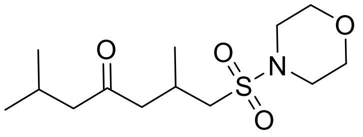

^{1}H NMR (500 MHz, $CDCl_3$): 3.77–3.70 (4 H, m), 3.23 (4 H, dd, J = 5.7, 3.6 Hz), 3.02 (1 H, dd, J = 13.8, 6.0 Hz), 2.79 (1 H, dd, J = 13.8, 6.5 Hz), 2.67 (1 H, dd, J = 17.3, 6.2 Hz), 2.59 (1 H, dd, J = 12.8, 6.4 Hz), 2.48 (1 H, dd, J = 17.3, 6.0 Hz), 2.25 (2 H, d, J = 7.0 Hz), 2.11 (1 H, dt, J = 13.5,

Go back to table of contents

6.7 Hz), 1.14 (3 H, d, J = 6.8 Hz), 0.89 (6 H, d, J = 6.6 Hz) ppm. – ^{13}C NMR (125 MHz, $CDCl_3$): 209.4, 66.6, 53.1, 52.3, 48.4, 45.8, 25.3, 24.7, 22.6, 20.3 ppm. – IR: 3023, 2982, 1731, 1373, 1241, 1045, 912, 847, 750, 670, 636, 608 cm^{-1}. – HRMS calcd for $C_{13}H_{26}NO_4S$: 292.1577, found 292.158 [M+H$^+$].

Photoinduced C–H sulfination of 2,6-dimethyl-4-heptanone: 1-((3,4-Dihydroisoquinolin-2(1*H*)-yl)sulfonyl)-2,6-dimethylheptan-4-one (43)

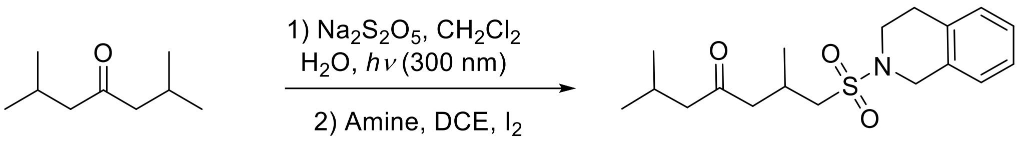

43

According to GP1, a stirred mixture of hexafluoroisopropanol (3 mL) and $Na_2S_2O_5$ (114 mg, 0.6 mmol) was degassed with Ar for 5 min in a quartz test-tube. 2,6-Dimethyl-4-heptanone (28 mg, 0.2 mmol) and water (0.75 mL, degassed prior to use) were then added. The solution was stirred vigorously and irradiated at 25 °C for 16 h in a Rayonet RPR-100 photochemical reactor. The reaction mixture was concentrated under nitrogen to dryness, 5 mL 1,2-dichloroethane was added. 1,2,3,4-Tetrahydroisoquinoline (13.3 mg, 0.1 mmol) and iodine (42 mg, 0.4 mmol) were added, and the reaction mixture was stirred under air at room temperature for 16 h. The reaction mixture was then washed with saturated sodium thiosulfate (10 mL) and extracted with EtOAc (3 × 15 mL). The organic phases were combined, dried over anhydrous Na_2SO_4 and concentrated under reduced pressure. The crude product was purified by flash chromatography on silica gel (EtOAc/hexane, 1 : 5 v/v) to give sulfonamide **43** (18 mg, 54%) as a colorless oil.

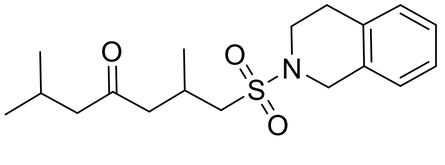

^{1}H NMR (300 MHz, $CDCl_3$): 7.21–7.12 (3 H, m), 7.11–7.04 (1 H, m), 3.64–3.48 (1 H, m), 3.09 (2 H, dd, J = 13.9, 6.2 Hz), 2.95 (2 H, dd, J = 14.3, 8.6 Hz), 2.88 (1 H, dd, J = 13.9, 6.3 Hz), 2.72 (2 H, dd, J = 17.3, 6.0 Hz), 2.62 (1 H, tt, J = 12.9, 6.4 Hz), 2.49 (1 H, dd, J = 17.3,

Go back to table of contents

6.4 Hz), 2.26 (2 H, d, J = 7.0 Hz), 2.18 – 2.07 (1 H, m), 1.16 (3 H, d, J = 11.5 Hz), 0.90 (6 H, d, J = 6.6 Hz) ppm. – ^{13}C NMR (75 MHz, $CDCl_3$): 209.6, 133.5, 132.1, 129.2, 127.0, 126.6, 126.4, 54.6, 52.4, 48.5, 47.2, 43.5, 29.2, 25.5, 24.7, 22.7, 20.3 ppm. – IR: 3390, 2953, 2871, 1453, 1376, 1307, 1240, 1101, 1017, 969, 922 cm^{-1}. – HRMS calcd for $C_{18}H_{28}NO_3S$: 338.1784, found 338.1785 [M+H$^+$].

Photoinduced C–H sulfination of 2,6-dimethyl-4-heptanone: 2,6-dimethyl-4-oxoheptane-1-sulfonyl fluoride (44)

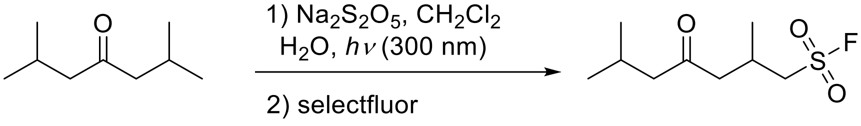

44

According to GP1, a stirred mixture of hexafluoroisopropanol (3 mL) and $Na_2S_2O_5$ (114 mg, 0.6 mmol) was degassed with Ar for 5 min in a quartz test-tube. 2,6-Dimethyl-4-heptanone (28 mg, 0.2 mmol) and water (0.75 mL, degassed prior to use) were then added. The solution was stirred vigorously and irradiated at 25 °C for 16 h in a Rayonet RPR-100 photochemical reactor. The reaction mixture was concentrated under nitrogen to remove the hexafluoroisopropanol, 3 mL 1,4-dioxane was added The reaction mixture was transferred into a 20mL vial, selectfluor (212 mg, 0.6 mmol) was added, and the reaction mixture was stirred at room temperature for 4 h. The reaction mixture was then extracted with EtOAc (3 × 15 mL). The organic phases were combined, dried over anhydrous Na_2SO_4 and concentrated under reduced pressure. The crude product was purified by flash chromatography on silica gel (EtOAc/hexane, 1 : 5 v/v) to give sulfonyl fluoride **44** (28 mg, 63%) as a colorless oil.

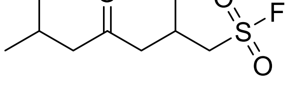

^{1}H NMR (500 MHz, $CDCl_3$): 3.58 (1 H, ddd, J = 14.7, 5.1, 4.3 Hz), 3.38 (1 H, ddd, J = 14.6, 6.5, 3.0 Hz), 2.78–2.48 (3 H, m), 2.27 (2 H, d, J = 7.0 Hz), 2.12 (1 H, dt, J = 13.5, 6.7 Hz), 1.19 (3 H, d, J = 6.6 Hz), 0.90 (6 H, d, J = 6.7 Hz) ppm. – ^{13}C NMR (125 MHz, $CDCl_3$): 208.6, 55.8, 55.7, 52.3, 47.4, 25.6, 24.7, 22.6, 19.4 ppm. – ^{19}F NMR (471 MHz, $CDCl_3$): 59.8 ppm. – IR: 2961, 1710, 1465, 1402, 1215, 908, 803, 754,

732, 670, 850, 631, 580, 534 cm^{-1}. – HRMS calcd for $C_9H_{18}FO_3S$: 225.0955, found 225.0957 [M+H^+].

Photoinduced C–H sulfination of 3-pentanone: 3-oxopentane-1-sulfonyl fluoride (45)

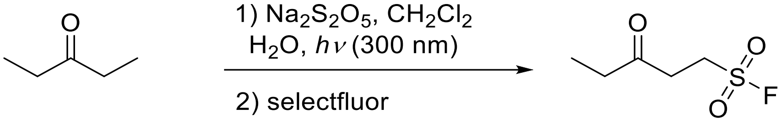

45

According to GP1, a stirred mixture of dichloromethane (3 mL) and $Na_2S_2O_5$ (190 mg, 1 mmol) was degassed with Ar for 5 min in a quartz test-tube. 3-Pentanone (26 mg, 0.3 mmol) and water (0.75 mL, degassed prior to use) were then added. The solution was stirred vigorously and irradiated at 25 °C for 16 h in a Rayonet RPR-100 photochemical reactor. The reaction mixture was transferred into a 20mL vial, Selectfluor (318 mg, 0.9 mmol) was added, and the reaction mixture was stirred at room temperature for 4 h. The reaction mixture was then extracted with EtOAc (3 × 15 mL). The organic phases were combined, dried over anhydrous Na_2SO_4 and concentrated under reduced pressure. The crude product was purified by flash chromatography on silica gel (EtOAc/hexane, 1 : 5 v/v) to give sulfonyl fluoride **45** (29 mg, 57%) as a colorless oil.

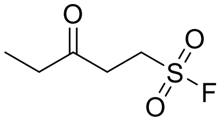

^1H NMR (500 MHz, $CDCl_3$): 3.70 (2 H, td, *J* = 7.3, 5.1 Hz), 3.05 (2 H, t, *J* = 7.4 Hz), 2.53 (2 H, q, *J* = 7.3 Hz), 1.12 (3 H, t, *J* = 7.3 Hz) ppm. – ^{13}C NMR (125 MHz, $CDCl_3$): 205.2, 45.4, 45.3, 36.1, 35.5, 7.8. ppm. – IR: 2984, 2255, 1732, 1446, 1373, 1238, 1096, 1045, 916, 847, 732, 648, 631, 608, 530 cm^{-1}. – HRMS calcd for $C_5H_9FNaO_3S$: 191.0149, found 191.0151 [M+H^+].

Go back to table of contents

Photoinduced C–H sulfination of cyclohexane: 7-chloro-4-(cyclohexylsulfonyl)quinoline (46)

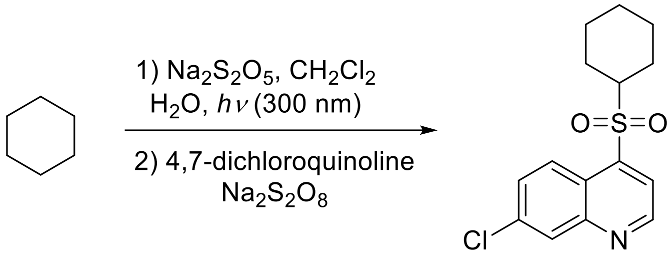

46

According to GP1, a stirred mixture of dichloromethane (3 mL) and $Na_2S_2O_5$ (190 mg, 1 mmol) was degassed with Ar for 5 min in a quartz test-tube. Cyclohexane (42 mg, 0.5 mmol) and water (0.75 mL, degassed prior to use) were then added. The solution was stirred vigorously and irradiated at 25 °C for 16 h in a Rayonet RPR-100 photochemical reactor. The reaction mixture was transferred into a 20 mL vial, 4,7-dichloroquinoline (50 mg, 0.25 mmol) and $Na_2S_2O_8$ (238 mg, 1 mmol) were added, and the reaction mixture was stirred at room temperature for 2 h. The reaction mixture was then extracted with EtOAc (3 × 15 mL). The organic phases were combined, dried over anhydrous Na_2SO_4 and concentrated under reduced pressure. The crude product was purified by flash chromatography on silica gel (EtOAc/hexane, 1 : 5 v/v) to give sulfone **46** (49 mg, 64%) as a white solid.

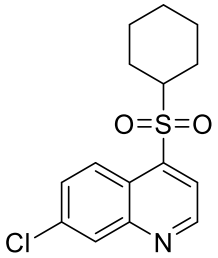

m.p. 169–171 °C. – ^{1}H NMR (300 MHz, $CDCl_3$): 9.12 (1 H, d, J = 4.1 Hz), 8.69 (1 H, d, J = 9.2 Hz), 8.24 (1 H, d, J = 2.1 Hz), 7.99 (1 H, d, J = 4.4 Hz), 7.68 (1 H, dd, J = 9.2, 2.2 Hz), 3.23–2.91 (1 H, m), 2.08–1.78 (4 H, m), 1.78–1.40 (3 H, m), 1.34–1.01 (3 H, m) ppm. – ^{13}C NMR (75 MHz, $CDCl_3$): 150.7, 149.9, 141.8, 136.7, 130.0, 129.6, 125.9, 123.5, 63.9, 25.1, 25.0.ppm. – IR: 3344, 2940, 1723, 1606, 1490, 1453, 1373, 1315, 1244, 1204, 1184, 1156, 1133, 1067, 974, 927, 849, 829, 756, 688, 633, 618, 575, 563, 537 cm^{-1}. – HRMS $C_{15}H_{17}ClNO_2S$: 310.0663, found 310.0659 [M+H$^+$].

Go back to table of contents

Photoinduced C–H sulfination of cyclopentane: sodium $^{18}O_2$-cyclopentanesulfinate (47)

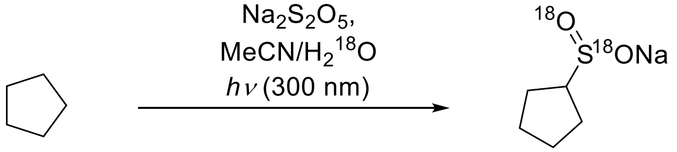

47

According to GP1, a stirred mixture of acetonitrile (2.5 mL) and $Na_2S_2O_5$ (68 mg, 0.36 mmol) was degassed with Ar for 5 min in a quartz test-tube. Cyclopentane (21 mg, 0.3 mmol) and $H_2{}^{18}O$ (0.5 mL) were then added. The solution was stirred vigorously and irradiated at 25 °C for 36 h in a Rayonet RPR-100 photochemical reactor. The reaction mixture was concentrated under nitrogen to dryness. The crude product was purified by flash chromatography on silica gel (MeOH/DCM, 1 : 10 v/v) to give sulfinate **45** (30 mg, 64%, 95% ^{18}O isotopic purity determined by HRMS) as a white solid.

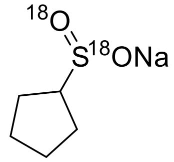

m.p. >250 °C. – ^{1}H NMR (500 MHz, CD_3OD): 3.25 (1 H, p, *J* = 8.0 Hz), 1.96 (4 H, tdd, *J* = 7.6, 5.0, 1.8 Hz), 1.80–1.70 (2 H, m), 1.59 (2 H, dddd, *J* = 12.1, 9.8, 6.6, 4.0 Hz) ppm. – ^{13}C NMR (125 MHz, CD_3OD): 61.1, 29.9, 27.0 ppm. – IR: 3328, 2935, 1662, 1425, 994, 985, cm^{-1}. – HRMS calcd for $C_5H_9{}^{18}O_2S$: 137.0403, found 137.0407 [M^-].

Go back to table of contents

Photoinduced C–H sulfination of cyclopentane: 7-chloro-4-$^{18}O_2$-(cyclopentylsulfonyl)quinoline (48)

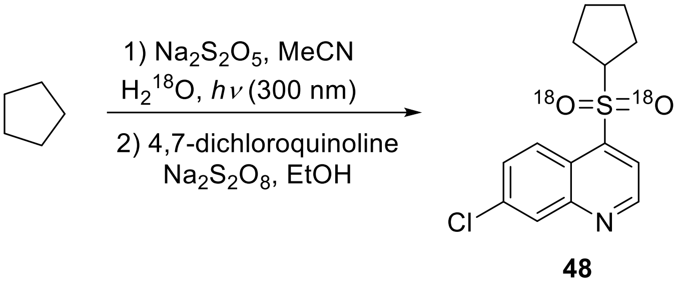

48

According to GP1, a stirred mixture of MeCN (1.7 mL) and $Na_2S_2O_5$ (46 mg, 0.24 mmol) was degassed with Ar for 5 min in a quartz test-tube. Cyclopentane (14 mg, 0.2 mmol) and $H_2{}^{18}O$ (0.3 mL) were then added. The solution was stirred vigorously and irradiated at 25 °C for 16 h in a Rayonet RPR-100 photochemical reactor. The reaction mixture was transferred into a 20 mL vial, 4,7-dichloroquinoline (20 mg, 0.1 mmol), $Na_2S_2O_8$ (71 mg, 0.3 mmol) and ethanol (2 mL) were added, and the reaction mixture was stirred at room temperature for 2 h. The reaction mixture was then extracted with EtOAc (3 × 15 mL). The organic phases were combined, dried over anhydrous Na_2SO_4 and concentrated under reduced pressure. The crude product was purified by flash chromatography on silica gel (EtOAc/hexane, 1 : 5 v/v) to give sulfone **48** (23 mg, 75%, 95% ^{18}O isotopic purity determined by HRMS) as a white solid.

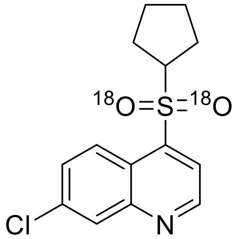

m.p. 163–165 °C. – ^1H NMR (500 MHz, $CDCl_3$): 9.13 (1 H, d, J = 4.4 Hz), 8.70 (1 H, d, J = 9.1 Hz), 8.26 (1 H, d, J = 2.1 Hz), 8.03 (1 H, d, J = 4.3 Hz), 7.69 (1 H, dd, J = 9.2, 2.2 Hz), 3.68 (1 H, tt, J = 8.1, 6.8 Hz), 2.16–2.05 (2 H, m), 1.82 (4 H, tdd, J = 8.9, 7.0, 4.1 Hz), 1.67–1.57 (2 H, m) ppm. – ^{13}C NMR (125 MHz, $CDCl_3$): 151.0, 150.1, 143.1, 136.8, 130.1, 129.8, 125.8, 122.8, 121.5, 64.4, 27.3, 26.1 ppm. – IR: 3335, 2928, 1802, 1615, 1482, 1463, 1362, 1325, 1223, 1205, 1195, 1165, 1162, 1035, 982 cm^{-1}. – HRMS $C_{14}H_{14}ClN^{18}O_2S$: 300.0591, found 300.0591 [M+H$^+$].

Go back to table of contents

Computational data

1. Software

Quantum chemical calculations were performed using the Stampede2 supercomputer at the Texas Advanced Computing Center (TACC) hosted by the University of Texas in Austin, Texas.[10] DFT geometry optimization, vibrational frequency, and IRC calculations were conducted using Gaussian 16 (rA.03).[11] The CREST utility[12] of the xTB software suite[13,14] was used in conjunction with manual conformational searching to locate initial starting geometries for optimization via DFT. Final images of minima and transition state geometries were rendered using CYLview.[15] ORCA[16,17] version 4.2.1 was used to calculate DLPNO[18]-CCSD(T)[19,20] / cc-pVTZ[21] single point energies. Energy decomposition analysis was performed with Q-Chem 5.4.0.[22] NBO and SOPT analyses were performed with the NBO 7.0 program suite.[23] Routine visualization and monitoring of calculations was performed with Chemcraft.[24] Plots of the noncovalent interactions (NCIs) were generated using Multiwfn[25] and rendered in VMD.[26]

2. Details of Computational Methods

Gaussian 16 DFT calculations

Geometries of ground state minima and transition states were optimized without constraints using the D3[27] dispersion-corrected M06-2X[28] DFA and the def2-TZVP[29] basis set in the PCM solvation model.[30,31,32] Separate geometry optimizations were conducted in DCM ("dichloromethane"), MeCN ("acetonitrile"), and HFIP (generic; $\varepsilon = 16.7$,[33] $\varepsilon_{inf} = 1.625625$[34]) to account for solvent-dependent energy differences. Convergence criteria for these calculations was set to "tight" and an ultrafine grid was selected. Frequency calculations at the same level of theory were used to confirm the nature of the isolated stationary points. The quasi-harmonic approximation from Grimme[35] was applied via GoodVibes[36] to all structures to correct for potential errors associated with low

Go back to table of contents

magnitude vibrational frequencies using a cut-off frequency of 50 cm^{-1}. Geometries with zero imaginary frequencies were deemed minima whereas those with exactly one imaginary frequency along the chemical path of interest were deemed transition states. IRC calculations were performed to further corroborate that the located transition states connected reactants to products. Single point corrections of the above geometries were calculated at the ωB97XD[37] / def2-QZVPP[38] / PCM (solvent) level of theory and provided the final electronic component to the reported free energies. The M06-2X(D3) and ωB97XD DFAs were selected based on their excellent general performance in both thermochemical and NCI benchmarking studies.[39,40]

3. Electronic structure analysis of ^{3}**TS**$_A$

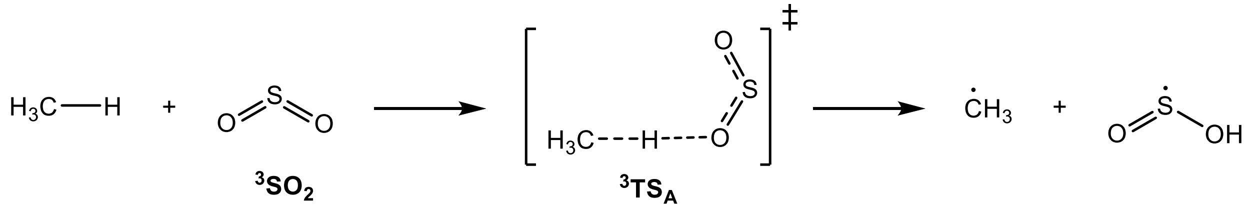

Several electronic structure analyses were performed in an effort to better understand the dominant electronic effects governing the HAT reaction between **CH_4** and **$^3SO_2$**. As the ALMO-EDA2 decomposition of the interaction energy (*vide infra*) was conducted in Q-Chem 5.4, the geometries of the gas phase minima for **CH_4**, **$^3SO_2$**, and **^3TS_A** were also optimized in Q-Chem at the M06-2X(D3) / def2-TZVP level of theory (SG-3 grid). The activation-strain/distortion interaction, ALMO-EDA2 energy decomposition, and NBO/SOPT analyses, as well as NCI plot utilize the Q-Chem optimized geometries. The free energies reported in Figure 2 refer to the Gaussian 16 optimized geometries. Additionally, all Kohn-Sham molecular orbitals (KS-MOs) were derived from the Gaussian 16 geometries. IRC calculations were used to derive the structures and free energies shown in Figure 2.B.

Go back to table of contents

Gaussian 16 M06-2X(D3) / def2-TZVP gas phase geometries

CH_4

E(RM062X) = -40.5002842996

Charge = 0 Multiplicity = 1

C	-0.254074785	-1.202515287	-1.439874969
H	0.177115389	-2.198073448	-1.35870327
H	-0.799607519	-1.118234137	-2.377416127
H	0.540571107	-0.459983086	-1.414803165
H	-0.934927752	-1.033198412	-0.608298498

$^3SO_2$

E(UM062X) = -548.512391044

Charge = 0 Multiplicity = 3

S	-1.7801057043	0.5034266337	0.1543253008
O	-3.2533388259	0.4285891145	-0.0096854142
O	-1.0643797899	0.8367126417	1.4110805836

^3TS_A

E(UM062X) = -589.007500802

Charge = 0 Multiplicity = 3

C	-0.2004082766	-1.3325906449	-1.4266472288
H	-0.3046449572	-1.8740299098	-2.3638436352
H	-0.8931650962	-0.5005475625	-1.3489119117
H	0.849687804	-0.8941177289	-1.4385951271
H	-0.2640024544	-1.9850551426	-0.5614536959
S	2.2254784853	0.5505867779	0.1467515497
O	0.9528272996	0.399098485	0.8633196049
O	2.2381871956	-0.2037442742	-1.2492195559

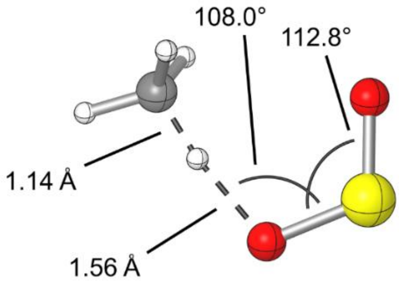

Go back to table of contents

CH_3

E(UM062X) = -39.8240152694

Charge = 0 Multiplicity = 2

C	-1.9647807545	-1.480366792	-1.1951321375
H	-1.7984865952	-1.9475311102	-2.1512325274
H	-2.3799050376	-0.4876470431	-1.1446459886
H	-1.7136336128	-2.0039880547	-0.2879193466

SO_2H

E(UM062X) = -549.204348331

Charge = 0 Multiplicity = 2

S	-1.7328236953	0.0083702296	0.177054192
O	-2.8650023496	0.9373769293	0.163400545
O	-0.5347874063	0.6945567178	1.0317970537
H	-0.8098451858	1.5873122473	1.2934123833

Q-Chem 5.4 M06-2X(D3) / def2-TZVP gas phase geometries

CH_4

E(RM062X) = -40.5001891867

Charge = 0 Multiplicity = 1

C	2.30396724	-0.183860075	-0.001021382
H	3.162743295	0.482884728	-0.004084343
H	2.251385995	-0.796167039	-0.895679124
H	1.387974897	0.491654081	-0.003862412
H	2.251735877	-0.788271397	0.899017761

Go back to table of contents

$^3SO_2$

E(UM062X) = -548.5054166

Charge = 0 Multiplicity = 3

S	-1.7801057043	0.5034266337	0.1543253008
O	-3.2533388259	0.4285891145	-0.0096854142
O	-1.0643797899	0.8367126417	1.4110805836

^3TS_A

E(UM062X) = -589.0003823

Charge = 0 Multiplicity = 3

C	2.30396724	-0.183860075	-0.001021382
H	3.162743295	0.482884728	-0.004084343
H	2.251385995	-0.796167039	-0.895679124
H	1.387974897	0.491654081	-0.003862412
H	2.251735877	-0.788271397	0.899017761
S	-1.133120695	0.11971034	0.002925564
O	-0.600975712	-1.248833254	0.005831246
O	0.007966842	1.222500262	-0.003458666

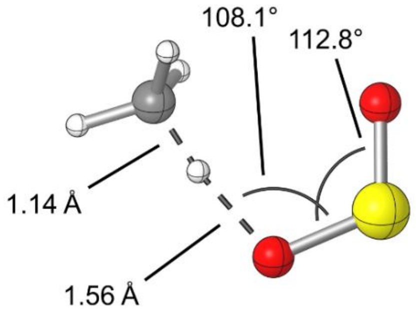

3.1 - Distortion/Interaction-Activation Strain Analysis of ^3TS_A

A distortion/interaction-activation strain analysis[41] was performed on the ^{3}**TS**$_A$ transition state geometry optimized in Q-Chem at the M06-2X(D3) / def2-TZVP level of theory. The electronic activation energy associated with HAT from **CH**$_4$ to ^{3}**SO**$_2$, $\Delta \boldsymbol{E}^{\ddagger}$, was partitioned into two terms:

$$\Delta \boldsymbol{E}^{\ddagger} = \Delta \boldsymbol{E}^{\ddagger}_{dist} + \Delta \boldsymbol{E}^{\ddagger}_{int} \qquad (1)$$

where $\Delta \boldsymbol{E}^{\ddagger}_{dist}$ is the sum of the strain energy required for **CH**$_4$ and ^{3}**SO**$_2$ to achieve their respective distorted geometries at the ^{3}**TS**$_A$ transition state structure:

Go back to table of contents

$$\Delta E^{\ddagger}_{dist} = \Delta E^{\ddagger}_{dist}(CH_4) + \Delta E^{\ddagger}_{dist}(^3SO_2) \quad (2)$$

$$\Delta E^{\ddagger}_{dist}(CH_4) = \Delta E_{dist}(CH_4) - \Delta E(CH_4) \quad (3)$$

$$\Delta E^{\ddagger}_{dist}(^3SO_2) = \Delta E_{dist}(^3SO_2) - \Delta E(^3SO_2) \quad (4)$$

and $\Delta E^{\ddagger}_{int}$ is the interaction energy between the two distorted fragments at the transition state geometry:

$$\Delta E^{\ddagger}_{int} = \Delta E^{\ddagger} - \Delta E^{\ddagger}_{dist} \quad (5)$$

3.2 - Energy decomposition analysis of ^3TS_A $\Delta E^{\ddagger}_{int}$ term (ALMO-EDA2)

The second generation Absolutely Localized Molecular Orbital Energy Decomposition Analysis (ALMO-EDA2) method of Head-Gordon and co-workers[42] was employed to gain insight into the intermolecular forces underlying the HAT reaction of **CH_4** and **$^3SO_2$** at the **^3TS_A** transition state. This method decomposes the interaction energy, $\Delta E^{\ddagger}_{int}$, into three initial terms:

$$\Delta E^{\ddagger}_{int} = \Delta E_{Frz} + \Delta E_{Pol} + \Delta E_{CT} \quad (1)$$

where ΔE_{Frz} is the difference between the energy of the isolated, non-interacting fragments and the "frozen density" energy, the energy associated with bringing the fragments together in the transition state geometry without allowing intrafragment orbital relaxation nor interfragment delocalization, thus isolating the ΔE_{Frz} term from polarization and charge transfer. The ΔE_{Pol} term is the energy contribution associated with polarization obtained by allowing the frozen fragment-localized orbitals to relax without interfragment orbital delocalization. Finally, the ΔE_{CT} term arises from the energy lowering effects of donor/acceptor interactions resulting from interfragment orbital delocalization(s).

Using ALMO-EDA2, the ΔE_{Frz} term can be further decomposed into three constituent terms:

$$\Delta \boldsymbol{E}_{\boldsymbol{Frz}} = \Delta \boldsymbol{E}_{\boldsymbol{Pauli}} + \Delta \boldsymbol{E}_{\boldsymbol{Elec}} + \Delta \boldsymbol{E}_{\boldsymbol{Disp}} \tag{2}$$

Where $\Delta \boldsymbol{E}_{\boldsymbol{Pauli}}$ represents Pauli repulsion, $\Delta \boldsymbol{E}_{\boldsymbol{Elec}}$ represents permanent electrostatic contribution, and $\Delta \boldsymbol{E}_{\boldsymbol{Disp}}$ represents attractive interactions associated with dispersion. ALMO-EDA2 was employed at the M06-2X(D3) / def2-TZVP level of theory in Q-Chem 5.4 using the ^{3}**TS**$_A$ geometry optimized at the same level of theory.

3.3 – Decomposition of the Δ*E*$_{CT}$ term via Complementary Occupied-Virtual orbital Pairs (COVPs)

ALMO-EDA2 analysis of the ^{3}**TS**$_A$ transition state revealed that the most dominant contribution to the total interaction energy, $\Delta \boldsymbol{E}^{\ddagger}_{\boldsymbol{int}}$, arose from the Pauli repulsion term (**Δ*E***$_{Pauli}$ = 36.6 kcal/mol). The second greatest contribution to $\Delta \boldsymbol{E}^{\ddagger}_{\boldsymbol{int}}$ resulted from the charge transfer term (**Δ*E***$_{CT}$ = -18.6 kcal/mol). To gain insight into the dominant donor/acceptor orbital interactions giving rise to **Δ*E***$_{CT}$, a Charge Decomposition Analysis (CDA) was performed using the Complementary Occupied-Virtual orbital Pairs (COVP) method[43] in tandem with the ALMO-EDA2 method at the M06-2X(D3) / def2-TZVP level of theory. This analysis revealed two major COVP contributions, with minor contributions from all other COVPs.

3.4 - NBO-derived SOPT analysis of ^{3}TS$_A$

Evaluation of the intramolecular interactions between the **CH_4** and ^{3}**SO_2** fragments was performed at the ^{3}**TS**$_A$ transition state geometry using NBO and second-order perturbative theory (SOPT) analyses (M06-2X(D3) / def2-TZVP) to identify the principal donor/acceptor NBO interactions. Two higher energy donor/acceptor pairs were identified. The first donor/acceptor pair, depicted below as **NBO-1**, corresponds with an interaction between a C–H σ-bonding NBO and an "LV" type NBO (signifying a "lone vacancy" type orbital) on the nearby oxygen atom (46.3 kcal/mol). The second highest

Go back to table of contents

energy donor/acceptor pair corresponds to a donation from the same oxygen's LP (identified as LP (3) – corresponding to the $^{3}SO_2$ SOMO-1) NBO to the C–H σ antibonding NBO of methane. A summary of the major donor/acceptor NBOs revealed through SOPT analysis of the Fock matrix in the NBO basis is provided **in Scheme S1.**

Scheme S1. Summary of principal donor/acceptor NBOs in $^{3}TS_A$ via SOPT

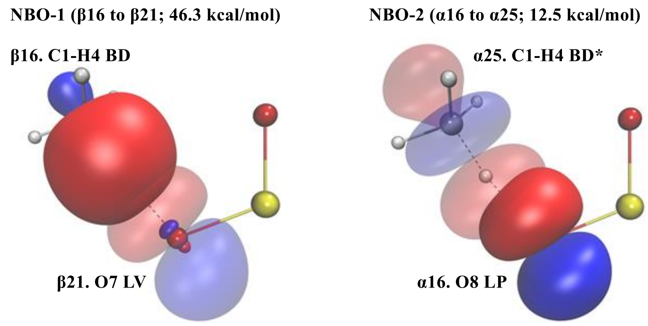

Entry	*Donor NBO*	*Acceptor NBO*	*ΔE (kcal/mol)*
**1**	**β16. BD (1) C1 - H4**	**β21. LV (1) O8**	**46.27**
**2**	**α16. LP (3) O8**	**α25. BD* (1) C1 - H4**	**12.45**
3	α15. LP (2) O8	α25. BD* (1) C1 - H4	4.83
4	β12. LP (1) O8	β24. BD* (1) C1 - H4	4.96
5	β11. LP (2) O7	β22. BD* (1) C1 - H2	0.28
6	α13. LP (3) O7	α23. BD* (1) C1 - H2	0.21
7	α11. LP (1) O7	α23. BD* (1) C1 - H2	0.12
8	β10. LP (1) O7	β22. BD* (1) C1 - H2	0.10

NBO images were rendered in VMD with an isosurface value of ±0.03. The opaque orbitals correspond to donor NBOs whereas translucent orbitals correspond to acceptor NBOs.

Go back to table of contents

3.5 – ³TS$_A$ NCI Plot and analysis of other interactions

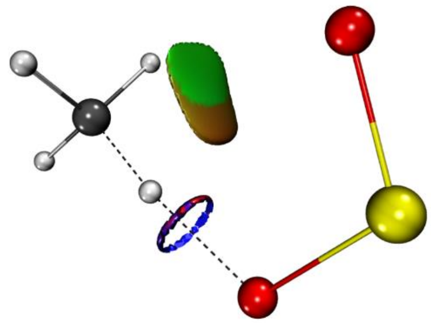

The noncovalent interactions of the **³TS$_A$** transition state were calculated at the M06-2X(D3) / def2-TZVP level of theory and subsequently plotted using Multiwfn and VMD. The NCI plot reveals, in addition to the strong interaction corresponding to the forming O–H bond, an additional favorable interaction between the second SO_2 oxygen atom (O7) and the methane moiety. This secondary stabilization can be rationalized by referring to the donor/acceptor NBO interactions described in entries **5** and **6** of **Scheme S1** which describe favorable interactions between the lone pair of **O7** and antibonding **C1-H2** NBOs. NBO plots for entries **5** and **6** of **Scheme S2**, below, show the favorable NBO donor/acceptor interactions corresponding to the stabilization revealed in the NCI plot.

Scheme S2. NBOs corresponding to LP / C-H σ* stabilization

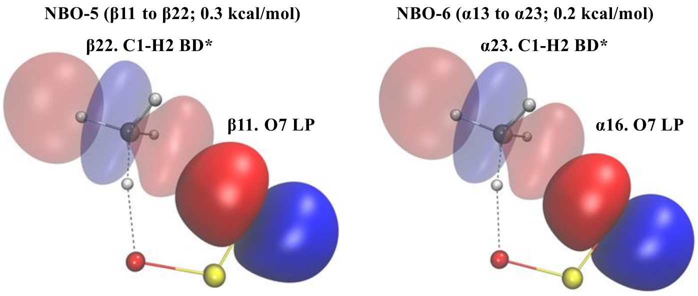

Go back to table of contents

4. Calculation of the reduction potentials of the sulfur dioxide species involved in the deactivation process

The reduction potentials of the O/R ^{3}**SO$_2$**/**SO$_2^{-\bullet}$** and **SO$_2$**/**SO$_2^{-\bullet}$** couples were calculated by taking the absolute difference in free energies of the optimized structures calculated at the ωB97XD / def2-QZVPP / PCM (MeCN)//M06-2X(D3) / def2-TZVP / PCM (MeCN) level of theory, converting the free energy value to units of eV, and subtracting the value of the absolute potential of the saturated calomel electrode (SCE) in MeCN.[44]

$$E^{\ominus}_{O/_{R},MeCN} = E^{\ominus}_{O/_{R},MeCN} - 4.43\ \mathrm{V}$$

5. Calculation of Boltzmann average Δ*G* and Δ*G*^≠ for the HAT steps in Figure 5

To improve the accuracy of the DFT computational analysis of the regioselectivity of the C–H sulfination of ketone **32** in DCM and HFIP, the ΔG and $\Delta G^{\neq}$ for the HAT steps in Figure 5 were calculated as Boltzmann averages of the conformers found for each compound. The Boltzmann average *G* values were calculated following equations (1), (2), and (3):

$$\boldsymbol{G_{av}} = \sum_i (\boldsymbol{G_i} * \boldsymbol{p_i}) \qquad (1)$$

$$\boldsymbol{p_i} = \frac{e^{\left(-\frac{\Delta G}{RT}\right)}}{\sum_i \left[e^{\left(-\frac{\Delta G}{RT}\right)}\right]} \qquad (2)$$

$$\Delta \boldsymbol{G} = \boldsymbol{G_i} - \boldsymbol{G_o} \qquad (3)$$

where $\boldsymbol{G_i}$ is the free energy of conformer $\boldsymbol{i}$, $\boldsymbol{G_o}$ is the free energy of the lowest conformer, and $\boldsymbol{p_i}$ is the probability for conformer $\boldsymbol{i}$ at 298.15 K.

The Gibbs free energy and Boltzmann average values are given below in kcal/mol. The Boltzmann averaged $\Delta\Delta G^{\neq}$ ($\Delta G^{\neq}_{33}$ – $\Delta G^{\neq}_{34}$) are in agreement with the experimentally derived values for products **33** and **34** in DCM (–1.3 kcal/mol) and HFIP (0.3 kcal/mol).

$^{3}TS_{B}$	G(kcal/mol)
a	-539396.3359
b	-539396.5551
c	-539396.5085
d	-539396.3845
e	-539396.1699
f	-539396.0755
g	-539395.8721
***G**av*	-539396.3501

S1	G(kcal/mol)
a	-195163.2175
b	-195163.1319
c	-195163.0701
d	-195162.8402
e	-195162.8258
f	-195162.8042
g	-195161.261
h	-195160.8733
***G**av*	-195163.0023

S6	G(kcal/mol)
a	-690867.0882
b	-690867.0695
c	-690867.0641
d	-690866.9479
e	-690865.6221
f	-690865.5923
g	-690865.1013
h	-690864.2254
***G**av*	-690866.964

$^{3}TS_{C}$	G(kcal/mol)
a	-859.5855945
b	-859.5830385
c	-859.5828361
***G**av*	-539397.5181

$^{3}TS_{E}$	G(kcal/mol)
a	-1035099.600
b	-1035099.292
c	-1035099.202
d	-1035099.173
e	-1035098.927
f	-1035098.642
g	-1035098.505
h	-1035098.437
i	-1035098.187
j	-1035097.591
k	-1035097.245
l	-1035097.213
***G**av*	-1035099.146

$^{3}TS_{D}$	G(kcal/mol)
a	-1035099.978
b	-1035099.936
c	-1035099.801
d	-1035099.639
e	-1035099.567
f	-1035099.512
g	-1035099.323
h	-1035099.124
i	-1035098.928
j	-1035098.804
k	-1035098.537
l	-1035098.535
m	-1035098.111
n	-1035097.758
***G**av*	-1035099.616

Go back to table of contents

6. DFT-Optimized Geometries

6.1 – ωB97XD / def2-QZVPP / PCM (DCM) // M06-2X (D3) / def2-TZVP / PCM (DCM)

S1-a

E(RM062X) = -311.068414670

E(RωB97XD) = -311.148747020

Charge = 0 Multiplicity = 1

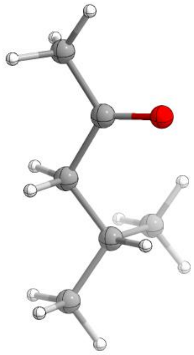

H	-3.417876322400	-0.208812416200	-0.229267577600
C	-2.498949087600	-0.689225527200	-0.554916174600
H	-2.507754194300	-1.748304549700	-0.294336984400
H	-2.421097360300	-0.626363881900	-1.642868248300
C	1.251317923600	0.110729739400	0.222126852900
H	1.085308030300	0.608287549800	1.180923377500
C	-1.302184899300	-0.005559170000	0.051765827200
O	-1.399284886400	1.055957974000	0.623849931300
C	0.018256054200	-0.730851636500	-0.081714390600
H	0.079768975000	-1.164550957900	-1.086018279900
H	-0.044368829300	-1.586749630500	0.601743699100
C	2.483337966500	-0.781239365500	0.337228269400
H	3.372425505600	-0.189272878900	0.559745441800
H	2.363878826900	-1.525752765000	1.126213560600
C	1.457870087700	1.179718775300	-0.847285865600
H	0.587517856000	1.830469971400	-0.932941349700
H	2.324331854200	1.800190239300	-0.613393725100
H	1.634656738700	0.711019958900	-1.819413550700
H	2.660841688400	-1.311322868300	-0.602363064700

Go back to table of contents

S1-b

E(RM062X) = -311.068417279

E(RωB97XD) = -311.148753609

Charge = 0 Multiplicity = 1

H	-2.8643967015	1.8293492133	-1.1513297811
C	-1.8565068807	1.8396750312	-0.7446955392
H	-1.2698800767	2.6412464756	-1.1951070263
H	-1.8993993136	2.036969753	0.329095178
C	0.9026886247	-0.9299827307	-0.5944752567
H	0.556656146	-1.5141716398	-1.450865966
C	-1.1864894858	0.5084290021	-0.9598253808
O	-1.8081161491	-0.4644954529	-1.3210753951
C	0.3052802555	0.4664414676	-0.7145305743
H	0.5310849993	1.0649480663	0.1750209977
H	0.7574558502	1.0101033592	-1.5534113432
C	2.425810684	-0.8563281031	-0.6323162712
H	2.8651939534	-1.8518285132	-0.5528487389
H	2.7797944732	-0.4009979095	-1.5588238873
C	0.4282643135	-1.6177978435	0.6825369164
H	0.7632293726	-1.0570022072	1.5596214458
H	-0.658854662	-1.6902058586	0.7155462905
H	0.8380465789	-2.6264145997	0.7546897112
H	2.800638018	-0.2576375101	0.2021946206

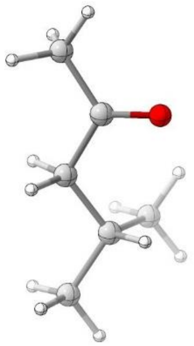

S1-c

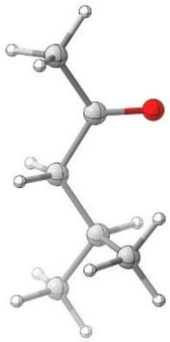

E(RM062X) = -311.068426387

E(RωB97XD) = -311.148747123

Charge = 0 Multiplicity = 1

H	-2.738950664	-0.5007637446	-1.3151247224

Go back to table of contents

C	-2.4937471115	-0.8477028226	-0.3106521764
H	-3.3428662492	-0.7193581915	0.3555416636
H	-2.2492530261	-1.9098129078	-0.3878886273
C	1.1859515292	0.4631217935	-0.0909521084
H	0.9812934043	1.3302733842	0.5418950861
C	-1.2895557516	-0.1172785114	0.2222013835
O	-1.2628301543	0.327898853	1.3467700588
C	-0.1285145557	0.0395953038	-0.7342577559
H	-0.4540649002	0.7808126531	-1.474738661
H	-0.0061376521	-0.8949687529	-1.2929870471
C	1.7473914551	-0.6525654865	0.7859279445
H	1.0419568932	-0.9390799543	1.5660028084
H	1.9668763809	-1.5350912946	0.1785072084
C	2.1935474786	0.8635594745	-1.1637687353
H	1.8197417711	1.6876216157	-1.7739127997
H	3.1375609162	1.1744428545	-0.7136518671
H	2.4011766444	0.0198794532	-1.8274251195
H	2.6754893054	-0.3386237841	1.266070592

S1-d

E(RM062X) = -311.067895079

E(RωB97XD) = -311.148682715

Charge = 0 Multiplicity = 1

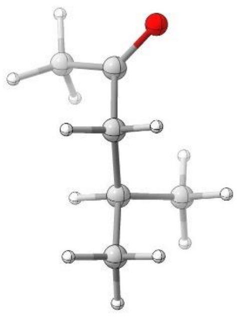

H	0.0434008225	-0.143549593	0.0002672932
C	1.1277496944	-0.2164247243	0.0059137272
H	1.5757931397	0.7352646195	0.293164275
H	1.4304089007	-0.9547461134	0.7527554515
C	3.9883860308	-1.2226295273	-0.5979313791
H	3.7877628166	-0.7977571069	0.3902644414

Go back to table of contents

C	1.6230034224	-0.6582821711	-1.3474191975
O	0.8684780909	-1.125471283	-2.1710450275
C	3.0993256855	-0.5047943353	-1.6230556852
H	3.3297031072	0.5655746012	-1.612024768
H	3.2992739368	-0.8878139488	-2.6258234959
C	3.6767682191	-2.7158035431	-0.5627993927
H	4.3116497586	-3.2276801749	0.1614853865
H	2.6363434559	-2.9114497758	-0.2941677556
C	5.4573585561	-0.9829190422	-0.9287372152
H	5.6954239184	-1.3906395301	-1.9142458737
H	5.6933098094	0.0822846082	-0.9372422473
H	6.1040325854	-1.4706575964	-0.1979611967
H	3.8557280494	-3.1609053632	-1.5448973405

S1-e

E(RM062X) = -311.067891266

E(RωB97XD) = -311.148683711

Charge = 0 Multiplicity = 1

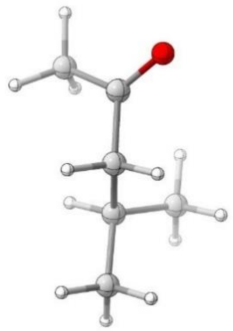

H	-1.3875419913	1.7227966821	-0.451943951
C	-1.9504454502	0.7876652419	-0.5076976128
H	-2.9552435594	0.9570688622	-0.1296679354
H	-1.9786800465	0.4903048432	-1.5563594533
C	1.0822364268	0.2000259985	-0.5774396189
H	0.6683235492	0.8795254294	-1.3285755615
C	-1.2518836994	-0.2537568987	0.3286669322
O	-1.6855034647	-0.5872009141	1.4087771863
C	0.0158527206	-0.8496837942	-0.2343096669
H	-0.2516769284	-1.4011244083	-1.1416312872
H	0.4101186977	-1.5605283285	0.4947215298

Go back to table of contents

C	1.462705741	1.0090289477	0.6589289667
H	1.8685098078	0.3496085556	1.4303519074
H	2.2219246082	1.753755324	0.4167319672
C	2.3067870997	-0.4818690193	-1.1776098717
H	2.7466322005	-1.1752735983	-0.4565782688
H	2.0465941206	-1.0462041169	-2.074384585
H	3.067720589	0.2528053959	-1.444781411
H	0.6044962206	1.531815819	1.0867840399

S1-f

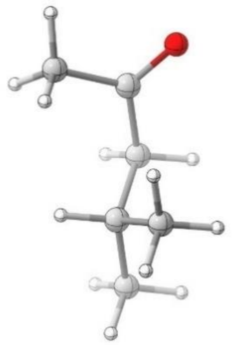

E(RM062X) = -311.067890040

E(RωB97XD) = -311.148689384

Charge = 0 Multiplicity = 1

H	-1.7450497711	-1.6008629856	1.0395700561
C	-1.8839606828	-0.524781273	0.9310831334
H	-2.9367906342	-0.2648508227	1.0027858674
H	-1.3427922399	-0.0450419325	1.7504558011
C	1.1265539764	-0.3463031175	0.2513667545
H	0.8331581992	-0.8452106596	1.1799267644
C	-1.3200879803	-0.0384657929	-0.3795143474
O	-1.9193623787	0.7609552981	-1.063462074
C	0.0264402919	-0.5820967846	-0.79290955
H	0.3038916604	-0.1174482128	-1.7412254709
H	-0.0873702879	-1.6584558313	-0.9585741971
C	2.4358282845	-0.9643755836	-0.2268363076
H	2.3262336182	-2.0346814206	-0.4079495841
H	2.7591014526	-0.4942704277	-1.1588634226
C	1.2995694418	1.1440152232	0.5285394133
H	2.0819594189	1.3125505434	1.2695594575

Go back to table of contents

H	1.582804587	1.6671111419	-0.3884147805
H	0.3804276266	1.60134202	0.9011975115
H	3.2249376451	-0.8217848869	0.5128200804

S1-g

E(RM062X) = -311.065694264

E(RωB97XD) = -311.145800992

Charge = 0   Multiplicity = 1

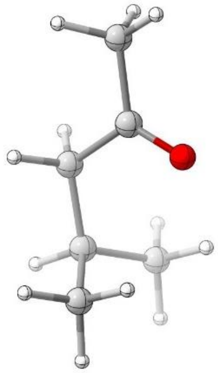

H	0.1196548881	0.3379216503	-0.0862290667
C	1.1634871745	0.0310583323	-0.0157851051
H	1.7663664635	0.9413631674	0.0214629252
H	1.3296577307	-0.5475734596	0.8891581145
C	1.6631306565	-0.8603897715	-3.829240339
H	1.2561235373	-0.2722327223	-4.6577168163
C	1.571747656	-0.7675537992	-1.2289152059
O	2.2146766613	-1.7866073848	-1.1208576203
C	1.129524209	-0.1978672761	-2.559340204
H	1.3864374819	0.8669929581	-2.5481978922
H	0.0334543112	-0.2254614712	-2.5506288618
C	3.1857738577	-0.7856911991	-3.9214287578
H	3.5261624969	-1.1560484007	-4.889845223
H	3.5361534445	0.2429381522	-3.8133116655
C	1.1599197312	-2.2925835264	-3.9980410516
H	1.5671741236	-2.9405951199	-3.2231120899
H	0.0700875899	-2.3344288975	-3.9436621948
H	1.4626645402	-2.6876822611	-4.9693271621
H	3.6514034459	-1.3904589708	-3.1435817837

Go back to table of contents

S1-h

E(RM062X) = -311.065904099

E(RωB97XD) = -311.146315150

Charge = 0 Multiplicity = 1

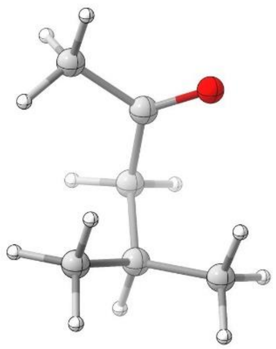

H	-2.906225195	-1.104703366	-0.4471290344
C	-2.1828274183	-1.8643863607	-0.1478796329
H	-2.0246702251	-1.7445936227	0.9270434381
H	-2.5658082656	-2.8607513873	-0.3529183644
C	-0.5084532581	0.4954741524	-2.1704563512
H	-0.138308241	1.510883493	-2.0052562329
C	-0.8687517216	-1.6461052388	-0.8483492541
O	-0.2865747044	-2.5544673517	-1.3990784612
C	-0.2979845225	-0.2451068169	-0.8322881686
H	0.7723809874	-0.3229891329	-0.6334952303
H	-0.7619492357	0.3291792654	-0.0282977504
C	0.3018609636	-0.123227262	-3.3046833241
H	1.3620275259	-0.1739469739	-3.0518552581
H	-0.039377661	-1.1363258115	-3.522143647
C	-1.984706645	0.5876398344	-2.5469831311
H	-2.5794724225	1.0220525554	-1.7410242414
H	-2.1128532217	1.2087585648	-3.4343574977
H	-2.3923080719	-0.4005849842	-2.7777803446
H	0.1943765693	0.4737544645	-4.211864829

$^3SO_2$

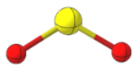

E(UM062X) = -548.515729039

E(UωB97XD) = -548.568257334

Charge = 0 Multiplicity = 3

S	-1.7776107479	0.5025751827	0.1507253422

Go back to table of contents

O	-3.2523449425	0.4294325772	-0.0064312776
O	-1.0678686296	0.8367206301	1.4114264054

^{3}TS$_{B}$-a

E(UM062X) = -859.583389402

E(UωB97XD) = -859.714244089

Charge = 0 Multiplicity = 3

C	0.0539761616	-1.3079862153	3.7814995762
H	0.6764015311	-0.4632739303	4.0789707248
H	0.080264574	-2.0305988411	4.6006030029
C	2.5854952577	-3.1597062982	1.4554614508
H	-0.9708008077	-0.9875010712	3.6139122356
C	0.6095668632	-1.96618853	2.5479990106
O	-0.096061272	-2.2843280662	1.6191569659
C	2.1062240919	-2.1941321707	2.5323723594
H	2.4203341927	-2.5313230102	3.5262080705
H	2.5567664375	-1.2024878371	2.3992486582
C	4.0858296775	-3.0644660062	1.2689949198
H	4.470043455	-3.7608092439	0.5225569327
H	4.4319348943	-2.0533036771	1.0510949774
O	5.0483608524	-3.3405089926	3.7958057286
O	5.7700717004	-1.7310704747	5.4256526344
S	5.4736757378	-1.792749824	3.9850692572
H	4.5910934838	-3.3853687868	2.2351037073
H	2.1197991194	-2.8583644981	0.5106735135
C	2.1634012821	-4.5914554865	1.7703860186
H	2.6240467001	-4.9207494907	2.7056463374
H	1.08142705	-4.6672213839	1.8738655015
H	2.4788430172	-5.2733201653	0.9798274168

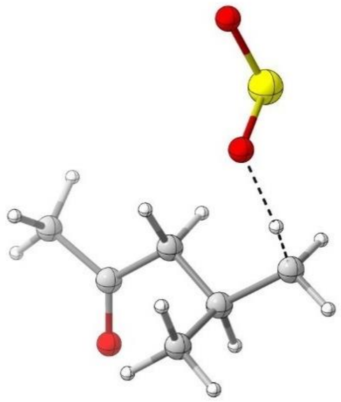

Go back to table of contents

^{3}TS$_B$-b

E(UM062X) = -859.583575678

E(UωB97XD) = -859.714424753

Charge = 0 Multiplicity = 3

C	1.9059254194	0.4807807684	2.0166409088
H	1.44512248	0.5433113092	3.0030099076
H	1.100401441	0.5393674258	1.2808256605
C	2.4630928611	-3.3928565673	1.877238282
H	1.287901577	-3.5595767316	0.0698058777
C	2.6142799791	-0.8356937388	1.8457007245
O	3.7259273891	-0.9068685325	1.373990146
C	1.8588279686	-2.0624858756	2.3071761538
H	0.8189581929	-1.9821237893	1.9707478985
H	1.8163481742	-1.9987115063	3.4006428406
C	2.3819041674	-3.5766666781	0.3739936448
H	2.8410004366	-2.7655301067	-0.1903792403
H	2.7850335717	-4.5352481998	0.0455049118
O	-0.2579546806	-4.0332835217	-0.1230418558
O	-1.506912136	-6.054149954	-0.4648969809
S	-0.1027239788	-5.6256057177	-0.3554231043
H	2.5959410221	1.3064050662	1.8641890773
C	1.7937859678	-4.547286918	2.6155634048
H	0.7165155561	-4.5467085621	2.4300928744
H	2.1938122945	-5.5077604099	2.2864477241
H	1.9470622214	-4.4643571948	3.6921193931
H	3.5276655762	-3.3781622138	2.1352990048

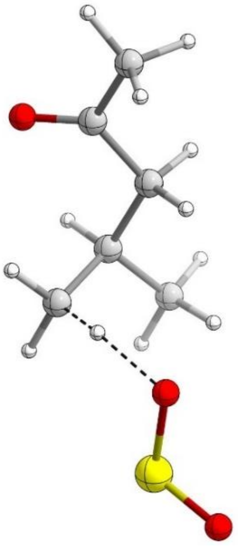

Go back to table of contents

^{3}TS$_{B}$-c

E(UM062X) = -859.583304359

E(UωB97XD) = -859.714223146

Charge = 0 Multiplicity = 3

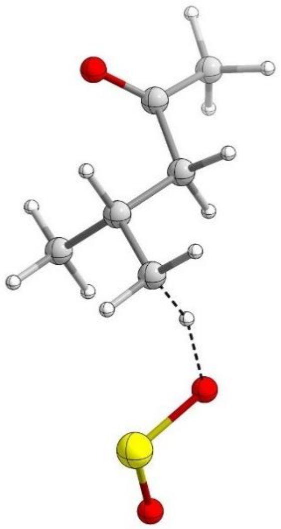

C	-0.2531643858	-0.5670682248	2.1943403268
H	0.0351230498	-0.044423325	1.2791961805
H	0.1618073716	-0.0030831418	3.030557298
C	2.4196009075	-3.4079539322	1.9733443491
H	4.4623879761	-2.6965134385	2.0412050861
C	0.3257209843	-1.9552046359	2.1546655024
O	-0.3670239868	-2.9325538087	1.9894712016
C	1.822511901	-2.0583547852	2.351286128
H	1.9998486259	-1.836909103	3.4105279094
H	2.3097736142	-1.2496124562	1.7957830871
C	3.8365140131	-3.5294657982	2.4955918069
H	3.922227938	-3.3774667151	3.5723886937
H	4.3120794001	-4.4688075083	2.2115248853
O	5.3091447896	-1.8566031962	0.9423278612
O	6.7260381754	-2.2558290591	-0.9545265675
S	6.0003650701	-3.0273760685	0.0677967098
H	-1.3369831306	-0.608628528	2.2619950496
C	2.3627255635	-3.6371278443	0.4661411194
H	2.9118695029	-2.849207927	-0.0565197229
H	2.8108305493	-4.5958384498	0.1999894672
H	1.3338361299	-3.6304414679	0.108537484
H	1.8249879867	-4.1865885802	2.4641200834

Go back to table of contents

^{3}TS$_{B}$-d

E(UM062X) = -859.583551165

E(UωB97XD) = -859.714397696

Charge = 0 Multiplicity = 3

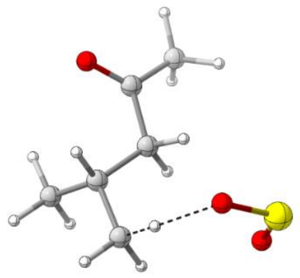

C	-0.1730106787	-0.5366424	2.1935557753
H	0.1045056265	-0.033783719	1.264071157
H	0.281896249	0.0250388479	3.01037498
C	2.4243825382	-3.448387452	1.9511173846
H	3.9834221125	-3.3856400574	3.4634220961
C	0.3642036344	-1.9420989763	2.1548328033
O	-0.3617667823	-2.8986479698	2.0097403595
C	1.8597492996	-2.0859336398	2.3286952428
H	2.0495348549	-1.872889916	3.3885747641
H	2.3637917203	-1.2852712206	1.7774632551
C	3.8826741435	-3.5551993336	2.3635081792
H	4.2962775776	-4.5469407571	2.1745593946
H	4.4992941855	-2.8029952499	1.8699451299
O	4.0245222704	-2.6771769418	5.0847646801
O	4.6683946798	-0.673938556	3.6417264917
S	4.4641346632	-1.1618003893	5.0147142832
H	-1.2553381684	-0.5452511482	2.2915103376
C	2.3019437579	-3.6970633166	0.445996777
H	2.8658949739	-2.9425441306	-0.1081038866
H	2.6993598249	-4.6775189027	0.1813839174
H	1.2599061881	-3.6535118212	0.1301980337
H	1.8470453292	-4.2122579499	2.4768488439

Go back to table of contents

^{3}TS$_B$-e

E(UM062X) = - 859.583575678

E(UωB97XD) = -859.713484271

Charge = 0 Multiplicity = 3

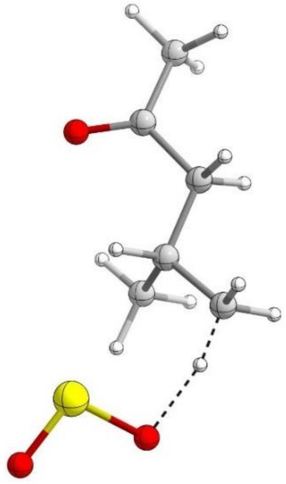

C	1.9059254194	0.4807807684	2.0166409088
H	1.44512248	0.5433113092	3.0030099076
H	1.100401441	0.5393674258	1.2808256605
C	2.4630928611	-3.3928565673	1.877238282
H	1.287901577	-3.5595767316	0.0698058777
C	2.6142799791	-0.8356937388	1.8457007245
O	3.7259273891	-0.9068685325	1.373990146
C	1.8588279686	-2.0624858756	2.3071761538
H	0.8189581929	-1.9821237893	1.9707478985
H	1.8163481742	-1.9987115063	3.4006428406
C	2.3819041674	-3.5766666781	0.3739936448
H	2.8410004366	-2.7655301067	-0.1903792403
H	2.7850335717	-4.5352481998	0.0455049118
O	-0.2579546806	-4.0332835217	-0.1230418558
O	-1.506912136	-6.054149954	-0.4648969809
S	-0.1027239788	-5.6256057177	-0.3554231043
H	2.5959410221	1.3064050662	1.8641890773
C	1.7937859678	-4.547286918	2.6155634048
H	0.7165155561	-4.5467085621	2.4300928744
H	2.1938122945	-5.5077604099	2.2864477241
H	1.9470622214	-4.4643571948	3.6921193931
H	3.5276655762	-3.3781622138	2.1352990048

Go back to table of contents

^{3}TS$_{B}$-f

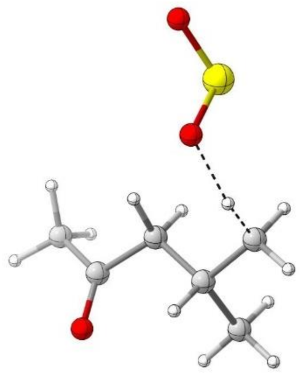

E(UM062X) = -859.582507520

E(UωB97XD) = -859.713451817

Charge = 0 Multiplicity = 3

C	-0.3007349884	-0.585625078	2.1612994151
H	0.0113789747	-0.0464271585	1.2636944841
H	0.0808111518	-0.0303445412	3.0188865065
C	2.4126740789	-3.3925427261	1.9979740111
H	3.8672771014	-3.337017974	3.6170147369
C	0.2934668579	-1.9672376557	2.1195778217
O	-0.3816615774	-2.9481722506	1.9100970666
C	1.7824543684	-2.0583518202	2.3749265201
H	1.9059510238	-1.8626447439	3.4473691332
H	2.2824266359	-1.2287698626	1.8628373591
C	3.8460373006	-3.467923148	2.4875852008
H	4.3121425787	-4.4377054586	2.3106692418
H	4.4710020047	-2.6773236711	2.0656023018
O	3.9525244049	-2.5756720201	5.0322288429
O	4.5975482429	-0.440725038	5.9293567289
S	4.6701465629	-1.1770478876	4.65693611
H	-1.3857015075	-0.6388758725	2.1926166912
C	2.3902718568	-3.607720634	0.4820124486
H	2.9594778342	-2.8218794785	-0.0203861666
H	2.8333711831	-4.5685452443	0.2188576743
H	1.36672545	-3.5890980337	0.1089174358
H	1.8329124617	-4.188587703	2.4695634362

Go back to table of contents

$^{3}TS_{B}$-g

E(UM062X) = -859.582600458

E(UωB97XD) = -859.713451817

Charge = 0 Multiplicity = 3

C	0.2634737363	-0.7376355103	3.6028363636
H	0.9552642306	0.0727974836	3.8346910439
H	0.1126521875	-1.3062275649	4.5235310407
C	2.7615117812	-3.2266165548	1.9188966155
H	-0.6892318432	-0.3412803458	3.2618097957
C	0.8513249697	-1.6567076321	2.5659355574
O	0.2081519349	-2.0517747005	1.6224400952
C	2.302090927	-2.0439245647	2.7701921234
H	2.4699975798	-2.2399567754	3.8342044433
H	2.8855205558	-1.1451426966	2.5381359859
O	5.0615585553	-5.6834017469	0.9467677187
O	5.6206498291	-7.7732969795	1.9930876867
S	5.3003295286	-6.3893758251	2.3798910735
C	4.2739238845	-3.3154105745	1.9802088526
H	4.6389456408	-3.5040738914	2.9925655794
H	4.6173003914	-4.1918346747	1.3423057492
H	4.7843036535	-2.4482512856	1.5595110361
C	2.1139207202	-4.5257970624	2.3870889659
H	2.3962390052	-5.3556994892	1.7373532106
H	2.4367045386	-4.7665910294	3.4039571062
H	1.0273565732	-4.4469903084	2.3799121073
H	2.4577516201	-3.0312422714	0.8885008491

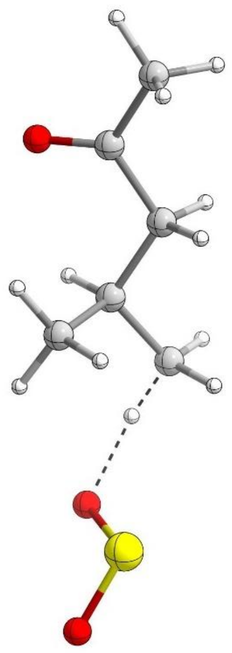

Go back to table of contents

^{3}TS$_{C}$-a

E(UM062X) = -859.587174533

E(UωB97XD) = -859.716968504

Charge = 0   Multiplicity = 3

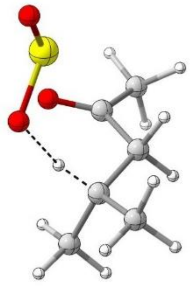

C	-0.2219128604	-0.5374743724	2.08675713
H	0.1489653338	0.0265819762	1.2275358343
H	0.1114361376	-0.0110888257	2.9823476854
C	2.5171668233	-3.2781966248	1.8011087065
H	-1.3068498229	-0.5855300069	2.0521517347
C	0.3707892369	-1.916406198	2.0428710178
O	-0.2961859365	-2.8962252154	1.7967838409
C	1.8488804591	-2.028843246	2.3525669744
H	1.9274435085	-1.9958412396	3.4467132161
H	2.3648657697	-1.1335993063	1.9907632132
O	1.3733486497	-5.5137962037	3.0124132951
O	0.348403593	-5.8154405799	5.1593072746
S	0.3885313236	-4.8081454054	4.0818771391
H	1.8611370688	-4.1504455218	2.0670895234
C	2.592810929	-3.2613253727	0.2812790766
H	3.2331184913	-2.4356725445	-0.0428791852
H	3.0220955218	-4.189178192	-0.0968584366
H	1.6080316474	-3.1295175008	-0.1668764404
C	3.8697225858	-3.5241533959	2.4461891544
H	4.3204444358	-4.441016238	2.0655133235
H	4.5475059107	-2.6954838836	2.2236479302
H	3.781412194	-3.6077361029	3.5305789919

Go back to table of contents

^{3}TS$_{C}$-b

E(UM062X) = -859.586959341

E(UωB97XD) = -859.715474466

Charge = 0 Multiplicity = 3

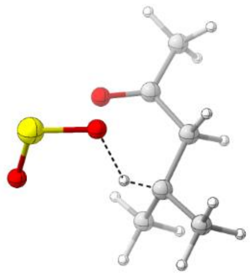

C	-0.0814871975	-0.9768197543	1.8782301872
H	0.2284809141	-0.2186184342	1.1551830878
H	0.1815125133	-0.6040429716	2.8687280643
C	2.9389303093	-3.4129986744	1.3611559736
H	-1.154247962	-1.1326254872	1.8009003655
C	0.6581768198	-2.2530870871	1.5783476534
O	0.1036075867	-3.2089482486	1.0870664024
C	2.1276091128	-2.2674582297	1.94195623
H	2.1631181333	-2.2883969089	3.0377838586
H	2.5648336127	-1.3051043792	1.6523960516
O	1.358451102	-5.1157178491	2.9525236086
O	0.9868235848	-6.2877739894	0.7354119278
S	0.4686210161	-6.1278067744	2.1019229329
H	2.3965975517	-4.3546731838	1.5972639582
C	3.0436947495	-3.3323257343	-0.1558972769
H	3.5825133752	-2.422825925	-0.4385620571
H	3.5935421113	-4.1866358153	-0.5516805842
H	2.0594130357	-3.3110129273	-0.6217611076
C	4.3072699231	-3.5016236847	2.0210903932
H	4.8801665108	-4.3374843307	1.6183715091
H	4.8735292743	-2.5845856692	1.835873689
H	4.2186481901	-3.6319269823	3.1007129628

Go back to table of contents

³TSC-c

E(UM062X) = -859.587179437

E(UωB97XD) = -859.714223146

Charge = 0 Multiplicity = 3

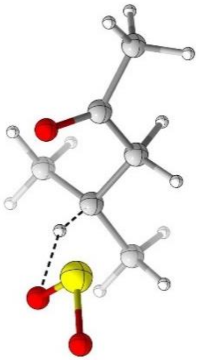

C	-0.2213877587	-0.5643552705	2.1366544561
H	0.0830010881	-0.033413306	1.2314395461
H	0.1499588254	0.0119022282	2.9853176123
C	2.564616212	-3.2568907546	1.8252029847
H	-1.3048466025	-0.6396355158	2.169509777
C	0.4034651875	-1.9297020815	2.1210365644
O	-0.25147247	-2.9350858773	1.9606278841
C	1.8995940249	-1.9931167371	2.3467230639
H	2.042306746	-1.8972522291	3.4306357945
H	2.3708097464	-1.1085770669	1.9058955539
O	1.5438889351	-5.4506515326	3.2085192229
O	0.6485116347	-5.6628779592	5.422813004
S	0.5949534447	-4.7171104156	4.2915781493
H	1.9430242263	-4.1257997708	2.172449537
C	2.5575750556	-3.3202307805	0.3048006914
H	3.1652290916	-2.5040628973	-0.0969535686
H	2.9816832433	-4.2606891309	-0.0471435481
H	1.5478686096	-3.2264574789	-0.0948129445
C	3.9547421652	-3.4426384261	2.4072258291
H	4.405584692	-4.3681936055	2.0483603645
H	4.6004168415	-2.6125637158	2.107742153
H	3.9256060615	-3.4732486763	3.4975818728

Go back to table of contents

S2

E(UM062X) = -310.398001383

E(UωB97XD) = -310.473376626

Charge = 0   Multiplicity = 2

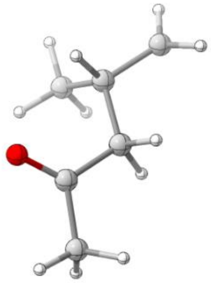

H	-1.3502748498	-0.4005686287	0.5379881202
C	-0.3280053746	-0.3926470387	0.9069291141
H	0.2490221458	0.395188064	0.4213956294
H	-0.329367476	-0.1772037457	1.9781298616
C	2.4024604276	-3.1894758675	1.0109377796
C	0.3209492146	-1.7336716291	0.6912614021
O	-0.3213482444	-2.7091070359	0.3770292853
C	1.8216630015	-1.7832271185	0.876064287
H	2.0938417468	-1.166405226	1.7386165658
H	2.2459538269	-1.2728946458	0.0030897124
C	3.8846348643	-3.1599607654	0.8784417362
H	4.4885107933	-3.9262868061	1.3446413484
H	4.3608287801	-2.5019215039	0.1640889949
C	1.9771057642	-3.8403197294	2.3272875477
H	2.3552062132	-3.2603982902	3.1726500957
H	0.8916653555	-3.8989439419	2.4023354957
H	2.379733516	-4.8510198843	2.4045714543
H	1.9789792999	-3.7871544987	0.190733656

S4

E(UM062X) = -310.407161237

E(UωB97XD) = -310.484638056

Charge = 0   Multiplicity = 2

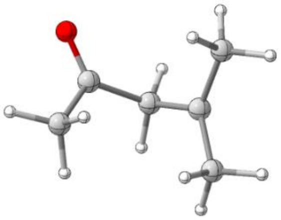

H	-1.97532302	-0.6339739954	3.0743317027
C	-1.0032933115	-0.4830769601	3.5371382953

Go back to table of contents

H	-0.7654517975	0.5841347577	3.5121202027
H	-1.0204938755	-0.788827874	4.5829635839
C	2.0303603043	-0.1629374475	3.8309943409
C	0.0584598068	-1.2245157727	2.7658288571
O	-0.1079701374	-1.5542912436	1.6141754503
C	1.3806066123	-1.4662265853	3.4824245222
H	2.0076247312	-2.0527979231	2.8076292402
H	1.1820169452	-2.0473079926	4.3866461784
C	2.5022395718	0.7022004542	2.7125362275
H	3.3099258949	1.361311458	3.037827197
H	2.8528570108	0.109490485	1.8658850966
C	1.8268785125	0.4537051344	5.1714401235
H	0.9684329353	1.1411545892	5.1825099876
H	1.6485207066	-0.2987867288	5.9407255822
H	2.6968600885	1.0485825448	5.4613820333
H	1.6991147947	1.354065541	2.3371556788

SO_2H

E(UM062X) = -549.208293933

E(UωB97XD) = -549.260754474

Charge = 0 Multiplicity = 2

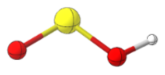

S	-1.9066686725	1.09953378	-0.0208331338
O	-3.0009090055	0.217651626	-0.4247251459
O	-2.5326288925	2.0006684899	1.1761100303
H	-1.8639785694	2.6204351441	1.5063950595

S3

E(RM062X) = -859.710203072

E(RωB97XD) = -859.830949804

Charge = 0 Multiplicity = 1

H	3.387136489	-1.2199564639	-0.5574447888
C	2.4682764405	-1.7103817983	-0.2366743277
H	1.9559872341	-2.0754059821	-1.1309766133
H	2.6828004779	-2.5461775151	0.4237114157
C	0.3023460685	1.5195253632	0.26001116
H	0.102757701	1.3096133893	1.3136477393
C	1.5378313522	-0.7403954919	0.4212767935
O	0.9108395334	-1.0424743385	1.4191801781
C	1.4582899526	0.6320344496	-0.198585691
H	1.481605448	0.5268102394	-1.2860223553
H	2.4096631125	1.1059706786	0.0745309262
C	0.6938792013	2.9935838843	0.1395068654
H	0.9445946953	3.2407245994	-0.8944543068
H	-0.1215958287	3.6438528942	0.4567740752
C	-0.9863892489	1.3083414248	-0.5346585125
H	-1.7776465708	1.9580233628	-0.1545846181
H	-0.8371810965	1.5419252319	-1.5925634964
H	1.564169437	3.2079515582	0.7605131113
S	-1.7181056174	-0.3418909974	-0.5546775852
O	-1.7371510629	-0.6393334721	1.0269512781
H	-0.8119805017	-0.7005583183	1.3665778098
O	-0.7102590877	-1.2182490951	-1.1891878154

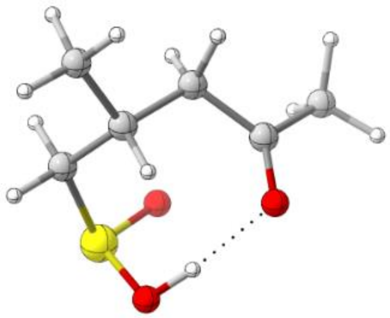

Go back to table of contents

S5

E(RM062X) = -859.704803609

E(RωB97XD) = -859.824365703

Charge = 0 Multiplicity = 1

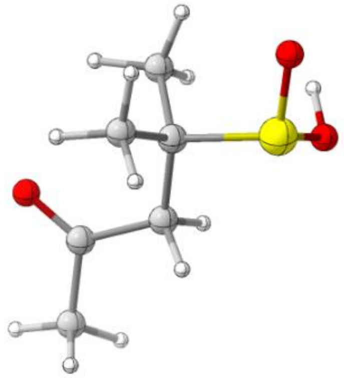

H	-1.1689508085	-0.0240377777	5.2900024609
C	-0.5447112236	-0.0933010963	4.4033408266
H	-1.1780933338	-0.1133356469	3.5134923065
H	0.1009502043	0.7813463335	4.3207553636
C	2.0890230061	-2.7836351315	3.2784376812
C	0.271839973	-1.3576176928	4.4412848187
O	0.0518887259	-2.2349377225	5.2415834799
C	1.3799247521	-1.4378954389	3.407687384
H	2.0981298366	-0.6535788366	3.6696107992
H	0.9502680822	-1.1335416622	2.4465501696
C	2.8750951479	-3.1844406357	4.5183001996
H	3.5678476307	-3.9935198178	4.2805174939
H	3.4296113746	-2.3425791848	4.9376044078
C	1.1419358897	-3.8941679854	2.8266001849
H	0.3748556081	-4.0416332677	3.5845315034
H	0.6530419753	-3.6397707793	1.8829470763
H	1.6889799409	-4.8270744947	2.6941774551
H	2.1842299557	-3.5342375554	5.2826605901
S	3.2546051765	-2.5885240512	1.8601403498
O	4.2528813348	-1.4699299257	2.4818009185
H	4.7445169423	-1.8418972946	3.2339070134
O	4.0020920179	-3.8531265335	1.7870220553

Go back to table of contents

6.2 - ωB97XD / def2-QZVPP / PCM (HFIP) // M06-2X (D3) / def2-TZVP / PCM (HFIP)

S6-a

E(RM062X) = -1100.98806038

E(RωB97XD) = -1101.15258574

Charge = 0 Multiplicity = 1

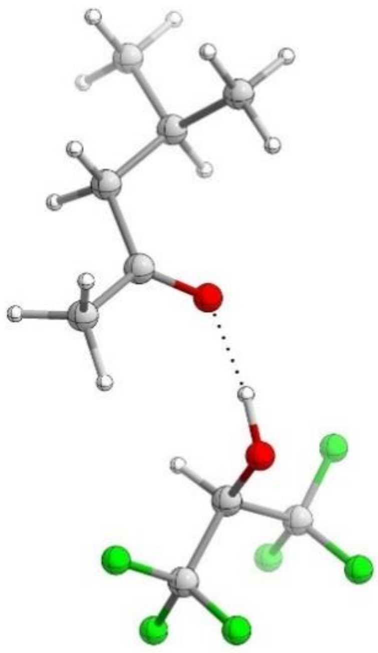

C	1.4022435024	-4.0473865469	3.9219868533
H	1.2149637062	-4.543762916	2.9696840054
H	0.4456034553	-3.6515358693	4.2719120285
C	2.340786324	-2.8933819689	3.7336710159
C	2.1024327559	-2.0078655983	2.5414852667
H	1.0222197121	-1.8756508987	2.4179740281
H	2.4223113842	-2.6006815613	1.6749482544
H	3.8727536813	-0.8531851115	2.7990615948
H	1.7869432318	-4.754088444	4.6536515952
C	2.8206503967	-0.6640896596	2.5721259736
C	2.7258952876	0.0090016883	1.2067160039
H	1.6809778515	0.1895337929	0.9412845121
H	3.2402312706	0.9709089526	1.2134487005
H	3.1703544738	-0.6098783482	0.4255526651
C	2.2429976589	0.2374360827	3.6596390636
H	2.7749476622	1.1891389568	3.6947546012
H	1.1903084595	0.4491913247	3.4536455241
H	2.3130824626	-0.225662056	4.6441059157
O	3.2635517971	-2.6856684065	4.5021067244
H	3.5827433244	-3.6966612354	5.8239572101
O	3.8745968117	-4.3994033097	6.4499990678
C	4.8025840815	-5.191446771	5.7963031046
H	4.7734246317	-5.0789894882	4.7072042016
C	4.4693433276	-6.6510566885	6.0995200267

Go back to table of contents

C	6.2157017015	-4.8002830379	6.2371701782
F	5.3573302066	-7.4901134008	5.5632006699
F	3.2727705793	-6.9489846039	5.5884012508
F	4.4176543885	-6.8939913715	7.4081123322
F	6.4079153376	-3.5034444271	5.9895764954
F	6.4120692916	-4.994772279	7.5412162138
F	7.1556457384	-5.4799150667	5.576962273

S6-b

E(RM062X) = -1100.98840856

E(RωB97XD) = -1101.15284996

Charge = 0 Multiplicity = 1

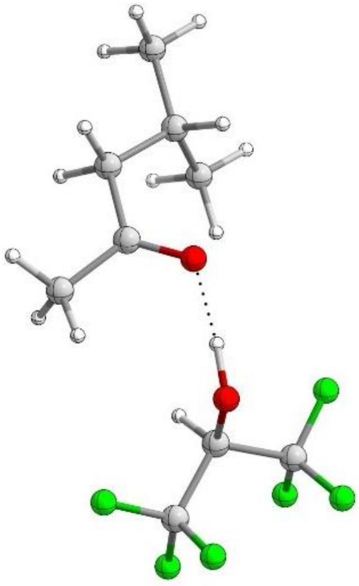

C	1.7352761216	-4.3087303622	3.5795807054
H	2.3357209222	-4.8623740038	2.8526091385
H	0.7208727639	-4.2595115744	3.1841275143
C	2.3166078156	-2.932332566	3.7088995182
C	2.0544234655	-1.9698595604	2.5839153473
H	0.9996392354	-1.6848872674	2.6877962794
H	2.1136913228	-2.5187015087	1.6378519758
H	2.9217861712	-0.2855453718	3.5555936725
H	1.7476737236	-4.832731839	4.5329073114
C	2.9493157271	-0.7364258965	2.5606348993
C	4.3923974839	-1.1150171225	2.2385013683
H	4.4507759378	-1.5673828233	1.2448499981
H	5.0324958148	-0.2317399741	2.2419859057
H	4.7929577142	-1.8248005003	2.9629221234
C	2.4180829673	0.2735788366	1.5486873787
H	3.045937633	1.1652844164	1.5266416758
H	2.413125534	-0.1579381845	0.5443253713

Go back to table of contents

H	1.3988505499	0.5800564287	1.7894208334
O	2.9691133339	-2.6037691552	4.6842359291
H	3.2897665958	-3.6792392923	5.9496945754
O	3.589675338	-4.3993956822	6.552529871
C	4.6059833891	-5.0926446476	5.9193470847
H	4.5832118965	-4.9956942753	4.8280308742
C	4.420153266	-6.5756403173	6.2336398021
C	5.9661026277	-4.5545271923	6.3733454776
F	5.4107760163	-7.3198575835	5.7385387319
F	3.2802096559	-7.0063604201	5.6897507922
F	4.3523115088	-6.8067947019	7.5434027711
F	6.0191642912	-3.2432345624	6.1313581199
F	6.1725826703	-4.7325351112	7.6780106156
F	6.9780225066	-5.1259131859	5.7174563385

S6-c

E(RM062X) = -1100.98842086

E(RωB97XD) = -1101.15280824

Charge = 0 Multiplicity = 1

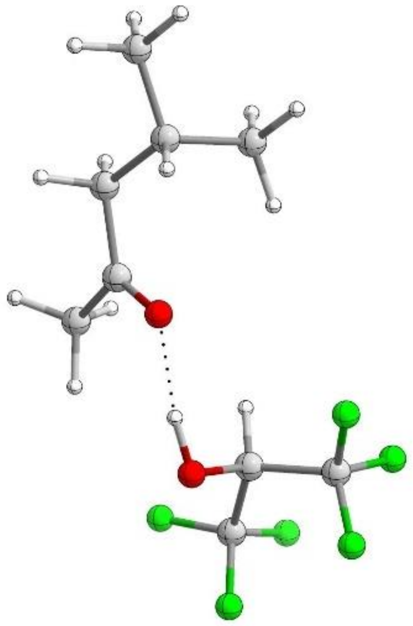

C	1.7023316565	-4.0537142824	4.1042531329
H	2.1145432848	-4.7423079889	3.3614082524
H	0.6274762146	-4.0036763179	3.9309514506
C	2.3331415244	-2.709599327	3.8915977415
C	1.8520473774	-1.9047359396	2.716316744
H	0.8611062716	-1.533564133	3.0076104353
H	1.6710131331	-2.5869990556	1.8788547924
H	2.9777102661	-0.1604511646	3.191516293
H	1.9097329309	-4.4324116784	5.1027478062
C	2.7611438765	-0.7543154288	2.3002285596

Go back to table of contents

C	4.0779003369	-1.2783793743	1.7334337064
H	3.891340652	-1.8778701827	0.838293837
H	4.73268869	-0.452460977	1.4524336195
H	4.6084456373	-1.8981602807	2.4570490388
C	2.0491710797	0.1301738903	1.2819270722
H	2.6868979955	0.9618450169	0.979523329
H	1.7990545711	-0.4437310131	0.3858662727
H	1.1236859515	0.5405408806	1.6891025247
O	3.1948757784	-2.2836351533	4.640538738
H	3.7712552317	-3.1949550669	5.9523141664
O	4.1908861323	-3.8561316819	6.5503781171
C	4.9980239006	-4.6723367098	5.7776623372
H	4.708799652	-4.6901590353	4.7207740507
C	4.8494427513	-6.0999950463	6.2995949567
C	6.4441752741	-4.1700659468	5.8234188243
F	5.6664831066	-6.949065988	5.6736469203
F	3.5987113608	-6.5194940554	6.0976123402
F	5.0953819743	-6.185321075	7.6055072588
F	6.4765427761	-2.8959052736	5.4284959548
F	6.9616870294	-4.2252228712	7.0505171895
F	7.2436445824	-4.8636787403	5.0108465376

S6-d

E(RM062X) = -1100.98805142

E(RωB97XD) = -1101.15255607

Charge = 0   Multiplicity = 1

C	1.432164019	-3.7751406021	4.4594575874
H	0.9863406603	-4.386736983	3.6749292591
H	0.6131502105	-3.273675582	4.9809551532

Go back to table of contents

C	2.3153510118	-2.7218344764	3.8609199621
C	1.788192921	-1.9920340237	2.6559673925
H	0.7180318626	-1.809832144	2.8009407752
H	1.8500508405	-2.7149530843	1.8324242237
H	3.5908674592	-0.9277274128	2.2667363084
H	1.9799441571	-4.3956294184	5.1651337186
C	2.5217662728	-0.7041766727	2.3010145951
C	2.0783212308	-0.2133514176	0.9266351331
H	1.0054650759	-0.0036915516	0.9233143706
H	2.5996436901	0.7063876497	0.6579236344
H	2.2792612282	-0.9571961954	0.1539270821
C	2.2832674341	0.368641988	3.3599870399
H	2.8264368965	1.2817844686	3.1127937064
H	1.2194667086	0.6154018533	3.4135011396
H	2.6087849216	0.0383658151	4.3466379679
O	3.4167296156	-2.4728933549	4.3192300273
H	4.0476602357	-3.2968909523	5.6525724239
O	4.4729836018	-3.9080862211	6.2985977223
C	5.1871901136	-4.8544613814	5.5848194917
H	4.8580229973	-4.9492236941	4.544203502
C	4.9541248574	-6.2102876998	6.2484949425
C	6.6665921161	-4.46152749	5.5401679018
F	5.6726849726	-7.1785891256	5.6772146627
F	3.6665606584	-6.5438770116	6.1382038274
F	5.2536946491	-6.1923977902	7.5461668901
F	6.7790863105	-3.2424944589	5.0094655971
F	7.2190431872	-4.425508775	6.7528244466
F	7.3867890839	-5.2961682558	4.7880455155

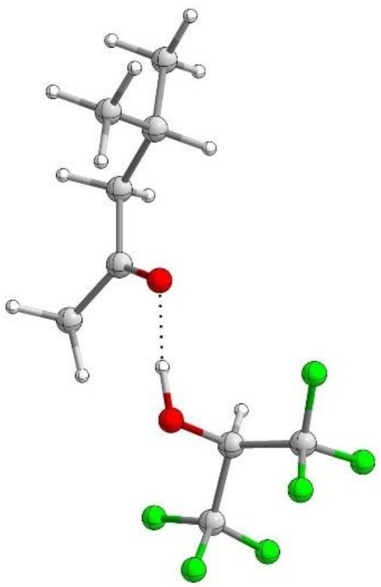

Go back to table of contents

S6-e

E(RM062X) = -1100.98790019

E(RωB97XD) = -1101.15210131

Charge = 0 Multiplicity = 1

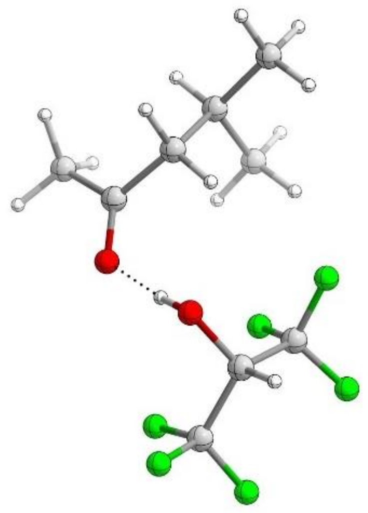

C	-0.0466026833	0.6694207142	-0.4352886753
H	1.0056097267	0.5488016321	-0.6769056703
H	-0.3758769537	1.6653872829	-0.740417439
C	-0.2584469186	0.5140449266	1.04162788
C	-1.6709457777	0.365473026	1.5416037356
H	-1.991107499	-0.6425552961	1.2534033754
H	-1.6647654391	0.4102093564	2.6332269156
H	-2.64902661	1.3124339932	-0.1183243994
H	-0.6541225307	-0.0511858409	-0.983929083
C	-2.6601995016	1.3898314715	0.9725358777
C	-4.0689380624	1.0659606568	1.4581475379
H	-4.1131776885	1.1129043821	2.5492356271
H	-4.7884942577	1.7831823329	1.0612345698
H	-4.3757743677	0.0658159446	1.1489236443
C	-2.266365035	2.8082570105	1.372827018
H	-2.9421213581	3.5374131914	0.9244397325
H	-2.3184425232	2.9194210766	2.4581175144
H	-1.2487790067	3.0581553766	1.0633899863
O	0.6936356713	0.4892920403	1.8014239904
H	0.5017736184	0.1974720835	3.4745469934
O	0.3222719533	-0.086038074	4.4048619646
C	0.5847602019	0.9272003213	5.306642616
H	0.3294433342	0.5847877363	6.3118402224
C	-0.2890841839	2.1527360645	5.0251218279
C	2.0798587317	1.2600628378	5.3189938697

Go back to table of contents

F	-0.1519930023	3.0984744637	5.9533182626
F	-1.5734603495	1.7913994581	5.0037106855
F	-0.0097698978	2.6993376272	3.8379672879
F	2.5152469284	1.6206344846	4.1097477666
F	2.3768651026	2.2426072549	6.1703102336
F	2.7726923778	0.1824954645	5.6877381317

S6-f

E(RM062X) = -1100.98817168

E(RωB97XD) = -1101.1519137

Charge = 0   Multiplicity = 1

C	-0.3456295627	1.4031107072	-0.336877206
H	0.6878551052	1.7339285911	-0.407699396
H	-0.9966763068	2.1961298037	-0.7143825475
C	-0.7177737223	1.1179120667	1.0895316978
C	-1.978380187	0.3398645894	1.3406075577
H	-1.8578713548	-0.6472497699	0.8839258247
H	-2.0945802412	0.2124503347	2.4182770683
H	-3.0924248131	1.1042845814	-0.3305835594
H	-0.5110735647	0.5242923806	-0.9597249793
C	-3.2244053236	1.0213076193	0.7521606745
C	-4.4532021446	0.1594139235	1.0183176063
H	-4.6136299679	0.0489715928	2.093473721
H	-5.3453775559	0.617912213	0.5899005938
H	-4.3395401788	-0.8365876389	0.5880308171
C	-3.4013055105	2.4197500228	1.3357155029
H	-4.2912836169	2.8990774393	0.9265477946
H	-3.5141700403	2.3640420123	2.4213904671
H	-2.5461418326	3.0650347109	1.1229117554

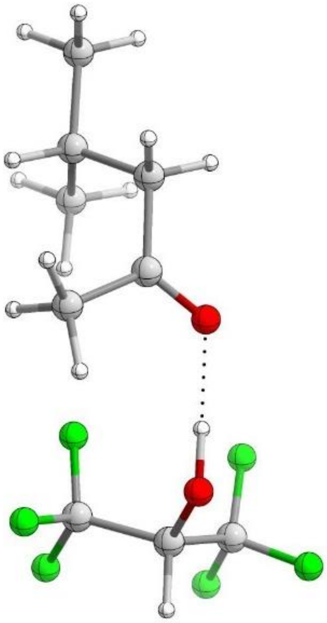

Go back to table of contents

O	-0.0363787877	1.5181346765	2.0170877818
H	1.3498674623	2.4739183242	1.747397554
O	2.1942746616	2.9437734734	1.5353537314
C	2.0521086238	4.3153264009	1.6226998756
H	3.0043796436	4.7884449892	1.3730793524
C	1.714758779	4.7336030677	3.0570020777
C	1.0305280876	4.8274610955	0.6028983047
F	1.5805090341	6.0541358617	3.1840628977
F	2.6928008006	4.346090529	3.8754954963
F	0.5856186241	4.1642800566	3.4850227369
F	-0.1951331737	4.3450677979	0.8412321144
F	0.9476600463	6.156632818	0.5884702501
F	1.3812700168	4.4307107295	-0.6203265659

S6-g

E(RM062X) = -1100.98556213

E(RωB97XD) = -1101.14980441

Charge = 0 Multiplicity = 1

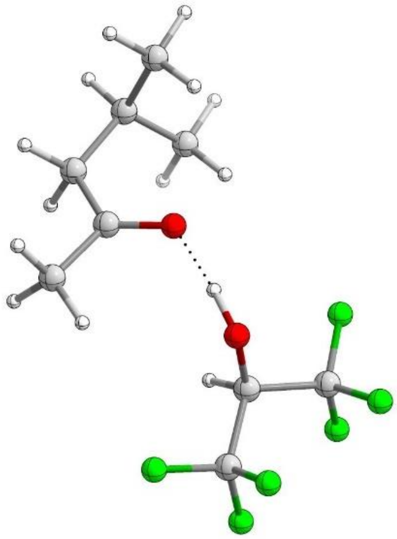

C	1.4655000378	-4.0239288162	4.263765223
H	1.2443491899	-4.6556358019	3.4037277942
H	0.5101237665	-3.6786433239	4.6656500613
C	2.2628182401	-2.8214729053	3.8453696936
C	1.7907809959	-2.105279223	2.6089275396
H	0.7111065957	-1.9614040042	2.7248956059
H	1.8920820204	-2.8283938011	1.790848376
H	1.9525193772	-0.4381017975	1.3442662874
H	1.9889122116	-4.591927032	5.0298774559
C	2.4731242974	-0.7879461381	2.2406991793
C	2.2922496498	0.2786133265	3.3187474322

Go back to table of contents

H	2.8304494592	0.0107182893	4.227801647
H	2.6761251573	1.2379579516	2.9685438917
H	1.2376778393	0.4097454105	3.569353869
C	3.9435554421	-0.97067394	1.8690137184
H	4.3524968993	-0.0351753792	1.4840211472
H	4.5333798658	-1.2676906549	2.7355739278
H	4.0613156031	-1.7319271755	1.0951997066
O	3.2324995822	-2.4530709961	4.4844253686
H	3.8231324156	-3.2914590226	5.8340777221
O	4.2598252305	-3.9096710847	6.4651130869
C	5.0737391726	-4.7564581886	5.733340975
H	4.7689257815	-4.8494467975	4.6848060121
C	4.9651119702	-6.1492116301	6.3501523869
C	6.5089578276	-4.2218015951	5.723630398
F	5.7881739211	-7.0210275299	5.7646013526
F	3.7199356159	-6.6057627164	6.2011932994
F	5.2367839064	-6.1443459574	7.6537955623
F	6.5075263733	-2.9750126791	5.2479380309
F	7.0462167623	-4.1874773656	6.9431250589
F	7.3113517923	-4.9490694219	4.9439141902

S6-h

E(RM062X) = -1100.98556213

E(RωB97XD) = -1101.14922962

Charge = 0 Multiplicity = 1

C	0.1162024297	0.6241931524	0.2145923798
H	0.6798084805	-0.2351871096	-0.1492162868
H	-0.8550360573	0.2543759779	0.5520120223
C	-0.1138322313	1.6065394148	-0.8981208391

Go back to table of contents

C	-0.5501417741	1.0271514758	-2.2177685893
H	-1.2709518506	0.2345591736	-1.993727683
H	0.3297972474	0.5077299644	-2.6171249357
H	-1.4473299905	1.3545691381	-4.0887443728
H	0.6322529382	1.0932954849	1.0489729596
C	-1.1215379713	1.9898552225	-3.2597362
C	-2.3521218231	2.7287002304	-2.7370445538
H	-2.0820376028	3.4191889321	-1.9375377048
H	-2.8171606569	3.3047648609	-3.538402777
H	-3.0962383472	2.0294489585	-2.3498181164
C	-0.0769829831	2.9575529705	-3.8121032215
H	-0.4975833694	3.5311405319	-4.6397733987
H	0.2519625684	3.6572177438	-3.0449626062
H	0.797133019	2.4205016909	-4.1857168587
O	0.0436399944	2.8020653349	-0.7310488459
H	0.2637197605	3.4506963592	0.826178592
O	0.4328568034	3.7750353844	1.7452289112
C	-0.7319428543	4.1953870362	2.3580841224
H	-0.4996663604	4.5282225477	3.3721539535
C	-1.733248659	3.0442291581	2.4982354711
C	-1.3226533972	5.4020918681	1.6223926929
F	-2.7979371882	3.3864293766	3.2223819286
F	-1.1471970053	2.0118027011	3.1041793412
F	-2.1708460855	2.61254343	1.3096384636
F	-1.558292242	5.1298662715	0.3370011225
F	-2.4655306025	5.8218075687	2.1666439119
F	-0.4581881895	6.4160921496	1.6634911171

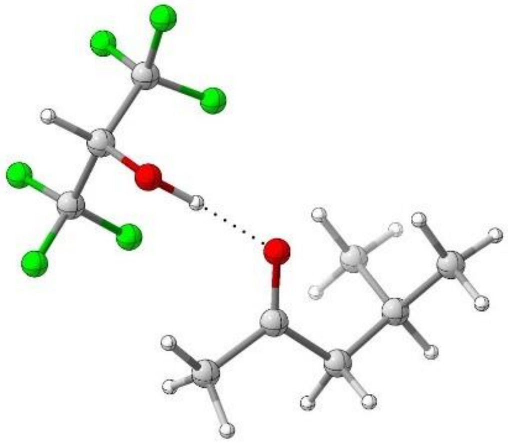

Go back to table of contents

$^3SO_2$

E(UM062X) = -548.516044583

E(UωB97XD) = -548.568531341

Charge = 0 Multiplicity = 3

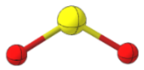

S	-1.7773873613	0.5024966893	0.1503940659
O	-3.2523276661	0.4294965398	-0.0061792745
O	-1.0681092926	0.8367351609	1.4115056786

^3TS_D-a

E(UM062X) = -1649.50355349

E(UωB97XD) = -1649.71817066

Charge = 0 Multiplicity = 3

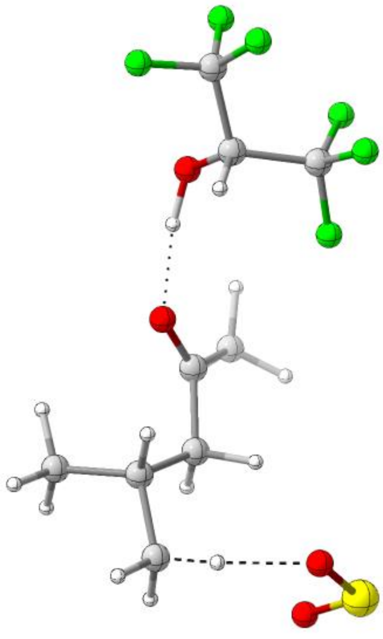

C	0.723908	2.163298	-1.180475
H	1.168852	2.658354	-2.046925
H	1.093933	2.676965	-0.292519
C	3.133627	-0.909958	-1.183727
C	1.176687	0.734802	-1.178029
C	2.618383	0.479211	-0.831505
H	2.683778	0.652715	0.250589
H	3.237088	1.259615	-1.285613
C	4.514195	-1.128527	-0.589732
H	4.889625	-2.135357	-0.776507
H	5.235036	-0.395628	-0.954562
O	4.289753	-0.514528	2.195786
O	5.212536	1.584541	1.088198
S	4.810119	0.970108	2.363572
H	-0.359238	2.233822	-1.237
C	3.191408	-1.107583	-2.700185
H	3.876614	-0.384672	-3.149317

Go back to table of contents

H	3.547334	-2.10854	-2.945584
H	2.206733	-0.976486	-3.148721
H	2.444403	-1.645296	-0.762711
O	0.416055	-0.182786	-1.429888
O	-2.22908	0.146632	-1.441643
C	-2.556777	-0.557365	-0.29563
H	-1.849744	-1.361408	-0.06595
C	-3.915109	-1.213878	-0.531745
C	-2.558001	0.383061	0.91348
F	-4.841446	-0.330824	-0.901966
F	-4.362006	-1.840034	0.558973
F	-3.812004	-2.119725	-1.50448
F	-3.478047	1.341941	0.812438
F	-2.767556	-0.266327	2.059294
F	-1.365049	0.979774	1.003048
H	-1.254334	0.080991	-1.568807
H	4.475681	-1.016961	0.521707

$^{3}TS_{D}$-b

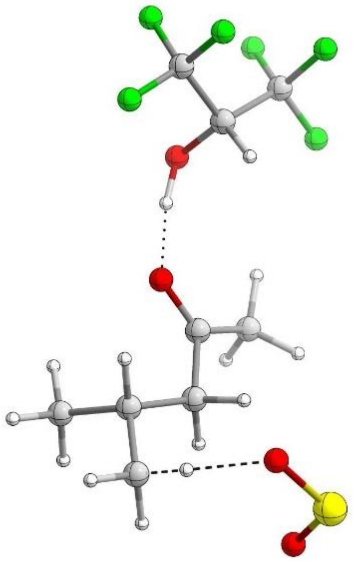

E(UM062X) = -1649.50320363

E(UωB97XD) = -1649.71828798

Charge = 0 Multiplicity = 3

C	0.3795310499	-0.7100150671	-2.9026111945
H	0.5090211312	-0.2211995654	-3.871504576
H	1.3666686473	-0.785213127	-2.4465517422
C	0.0807567557	-4.3613169315	-4.2581901229
C	-0.1895724369	-2.0750189249	-3.1454711483
C	0.7492616644	-3.1166585746	-3.6898190505
H	1.4001171437	-3.3787559602	-2.8453833829

Go back to table of contents

H	1.4091809421	-2.6479918808	-4.4268207903
C	1.121010751	-5.4217133895	-4.5747131946
H	0.6703980654	-6.35036324	-4.9269518629
H	1.8521130441	-5.0698985919	-5.3037636567
O	2.659777446	-5.7260152137	-2.1863162345
O	4.0045502493	-4.1739521918	-3.6924529936
S	3.9663735195	-4.8573977845	-2.3896487543
H	-0.2847626527	-0.1104822903	-2.2845855278
C	-0.7138926472	-4.0308272089	-5.5237280329
H	-0.0486570202	-3.6340049581	-6.294319509
H	-1.1960578832	-4.9245525479	-5.9205992868
H	-1.4862262614	-3.2900512249	-5.3174105542
H	-0.6084331989	-4.7514427796	-3.5059521706
O	-1.3538553227	-2.3389025645	-2.9021572557
O	-2.95523163	-0.5704072151	-1.7064421963
C	-2.8235009726	-0.9570679433	-0.3839127072
H	-1.9512476618	-1.5951402871	-0.2062294057
C	-4.0517372084	-1.7674846164	0.0397503915
C	-2.6269694854	0.3042880007	0.454978123
F	-5.1774883086	-1.0576077043	-0.0351795016
F	-3.9442006354	-2.2322190159	1.2860898448
F	-4.183081635	-2.8184162074	-0.7717924364
F	-3.607023202	1.1876563266	0.2724234183
F	-2.5595608433	0.0330886274	1.7596441238
F	-1.4827144728	0.8964274927	0.1063401486
H	-2.4144457863	-1.1823368153	-2.2572680751
H	1.6905858551	-5.6892936256	-3.6505656864

^{3}TS$_{D}$-c

E(UM062X) = -1649.50354643

E(UωB97XD) = -1649.71845268

Charge = 0 Multiplicity = 3

C	-0.2050021971	-0.5001605919	2.1326673306
H	0.0384092632	-0.0595351866	1.1619301498
H	0.2830174206	0.1112064203	2.8916494063
C	2.3806587364	-3.4180941298	1.9561694464
C	0.3376355738	-1.8973559123	2.1547691567
C	1.8234329401	-2.0503361204	2.3291026761
H	2.0080309825	-1.8332813445	3.3894942209
H	2.3296730446	-1.2514373813	1.7781132236
C	3.8448892279	-3.5164864298	2.3472309649
H	4.2561613385	-4.5102661771	2.1659583157
H	4.4531749091	-2.7691123452	1.8365596426
O	4.0051922044	-2.656972687	5.0572539556
O	4.6241181895	-0.6423721245	3.6255641015
S	4.4415633725	-1.1378208796	4.9992356785
H	-1.2837711524	-0.4940938519	2.2727988068
C	2.235260602	-3.6821077843	0.455366487
H	2.7834795551	-2.9287737832	-0.115464875
H	2.6370205926	-4.6618147038	0.1956696269
H	1.1882291916	-3.6531461896	0.1530907386
H	1.814125524	-4.1770107188	2.5000989899
O	-0.3885887459	-2.8691286456	2.0437306769
O	-2.970711182	-2.5377176877	1.4601262085
C	-2.872526451	-2.2285188076	0.1147940142
H	-1.8853654274	-1.8484445332	-0.1722133619
C	-3.8750093907	-1.1141474519	-0.1771949588

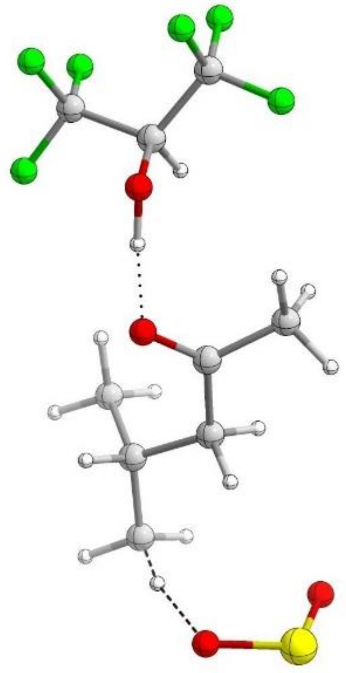

Go back to table of contents

C	-3.1153552347	-3.4849000751	-0.7268242131
F	-5.1095530563	-1.436783189	0.2036795142
F	-3.9151945222	-0.8047035331	-1.4743408966
F	-3.5226435493	-0.0135907516	0.4902729474
F	-4.3403512127	-3.9810243692	-0.5555021907
F	-2.9440011002	-3.259434547	-2.0307100796
F	-2.2432070535	-4.4276601576	-0.3634279301
H	-2.062063681	-2.7114485057	1.7983729898
H	3.9622382879	-3.3331328246	3.4449502365

$^{3}TS_{D}$-d

E(UM062X) = -1649.50355264

E(UωB97XD) = -1649.71819745

Charge = 0 Multiplicity = 3

C	-0.335683628	-0.6367735473	2.1164979331
H	0.1245477746	0.1229903024	1.480091999
H	-0.1084247651	-0.3704555985	3.1492352866
C	2.4088729442	-3.3534151282	1.5442130539
C	0.2775449705	-1.9608632413	1.7759600092
C	1.6986103621	-2.1857396876	2.2161556588
H	1.6358109717	-2.3450567829	3.3007252256
H	2.2613940067	-1.2550760921	2.0938976222
C	3.7468679519	-3.6142390472	2.2138146636
H	4.2552244693	-4.4841461405	1.796249495
H	4.4043392806	-2.7454872979	2.1607977725
O	3.2064438731	-3.8536037523	5.0109332309
O	3.9608030865	-1.4495180458	4.6417640625
S	3.5255023242	-2.4382653671	5.6411074247
H	-1.410216791	-0.646052468	1.9530727606

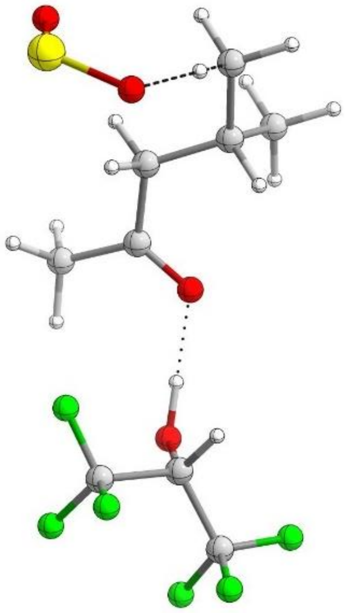

Go back to table of contents

C	2.6273684928	-3.0812507421	0.0541252612
H	3.2513831364	-2.194449085	-0.0800741705
H	3.129707397	-3.9235895456	-0.4222600156
H	1.6781685387	-2.9177578034	-0.4560194245
H	1.7797712267	-4.2402723476	1.6476026024
O	-0.3433625513	-2.8250916274	1.1831125287
O	-2.9963448661	-2.7522916932	0.9342908735
C	-3.3237873266	-3.7881671496	1.7925433895
H	-2.5264223797	-4.5314868687	1.8952748454
C	-4.5332073261	-4.5115275082	1.2042808767
C	-3.5901354424	-3.2360157253	3.1960278433
F	-5.5514810468	-3.6827197921	0.9769848787
F	-4.9709010329	-5.4843736045	2.0064138682
F	-4.2000674382	-5.0674954796	0.0393496454
F	-4.620273309	-2.3911401367	3.2237714589
F	-3.8290407769	-4.2024137394	4.0834911847
F	-2.5109390284	-2.5662185836	3.6130709928
H	-2.0144369394	-2.6796133998	0.9117718337
H	3.5961348409	-3.838896274	3.2985683292

^{3}TS$_D$-e

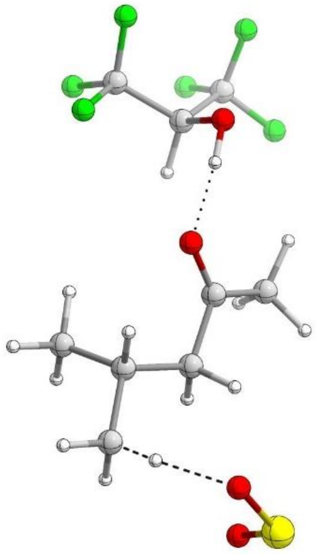

E(UM062X) = -1649.50355077

E(UωB97XD) = -1649.71845424

Charge = 0 Multiplicity = 3

C	-0.1839600222	-0.4859628264	2.0906434728
H	0.0582911047	-0.0650182856	1.1108800205
H	0.3110723854	0.1364286563	2.8360190624
C	2.382508552	-3.4223123352	1.9523758536
C	0.3500277867	-1.885862516	2.1373935426

Go back to table of contents

C	1.8360390644	-2.0447801351	2.3046163549
H	2.029006497	-1.8102263183	3.3597772846
H	2.3436308647	-1.2589386079	1.7364337143
C	3.8486174701	-3.5227224254	2.3357548994
H	4.2530122053	-4.5217118427	2.1682269285
H	4.4579927288	-2.7873686397	1.8091019401
O	4.0317769891	-2.6260055808	5.0333156546
O	4.6456753821	-0.6328873343	3.5701158375
S	4.4750377325	-1.1099787474	4.9518215397
H	-1.2620242169	-0.4702783399	2.2354750239
C	2.2253878755	-3.7117180965	0.4574682155
H	2.773933019	-2.9716693096	-0.1301856835
H	2.6197087957	-4.6981718065	0.2123135659
H	1.1764586766	-3.6819355035	0.1618782312
H	1.8150335621	-4.1680773812	2.5132409842
O	-0.3830440754	-2.8550410244	2.0515296386
O	-2.9717245193	-2.5194744538	1.4972774204
C	-2.8874656627	-2.2273862076	0.1471704292
H	-1.902602952	-1.8529018616	-0.1546669459
C	-3.8909147893	-1.1147638402	-0.1480701028
C	-3.141319406	-3.4937198854	-0.6760991988
F	-5.1219635874	-1.4303329605	0.2496063268
F	-3.944035406	-0.8212486039	-1.4484338892
F	-3.5296590806	-0.0066958716	0.5020290272
F	-4.3651022891	-3.9857534388	-0.4854277407
F	-2.9837886469	-3.2848583197	-1.9844978902
F	-2.2666100603	-4.4332021518	-0.3103529515
H	-2.0598840963	-2.6930101404	1.8267423413
H	3.9738571186	-3.3220168646	3.4295040928

Go back to table of contents

[3]TS$_D$-f

E(UM062X) = -1649.50357890

E(UωB97XD) = -1649.71817428

Charge = 0 Multiplicity = 3

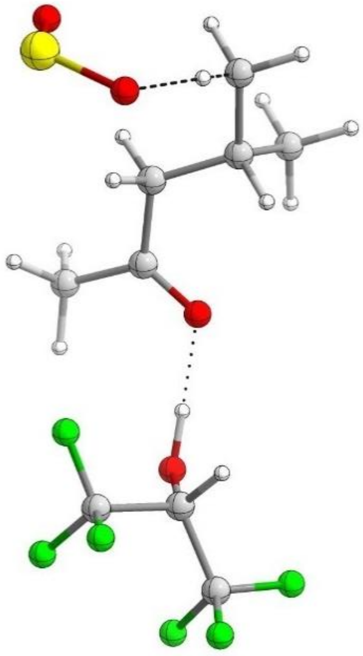

C	-0.3844439864	-3.7231746868	0.1045470924
H	-0.2478856244	-4.7768916141	-0.1506514556
H	0.151131785	-3.1399226592	-0.6450454373
C	2.2703503851	-3.6497786205	2.9655501344
C	0.2096582687	-3.4948445938	1.4610858482
C	1.7112295017	-3.5062211607	1.5562637581
H	2.0284447077	-2.5542518993	1.1103596359
H	2.1047690882	-4.2818230868	0.8918488727
C	3.7736329218	-3.4336844042	2.9629582313
H	4.1975069458	-3.4625844426	3.9675074901
H	4.2873865405	-4.1560801807	2.3275738618
O	4.2236518294	-0.9753432056	1.5852807369
O	4.5975957505	-2.8270440914	-0.1242684485
S	4.6000955409	-1.3727784451	0.101799208
H	-1.4439272393	-3.4807414854	0.0912705743
C	1.9538550129	-5.030439371	3.5451450056
H	2.4062153875	-5.8111350683	2.9289297581
H	2.3509504031	-5.1248129036	4.5561712066
H	0.8779165594	-5.1999810789	3.5843345711
H	1.8022447133	-2.8911770887	3.5966211052
O	-0.4830503703	-3.2953148961	2.4431051678
O	-3.0767509821	-2.7225415399	2.2223976415
C	-3.0946689661	-1.3997002757	2.6290750914
H	-2.3081971006	-1.1577717543	3.3515316505

Go back to table of contents

C	-4.4306146776	-1.1540163127	3.3269090119
C	-2.8806210068	-0.4773272257	1.4249070985
F	-5.4656205376	-1.5079383253	2.5664246186
F	-4.590277916	0.1284954968	3.6604819965
F	-4.4907778335	-1.8742768856	4.4471973636
F	-3.8615812142	-0.5741490879	0.5278162443
F	-2.7770236116	0.8035697501	1.7816091204
F	-1.7419323282	-0.8165675134	0.8123499218
H	-2.1501669145	-3.0521302491	2.2898035159
H	4.0144219676	-2.4191660944	2.5584788082

^{3}TS$_{D}$-g

E(UM062X) = -1649.50540949

E(UωB97XD) = -1649.71924541

Charge = 0 Multiplicity = 3

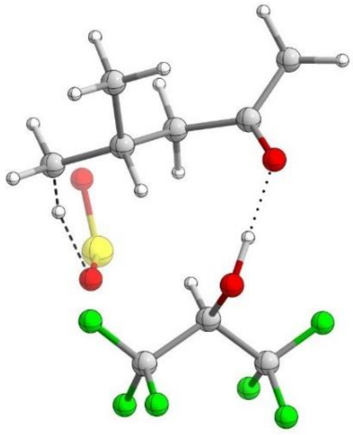

C	1.4178535927	-1.5003829448	0.4737394253
H	2.4118317127	-1.9420794794	0.5829736973
H	1.5454720618	-0.4187633162	0.5479082384
C	0.8886910746	-2.7198877474	4.0105099008
C	0.5618475926	-1.9802482293	1.6010334103
C	0.9529796975	-1.5719334017	3.0016514737
H	0.2573532193	-0.7719760471	3.283388777
H	1.9497696577	-1.1260978201	2.9986430821
C	1.1048506159	-2.2053993811	5.4212570053
H	1.0379058916	-3.0019609533	6.1630839519
H	2.0563169761	-1.6817743587	5.525515524
O	-1.00974647	-0.3035914873	5.656172789
O	1.0654077268	1.0921738779	5.2091588866
S	-0.3866856211	1.1417727465	5.4376409715

Go back to table of contents

H	0.9882464833	-1.7727005927	-0.4858736129
C	1.9324313781	-3.7869566313	3.673862911
H	2.9372496146	-3.3600929087	3.710568744
H	1.8851586609	-4.6114421139	4.3851016705
H	1.768461167	-4.1948631531	2.6745353796
H	-0.0990707821	-3.1833685999	3.9560798835
O	-0.4226317164	-2.6667028132	1.3980377287
O	-2.4443732085	-3.5113027265	2.9760755365
C	-2.7737510437	-2.3690063403	3.6849332144
H	-1.9407225212	-1.6644273777	3.7875187018
C	-3.1572714626	-2.8018291671	5.0992588832
C	-3.8995539098	-1.6098896431	2.9767987277
F	-4.0468902805	-3.7924670887	5.0925811657
F	-3.6717289042	-1.7982339836	5.8104126931
F	-2.0717025425	-3.2413931557	5.74099131
F	-5.0464011342	-2.2897669798	2.9721293204
F	-4.1300731567	-0.4219631372	3.5399845479
F	-3.5526830738	-1.3922667118	1.707428989
H	-1.6963767921	-3.2962692498	2.3779432634
H	0.3051974959	-1.4720670836	5.6907508094

$^{3}TS_{D}$-h

E(UM062X) = -1649.50357923

E(UωB97XD) = -1649.71801578

Charge = 0 Multiplicity = 3

C	-0.4247281288	-0.9182203879	-0.2564869816
H	-0.4996110593	-0.3021883323	-1.1558247619
H	-0.2446904184	-0.2428842887	0.5805694767
C	3.2318204988	-2.1214848046	-0.9035258305

Go back to table of contents

C	0.7353532998	-1.8509631451	-0.4279669646
C	2.1112011271	-1.245174483	-0.3599780741
H	2.2706243215	-1.0304560266	0.7049322396
H	2.0957825312	-0.2680709503	-0.8527452004
C	4.585084436	-1.5155793378	-0.5757882152
H	5.4110333969	-2.1448330709	-0.909514642
H	4.6956206821	-0.5138739557	-0.9932559733
O	4.5240961426	-1.1241469481	2.2527846324
O	4.0159532006	1.1488620618	1.226446636
S	4.1877113417	0.4049991196	2.4845622172
H	-1.3528191182	-1.4651004596	-0.1116189688
C	3.1031729688	-2.3003056765	-2.4180250925
H	3.1753733938	-1.3324957737	-2.9199972912
H	3.8990078446	-2.9400076609	-2.8003711831
H	2.1467528197	-2.7543111555	-2.6774811105
H	3.1569402876	-3.1026468408	-0.4295021901
O	0.5812351988	-3.0470723366	-0.599202382
O	-1.7158245623	-4.2591778435	0.0071276462
C	-1.3201278384	-4.9303351906	1.1515584179
H	-0.2583771654	-5.1980000753	1.149560251
C	-2.108962202	-6.236510634	1.212826919
C	-1.5459940769	-4.0465832572	2.3822286264
F	-3.4226644857	-6.0328803001	1.1254148251
F	-1.87529539	-6.9071961253	2.3428499703
F	-1.7555551369	-7.0220761509	0.1953751047
F	-2.8330349745	-3.7590048302	2.5740765404
F	-1.0802670275	-4.6063141173	3.4993735502
F	-0.8972963122	-2.8896994798	2.2132161296
H	-0.9331459464	-3.7780122406	-0.3475809171

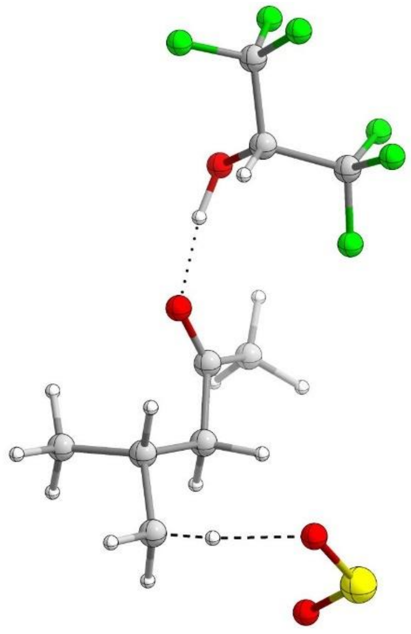

Go back to table of contents

H	4.7037683514	-1.4170863021	0.5317675963

^{3}TS$_{D}$-i

E(UM062X) = -1649.50310646

E(UωB97XD) = -1649.71740672

Charge = 0 Multiplicity = 3

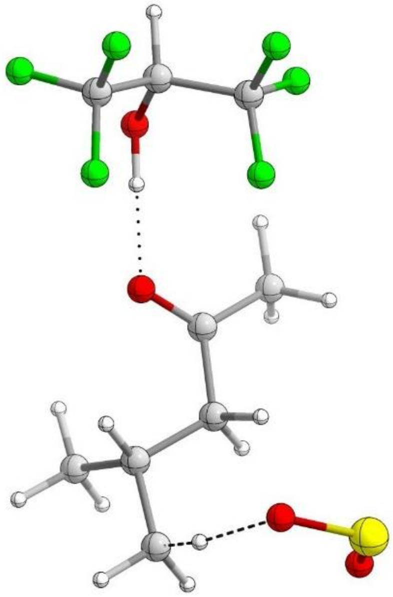

C	0.5887500871	-1.3963252148	-2.8009019016
H	1.1574348123	-1.0125536058	-3.6507233642
H	1.2723970411	-2.0038493495	-2.2061402663
C	-1.2453459735	-4.1917442274	-4.8198407327
C	-0.5287399162	-2.2479100069	-3.3242889996
C	-0.1416389183	-3.5361439103	-4.0006096591
H	0.178473384	-4.1975041226	-3.1849473585
H	0.7547494484	-3.3675899428	-4.6054038752
C	-0.8262133953	-5.5845577999	-5.2559780572
H	-1.6146025397	-6.0995570974	-5.8062413111
H	0.0862166757	-5.5674229654	-5.8537428732
O	-0.0961237572	-6.9003011447	-2.8227373019
O	2.0950101342	-6.174275611	-3.8955990859
S	1.4833340463	-6.8476641876	-2.7387684004
H	0.2089256566	-0.5659540883	-2.2111483806
C	-1.5888039121	-3.3502182638	-6.0510947867
H	-0.7151012287	-3.257014535	-6.7006318594
H	-2.3897843083	-3.8158706288	-6.6259162102
H	-1.9135232541	-2.3508580889	-5.7615447912
H	-2.1353786148	-4.2676387625	-4.1912870886
O	-1.6966519246	-1.9314066547	-3.193920583
O	-2.3843654167	0.1360169719	-1.6068734917
C	-2.7152943282	-0.2230824335	-0.3140030999

Go back to table of contents

H	-2.965094191	0.6775954067	0.2511640033
C	-1.5215260542	-0.8668644906	0.3981200002
C	-3.9627254869	-1.11155977	-0.2990711759
F	-1.1429356345	-2.0059294095	-0.1925326982
F	-1.776754131	-1.1371887327	1.6771585453
F	-0.4791864647	-0.0360597364	0.3607942565
F	-3.7799230096	-2.2336584082	-0.9987811936
F	-4.3270326256	-1.4555654096	0.9366001804
F	-4.9824202053	-0.4565062567	-0.85359483
H	-2.1632009824	-0.6521243854	-2.1607192457
H	-0.6123560125	-6.2190941379	-4.361112364

[3]TS$_D$-j

E(UM062X) = -1649.50303701

E(UωB97XD) = -1649.71731214

Charge = 0 Multiplicity = 3

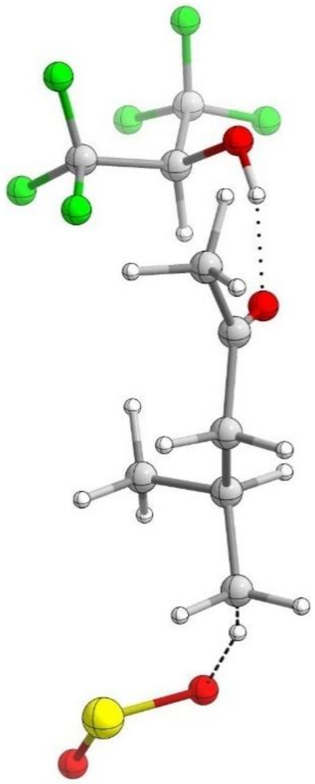

C	-1.5595790889	-1.6537669782	3.0561263139
H	-1.5370678178	-1.7748871994	4.1398712469
H	-1.8770125511	-2.6093171358	2.6324778849
C	2.2037017182	-2.1590965926	2.191616675
C	-0.1848553009	-1.3450393377	2.5471702367
C	0.9383966532	-2.2174974931	3.0476556715
H	0.5734281718	-3.2446492412	3.1406183527
H	1.1397152146	-1.8780554074	4.0708669095
O	5.3915258141	-3.1873930825	1.2312989972
O	6.081560738	-5.0609017968	-0.1028284184
S	5.1029727602	-4.7527747726	0.9535319748
H	-2.2661841686	-0.8772736349	2.7742307591
C	3.3308701991	-2.846345671	2.9373979546

Go back to table of contents

H	3.1317205354	-3.9061790366	3.1112663838
H	4.2747731872	-2.7927113501	2.3044337416
H	3.5993131795	-2.3601740356	3.8758505737
C	1.9703738458	-2.8024302521	0.8278946407
H	2.853067394	-2.6990008408	0.1952966897
H	1.7567672358	-3.8686712149	0.9420979739
H	1.1304777117	-2.3389441384	0.3097922149
H	2.461945429	-1.1078581717	2.0501423155
O	0.0286916771	-0.4273460484	1.7775360509
H	-1.2329198781	0.4173657616	0.9645442503
O	-1.8527509337	0.7337087964	0.2710658575
C	-1.4579576289	0.1661607938	-0.928835497
H	-0.3849266262	-0.0491488525	-0.9711736134
C	-1.7525856179	1.1776056795	-2.0340829693
C	-2.1810555636	-1.1673159822	-1.1425240709
F	-1.5016315882	0.6762191736	-3.2452829029
F	-0.983357141	2.2543234783	-1.8736077606
F	-3.0209464485	1.5844897701	-2.0170032601
F	-1.9605737226	-1.9583984019	-0.0870271637
F	-3.4999037316	-1.0195303786	-1.2618220617
F	-1.7409886572	-1.8118624064	-2.2236959514

^{3}TS$_{D}$-k

E(UM062X) = -1649.50459970

E(UωB97XD) = -1649.71761929

Charge = 0 Multiplicity = 3

C	-0.6452427603	-2.7025633626	-0.3305638923
H	-0.9481633069	-2.2730620983	-1.2888608066
H	-0.796638191	-1.9377823838	0.4307053637

Go back to table of contents

C	3.2305777002	-2.3344709289	-0.6825979481
C	0.8042800673	-3.0706411093	-0.4285968061
C	1.8014403576	-1.9499323314	-0.3227606266
H	1.7363597638	-1.6156863734	0.720883932
H	1.447581491	-1.1037172036	-0.9210105731
C	4.2183434299	-1.3244905319	-0.130647131
H	5.2510326343	-1.5878753394	-0.3579073932
H	4.010633052	-0.3064582505	-0.4689160159
O	3.4850399157	-1.6808176894	2.5363475016
O	4.8420743713	-3.7419742391	1.9152588273
S	3.9013265712	-3.1662489915	2.8893525793
H	-1.2562326624	-3.577331736	-0.1209803742
C	3.3939670065	-2.463640641	-2.1975037589
H	3.2102051894	-1.5025425154	-2.6836022204
H	4.4029579273	-2.7874578599	-2.4539251251
H	2.6893241831	-3.1926899231	-2.5997414846
H	3.4429488074	-3.3051099787	-0.2302818213
O	1.1633166333	-4.2264210449	-0.5661177116
O	-0.1180241806	-6.1298145937	0.799527994
C	0.7920647166	-6.1491548539	1.8442292014
H	1.8004728319	-5.8459036091	1.5428784911
C	0.8895495408	-7.5897901369	2.3430090962
C	0.347129754	-5.1726957707	2.9371341416
F	-0.298260304	-8.0775115851	2.6994563767
F	1.7070290806	-7.7002749358	3.3917408464
F	1.3693867586	-8.3669191951	1.3726699318
F	-0.8523525589	-5.473108493	3.4298820056
F	1.2081189309	-5.1228830479	3.9546087011
F	0.2700386605	-3.9404905411	2.4190494872

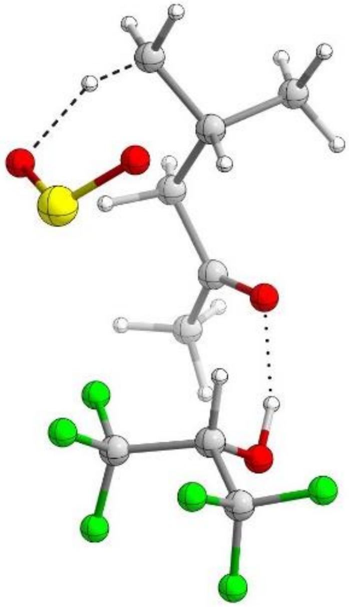

Go back to table of contents

H	0.2133072643	-5.4992723006	0.1249667845
H	4.1450103245	-1.287946405	0.9906754279

^{3}TS$_{D}$-l

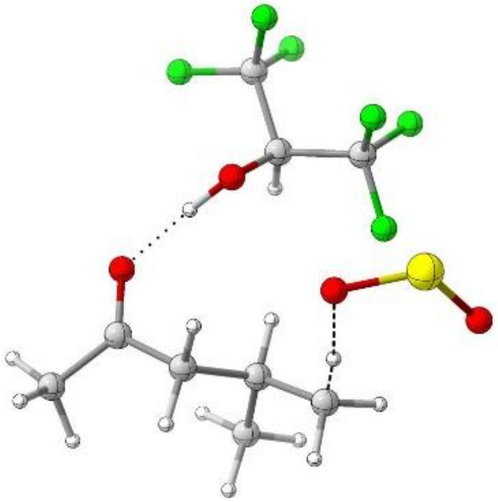

E(UM062X) = -1649.50427831

E(UωB97XD) = -1649.71782085

Charge = 0 Multiplicity = 3

C	-1.5776760103	-1.7132599306	1.3883055993
H	-1.6790370829	-0.7464267748	0.8890413607
H	-1.3361661277	-1.5014318354	2.4319597881
C	1.8256078651	-2.770259231	1.770133241
C	-0.4458961585	-2.4546733722	0.7548740646
C	0.9438569404	-1.8822854932	0.877578757
H	0.9059780581	-0.8699089912	1.2837670938
H	1.3784812764	-1.8394617636	-0.1247332974
O	4.021676511	-2.0746869053	-0.9361622696
O	6.1385724961	-2.8796658466	0.222950876
S	5.521160742	-2.5505242487	-1.0715875179
H	-2.5047425337	-2.2741295094	1.3118585014
C	3.2511332233	-2.253624342	1.7781066174
H	3.9268001496	-2.9080344267	2.3274893286
H	3.6327633813	-2.2100134688	0.7245140082
H	3.3198565007	-1.2365585572	2.1693025053
C	1.2836959337	-2.8501463971	3.1979626953
H	1.9416527557	-3.4520309941	3.8248687224
H	1.2122342562	-1.8524747071	3.637493447
H	0.2924420682	-3.3078205845	3.2215936051
H	1.8254783248	-3.7757890626	1.3429220248
O	-0.6293352345	-3.5052323067	0.1632903969

Go back to table of contents

H	0.6413895259	-4.0992641113	-0.7776131703
O	1.4174878217	-4.4295540235	-1.2896321867
C	1.7530350669	-5.6968032679	-0.8499955016
H	1.4034473731	-5.9145367232	0.165229813
C	3.2784356936	-5.7857010907	-0.8227203781
C	1.1182781676	-6.7503148317	-1.7635484814
F	3.7037285291	-7.0199437817	-0.5510351508
F	3.7578682205	-4.9761371744	0.1263537116
F	3.8233808341	-5.4107525222	-1.9784450144
F	-0.1974617793	-6.5395200666	-1.8258631918
F	1.5956389815	-6.695321886	-3.007297184
F	1.3063672302	-7.9901387717	-1.3081838136

$^{3}TS_{D}$-m

E(UM062X) = -1649.50480700

E(UωB97XD) = -1649.71731292

Charge = 0 Multiplicity = 3

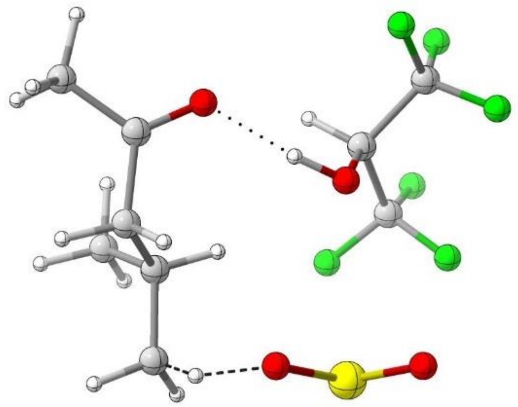

C	-1.5693157116	-2.0584288006	1.3454803428
H	-1.7930322591	-1.0263456468	1.0664148456
H	-1.4329022808	-2.0660232964	2.4297982804
C	2.0544510423	-2.5493152678	1.6131354724
C	-0.289370657	-2.4710640203	0.6951327653
C	0.9547838729	-1.6728272564	0.9995205761
H	0.7201872512	-0.8421651088	1.6676591374
H	1.3072602167	-1.2570534171	0.0507384218
O	3.844702039	-1.5270428788	-0.8317250703
O	4.8531668597	-3.0198422849	-2.4211128951
S	4.7501928389	-2.8602898701	-0.9620308742
H	-2.3846375479	-2.7157389261	1.0577087033

Go back to table of contents

C	3.3379680229	-1.7604476681	1.7524118776
H	4.1672056042	-2.360526214	2.1278537816
H	3.6500358815	-1.3868954311	0.7154483005
H	3.2273339931	-0.8493425339	2.3445027483
C	1.6275695541	-3.1149243748	2.9681652673
H	2.4190073117	-3.7307416159	3.3957839
H	1.4040282889	-2.3075130087	3.6690407649
H	0.7365462441	-3.7387595661	2.8686174849
H	2.2424485816	-3.3829949015	0.9330321704
O	-0.240599131	-3.4286384764	-0.0565095406
H	1.1513812881	-3.9316912166	-0.9534368191
O	1.8967990576	-4.4651279778	-1.3077614086
C	1.7337969897	-5.7602307428	-0.8423730197
H	1.0550465407	-5.8237395823	0.0148766557
C	3.0961756724	-6.2655768864	-0.3699071676
C	1.1320039805	-6.632613131	-1.9479438641
F	3.065272657	-7.5572659249	-0.0399946549
F	3.4757059574	-5.5821966349	0.7141244852
F	4.0399495562	-6.1029806141	-1.2951954161
F	0.0022058386	-6.073465064	-2.3826190105
F	1.9499669835	-6.7579098214	-2.9933801492
F	0.8334624627	-7.8584928392	-1.5137320916

^{3}TS$_{D}$-n

E(UM062X) = -1649.50672598

E(UωB97XD) = -1649.71762810

Charge = 0 Multiplicity = 3

C	7.7725963616	2.9938329971	-0.8651172218
H	8.615795851	2.4638469075	-1.3157424569

Go back to table of contents

H	7.8661463379	2.8948822035	0.2159904702
C	5.0223156886	0.2752529194	-1.4181183295
C	6.512398241	2.3438278653	-1.3514137549
C	6.1177455479	1.0478865562	-0.6967237083
H	5.7989123178	1.3362926444	0.3138785177
H	7.0140018956	0.4364696553	-0.5493848731
C	4.358088911	-0.7157621941	-0.4810814234
H	3.5439369094	-1.2625891201	-0.9577230906
H	5.0689901929	-1.4209962513	-0.0455033215
O	3.6890848422	0.6759182072	1.880410378
O	3.2405394134	2.4258341646	0.104361411
S	3.2698199818	2.1714714573	1.555403673
H	7.8088088726	4.0400807661	-1.1592113958
C	5.5866543873	-0.4483387049	-2.6420076534
H	6.3298211138	-1.1902482124	-2.3405383749
H	4.7958390037	-0.9614760293	-3.18971685
H	6.0649504874	0.2607202239	-3.3197173083
H	4.2726742003	0.9929335719	-1.7509995728
O	5.8290042217	2.8464878615	-2.2244958788
O	5.3207928197	5.4716741637	-2.0332479898
C	4.2027785199	5.2744989831	-1.2351165085
H	3.7757173774	4.2710906358	-1.3274453948
C	3.1305344586	6.2674193713	-1.6772070538
C	4.5978882455	5.4431620406	0.2338019463
F	3.5427899343	7.5320057513	-1.576982913
F	2.0124599161	6.1460218207	-0.9586428031
F	2.8154132746	6.047711149	-2.9532573928
F	5.1615898625	6.6248642548	0.4758977727
F	3.5618960126	5.3103046664	1.0655641454

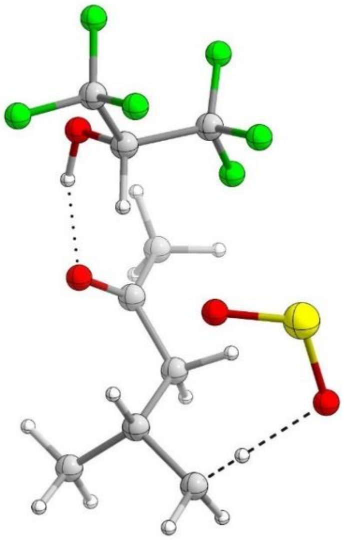

Go back to table of contents

F	5.4930391965	4.5008833405	0.5570276753
H	5.6651478769	4.5910784488	-2.2863774701
H	3.8951817265	-0.1685461151	0.3836907502

^{3}TS$_E$-a

E(UM062X) = -1649.50757855

E(UωB97XD) = -1649.71993460

Charge = 0   Multiplicity = 3

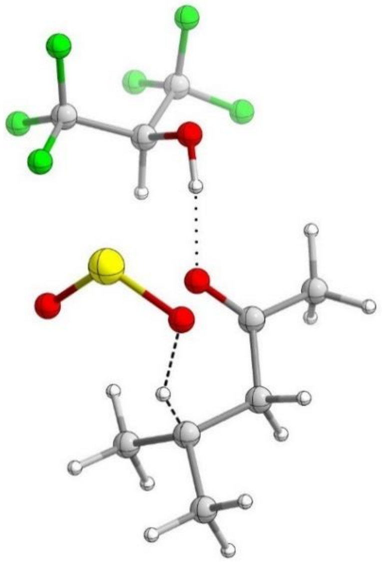

C	-0.123562	-0.964671	2.006366
H	0.102555	-0.210357	1.248456
H	0.228367	-0.576345	2.96199
C	2.866009	-3.366658	1.222266
H	-1.197042	-1.136762	2.033867
C	0.612939	-2.218475	1.644068
C	2.10029	-2.227608	1.875338
H	2.224028	-2.252993	2.96517
H	2.504419	-1.259376	1.559459
O	1.383645	-5.244566	2.758679
O	0.995851	-6.183346	0.44104
S	0.494074	-6.181549	1.823044
H	2.355282	-4.313651	1.511247
C	2.834416	-3.286813	-0.297484
H	3.335001	-2.37114	-0.62615
H	3.357656	-4.135464	-0.738191
H	1.813545	-3.280146	-0.676911
C	4.287402	-3.442286	1.759459
H	4.827917	-4.274482	1.308049
H	4.826849	-2.521122	1.522399
H	4.296242	-3.570402	2.842761

Go back to table of contents

O	0.036229	-3.191196	1.192157
H	-1.650163	-3.370499	1.24979
O	-2.621605	-3.41459	1.101448
C	-2.846813	-3.174178	-0.243439
H	-2.005699	-2.674174	-0.73562
C	-4.04716	-2.23691	-0.355505
C	-3.056571	-4.502791	-0.974744
F	-5.11569	-2.710112	0.283812
F	-4.394964	-2.018283	-1.625128
F	-3.743193	-1.057431	0.18939
F	-4.136418	-5.155022	-0.542667
F	-3.179776	-4.338387	-2.293272
F	-1.999556	-5.288446	-0.762796

$^{3}TS_{E}$-b

E(UM062X) = -1649.50757761

E(UωB97XD) = -1649.71924930

Charge = 0 Multiplicity = 3

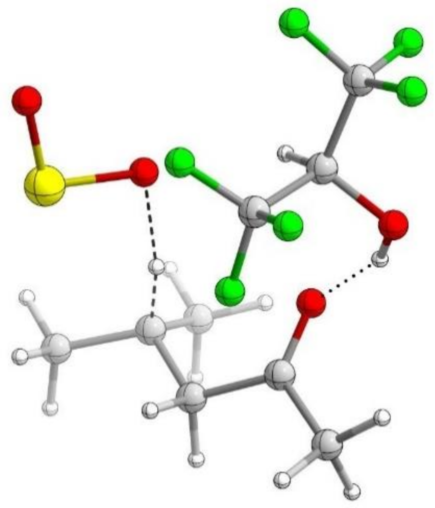

C	1.106729	-3.687258	4.942775
H	1.241138	-4.558624	4.296962
H	0.082176	-3.340354	4.811591
C	2.083572	-2.635026	4.511164
C	1.751334	-1.878552	3.25197
H	0.81909	-1.342297	3.467088
H	1.49485	-2.601903	2.469133
O	3.329286	0.868096	4.959792
O	2.567866	3.094577	5.438912
S	2.29319	2.015351	4.476603
H	3.12796	-0.313486	3.660053

Go back to table of contents

H	1.281543	-3.978793	5.975566
C	2.822491	-0.917072	2.76444
C	4.080107	-1.627204	2.283335
H	3.836503	-2.24142	1.411653
H	4.839313	-0.904792	1.982796
H	4.498183	-2.271097	3.054796
C	2.272851	0.025553	1.705262
H	3.016533	0.771464	1.421785
H	2.008185	-0.542664	0.809665
H	1.373638	0.542002	2.045339
O	3.088219	-2.391142	5.152119
H	3.010825	-2.570218	6.911868
O	2.851886	-2.267661	7.828375
C	2.891649	-0.881194	7.816075
H	3.4909	-0.476491	6.995028
C	3.546063	-0.431317	9.120074
C	1.479762	-0.313521	7.640602
F	3.533895	0.896535	9.251156
F	4.818188	-0.829502	9.143154
F	2.944058	-0.951069	10.189368
F	0.928883	-0.823375	6.532338
F	0.675737	-0.616811	8.658747
F	1.487135	1.013262	7.504321

^{3}TS$_E$-c

E(UM062X) = -1649.50852743

E(UωB97XD) = -1649.71989565

Charge = 0 Multiplicity = 3

C	0.485272375	-0.4212318705	0.9398642143

Go back to table of contents

H	1.0901597286	0.0309847178	0.1499910362
H	0.6996927237	0.1222141069	1.8600158832
C	2.7646752832	-3.5340158726	1.5842059354
H	-0.5671428785	-0.3358474628	0.6802348821
C	0.8955007776	-1.8567764853	1.0713700623
C	2.215417987	-2.1258740029	1.7429855826
H	2.0565199135	-1.8901226815	2.8024966692
H	2.9395237283	-1.3855204062	1.3854443085
O	0.564426821	-4.3857823291	3.1126126514
O	0.2278723123	-5.9406896794	1.1442399416
S	-0.4135622189	-5.3818976679	2.3417329442
H	1.951931527	-4.2393702628	1.864415573
C	3.1431786595	-3.8460030432	0.142454082
H	3.9491906299	-3.1788594786	-0.1769769816
H	3.4988900792	-4.8724930897	0.0515797605
H	2.2977041596	-3.714767608	-0.5318495741
C	3.9231715571	-3.7728332895	2.5410337837
H	4.3039189067	-4.7896292121	2.442412903
H	4.7415966268	-3.0827072195	2.3187197437
H	3.6197492394	-3.6158151399	3.5771315312
O	0.193479662	-2.7650722636	0.6630039165
H	-1.3182517917	-2.4506029547	-0.0737425266
O	-2.2730902989	-2.2977827405	-0.2540625705
C	-2.9802882113	-2.731446459	0.8536573871
H	-2.3374848573	-2.9310445158	1.7180461014
C	-3.6937876864	-4.0455522068	0.5258249421
C	-3.9453082841	-1.6216617377	1.2679312784
F	-4.6228955978	-3.897946485	-0.4182559316
F	-4.2823687413	-4.5758398941	1.6000762792

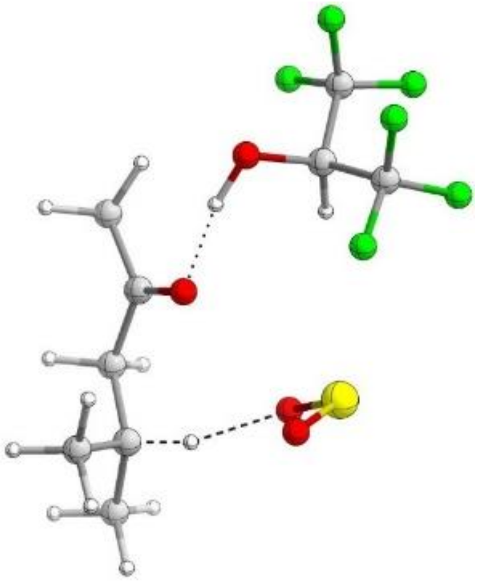

Go back to table of contents

F	-2.801427039	-4.9311905907	0.0785687374
F	-4.7209626365	-1.227338285	0.2599556059
F	-4.7395479618	-1.9974278633	2.2722142494
F	-3.2490244939	-0.5592340268	1.6779425986

^{3}TS$_{E}$-d

E(UM062X) = -1649.50795555

E(UωB97XD) = -1649.71969806

Charge = 0 Multiplicity = 3

C	-0.186434	-1.060423	1.453771
H	-0.075127	-0.684934	0.43376
H	0.225064	-0.304057	2.122687
C	2.848122	-3.51729	1.331727
H	-1.240329	-1.225243	1.66553
C	0.591812	-2.336009	1.572595
C	2.093927	-2.222038	1.584783
H	2.35136	-1.809999	2.568227
H	2.389472	-1.456815	0.858815
O	1.822241	-4.452654	3.765181
O	1.062392	-6.407028	2.343181
S	0.861725	-5.716143	3.626017
H	2.44441	-4.276299	2.041029
C	2.613014	-4.054281	-0.073663
H	3.007478	-3.343898	-0.806376
H	3.127579	-5.004973	-0.214433
H	1.552535	-4.204627	-0.273754
C	4.328353	-3.354054	1.640814
H	4.863577	-4.290281	1.480711
H	4.766779	-2.597798	0.983926

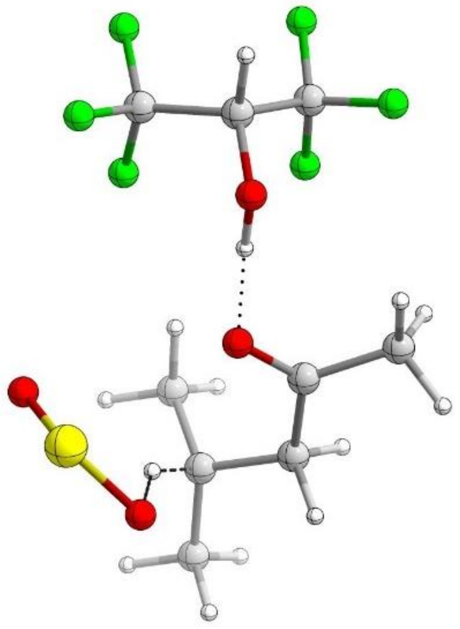

Go back to table of contents

H	4.484852	-3.037782	2.673077
O	0.042153	-3.417429	1.666154
H	-1.638889	-3.613499	1.534144
O	-2.624426	-3.68876	1.524449
C	-3.095476	-4.029455	0.27084
H	-4.185282	-4.094896	0.306816
C	-2.585821	-5.412315	-0.147358
C	-2.757406	-2.941904	-0.753646
F	-1.254406	-5.44647	-0.235669
F	-3.083261	-5.799697	-1.3227
F	-2.949817	-6.315503	0.762613
F	-1.436872	-2.779109	-0.891761
F	-3.26219	-3.203329	-1.958524
F	-3.259422	-1.77512	-0.348292

^{3}TS$_{E}$-e

E(UM062X) = -1649.50953185

E(UωB97XD) = -1649.72011057

Charge = 0 Multiplicity = 3

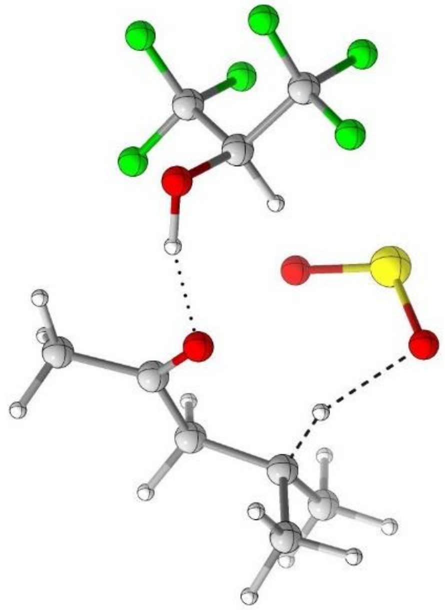

C	0.787081	-3.339719	4.831594
H	0.80766	-4.210739	4.17256
H	-0.16709	-2.836661	4.673194
C	1.921868	-2.435972	4.452714
C	1.801885	-1.705677	3.142867
H	0.959443	-1.017058	3.269673
H	1.490145	-2.420786	2.373059
O	3.982811	1.250097	4.327519
O	1.635122	0.758152	5.082083
S	2.682871	1.793526	5.093856

Go back to table of contents

H	3.426901	-0.423809	3.608057
H	0.877047	-3.664232	5.865177
C	3.050012	-0.952783	2.707416
C	4.155545	-1.895299	2.251023
H	3.836366	-2.428935	1.35124
H	5.062455	-1.340511	2.007963
H	4.396613	-2.629515	3.019367
C	2.718112	0.084722	1.646256
H	3.610299	0.638572	1.352027
H	2.312793	-0.402458	0.755547
H	1.974073	0.796336	2.009535
O	2.892232	-2.290608	5.173427
H	2.855024	-2.598129	6.90658
O	2.753806	-2.343156	7.84664
C	2.554507	-0.971179	7.870375
H	2.791273	-0.496131	6.913895
C	3.488652	-0.370504	8.917841
C	1.079123	-0.674266	8.143208
F	3.332031	0.950935	9.027533
F	4.755258	-0.598142	8.569011
F	3.302438	-0.903825	10.12512
F	0.335384	-1.240707	7.189604
F	0.670701	-1.16659	9.313544
F	0.816359	0.63335	8.138082

^{3}TS$_E$-f

E(UM062X) = -1649.50795891

E(UωB97XD) = -1649.71967844

Charge = 0 Multiplicity = 3

Go back to table of contents

C	-0.122892	-0.975731	1.833875
H	0.08798	-0.302043	0.999854
H	0.230016	-0.486311	2.741813
C	2.885515	-3.404323	1.256949
H	-1.193684	-1.15779	1.888546
C	0.627974	-2.252577	1.604999
C	2.119043	-2.216411	1.814811
H	2.261749	-2.138228	2.899521
H	2.50485	-1.278819	1.399771
O	1.550751	-4.983368	3.139859
O	0.929486	-6.355081	1.103477
S	0.584791	-6.080373	2.506537
H	2.388922	-4.325213	1.642984
C	2.820554	-3.469569	-0.263023
H	3.309407	-2.587174	-0.686751
H	3.339466	-4.353714	-0.6337
H	1.791521	-3.499841	-0.619983
C	4.317509	-3.418156	1.769181
H	4.857774	-4.284337	1.386519
H	4.842822	-2.518642	1.436728
H	4.347963	-3.444708	2.859365
O	0.06419	-3.279021	1.27527
H	-1.602395	-3.394756	1.001287
O	-2.584963	-3.472348	0.924152
C	-2.994258	-3.424678	-0.394764
H	-4.08055	-3.531632	-0.433939
C	-2.409562	-4.592345	-1.195766
C	-2.664895	-2.062754	-1.013624
F	-1.078209	-4.529107	-1.270724

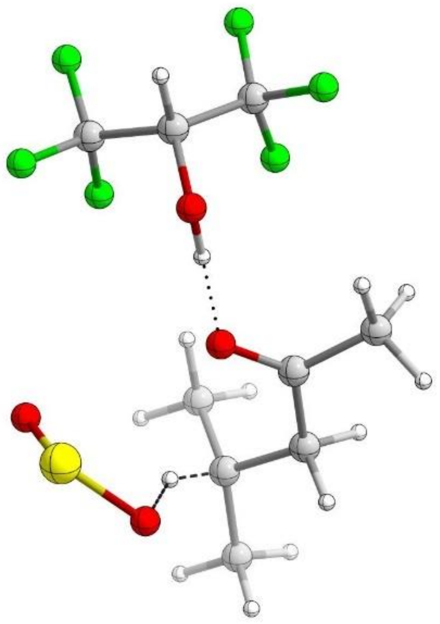

Go back to table of contents

F	-2.886706	-4.637525	-2.440582
F	-2.723126	-5.743103	-0.601515
F	-1.352368	-1.807954	-0.985149
F	-3.07031	-1.965145	-2.279147
F	-3.269633	-1.09887	-0.318482

[3]TS$_E$-g

E(UM062X) = -1649.50663740

E(UωB97XD) = -1649.71824760

Charge = 0 Multiplicity = 3

C	1.123674	-2.84441	5.241846
H	0.19223	-3.352949	4.981346
H	0.876864	-1.788223	5.371712
C	2.079885	-2.992411	4.104925
C	1.709764	-2.391067	2.769878
H	0.679756	-2.026904	2.78601
H	1.78731	-3.174579	2.012423
O	5.333011	-1.310544	2.415038
O	7.207578	-1.503628	3.892099
S	5.77936	-1.866584	3.861234
H	3.693433	-1.644375	2.394221
H	1.53721	-3.258993	6.15657
C	2.637167	-1.227299	2.405831
C	2.38827	-0.731295	0.994059
H	1.383214	-0.306312	0.924042
H	3.104629	0.047001	0.730211
H	2.46881	-1.542602	0.270312
C	2.60617	-0.109409	3.433671
H	3.288275	0.691402	3.148525

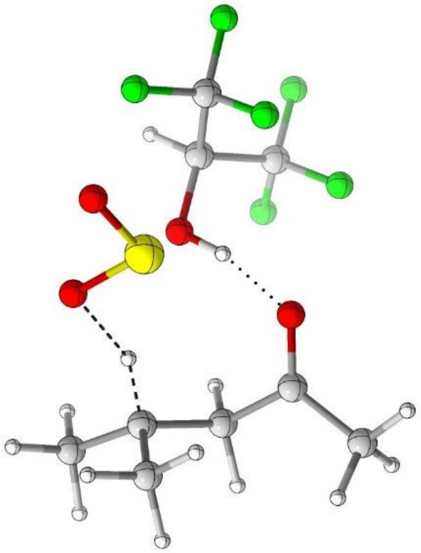

Go back to table of contents

H	1.59717	0.306818	3.498773
H	2.89385	-0.457643	4.428438
O	3.146369	-3.56726	4.237353
H	3.933608	-4.055639	2.75699
O	4.239699	-4.185861	1.829325
C	4.851962	-5.415602	1.663912
H	5.167517	-5.516943	0.623641
C	3.861383	-6.549684	1.940843
C	6.115906	-5.501493	2.524054
F	4.393654	-7.752631	1.731033
F	2.80746	-6.426204	1.133985
F	3.408581	-6.513333	3.197116
F	6.957535	-4.527839	2.176068
F	5.838143	-5.345796	3.821758
F	6.754747	-6.661097	2.37814

^{3}TS$_{E}$-h

E(UM062X) = -1649.50351536

E(UωB97XD) = -1649.71754825

Charge = 0 Multiplicity = 3

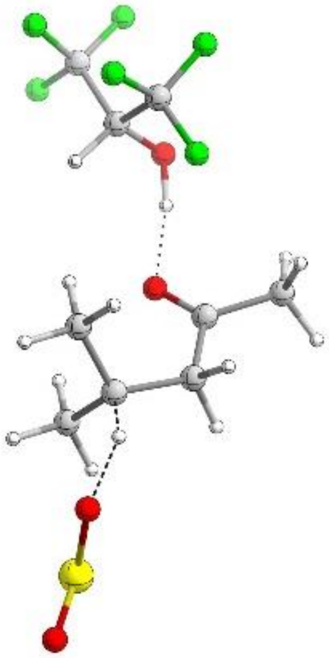

C	0.9049682523	-4.0393777948	3.9534030515
H	0.0035849665	-3.9158957342	3.3529708084
H	0.6813109183	-3.663590579	4.9544426014
C	2.0277612253	-3.2228059949	3.3842503564
C	1.6830615667	-1.8072352552	2.9900336611
H	1.0584931775	-1.3982375158	3.7898480141
H	1.0238245374	-1.8888962733	2.1180148358
O	2.2429627575	1.8608040957	2.0460789937
O	1.9527792499	3.0661414198	-0.0126345775

Go back to table of contents

S	2.1418165333	1.6777748233	0.4413752937
H	2.3684077449	0.1172716928	2.5258382859
H	1.1803643282	-5.08923023	4.0145534853
C	2.8533195203	-0.8753489548	2.6934643465
C	3.7779622228	-0.6787169334	3.8873063833
H	4.3339777005	-1.5956560972	4.0889087806
H	4.4970738024	0.1154242786	3.683959991
H	3.2160018442	-0.4117848389	4.7836169121
C	3.6117480057	-1.2322732776	1.4230290973
H	4.3462424676	-0.4599344594	1.1869799782
H	4.1417914434	-2.1756125229	1.5535981948
H	2.9371146083	-1.3367009294	0.5708202517
O	3.1483481594	-3.6785314749	3.2578002891
H	3.6305299021	-5.1092359217	4.1170237867
O	4.0478494875	-5.6898060725	4.7890220708
C	4.9125345213	-4.9062701494	5.5353310937
H	5.3096571594	-4.0527510226	4.9761740997
C	6.1046093326	-5.7793452559	5.9207734363
C	4.1854829692	-4.3313579759	6.7553781525
F	6.9436904161	-5.1405368093	6.738659884
F	6.7849117068	-6.1183310828	4.8256988706
F	5.7273133807	-6.9056823354	6.5234467571
F	3.0937194564	-3.6758943054	6.3476314364
F	3.7888635746	-5.2773998136	7.6057649093
F	4.9429350613	-3.4651007	7.4291694682

^{3}TS$_E$-i

E(UM062X) = -1649.50916111

E(UωB97XD) = -1649.71867427

Go back to table of contents

Charge = 0 Multiplicity = 3

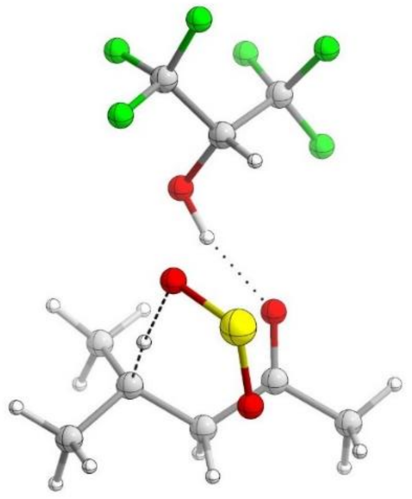

C	0.838141	-3.486918	5.250749
H	-0.121252	-2.970403	5.232852
H	1.378646	-3.143667	6.136449
C	1.660432	-3.123744	4.051393
C	1.672284	-1.669105	3.660587
H	1.828437	-1.102459	4.581986
H	0.658476	-1.41979	3.3228
O	5.139767	-2.846697	3.352174
O	3.995035	-2.174337	5.499994
S	5.139257	-2.920745	4.953721
H	3.594386	-1.920489	2.773604
H	0.697428	-4.562857	5.306631
C	2.693504	-1.29484	2.595321
C	2.186081	-1.606652	1.192448
H	1.376376	-0.915856	0.940863
H	2.976693	-1.487061	0.451338
H	1.793268	-2.620779	1.11486
C	3.124599	0.158786	2.724768
H	3.854634	0.417143	1.956853
H	2.261051	0.818569	2.605774
H	3.567132	0.349737	3.703455
O	2.301149	-3.969687	3.455131
H	3.198196	-4.12184	1.953331
O	3.708494	-4.434189	1.177089
C	4.520412	-5.480588	1.579456
H	4.798757	-5.427692	2.636863
C	5.813118	-5.39359	0.769352
C	3.796651	-6.816934	1.37825

Go back to table of contents

F	6.604839	-6.447244	0.98429
F	6.49298	-4.300554	1.113684
F	5.578262	-5.322489	-0.540128
F	2.615887	-6.778037	1.996098
F	3.568104	-7.082275	0.090988
F	4.484639	-7.84043	1.888914

[3]TS$_E$-j

E(UM062X) = -1649.50653562

E(UωB97XD) = -1649.71836451

Charge = 0 Multiplicity = 3

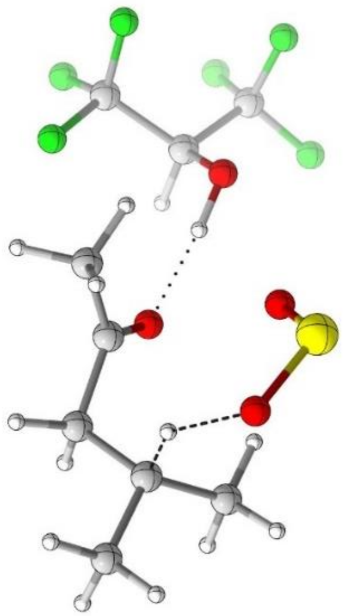

C	0.958483	-1.941386	1.215581
H	1.920284	-2.345155	0.89774
H	0.898696	-0.915103	0.842433
C	0.884997	-1.8835	2.711021
C	2.115761	-1.418034	3.438521
H	1.816984	-0.925063	4.366274
H	2.660432	-0.709679	2.812554
O	3.714035	-4.828264	2.149493
O	1.188616	-4.80654	2.151313
S	2.36685	-5.641441	1.863743
H	3.156046	-3.202676	2.852255
H	0.143768	-2.52742	0.799545
C	3.034436	-2.60512	3.785884
C	4.420181	-2.116924	4.179317
H	4.352128	-1.492462	5.074025
H	5.073453	-2.960668	4.404772
H	4.876257	-1.526123	3.38467
C	2.432905	-3.508236	4.851462

Go back to table of contents

H	3.073102	-4.376076	5.017915
H	2.353847	-2.960535	5.794085
H	1.439917	-3.858124	4.573252
O	-0.118414	-2.197633	3.326714
H	-1.345052	-3.013071	2.511672
O	-2.149664	-3.3742	2.068263
C	-3.194923	-2.497839	2.299382
H	-3.083982	-1.926953	3.227399
C	-4.468875	-3.328147	2.438466
C	-3.279377	-1.473266	1.16397
F	-5.552106	-2.560218	2.576352
F	-4.383161	-4.103776	3.519334
F	-4.666487	-4.124072	1.388213
F	-2.10472	-0.845531	1.054999
F	-3.541854	-2.038486	-0.01439
F	-4.210421	-0.544588	1.388268

$^{3}TS_{E}$-k

E(UM062X) = -1649.50461564

E(UωB97XD) = -1649.71632146

Charge = 0 Multiplicity = 3

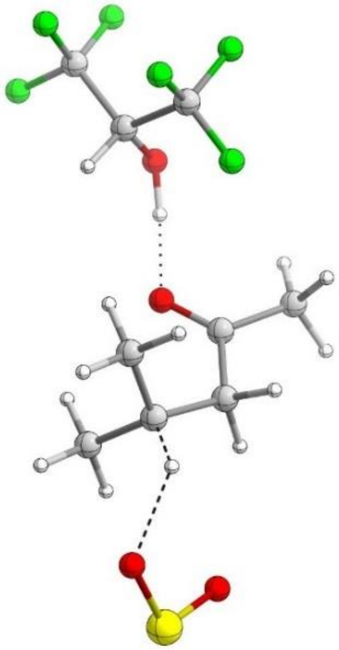

C	1.1929546991	-4.2279146359	4.0914042726
H	1.758441373	-4.9565454967	4.676449245
H	0.680690476	-4.7364689738	3.2785045608
C	2.1501277363	-3.1986087825	3.5645658968
C	2.7329700077	-2.2525579261	4.5815765316
H	2.9903987526	-2.8504292541	5.4613249973
H	1.9093285292	-1.605128178	4.9041347001
O	4.9108820195	0.8831500494	5.6673109074

Go back to table of contents

O	2.4507099529	0.704506259	6.1482552964
S	3.580701649	1.6460862965	6.160941636
H	4.2272670577	-0.868740187	5.0606675297
H	0.4720819294	-3.7633717977	4.7646095596
C	3.9238932875	-1.4051287434	4.1411638841
C	5.1215253231	-2.2467914695	3.7157203986
H	4.9096195935	-2.7684787808	2.781120156
H	5.9947375846	-1.6123373729	3.5574859553
H	5.3726838776	-2.989810387	4.4747805434
C	3.560785253	-0.3522266969	3.1008588897
H	4.4092191397	0.3103135497	2.9226279708
H	3.2891044524	-0.8224766471	2.1563898013
H	2.7182702024	0.2546989346	3.4384248418
O	2.4347933211	-3.1418214408	2.3821061785
H	2.0613582468	-4.4250897461	1.3133620666
O	2.1327348971	-5.2130691103	0.7289352268
C	3.4792287847	-5.4937980071	0.570502623
H	4.1172094806	-4.6076645138	0.6551654915
C	3.6693806277	-6.0456456946	-0.8404896742
C	3.9443236733	-6.4719555488	1.6538696171
F	4.9262114004	-6.4401552087	-1.0551706722
F	3.3837674503	-5.0989756643	-1.7345512999
F	2.8702944877	-7.0831759956	-1.0853642034
F	3.6599220136	-5.9609063659	2.856222602
F	3.3365942438	-7.6547612066	1.5685231689
F	5.2604304767	-6.6832992574	1.610843301

Go back to table of contents

^{3}TS$_E$-l

E(UM062X) = -1649.50450382

E(UωB97XD) = -1649.71611375

Charge = 0 Multiplicity = 3

C	1.07018	-4.00069	3.882734
H	0.439109	-4.179747	3.009102
H	0.448892	-3.520592	4.638387
C	2.195483	-3.095628	3.470227
C	1.826842	-1.662038	3.193413
H	1.405983	-1.275092	4.128507
H	0.990309	-1.675882	2.487072
O	2.414628	1.818882	1.361852
O	0.456207	0.333055	0.843734
S	1.179478	1.522914	0.369534
H	2.427803	0.23651	2.556625
H	1.451095	-4.949351	4.252831
C	2.931772	-0.740128	2.686318
C	4.04627	-0.518851	3.702782
H	4.626742	-1.431681	3.840062
H	4.72219	0.264796	3.35694
H	3.643385	-0.217409	4.671336
C	3.47045	-1.159848	1.323658
H	4.153632	-0.40234	0.936644
H	4.014109	-2.101574	1.399501
H	2.658372	-1.287103	0.605021
O	3.334737	-3.510856	3.358686
H	3.800329	-4.981669	4.089607
O	4.157801	-5.653163	4.713745
C	4.906275	-4.98552	5.668353

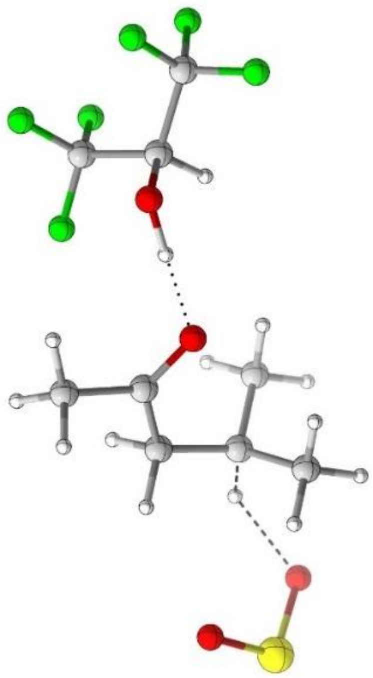

Go back to table of contents

H	5.341181	-4.050485	5.299424
C	6.068808	-5.896637	6.056432
C	4.024065	-4.615062	6.864864
F	6.792354	-5.384175	7.054012
F	6.879337	-6.060044	5.01079
F	5.65316	-7.104566	6.434648
F	2.98216	-3.895628	6.434563
F	3.538322	-5.686234	7.491028
F	4.676164	-3.875642	7.762814

S7

E(UM062X) = - 1100.31821699

E(UωB97XD) = -1100.47697294

Charge = 0 Multiplicity = 2

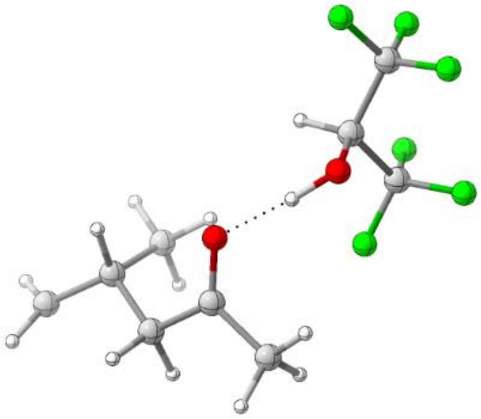

C	0.4150871609	0.0848117913	1.6689860752
H	0.9327107089	0.7149473808	2.3957480539
H	-0.4848422713	-0.2930369207	2.1559944953
C	2.824135152	-2.9384410592	2.1434461992
H	1.9332156406	-4.6111557017	3.3403646507
C	1.3165218216	-1.0556983358	1.3073008084
O	1.8075569161	-1.1621264438	0.1980469421
C	1.5784767493	-2.0839708369	2.3756069037
H	0.6790861991	-2.7111623888	2.4056088449
H	1.623138554	-1.5829103946	3.3470380527
H	0.1554172231	0.6754404291	0.794085583
C	2.8467388016	-4.0926436131	3.0827307102
H	3.7871570023	-4.5471361772	3.3618164607
H	2.7653276765	-3.3147260427	1.1122306301
H	1.7701137303	0.1178073744	-0.9432299759

Go back to table of contents

O	2.0342174035	0.8730543054	-1.5148315334
C	3.4007929684	1.0429170129	-1.370565959
H	3.9254780963	0.1148384495	-1.1200261444
C	3.6916819902	2.0297012987	-0.2355505162
C	3.9495417824	1.5157696679	-2.7145847161
F	4.9959911411	2.1292628453	0.0240125116
F	3.09284711	1.6009032148	0.8805076351
F	3.2302355618	3.2532788018	-0.4914983595
F	3.7997944477	0.5528552809	-3.6243149431
F	3.3126329977	2.5956768649	-3.1648736459
F	5.2497371728	1.8091807281	-2.6446296886
C	4.0976131167	-2.1004495868	2.2641139843
H	4.9794325655	-2.7125898922	2.0724167935
H	4.0968695444	-1.2737301267	1.5530483593
H	4.1832238313	-1.6895335704	3.2726377459

S8

E(UM062X) = - 1100.32736150

E(UωB97XD) = -1100.48751997

Charge = 0 Multiplicity = 2

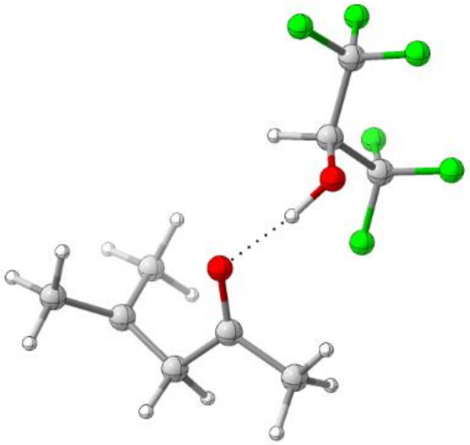

C	-1.663422	1.309711	1.257473
H	-1.337692	0.557841	0.53533
H	-2.625039	1.691392	0.912652
C	1.961221	3.510947	-0.279283
H	2.691328	4.23293	-0.650135
C	-0.649684	2.413747	1.281381
O	0.080236	2.599417	2.2368
C	-0.560575	3.293529	0.046971
H	-1.484501	3.883847	0.03351

Go back to table of contents

H	-0.616633	2.625939	-0.820092
H	-1.766119	0.851092	2.237828
C	0.649519	4.159955	-0.00077
C	0.632659	5.473803	0.699376
H	0.855618	5.354686	1.769095
H	1.387812	6.14879	0.291005
H	-0.342678	5.958224	0.626108
H	2.385925	3.068794	0.633937
H	1.866033	2.705469	-1.010362
H	0.322739	1.384553	3.442602
O	0.754295	0.666107	3.954457
C	2.049013	0.543531	3.478154
H	2.44049	1.474873	3.056162
C	2.102445	-0.499562	2.357474
C	2.942053	0.179791	4.661968
F	3.302868	-0.55484	1.779042
F	1.216924	-0.172089	1.41082
F	1.796536	-1.724356	2.784196
F	2.975268	1.193585	5.527074
F	2.496264	-0.891466	5.316386
F	4.196514	-0.068864	4.279848

SO_2H

E(UM062X) = -549.209143949

E(UωB97XD) = -549.261521364

Charge = 0 Multiplicity = 2

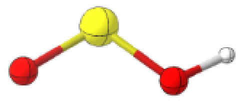

S	-1.9080698452	1.1053614603	-0.0267748944
O	-2.9980775717	0.2109803957	-0.4168286342
O	-2.5381737664	2.0132529492	1.1623489409

Go back to table of contents

H	-1.8598639567	2.6086942349	1.5182013976

S3

E(RM062X) = -859.711177280

E(RωB97XD) = -859.831939516

Charge = 0 Multiplicity = 1

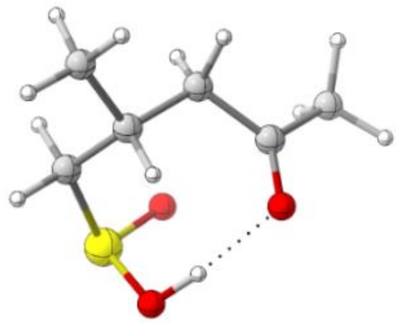

H	3.3935483758	-1.2158849262	-0.5485490049
C	2.4731063914	-1.7087437913	-0.236354015
H	1.968694364	-2.0707490169	-1.1363561951
H	2.6843372617	-2.5469820759	0.4220160645
C	0.3017921244	1.5183280311	0.260200098
H	0.1022346466	1.3095862457	1.3140687153
C	1.5371950381	-0.7422861613	0.4189384828
O	0.9060075142	-1.048646827	1.4134278385
C	1.4573714352	0.6305969944	-0.1984785499
H	1.4819251062	0.5272243522	-1.2860993808
H	2.4088361597	1.1038179573	0.0754675157
C	0.6942775749	2.992116518	0.1387067573
H	0.9446269027	3.2381527478	-0.8955644828
H	-0.1207743457	3.6428466662	0.4560189446
C	-0.9872922074	1.3087049357	-0.5343422872
H	-1.7768916637	1.9612274612	-0.1557805276
H	-0.837663779	1.5395844227	-1.5927257275
H	1.5650508962	3.2057980171	0.7592088072
S	-1.7246269554	-0.3383081582	-0.5507130234
O	-1.7380199849	-0.6364416055	1.0311616313
H	-0.8109090923	-0.695954538	1.3669455033
O	-0.7229576342	-1.2204536463	-1.1883459222

Go back to table of contents

S5

E(RM062X) = -859.705889738

E(RωB97XD) = -859.825465705

Charge = 0 Multiplicity = 1

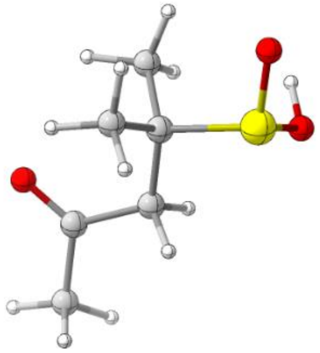

H	-1.1672758244	-0.0231226252	5.289411996
C	-0.5435794253	-0.0937553808	4.4024529234
H	-1.177385825	-0.1155820456	3.512952534
H	0.1017665696	0.780873236	4.3179397393
C	2.0896062658	-2.7841656081	3.2782444767
C	0.2732194824	-1.3574279915	4.441366452
O	0.0535069126	-2.2346175149	5.2424908591
C	1.3811729761	-1.4380211089	3.4080288912
H	2.0993198314	-0.6540651484	3.6709798261
H	0.9517130311	-1.1328972899	2.4471484533
C	2.8759904883	-3.1856667103	4.5177443799
H	3.5684609304	-3.9950394386	4.2802950065
H	3.4307065748	-2.3443849392	4.9378506716
C	1.141572227	-3.8939916861	2.8265145745
H	0.3744496397	-4.0403070158	3.5845925616
H	0.6525445374	-3.6392250735	1.8831213788
H	1.6873296191	-4.8277507259	2.6947478853
H	2.1848390746	-3.5354733547	5.2818330192
S	3.2542417581	-2.5890780566	1.859352967
O	4.2530154482	-1.4705549559	2.4783024496
H	4.7333885656	-1.8324981718	3.2425805202
O	4.0013593512	-3.8546845921	1.7850029729

Go back to table of contents

HFIP

E(RM062X) = -789.904885443

E(RωB97XD) = -789.989591603

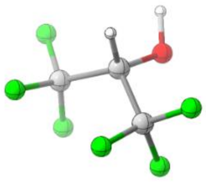

Charge = 0 Multiplicity = 1

O	-1.3112772363	-3.2907727311	2.3981557222
H	-1.0767821938	-2.3575823951	2.4515772235
C	-2.2264097643	-3.6072255418	3.3967023894
H	-2.0718357292	-3.0479666967	4.323211754
C	-2.016863591	-5.0841719646	3.7257713412
C	-3.6509138606	-3.3040984255	2.9205125955
F	-3.7143163825	-2.0320368421	2.5205376432
F	-4.0159148319	-4.0686042334	1.8928309674
F	-4.5452208695	-3.4677927063	3.8944132275
F	-2.9394999187	-5.5297119254	4.5785768255
F	-0.8221852528	-5.2520313798	4.2910886331
F	-2.0592458892	-5.8497678284	2.6379405874

6.3 - ωB97XD / def2-QZVPP / PCM (MeCN) // M06-2X (D3) / def2-TZVP / PCM (MeCN)

CH_4

E(RM062X) = -40.5004994644

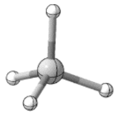

E(RωB97XD) = -40.5240597367

Charge = 0 Multiplicity = 1

C	-0.2540747666	-1.2025152009	-1.4398748672
H	0.1771153762	-2.1980732892	-1.3587031746
H	-0.7996074611	-1.1182340561	-2.3774159573
H	0.5405710668	-0.4599830522	-1.4148030627
H	-0.934927682	-1.0331983349	-0.6082984514

Go back to table of contents

$^3SO_2$

E(UM062X) = -548.515729039

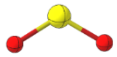

E(UωB97XD) = -548.568709860

Charge = 0 Multiplicity = 3

S	-1.7771947109	0.5024332492	0.1501252313
O	-3.2521948165	0.4295704429	-0.005898272
O	-1.0684347926	0.8367246977	1.4114935108

^3TS_A

E(UM062X) = -589.011318541

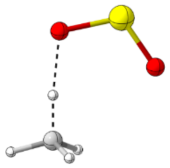

E(UωB97XD) = -589.086213660

Charge = 0 Multiplicity = 3

C	-0.2883949416	-1.2356595123	-1.4385474145
H	0.1836749894	-2.2100165979	-1.3546616668
H	-0.7988462631	-1.1099285308	-2.3902742945
S	2.4153985926	0.3467629148	0.1555457261
O	1.7304610084	-0.6920180903	0.9412589035
O	1.7667020911	0.5523133581	-1.2755355676
H	0.5359813328	-0.4600898781	-1.4214245145
H	-0.9410128098	-1.0317516635	-0.5949701716

SO_2H

E(UM062X) = -549.209711301

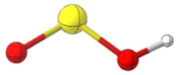

E(UωB97XD) = -549.262032453

Charge = 0 Multiplicity = 2

S	-1.9174051363	1.1275553917	-0.0478551701
O	-2.9875794151	0.1924352164	-0.3964449601

Go back to table of contents

O	-2.5541825277	2.0464715889	1.1283244614
H	-1.9019211709	2.6993685703	1.4273430826

CH_3

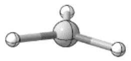

E(UM062X) = -39.8246896962

E(UωB97XD) = -39.8432636321

Charge = 0 Multiplicity = 2

C	-0.08576695	-1.2479759174	-1.6509235425
H	0.1318792184	-2.2355521669	-1.2781662931
H	-0.8907148261	-1.1021166059	-2.3526505839
H	0.5021339133	-0.4064219321	-1.3226459226

S9

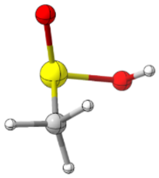

E(RM062X) = -589.133206909

E(RωB97XD) = -589.200337064

Charge = 0 Multiplicity = 1

C	-2.0482241751	1.1059234148	0.2555061273
H	-3.1143385332	1.1638162838	0.4659242626
H	-1.4874685976	1.6869505776	0.9879468058
S	-1.7911108656	1.8417903883	-1.3532255123
O	-2.0592422499	3.2761150115	-1.1560283392
O	-0.1863223312	1.6326339764	-1.4489477223
H	0.2665445541	2.0388056042	-0.690919508
H	-1.7200186416	0.0691657234	0.2303744761

^3TS_F

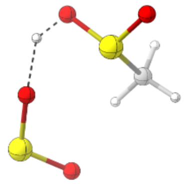

E(UM062X) = -1137.67966292

E(UωB97XD) = -1137.79651614

Go back to table of contents

Charge = 0 Multiplicity = 3

H 0.8662161432 -1.3127933645 0.7783433391

C 0.1907474945 -0.4709439731 0.6508881291

H -0.8476069705 -0.7922494453 0.6332155159

H 0.3653706581 0.3463509561 1.3495309339

S 0.5268247601 0.1961966211 -0.96983399

O -0.3966984575 1.2654204767 -1.2659595309

O 1.9796882948 0.7369722322 -0.8978328612

H 2.5042433696 -0.0320106046 -1.2947160386

O 2.1051820911 -1.4651122847 -1.9859958587

S 2.1822761533 -2.9220906841 -1.4951956431

O 1.0376227721 -3.2221996523 -0.5903127669

S10

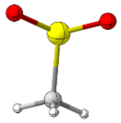

E(UM062X) = -588.497977733

E(UωB97XD) = -588.566696490

Charge = 0 Multiplicity = 2

C -2.033298176 1.1173907702 0.3026272415

H -3.103865252 1.2110031069 0.4559635007

H -1.4681907545 1.686938365 1.0389780916

S -1.6526361935 1.8577778132 -1.2898178466

O -2.1849438337 3.2097899849 -1.2929313155

O -0.2402847406 1.6448203306 -1.5558747542

H -1.7135845697 0.0806961491 0.2634527124

TS_F

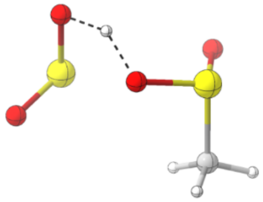

E(RM062X) = -1137.71921397

E(RωB97XD) = -1137.82950979

Charge = 0 Multiplicity = 1

Go back to table of contents

C	-1.6586125907	1.1408907846	-0.694297586
H	-2.622450931	1.4494082172	-0.294620203
H	-0.8588269408	1.7608846073	-0.2908266609
S	-1.7225077788	1.4056031396	-2.459944396
O	-1.7614652192	2.8667492329	-2.640696999
O	-0.2165890329	0.9151423302	-2.7602927333
H	0.4104539499	1.2446109014	-3.8251719617
O	1.4370151894	1.8098087871	-3.9874390212
S	1.5373526221	2.0371443903	-2.4893657764
O	2.486428333	1.1682766558	-1.8785936041
H	-1.4833070109	0.0850525237	-0.5007575884

SO_2

E(RM062X) = -548.632512189

E(RωB97XD) = -548.682296368

Charge = 0 Multiplicity = 1

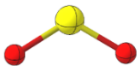

S	-1.7564509587	0.4953428241	0.1201496794
O	-3.1818278908	0.448165955	0.0614813155
O	-1.1595454707	0.8252196109	1.3740894752

$SO_2^{-\cdot}$

E(UM062X) = -548.763947284

E(UωB97XD) = -548.815572380

Charge = -1 Multiplicity = 2

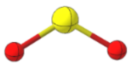

S	-1.7198910084	0.4828715721	0.0674194235
O	-3.2252314949	0.4497181366	0.0715427339
O	-1.1527018167	0.8361386812	1.4167583127

Go back to table of contents

6.4 - Orca 4.2.1 DLPNO-CCSD(T) single point calculations

DLPNO-CCSD(T) provided single point energy corrections to the Gaussian 16 M06-2X (D3) / def2-TZVP/ PCM (MeCN)-optimized geometries used for the calculation of the ΔG and $\Delta G^{\neq}$ of the HAT pathway shown in Figure 4, given the near-barrierless character of the pathway and the superior performance of DLPNO-CCSD(T) with a variety of systems.[45] The correlation-consistent cc-pVTZ[21] basis set was used in conjunction with the cc-pVTZ/C correlation fitting set and the "tightPNO" keyword controlled the DLPNO parameter thresholds.[46] Geometries and images for the corresponding structures are provided in **Section 7.3**.

DLPNO-CCSD(T) / cc-pVTZ // M06-2X (D3) / def2-TZVP/ PCM (MeCN)

CH_4

E(RM062X) = -40.5004994644
E(RCCSD(T)) = -40.438111043064

$^3SO_2$

E(UM062X) = -548.516248402
E(UCCSD(T)) = -547.856468017090

^3TS_A

E(UM062X) = -589.011318541
E(UCCSD(T)) = -588.285127209269

SO_2H

E(UM062X) = -549.209711301
E(UCCSD(T)) = -548.541418606207

Go back to table of contents

CH_3

E(UM062X) = -39.8246896962
E(UCCSD(T)) = -39.760983929895

S9

E(RM062X) = - 589.133206909
E(RCCSD(T)) = -588.394051194904

^3TS_F

E(UM062X) = -1137.67966292
E(UCCSD(T)) = -1136.269694320248

S10

E(UM062X) = -588.497977733
E(UCCSD(T)) = -587.755438032247

TS_F

E(RM062X) = -1137.71921397
E(RCCSD(T)) = -1136.316265247819

SO_2

E(RM062X) = -548.63171435
E(RCCSD(T)) = -547.967524722562

Go back to table of contents

X-Ray crystallographic data

1-(Allylsulfonyl)adamantan-2-one (14a)

CCDC 2091570

Bond precision:	C–C = 0.0020 Å		Wavelength = 0.71073
Cell:	a = 6.8529(2)	b = 9.2662(3)	c = 19.1898(5)
	α = 90	β = 90.051(2)	γ = 90
Temperature:	98 K		

	Calculated	Reported
Volume	1218.56(6)	1218.56(6)
Space group	P 21/c	P 1 21/c 1
Hall group	-P 2ybc	-P 2ybc
Moiety formula	$C_{13}H_{18}O_3S$	$C_{13}H_{18}O_3S$
Sum formula	$C_{13}H_{18}O_3S$	$C_{13}H_{18}O_3S$
M_r	254.33	254.33
D_x, g cm^{-3}	1.386	1.386
Z	4	4
Mu (mm^{-1})	0.259	0.259
F000	544.0	544.0
F000'	544.74	
h,k,l_{max}	8,12,24	8,12,24
N_{ref}	2787	2778
T_{min},T_{max}	0.911,0.982	0.883,1.000
T_{min}'	0.911	

Correction method = # Reported T Limits: T_{min} = 0.883 T_{max} = 1.000
AbsCorr = MULTI-SCAN

Data completeness = 0.997 Theta(max) = 27.497

R(reflections)= 0.0405(2728) wR2(reflections)= 0.0894(2778)

S = 1.051 N_{par}= 154

Go back to table of contents

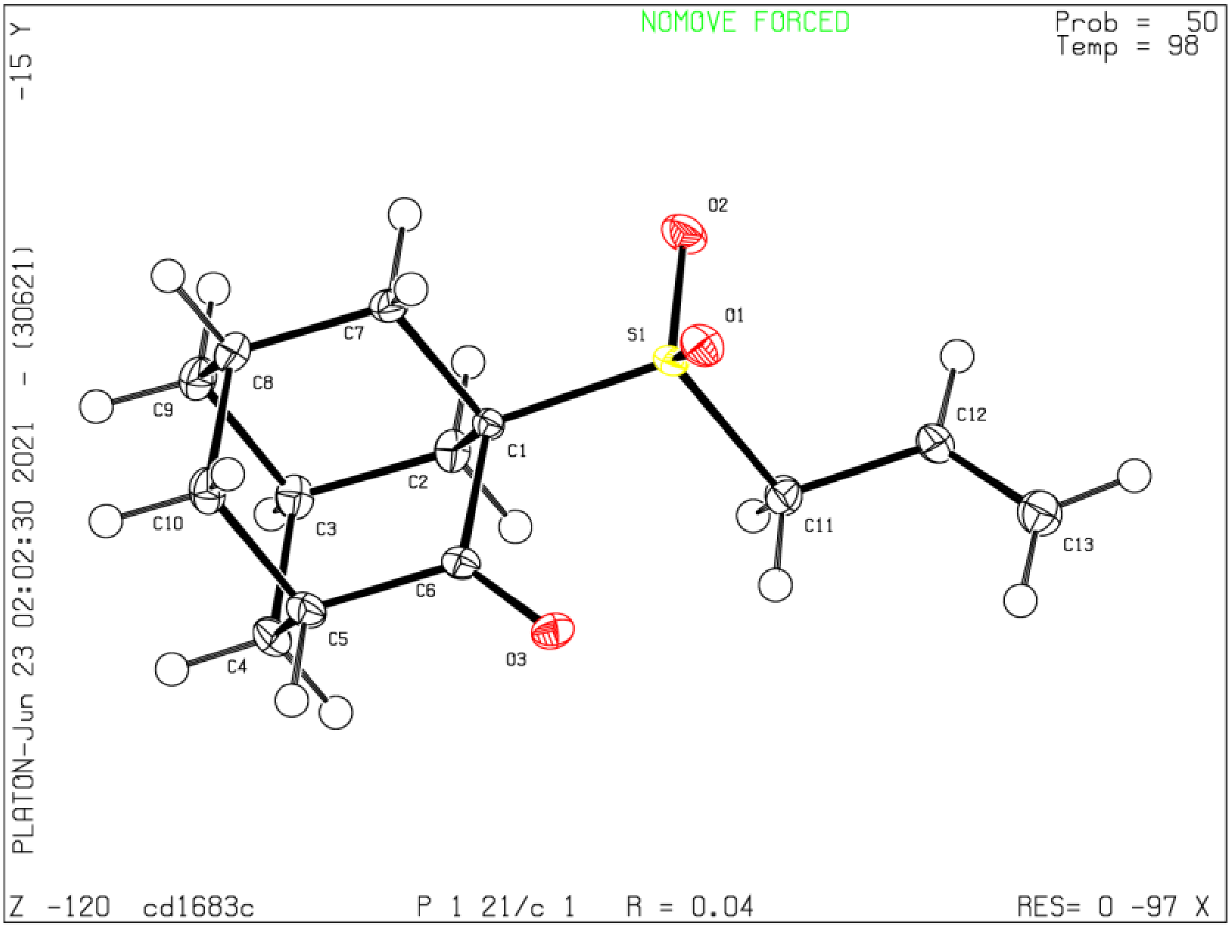

Go back to table of contents

4-(Cyclohexylsulfonyl)morpholine (37)

CCDC 2092038

Bond precision:	C–C = 0.0019 Å		Wavelength = 0.71073
Cell:	a = 11.7797(3)	b = 10.1425(2)	c = 9.6720(2)
	α = 90	β = 96.019(2)	γ = 90
Temperature:	98 K		

	Calculated	Reported
Volume	1149.20(4)	1149.20(4)
Space group	P 21/c	P 1 21/c 1
Hall group	-P 2ybc	-P 2ybc
Moiety formula	$C_{10}H_{19}NO_3S$	$C_{10}H_{19}NO_3S$
Sum formula	$C_{10}H_{19}NO_3S$	$C_{10}H_{19}NO_3S$
M_r	233.32	233.32
D_x, g cm^{-3}	1.349	1.349
Z	4	4
Mu (mm^{-1})	0.270	0.270
F000	504.0	504.0
F000'	504.72	
h,k,l_{max}	15,13,12	15,13,12
N_{ref}	2643	2642
T_{min},T_{max}	0.907,0.955	0.869,1.000
T_{min}'	0.874	

Correction method = # Reported T Limits: T_{min} = 0.869 T_{max} = 1.000
AbsCorr = MULTI-SCAN

Data completeness = 1.000 Theta(max) = 27.497

R(reflections) = 0.0341(2630) wR2(reflections) = 0.0848(2642)

S = 1.036 N_{par} = 136

Go back to table of contents

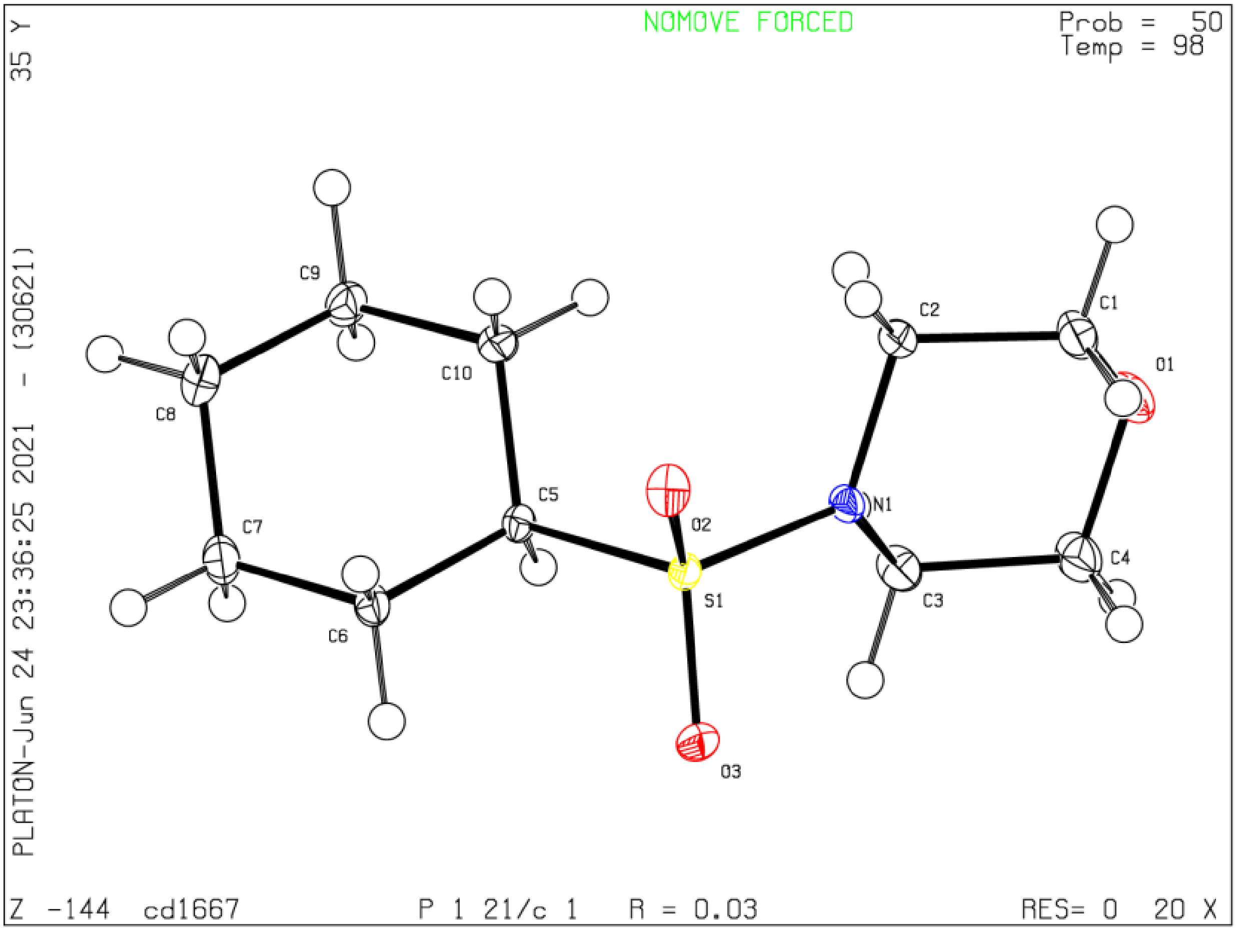

Go back to table of contents

(*R*)-*N*-(1-(4-Methoxyphenyl)ethyl)cyclopentanesulfonamide (39)

CCDC 2091573

Bond precision:	C–C = 0.0032 Å		Wavelength = 0.71073
Cell:	a = 9.1940(2)	b = 10.5230(3)	c = 15.2153(4)
	α = 90	β = 90	γ = 90
Temperature:	98 K		

	Calculated	Reported
Volume	1472.06(7)	1472.06(7)
Space group	P 21 21 21	P 21 21 21
Hall group	P 2ac 2ab	P 2ac 2ab
Moiety formula	$C_{14}H_{21}NO_3S$	$C_{14}H_{21}NO_3S$
Sum formula	$C_{14}H_{21}NO_3S$	$C_{14}H_{21}NO_3S$
M_r	283.38	283.38
D_x, g cm^{-3}	1.279	1.279
Z	4	4
Mu (mm^{-1})	0.224	0.224
F000	608.0	608.0
F000'	608.76	
h,k,l_{max}	11,13,19	11,13,19
N_{ref}	3372[1935]	3329
T_{min},T_{max}	0.948,0.993	0.944,1.000
T_{min}'	0.929	

Correction method = # Reported T Limits: T_{min} = 0.944 T_{max} = 1.000

AbsCorr = MULTI-SCAN

Data completeness = 1.72/0.99 Theta(max) = 27.497

R(reflections) = 0.0333(3267) wR2(reflections) = 0.0786(3329)

S = 1.059 N_{par} = 174

Go back to table of contents

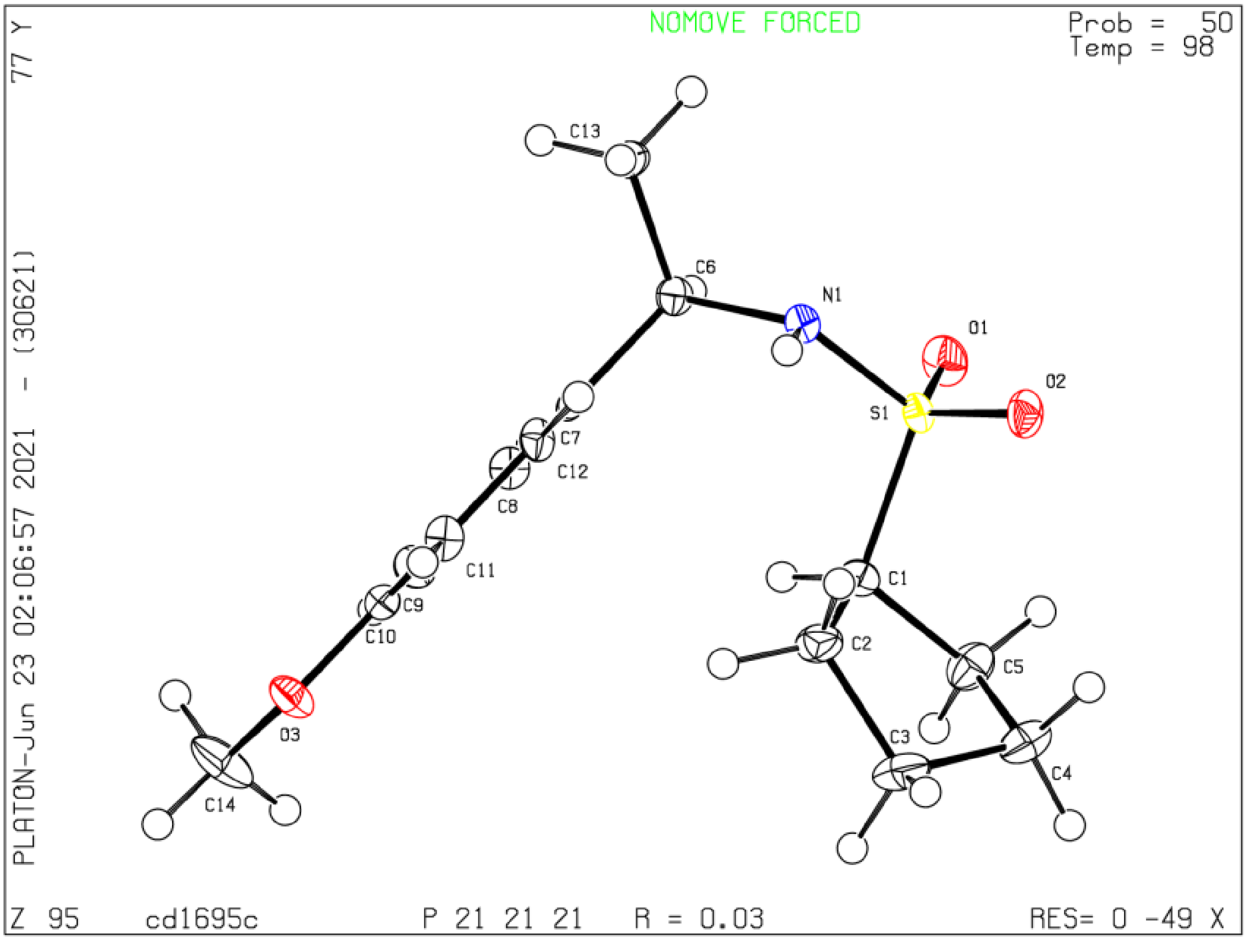

Go back to table of contents

7-Chloro-4-(cyclohexylsulfonyl)quinoline (42)

CCDC 2091572

Bond precision: C–C = 0.0025 Å Wavelength = 0.71073

Cell:	a = 5.5955(2)	b = 10.7380(3)	c = 11.8452(3)
	α = 99.357(2)	β = 93.406(2)	γ = 90.654(3)

Temperature: 98 K

	Calculated	Reported
Volume	700.84(4)	700.84(4)
Space group	P -1	P -1
Hall group	-P 1	-P 1
Moiety formula	$C_{15}H_{16}ClNO_2S$	$C_{15}H_{16}ClNO_2S$
Sum formula	$C_{15}H_{16}ClNO_2S$	$C_{15}H_{16}ClNO_2S$
M_r	309.80	309.80
D_x, g cm^{-3}	1.468	1.468
Z	2	2
Mu (mm^{-1})	0.422	0.422
F000	324.0	324.0
F000'	324.66	
h,k,l_{max}	7,13,15	7,13,15
N_{ref}	3214	3213
T_{min},T_{max}	0.890,0.947	0.868,1.000
T_{min}'	0.834	

Correction method = # Reported T Limits: T_{min} = 0.868 T_{max} = 1.000
AbsCorr = MULTI-SCAN

Data completeness = 1.000 Theta(max) = 27.499

R(reflections) = 0.0398(3195) wR2(reflections) = 0.0904(3213)

S = 0.998 N_{par} = 181

Go back to table of contents

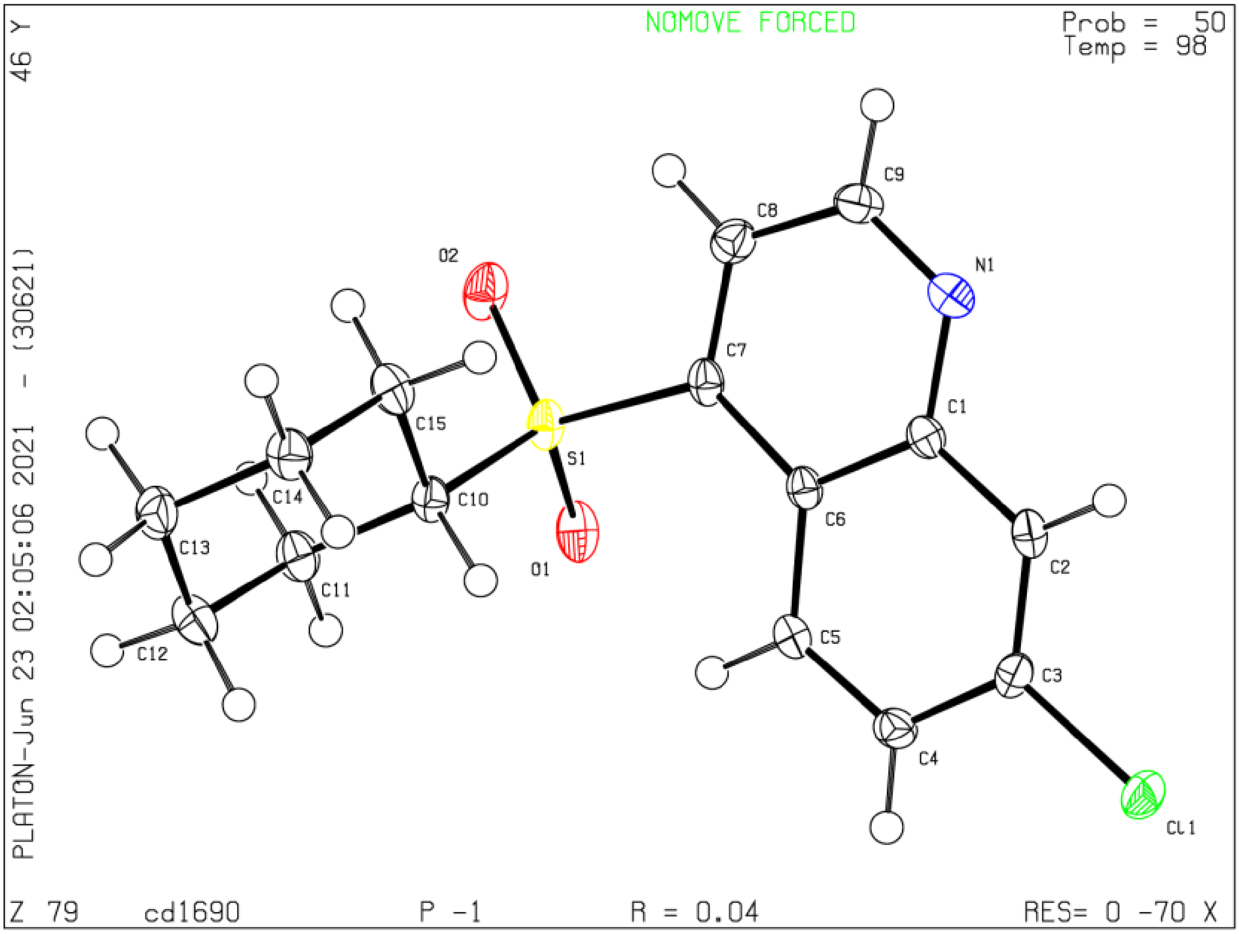
NOMOVE FORCED
Prob = 50
Temp = 98
46 Y
PLATON-Jun 23 02:05:06 2021 - (30621)
O2
C8
C9
N1
C7
C15
C1
S1
C14
C10
C6
C13
O1
C2
C11
C5
C12
C3
C4
Cl1
Z 79
cd1690
P -1
R = 0.04
RES= 0 -70 X

NMR Spectra

Sodium cyclohexanesulfinate (1)

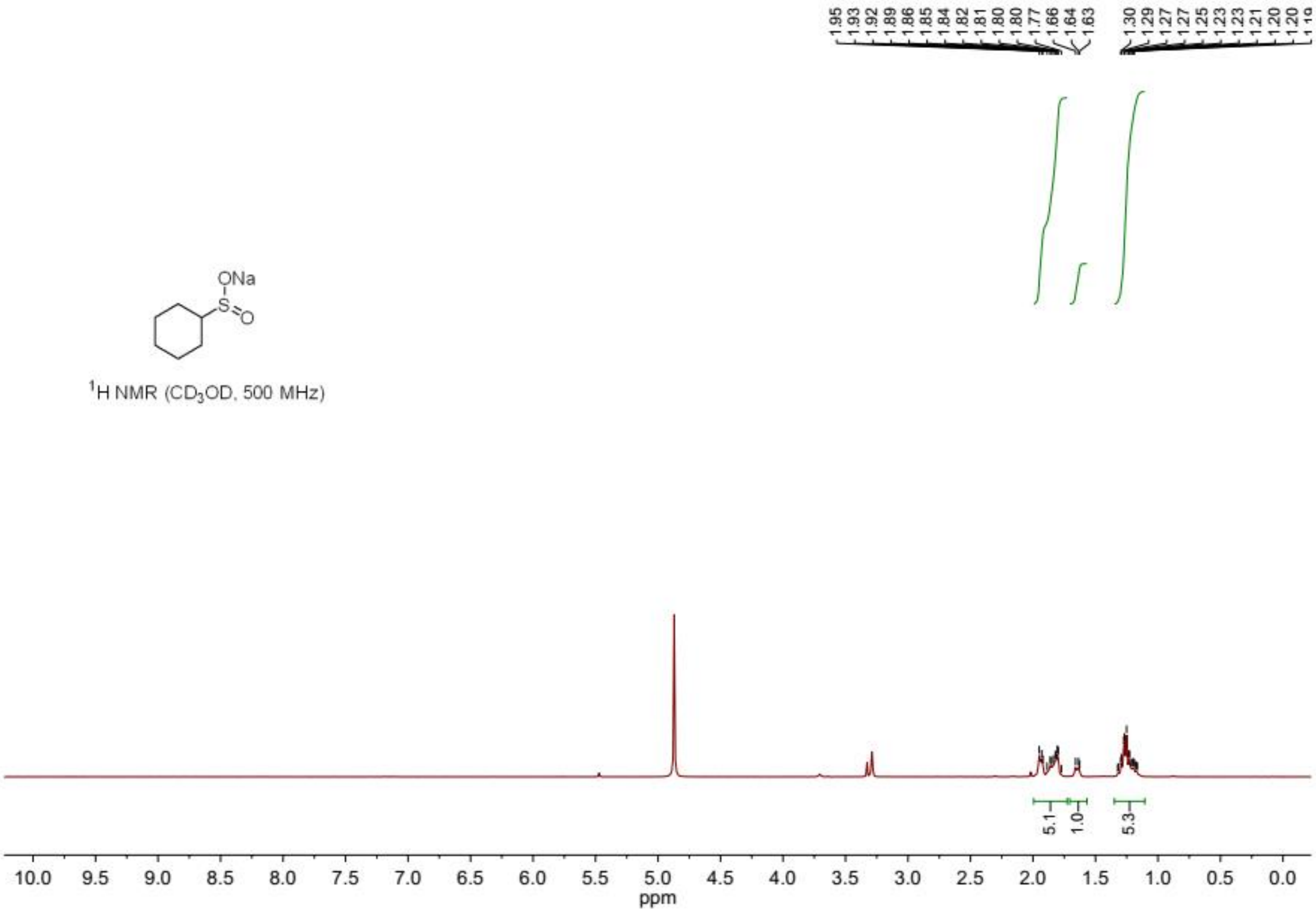

Go back to table of contents

Sodium cyclohexanesulfinate (1)

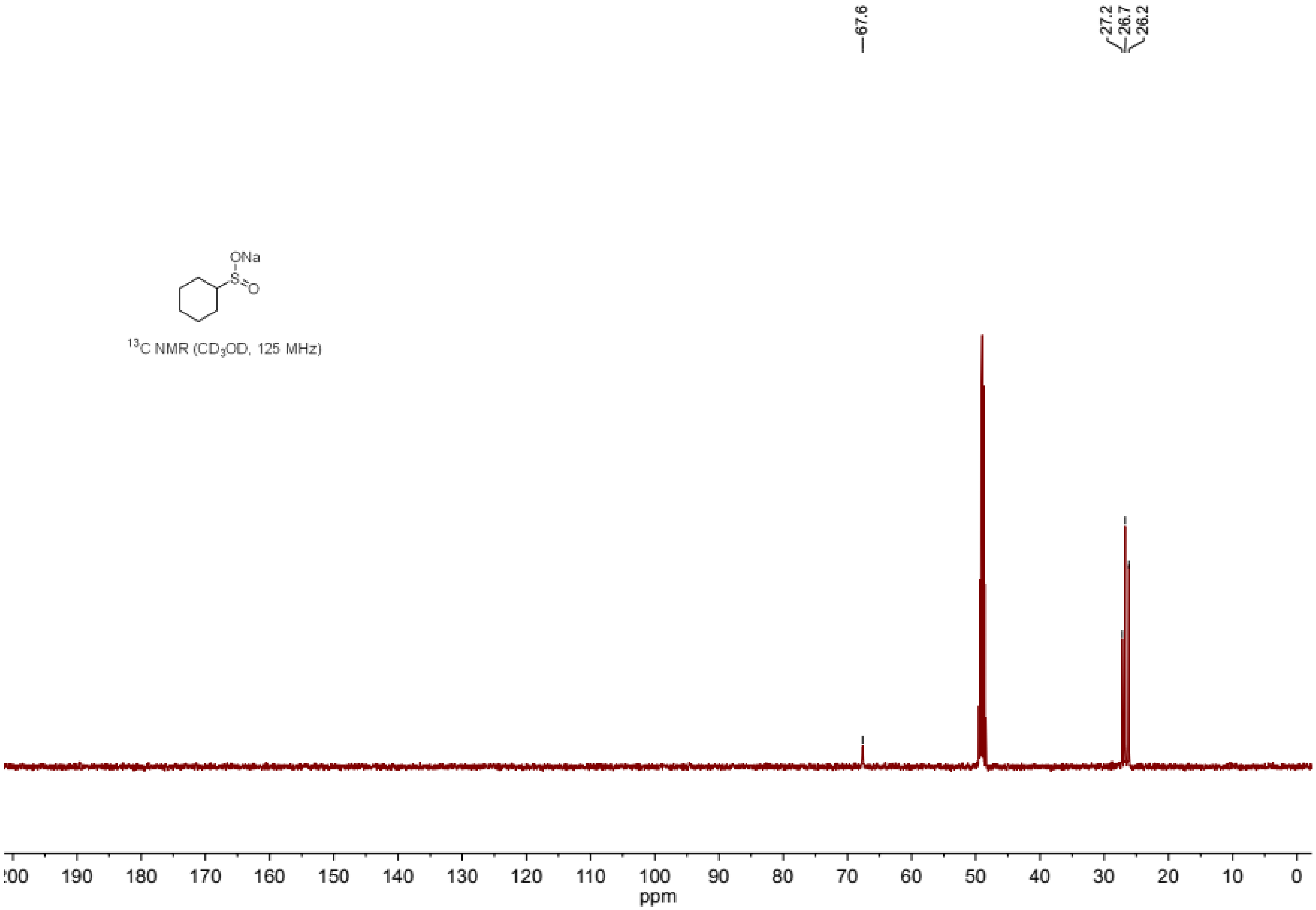

Go back to table of contents

(Methylsulfonyl)cyclopentane (2)

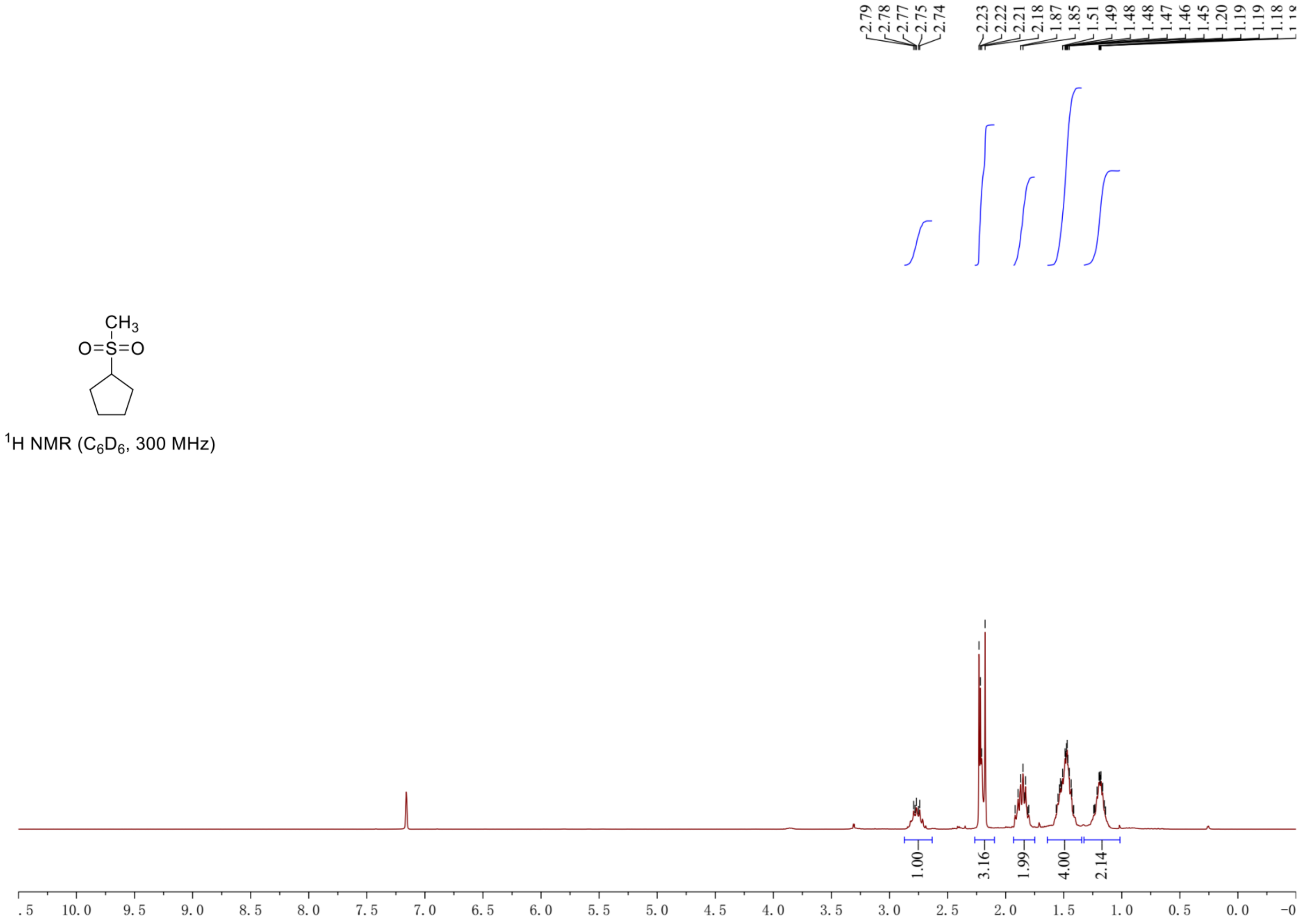

Go back to table of contents

(Methylsulfonyl)cyclopentane (2)

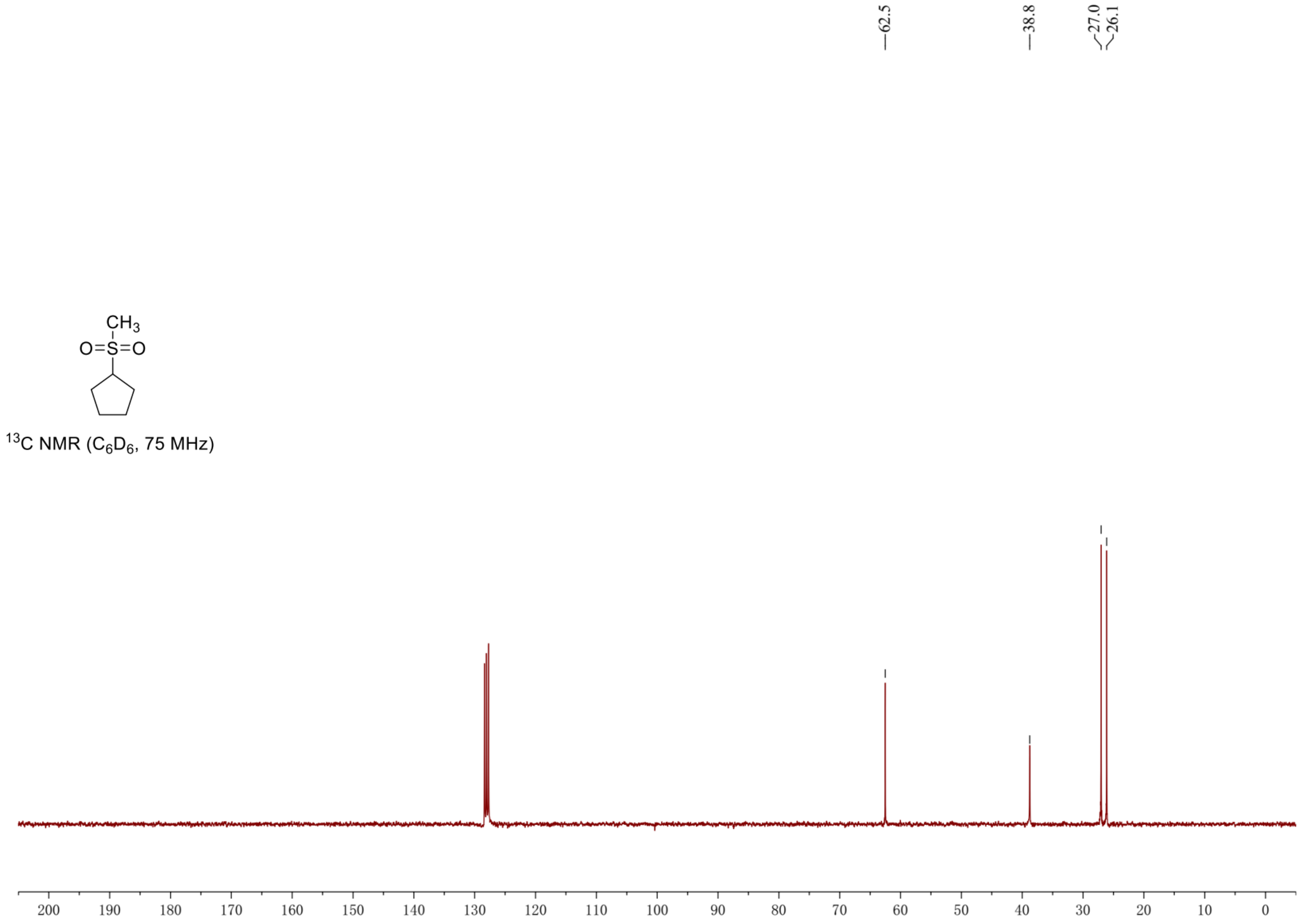

Go back to table of contents

(Methylsulfonyl)cyclohexane (3)

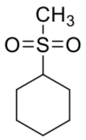

^{1}H NMR ($CDCl_3$, 500 MHz)

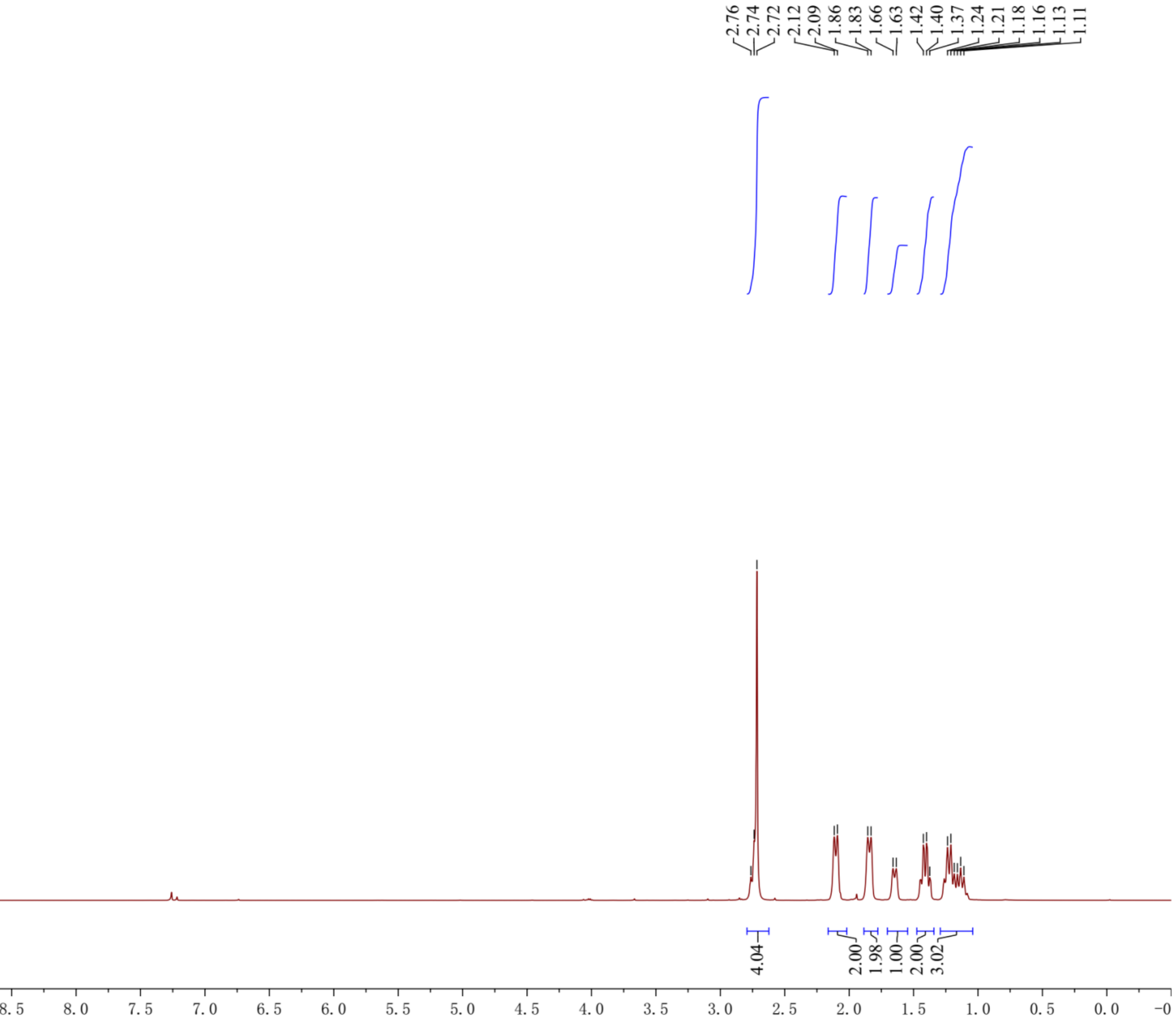

Go back to table of contents

(Methylsulfonyl)cyclohexane (3)

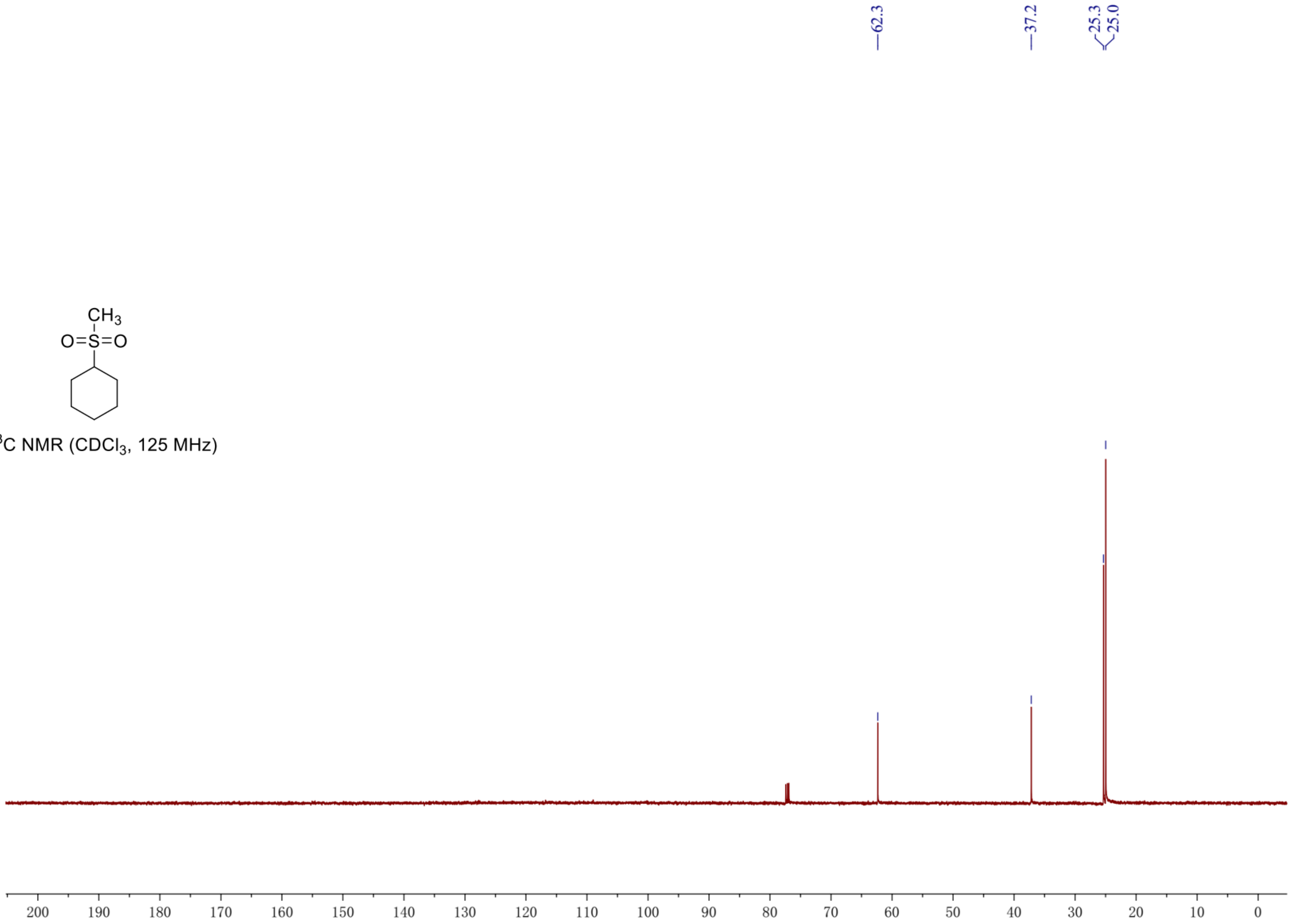

^{13}C NMR ($CDCl_3$, 125 MHz)

Go back to table of contents

(Methylsulfonyl)cycloheptane (4)

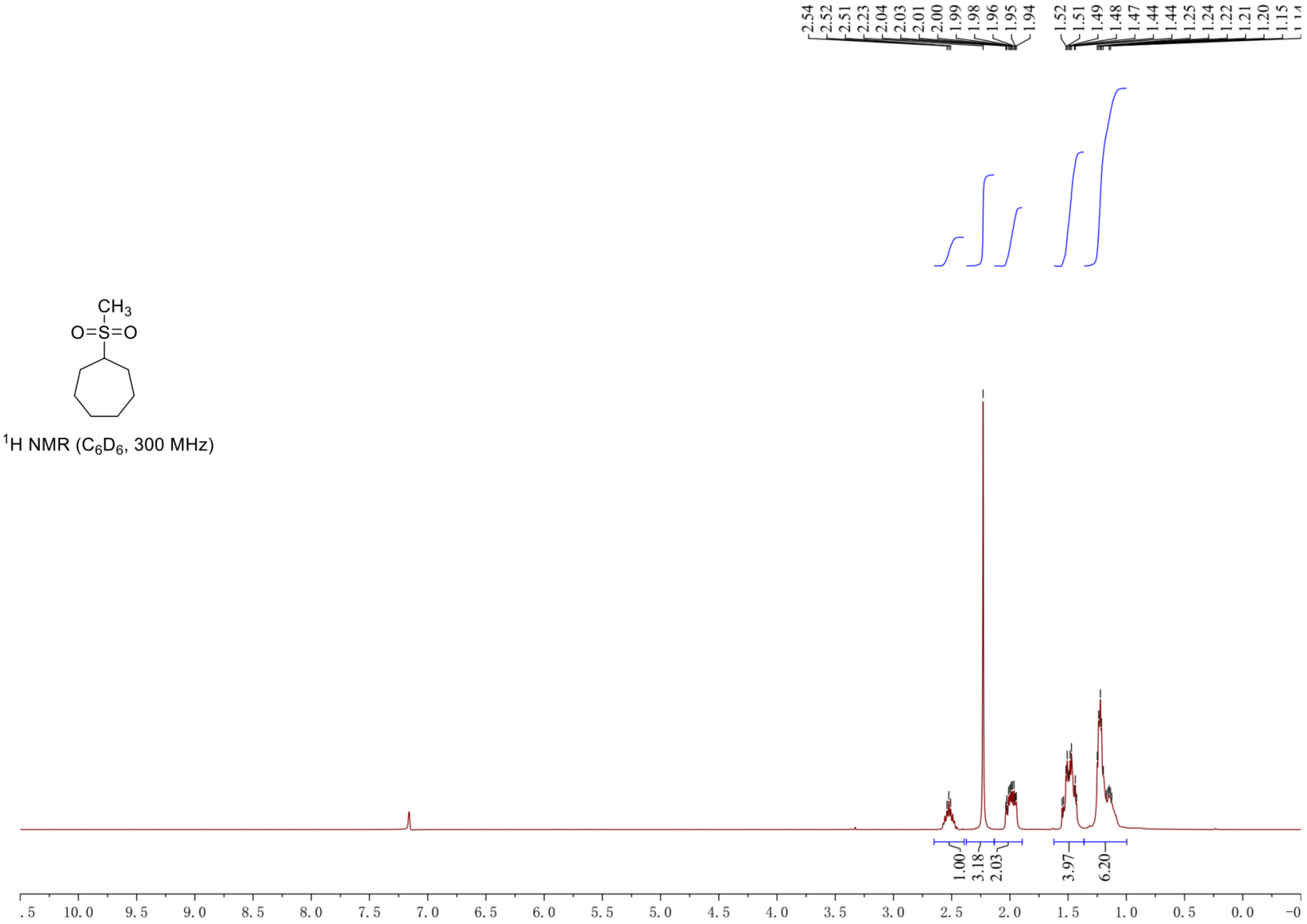

Go back to table of contents

(Methylsulfonyl)cycloheptane (4)

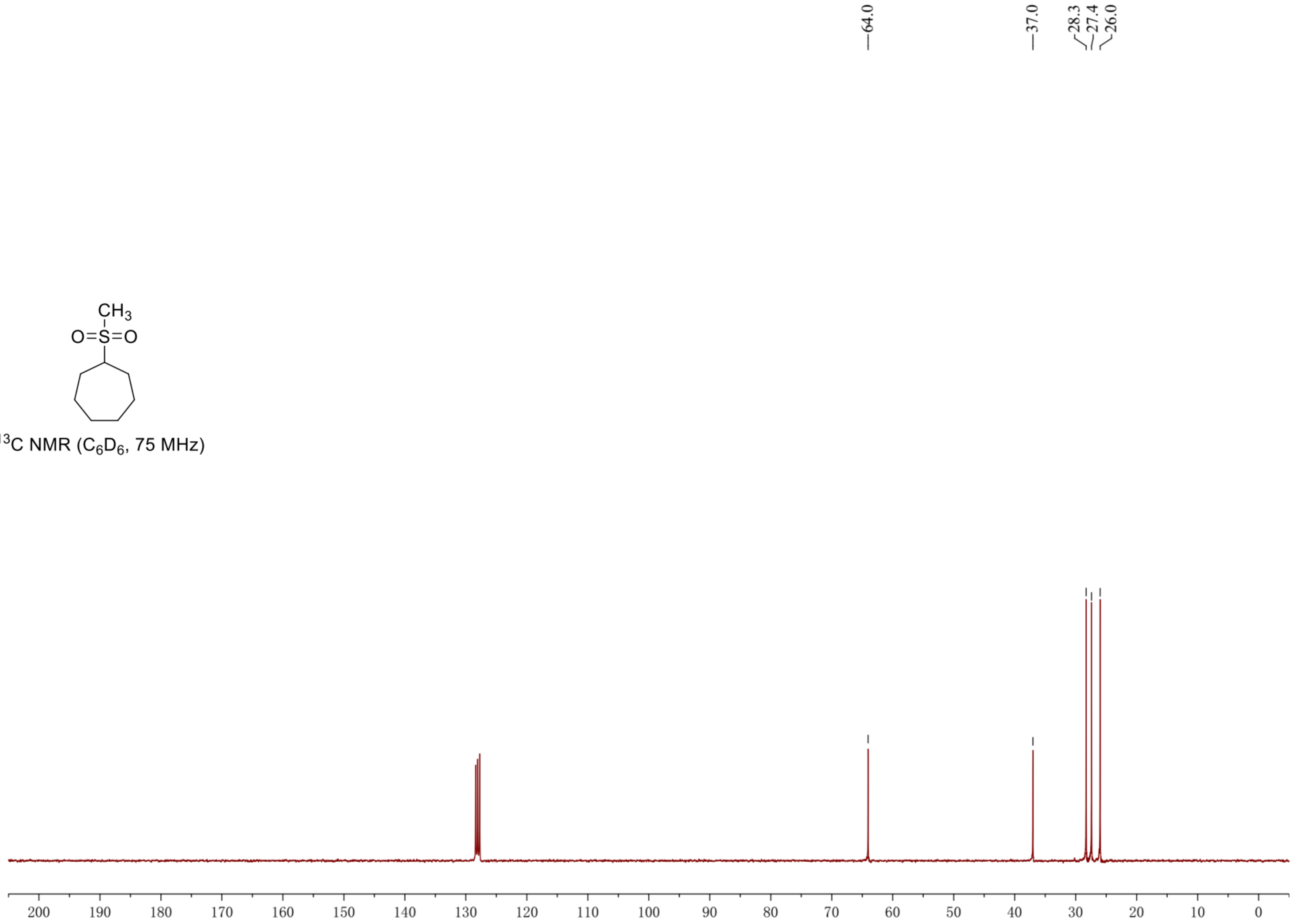

^{13}C NMR (C_6D_6, 75 MHz)

Go back to table of contents

(Methylsulfonyl)cyclooctane (5)

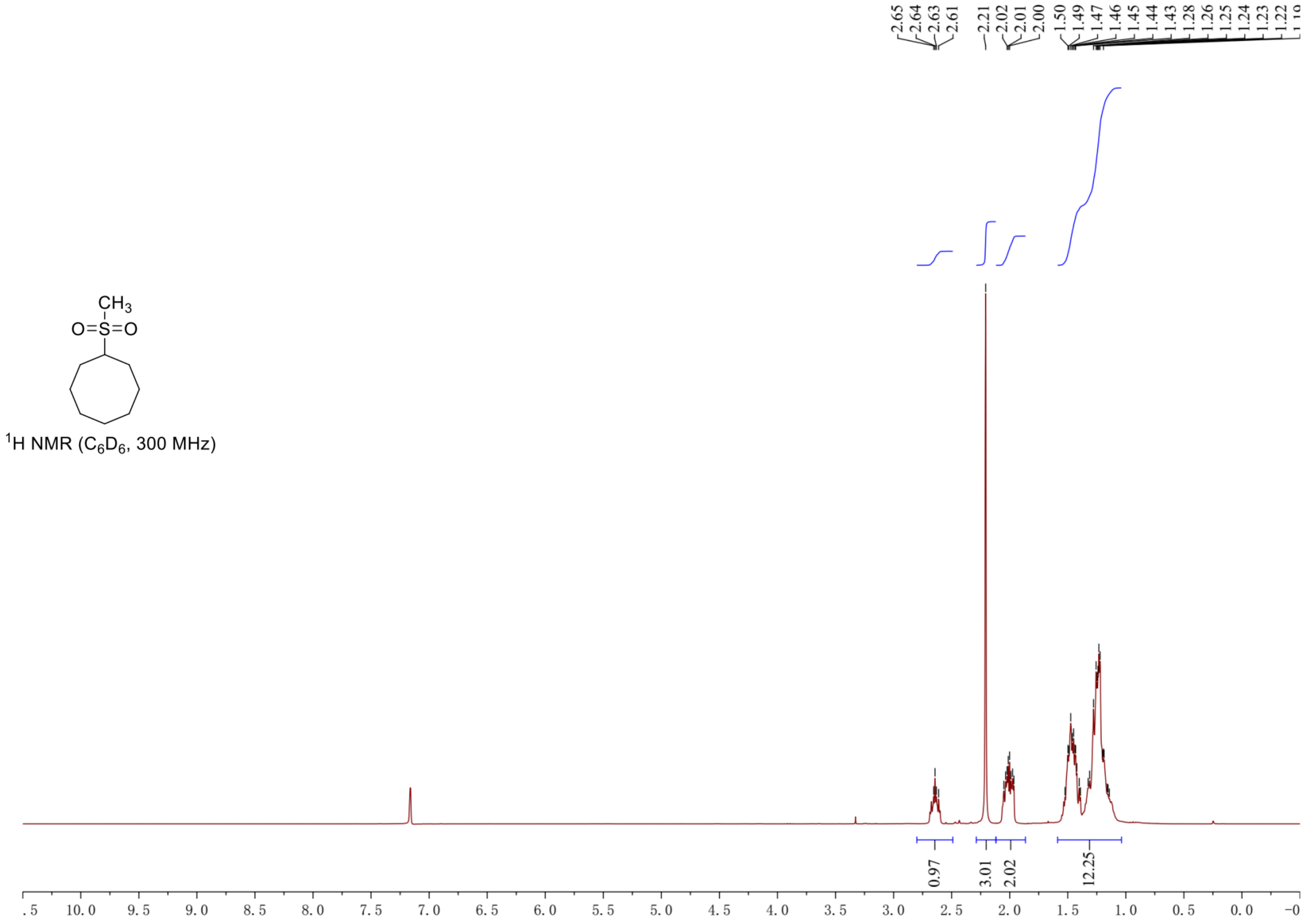

Go back to table of contents

(Methylsulfonyl)cyclooctane (5)

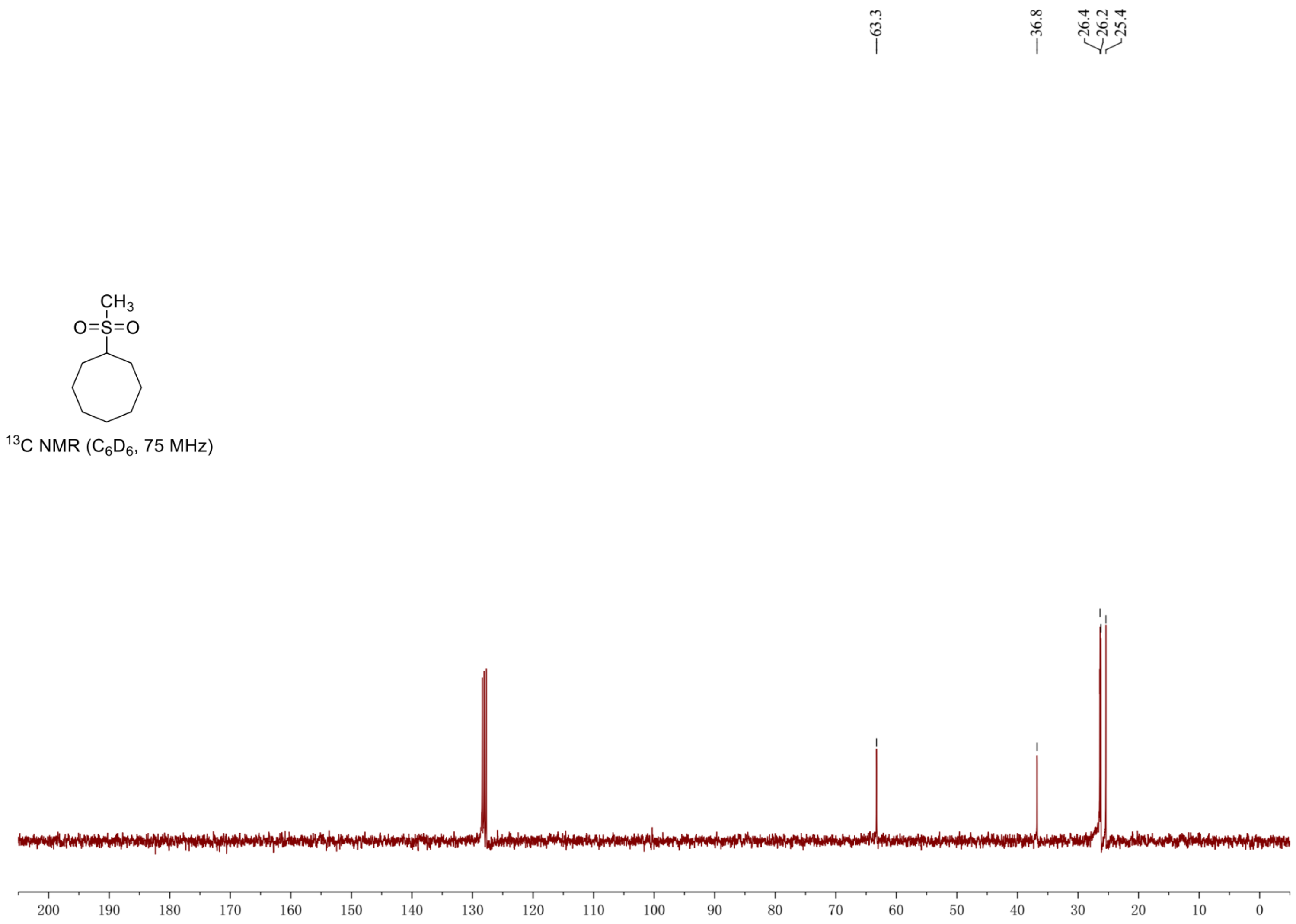

Go back to table of contents

1-(Methylsulfonyl)pentane (6a)

2-(Methylsulfonyl)pentane (6b)
3-(Methylsulfonyl)pentane (6c)

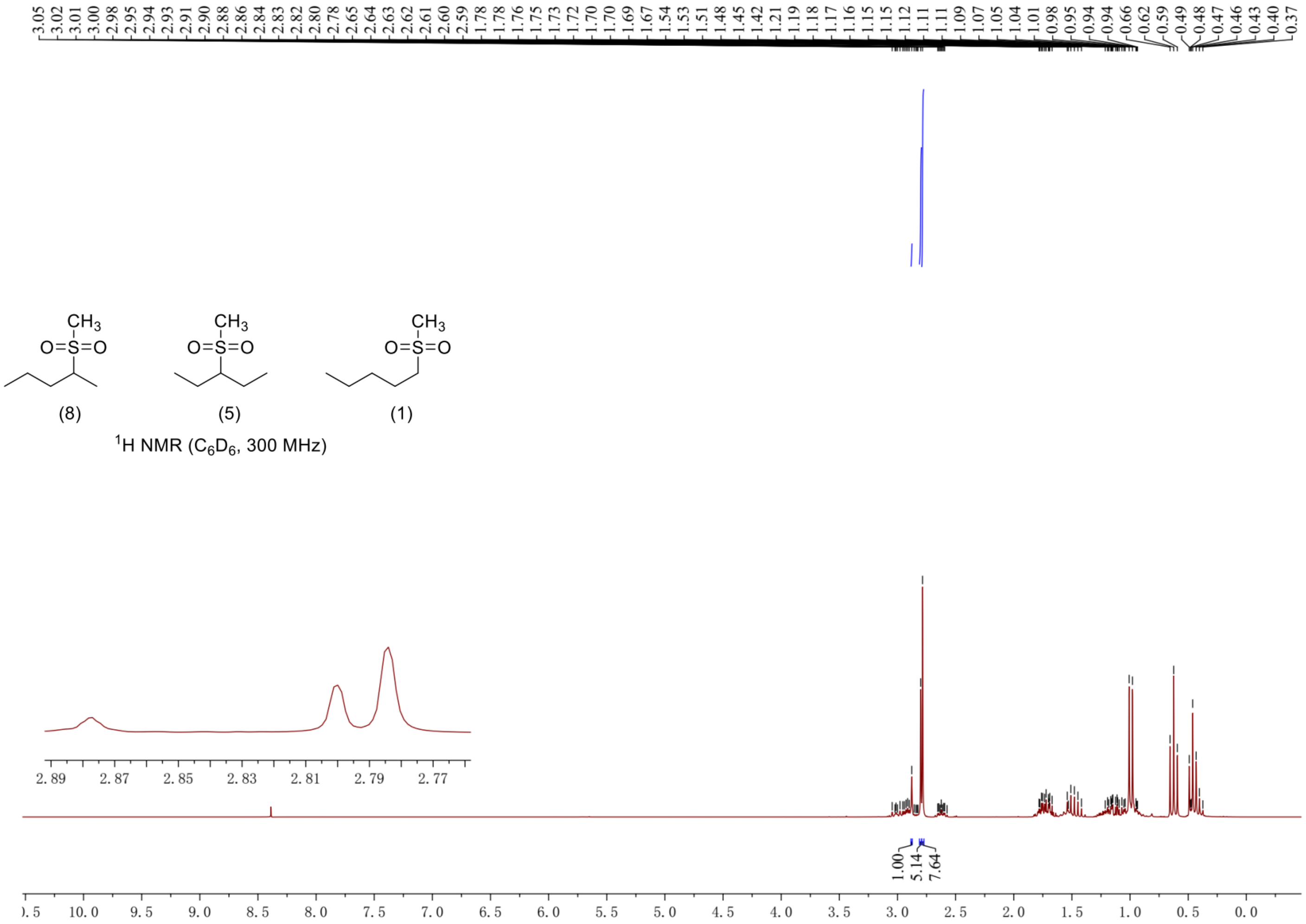

Go back to table of contents

1-(Methylsulfonyl)pentane (6a)

2-(Methylsulfonyl)pentane (6b)

3-(Methylsulfonyl)pentane (6c)

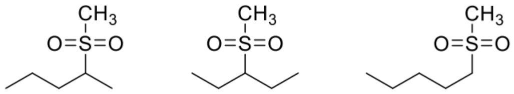

^{13}C NMR (C_6D_6, 75 MHz)

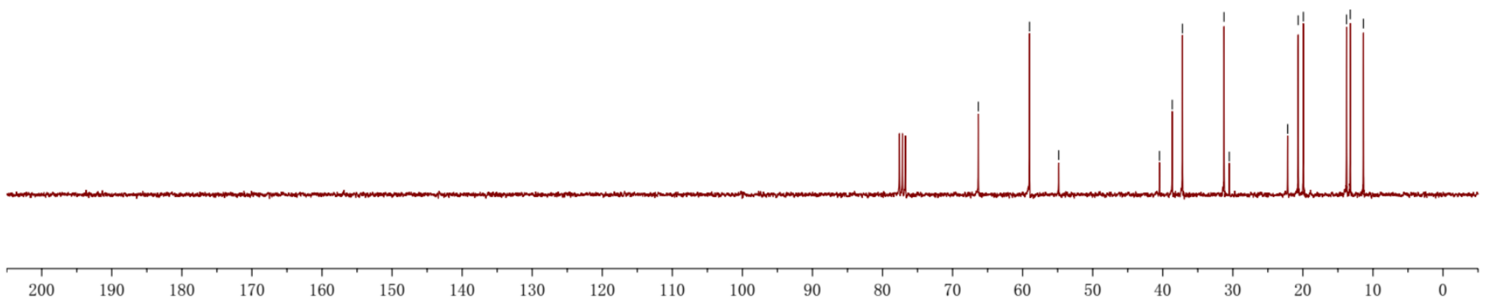

Go back to table of contents

2,3-Dimethyl-1-(methylsulfonyl)butane (7a)

2,3-Dimethyl-2-(methylsulfonyl)butane (7b)

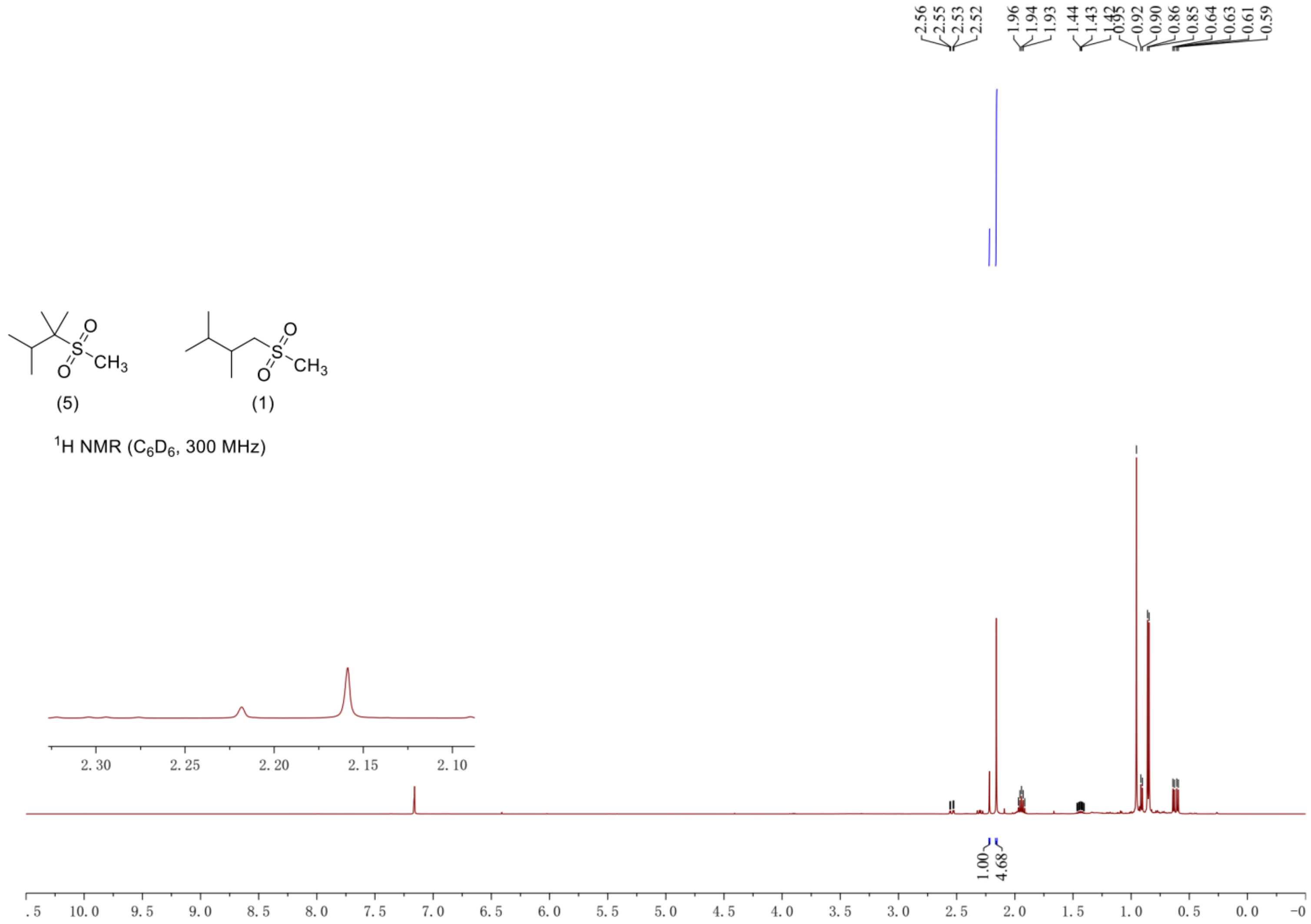

Go back to table of contents

2,3-Dimethyl-1-(methylsulfonyl)butane (7a)

2,3-Dimethyl-2-(methylsulfonyl)butane (7b)

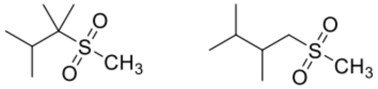

^{13}C NMR (C_6D_6, 75 MHz)

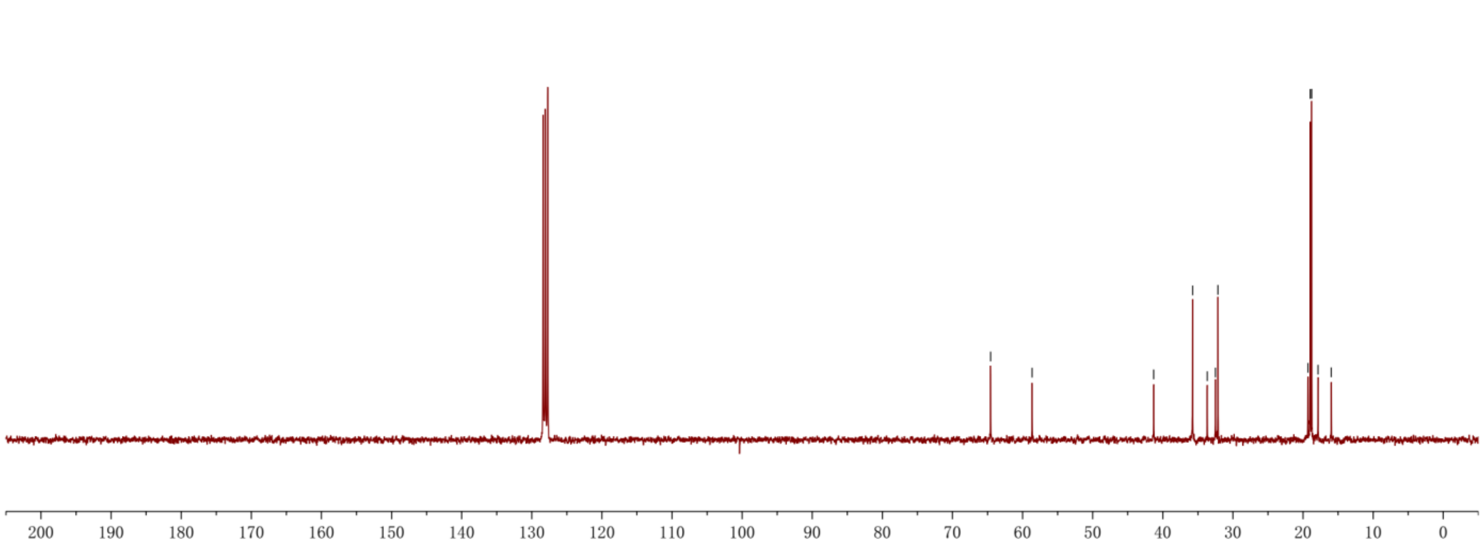

Go back to table of contents

3,3-Dimethyl-4-(methylsulfonyl)butan-2-one (8)

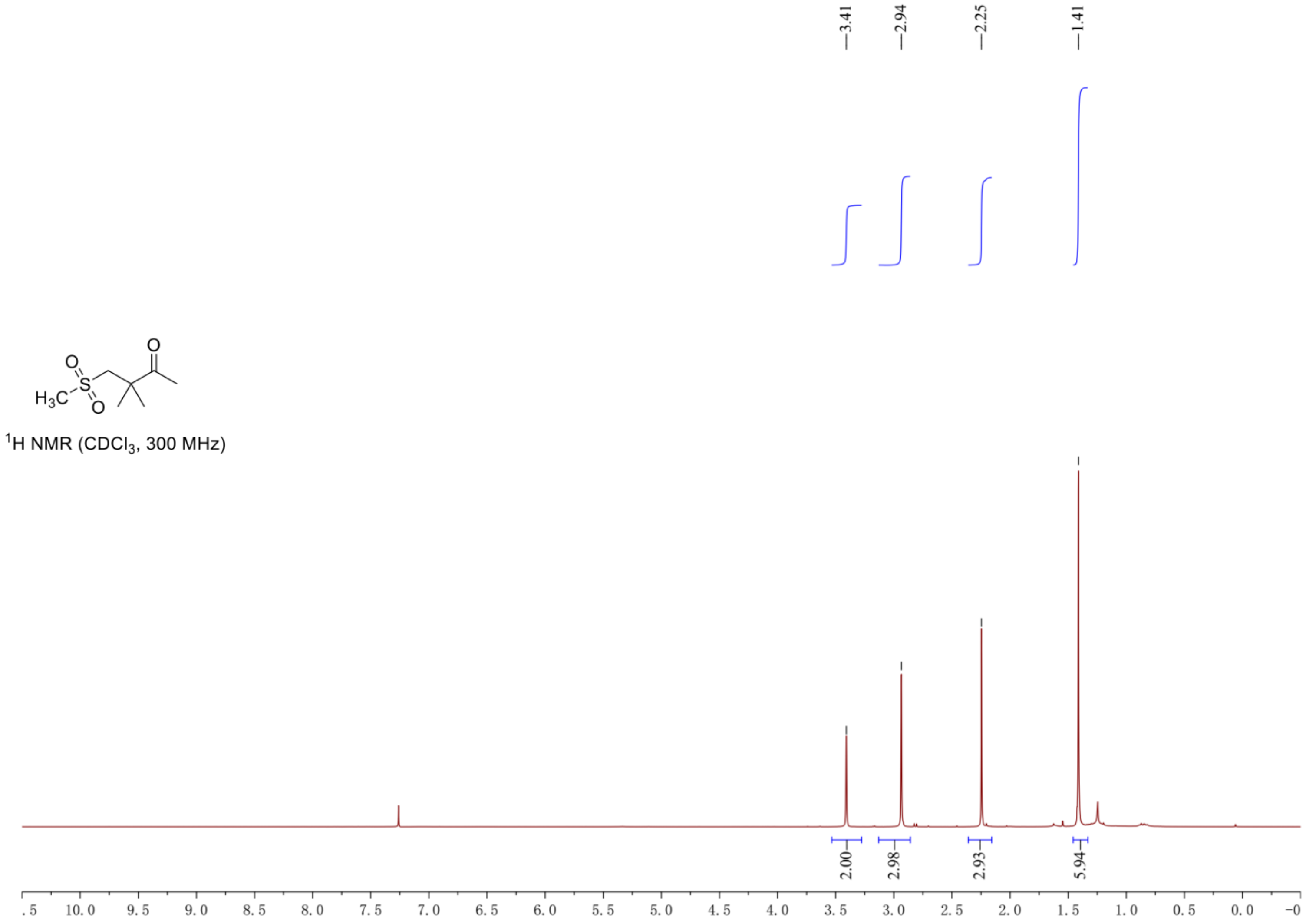

Go back to table of contents

3,3-Dimethyl-4-(methylsulfonyl)butan-2-one (8)

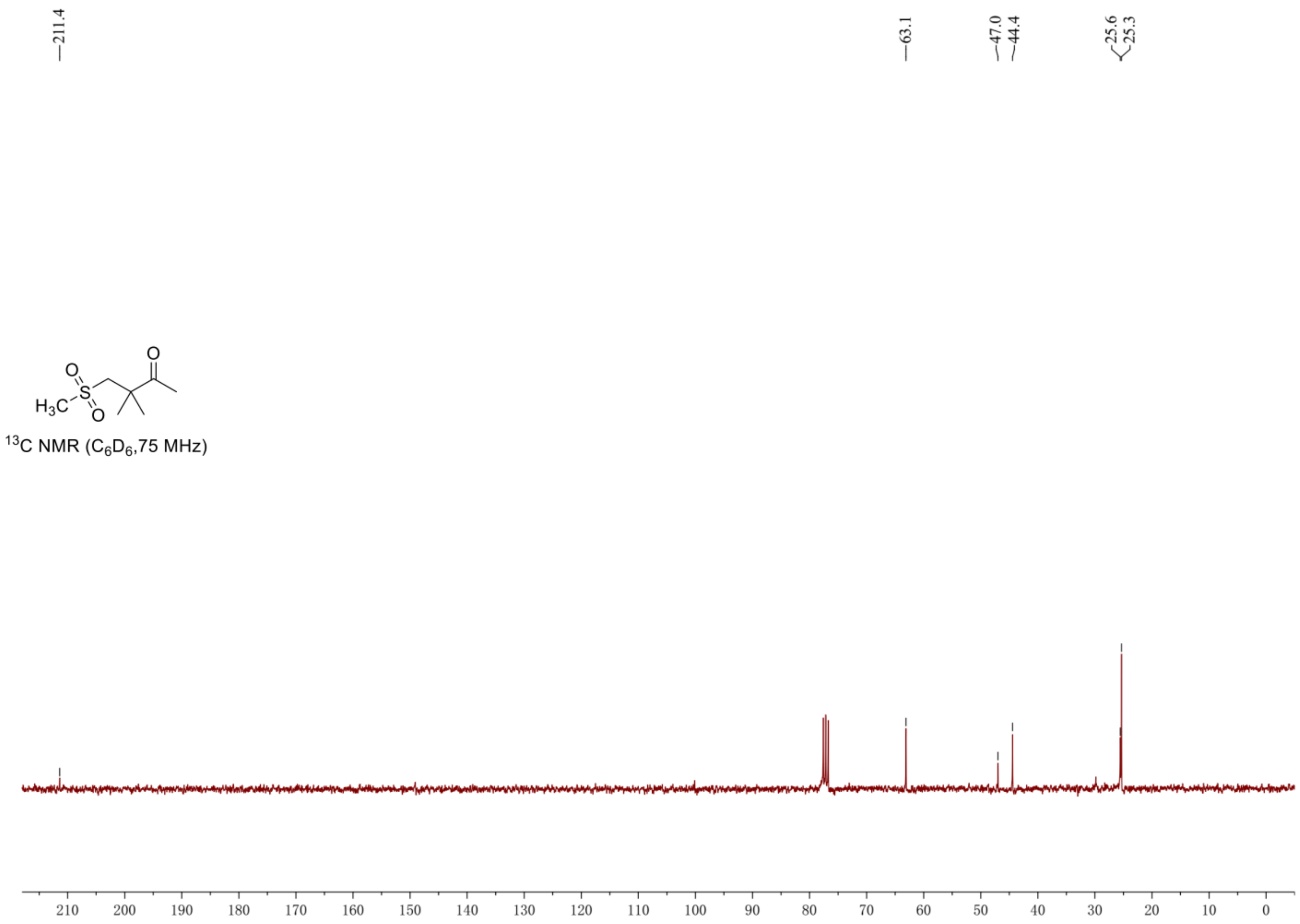

Go back to table of contents

1-(Allylsulfonyl)pentan-3-one (9)

^{1}H NMR ($CDCl_3$, 500 MHz)

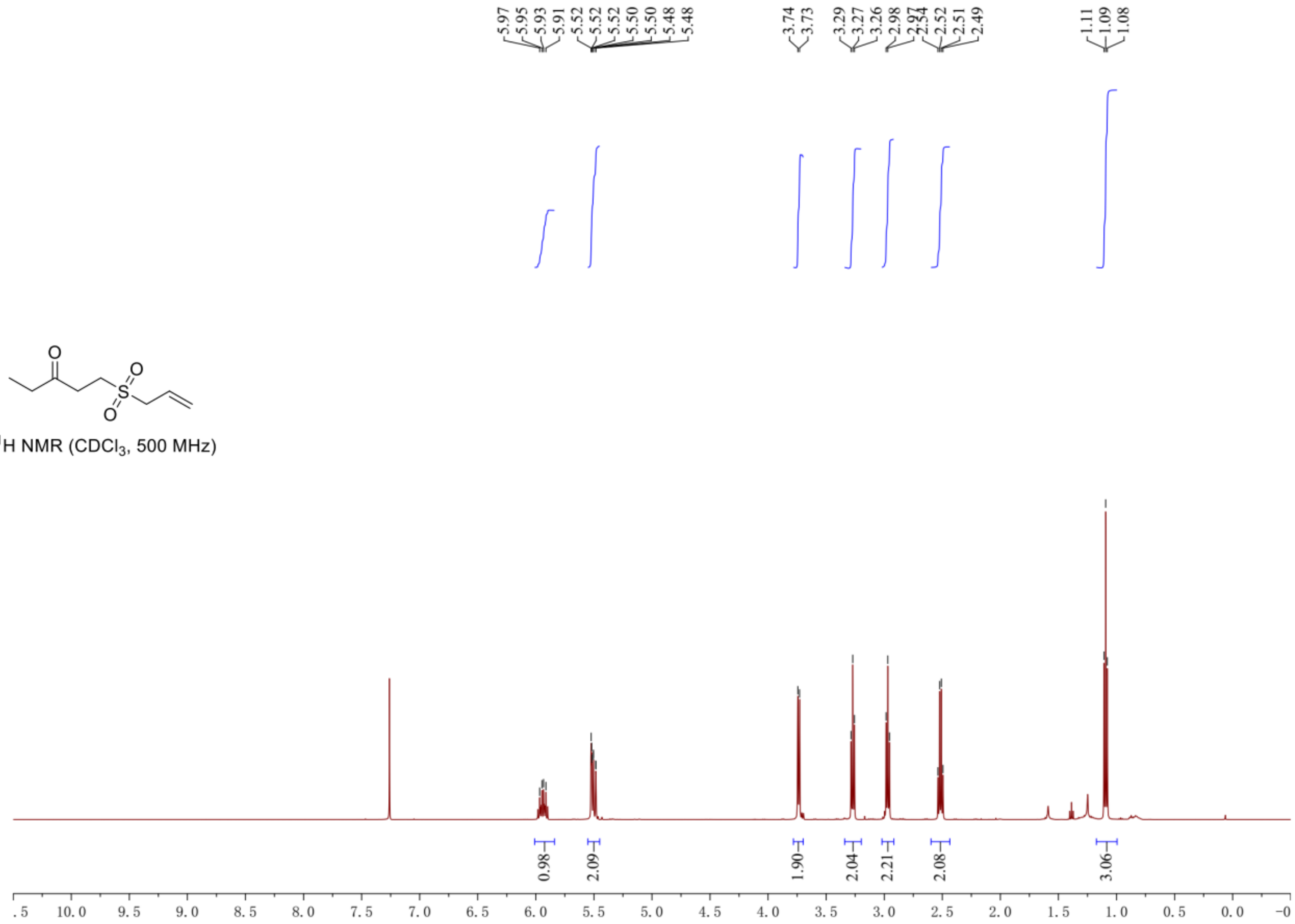

Go back to table of contents

1-(Allylsulfonyl)pentan-3-one (9)

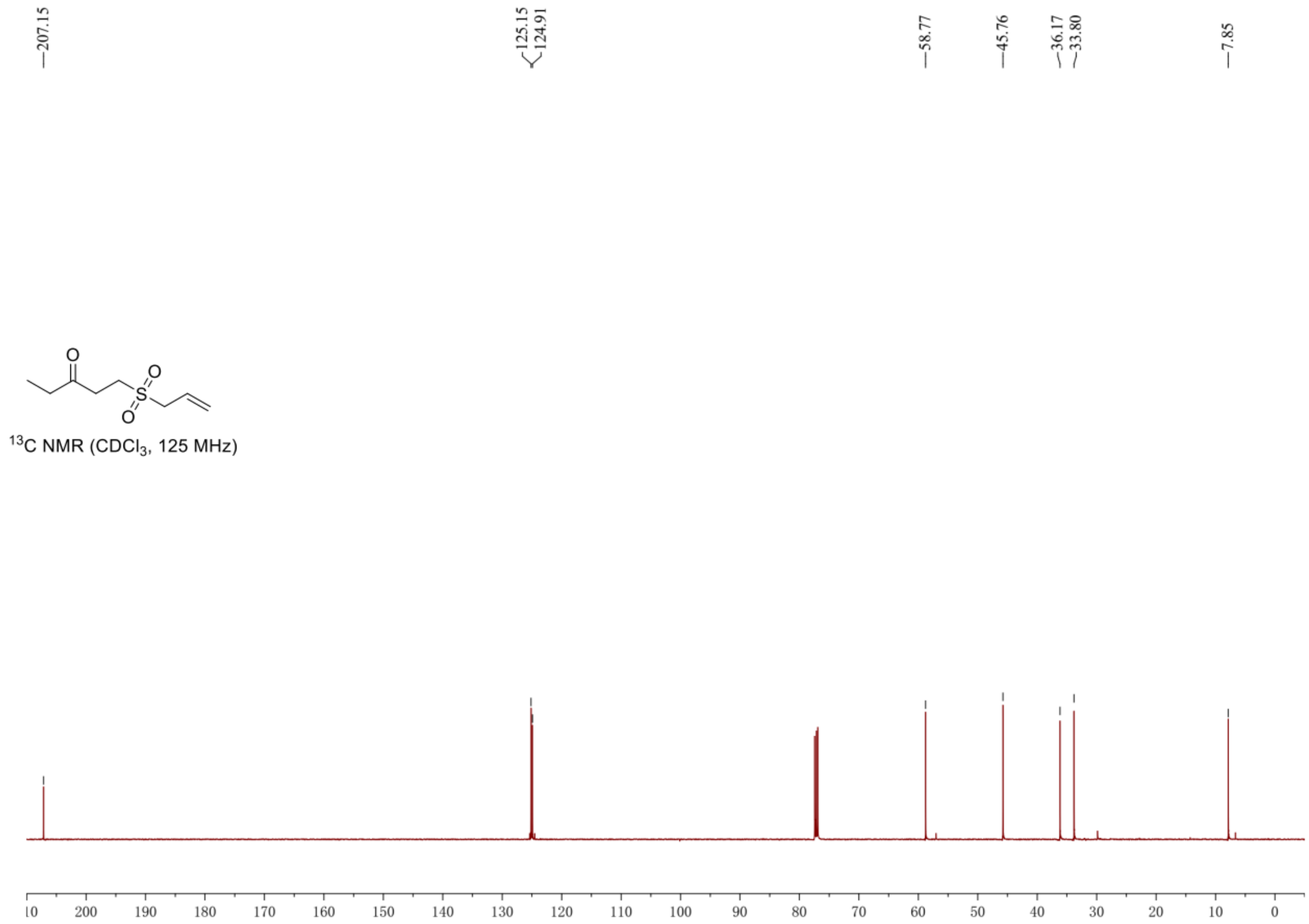

Go back to table of contents

5-(Allylsulfonyl)pentan-2-one (10a)

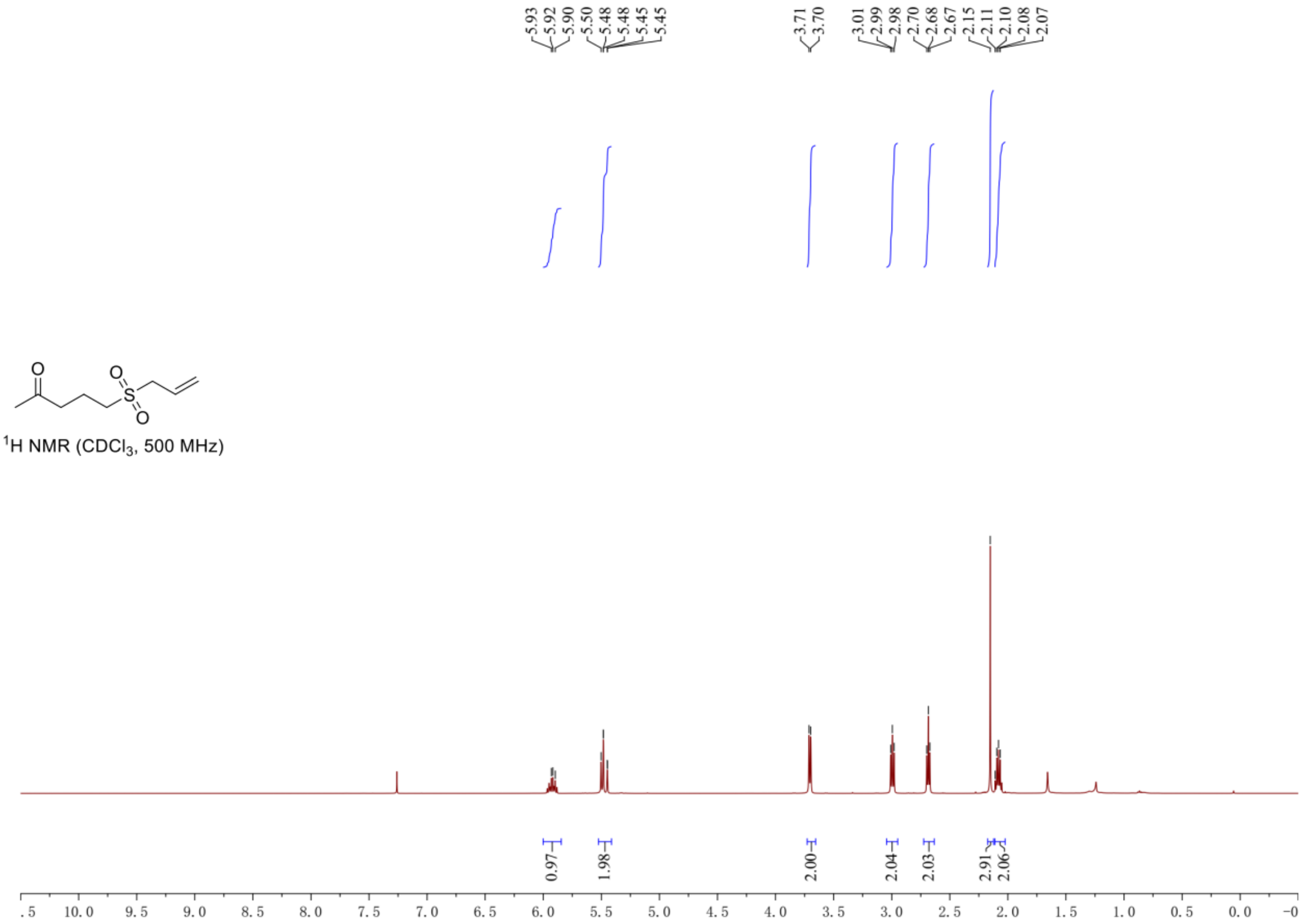

Go back to table of contents

5-(Allylsulfonyl)pentan-2-one (10a)

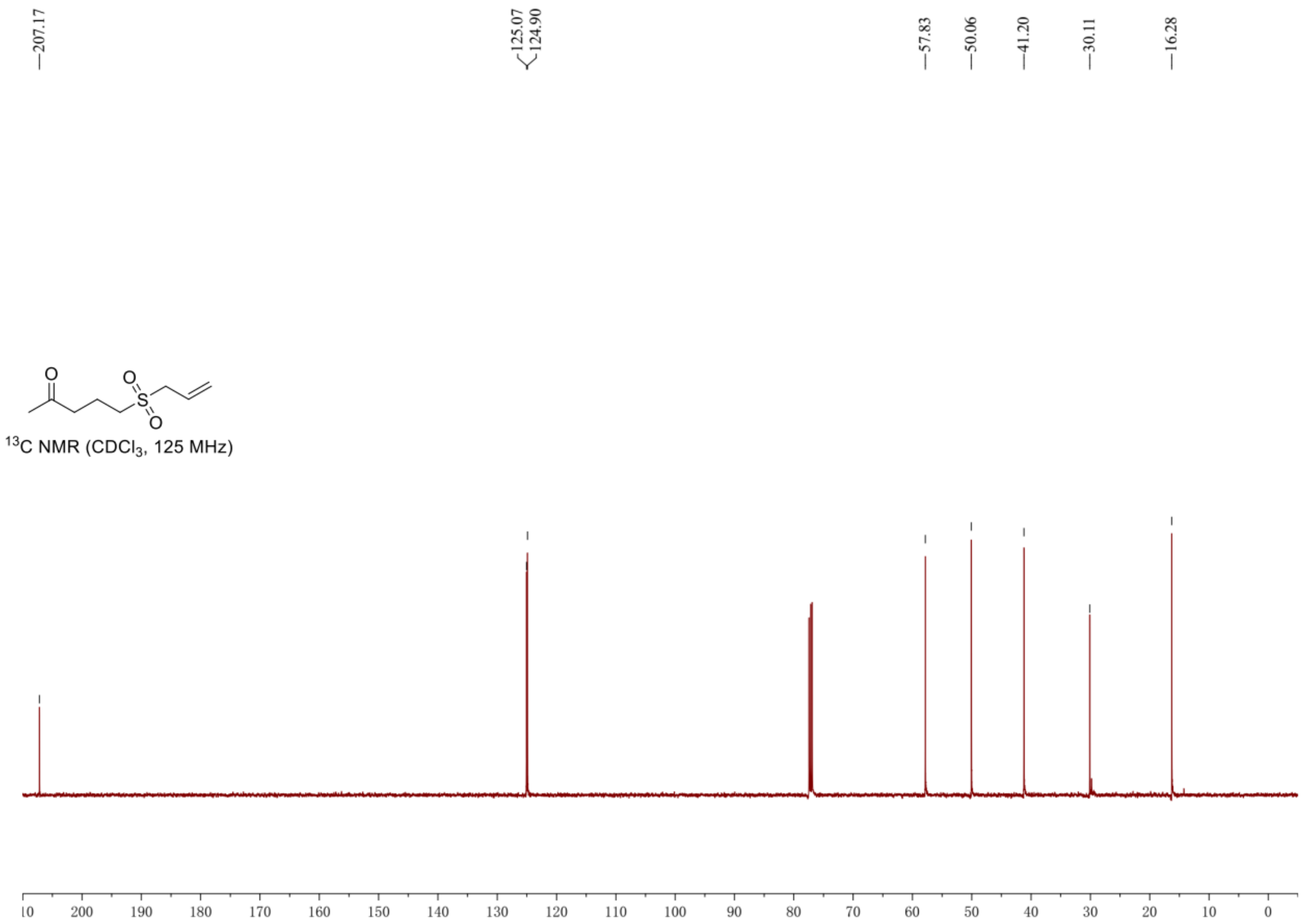

Go back to table of contents

4-(Allylsulfonyl)pentan-2-one (10b)

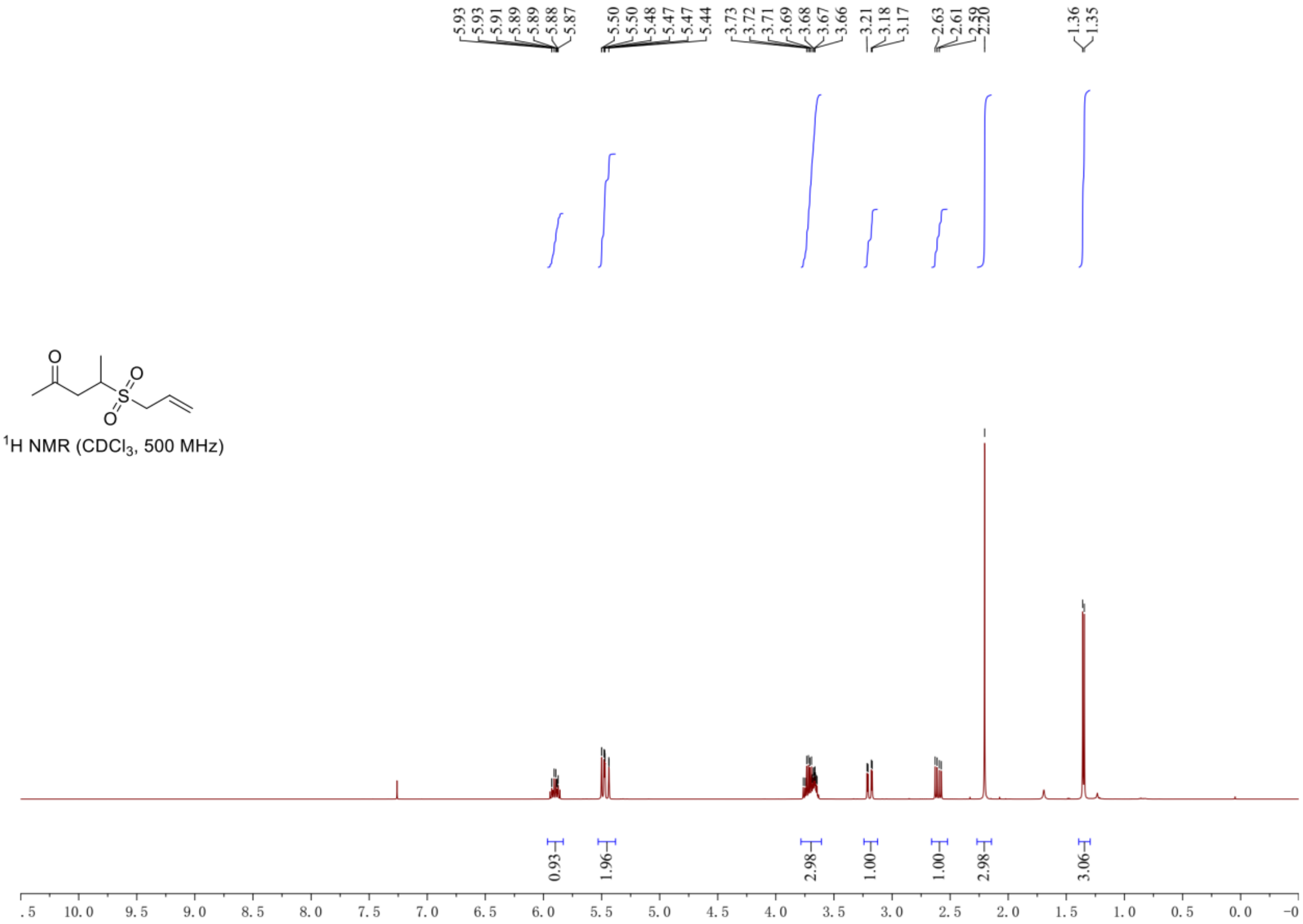

Go back to table of contents

4-(Allylsulfonyl)pentan-2-one (10b)

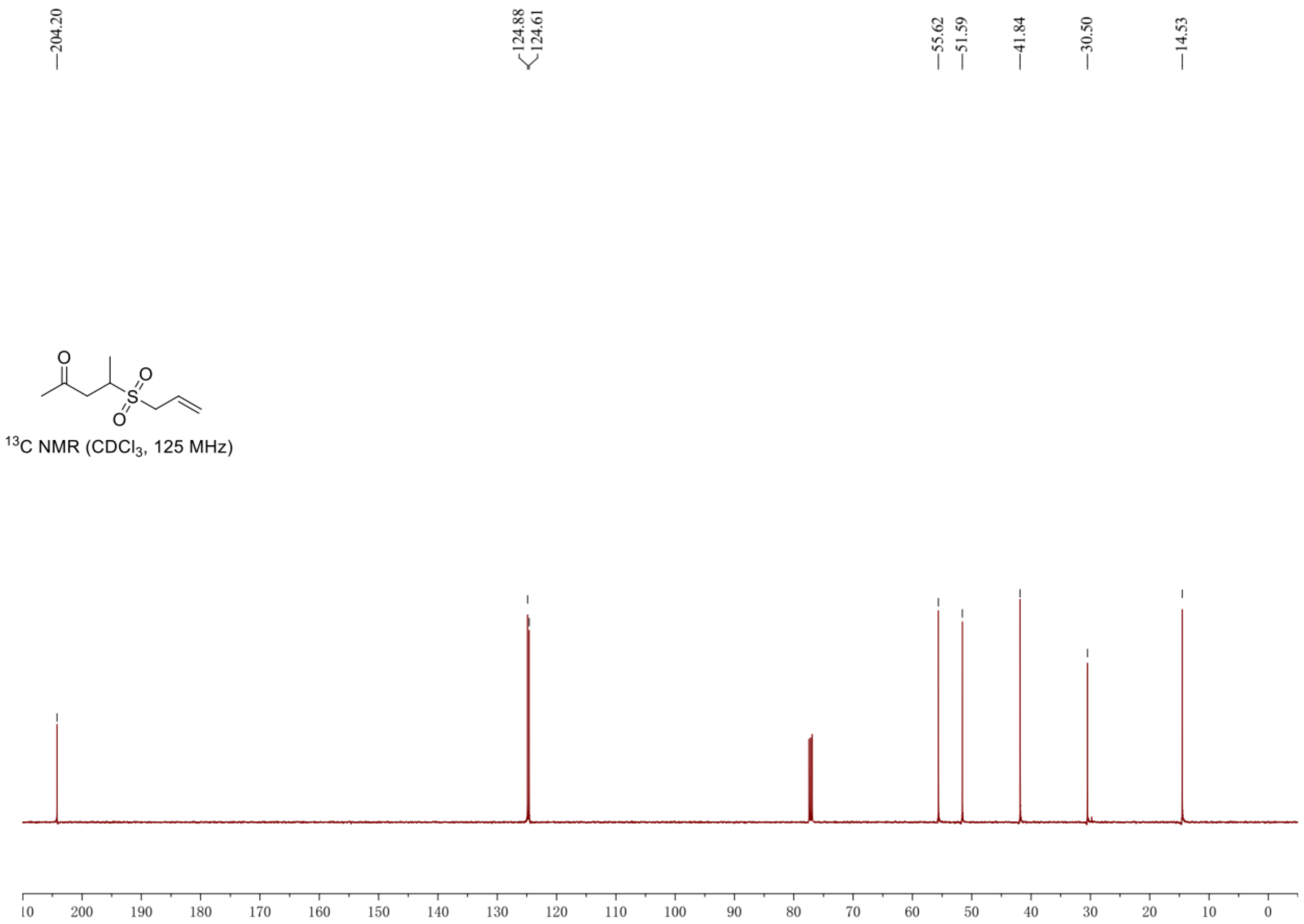

Go back to table of contents

6-(Allylsulfonyl)hexan-2-one (11a)

^1H NMR ($CDCl_3$, 500 MHz)

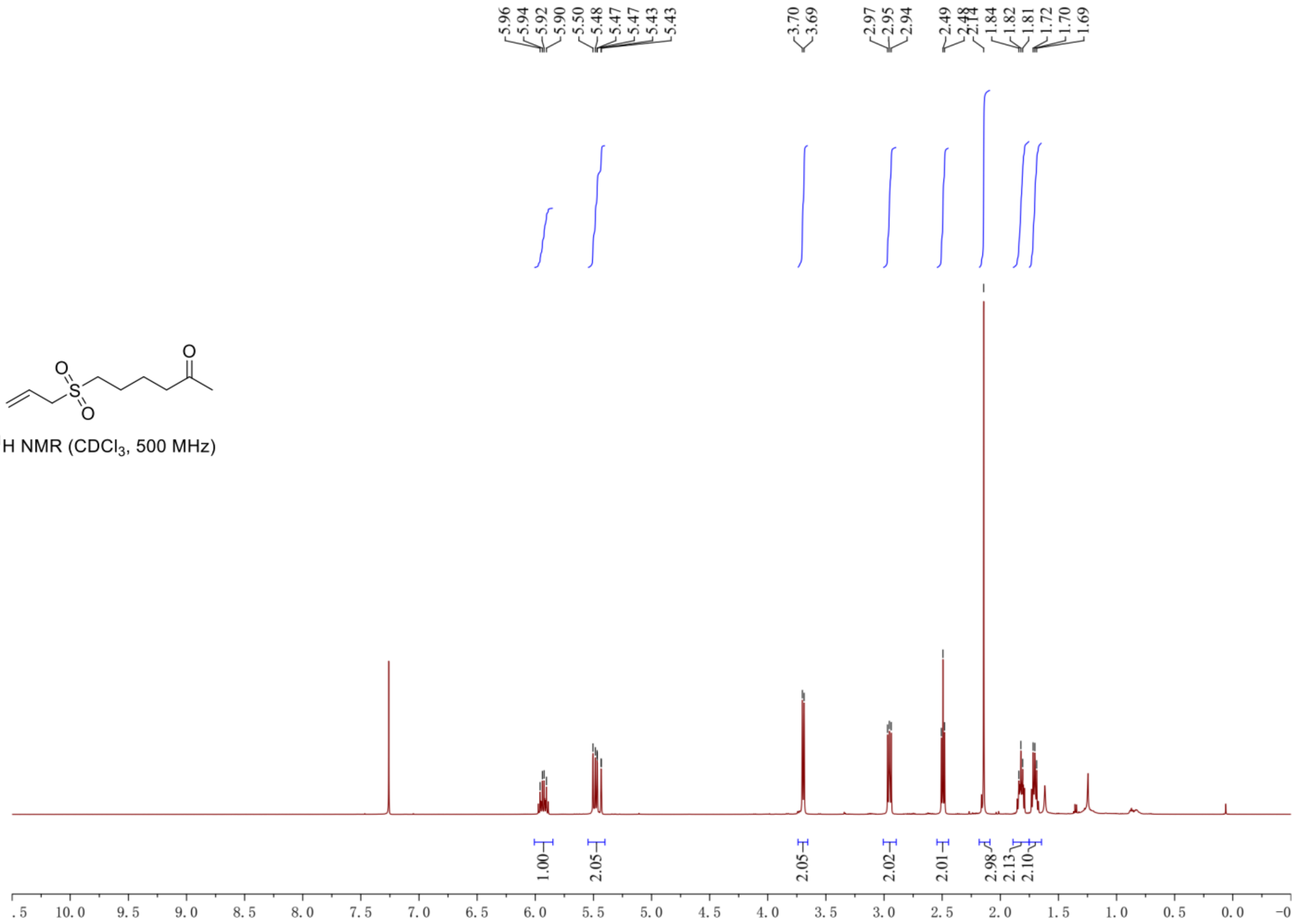

Go back to table of contents

6-(Allylsulfonyl)hexan-2-one (11a)

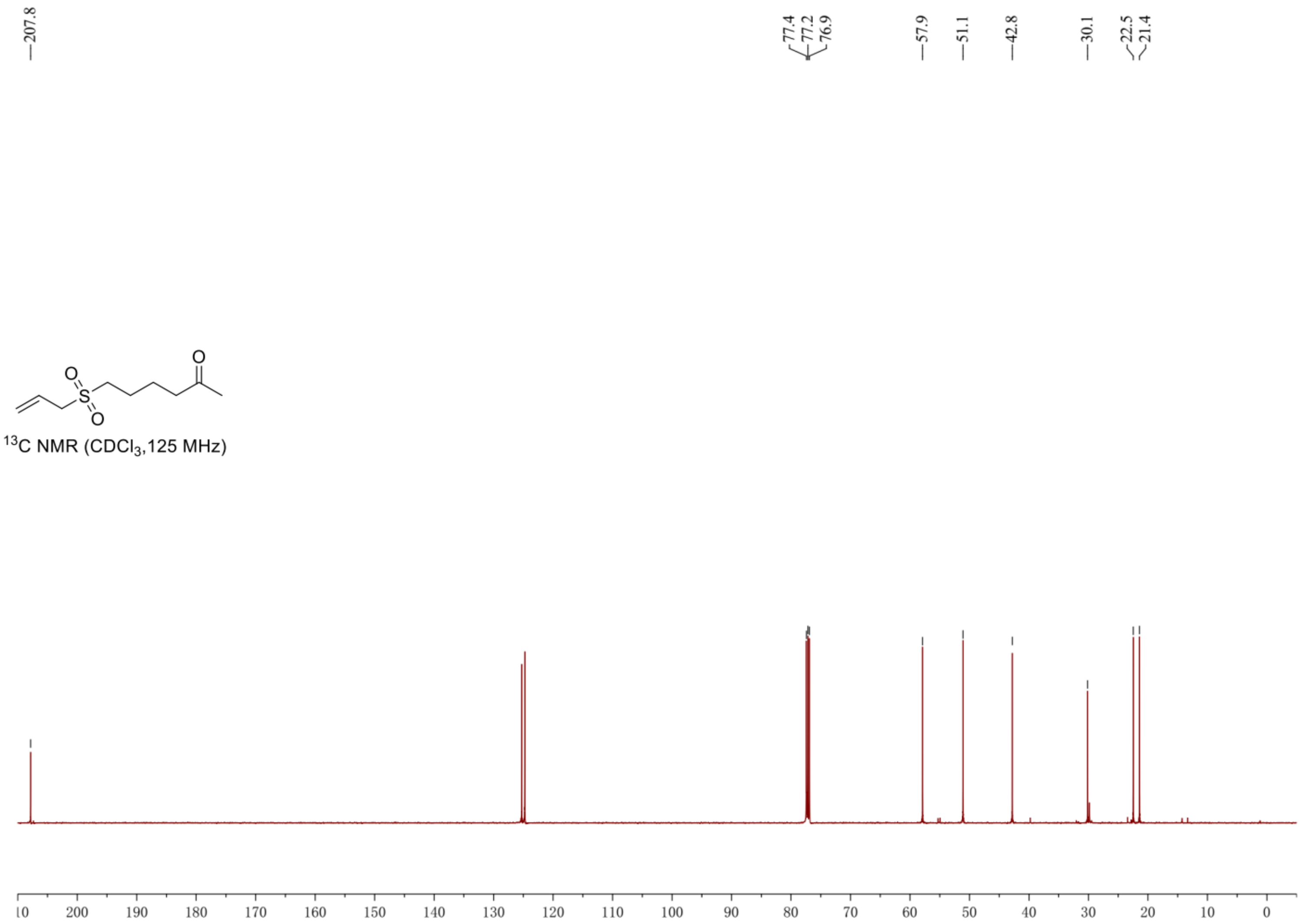

Go back to table of contents

5-(Allylsulfonyl)hexan-2-one (11b)

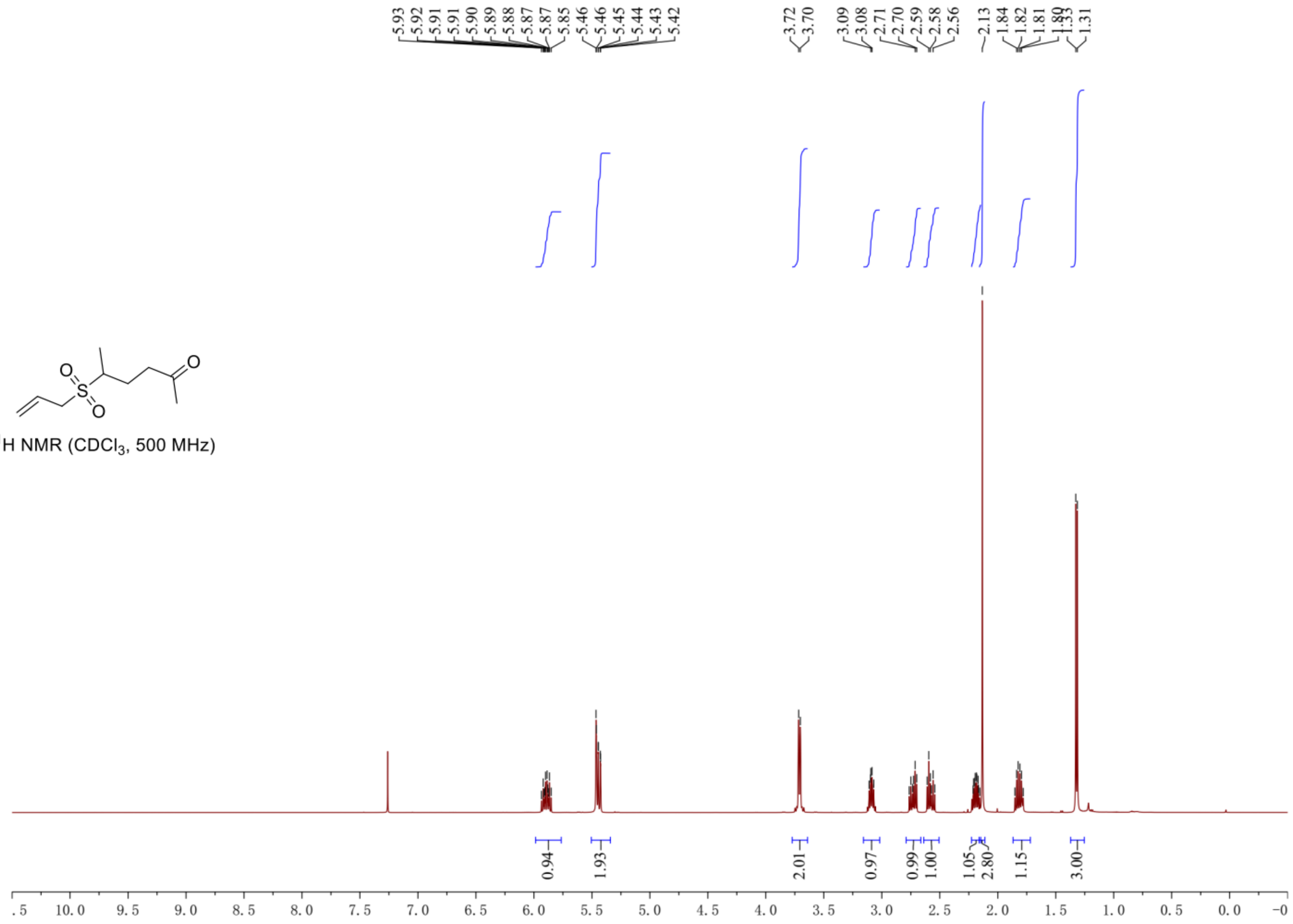

^{1}H NMR ($CDCl_3$, 500 MHz)

Go back to table of contents

5-(Allylsulfonyl)hexan-2-one (11b)

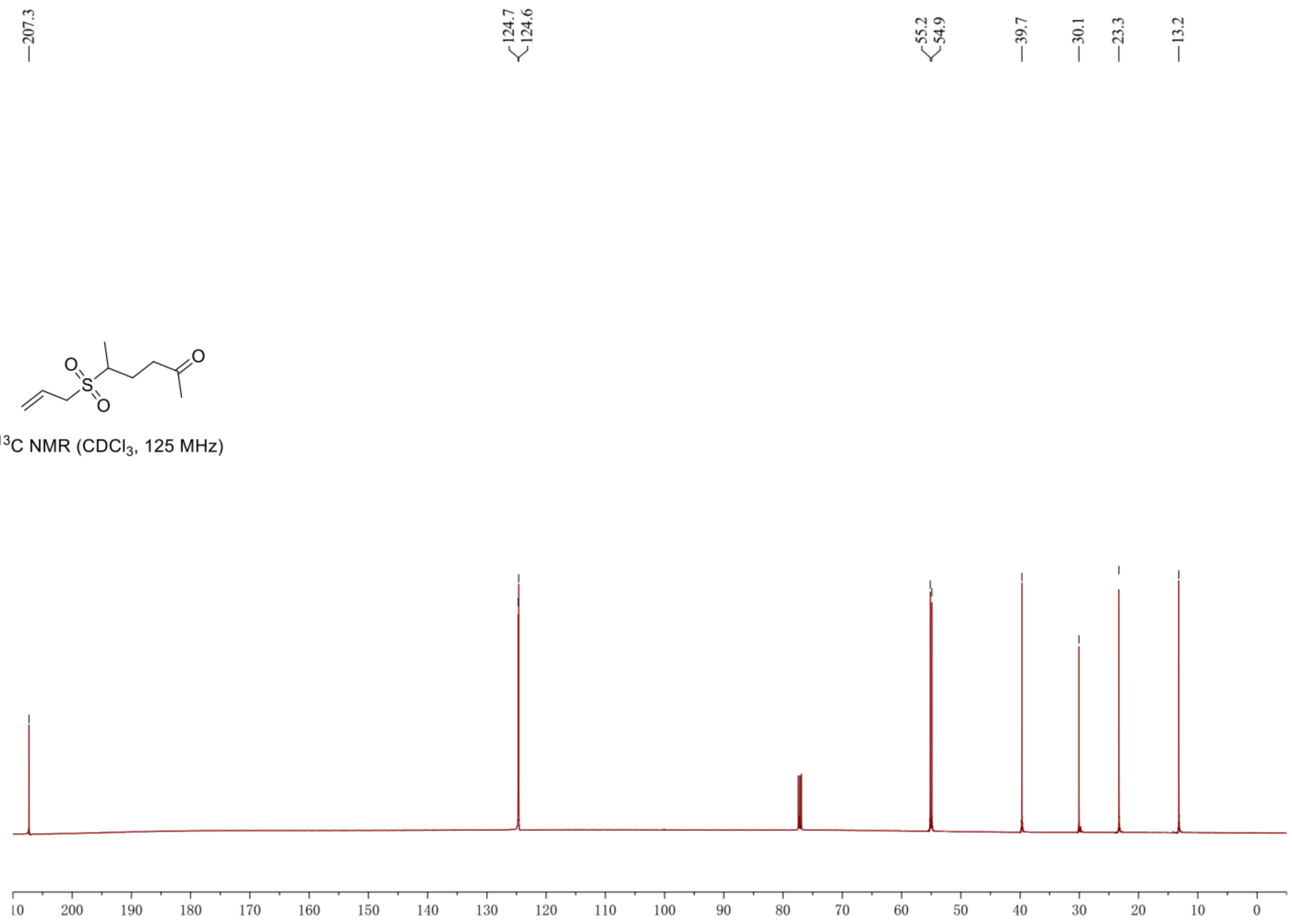

^{13}C NMR ($CDCl_3$, 125 MHz)

Go back to table of contents

4-(Allylsulfonyl)hexan-2-one (11c)

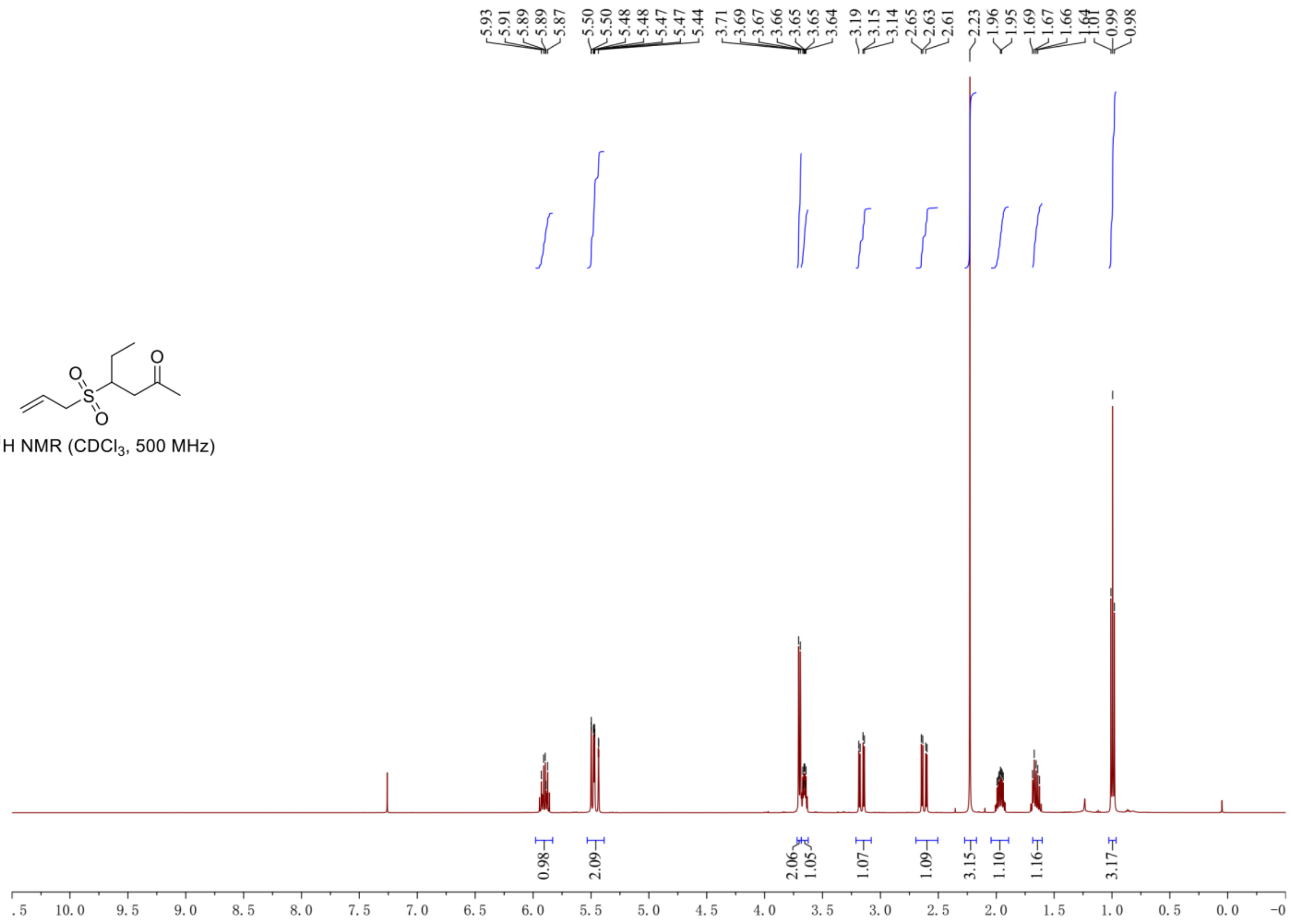

^{1}H NMR ($CDCl_3$, 500 MHz)

4-(Allylsulfonyl)hexan-2-one (11c)

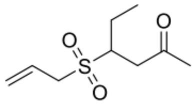

^{13}C NMR ($CDCl_3$, 125 MHz)

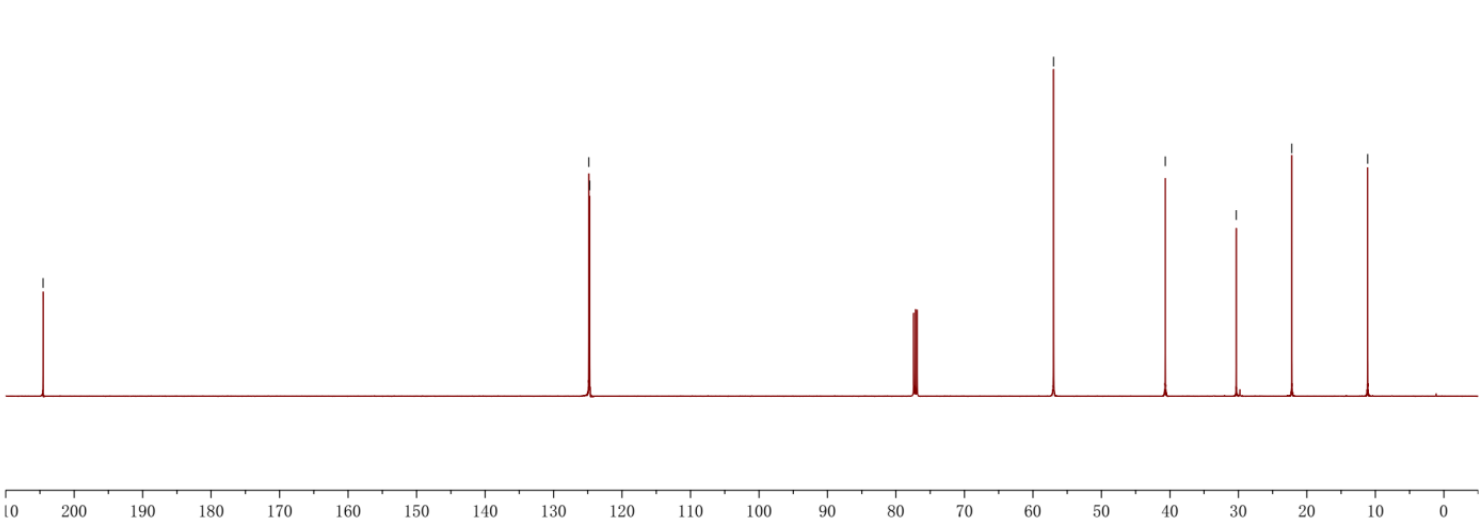

Go back to table of contents

5-Methyl-6-(methylsulfonyl)hexan-2-one (12a)

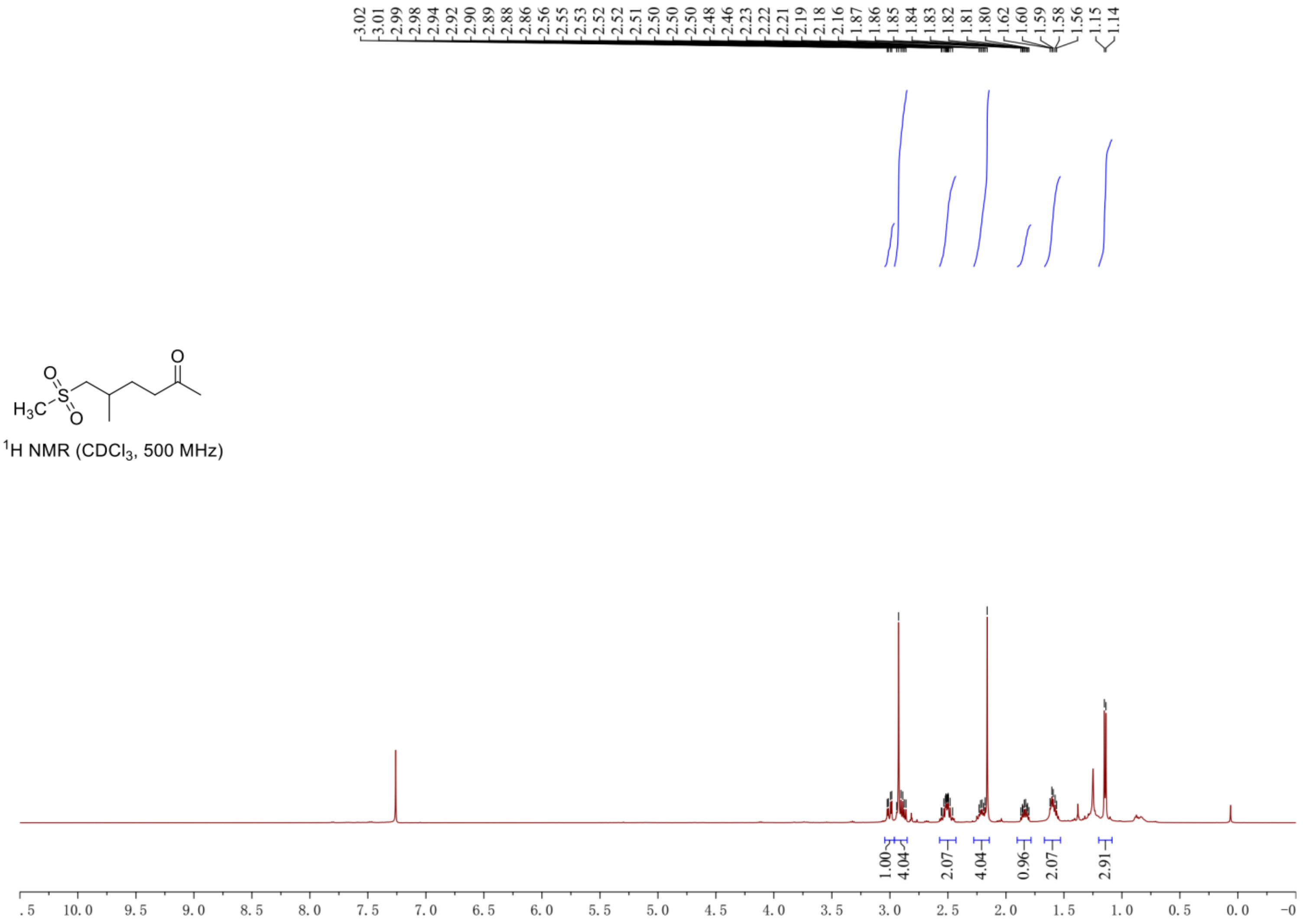

Go back to table of contents

5-Methyl-6-(methylsulfonyl)hexan-2-one (12a)

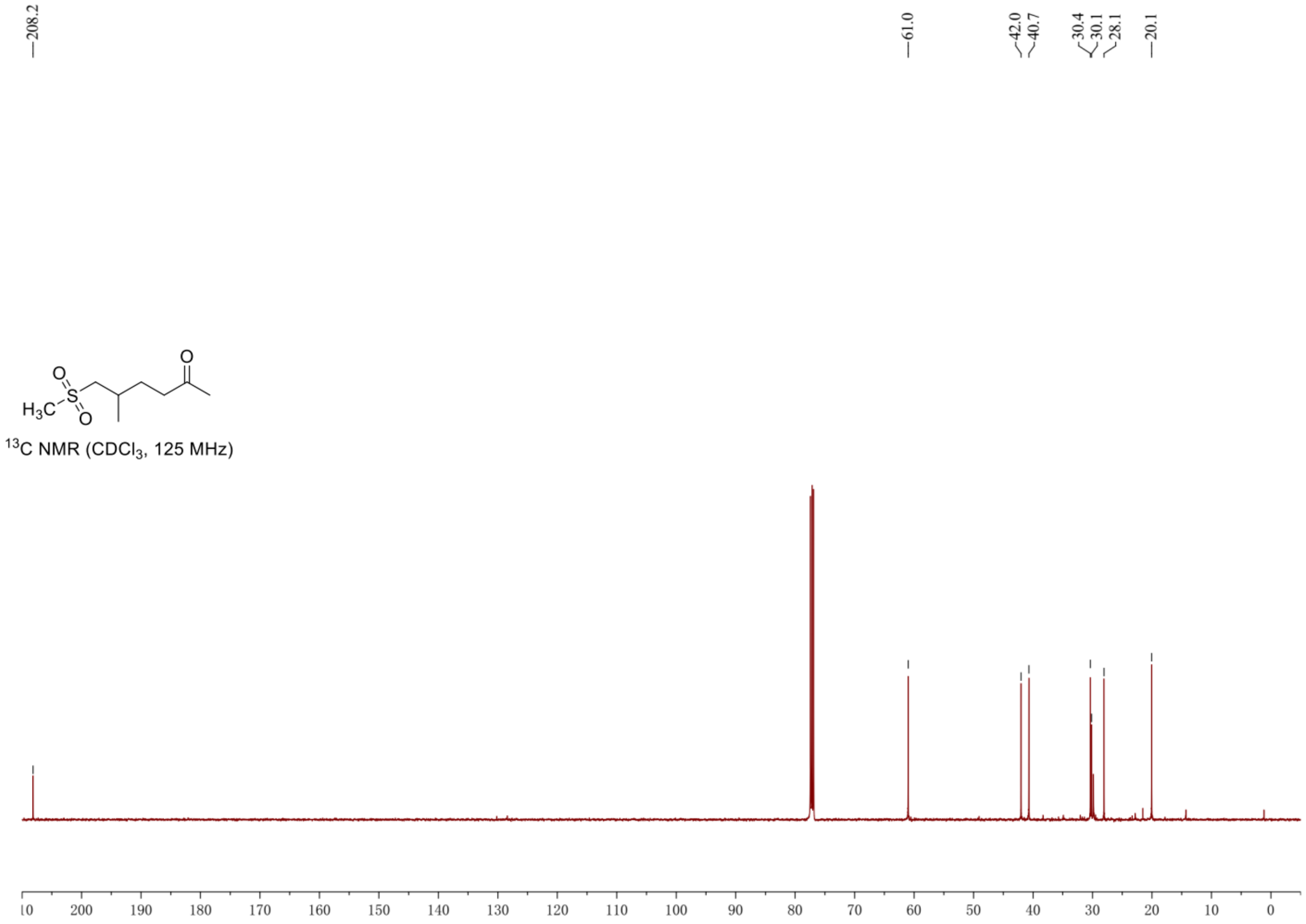

Go back to table of contents

5-Methyl-5-(methylsulfonyl)hexan-2-one (12b)

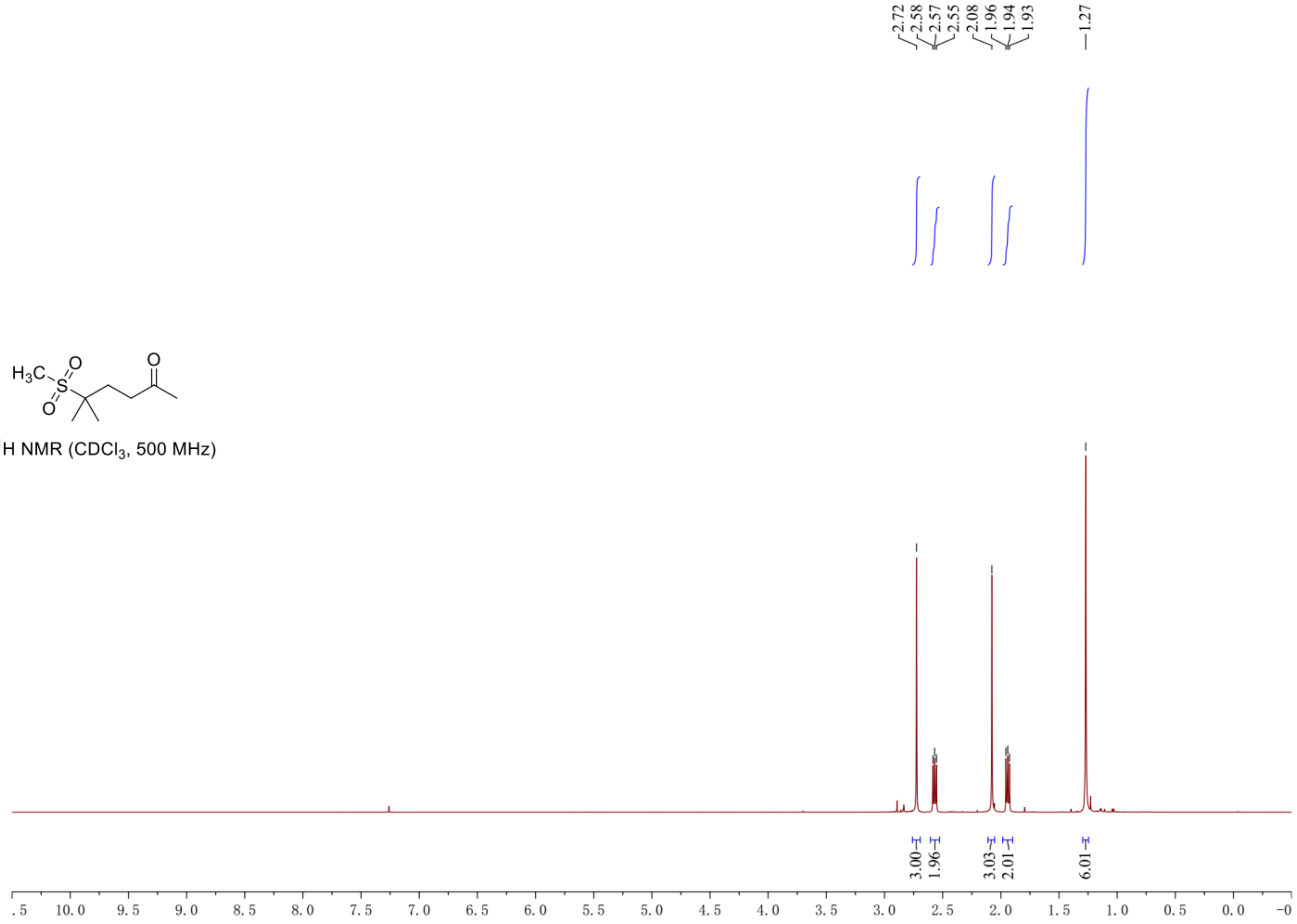

Go back to table of contents

5-Methyl-5-(methylsulfonyl)hexan-2-one (12b)

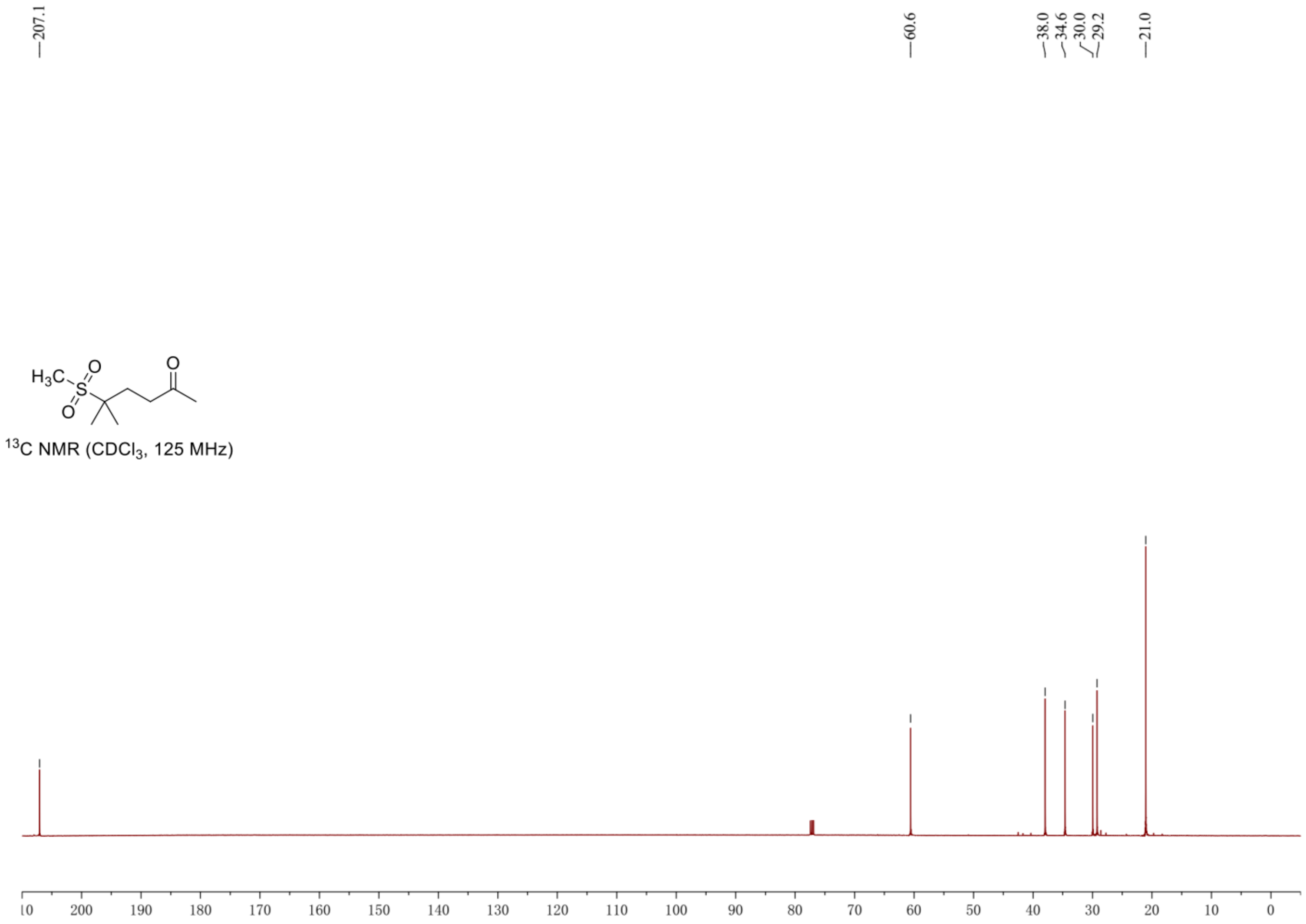

Go back to table of contents

5-Methyl-4-(methylsulfonyl)hexan-2-one (12c)

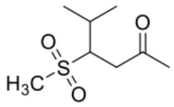

^{1}H NMR ($CDCl_3$, 500 MHz)

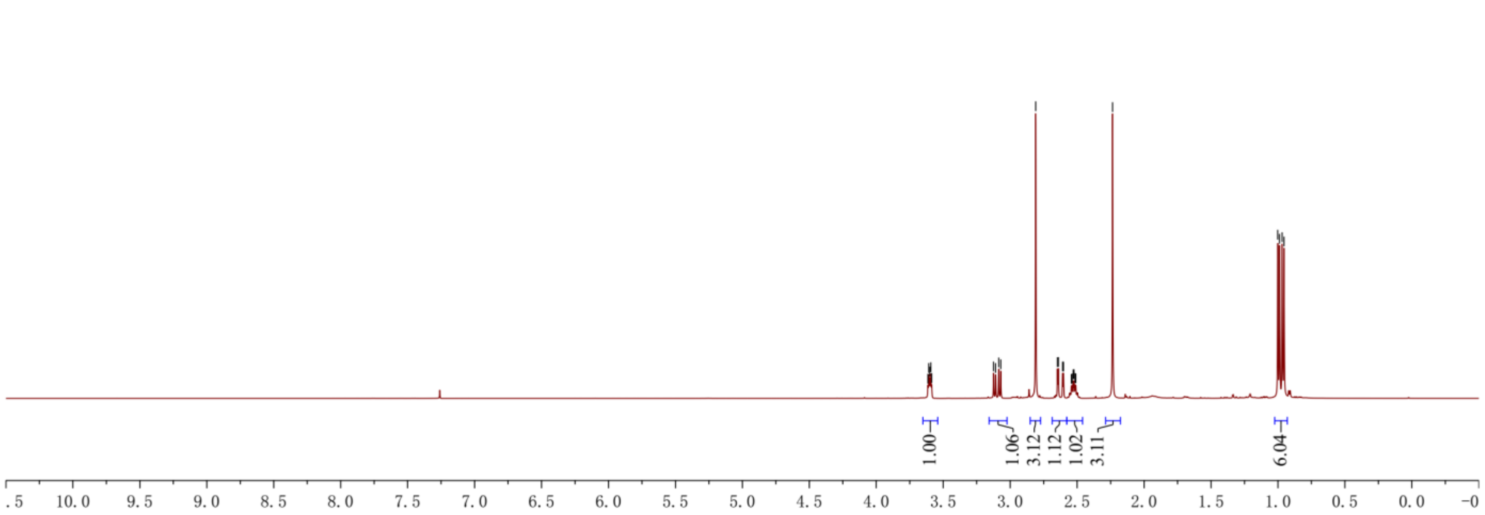

Go back to table of contents

5-Methyl-4-(methylsulfonyl)hexan-2-one (12c)

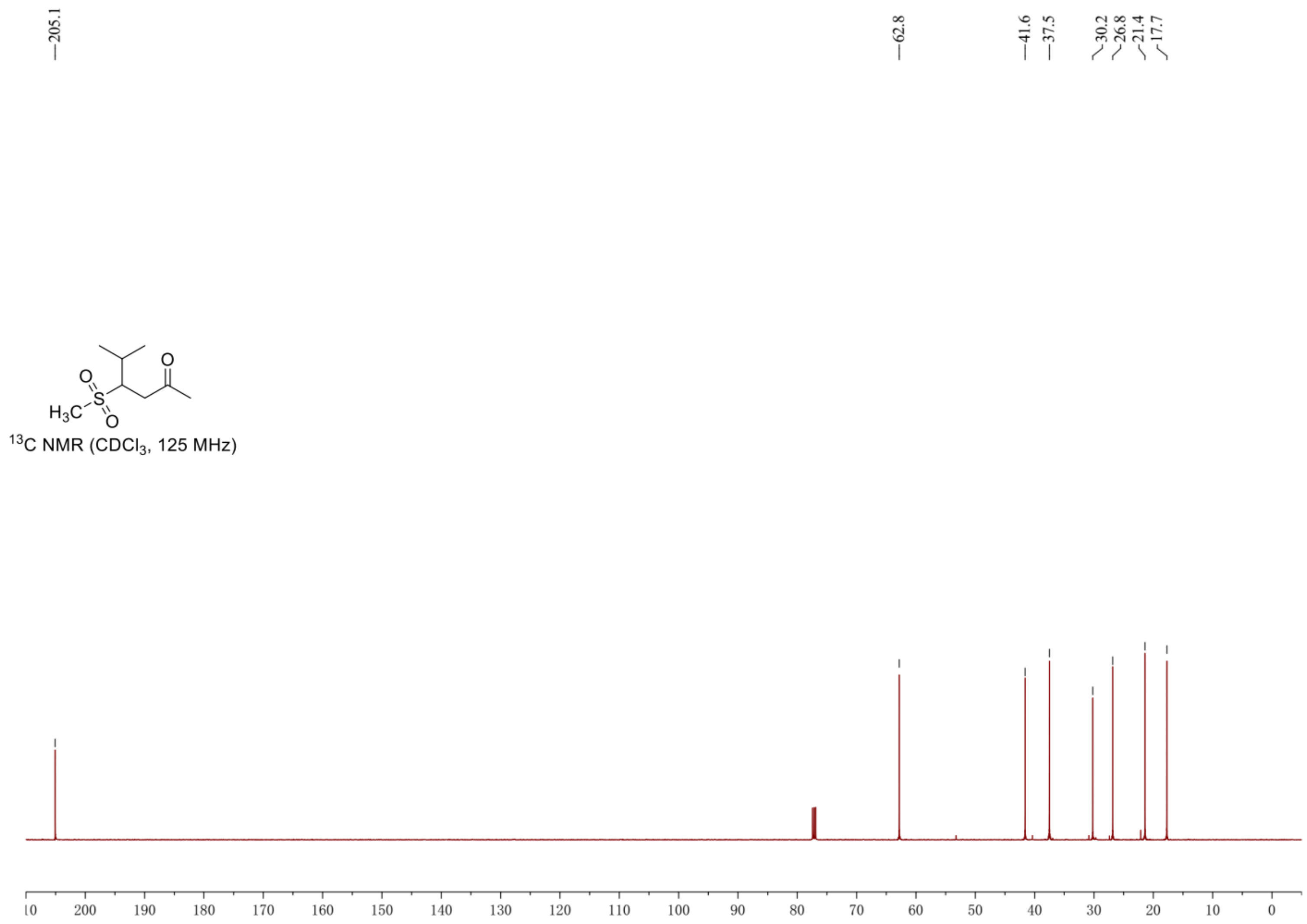

Go back to table of contents

2,6-Dimethyl-1-(methylsulfonyl)heptan-4-one (13a)

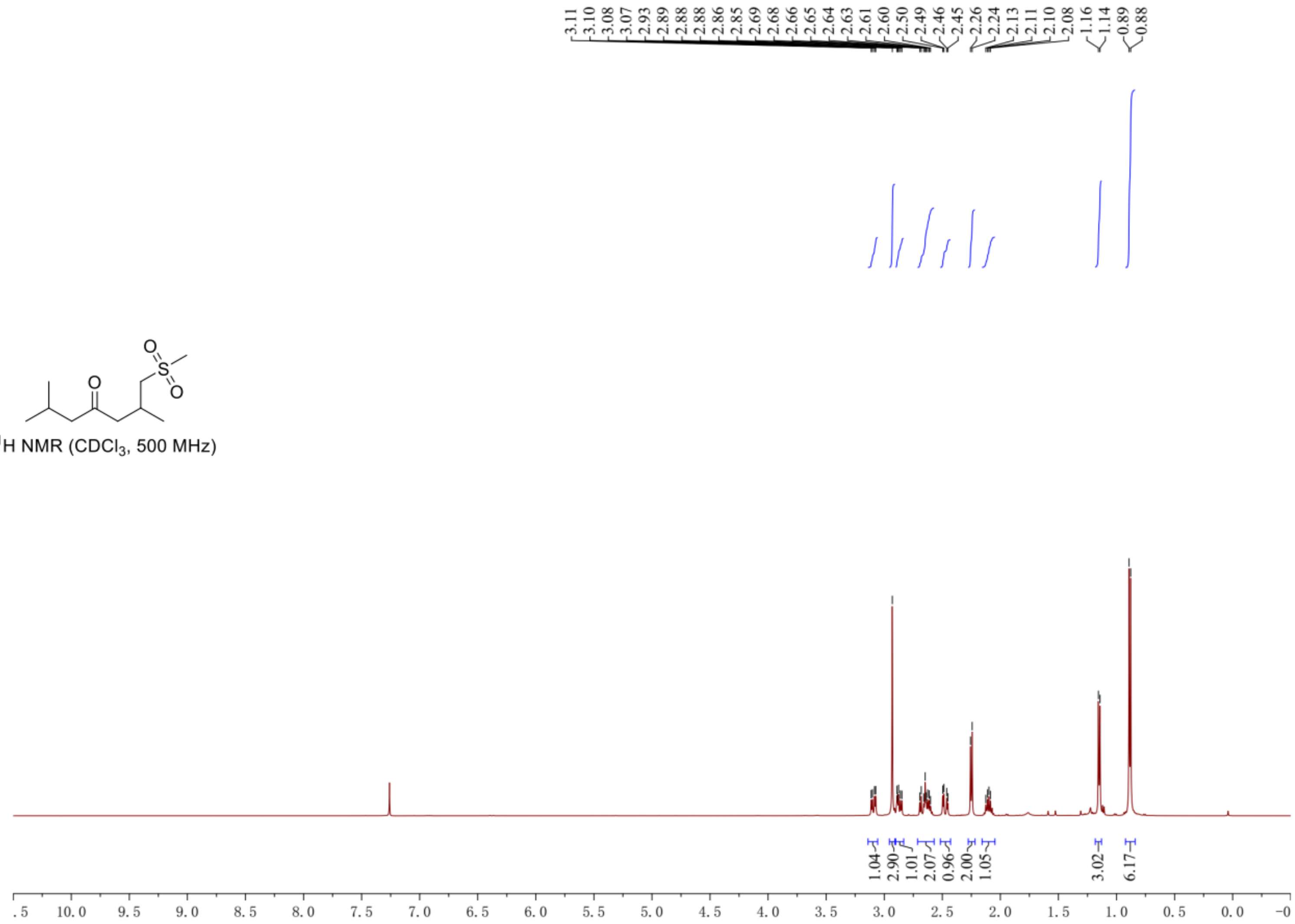

^{1}H NMR ($CDCl_3$, 500 MHz)

Go back to table of contents

2,6-Dimethyl-1-(methylsulfonyl)heptan-4-one (13a)

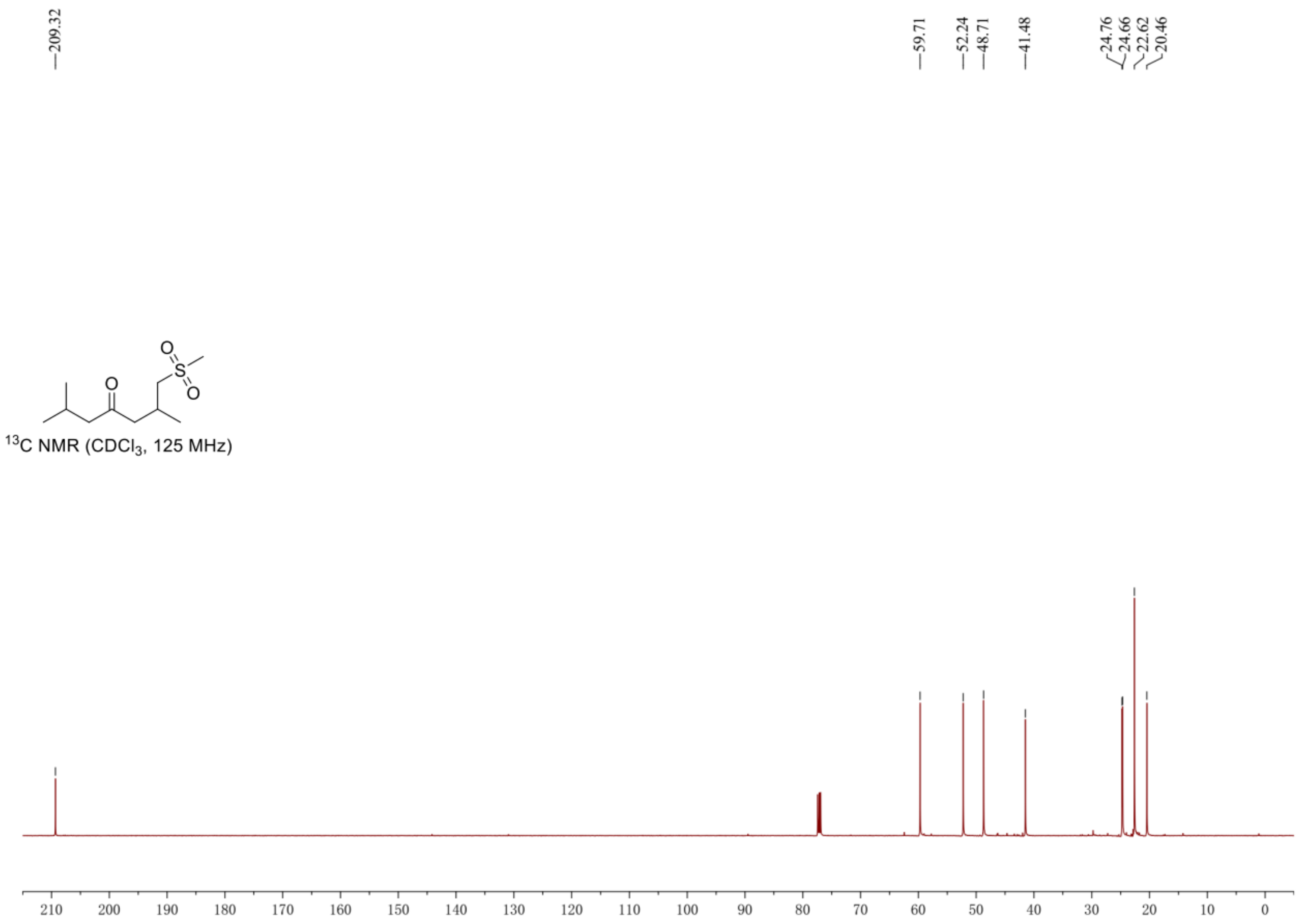

Go back to table of contents

2,6-Dimethyl-2-(methylsulfonyl)heptan-4-one (13b)

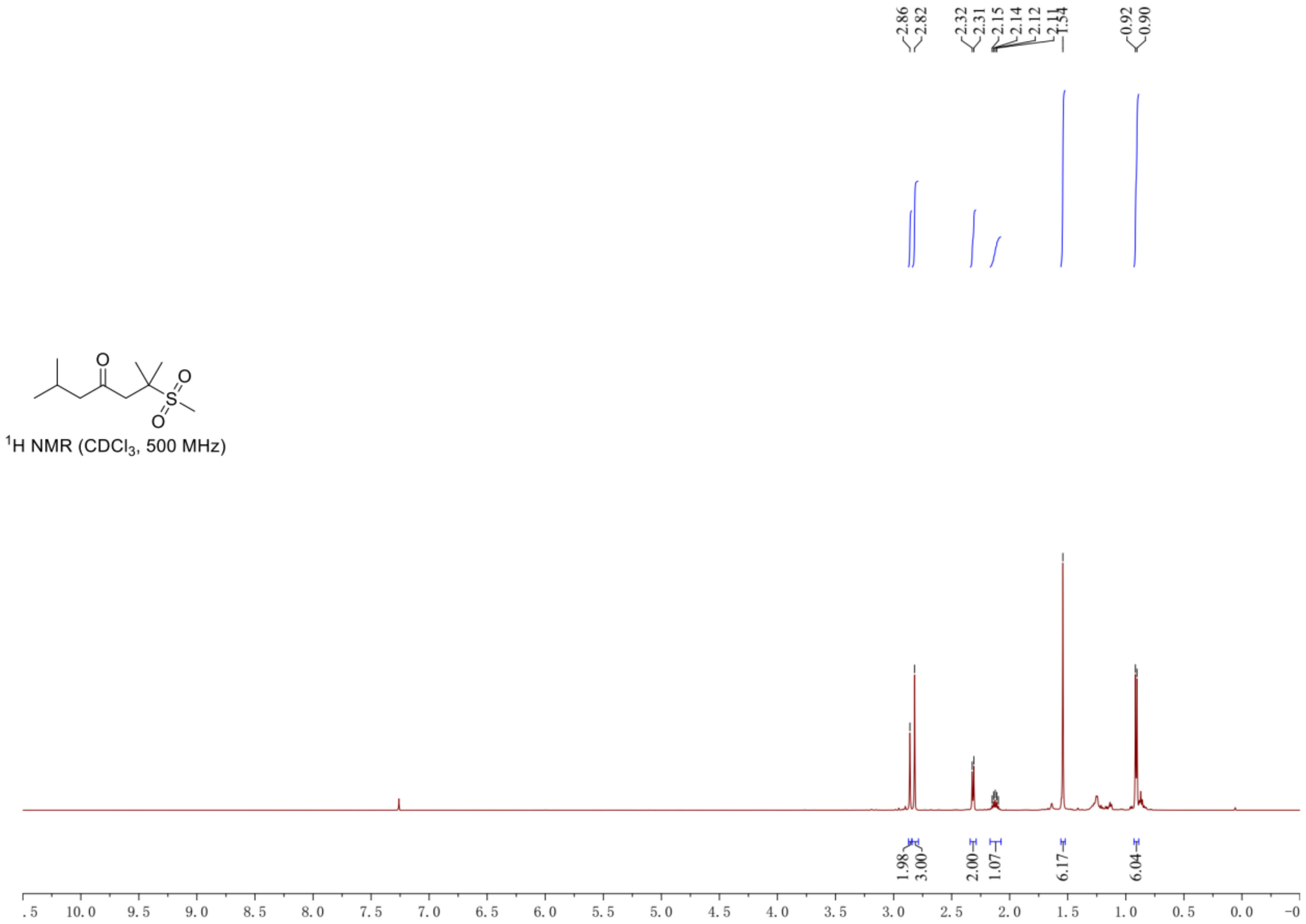

Go back to table of contents

2,6-Dimethyl-2-(methylsulfonyl)heptan-4-one (13b)

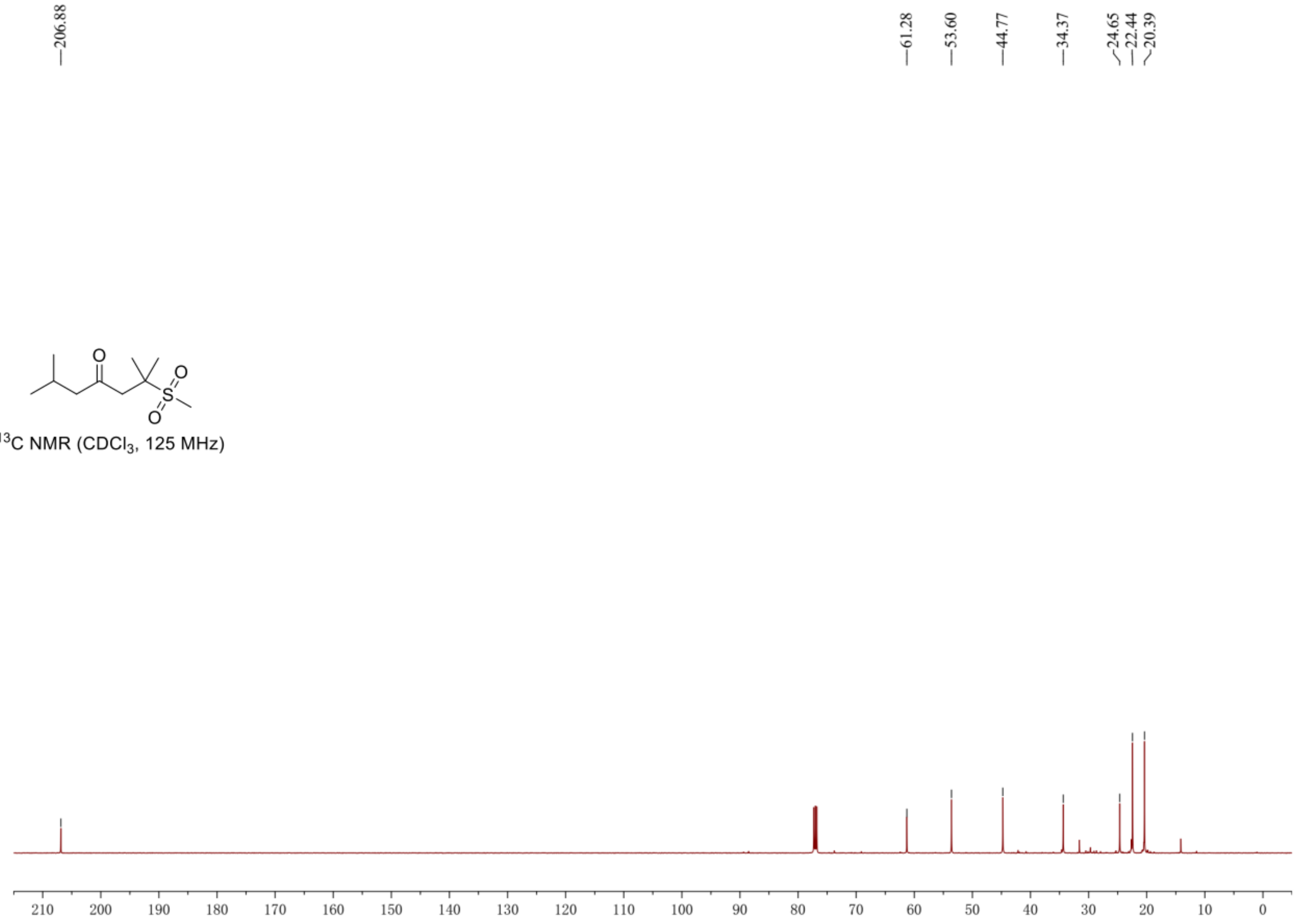

^{13}C NMR ($CDCl_3$, 125 MHz)

Go back to table of contents

1-(Allylsulfonyl)adamantan-2-one (14a)

^{1}H NMR ($CDCl_3$, 500 MHz)

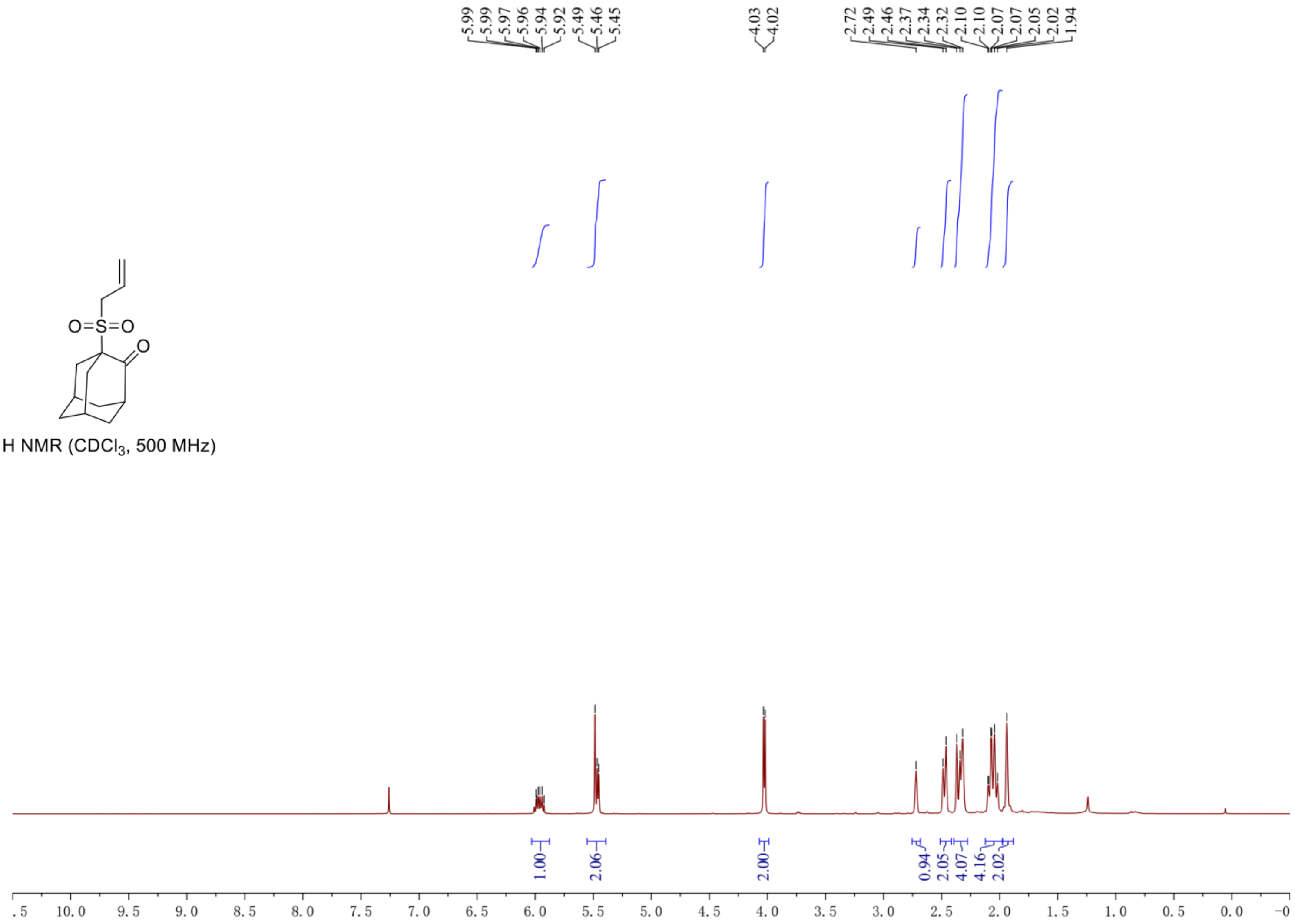

Go back to table of contents

1-(Allylsulfonyl)adamantan-2-one (14a)

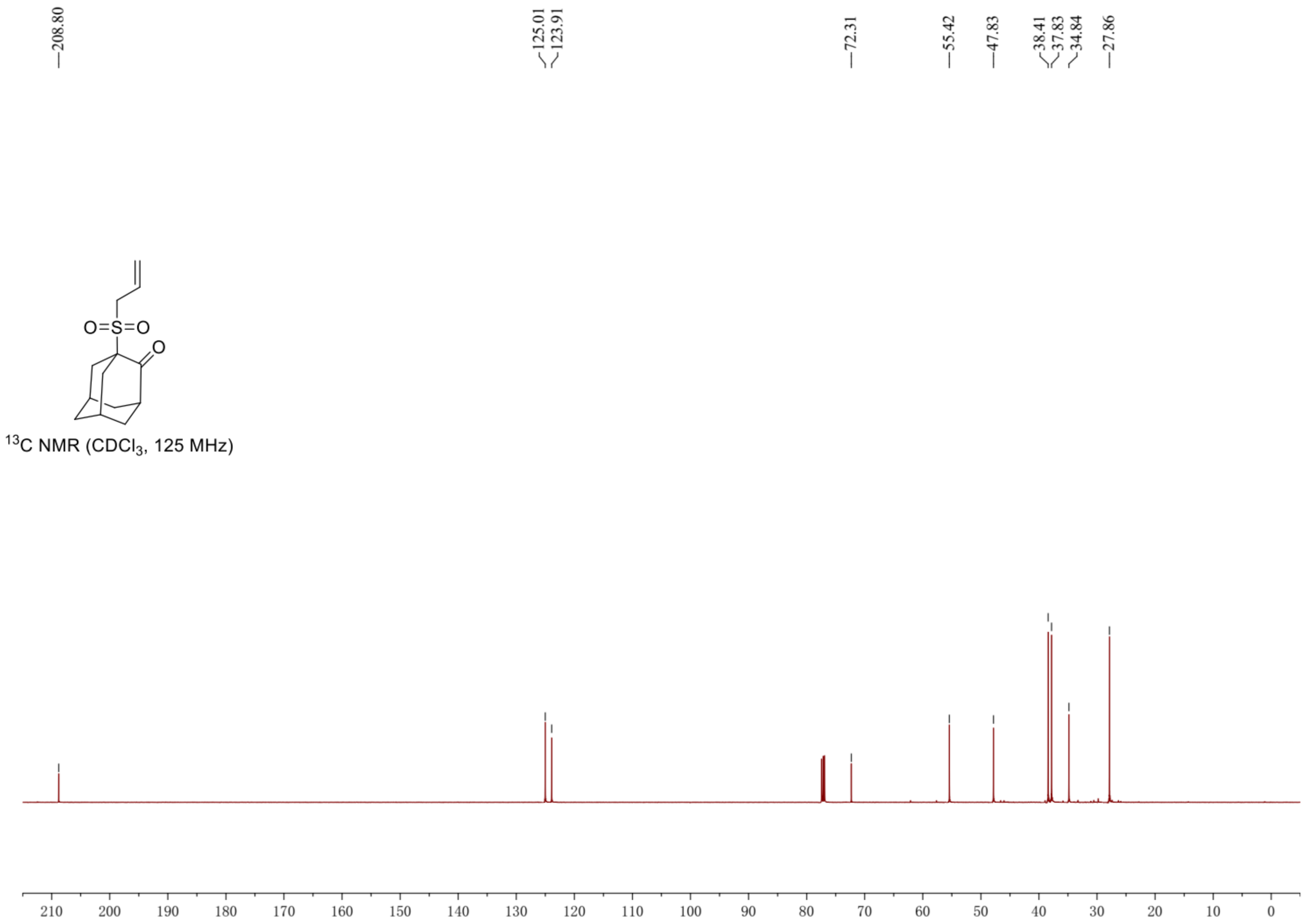

Go back to table of contents

4-(Allylsulfonyl)adamantan-2-one (14b)

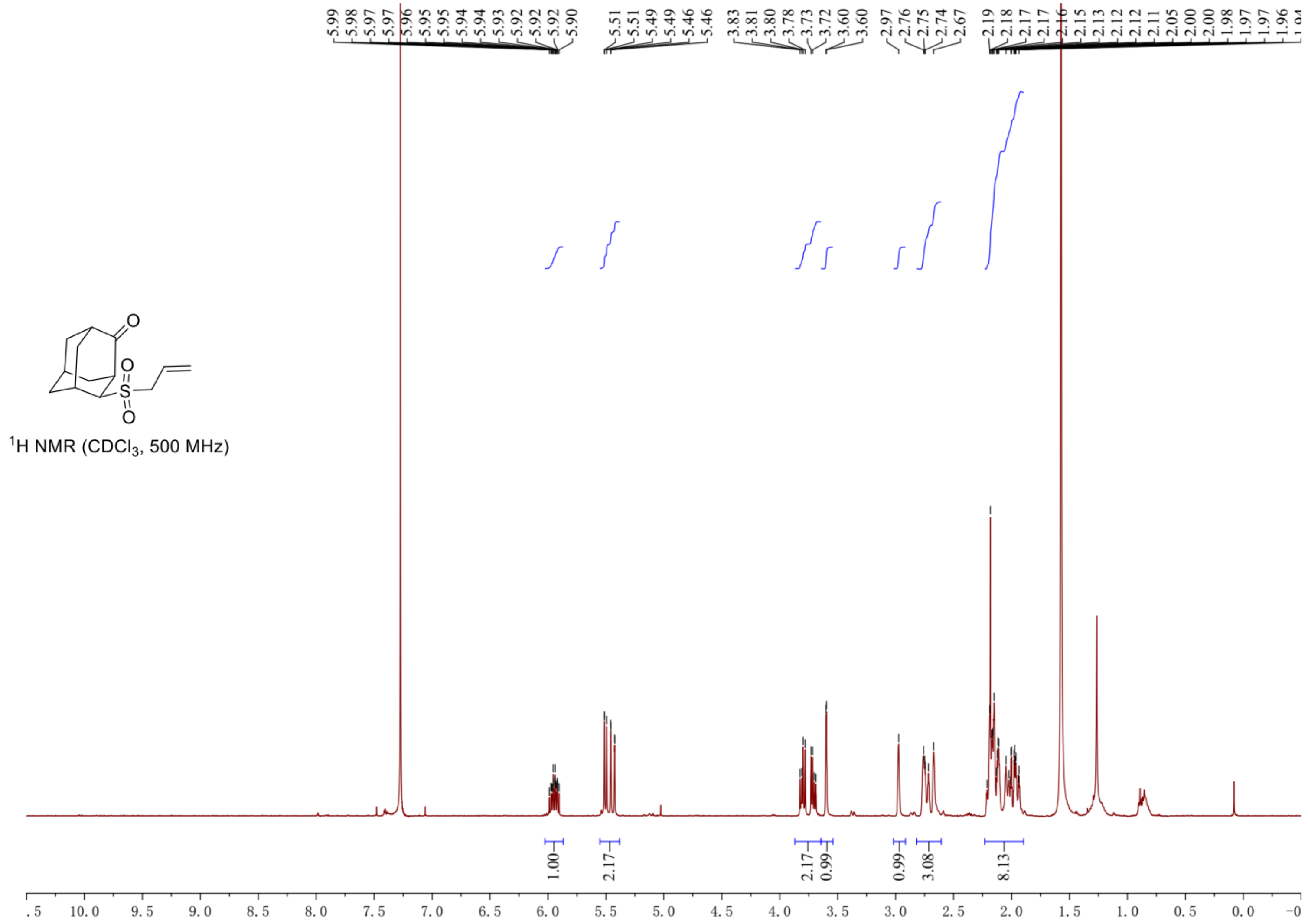

Go back to table of contents

4-(Allylsulfonyl)adamantan-2-one (14b)

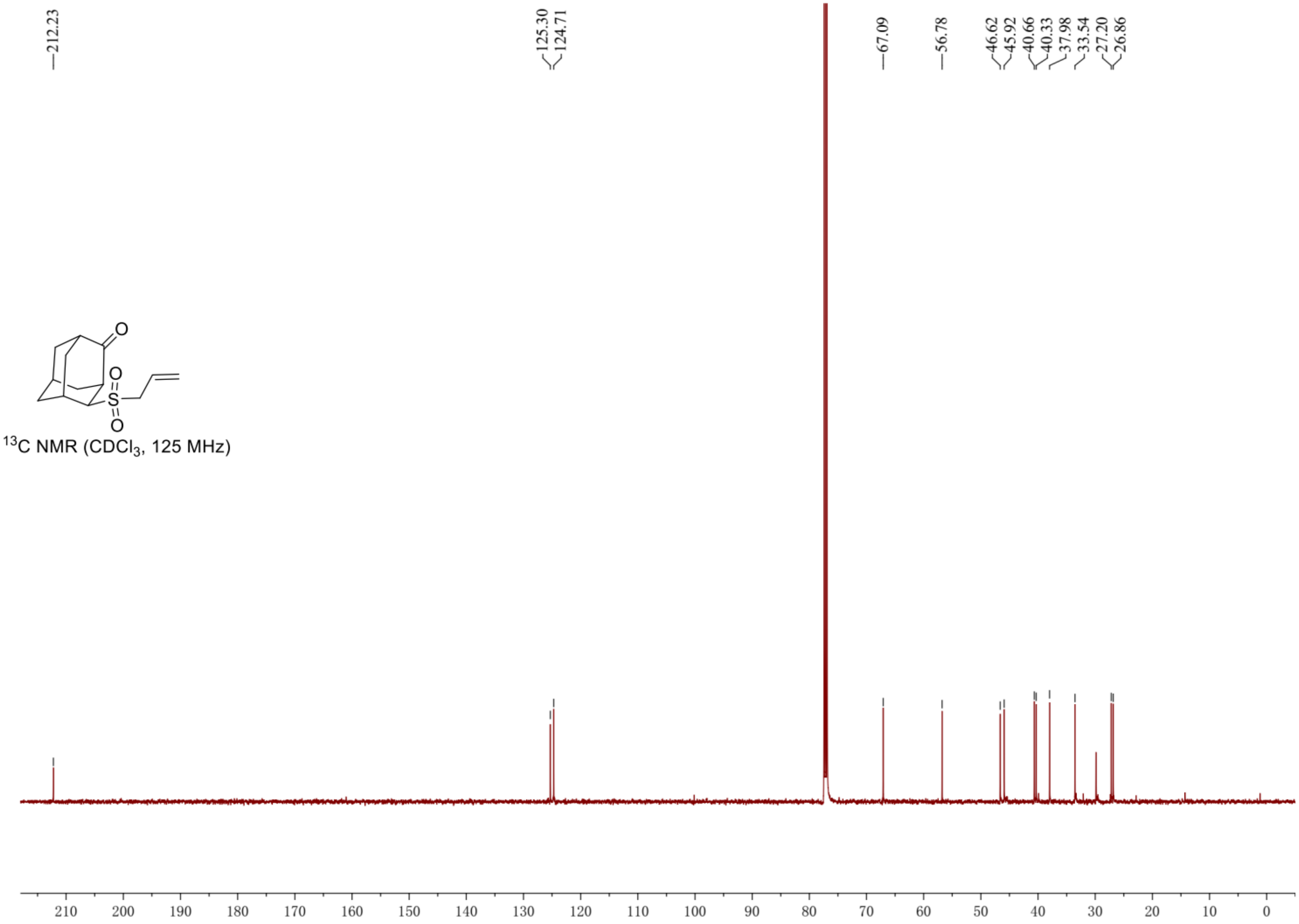

Go back to table of contents

5-(Allylsulfonyl)adamantan-2-one (14c)

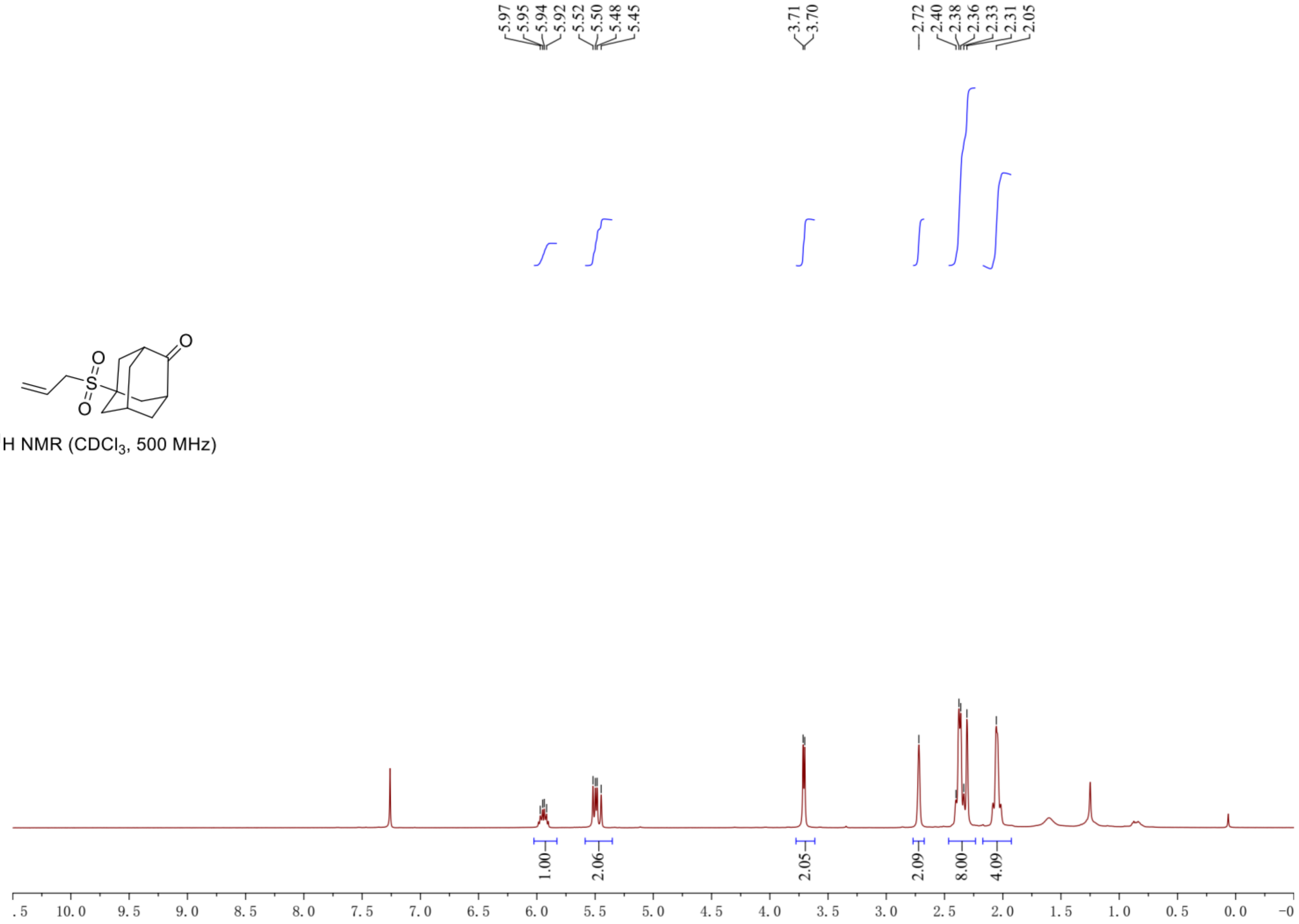

^{1}H NMR (CDCl$_3$, 500 MHz)

5-(Allylsulfonyl)adamantan-2-one (14c)

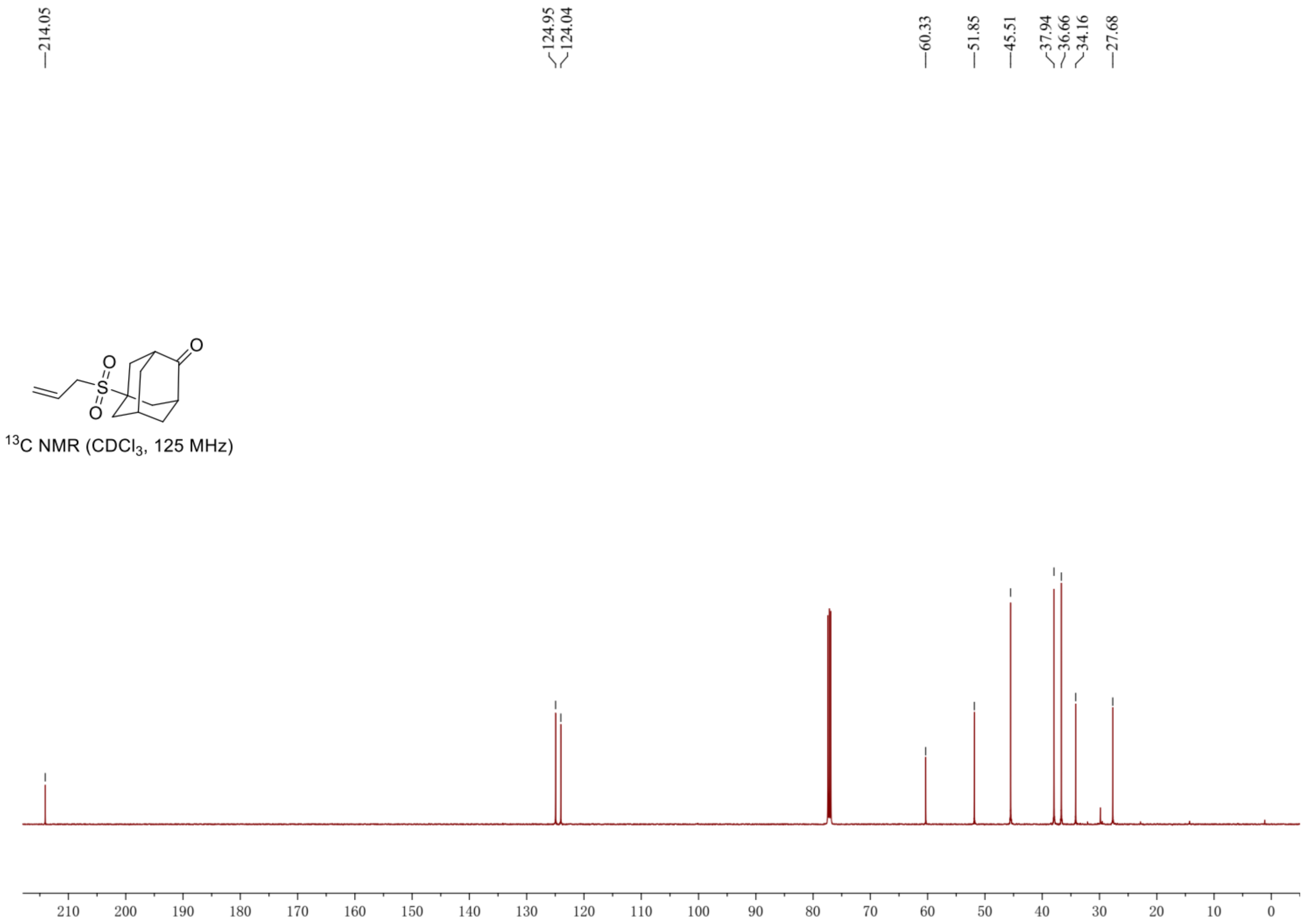

Go back to table of contents

6-(Allylsulfonyl)adamantan-2-one (14d)

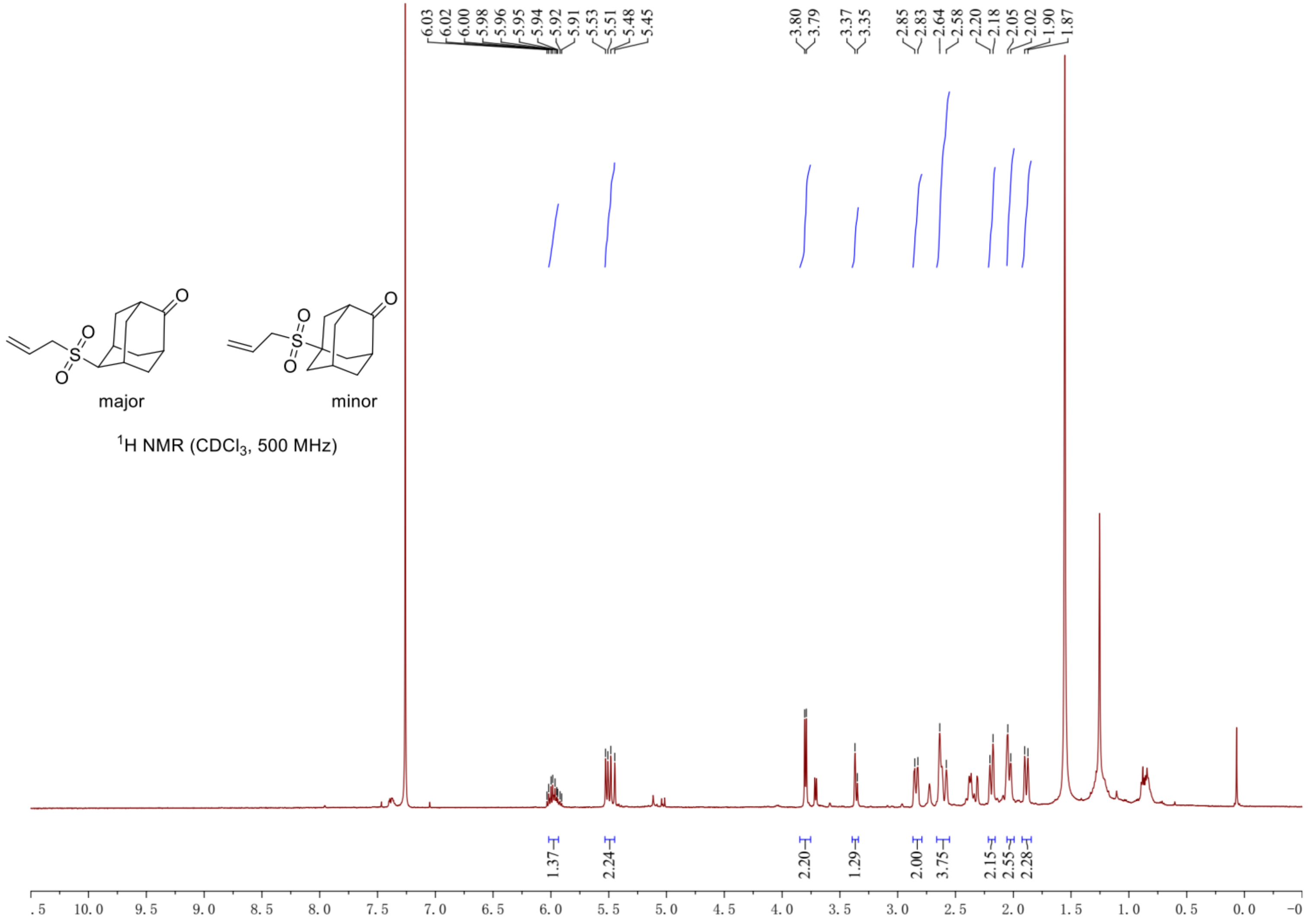

Go back to table of contents

6-(Allylsulfonyl)adamantan-2-one (14d)

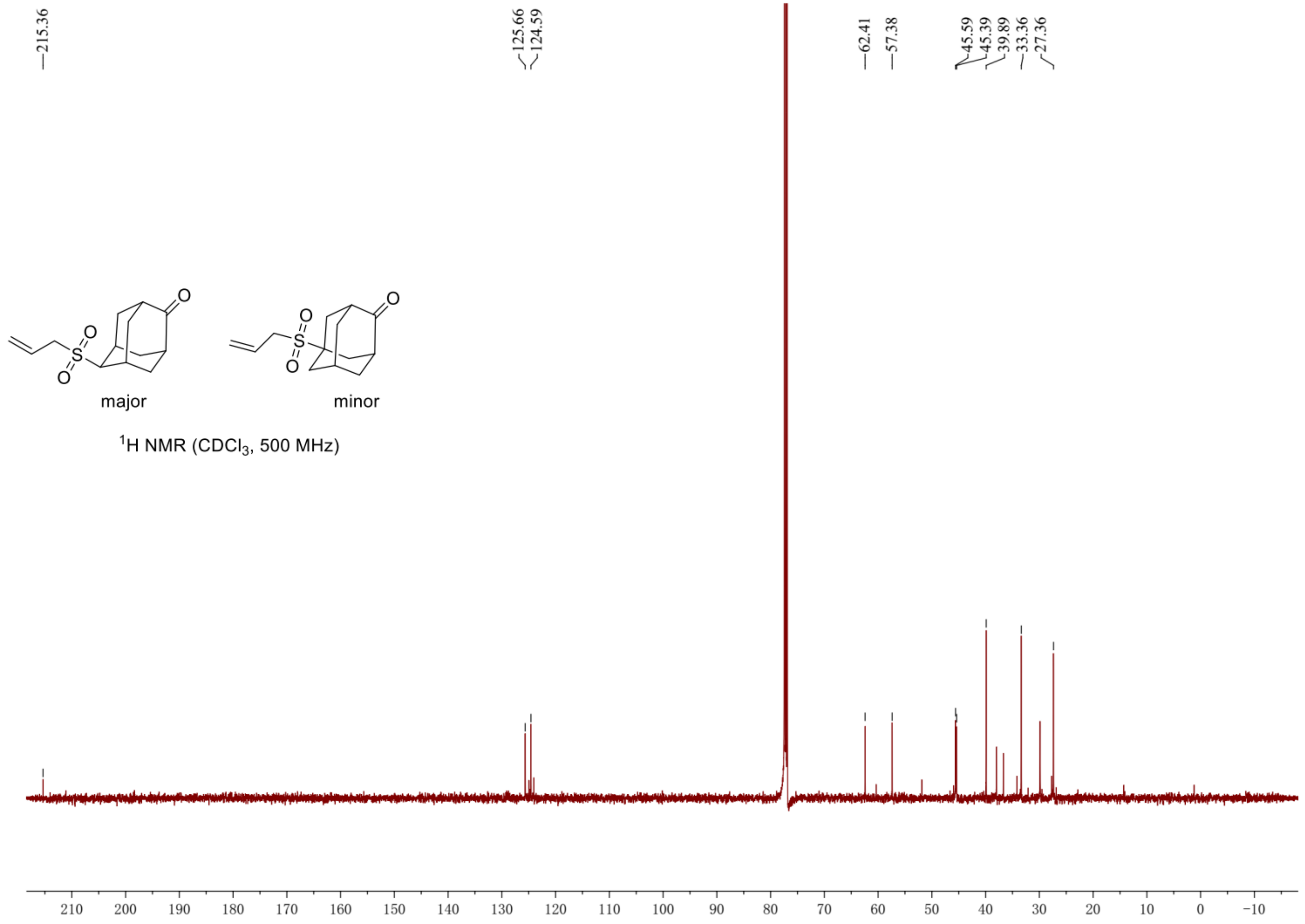

Go back to table of contents

Methyl 4-(allylsulfonyl)butanoate (15a)

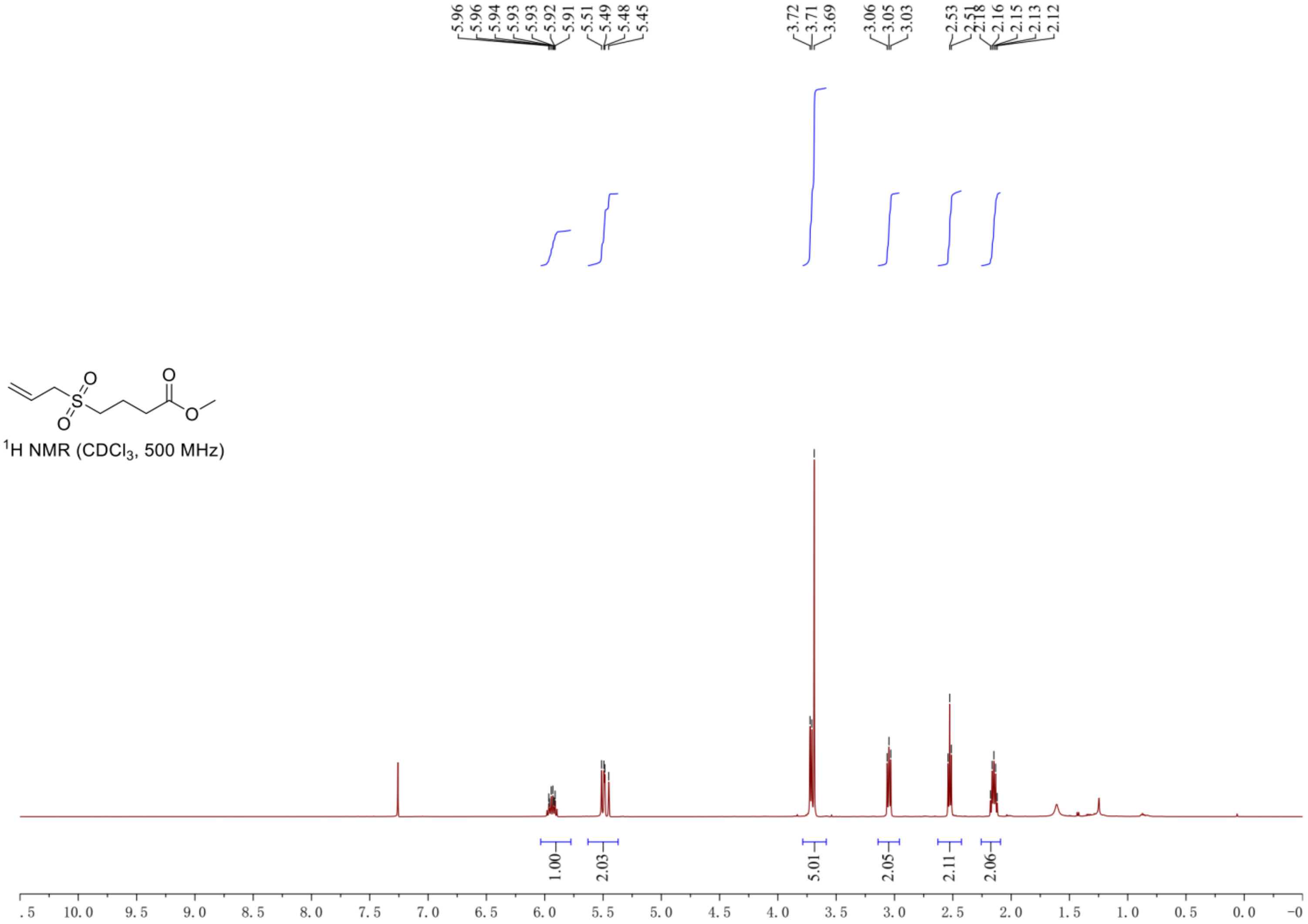

Go back to table of contents

Methyl 4-(allylsulfonyl)butanoate (15a)

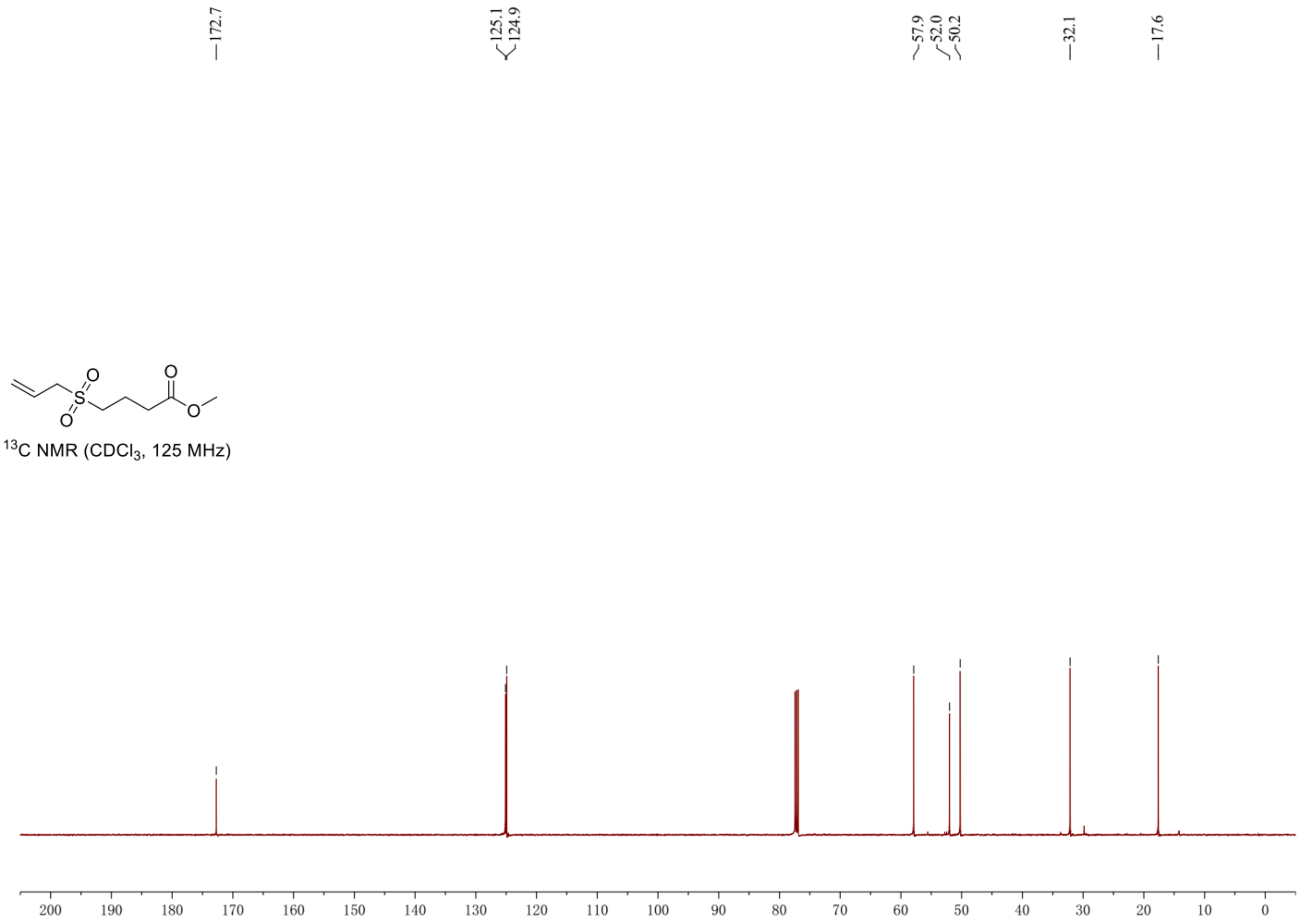

Go back to table of contents

Methyl 3-(allylsulfonyl)butanoate (15b)

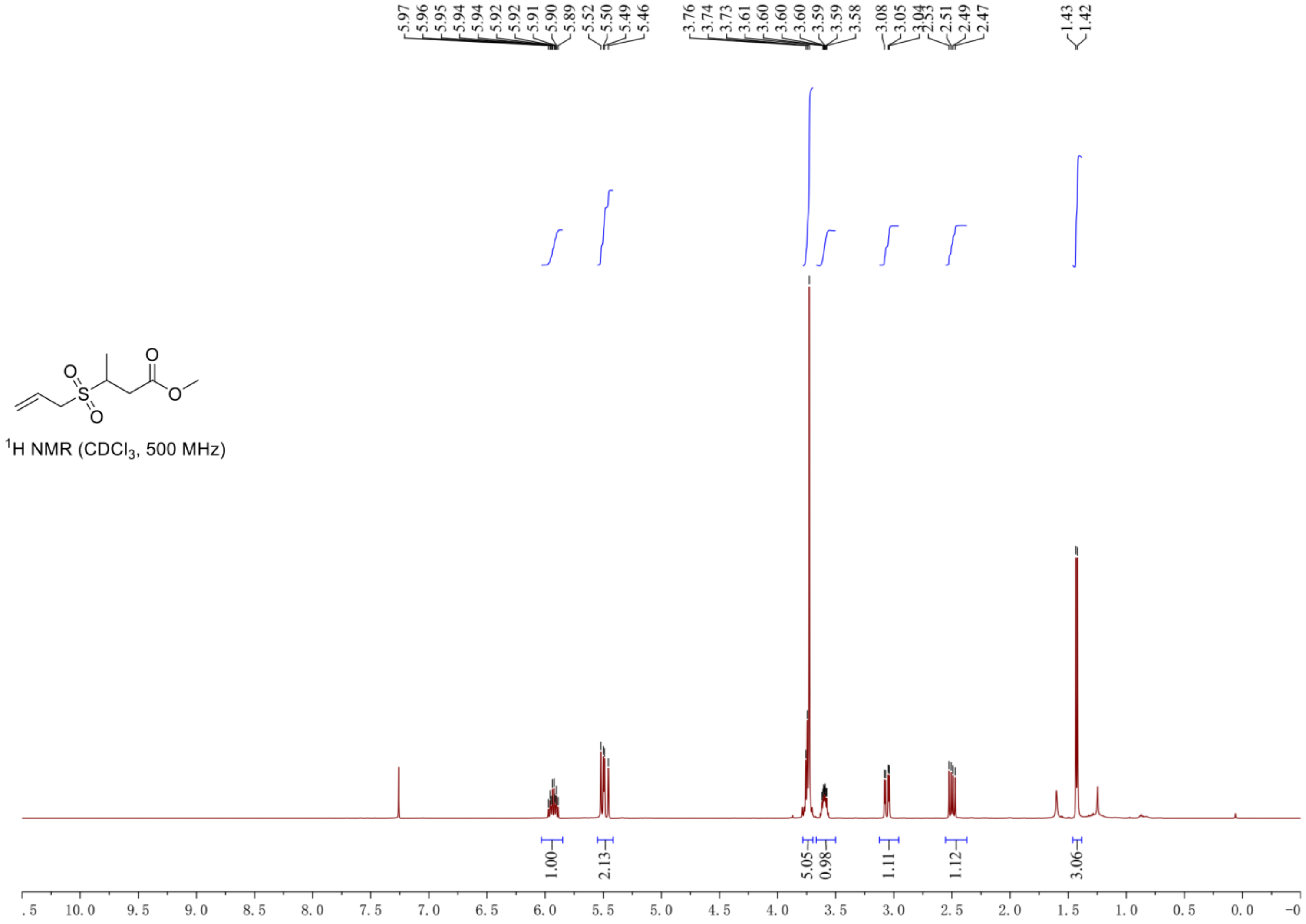

Go back to table of contents

Methyl 3-(allylsulfonyl)butanoate (15b)

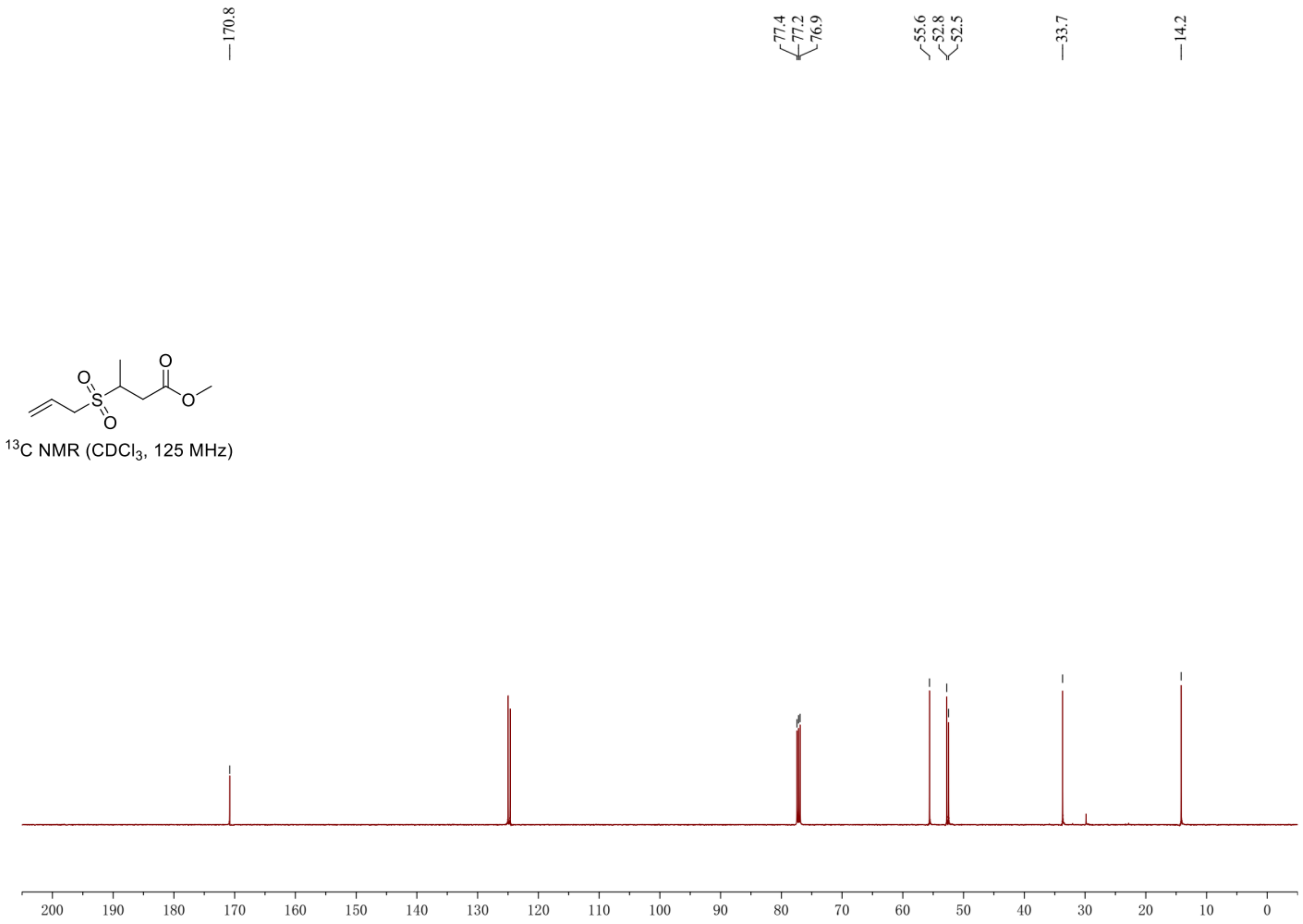

Go back to table of contents

trans-Dimethyl-4-(allylsulfonyl)cyclopentane-1,2-dicarboxylate (16a)

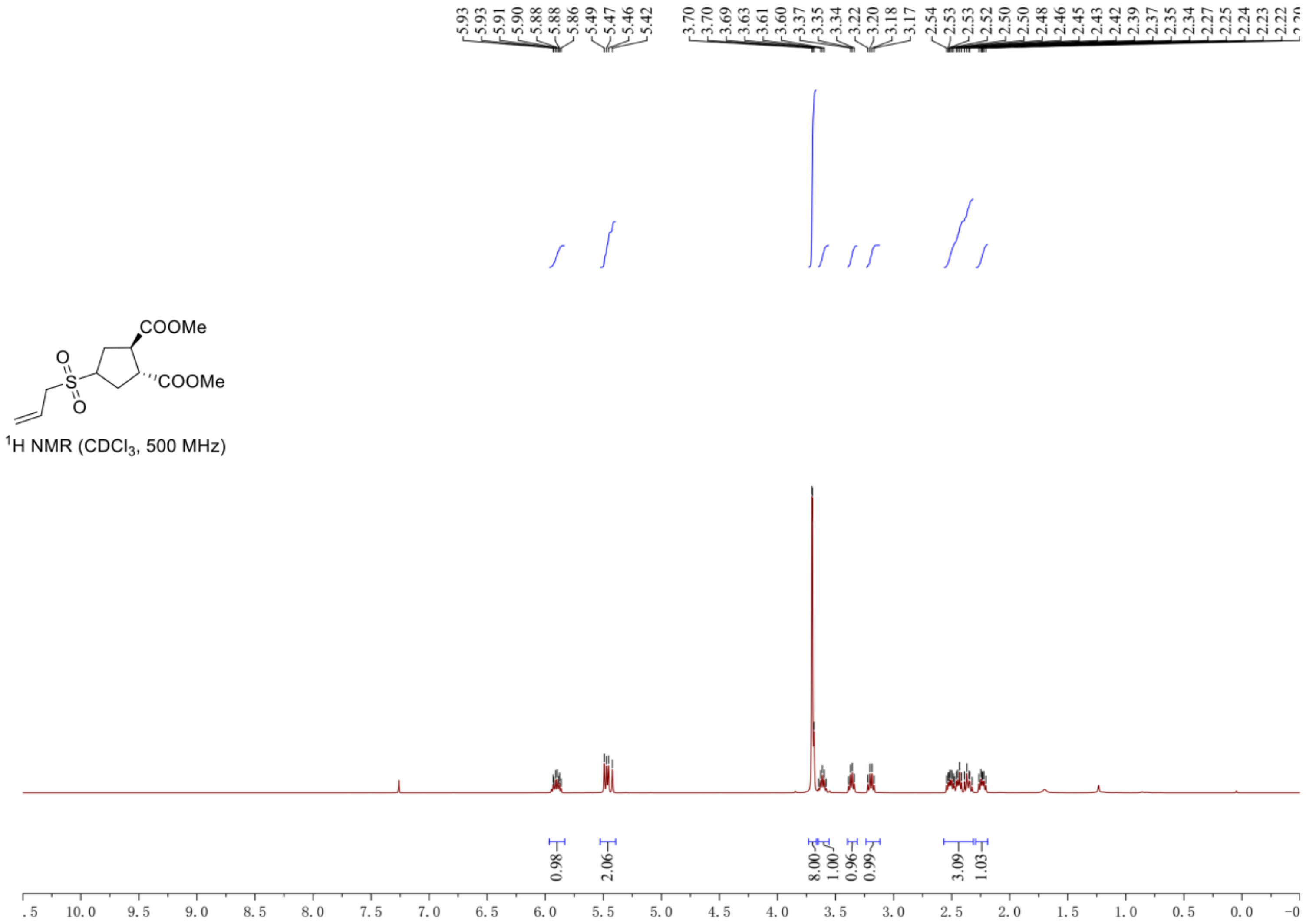

^1H NMR ($CDCl_3$, 500 MHz)

Go back to table of contents

trans-Dimethyl-4-(allylsulfonyl)cyclopentane-1,2-dicarboxylate (16a)

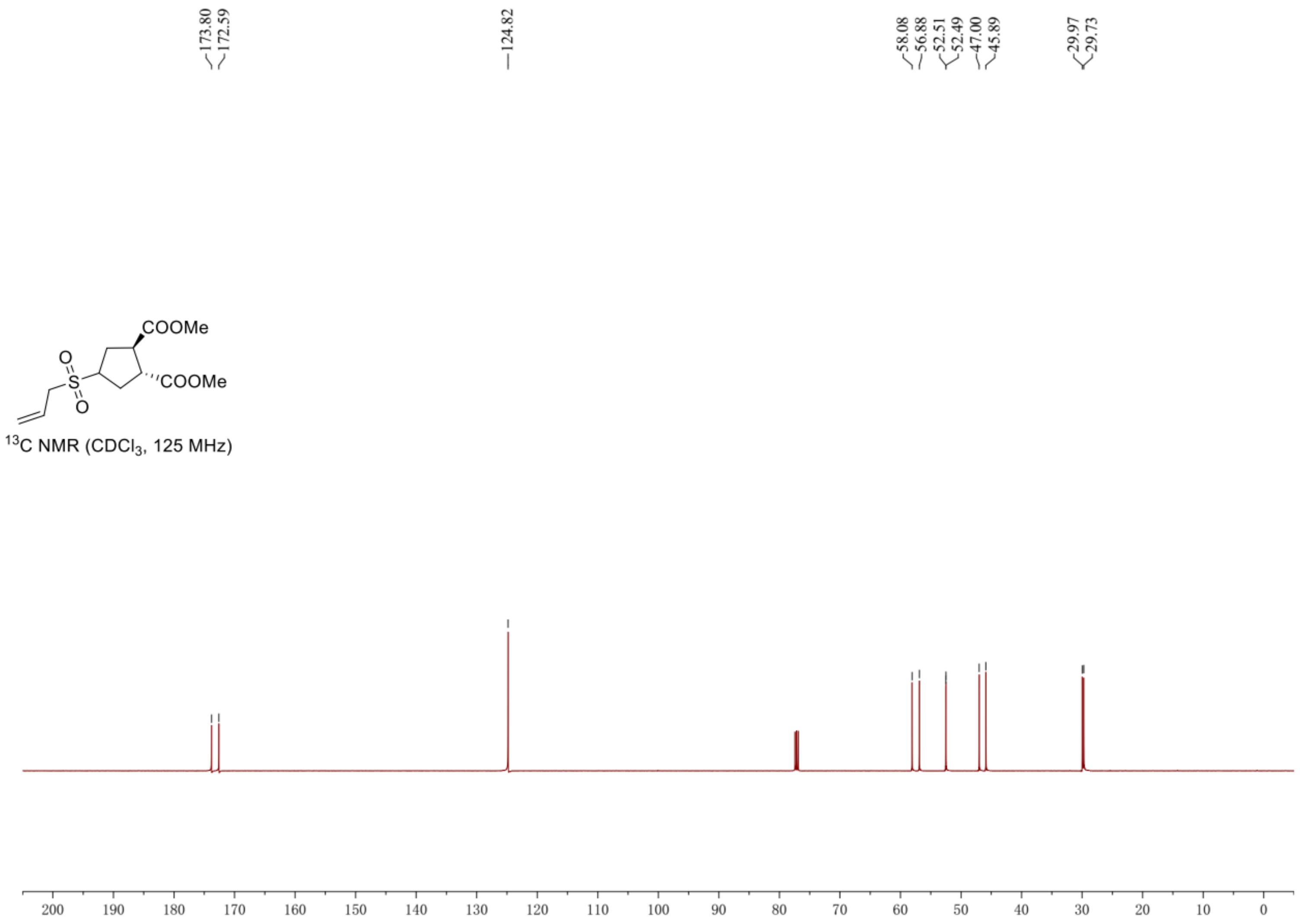

Go back to table of contents

trans, _trans_-Dimethyl-3-(allylsulfonyl)cyclopentane-1,2-dicarboxylate (16b)

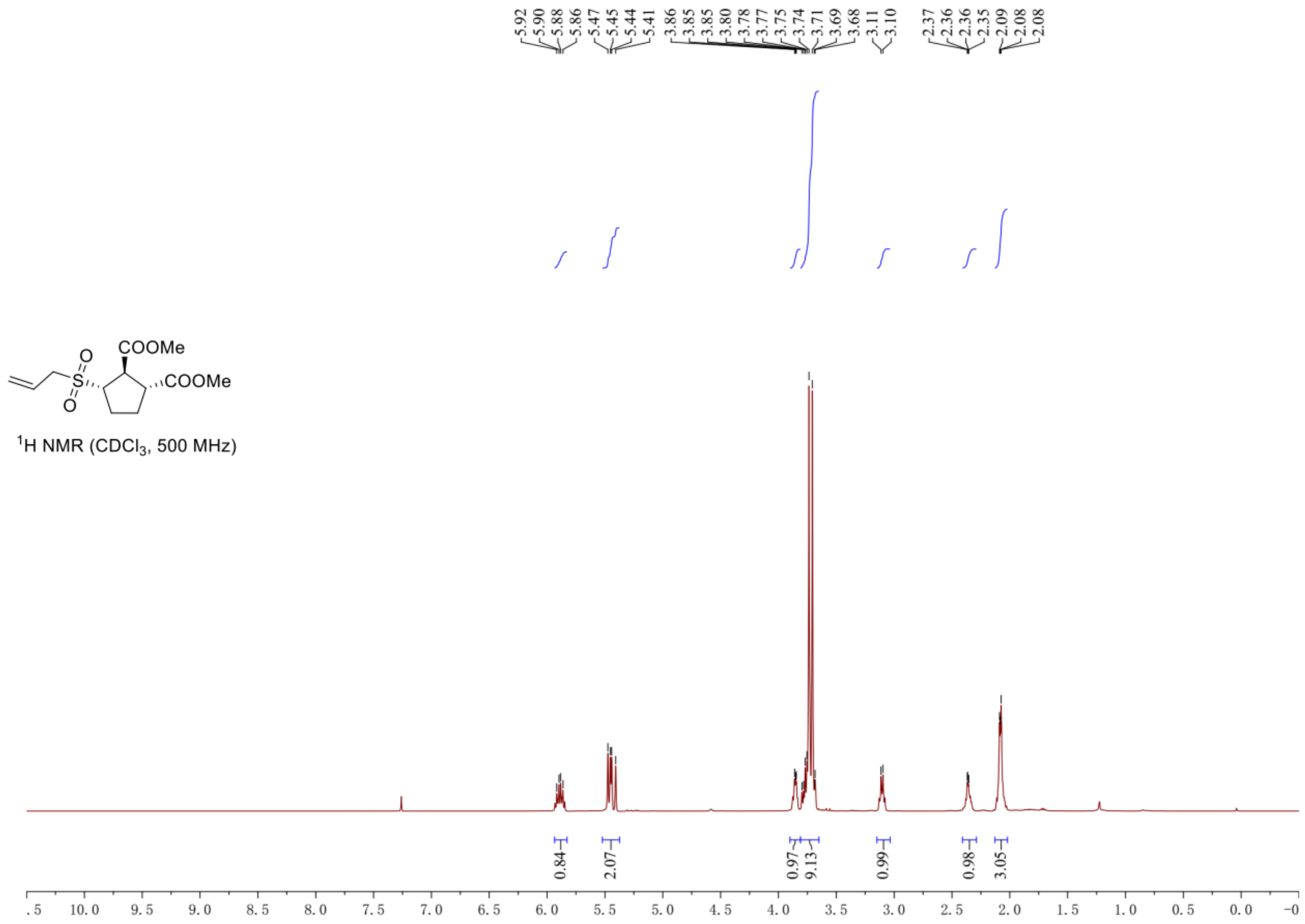

Go back to table of contents

trans, *trans*-Dimethyl-3-(allylsulfonyl)cyclopentane-1,2-dicarboxylate (16b)

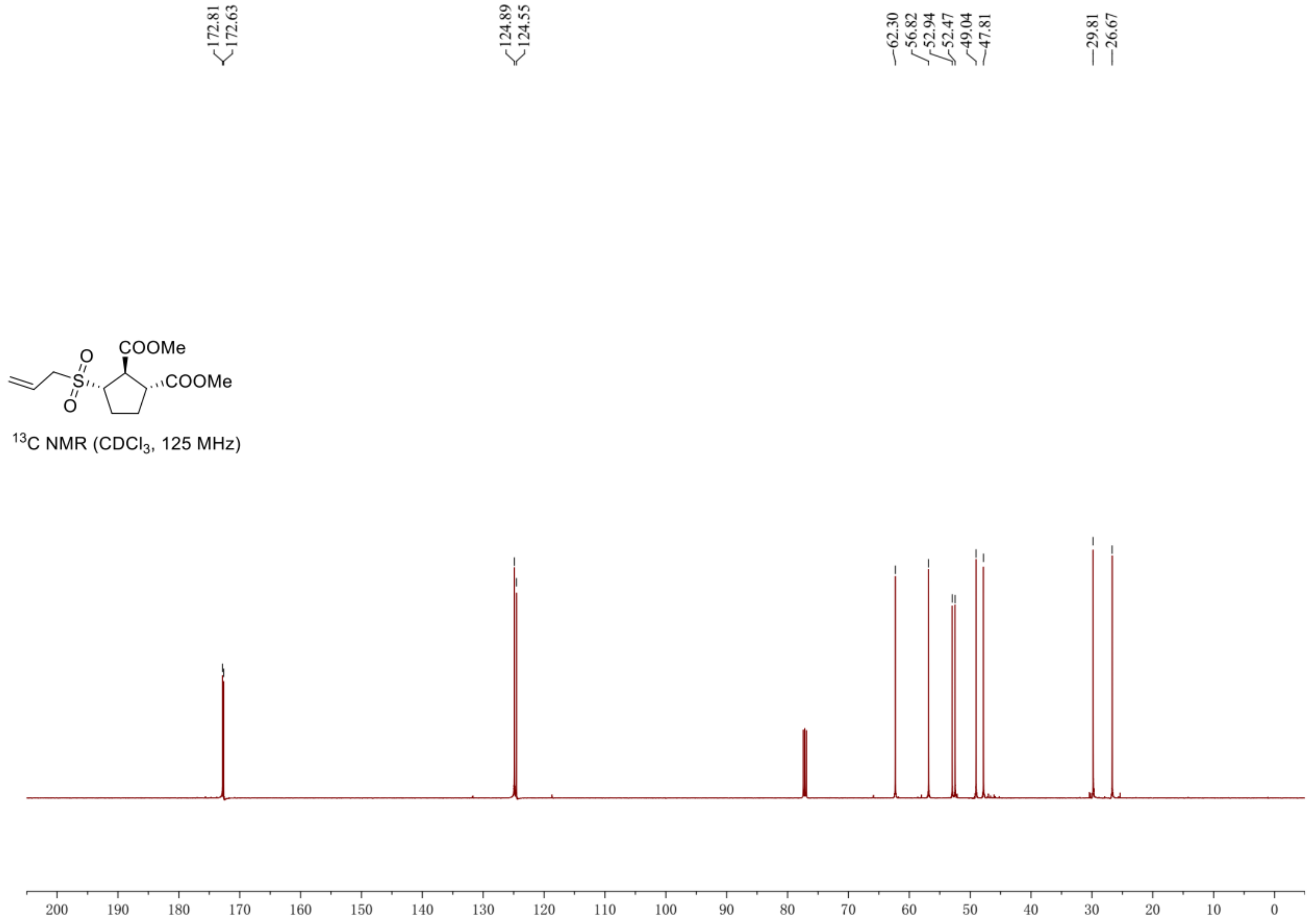

Go back to table of contents

2-Methyl-1-(methylsulfonyl)propyl acetate (17a)

2-Methyl-2-(methylsulfonyl)propyl acetate (17b)

2-Methyl-3-(methylsulfonyl)propyl acetate (17c)

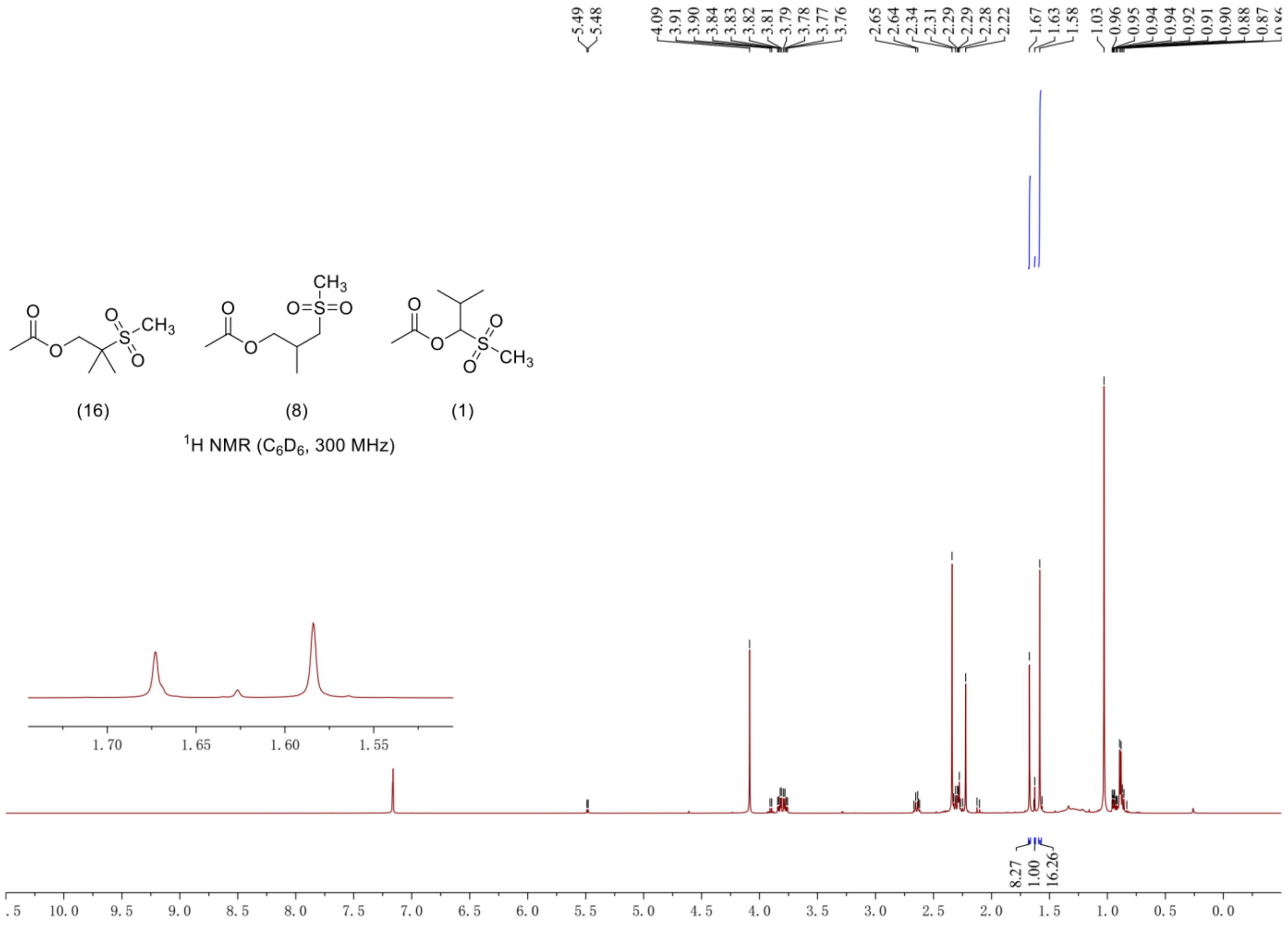

Go back to table of contents

2-Methyl-1-(methylsulfonyl)propyl acetate (17a)

2-Methyl-2-(methylsulfonyl)propyl acetate (17b)

2-Methyl-3-(methylsulfonyl)propyl acetate (17c)

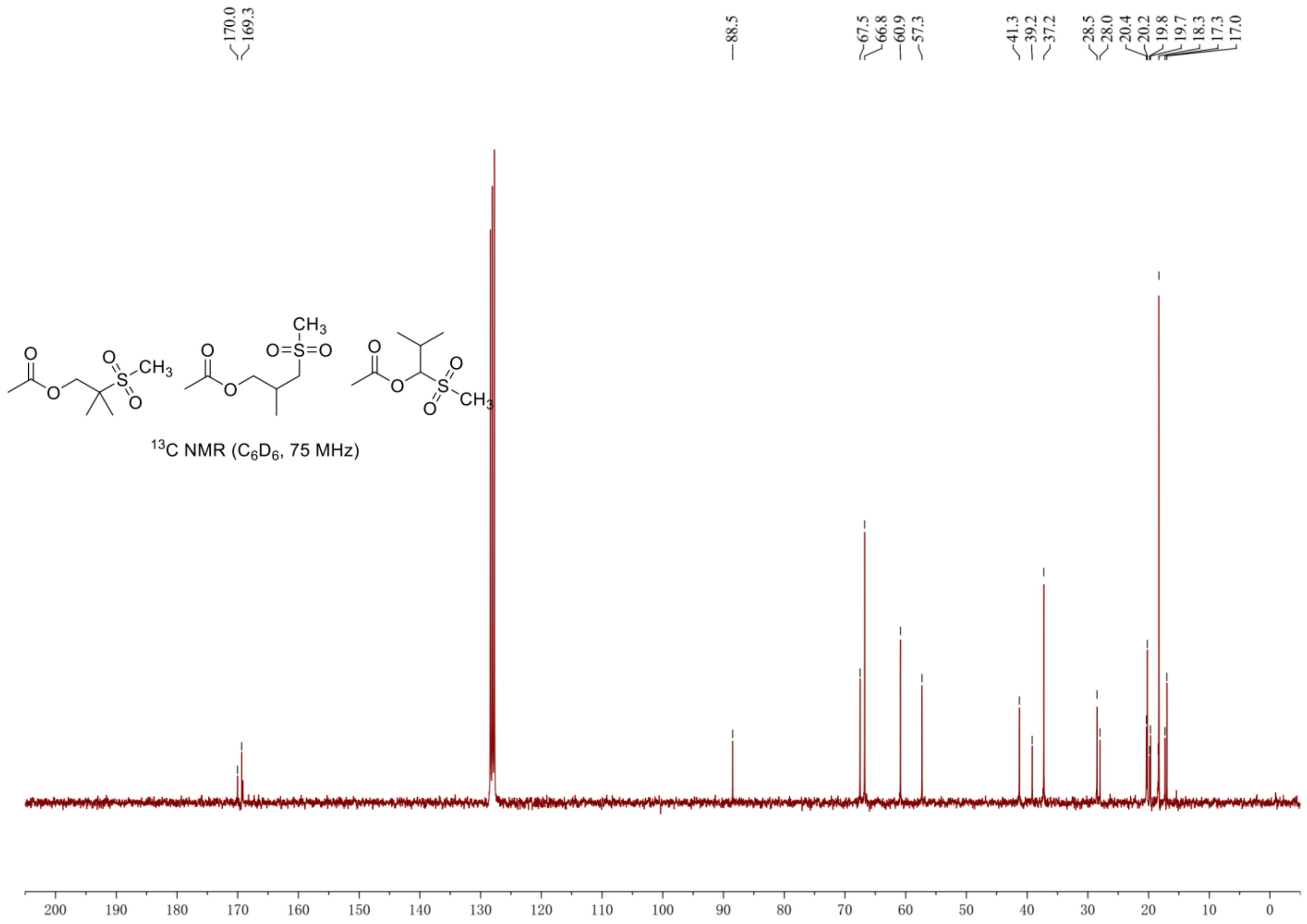

Go back to table of contents

5-(Allylsulfonyl)-4-methylpentanenitrile (18a)

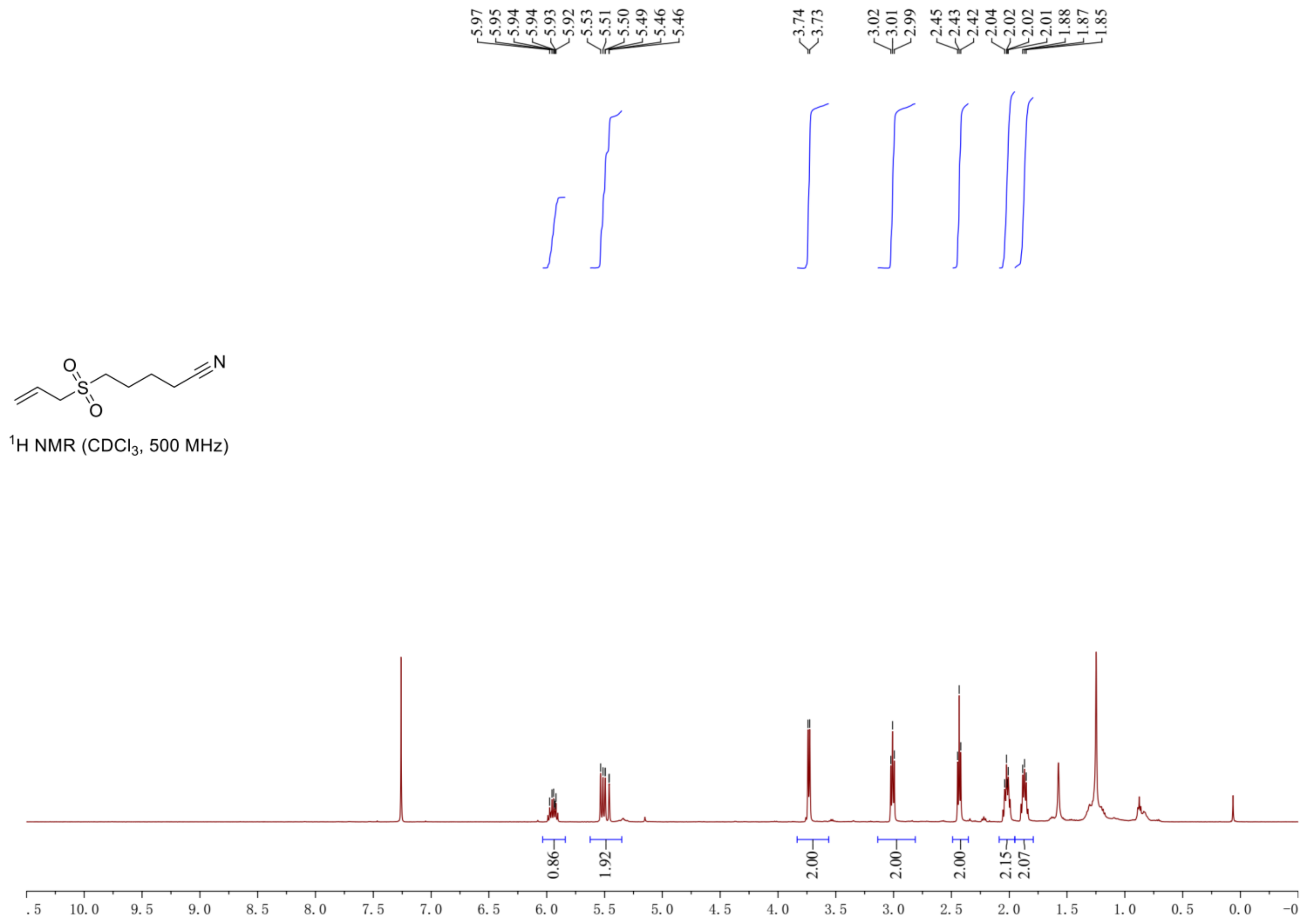

Go back to table of contents

5-(Allylsulfonyl)-4-methylpentanenitrile (18a)

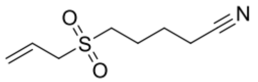

^{13}C NMR ($CDCl_3$, 125 MHz)

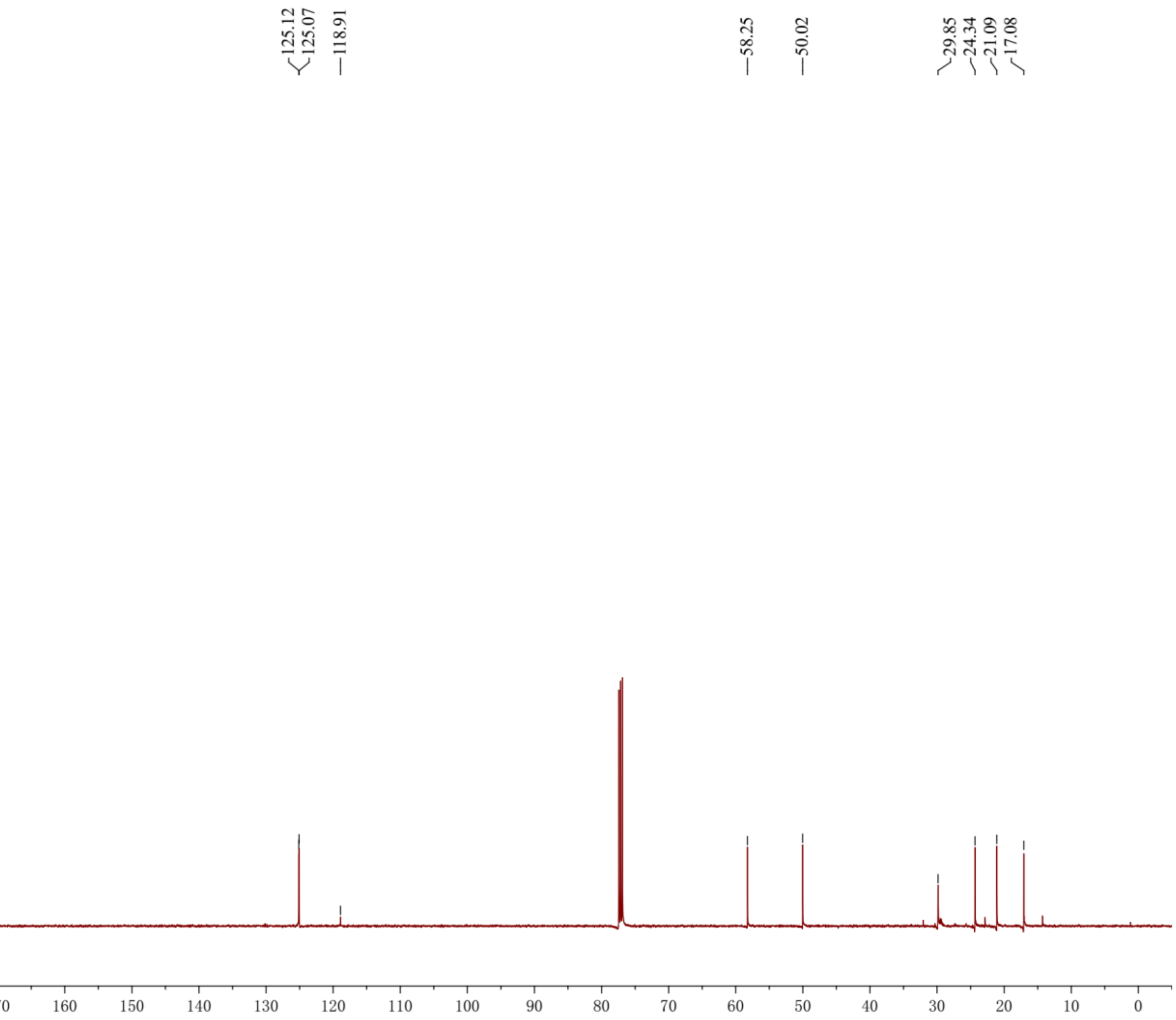

Go back to table of contents

4-(Allylsulfonyl)-4-methylpentanenitrile (18b)

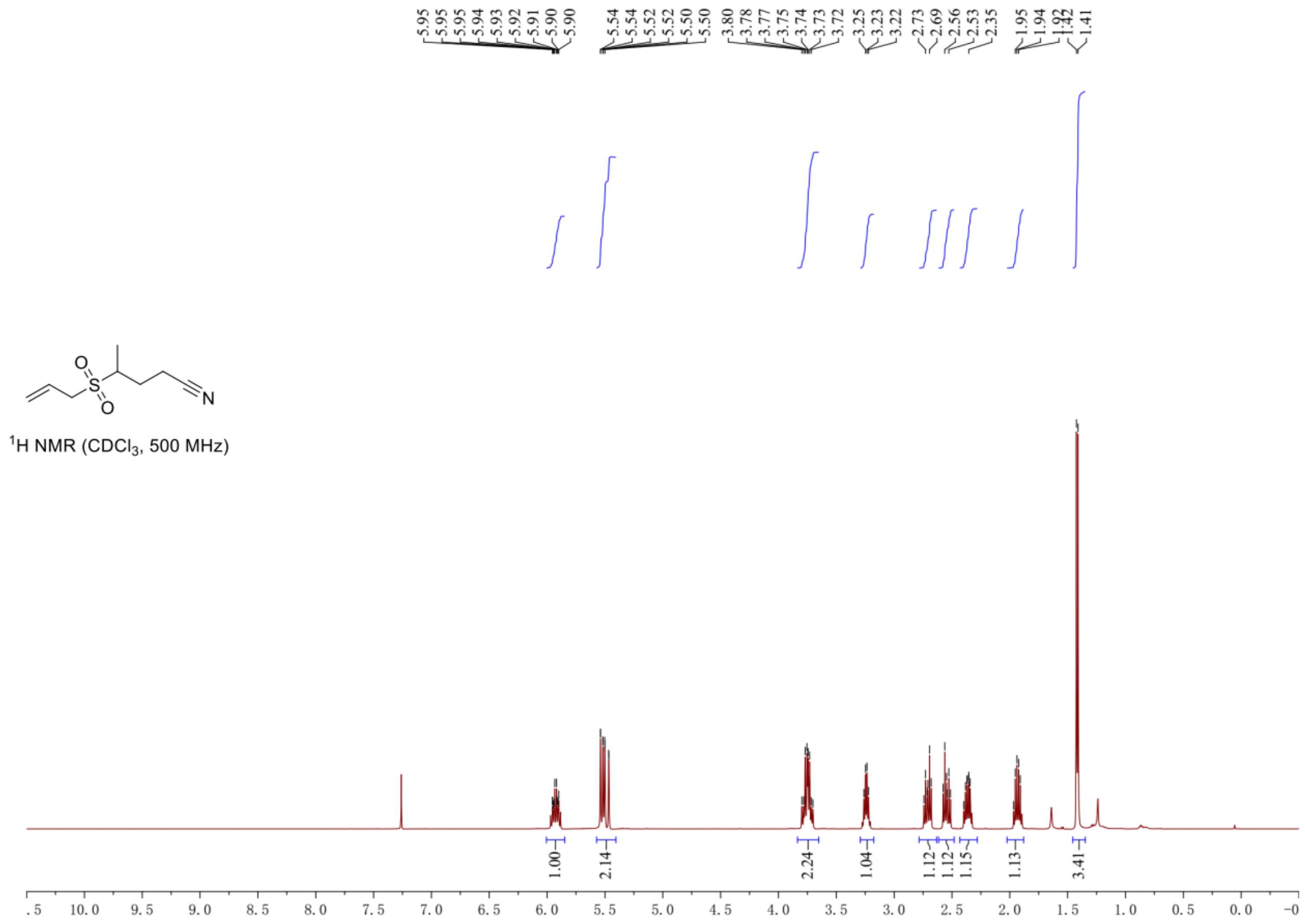

Go back to table of contents

4-(Allylsulfonyl)-4-methylpentanenitrile (18b)

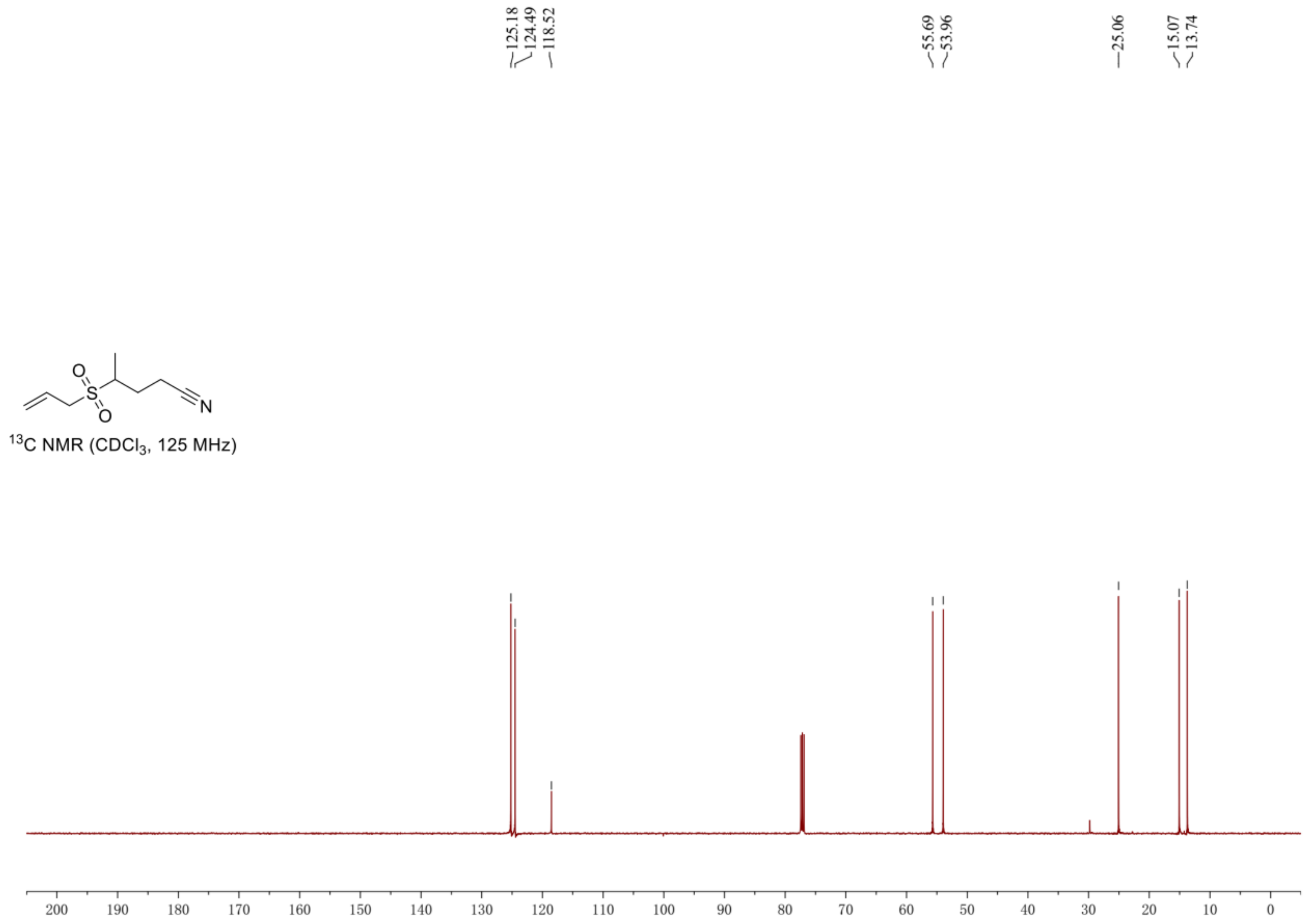

Go back to table of contents

(1,2-*cis*)-2-(allylsulfonyl)cyclopentane-1-carbonitrile (19a)

^{1}H NMR ($CDCl_3$, 500 MHz)

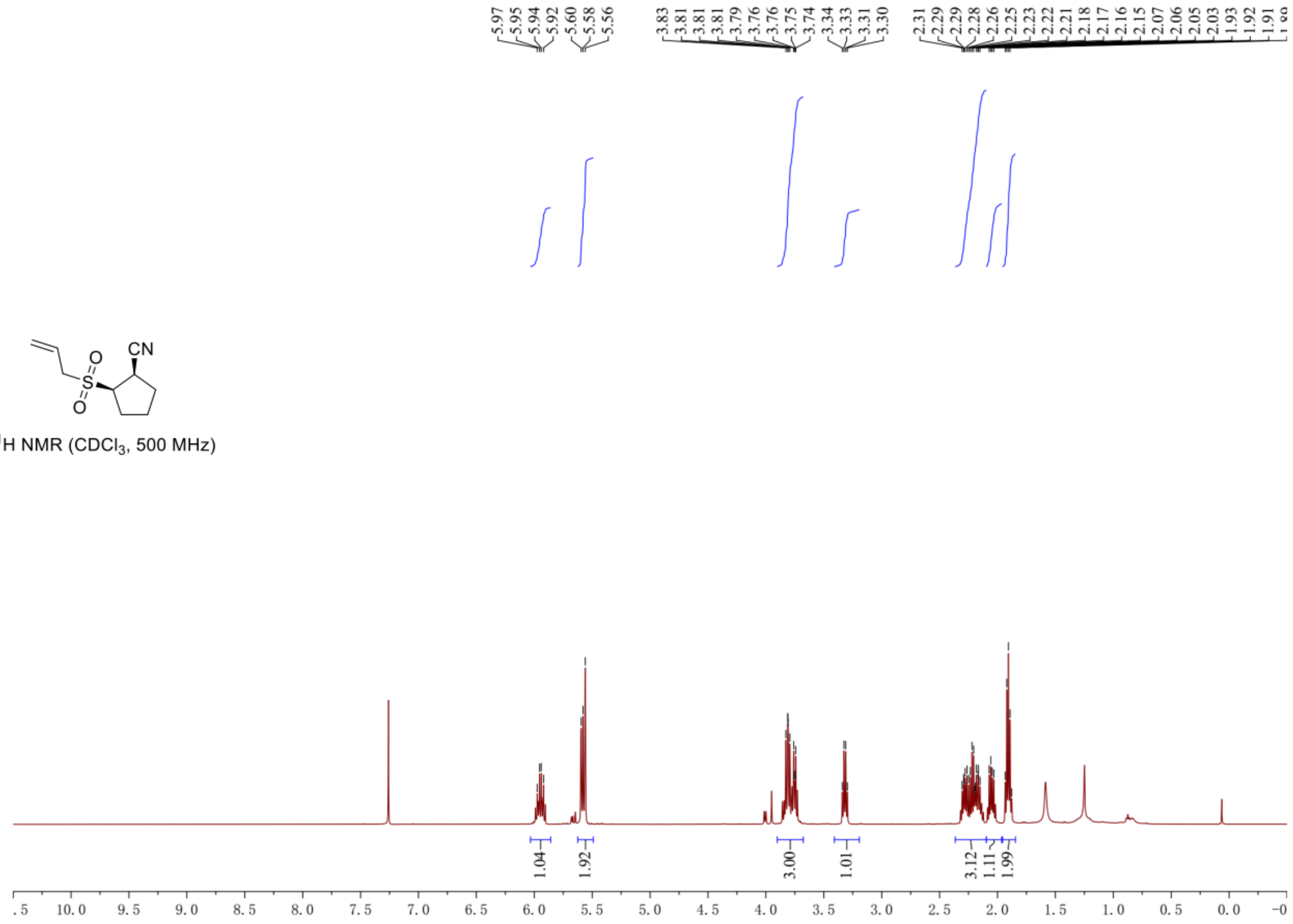

Go back to table of contents

(1,2-*cis*)-2-(allylsulfonyl)cyclopentane-1-carbonitrile (19a)

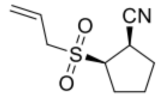

^{13}C NMR (CDCl$_3$, 125 MHz)

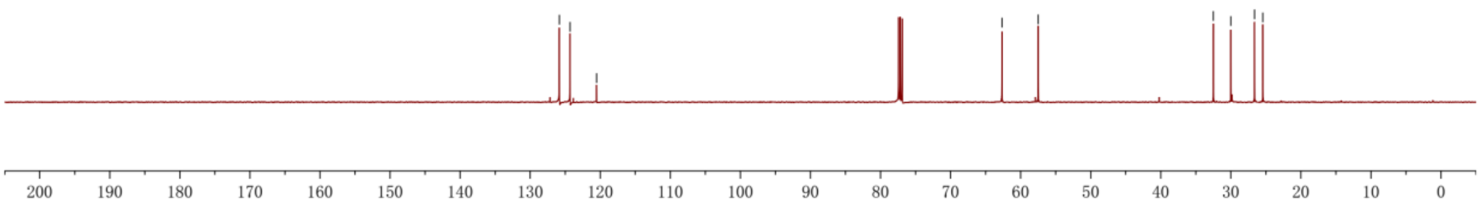

Go back to table of contents

(1,3-*trans*)-2-(allylsulfonyl)cyclopentane-1-carbonitrile (19b)

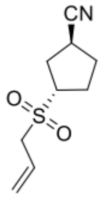

^{1}H NMR ($CDCl_3$, 500 MHz)

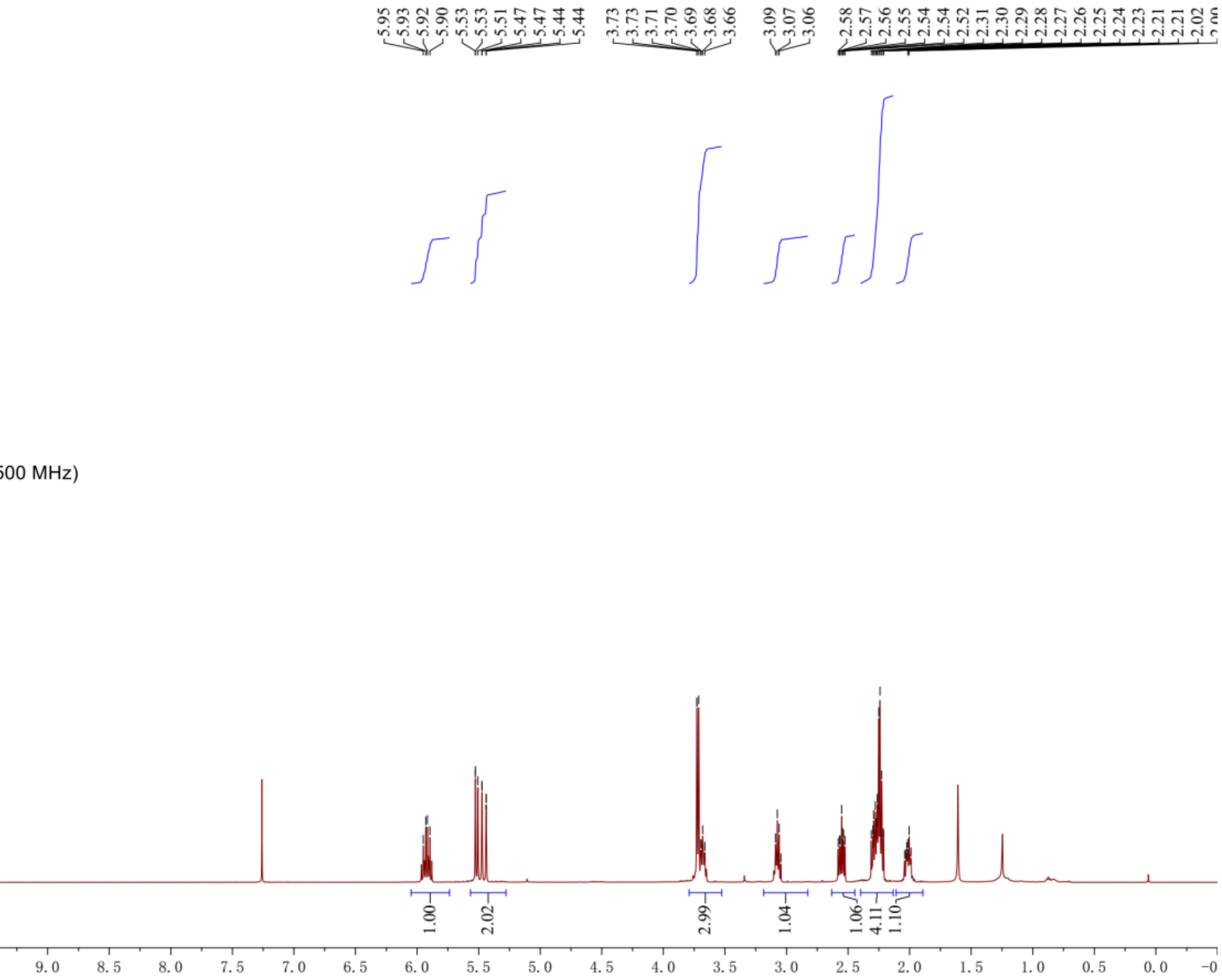

Go back to table of contents

(1,3-*trans*)-2-(allylsulfonyl)cyclopentane-1-carbonitrile (19b)

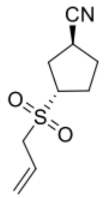

^{13}C NMR ($CDCl_3$, 125 MHz)

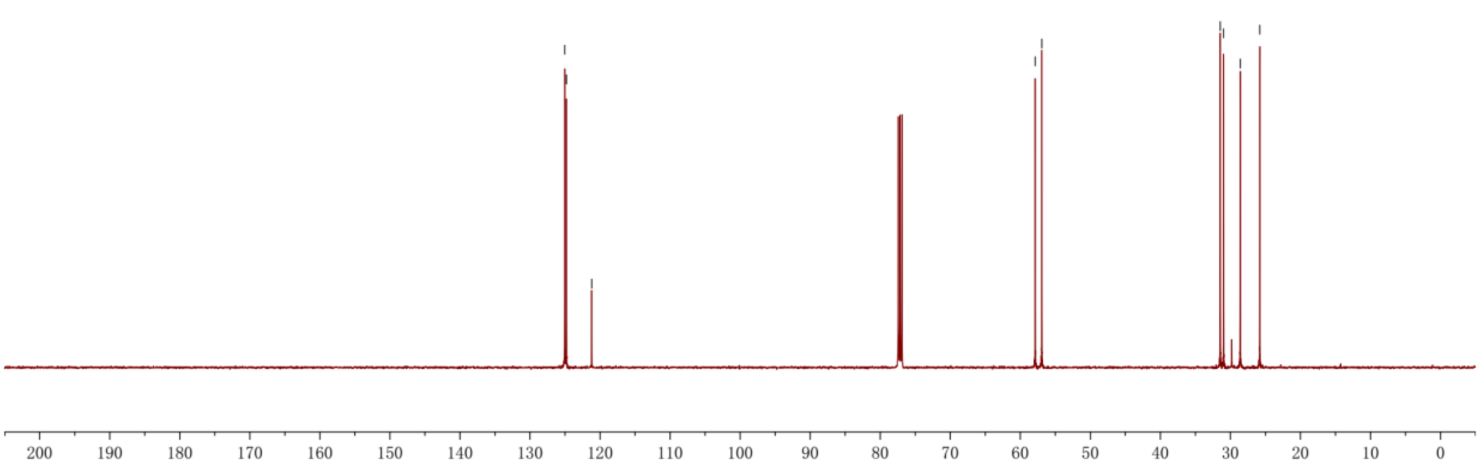

Go back to table of contents

(1,3-*cis*)-2-(allylsulfonyl)cyclopentane-1-carbonitrile (19c)

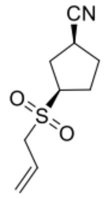

^{1}H NMR ($CDCl_3$, 500 MHz)

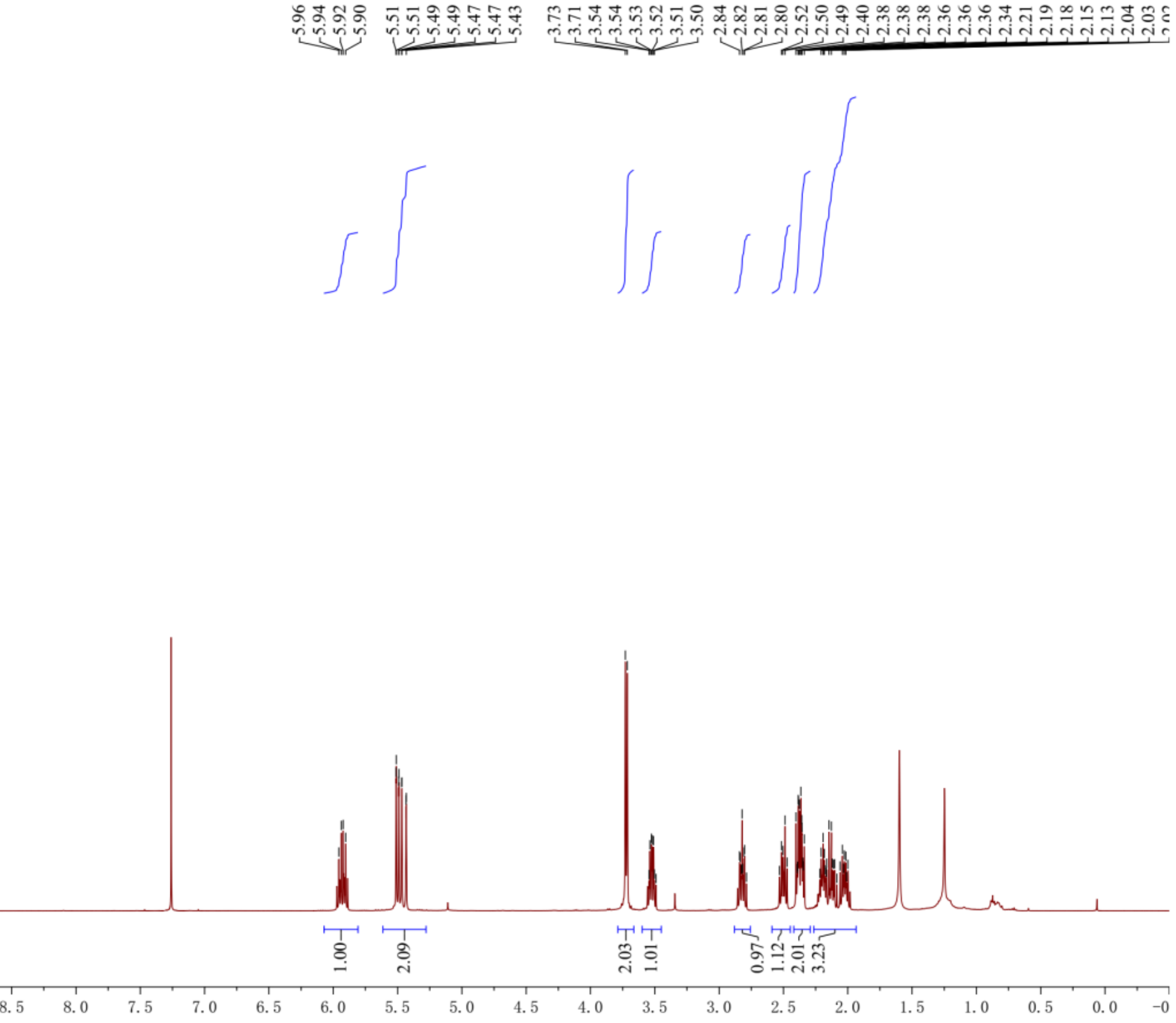

Go back to table of contents

(1,3-*cis*)-2-(allylsulfonyl)cyclopentane-1-carbonitrile (19c)

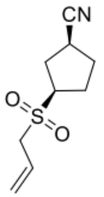

^{13}C NMR ($CDCl_3$, 125 MHz)

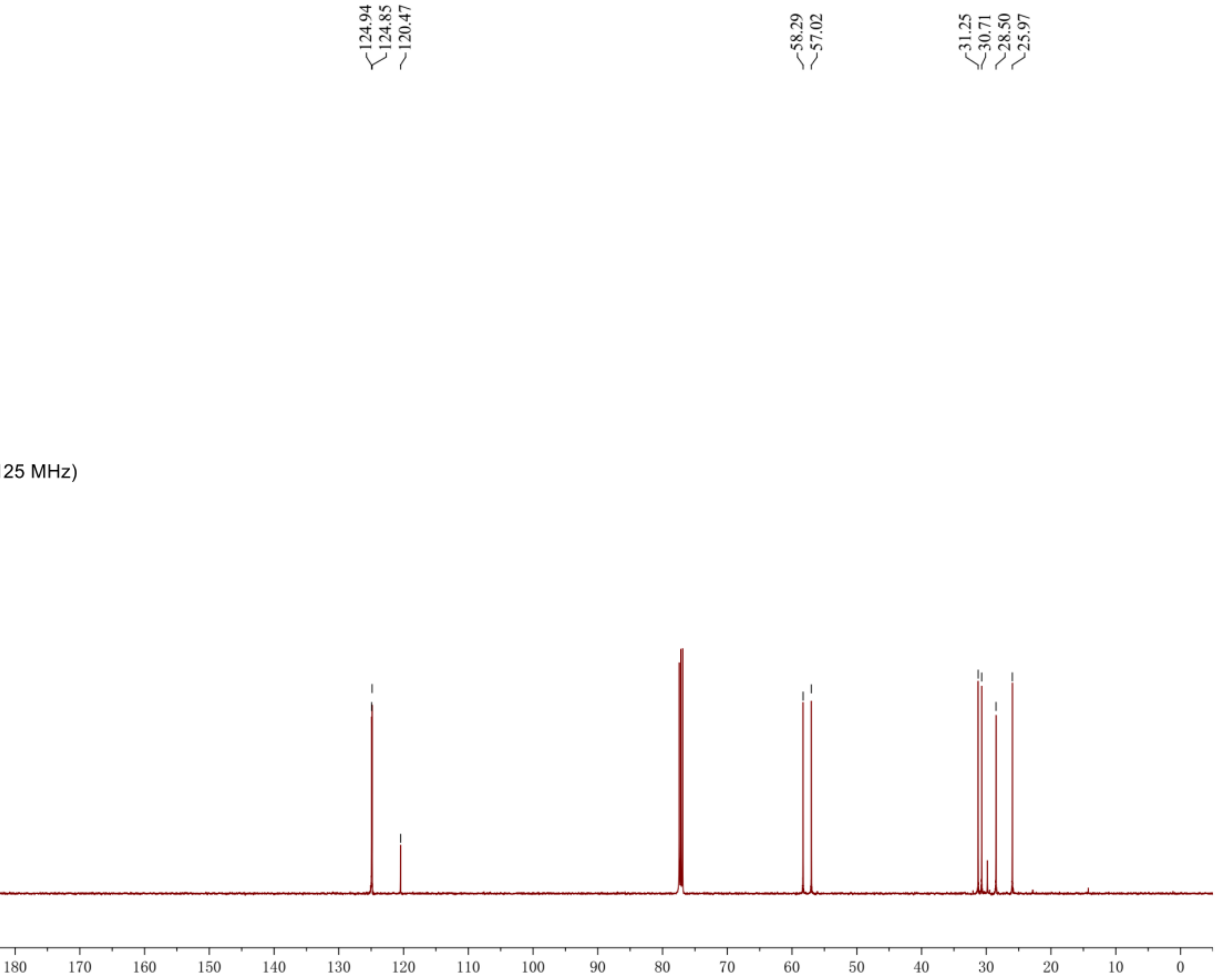

Go back to table of contents

1-(1-(Allylsulfonyl)ethyl)-4-fluorobenzene (20)

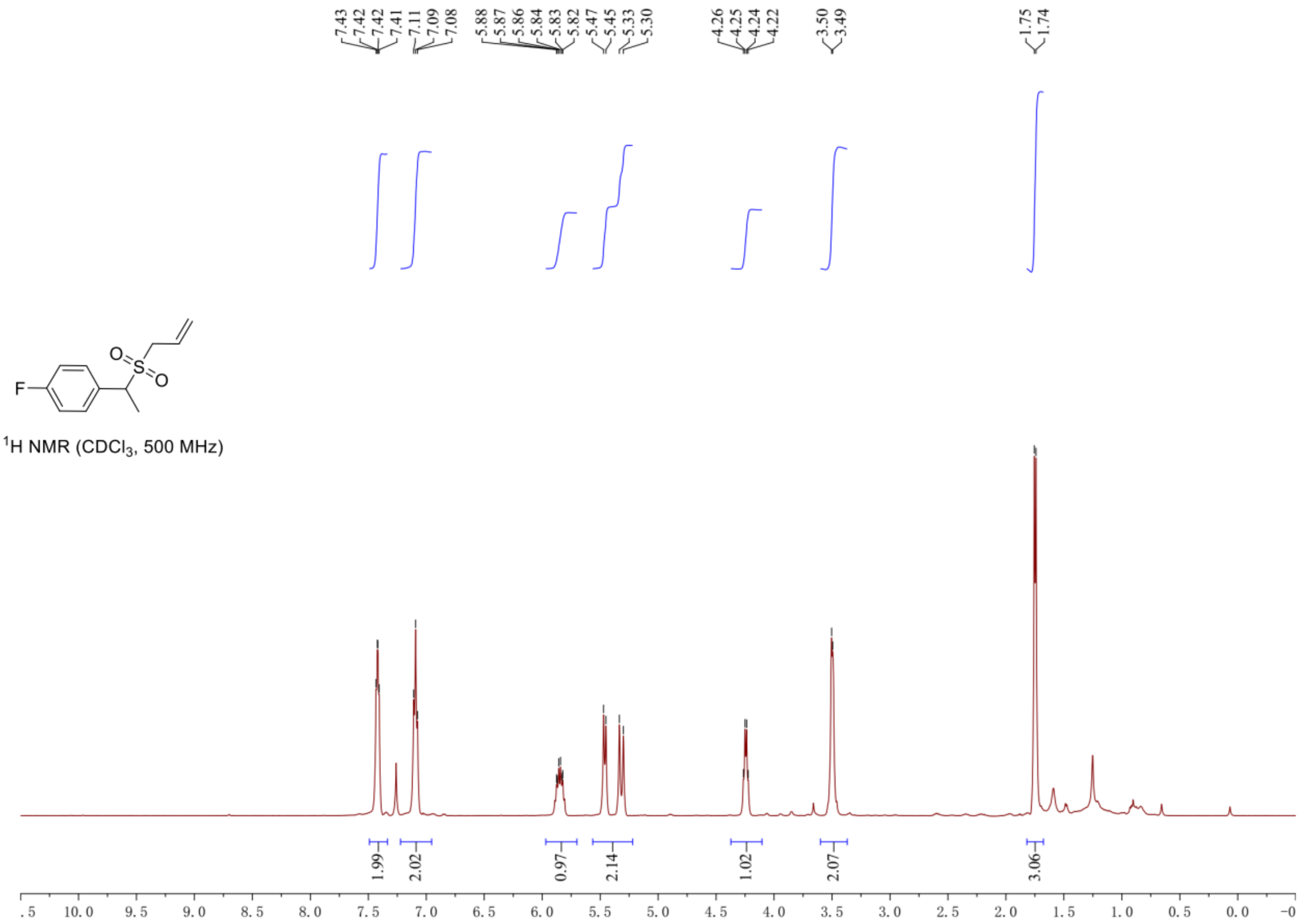

Go back to table of contents

trans-3-Methyl-1,2-oxathiolane 2-oxide (21a)

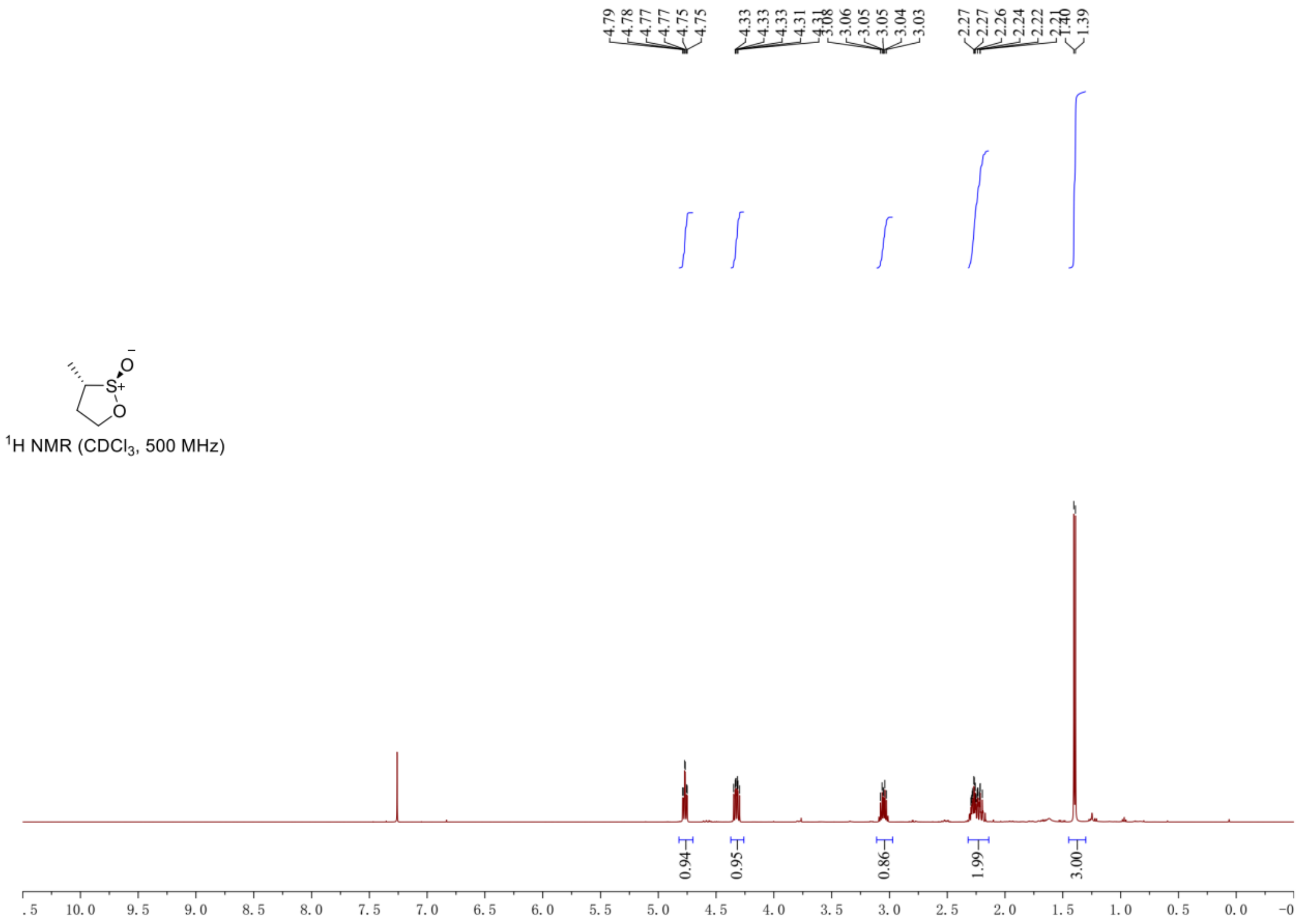

Go back to table of contents

trans-3-Methyl-1,2-oxathiolane 2-oxide (21a)

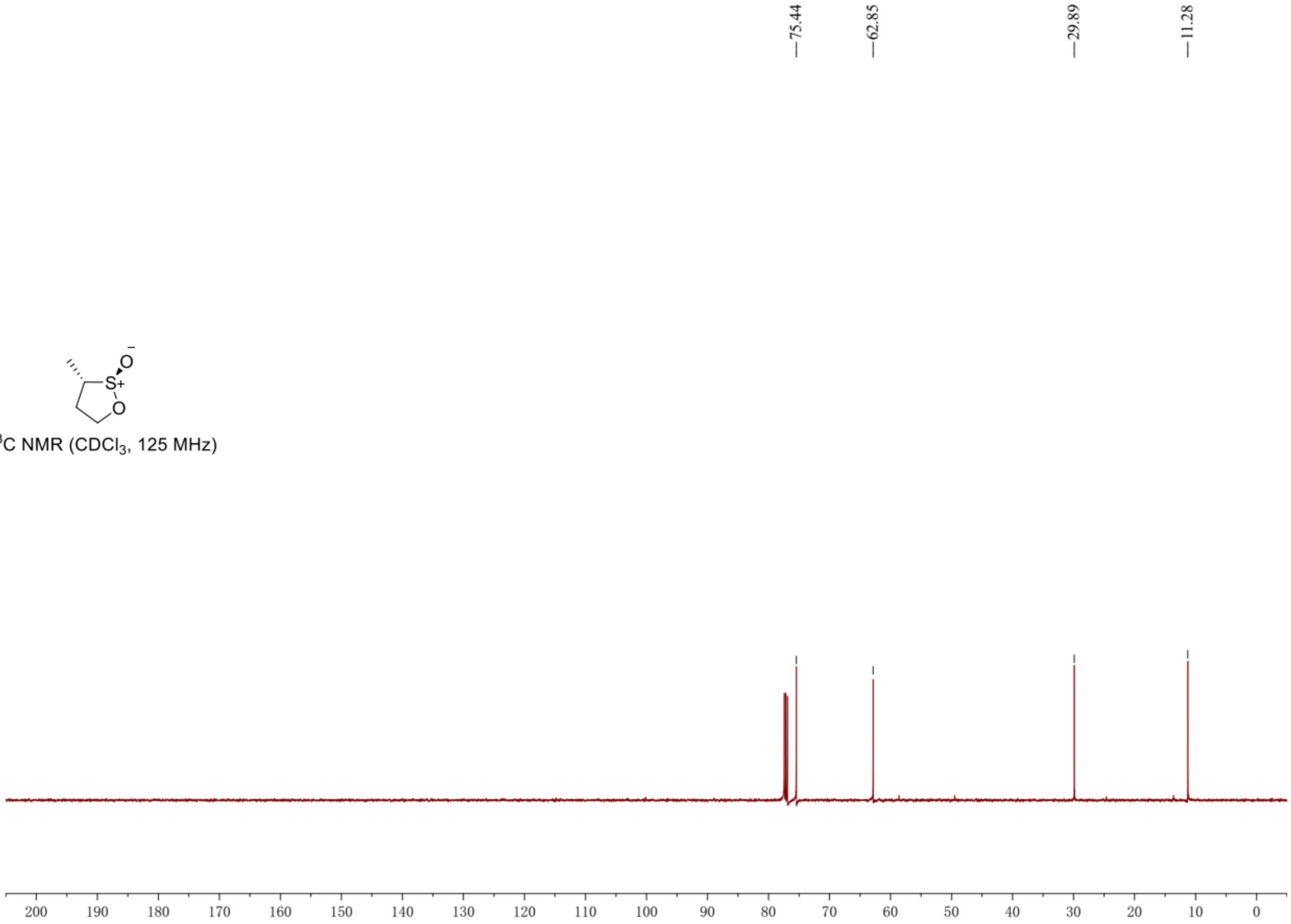

^{13}C NMR ($CDCl_3$, 125 MHz)

Go back to table of contents

cis-3-Methyl-1,2-oxathiolane 2-oxide (21b)

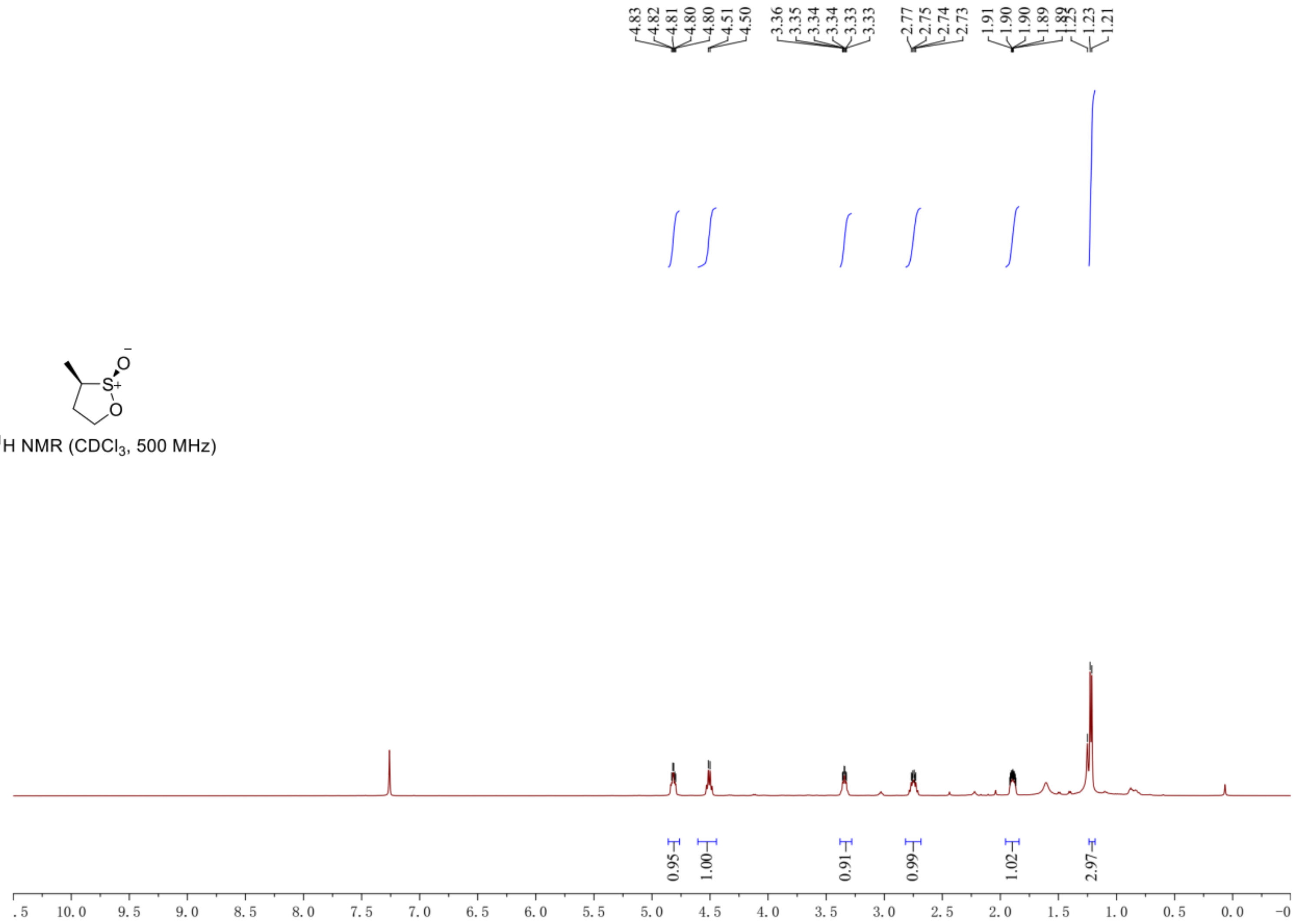

^{1}H NMR ($CDCl_3$, 500 MHz)

Go back to table of contents

cis-3-Methyl-1,2-oxathiolane 2-oxide (21b)

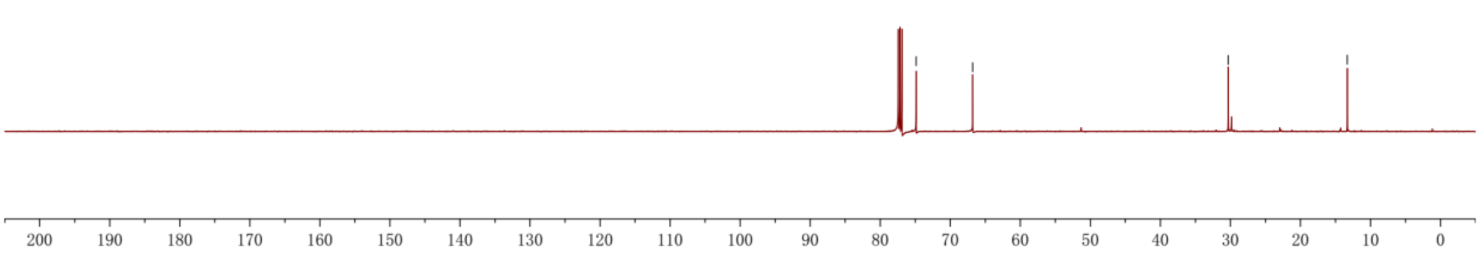

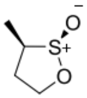

^{13}C NMR ($CDCl_3$, 125 MHz)

Go back to table of contents

N-(4-(Allylsulfonyl)butyl)-2,2,2-trifluoroacetamide (22a)

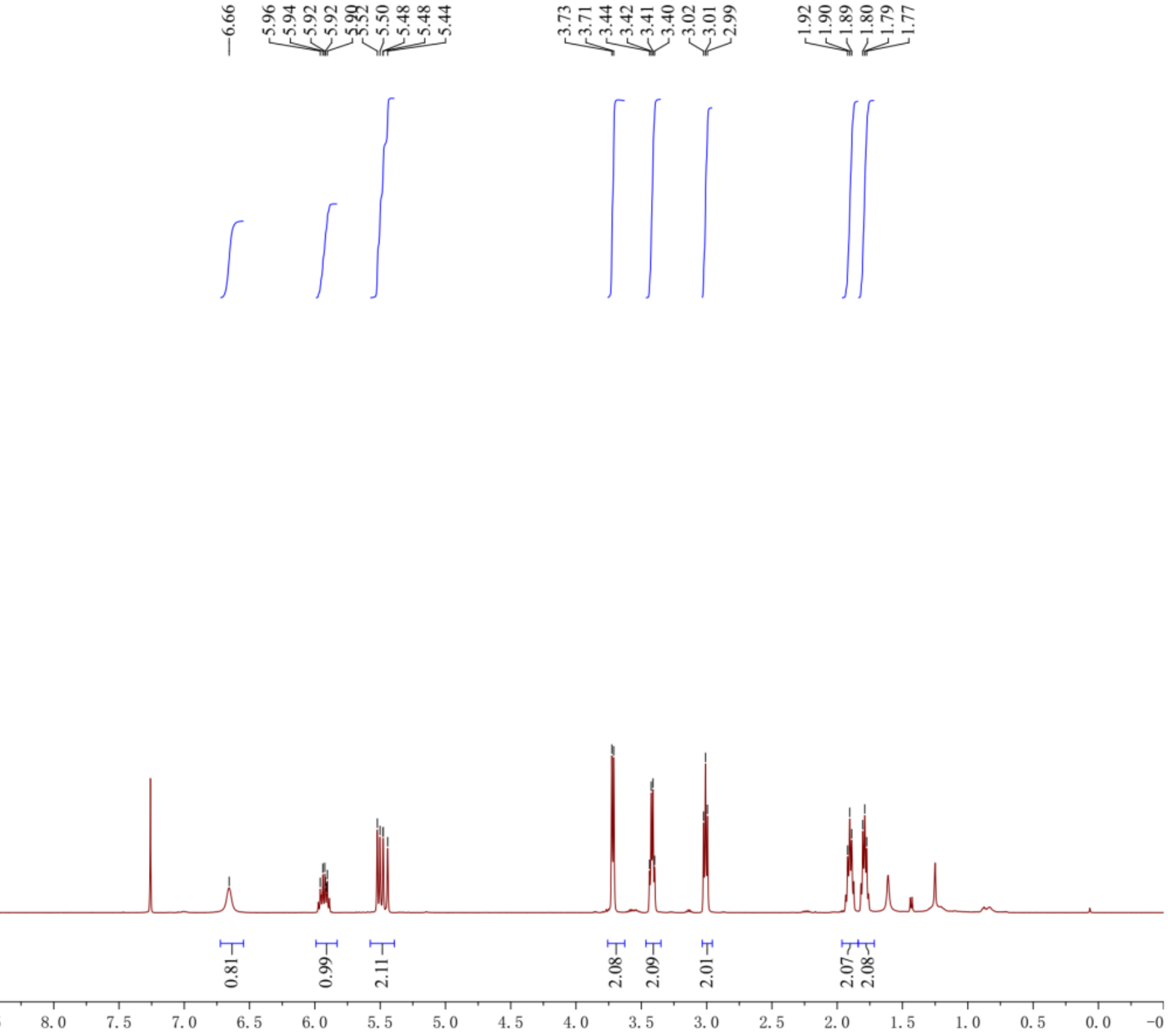

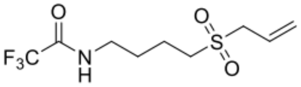

^{1}H NMR ($CDCl_3$, 500 MHz)

Go back to table of contents

_N_-(4-(Allylsulfonyl)butyl)-2,2,2-trifluoroacetamide (22a)

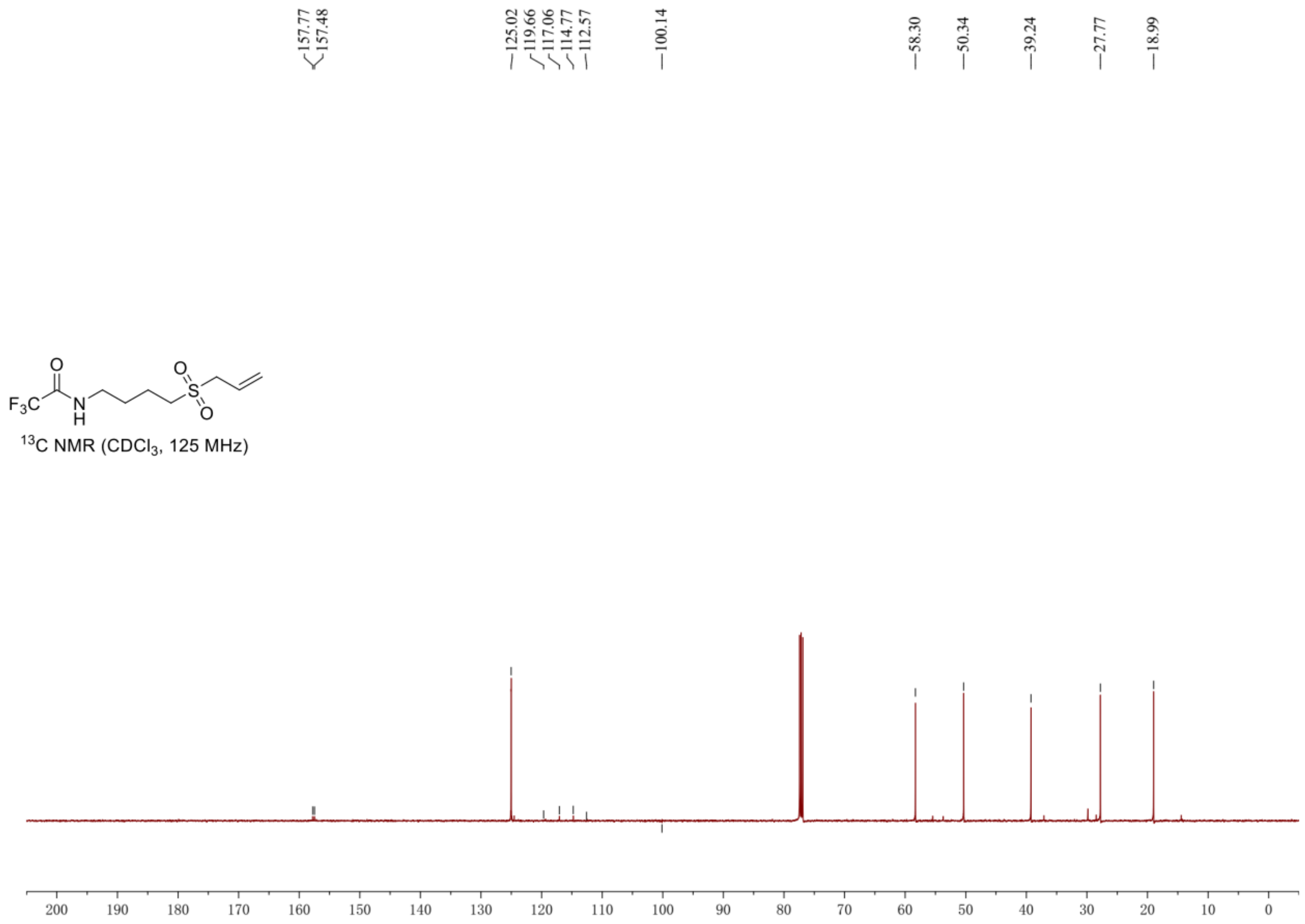

Go back to table of contents

N-(3-(Allylsulfonyl)butyl)-2,2,2-trifluoroacetamide (22b)

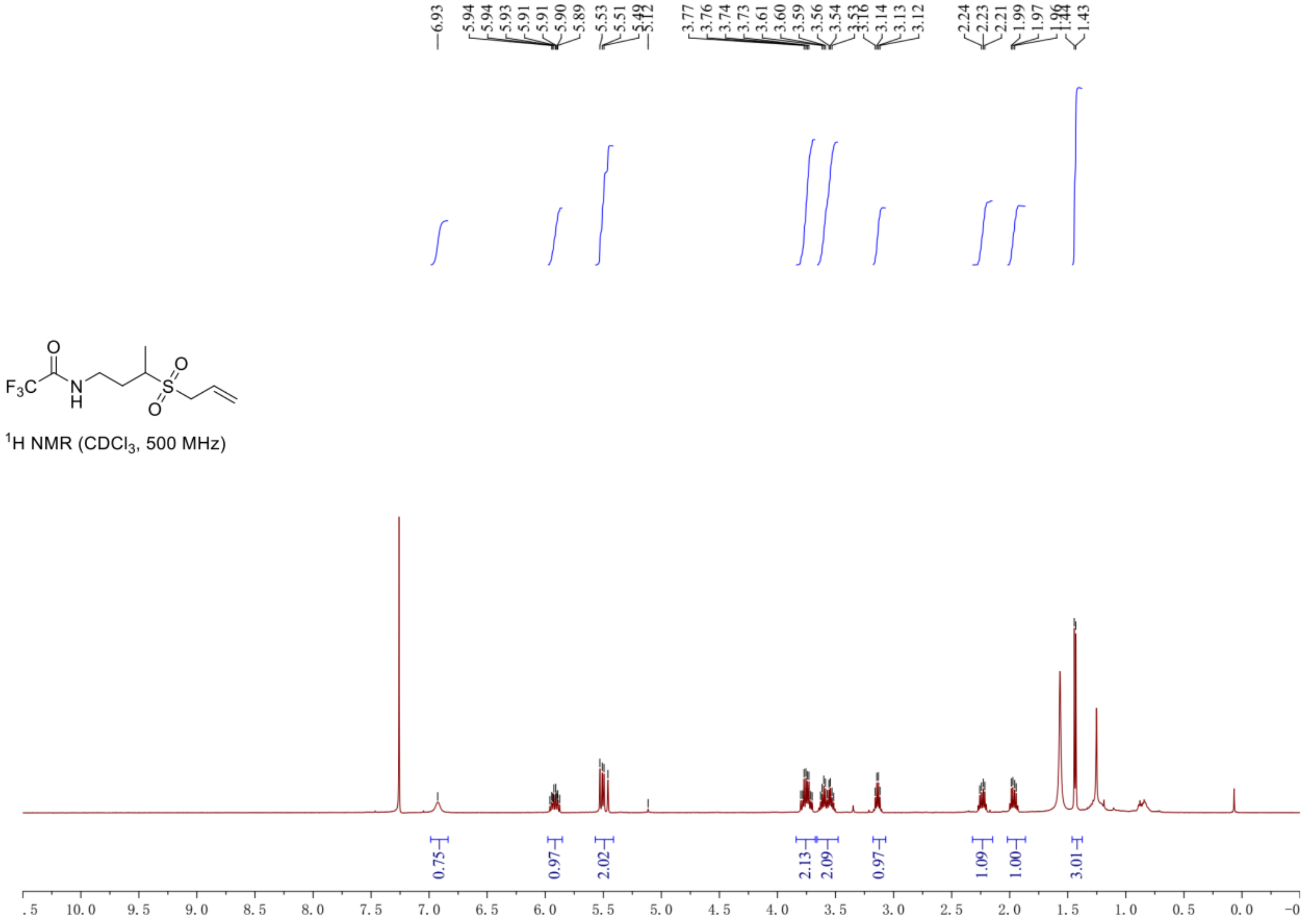

Go back to table of contents

N-(3-(Allylsulfonyl)butyl)-2,2,2-trifluoroacetamide (22b)

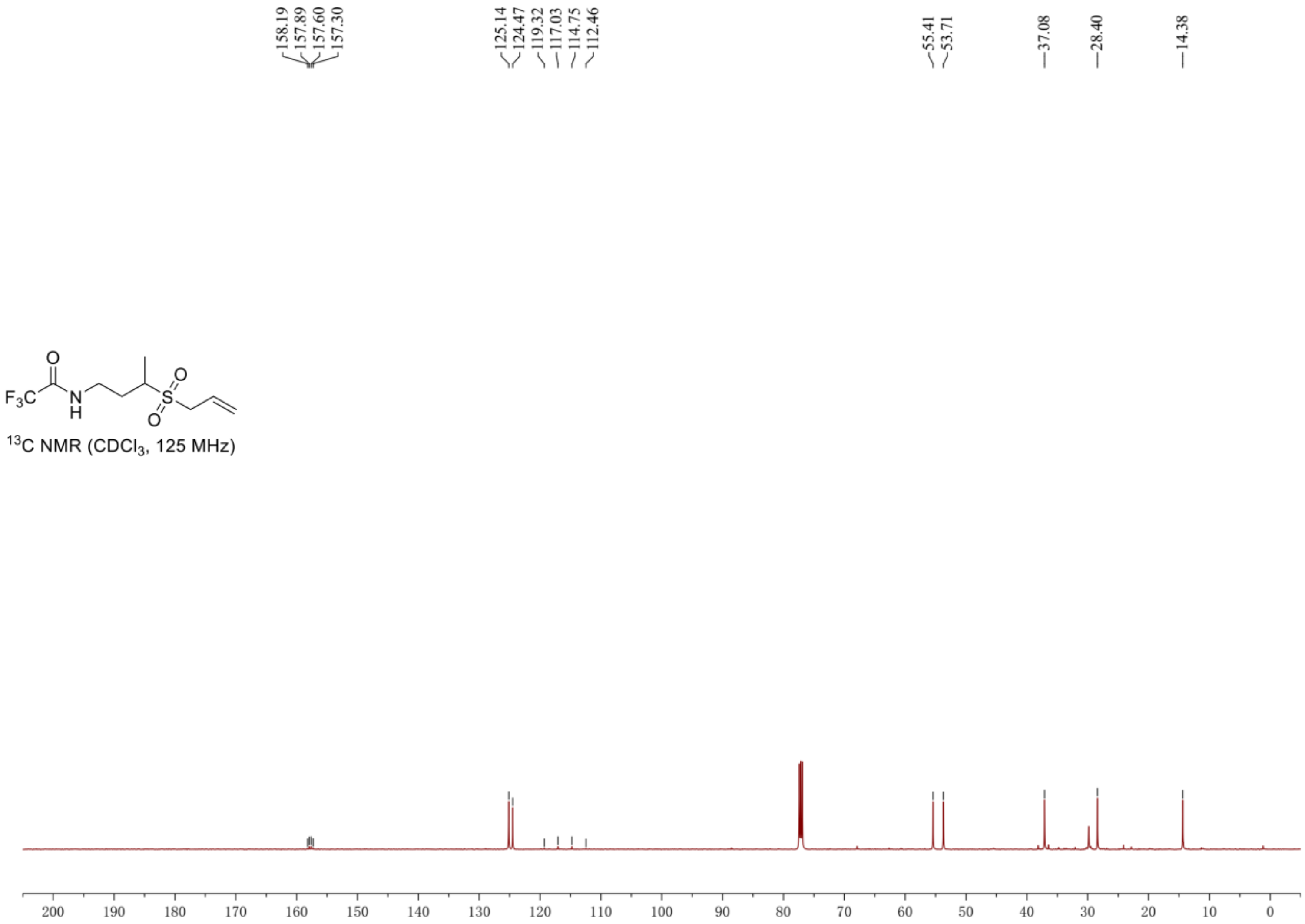

Go back to table of contents

N-(2-(Allylsulfonyl)butyl)-2,2,2-trifluoroacetamide (22c)

^{1}H NMR ($CDCl_3$, 500 MHz)

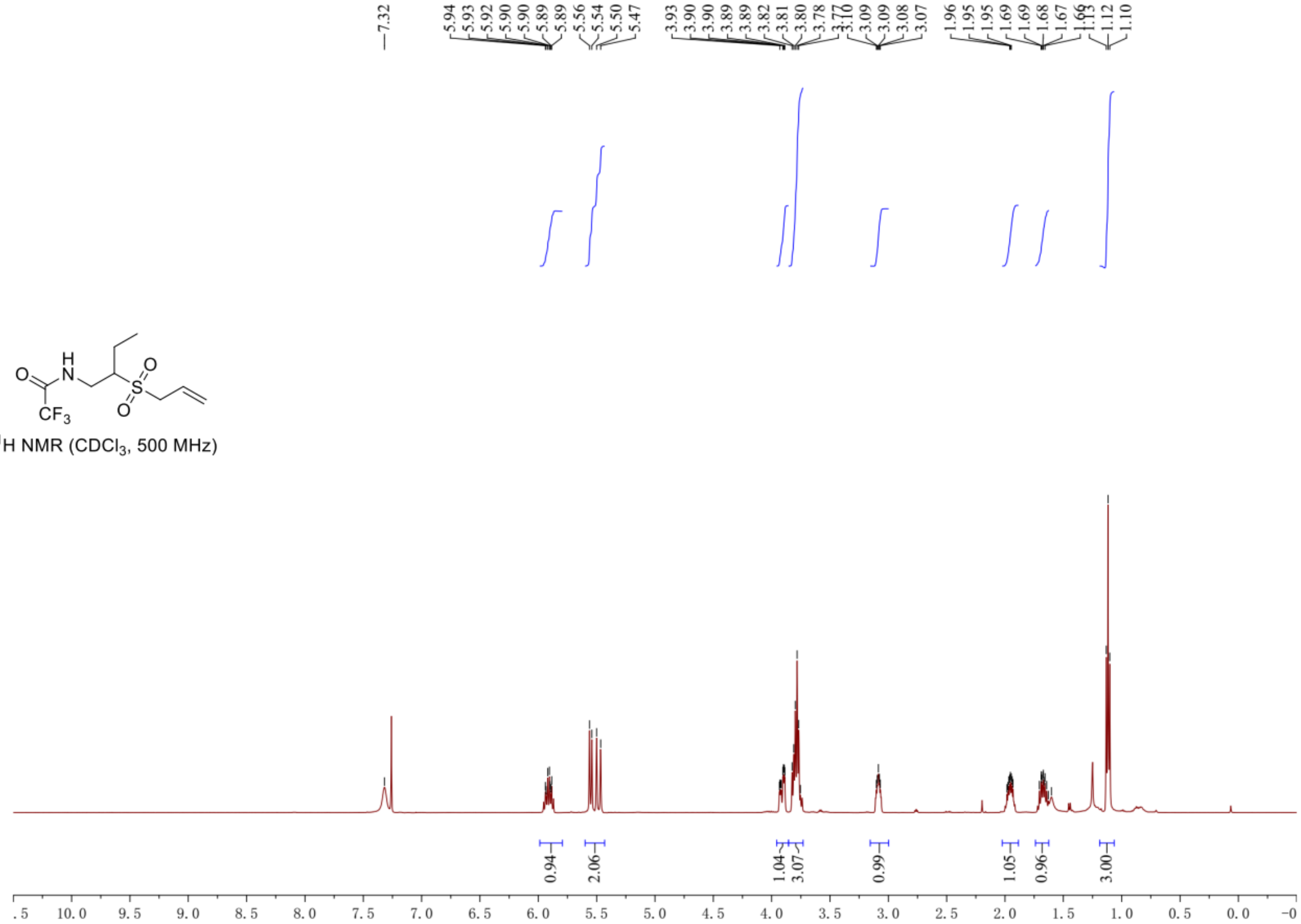

Go back to table of contents

N-(2-(Allylsulfonyl)butyl)-2,2,2-trifluoroacetamide (22c)

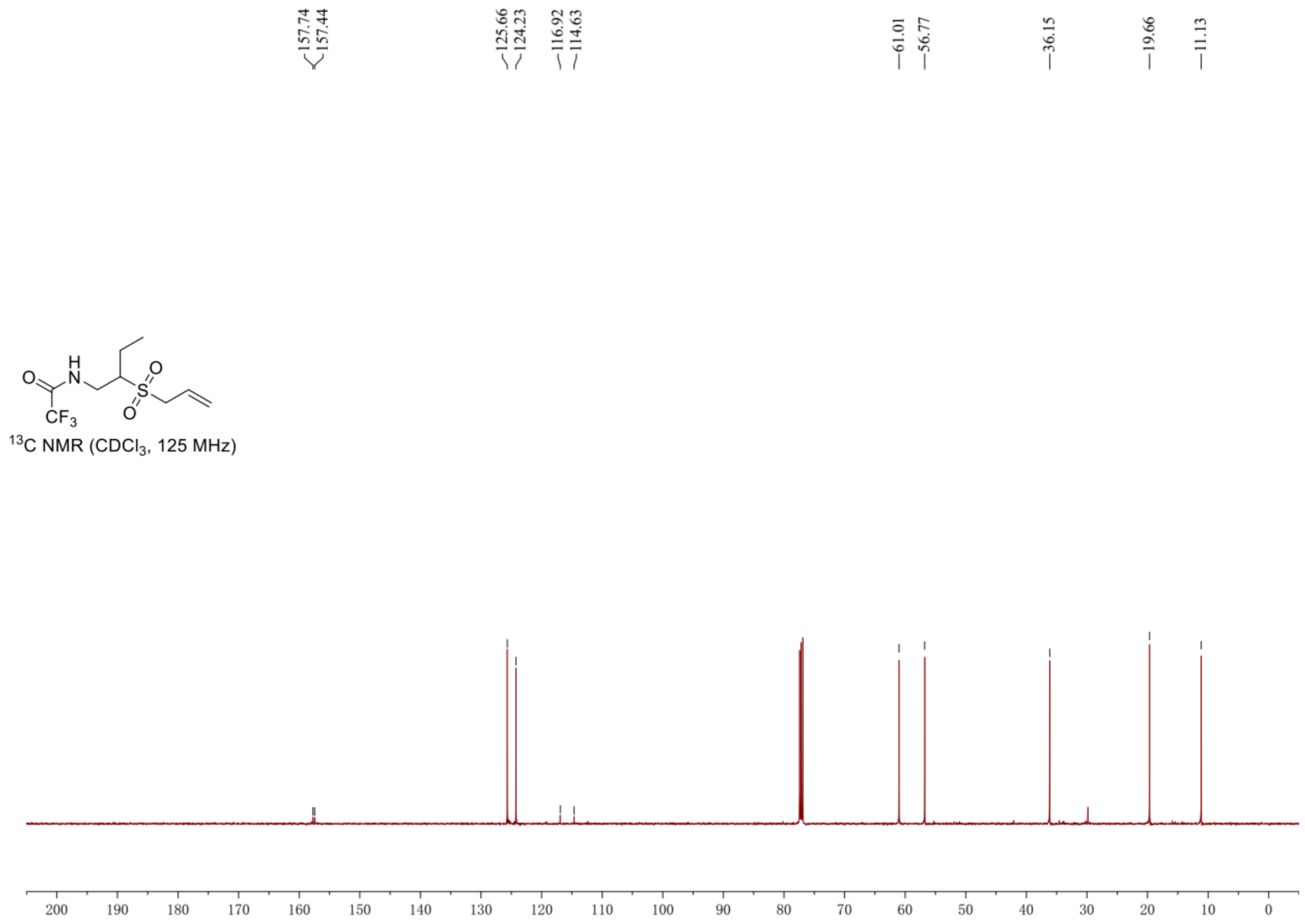

Go back to table of contents

Methyl *C*[4]-(allylsulfonyl)(2,2,2-trifluoroacetyl)-L-valinate (23)

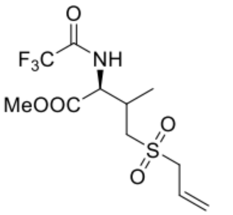

^{1}H NMR ($CDCl_3$, 500 MHz)

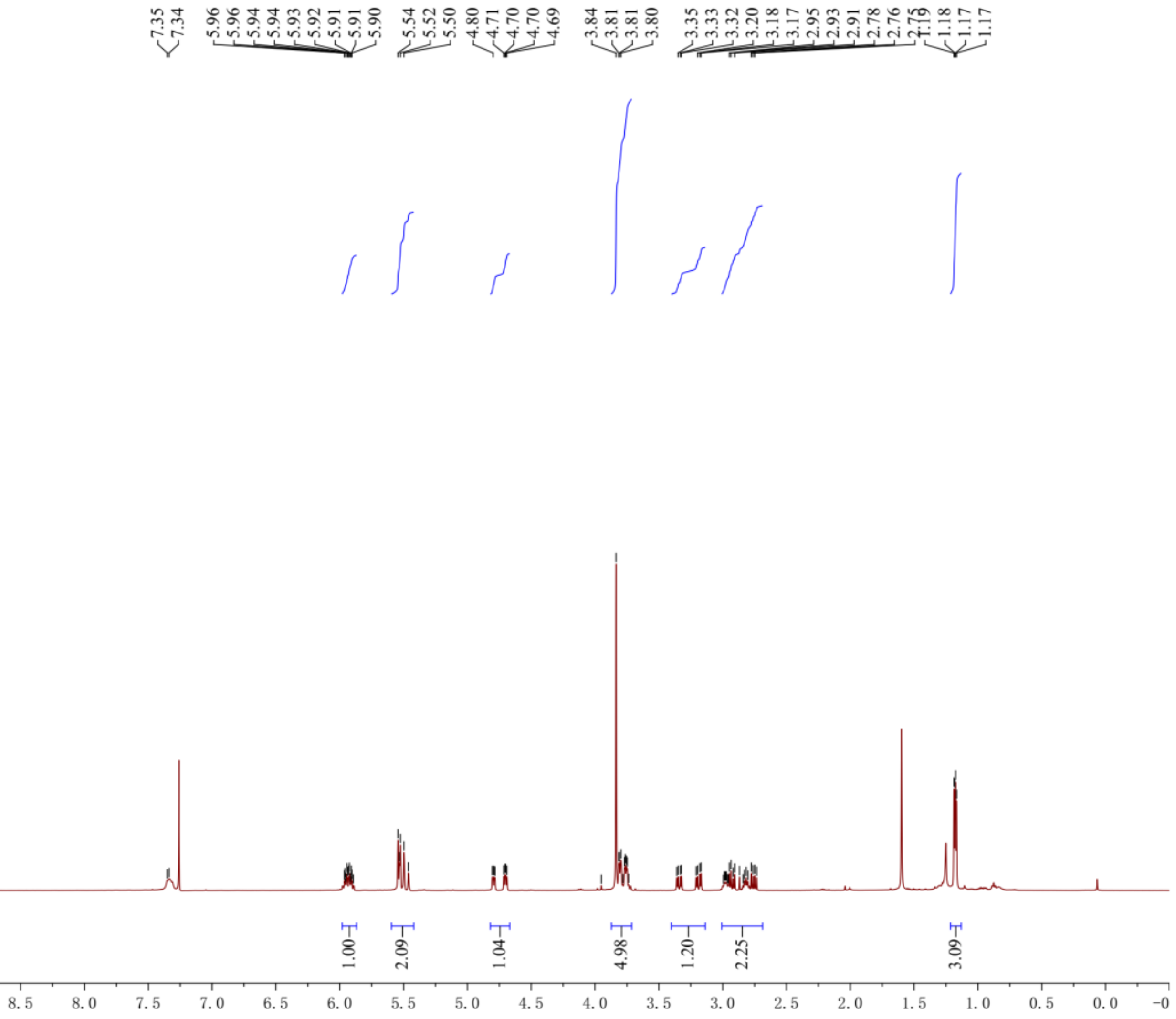

Go back to table of contents

Methyl C^4-(allylsulfonyl)(2,2,2-trifluoroacetyl)-L-valinate (23)

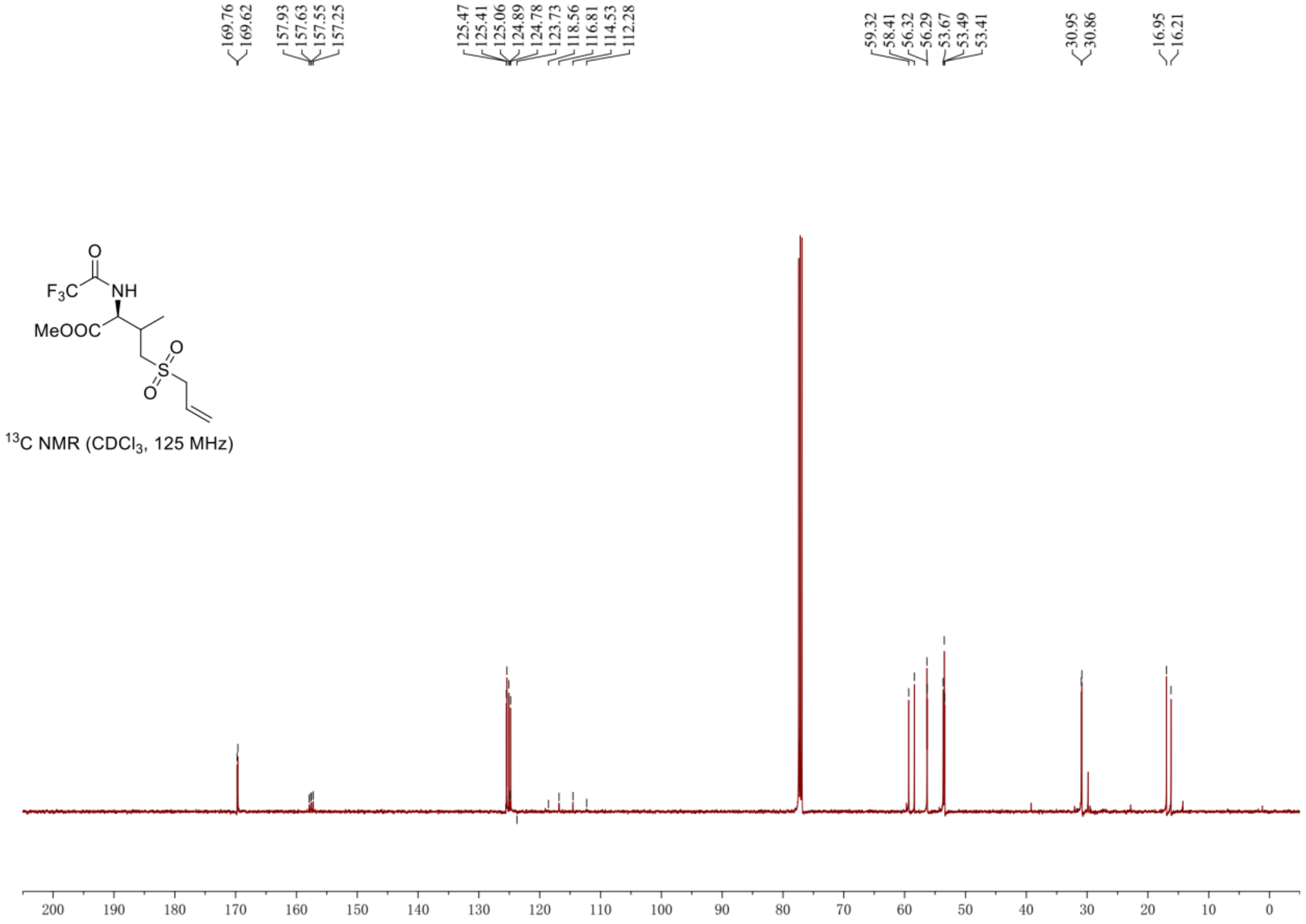

Go back to table of contents

Methyl 2-(4-(3-(allylsulfonyl)-2-methylpropyl)phenyl)propanoate (24a)

Methyl 2-(4-(2-(allylsulfonyl)-2-methylpropyl)phenyl)propanoate (24b)

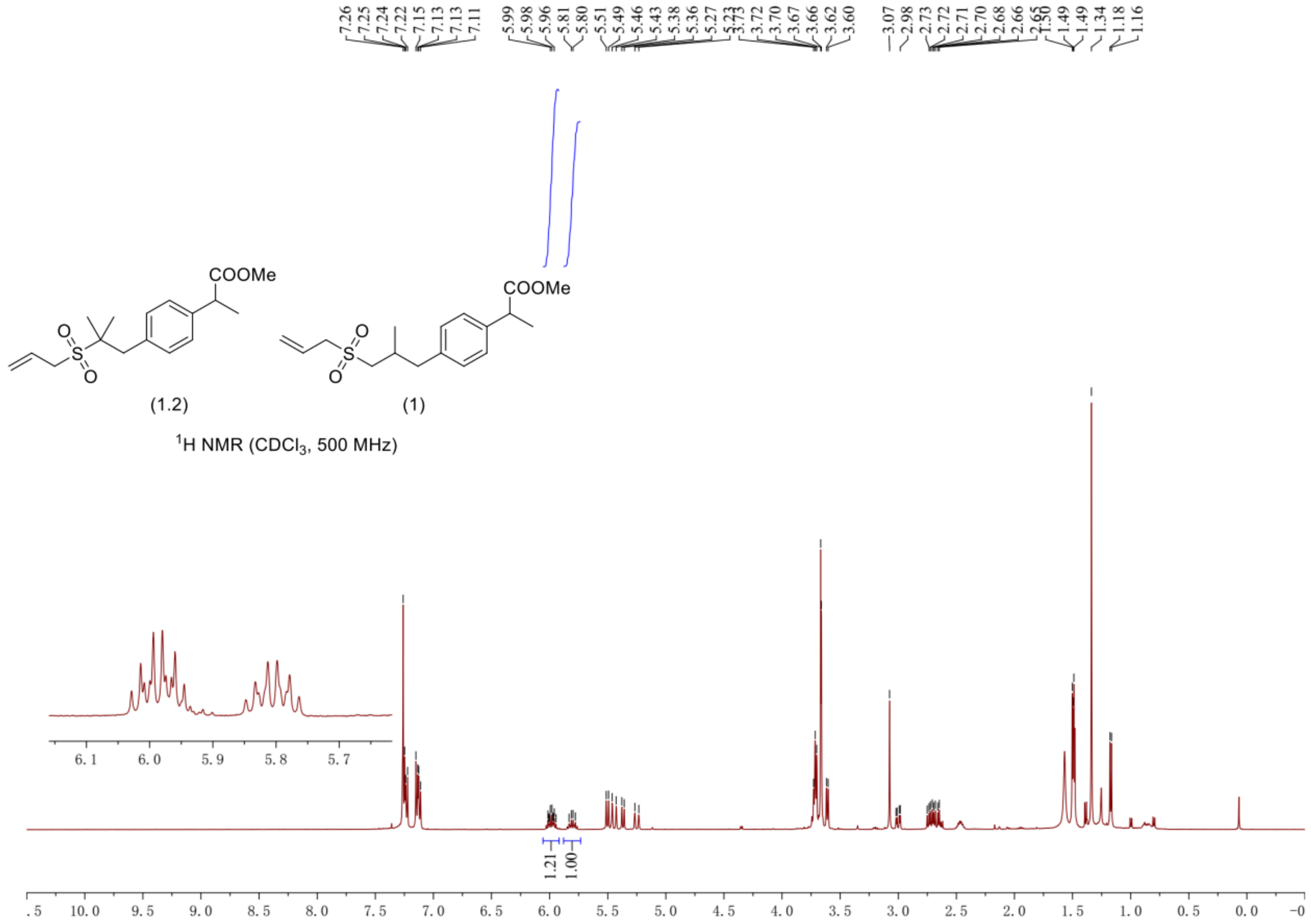

Go back to table of contents

Methyl 2-(4-(3-(allylsulfonyl)-2-methylpropyl)phenyl)propanoate (24a)

Methyl 2-(4-(2-(allylsulfonyl)-2-methylpropyl)phenyl)propanoate (24b)

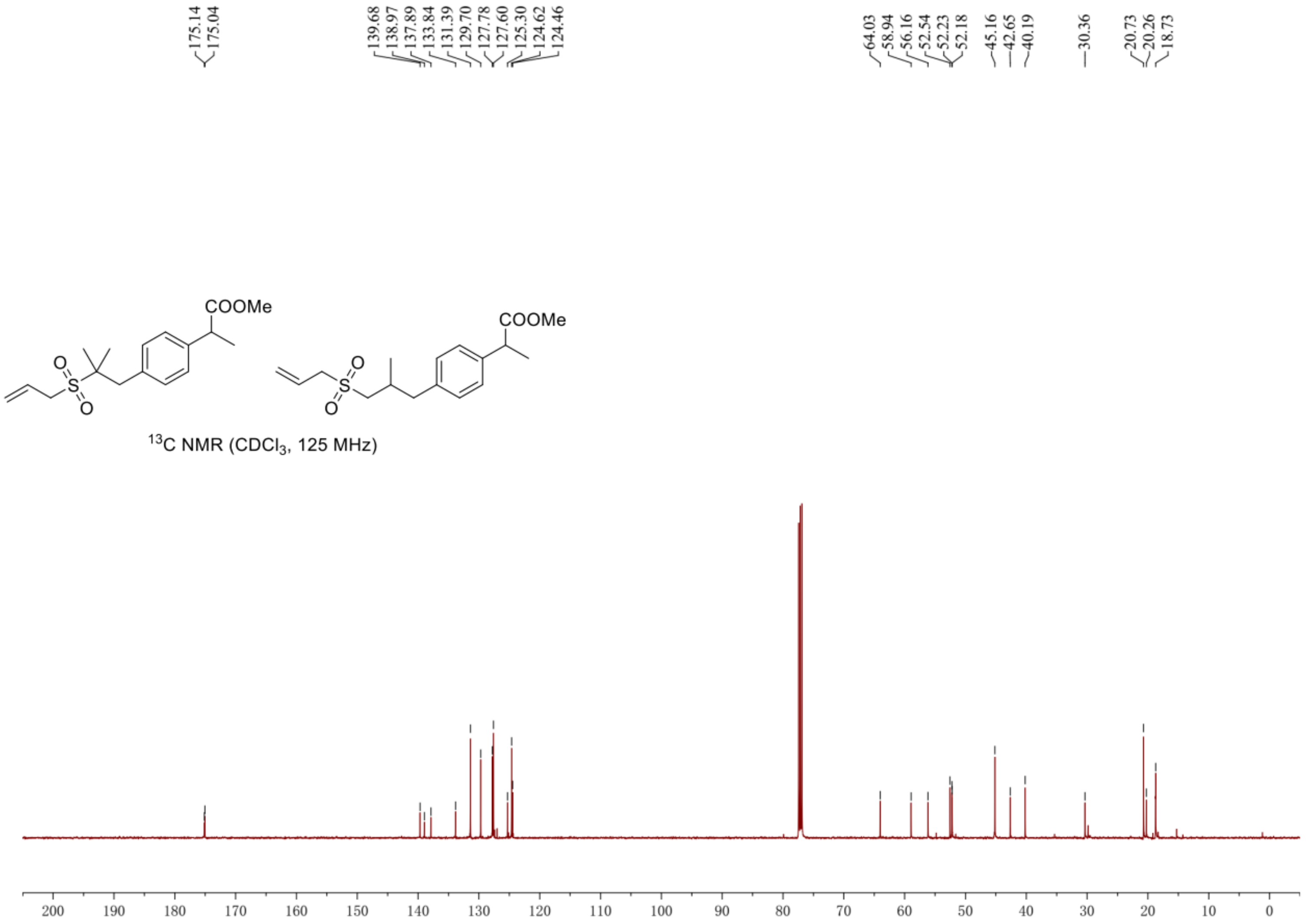

Go back to table of contents

2-(Allylsulfonyl)-1,4-dioxane (25)

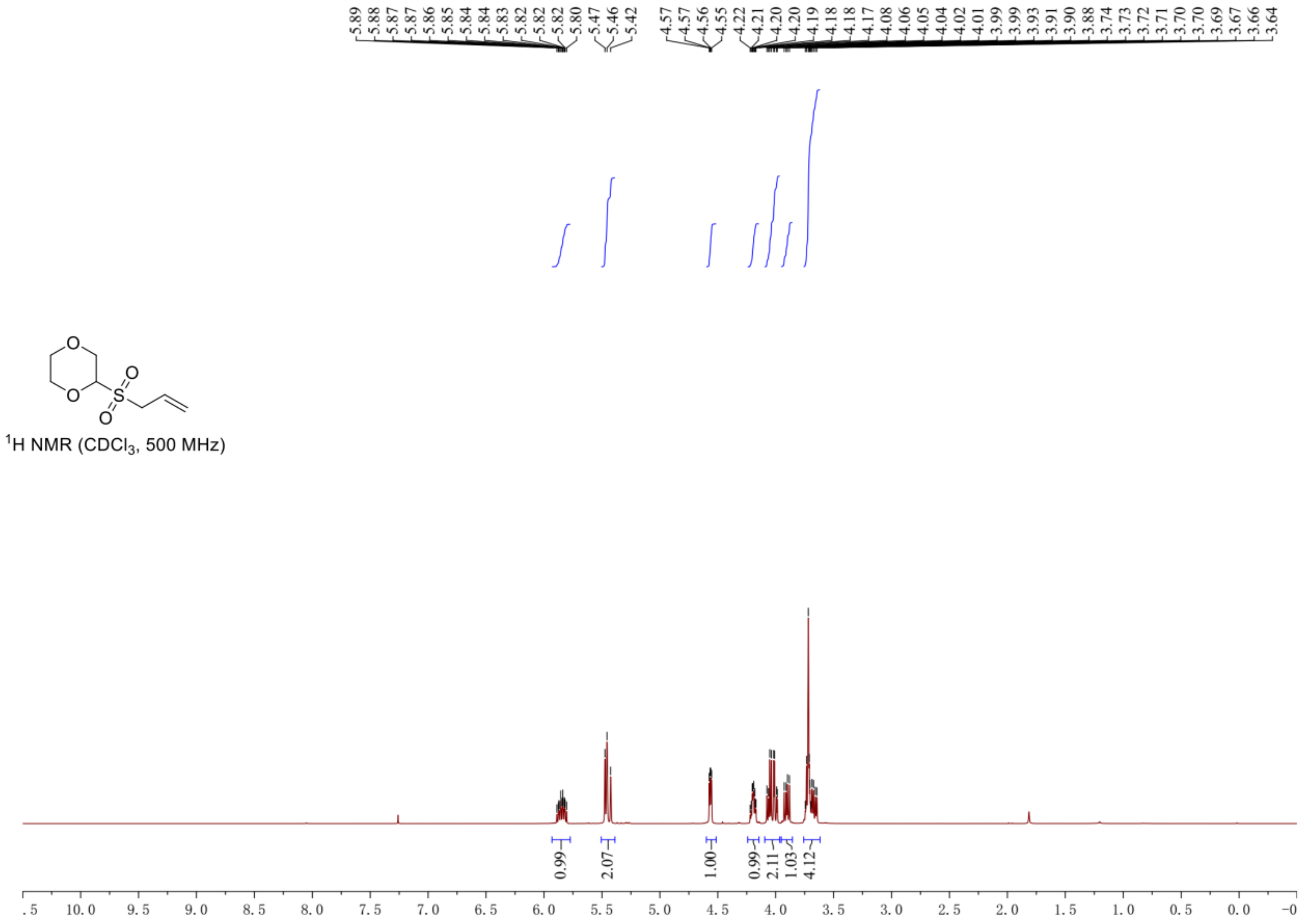

Go back to table of contents

2-(Allylsulfonyl)-1,4-dioxane (25)

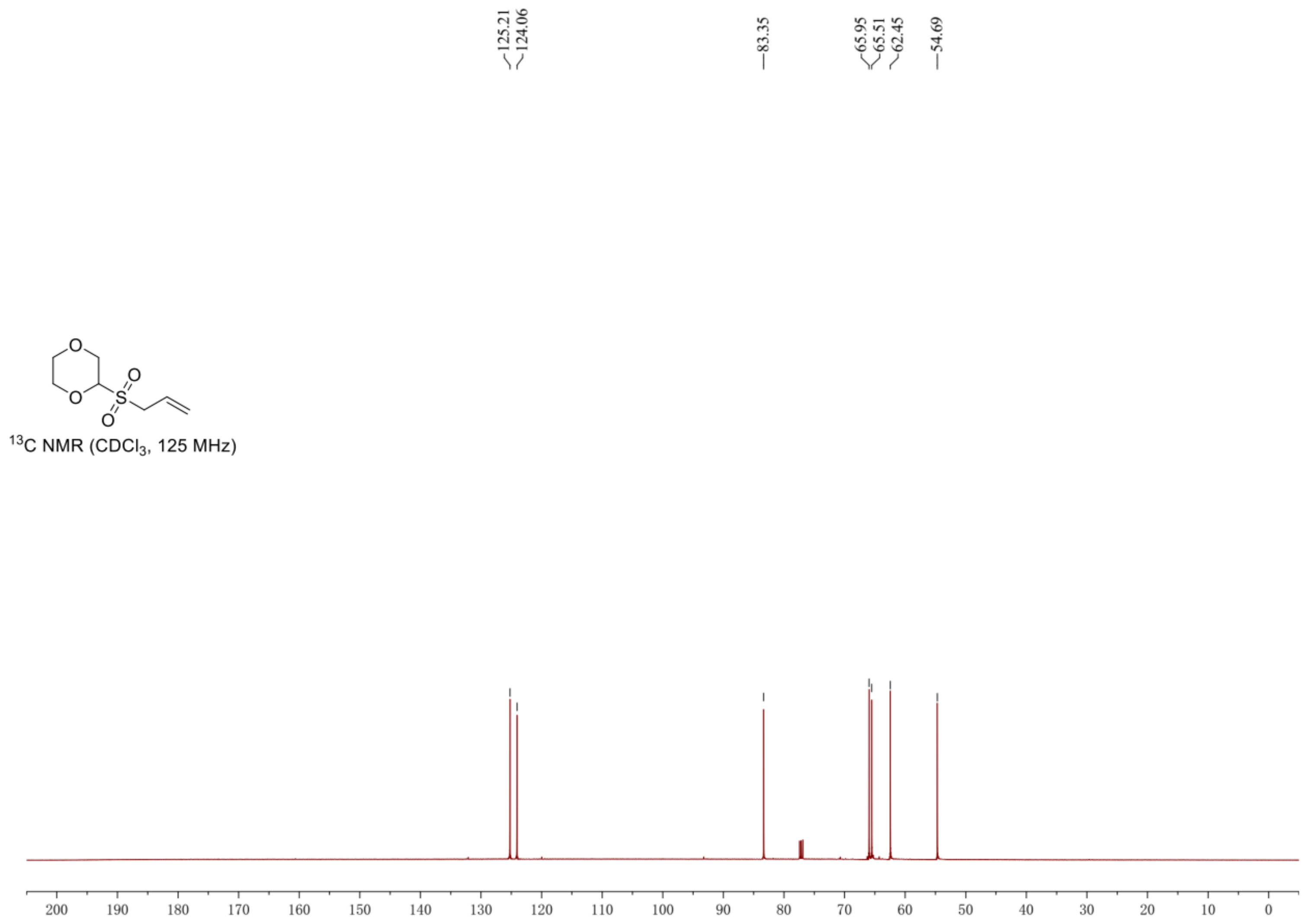

Go back to table of contents

2-(Allylsulfonyl)tetrahydrofuran (26)

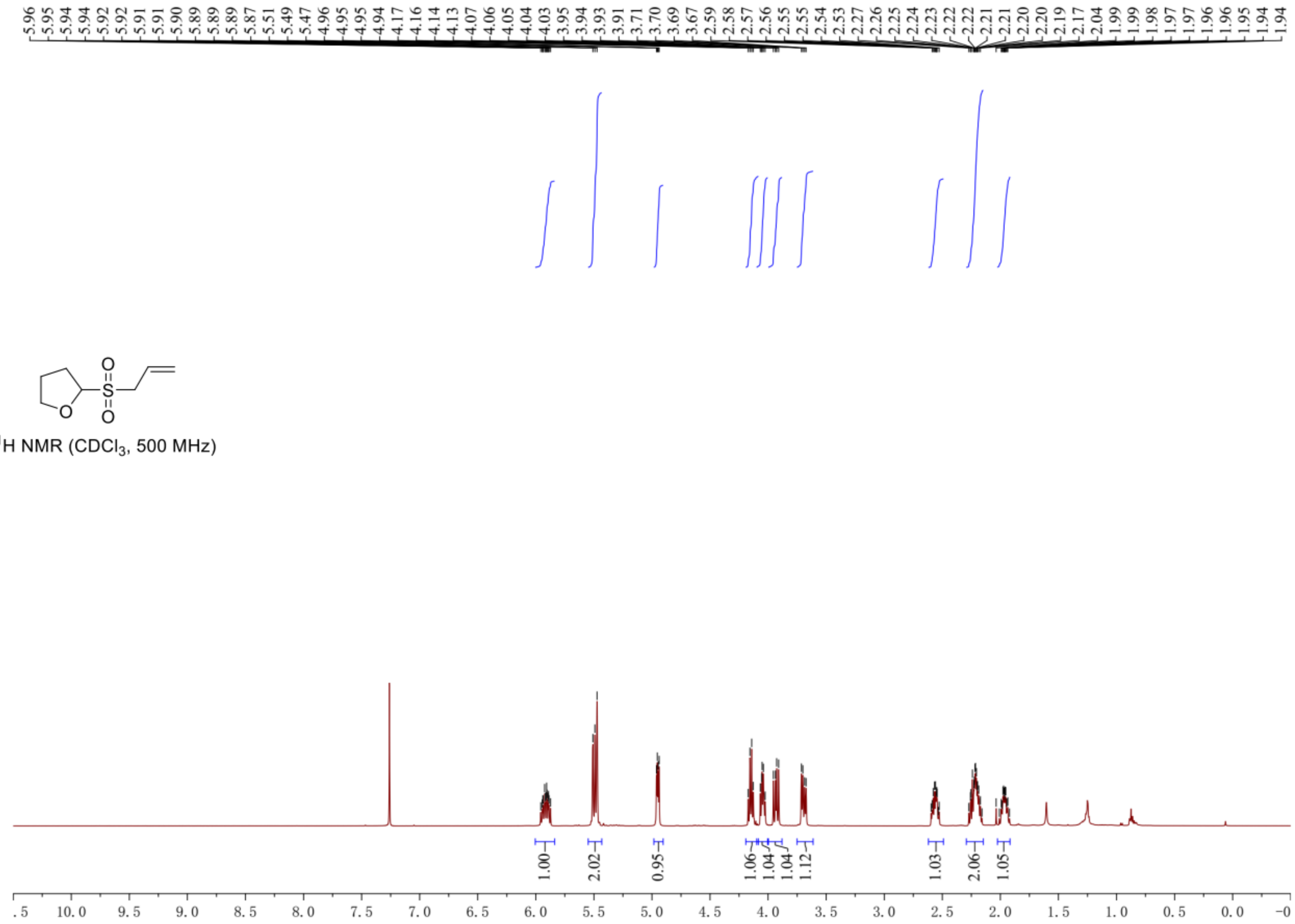

^{1}H NMR ($CDCl_3$, 500 MHz)

Go back to table of contents

2-(Allylsulfonyl)tetrahydrofuran (26)

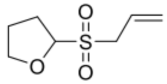

^{13}C NMR ($CDCl_3$, 125 MHz)

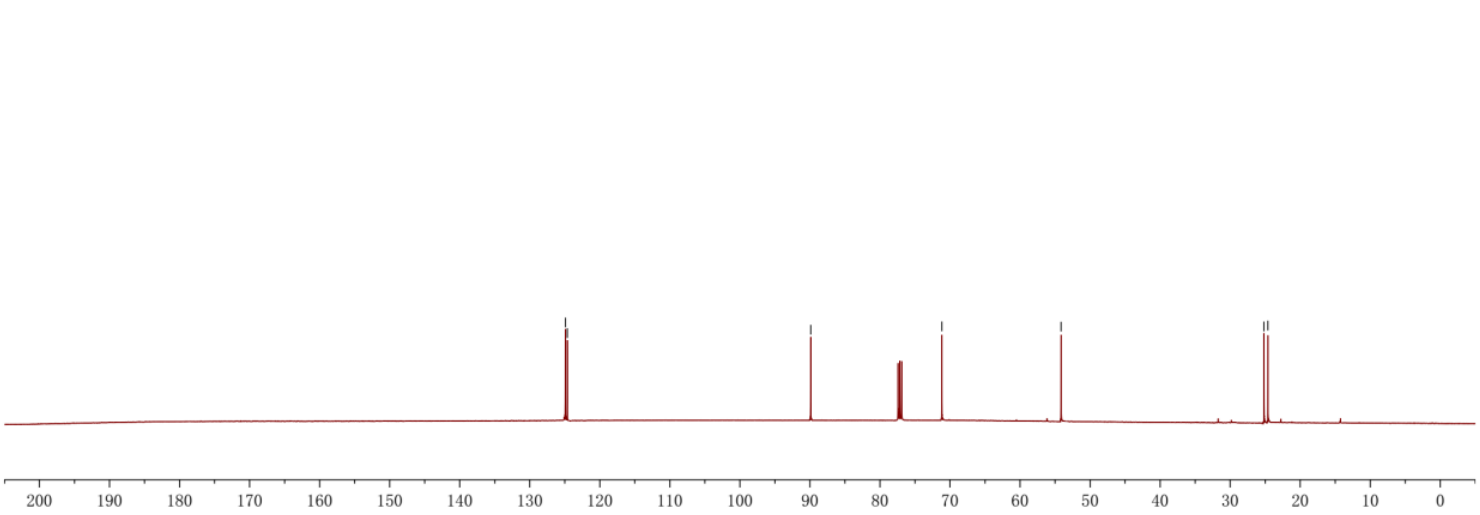

Go back to table of contents

3-(((Methoxymethoxy)methyl)sulfonyl)prop-1-ene (27)

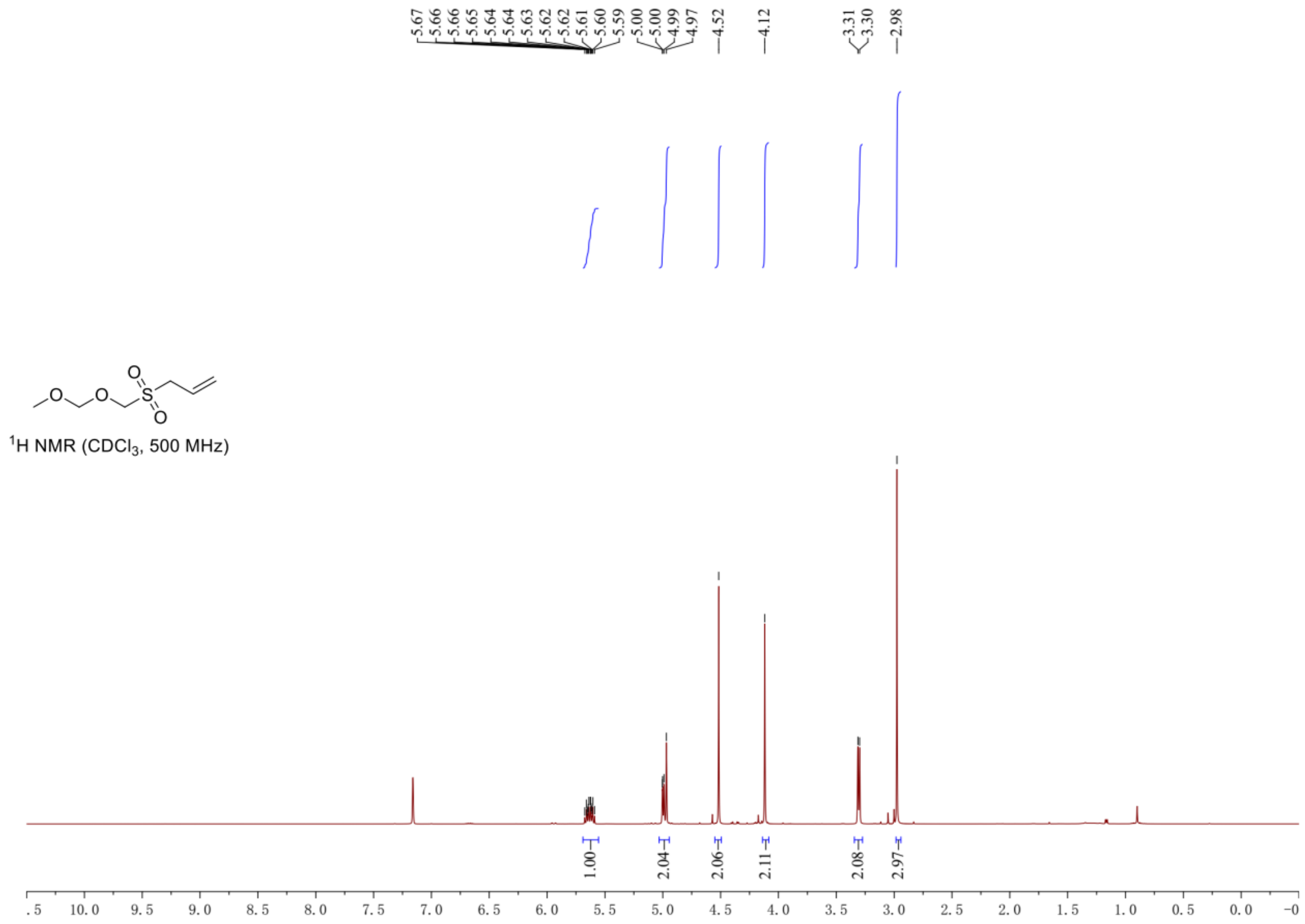

Go back to table of contents

3-(((Methoxymethoxy)methyl)sulfonyl)prop-1-ene (27)

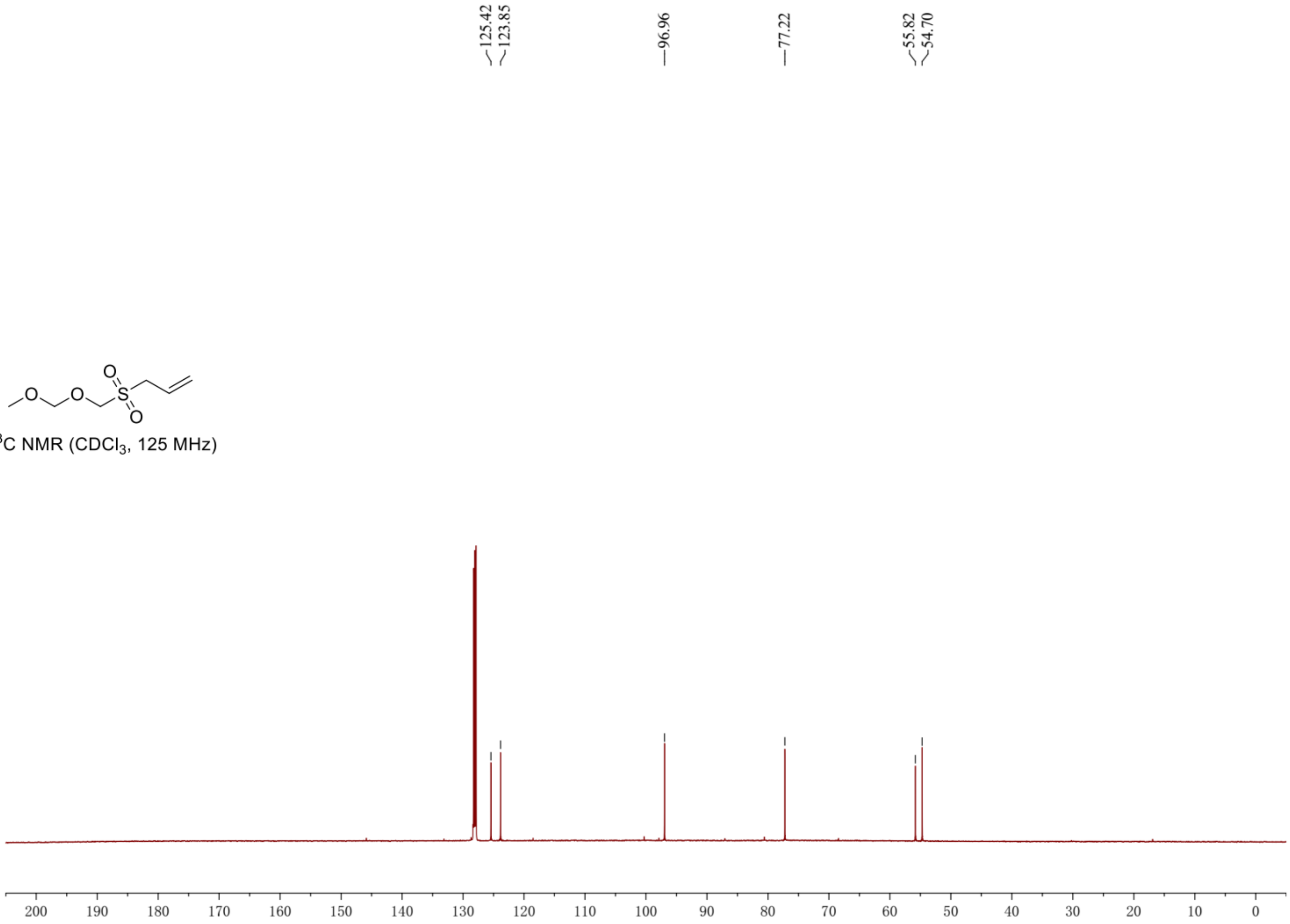

Go back to table of contents

3-((*tert*-Butoxymethyl)sulfonyl)prop-1-ene (28)

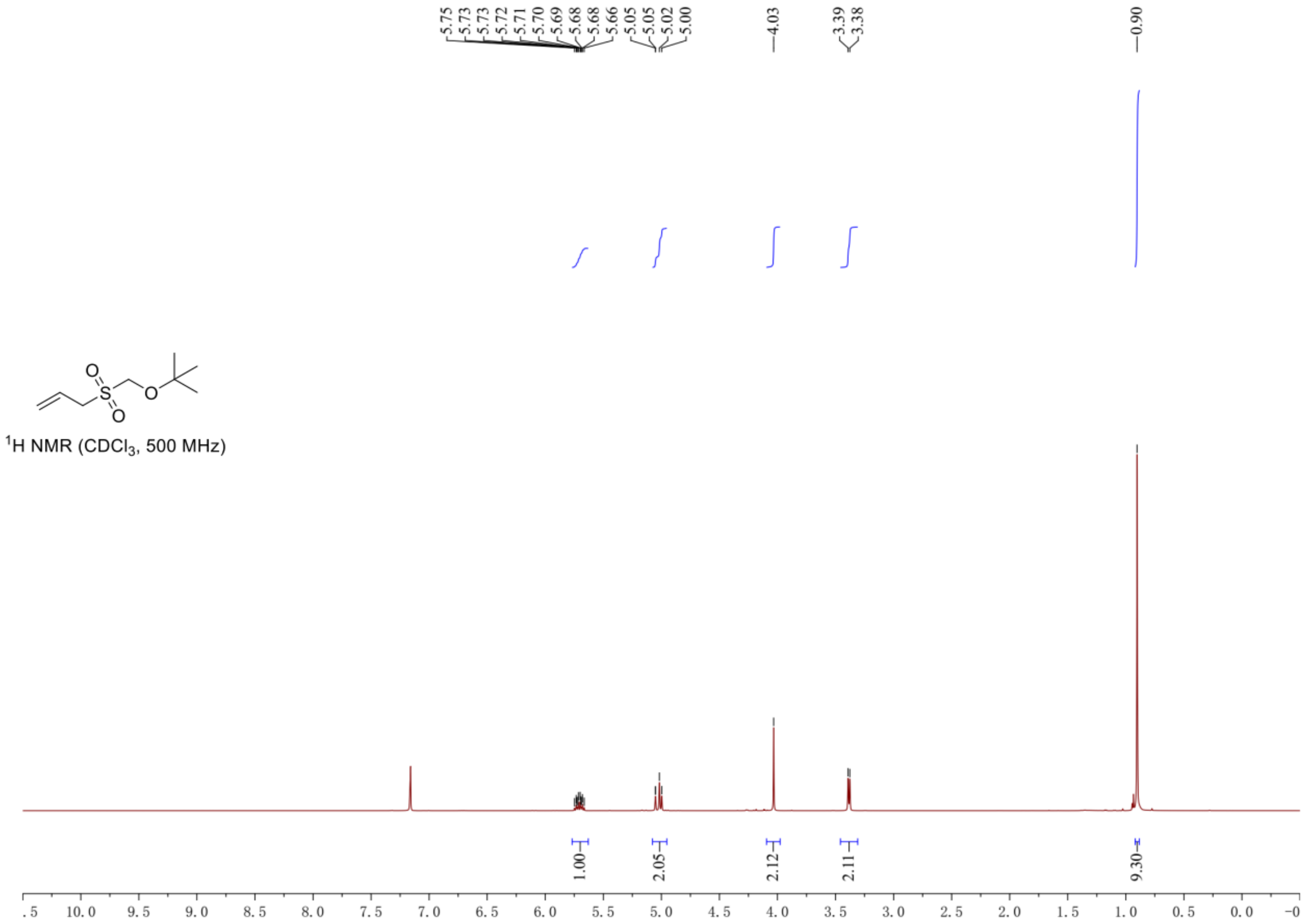

Go back to table of contents

3-((*tert*-Butoxymethyl)sulfonyl)prop-1-ene (28)

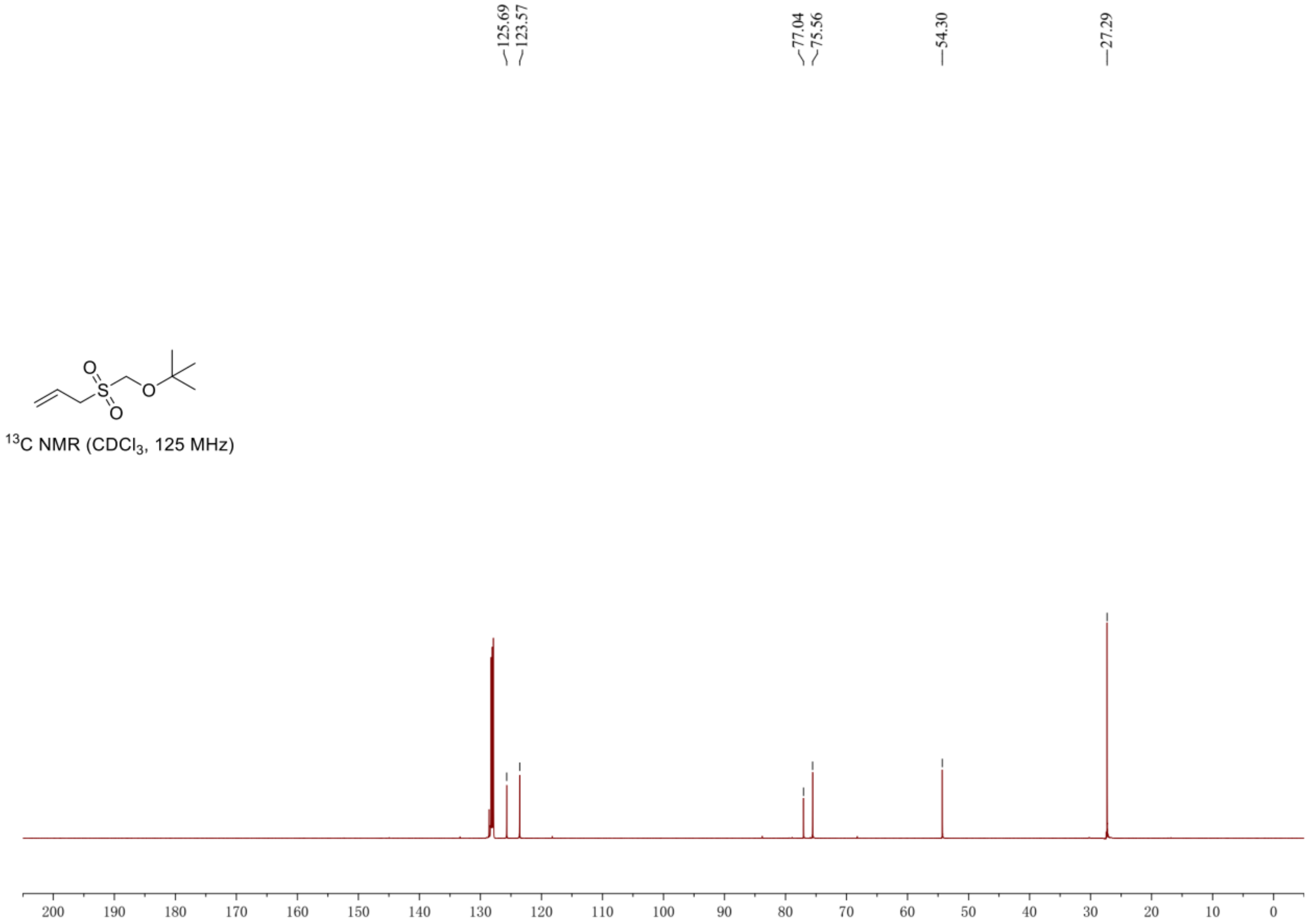

Go back to table of contents

2-(Allylsulfonyl)-1,4,7,10,13-pentaoxacyclopentadecane (29)

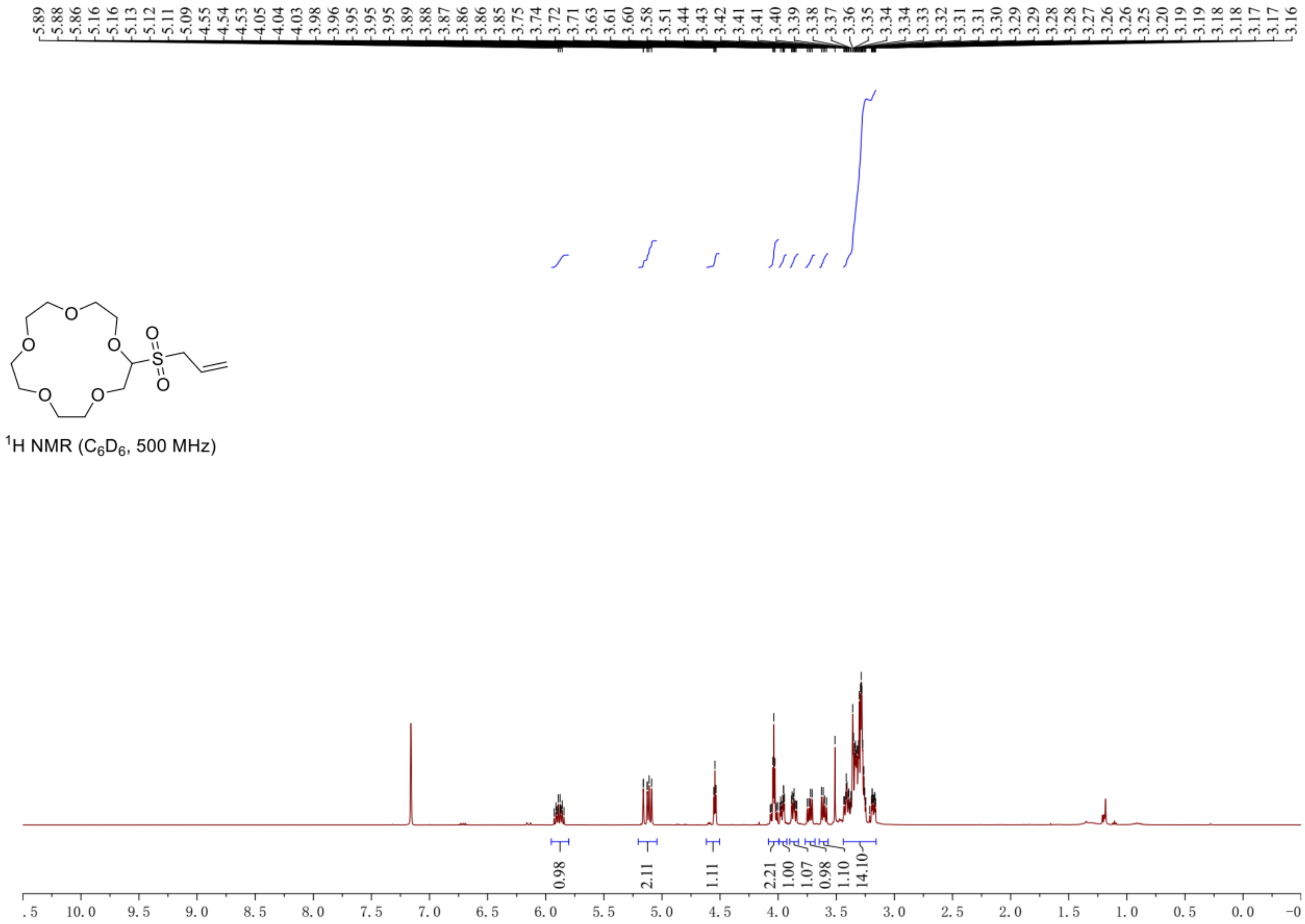

^{1}H NMR (C_6D_6, 500 MHz)

Go back to table of contents

2-(Allylsulfonyl)-1,4,7,10,13-pentaoxacyclopentadecane (29)

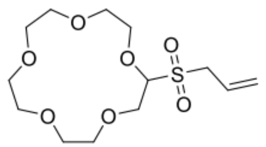

^{13}C NMR (C_6D_6, 125 MHz)

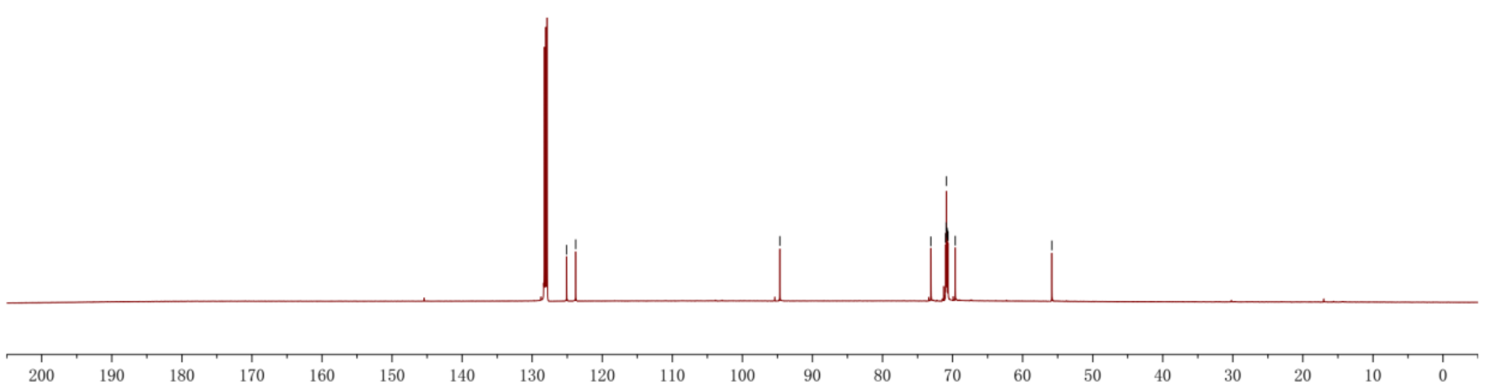

Go back to table of contents

2-(Allylsulfonyl)-1,4,7,10,13,16-hexaoxacyclooctadecane (30)

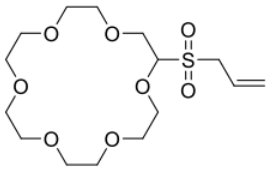

^{1}H NMR (C_6D_6, 500 MHz)

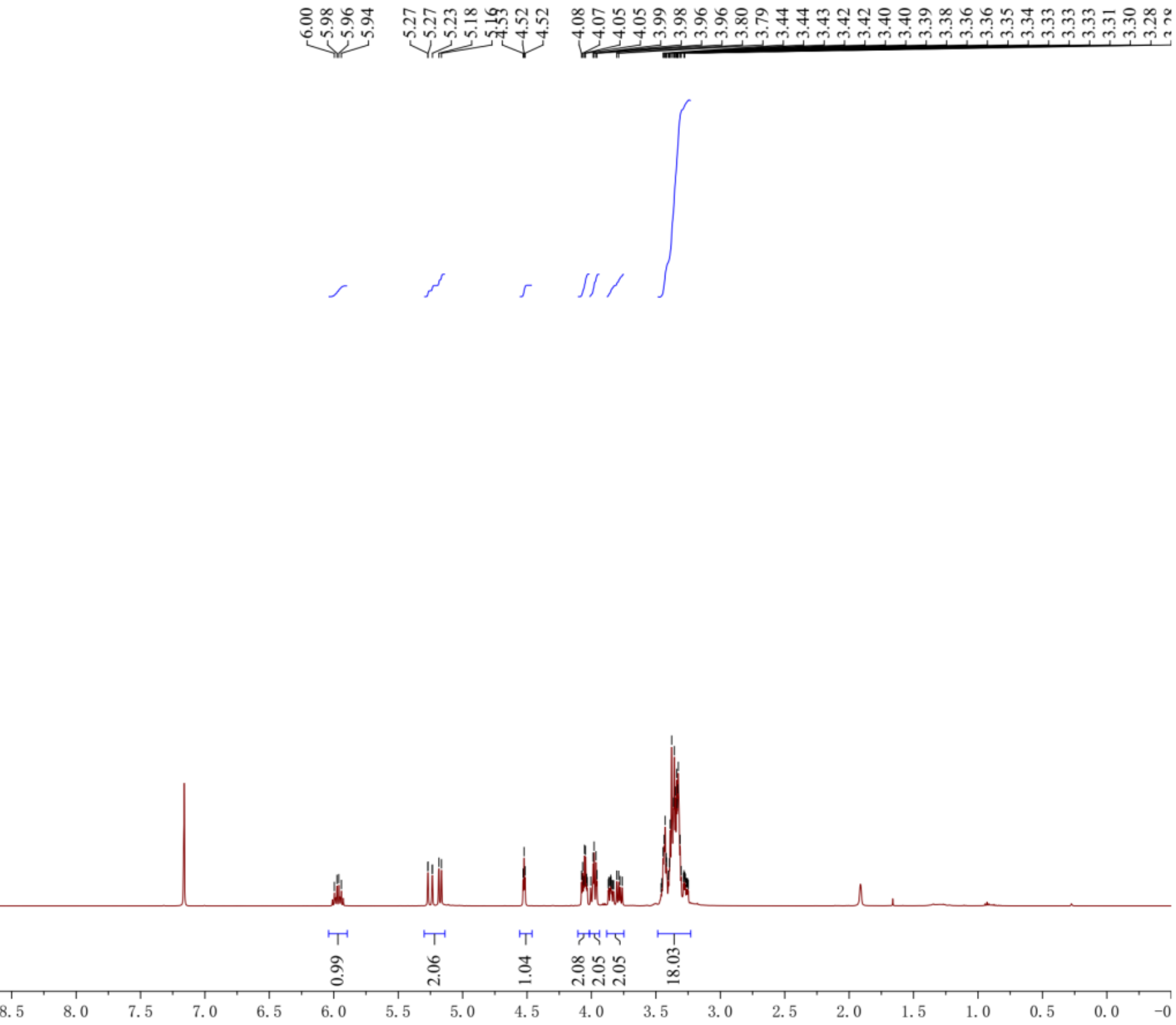

Go back to table of contents

2-(Allylsulfonyl)-1,4,7,10,13,16-hexaoxacyclooctadecane (30)

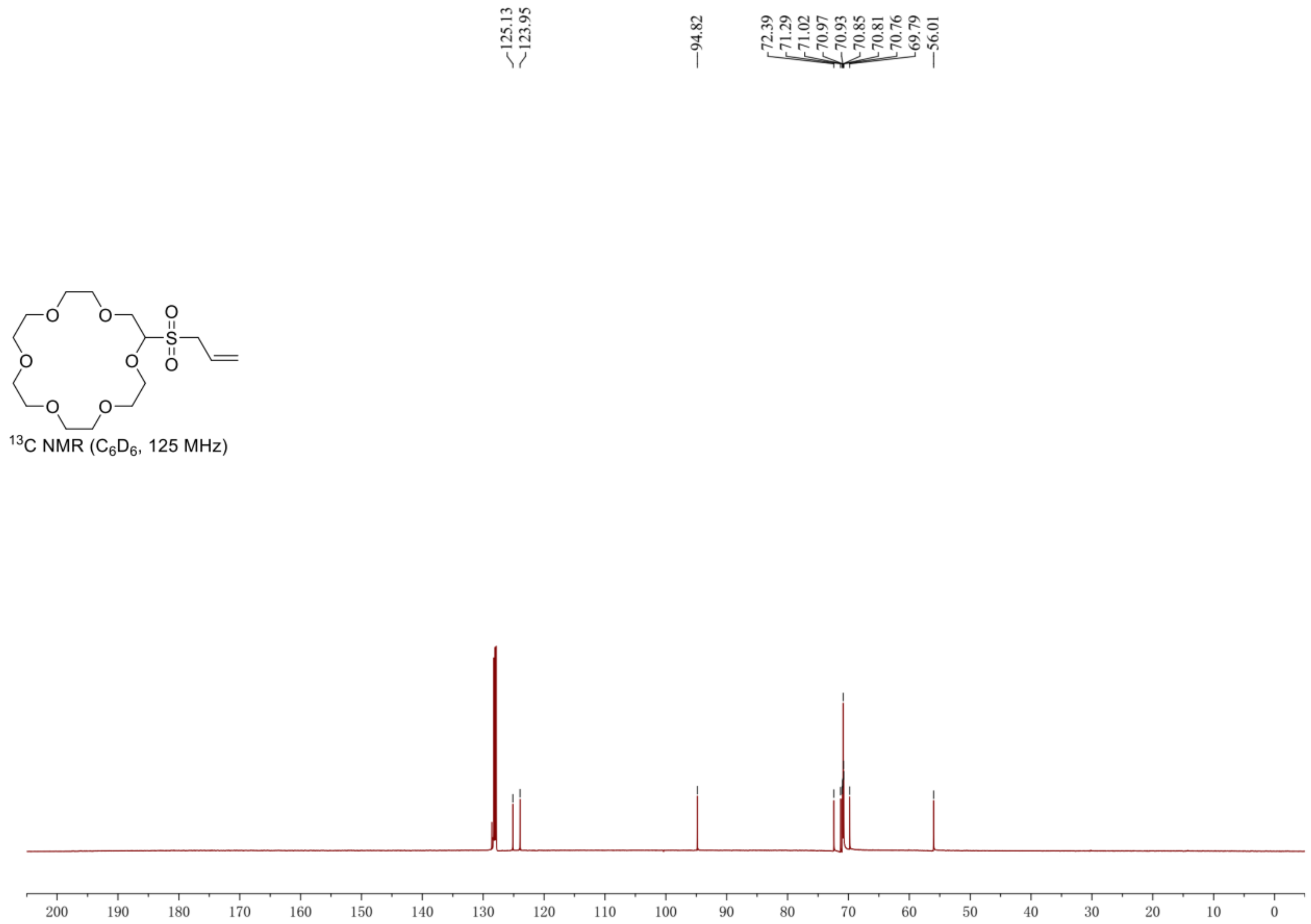

Go back to table of contents

(3*R*,3a*R*,6*R*,6a*R*)-3-((Allylsulfonyl)methoxy)-6-methoxyhexahydrofuro[3,2-*b*]furan (31a)

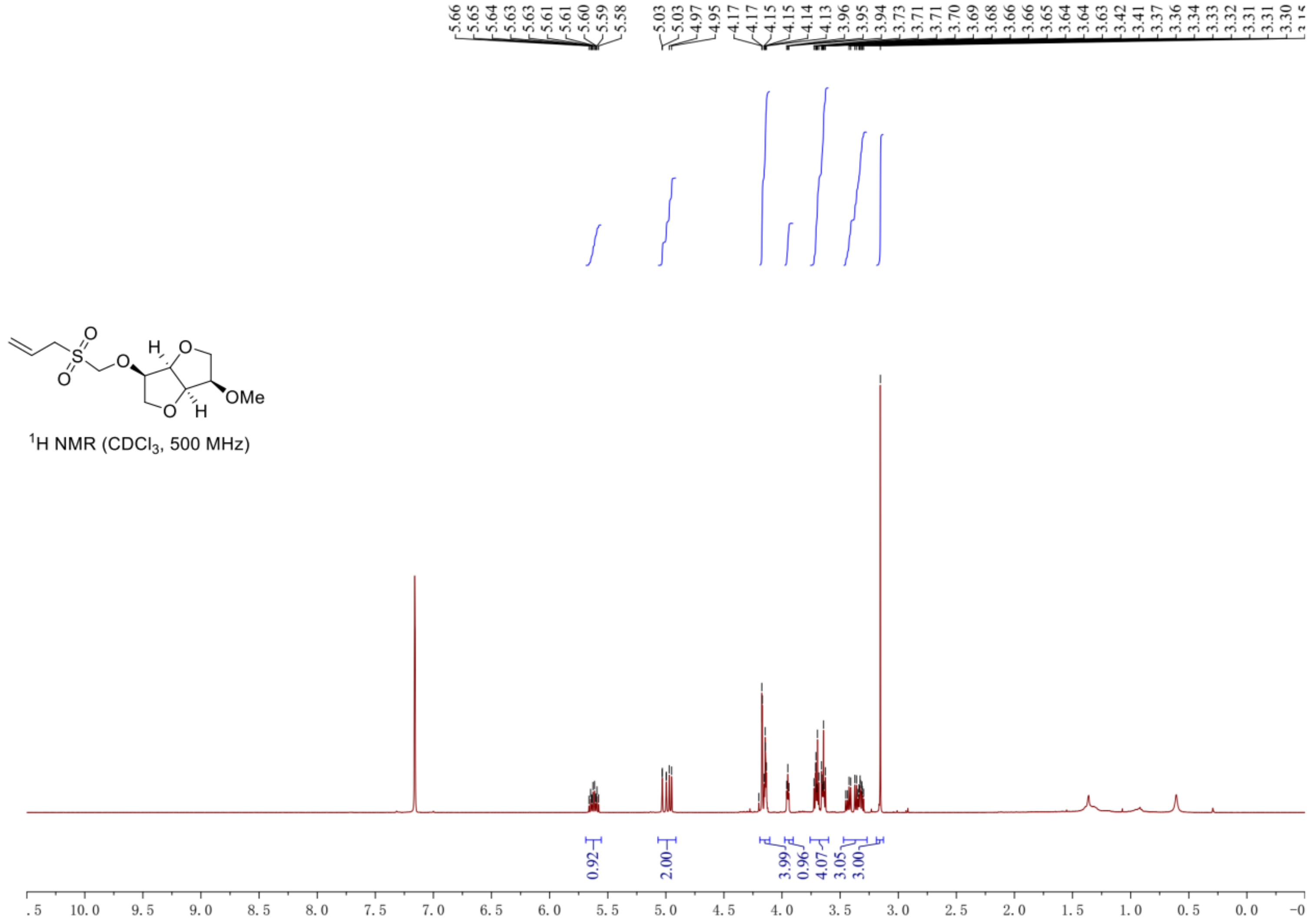

Go back to table of contents

(3*R*,3a*R*,6*R*,6a*R*)-3-((Allylsulfonyl)methoxy)-6-methoxyhexahydrofuro[3,2-*b*]furan (31a)

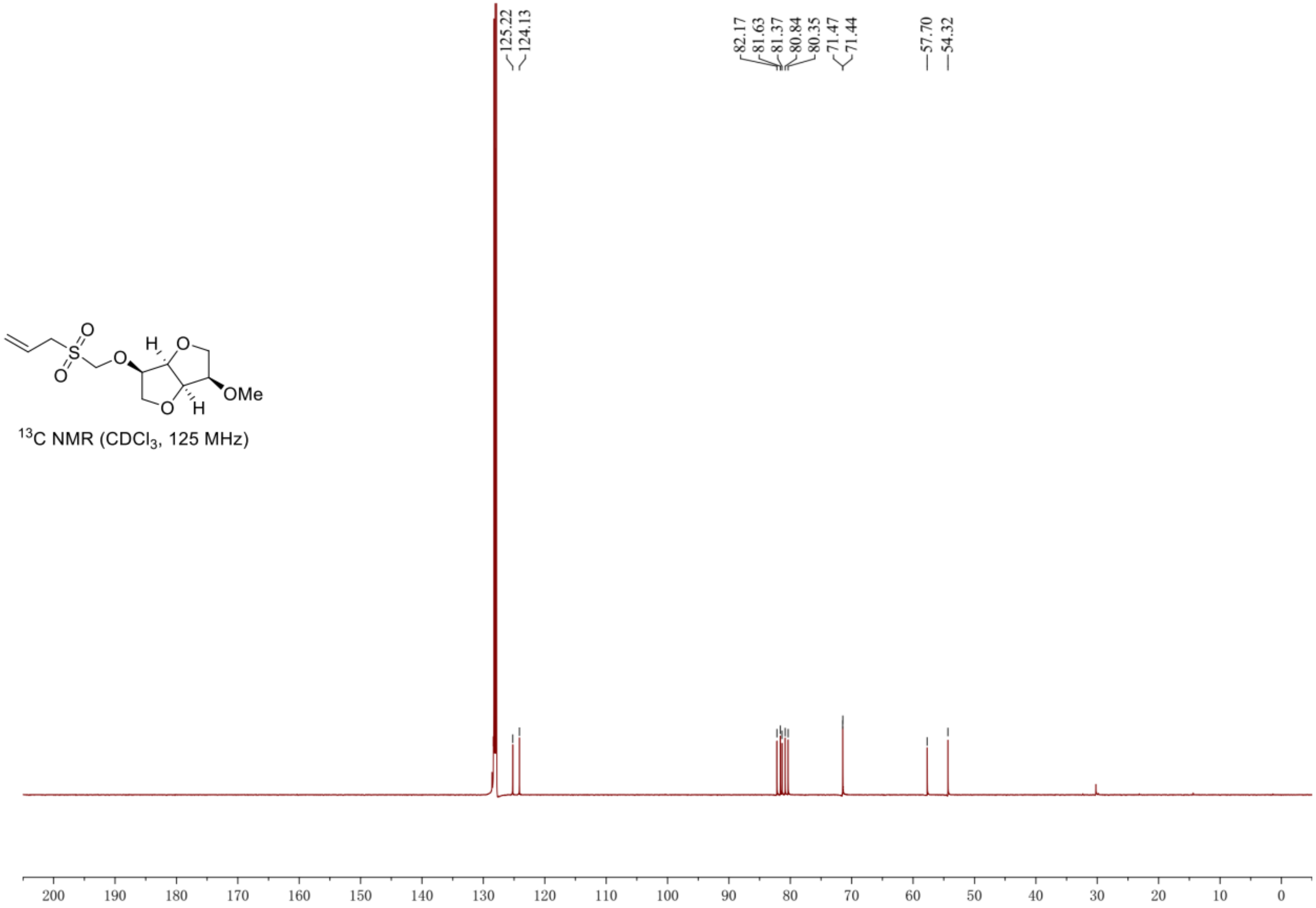

^{13}C NMR ($CDCl_3$, 125 MHz)

Go back to table of contents

(3*R*,3a*S*,6*R*,6a*R*)-3a-(Allylsulfonyl)-3,6-dimethoxyhexahydrofuro[3,2-*b*]furan (31b)

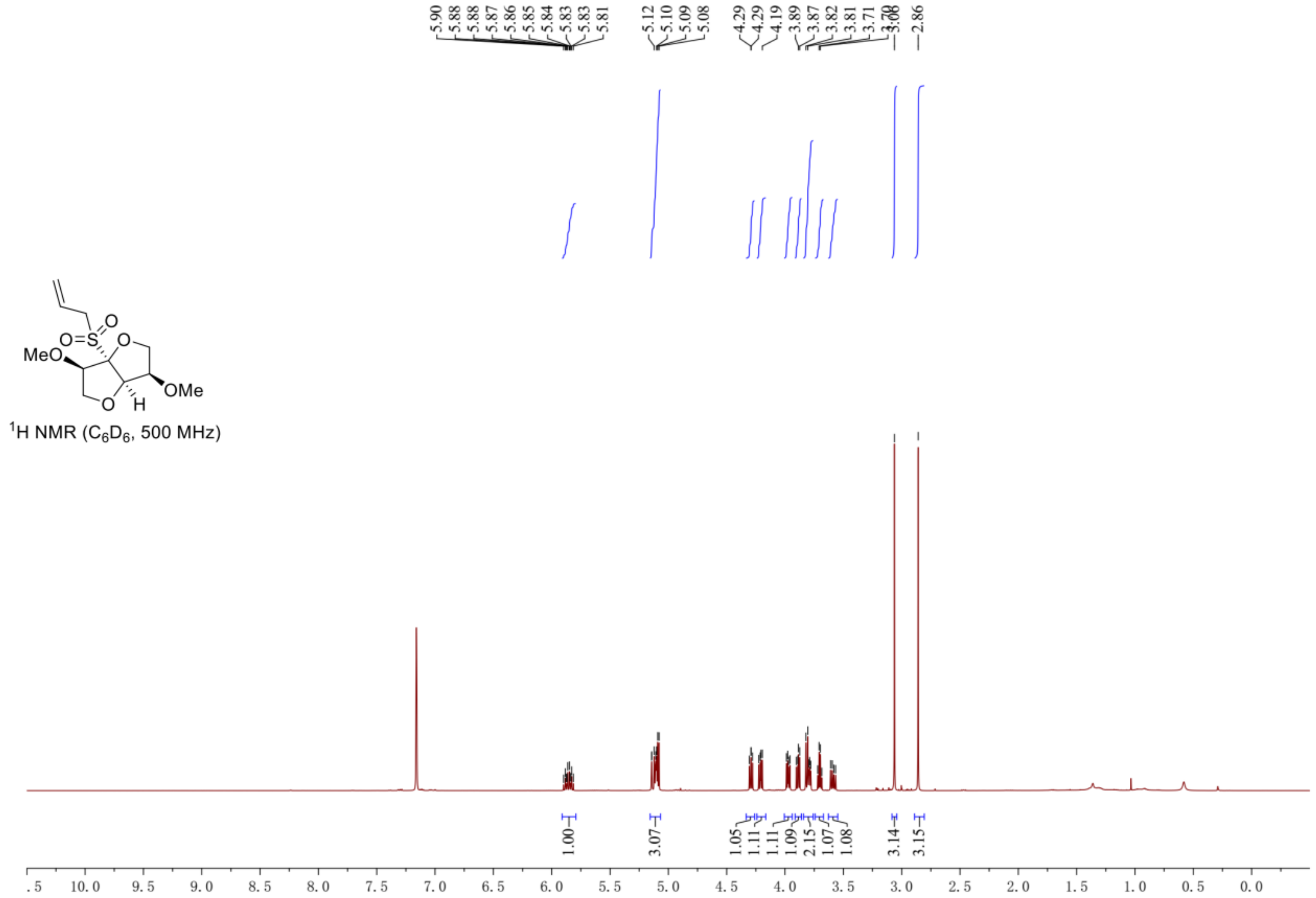

Go back to table of contents

(3*R*,3a*S*,6*R*,6a*R*)-3a-(Allylsulfonyl)-3,6-dimethoxyhexahydrofuro[3,2-*b*]furan (31b)

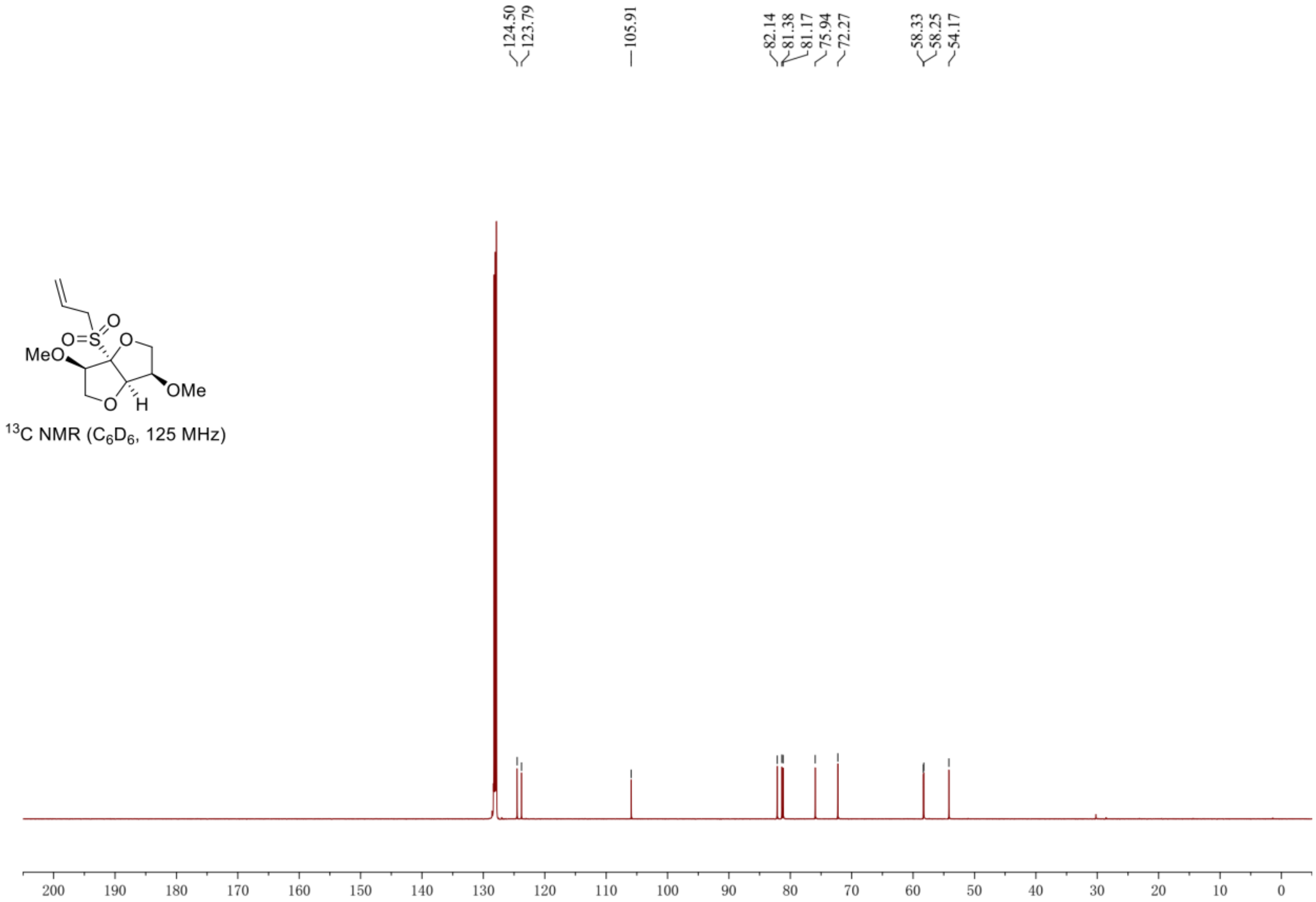

Go back to table of contents

(2*R*,3*S*,3a*S*,6*R*,6a*R*)-2-(Allylsulfonyl)-3,6-dimethoxyhexahydrofuro[3,2-*b*]furan (31c)

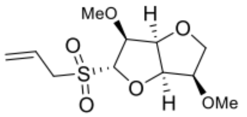

^1H NMR (C_6D_6, 500 MHz)

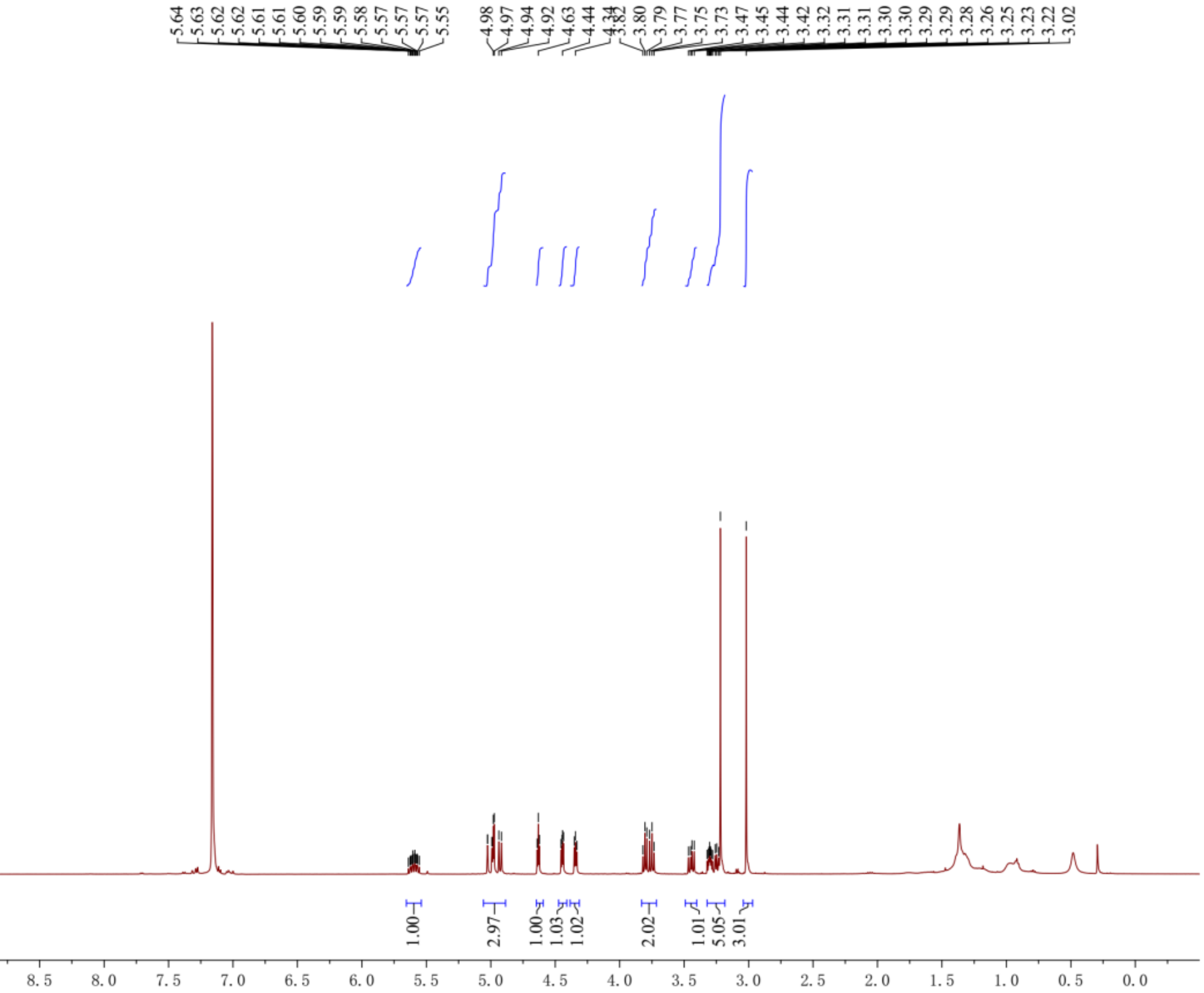

Go back to table of contents

(2*R*,3*S*,3a*S*,6*R*,6a*R*)-2-(Allylsulfonyl)-3,6-dimethoxyhexahydrofuro[3,2-*b*]furan (31c)

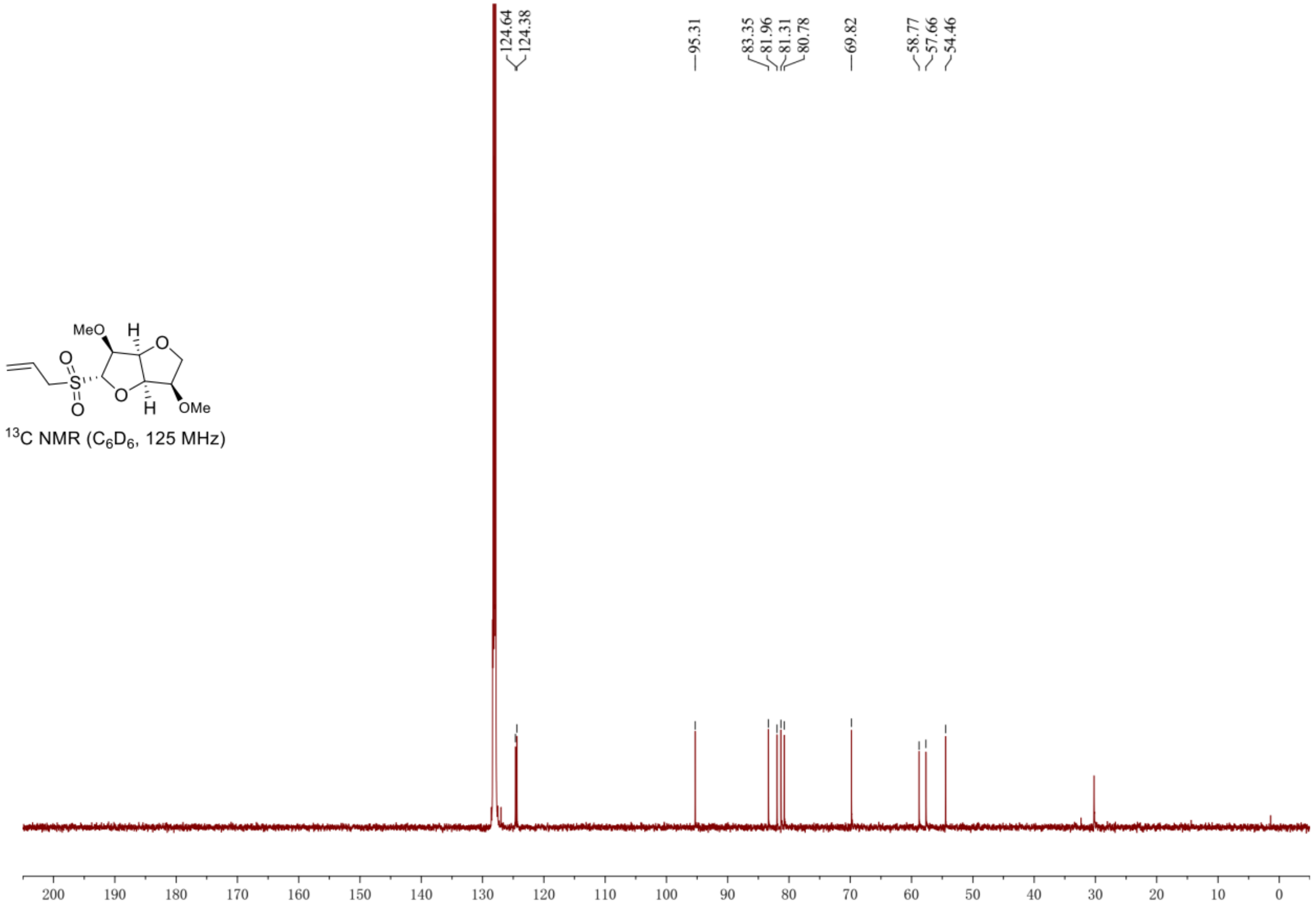

Go back to table of contents

4-Methyl-4-(methylsulfonyl)pentan-2-one (33)

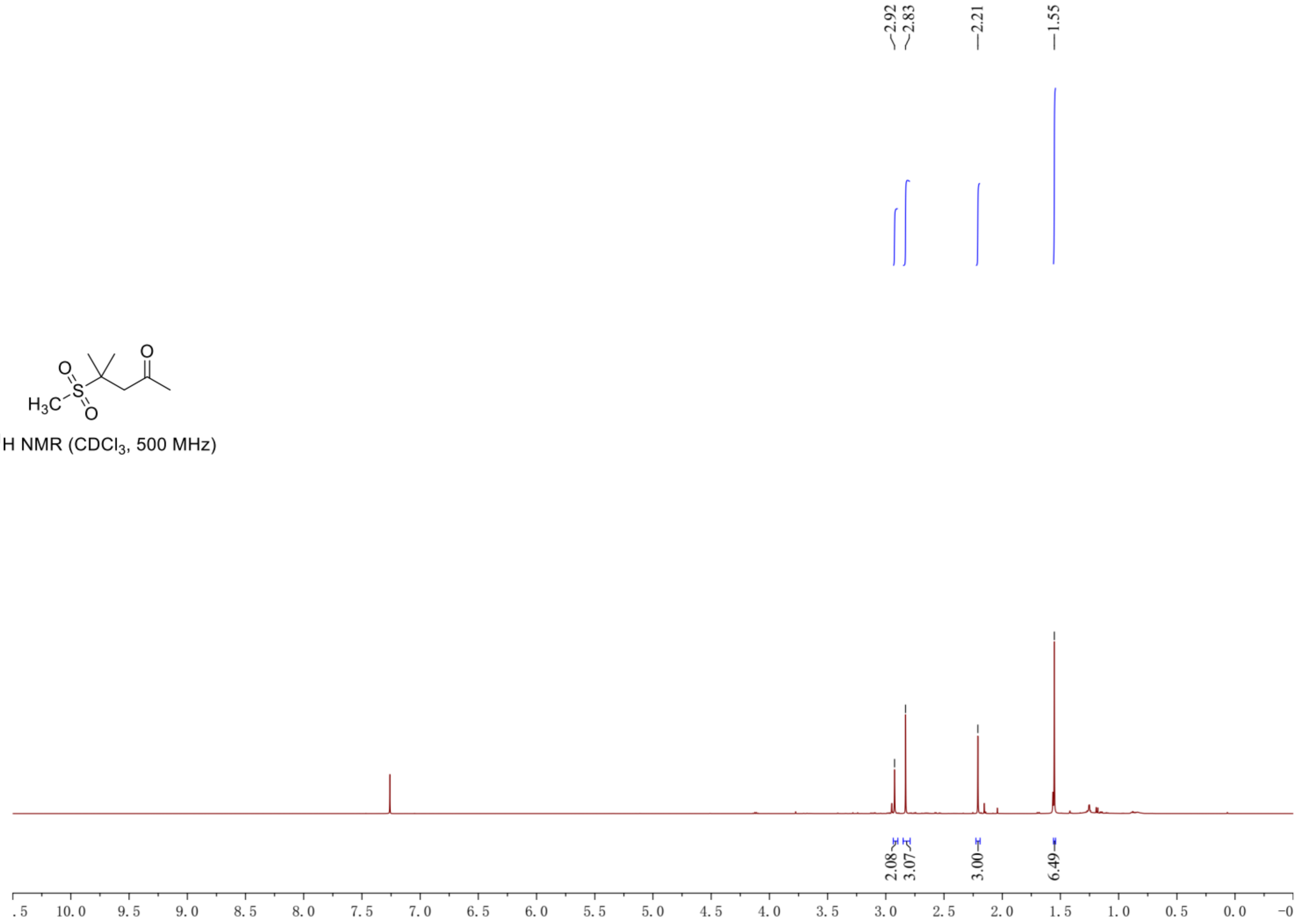

^{1}H NMR (CDCl$_3$, 500 MHz)

Go back to table of contents

4-Methyl-4-(methylsulfonyl)pentan-2-one (33)

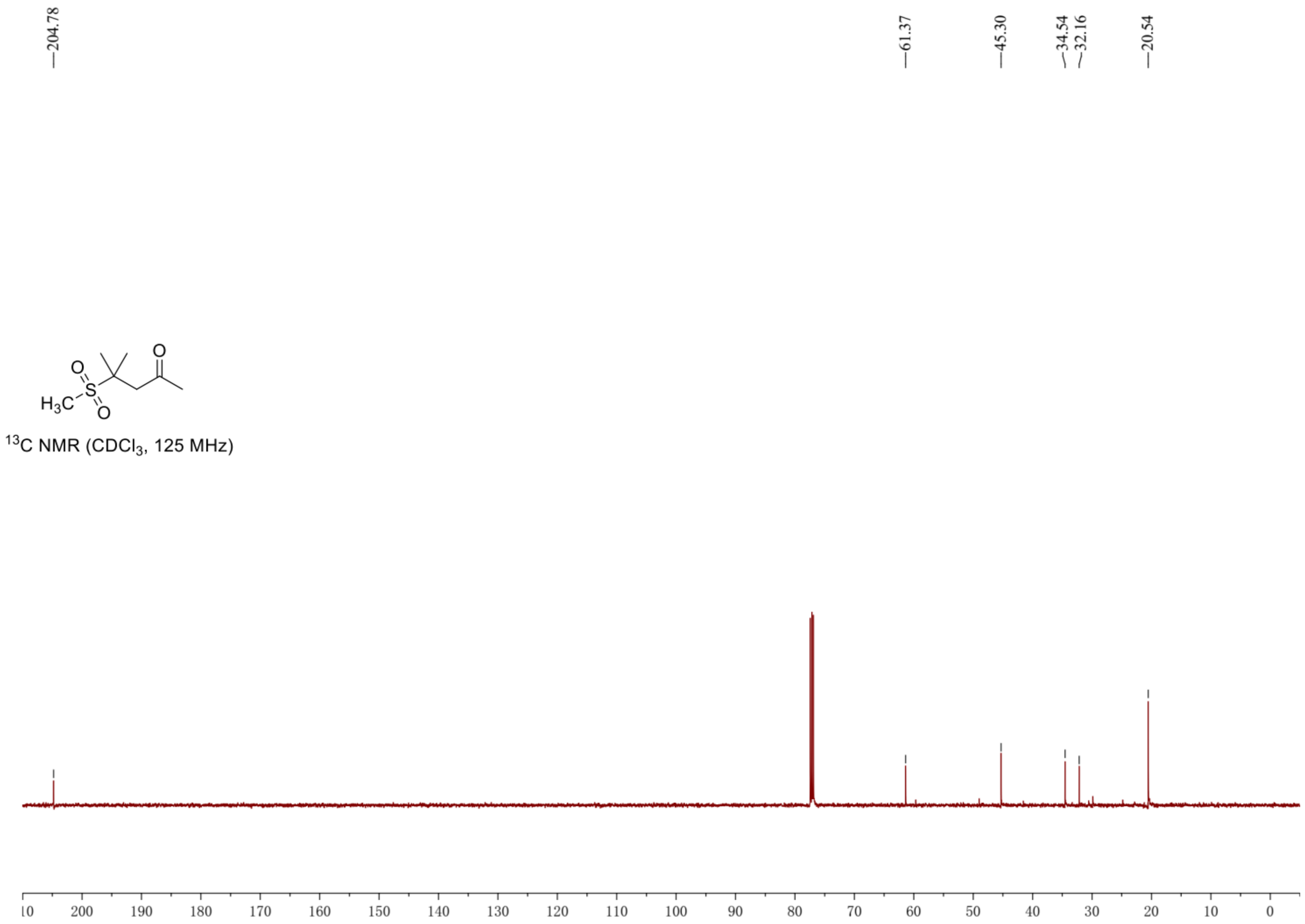

Go back to table of contents

4-Methyl-5-(methylsulfonyl)pentan-2-one (34)

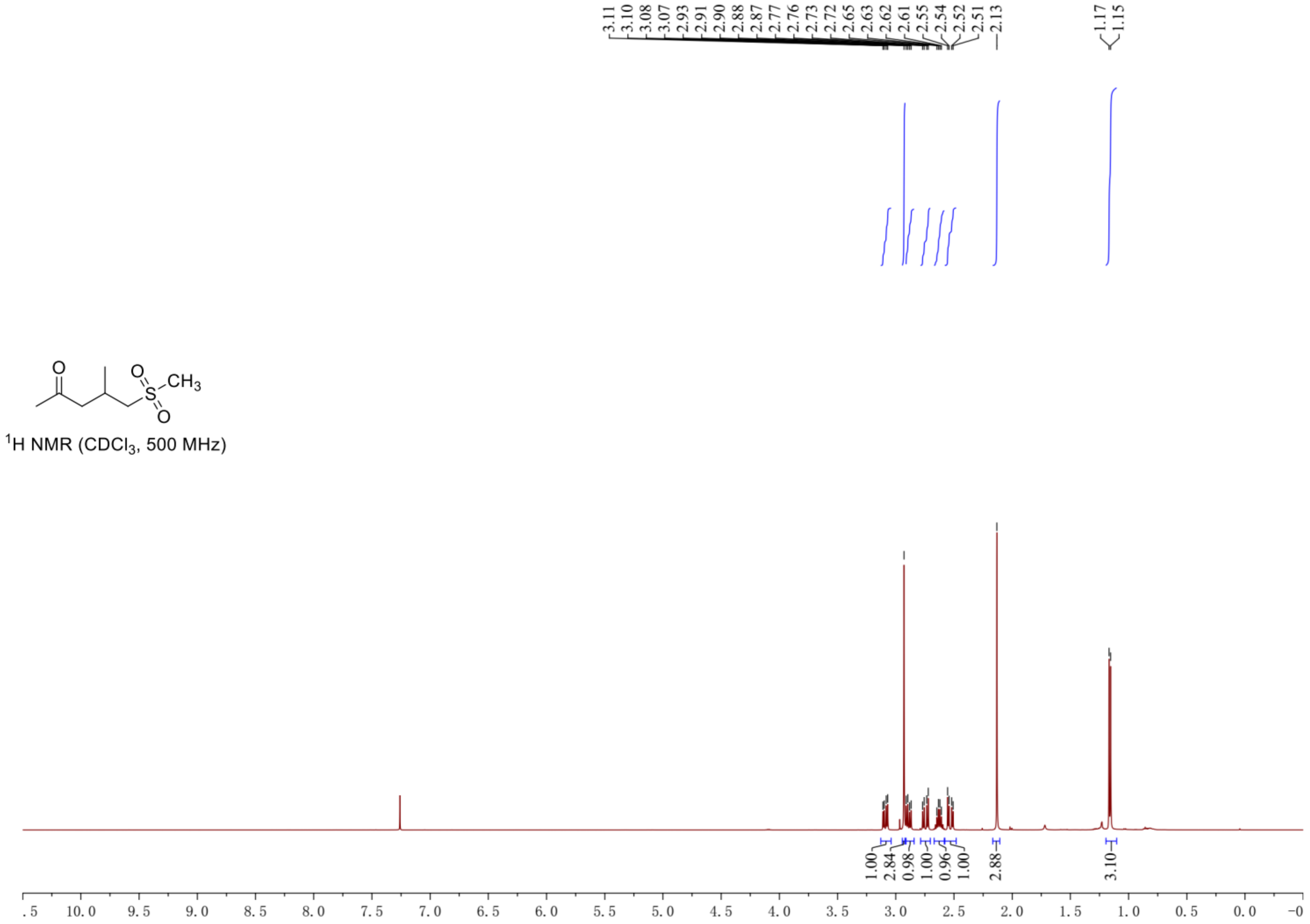

Go back to table of contents

4-Methyl-5-(methylsulfonyl)pentan-2-one (34)

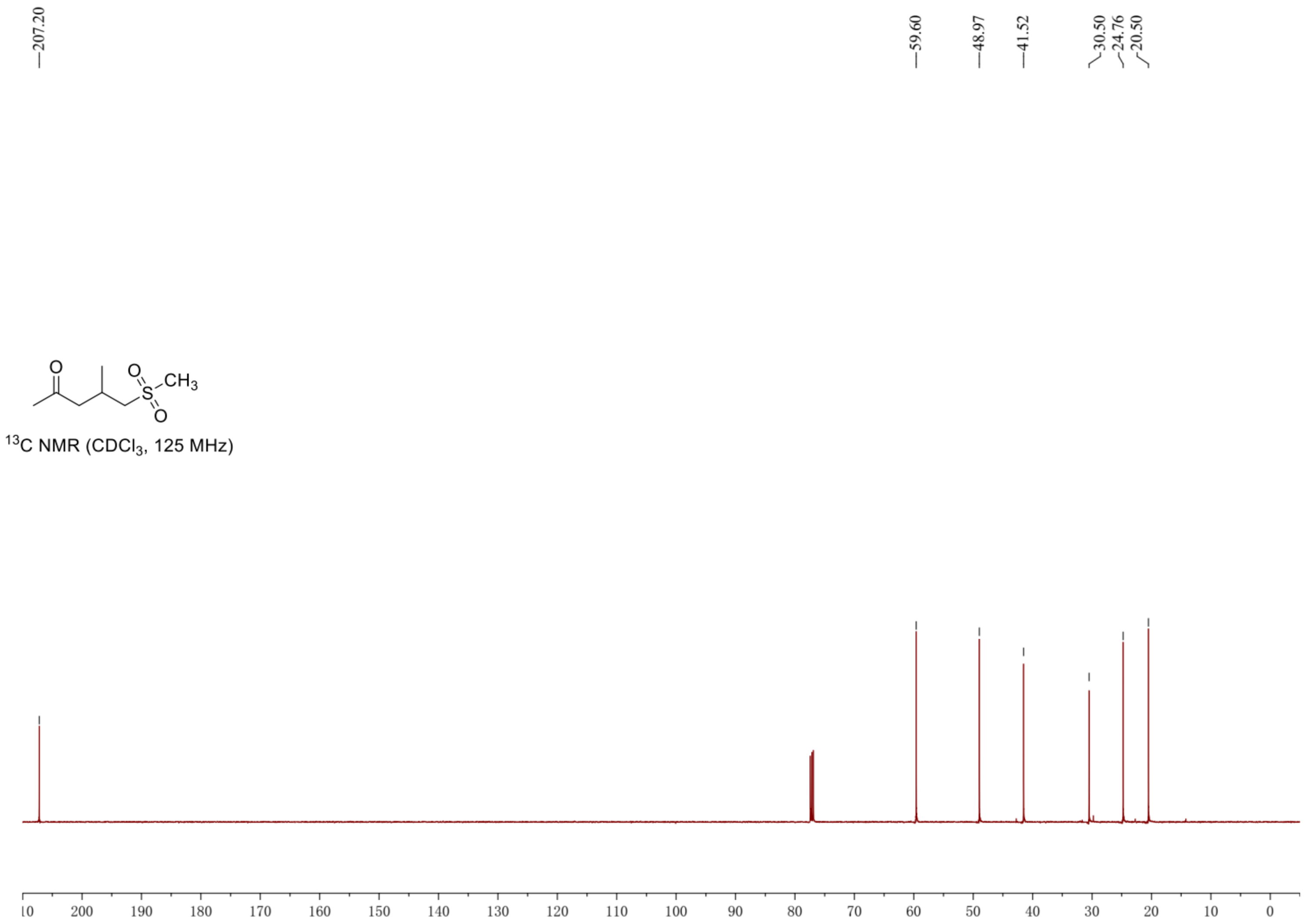

Go back to table of contents

1-(Methylsulfonyl)heptan-4-one (S1)

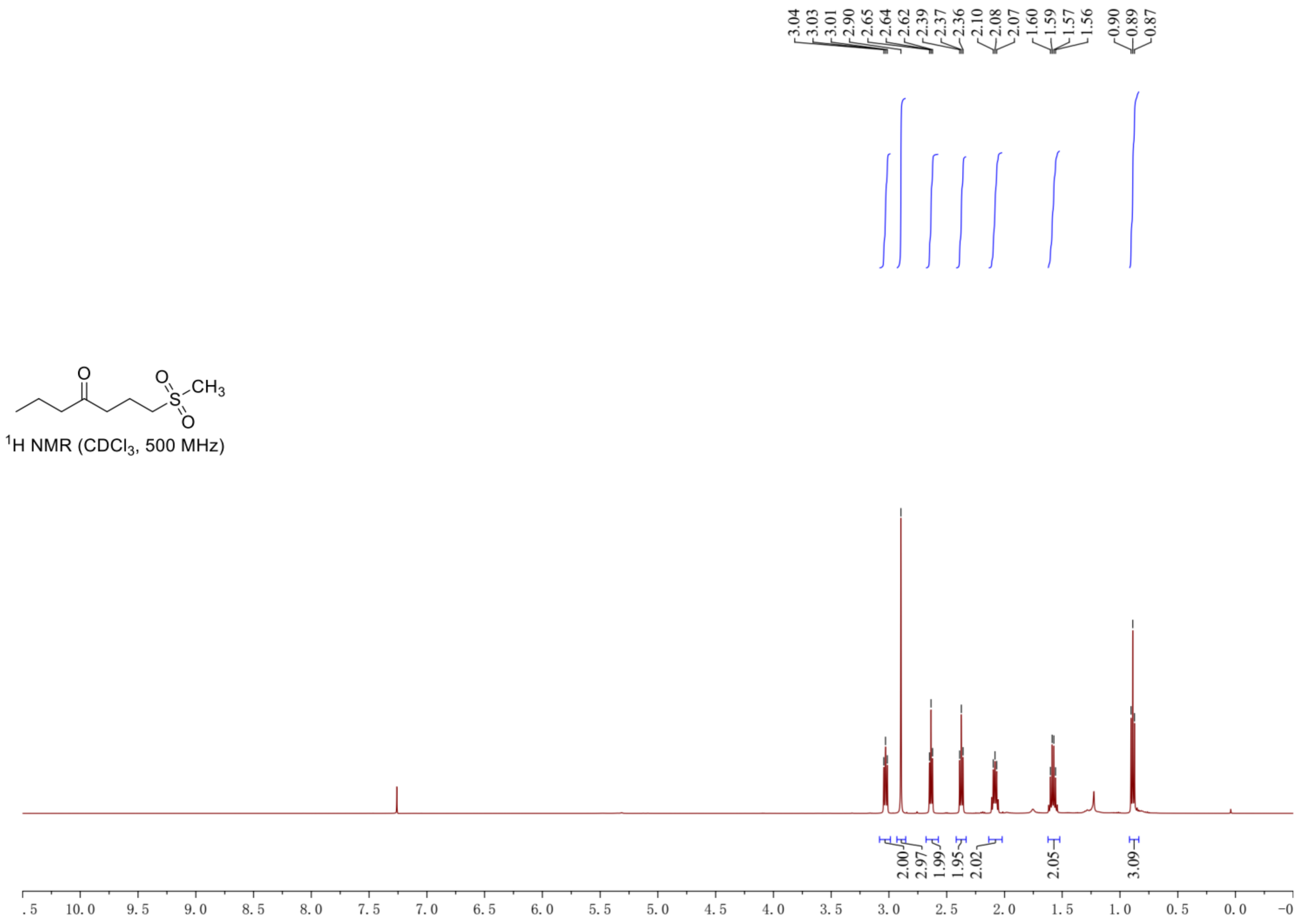

Go back to table of contents

1-(Methylsulfonyl)heptan-4-one (S1)

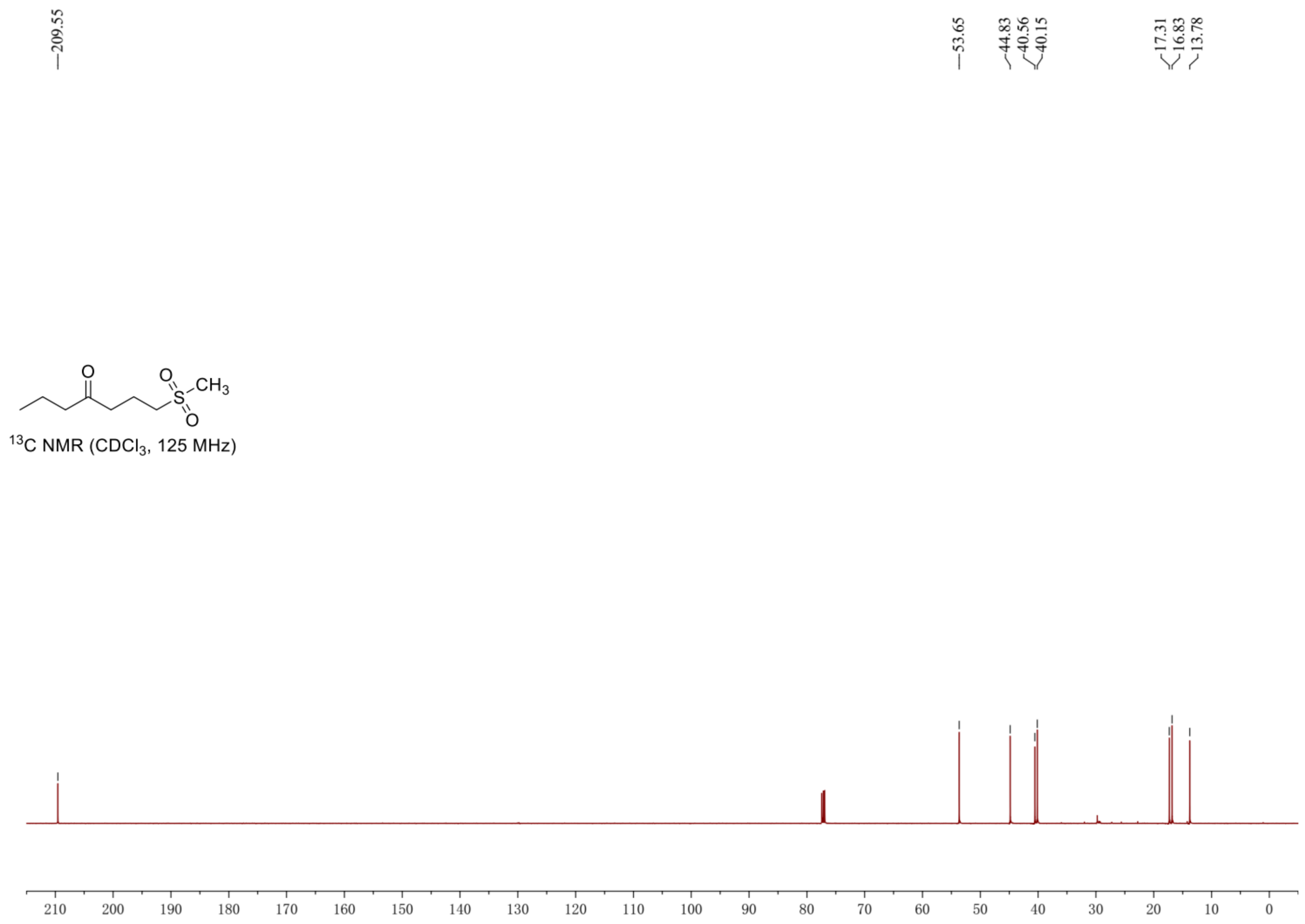

Go back to table of contents

2-(Methylsulfonyl)heptan-4-one (S2)

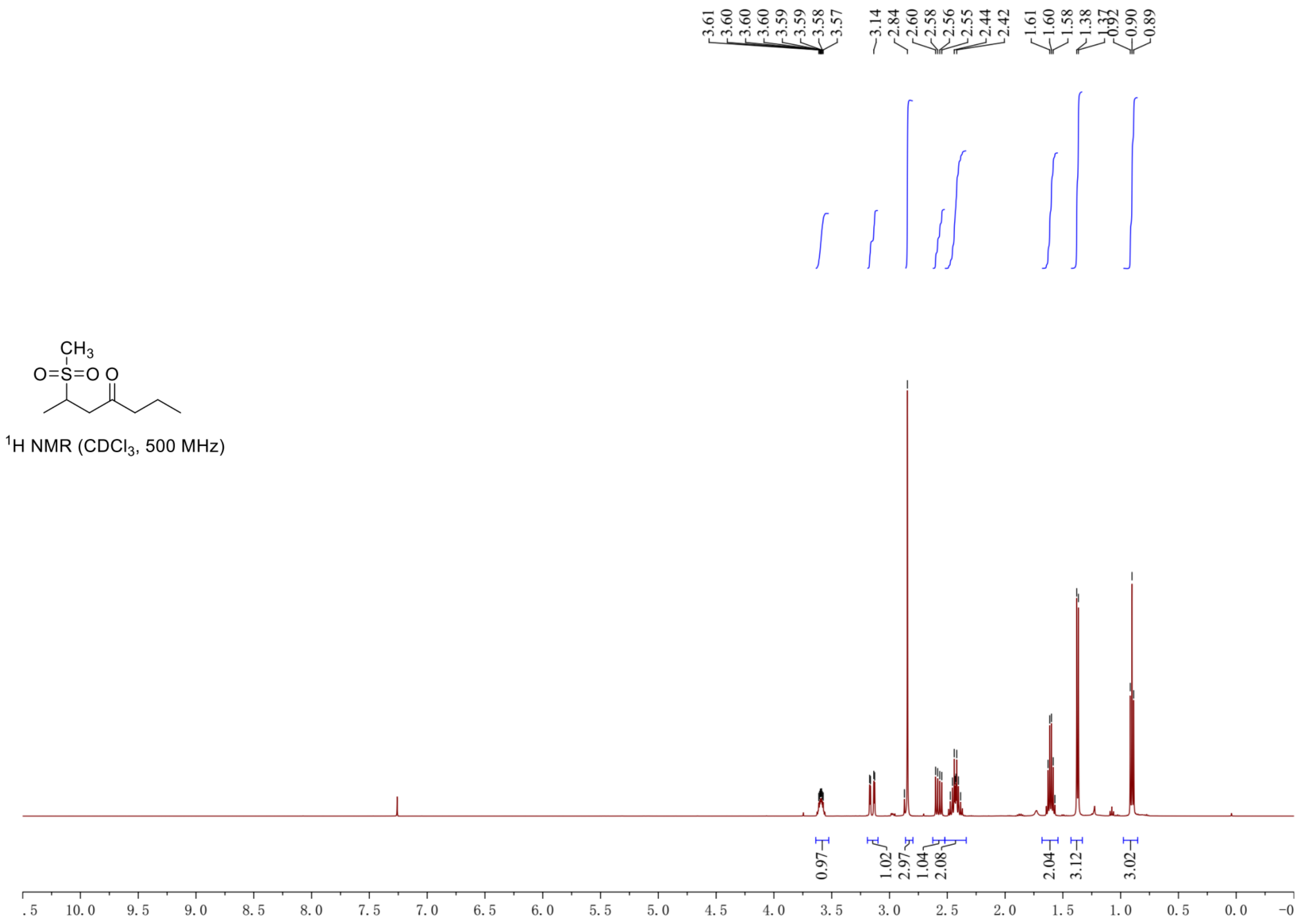

Go back to table of contents

2-(Methylsulfonyl)heptan-4-one (S2)

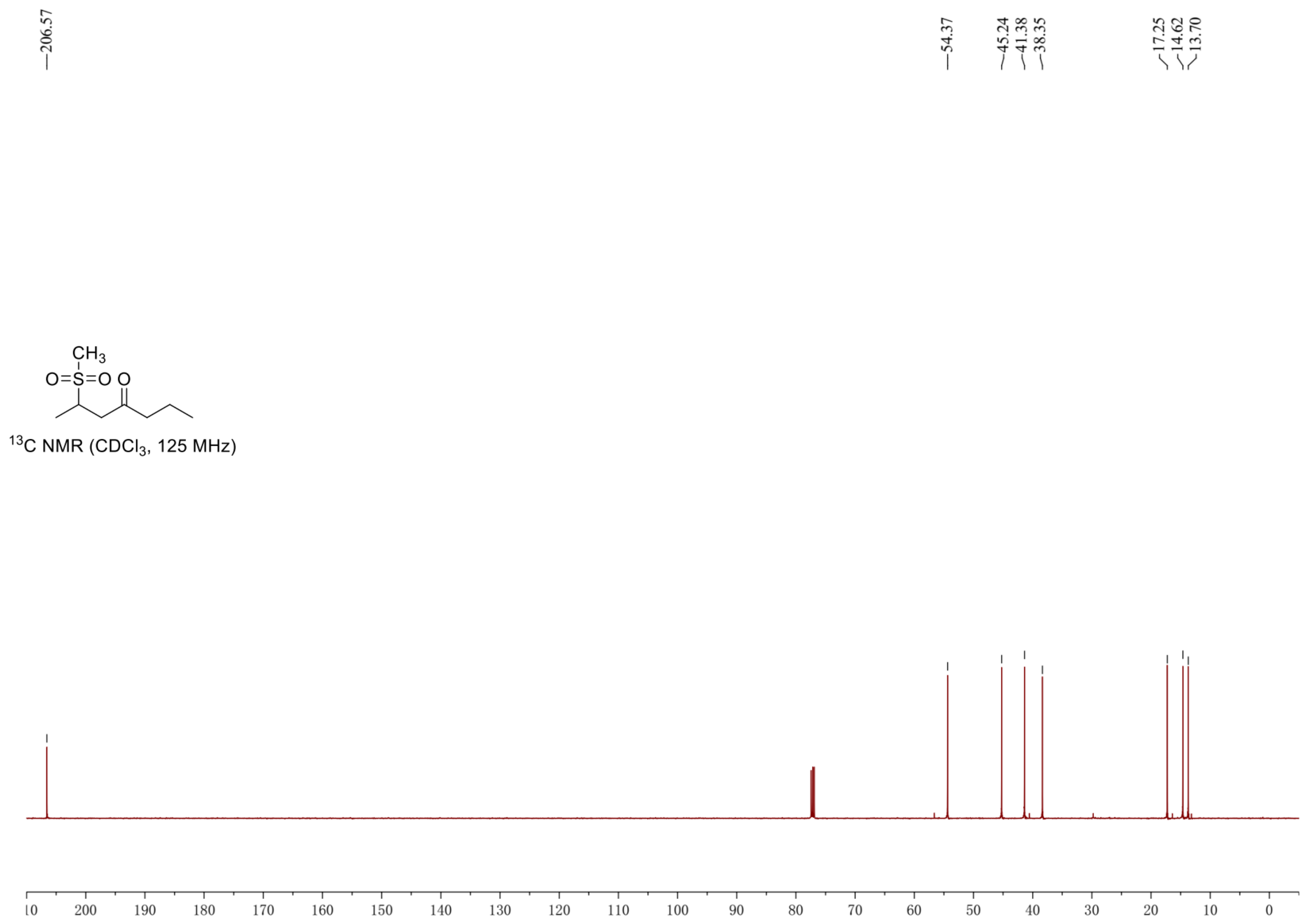

Go back to table of contents

Methyl (1*R,2*R**)-2-(allylsulfonyl)cyclopentane-1-carboxylate (S3)**

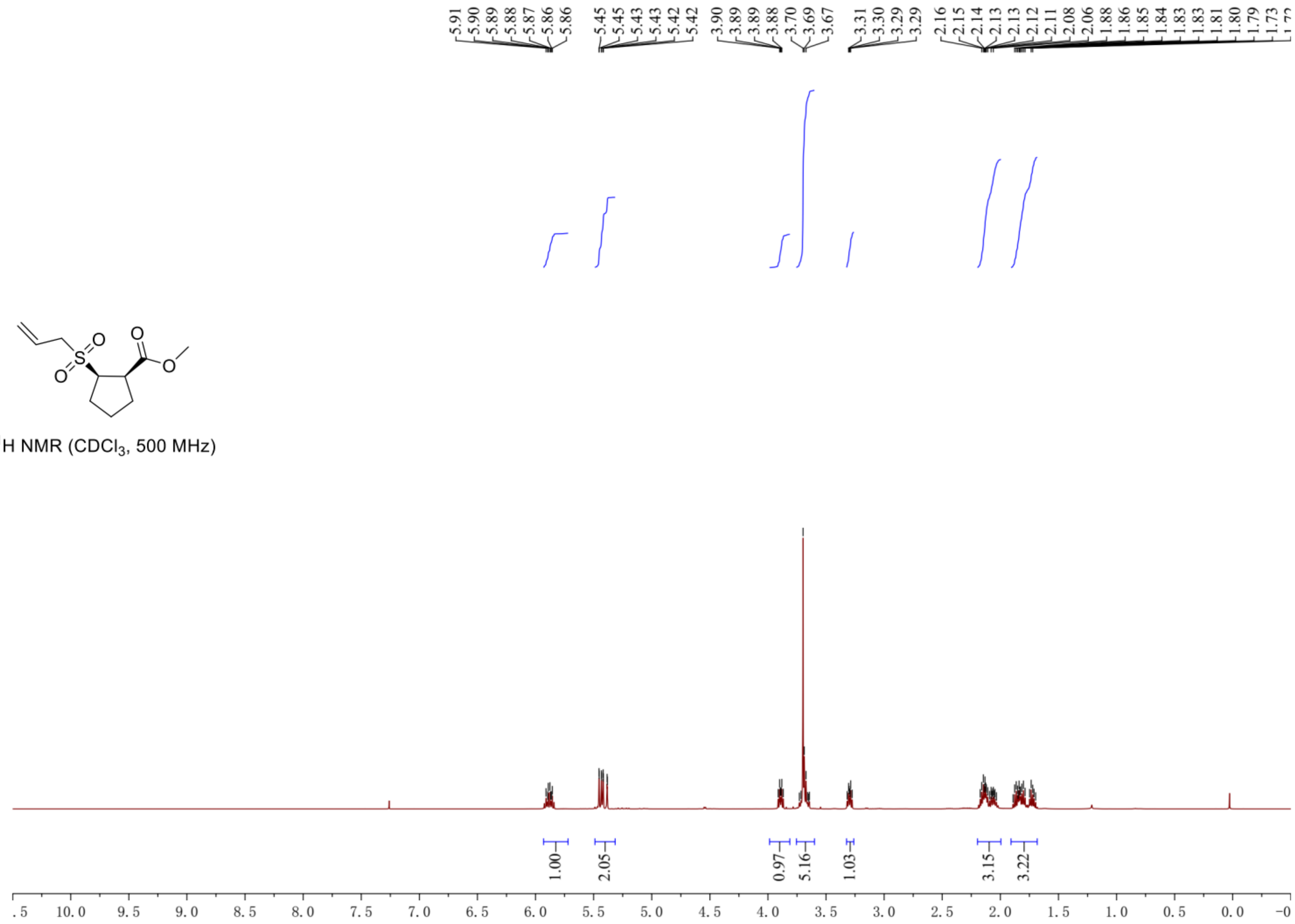

^{1}H NMR ($CDCl_3$, 500 MHz)

Go back to table of contents

Methyl (1*R,2*R**)-2-(allylsulfonyl)cyclopentane-1-carboxylate (S3)**

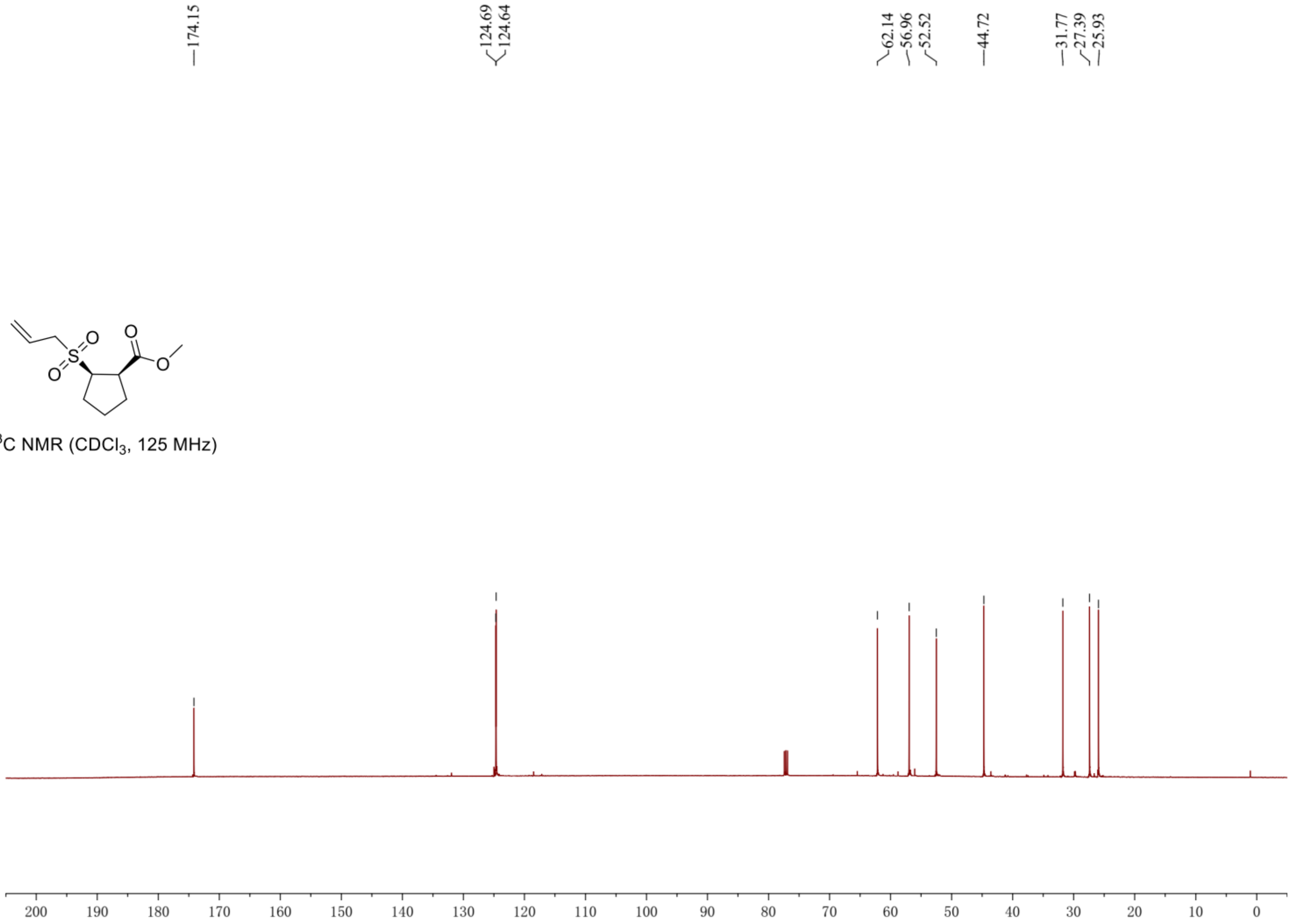

^{13}C NMR ($CDCl_3$, 125 MHz)

Go back to table of contents

Methyl (1*S**,3*S**)-3-(allylsulfonyl)cyclopentane-1-carboxylate (S4)

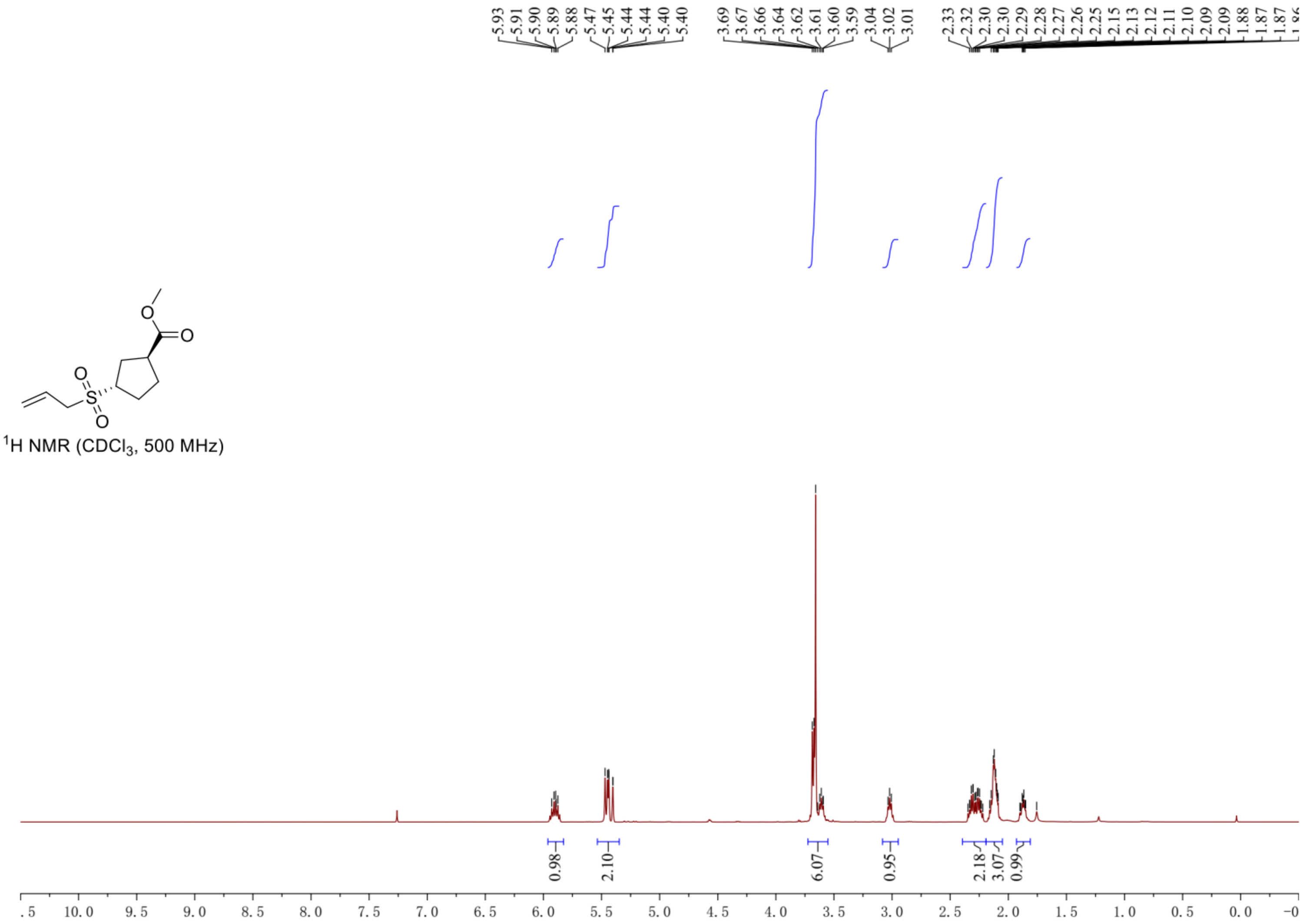

[Go back to table of contents](#)

Methyl (1*S**,3*S**)-3-(allylsulfonyl)cyclopentane-1-carboxylate (S4)

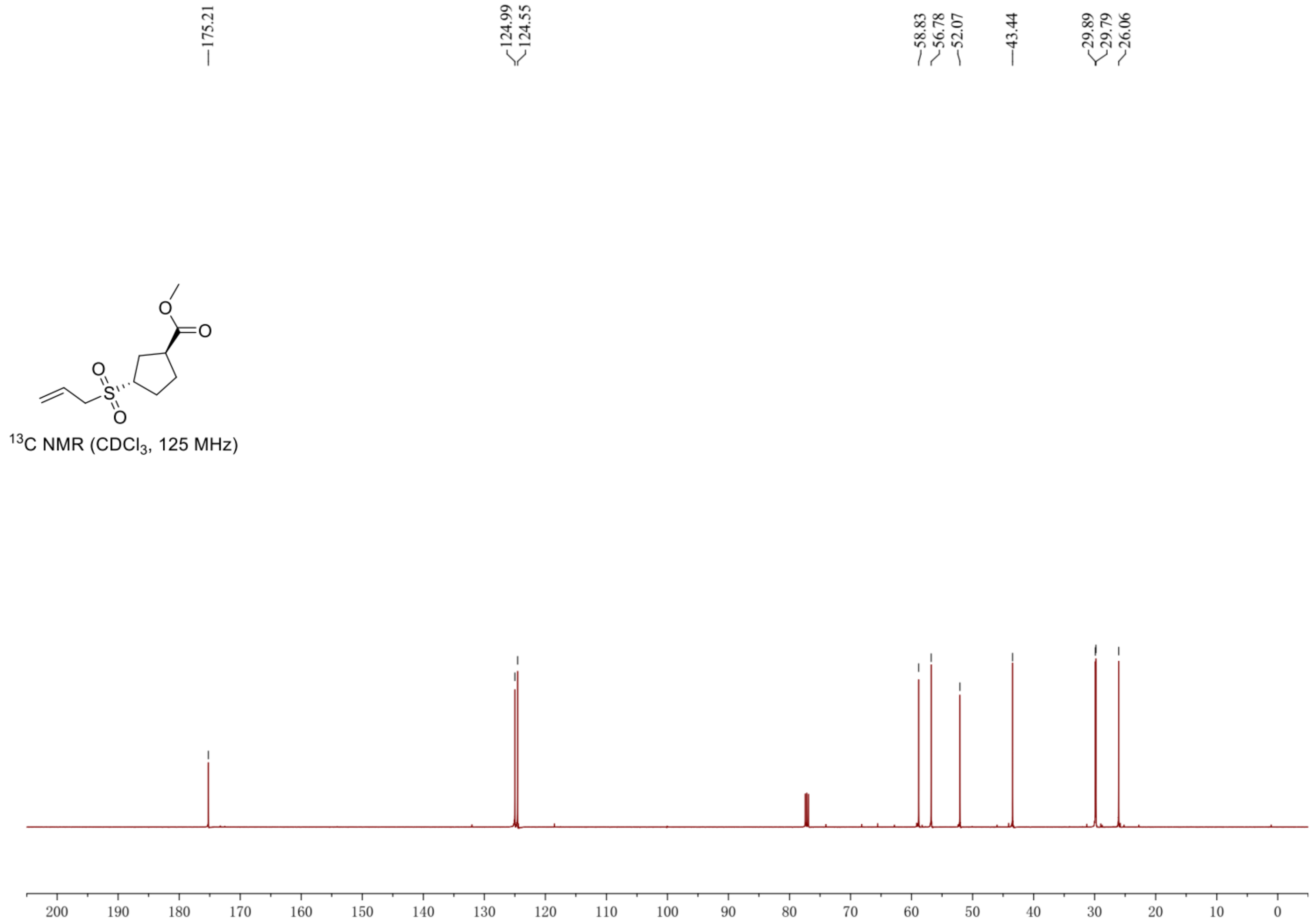

Go back to table of contents

Methyl (1*S,3*R**)-3-(allylsulfonyl)cyclopentane-1-carboxylate (S5)**

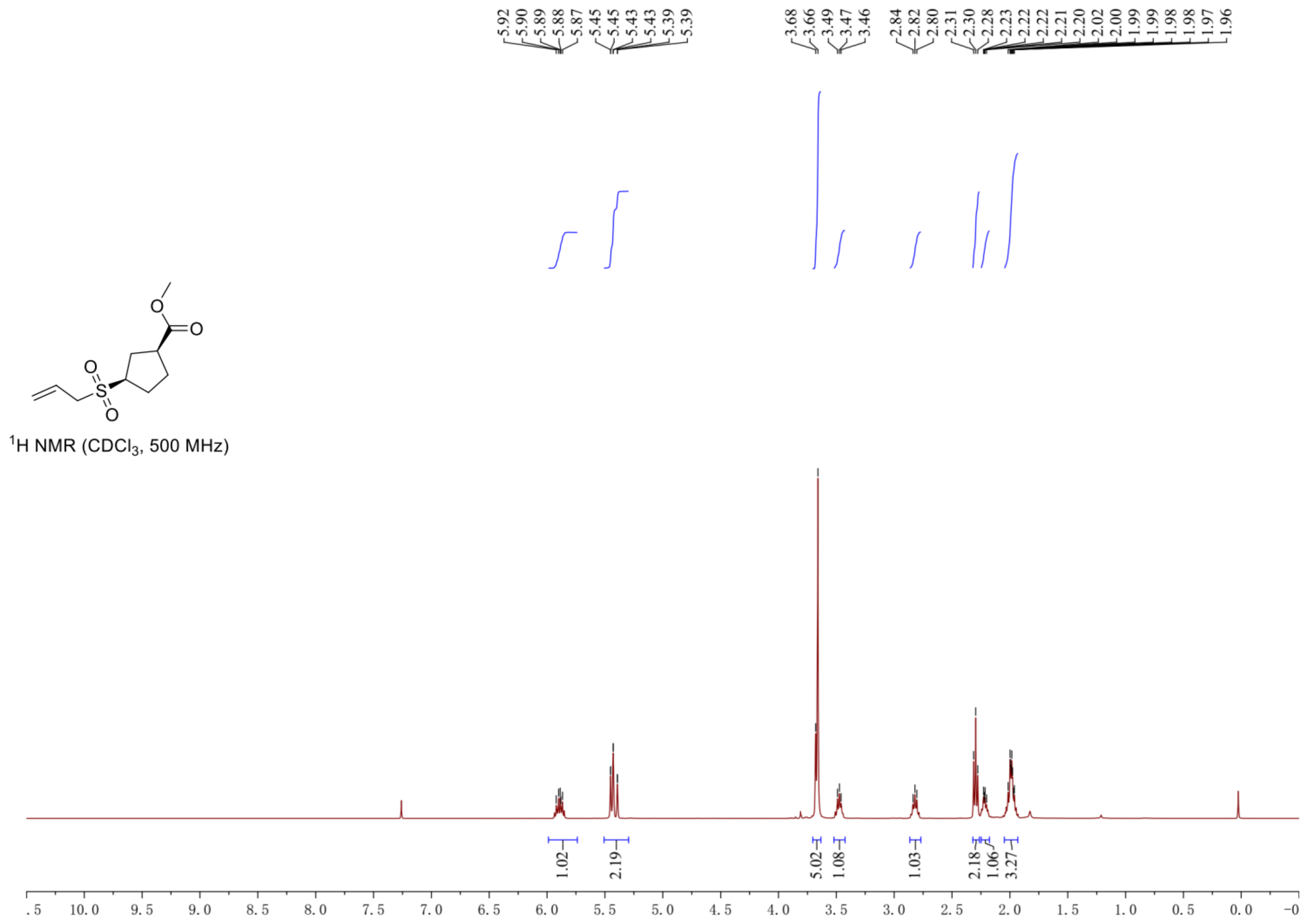

Go back to table of contents

Methyl (1*S,3*R**)-3-(allylsulfonyl)cyclopentane-1-carboxylate (S5)**

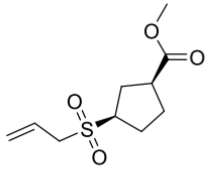

^{13}C NMR ($CDCl_3$, 125 MHz)

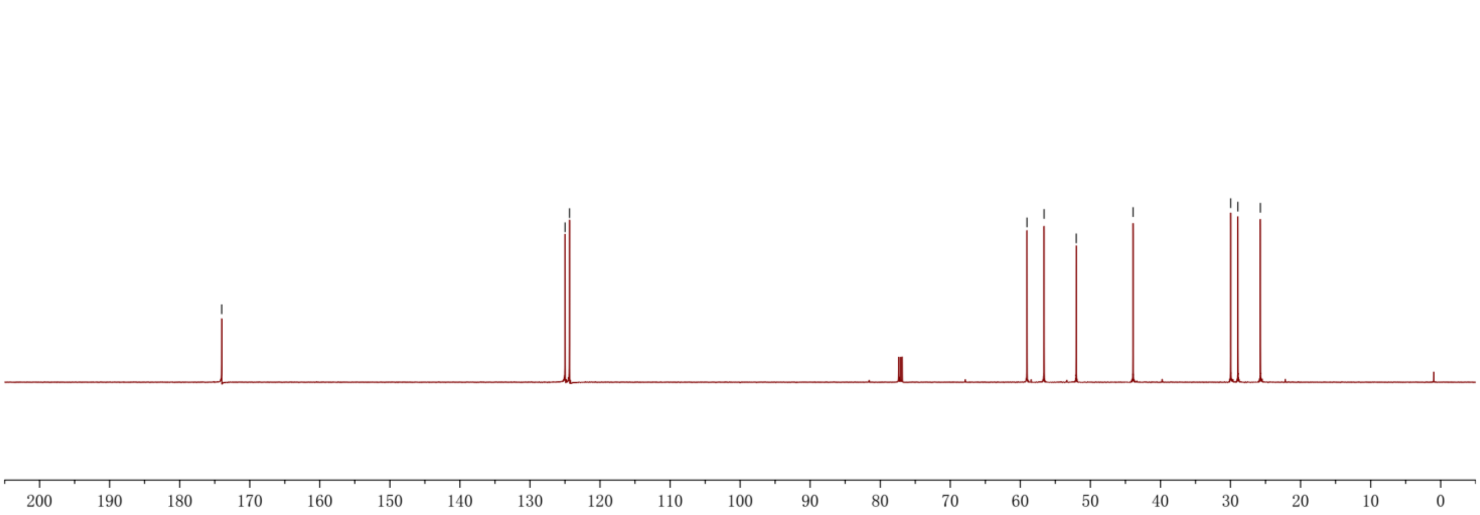

Go back to table of contents

Cycloheptanesulfinic acid (37)

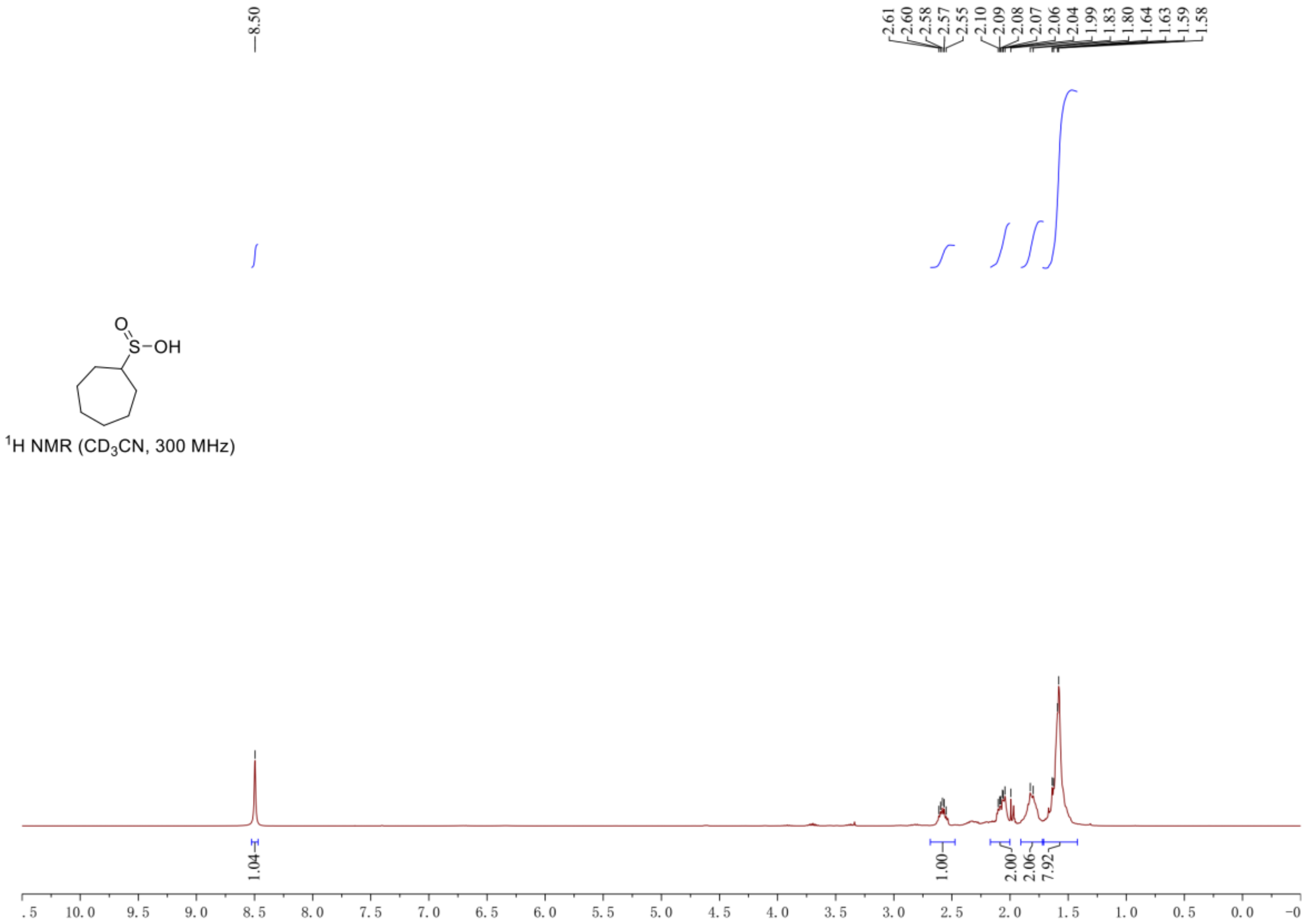

Go back to table of contents

Cycloheptanesulfinic acid (37)

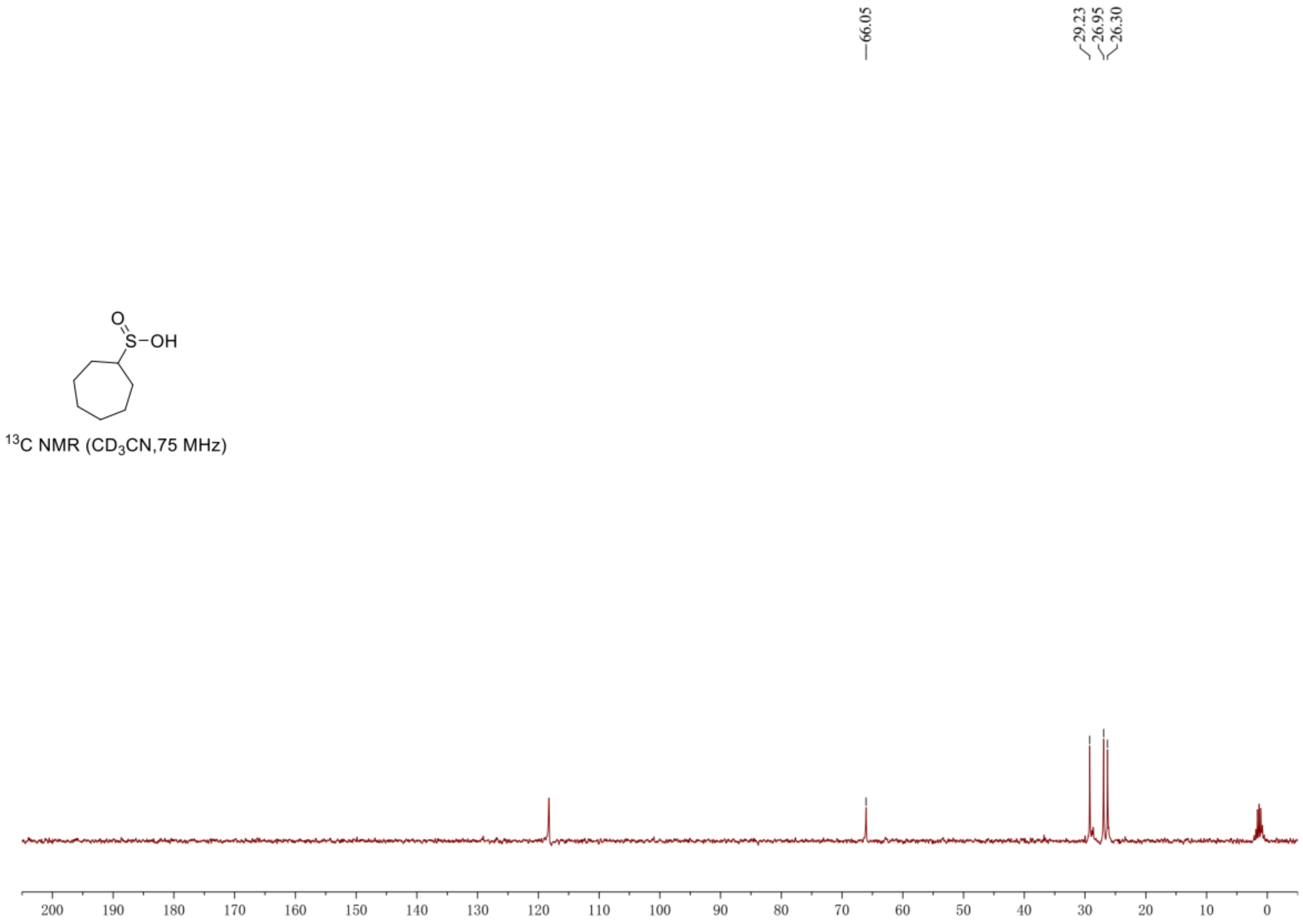

Go back to table of contents

Sodium cycloheptanesulfinate (38)

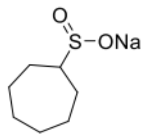

^{1}H NMR (CD_3OD, 300 MHz)

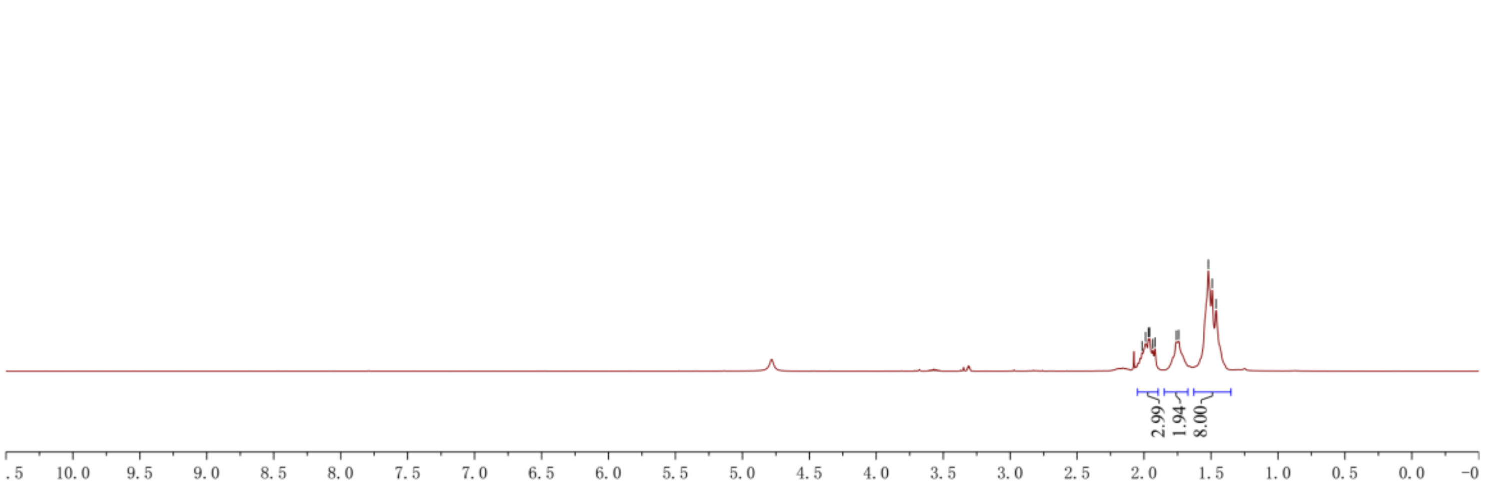

Go back to table of contents

Sodium cycloheptanesulfinate (38)

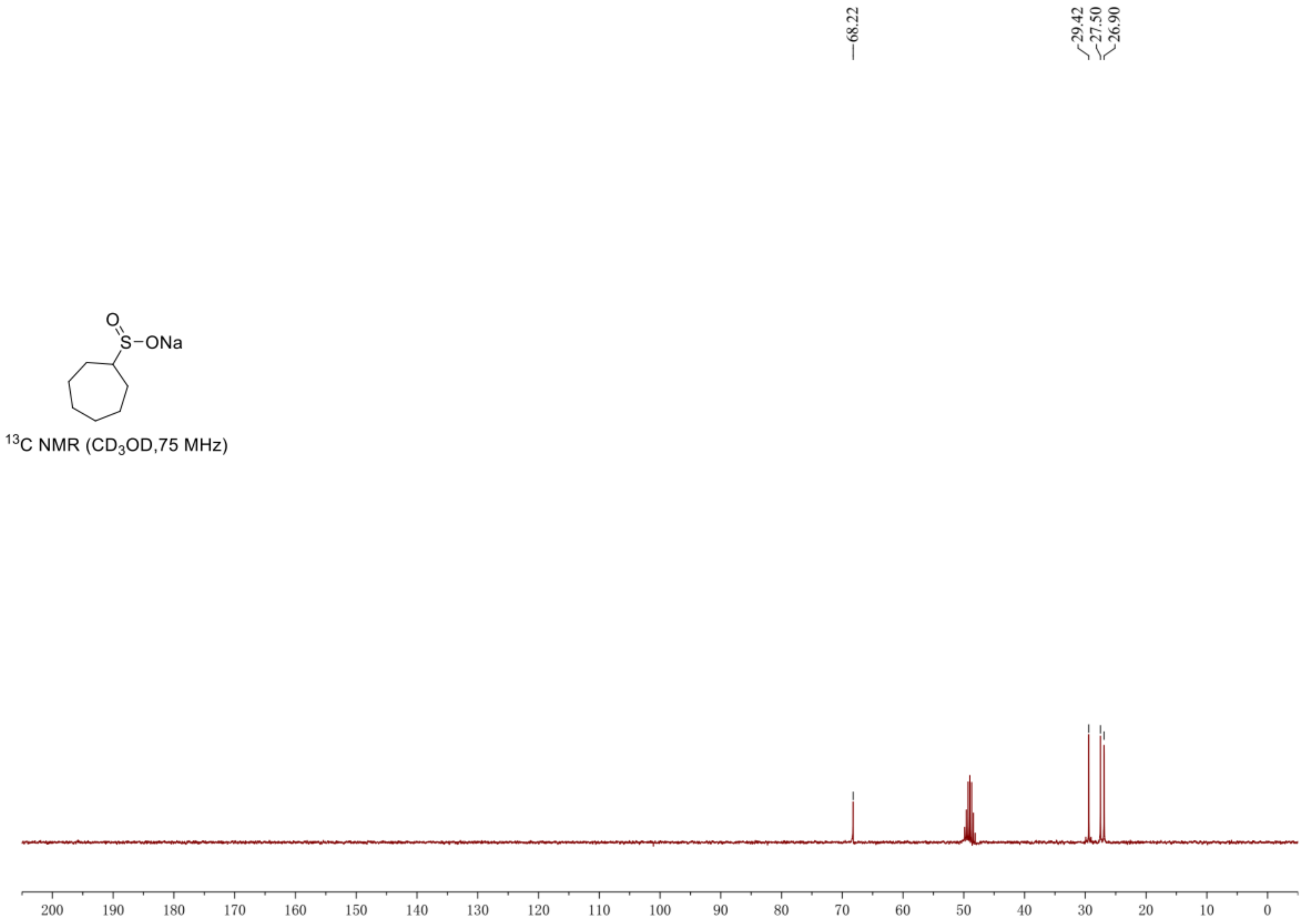

Go back to table of contents

4-(Cyclohexylsulfonyl)morpholine (39)

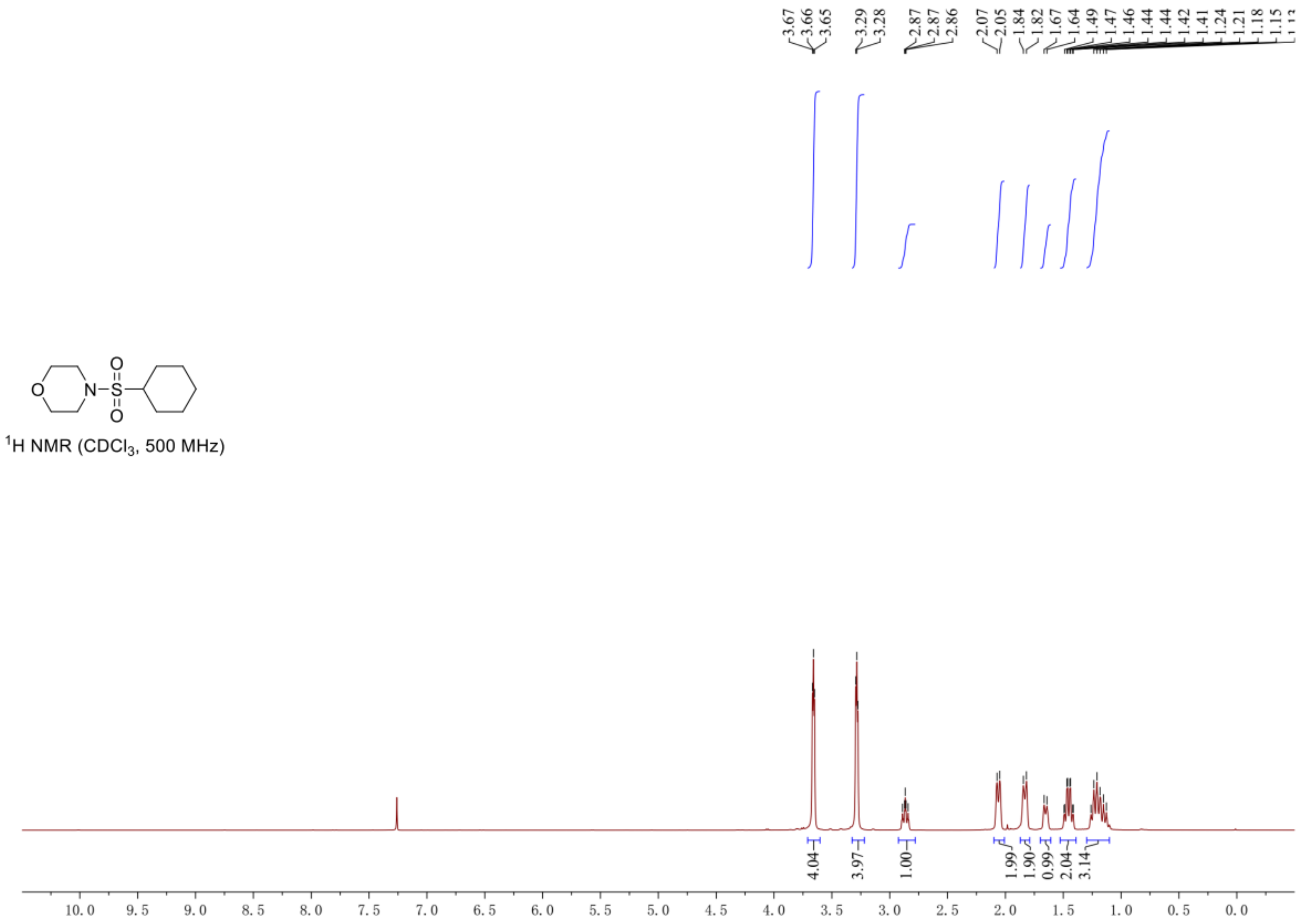

4-(Cyclohexylsulfonyl)morpholine (39)

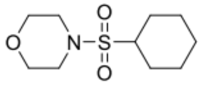

^{13}C NMR ($CDCl_3$, 125 MHz)

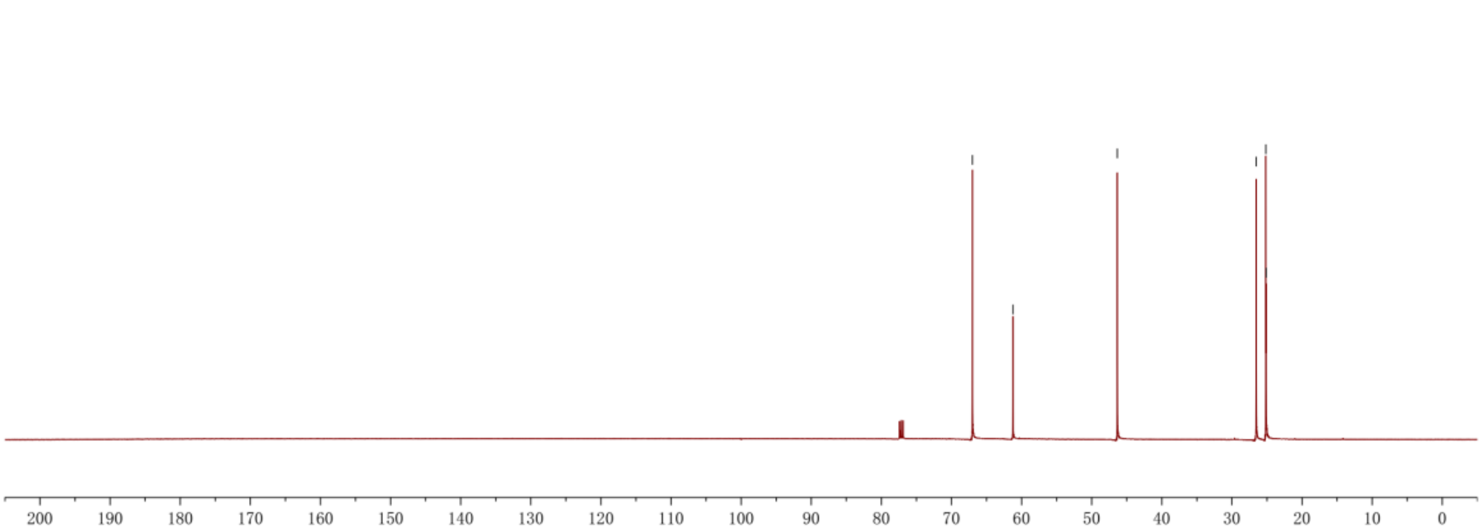

Go back to table of contents

2-(Cyclopentylsulfonyl)-1,2,3,4-tetrahydroisoquinoline (40)

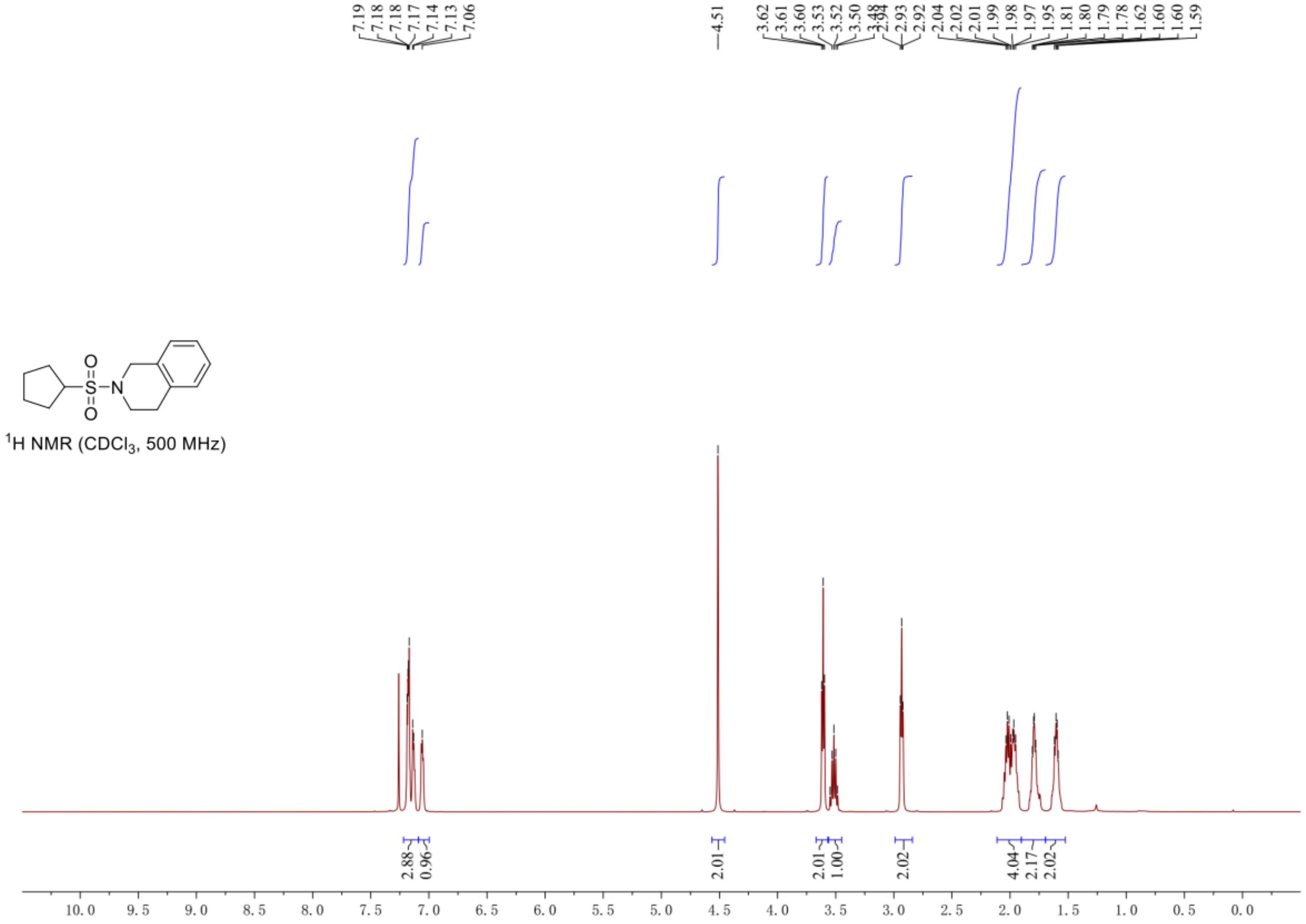

Go back to table of contents

2-(Cyclopentylsulfonyl)-1,2,3,4-tetrahydroisoquinoline (40)

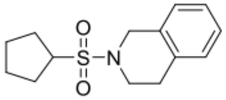

^{13}C NMR (CDCl$_3$, 125 MHz)

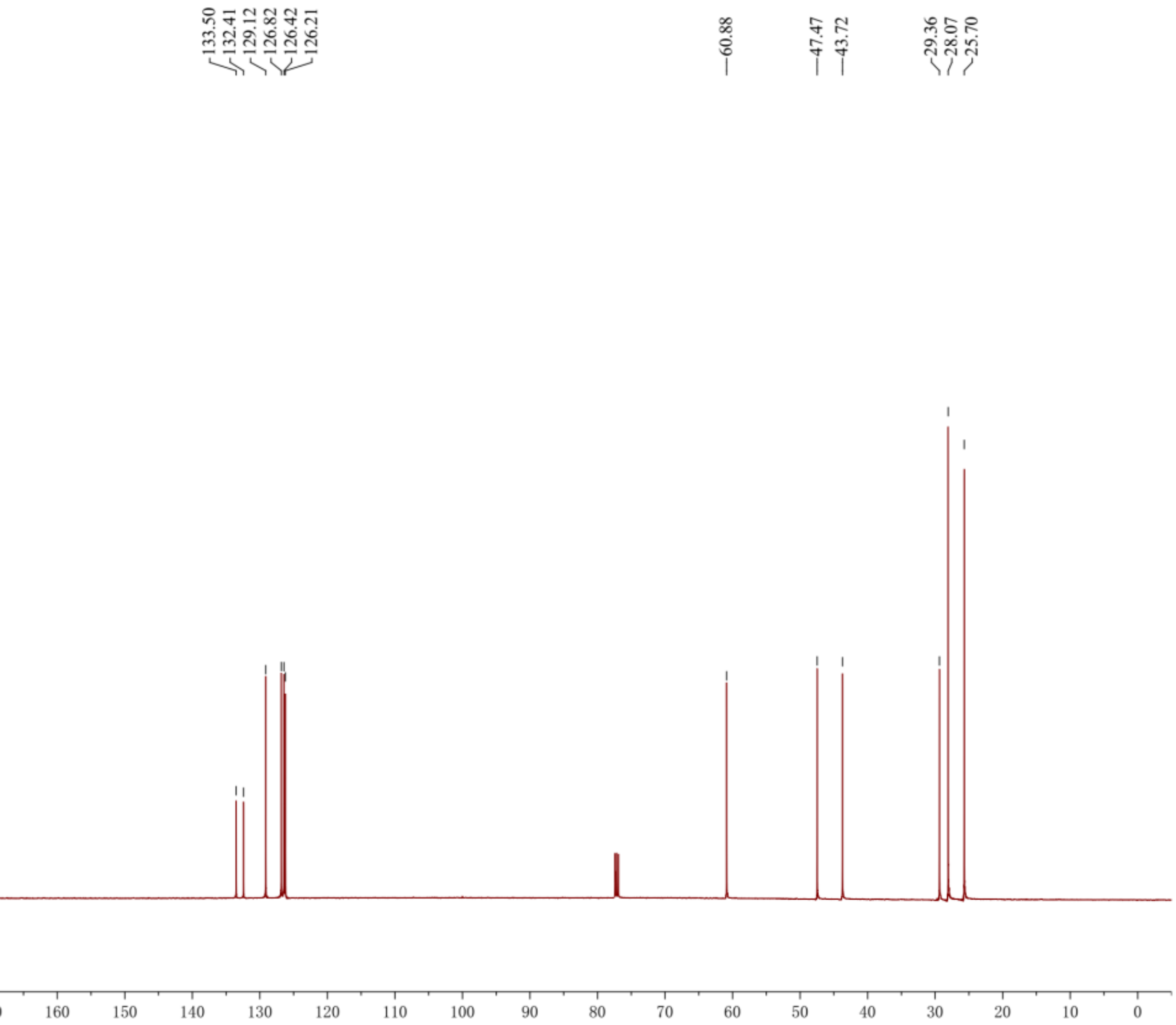

Go back to table of contents

(*R*)-*N*-(1-(4-Methoxyphenyl)ethyl)cyclopentanesulfonamide (41)

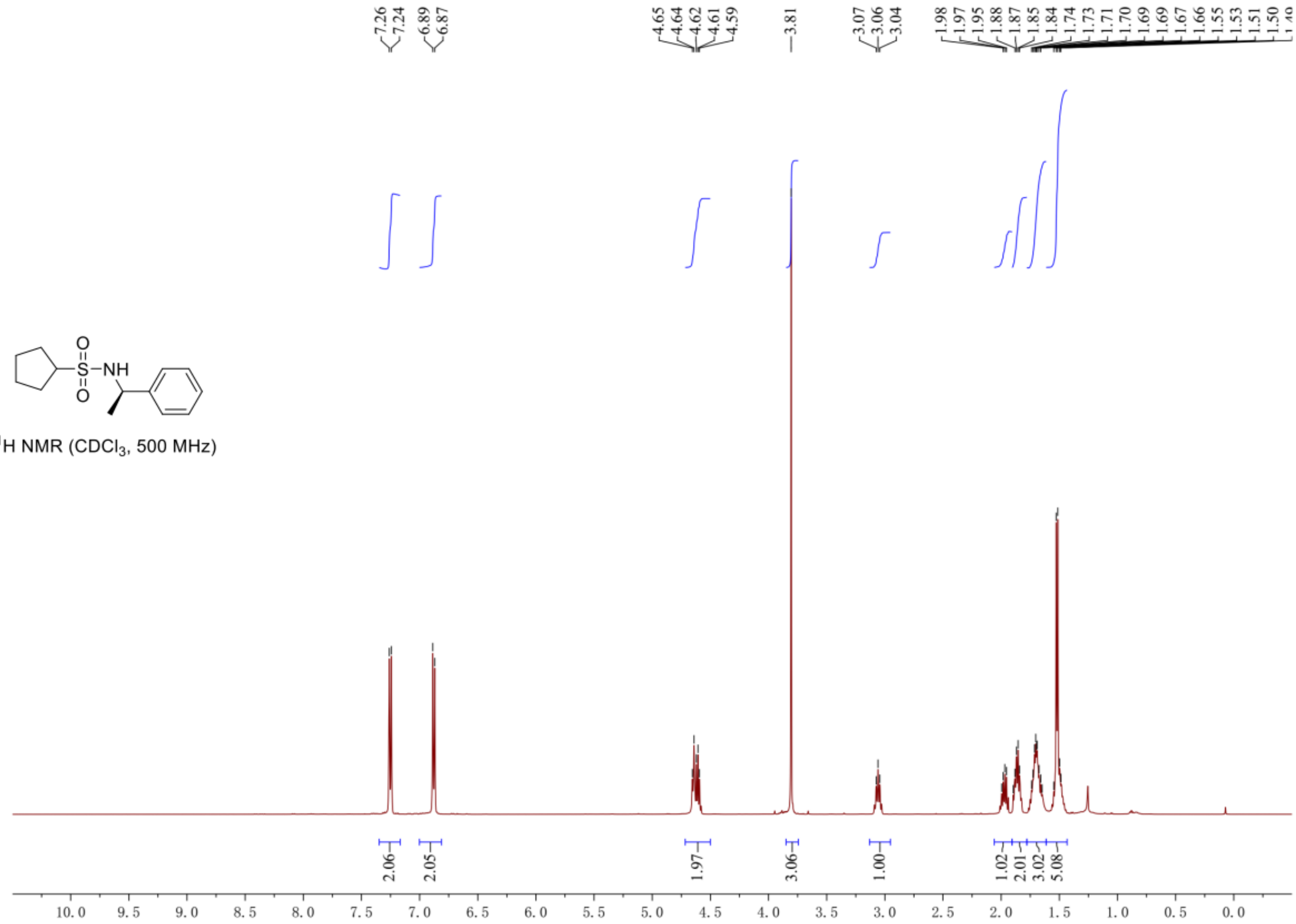

Go back to table of contents

(*R*)-*N*-(1-(4-Methoxyphenyl)ethyl)cyclopentanesulfonamide (41)

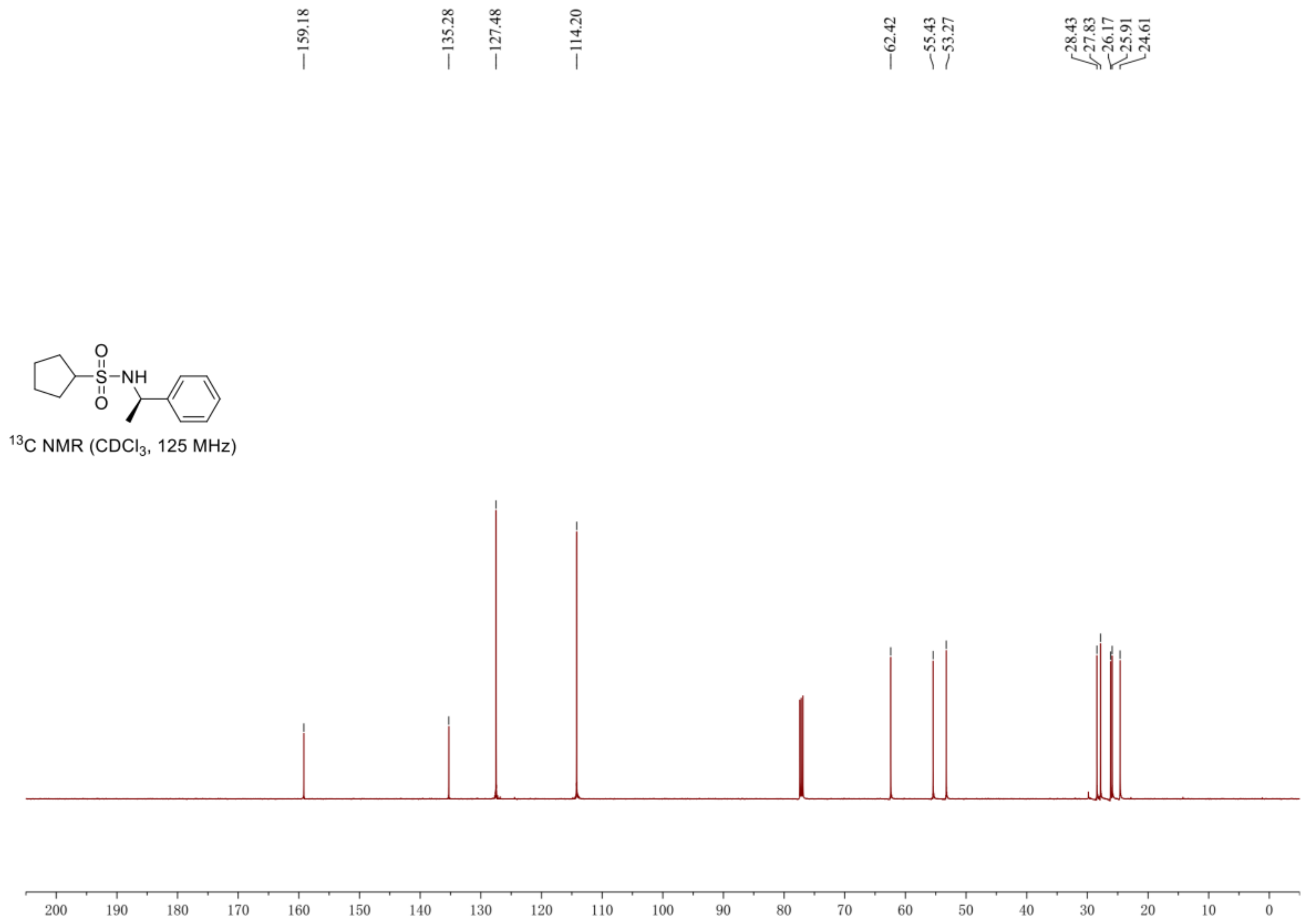

Go back to table of contents

2,6-Dimethyl-1-(morpholinosulfonyl)heptan-4-one (42)

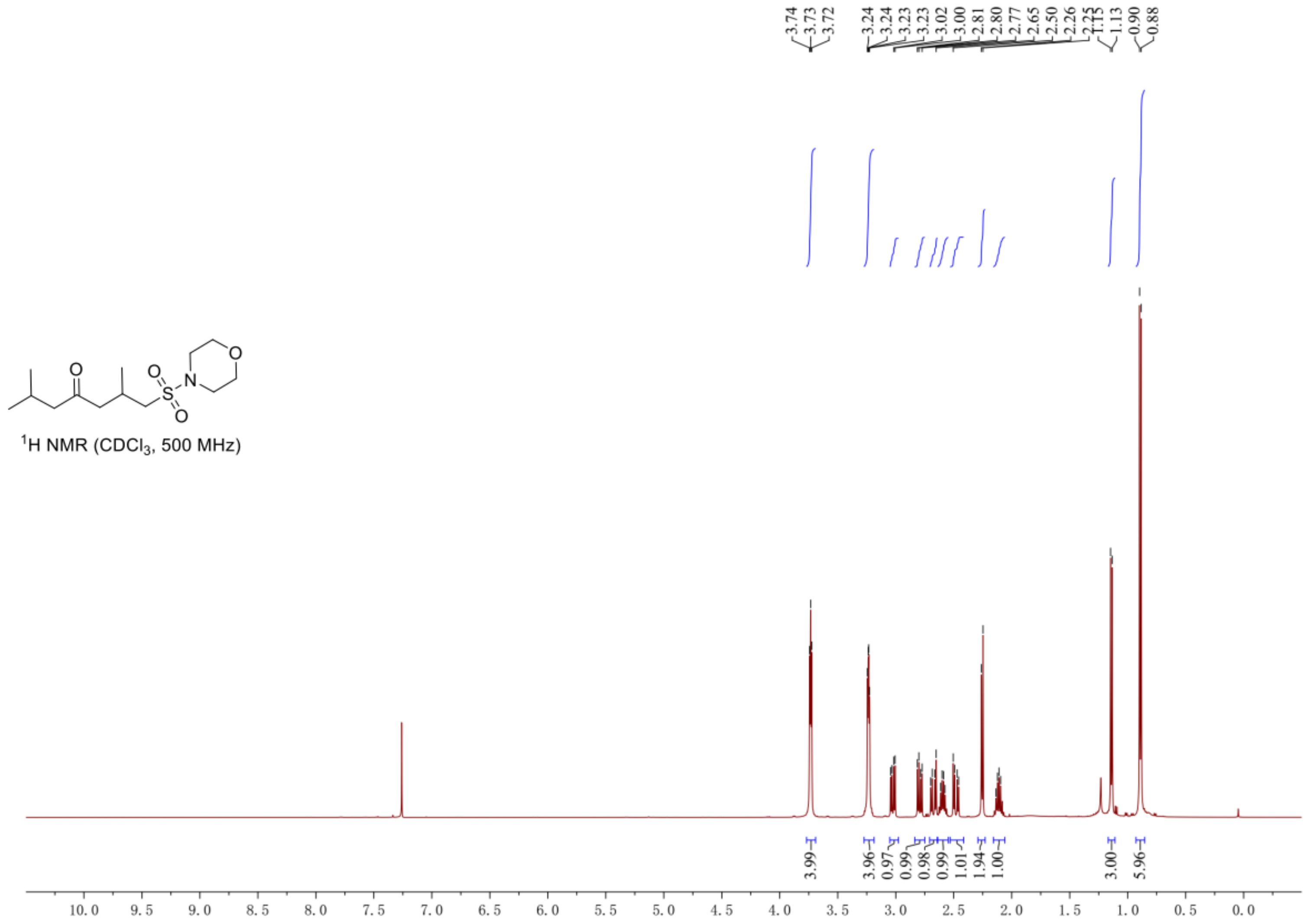

Go back to table of contents

2,6-Dimethyl-1-(morpholinosulfonyl)heptan-4-one (42)

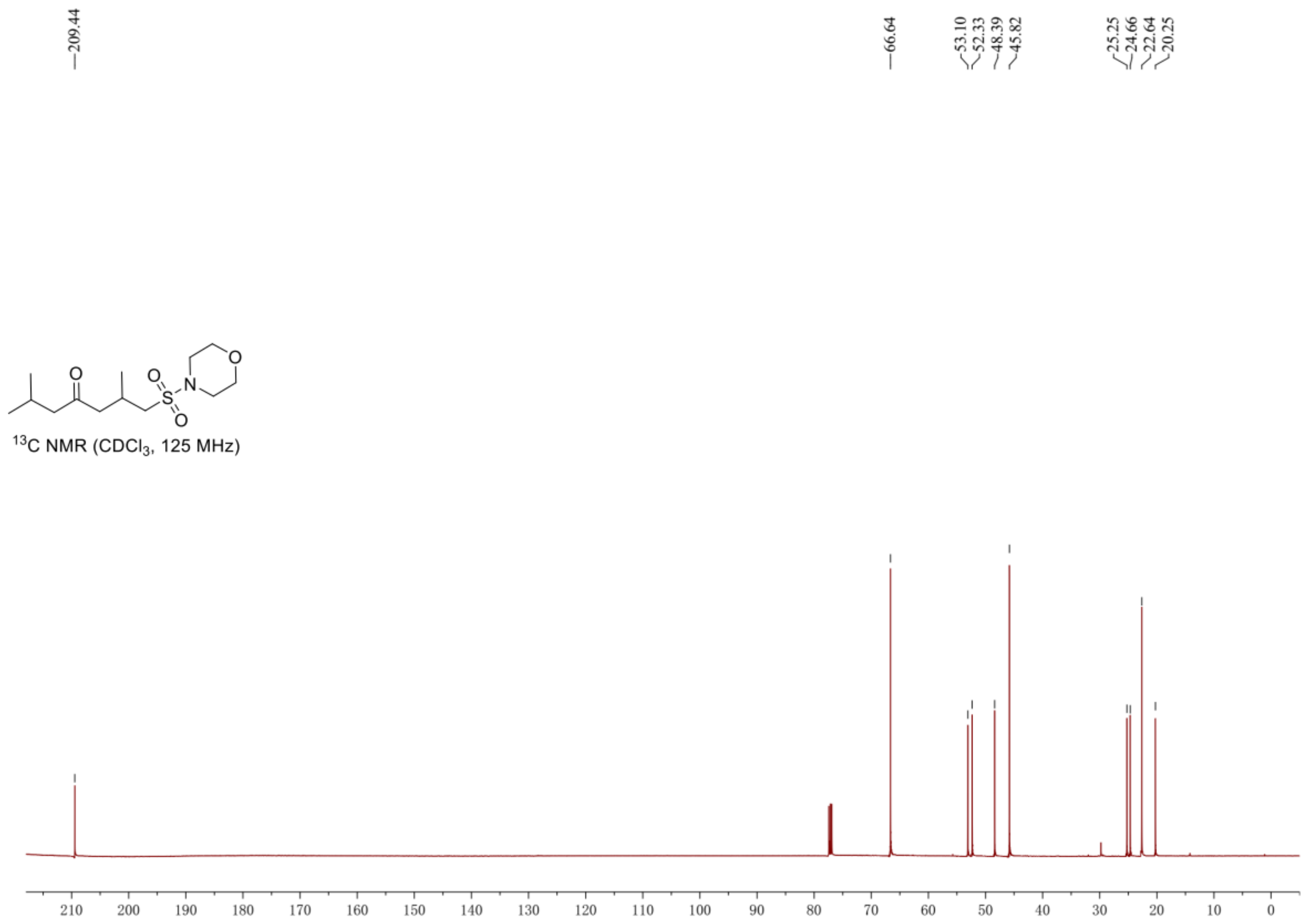

Go back to table of contents

1-((3,4-Dihydroisoquinolin-2(1*H*)-yl)sulfonyl)-2,6-dimethylheptan-4-one (43)

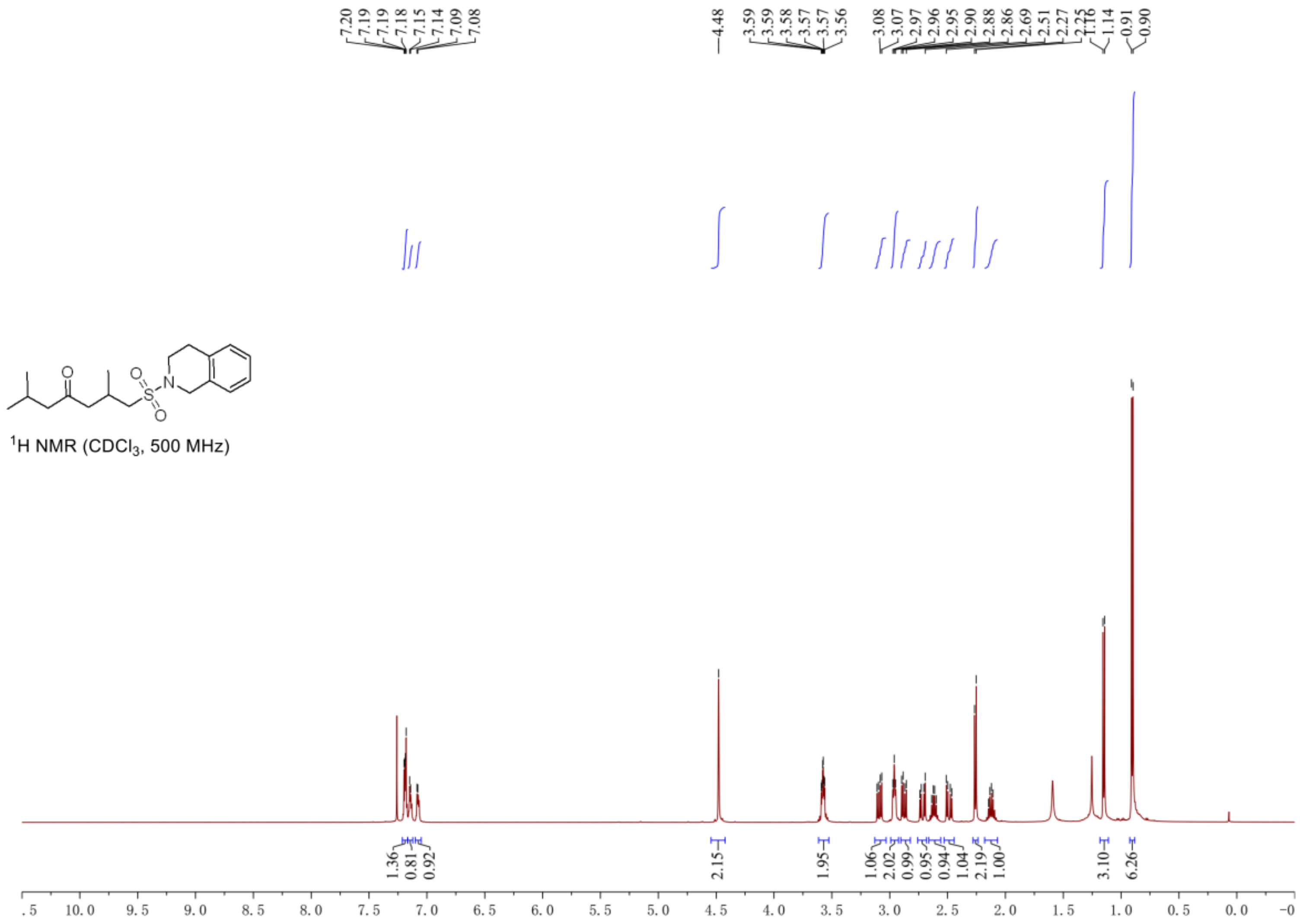

Go back to table of contents

1-((3,4-Dihydroisoquinolin-2(1*H*)-yl)sulfonyl)-2,6-dimethylheptan-4-one (43)

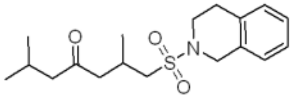

^{13}C NMR ($CDCl_3$, 125 MHz)

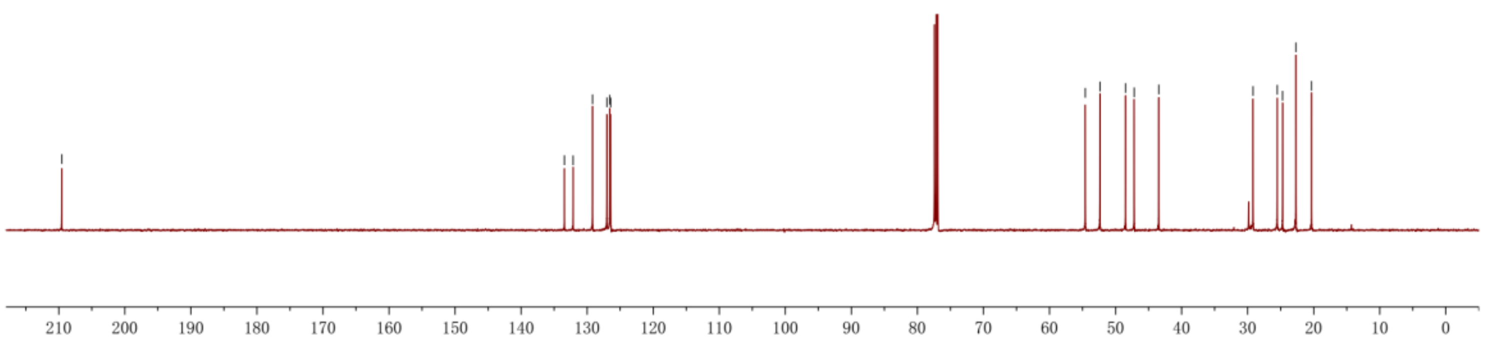

Go back to table of contents

2,6-Dimethyl-4-oxoheptane-1-sulfonyl fluoride (44)

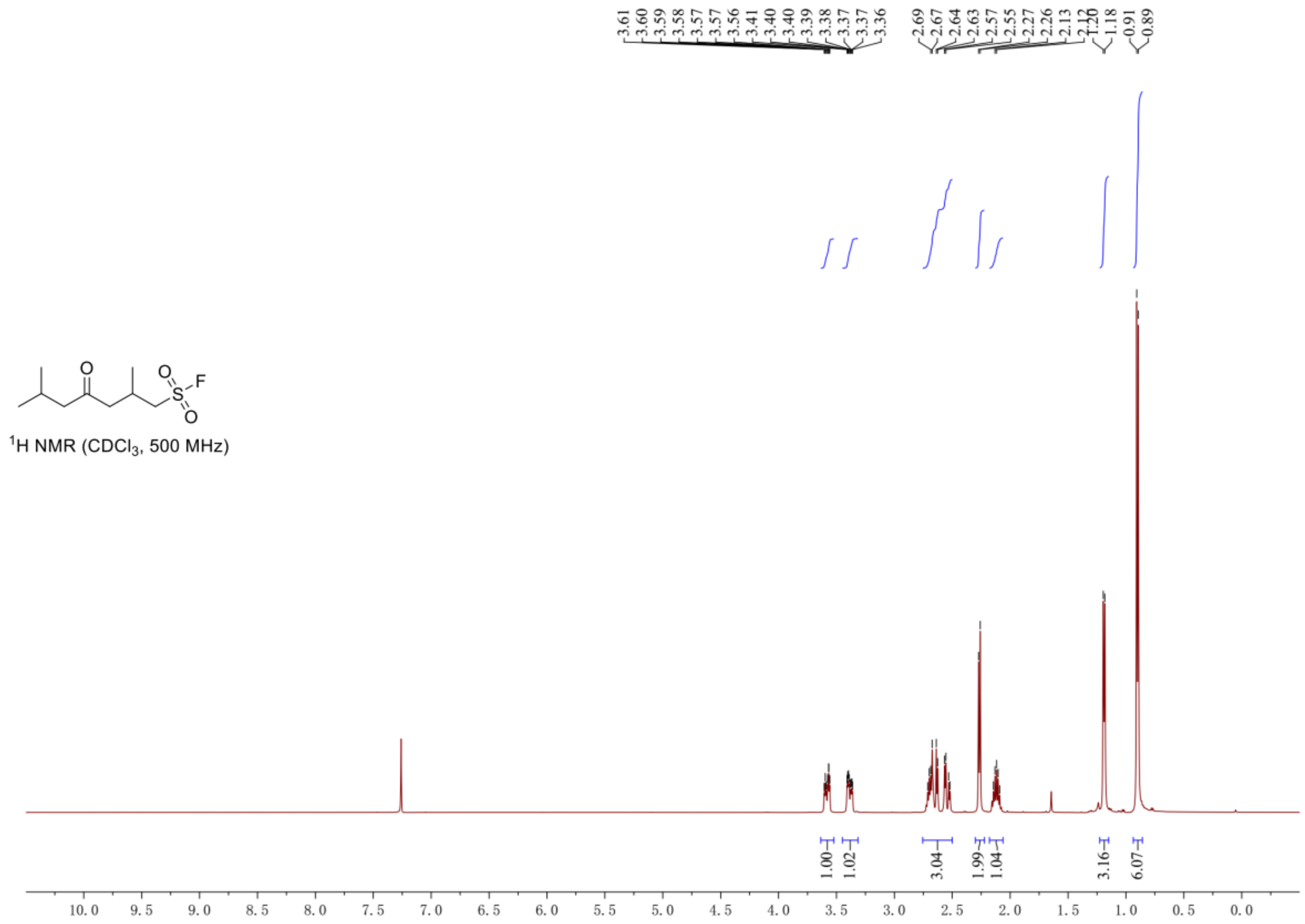

Go back to table of contents

2,6-Dimethyl-4-oxoheptane-1-sulfonyl fluoride (44)

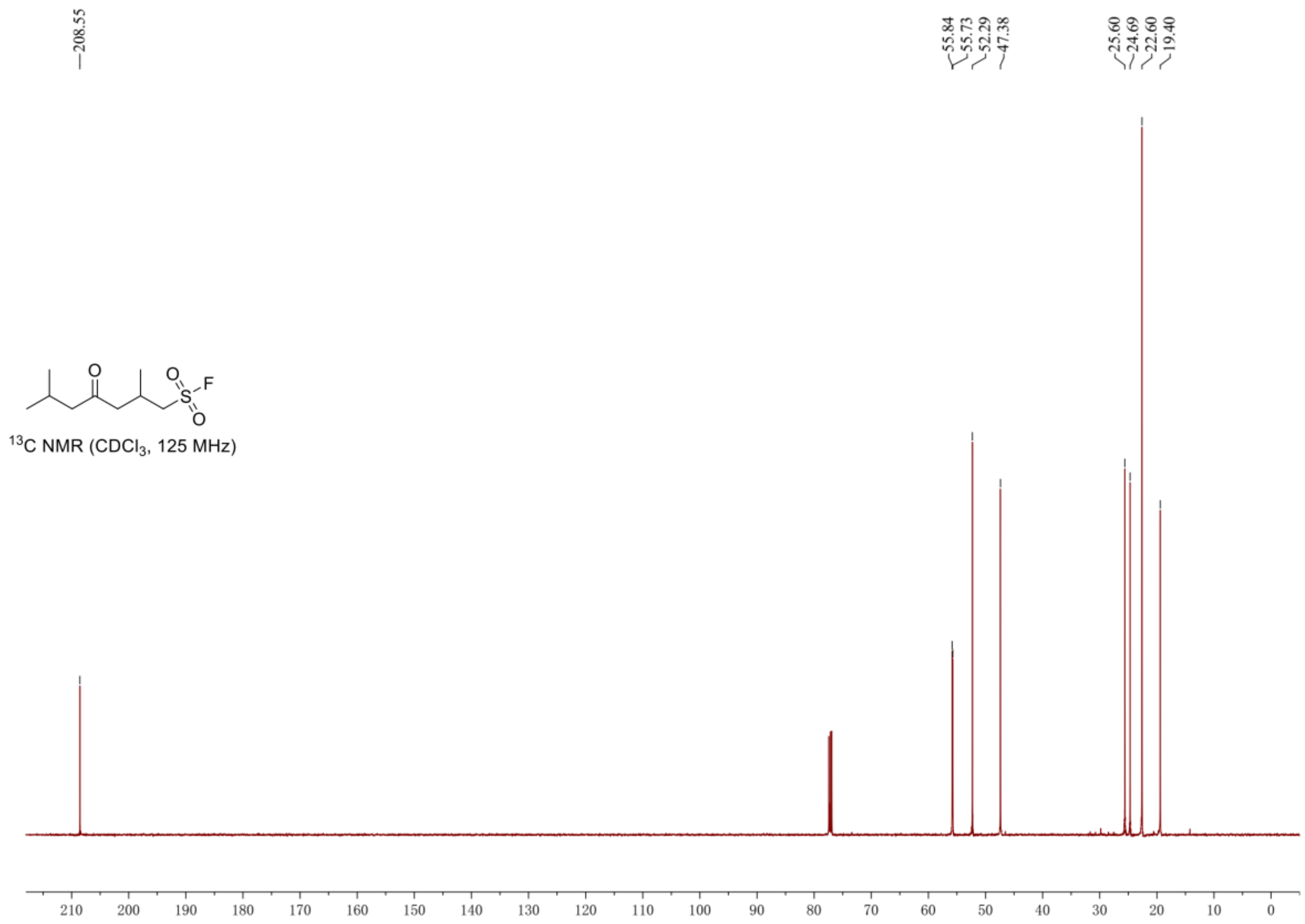

Go back to table of contents

3-Oxopentane-1-sulfonyl fluoride (45)

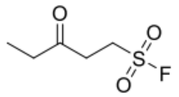

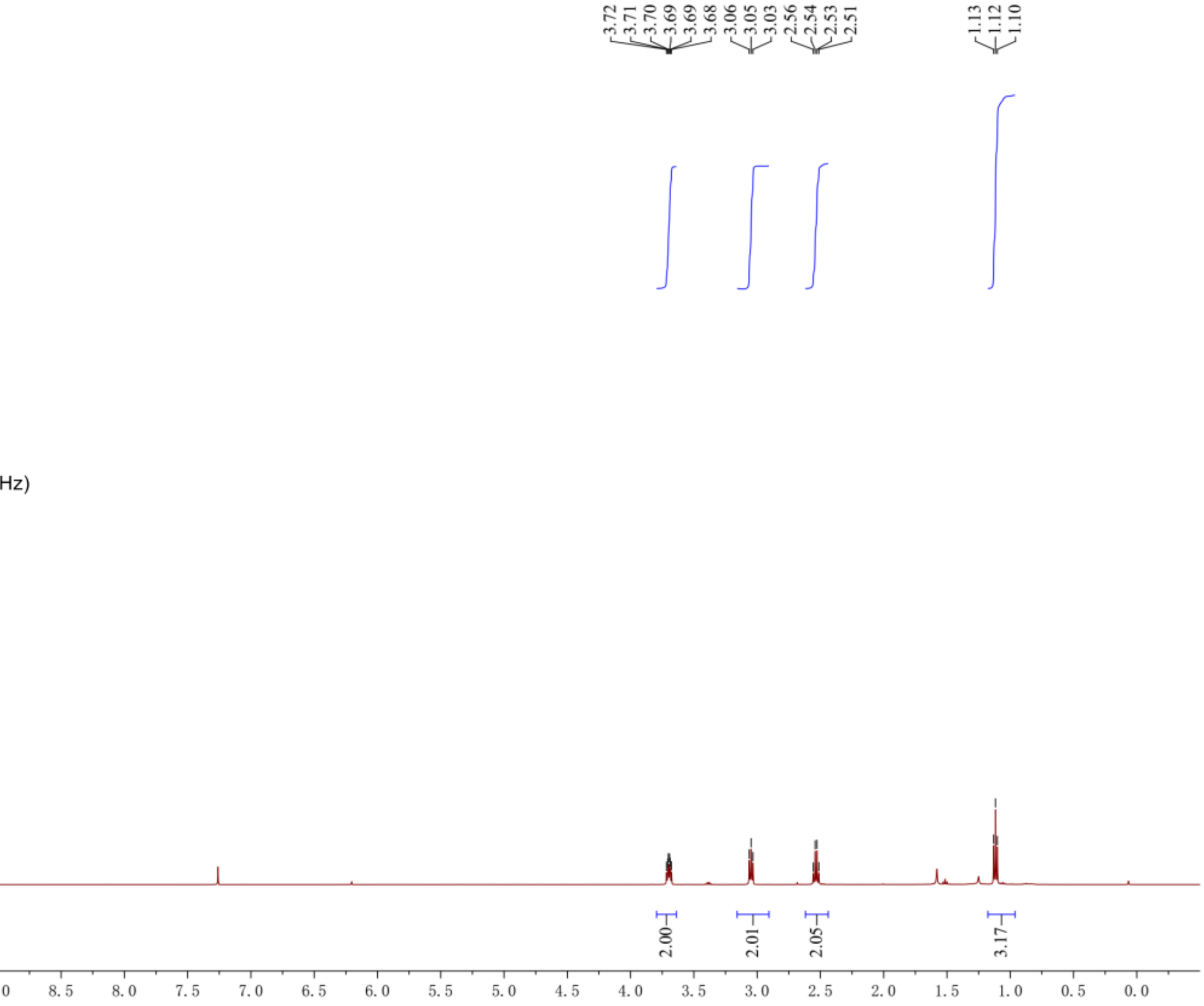

^{1}H NMR ($CDCl_3$, 500 MHz)

Go back to table of contents

3-Oxopentane-1-sulfonyl fluoride (45)

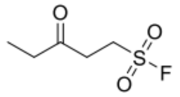

^{13}C NMR ($CDCl_3$, 125 MHz)

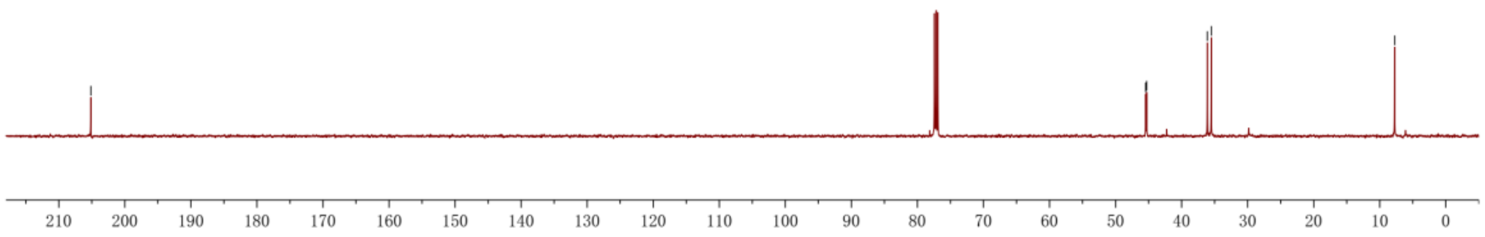

Go back to table of contents

7-Chloro-4-(cyclohexylsulfonyl)quinoline (46)

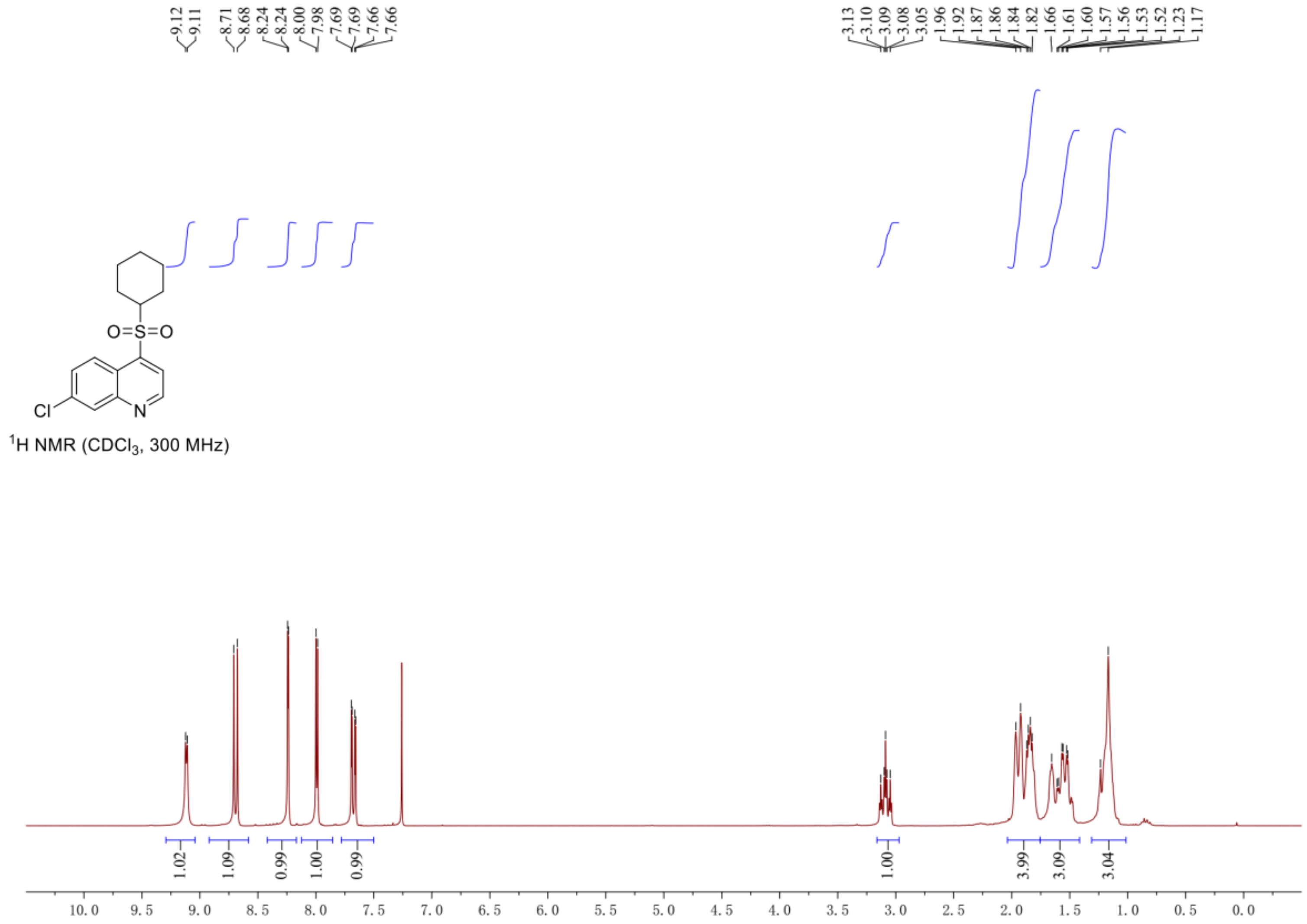

Go back to table of contents

7-Chloro-4-(cyclohexylsulfonyl)quinoline (46)

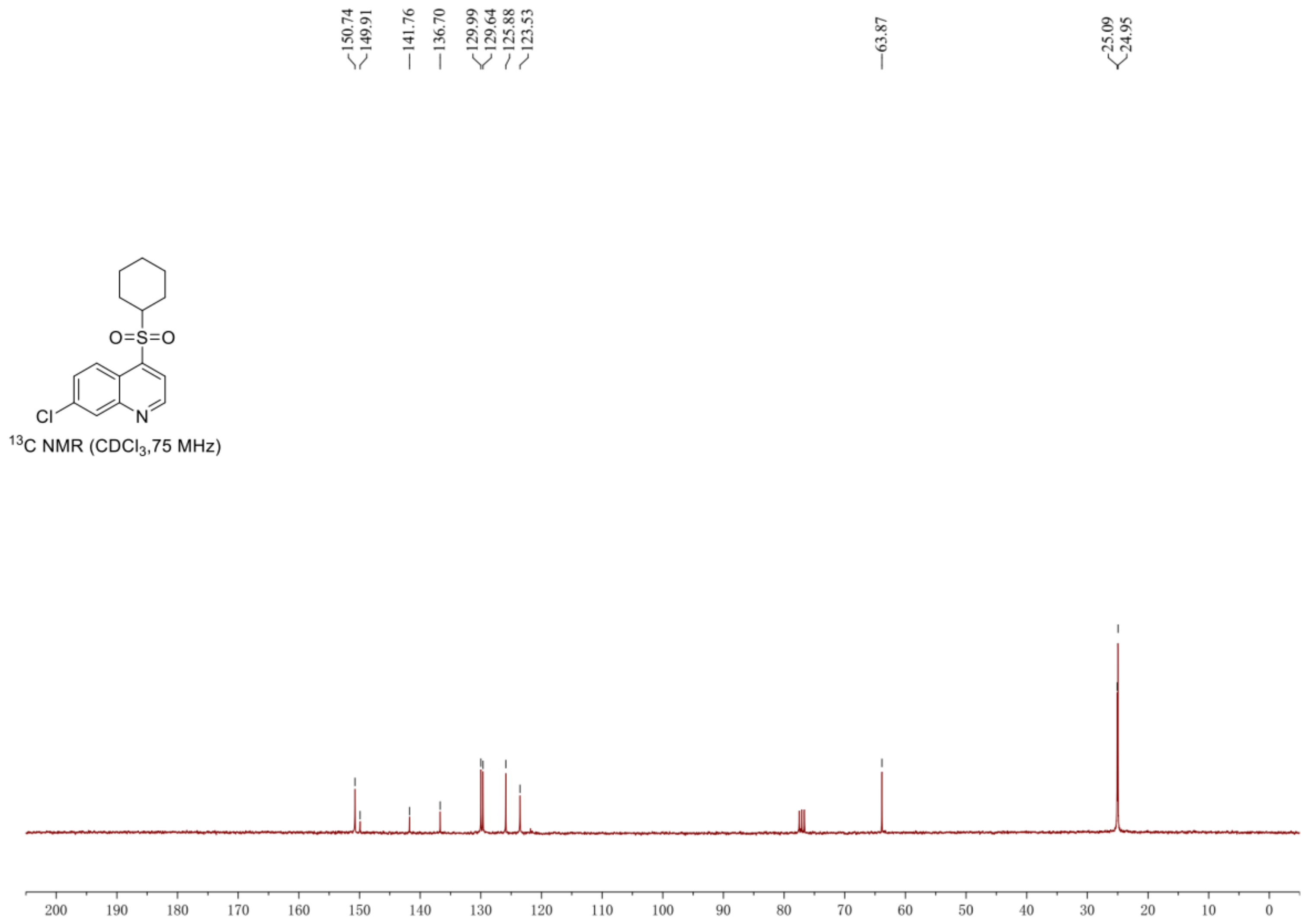

Go back to table of contents

Sodium cyclopentanesulfinate (47)

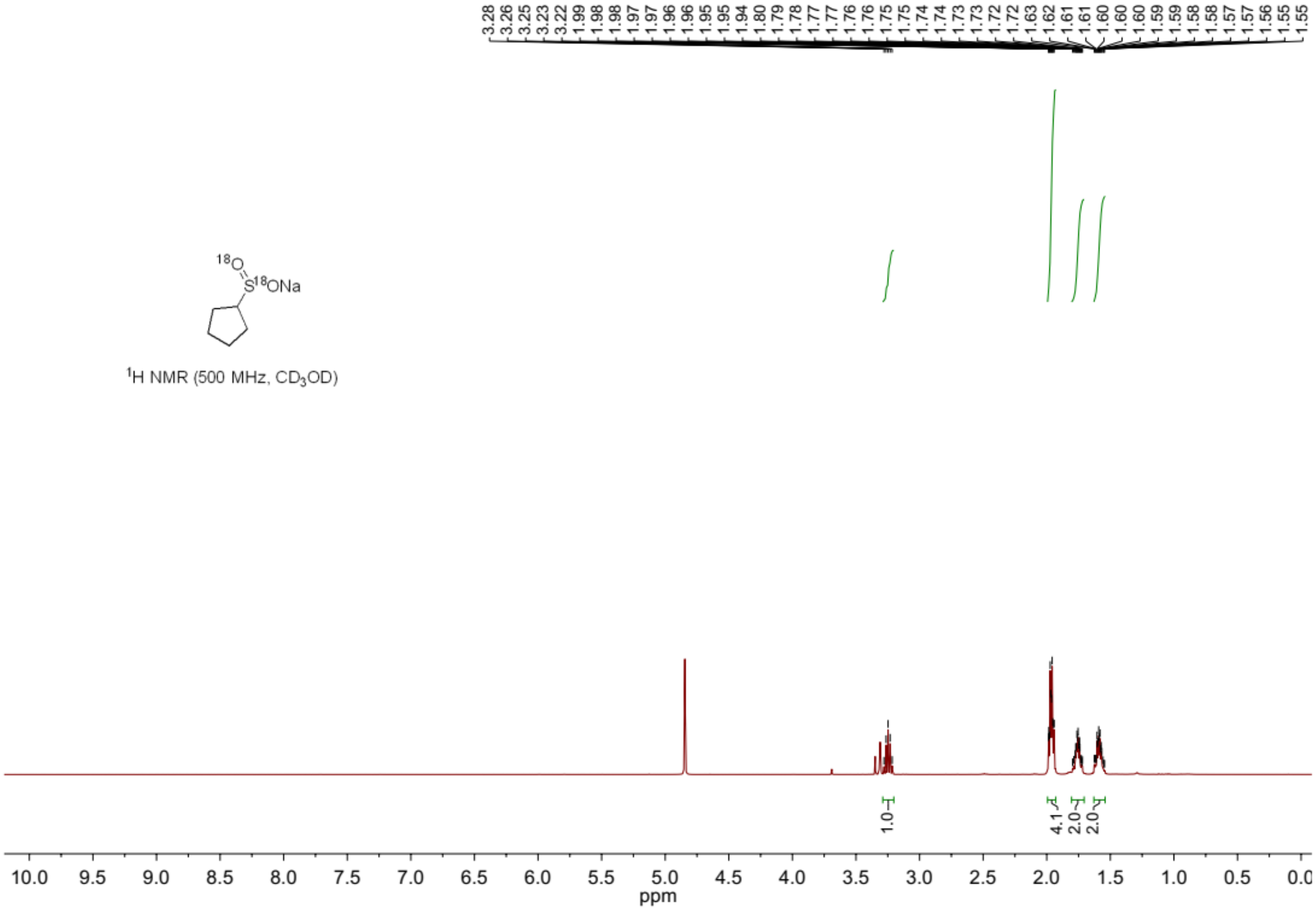

^{1}H NMR (500 MHz, CD_3OD)

Go back to table of contents

Sodium cyclopentanesulfinate (47)

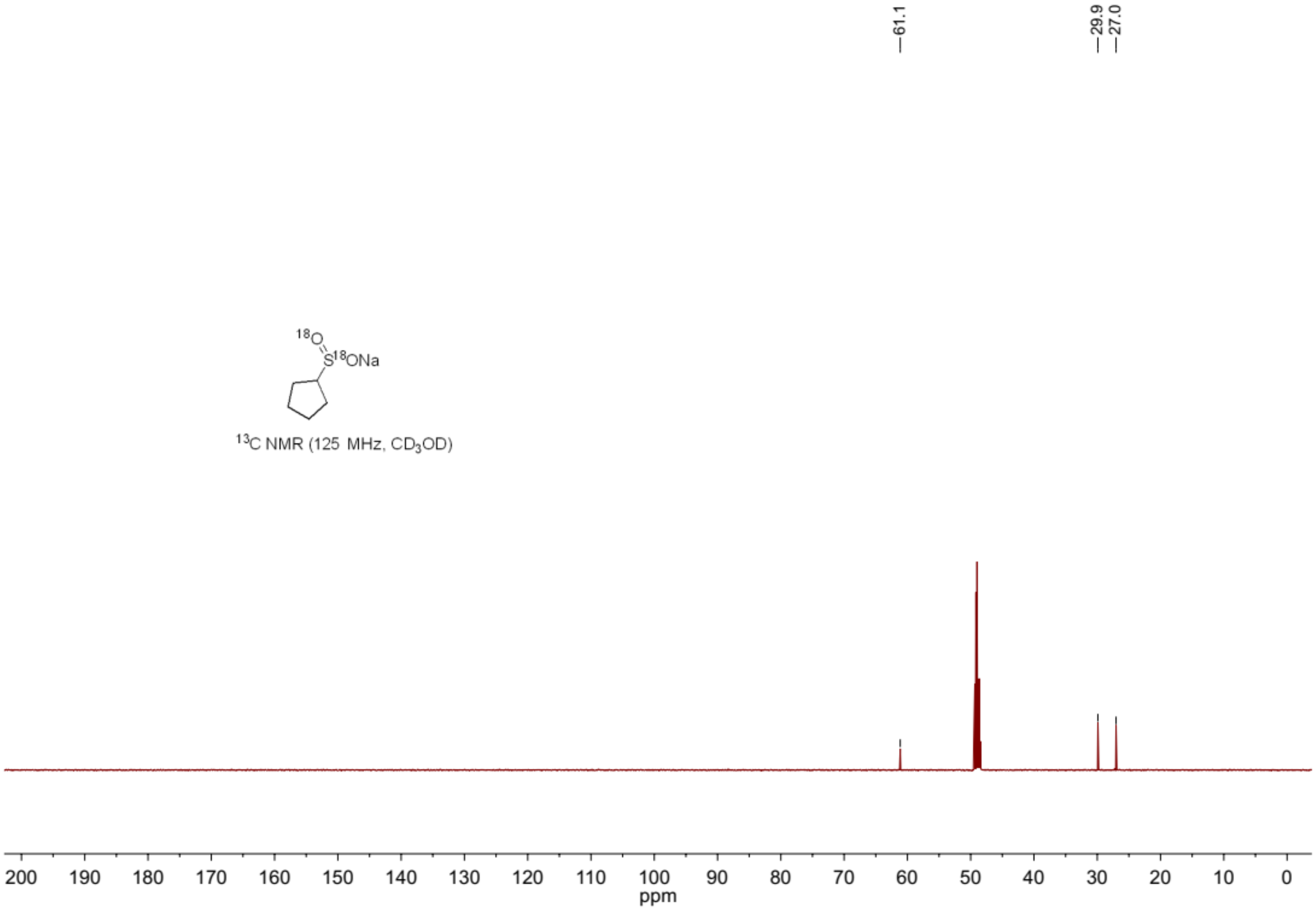

Go back to table of contents

7-Chloro-4-(cyclopentylsulfonyl)quinoline (48)

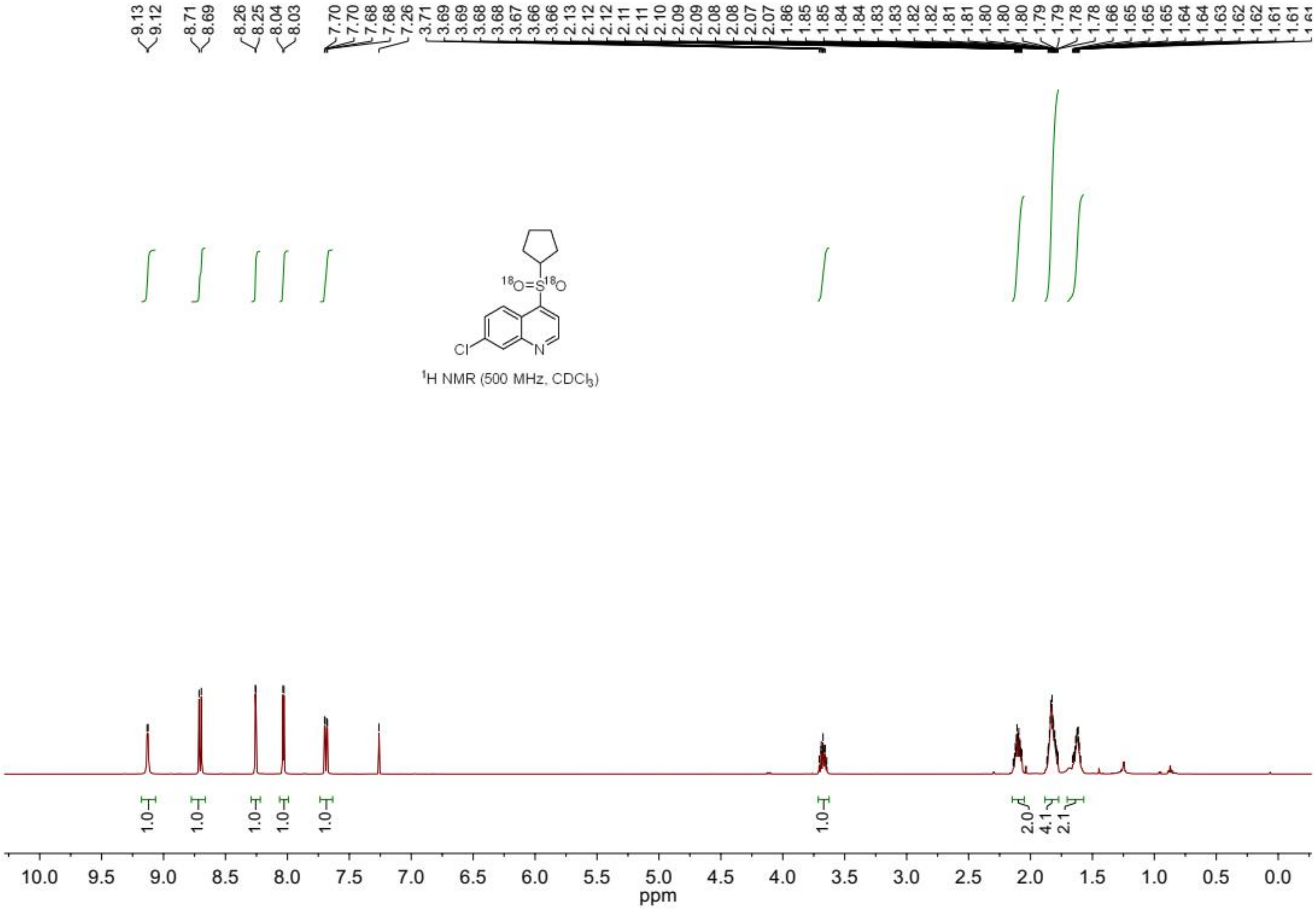

Go back to table of contents

7-Chloro-4-(cyclopentylsulfonyl)quinoline (48)

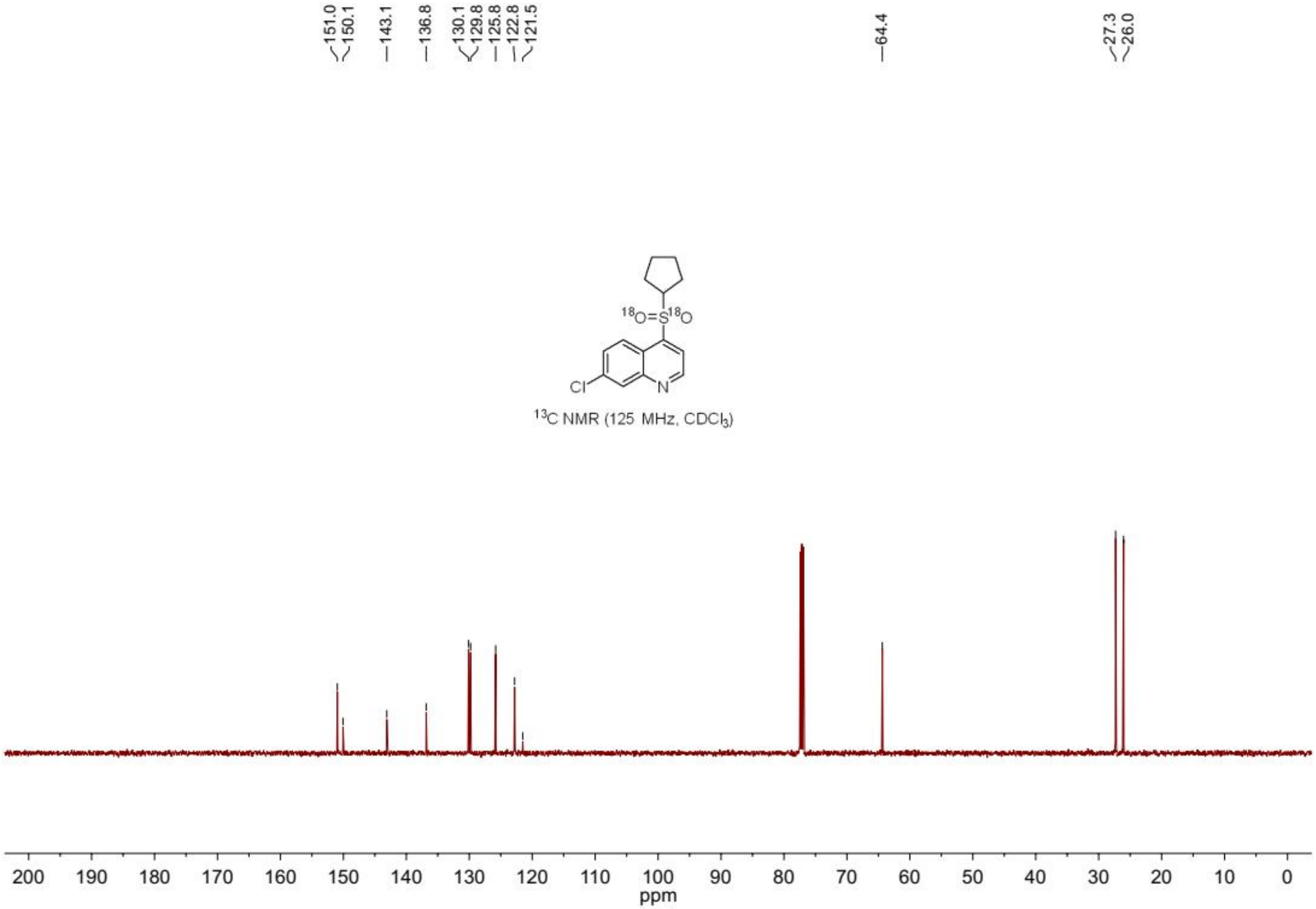

Go back to table of contents

References

1 L. R. Reddy, B. V. S. Reddy and E. J. Corey, *Org. Lett.*, 2006, **8**, 2819–2821.

2 A. Ortiz, L. Quintero, H. Hernandez, S. Maldonado, G. Mendoza and S. Bernes, *Tetrahedron Lett.*, 2003, **44**, 1129–1132.

3 G-X. Li, C. A. Morales-Rivera, F. Gao, Y. Wang, G. He, P. Liu and G. Chen, *Chem. Sci.*, 2017, **8**, 7180–7185.

4 E. M. Espinoza, J. A. Clark, J. Soliman, J. B. Derr, M. Morales and V. I. Vullev, *J. Electrochem. Soc.*, 2019, **116**, H3175−H3187.

5 N. J. Bunce, J. Lamarre and S. P. Vaish, *Photochem. Photobiol.*, 1984, **39**, 531-533.

6 J. M. Zaķis, K. Ozols, I. Novosjolova, R. Vilšķērsts, A. Mishnev and M. Turks, *J. Org. Chem.*, 2020, **85**, 4753–4771.

7 S. Chen, Y. Li, M. Wang and X. Jiang, *Green Chem.*, 2020, **22**, 322–326.

8 B. Zheng, T. Jia and P. J. Walsh, *Org. Lett.*, 2013, **15**, 1690–1693.

9 J. F. King and R. Rathore, *Tetrahedron Lett.*, 1989, **30**, 2763–2766.

10 Texas Advanced Computing Center (TACC), The University of Texas at Austin

11 Gaussian 16, Revision A.03, M. J. Frisch, G. W. Trucks, H. B. Schlegel, G. E. Scuseria, M. A. Robb, J. R. Cheeseman, G. Scalmani, V. Barone, G. A. Petersson, H. Nakatsuji, X. Li, M. Caricato, A. V. Marenich, J. Bloino, B. G. Janesko, R. Gomperts, B. Mennucci, H. P. Hratchian, J. V. Ortiz, A. F. Izmaylov, J. L. Sonnenberg, D.Williams-Young, F. Ding, F. Lipparini, F. Egidi, J. Goings, B. Peng, A. Petrone, T. Henderson, D. Ranasinghe, V. G. Zakrzewski, J. Gao, N. Rega, G. Zheng, W. Liang, M. Hada, M. Ehara, K. Toyota, R. Fukuda, J. Hasegawa, M. Ishida, T. Nakajima, Y. Honda, O. Kitao, H. Nakai, T. Vreven, K. Throssell, J. A. Montgomery, Jr., J. E. Peralta, F. Ogliaro, M. J. Bearpark, J. J. Heyd, E. N. Brothers, K. N. Kudin, V. N. Staroverov, T. A. Keith, R. Kobayashi, J. Normand, K. Raghavachari, A. P. Rendell, J. C. Burant, S. S. Iyengar, J. Tomasi, M. Cossi, J. M. Millam, M. Klene, C. Adamo, R. Cammi, J. W. Ochterski, R. L.

Go back to table of contents

Martin, K. Morokuma, O. Farkas, J. B. Foresman and D. J. Fox, Gaussian, Inc., Wallingford CT, 2016.

12 P. Pracht, F. Bohle and S. Grimme, *Phys. Chem. Chem. Phys.*, 2020, **22**, 7169–7192.

13 S. Grimme, C. Bannwarth and P. Shushkov, *J Chem. Theory Comput.*, 2017, **13**, 1989–2009.

14 C. Bannwarth, S. Ehlert and S. Grimme, *J. Chem. Theory Comput.*, 2019, **15**, 1652–1671.

15 CYLview, 1.0b, C. Y. Legault, Université de Sherbrooke, 2009 (http://www.cylview.org).

16 F. Neese, *WIREs: Comput. Mol. Sci.*, 2012, **2**, 73–78.

17 F. Neese, *WIREs: Comput. Mol. Sci.*, 2017, **8**, e1327.

18 (*a*) C. Riplinger and F. Neese, *J. Chem. Phys.*, 2013, **138**, 034106; (*b*) M. Sparta, B. Sandhoefer, A. Hansen and F. Neese, *J. Chem. Phys.*, 2013, **139**, 134101.

19 J. A. People, M. Head-Gordon and K. Raghavachari, *J. Chem. Phys.*, 1987, **87**, 5968–5975.

20 G. D. Purvis and R. J. Bartlett, *J. Chem. Phys*., 1982, **76**, 1910–1918.

21 R. A. Kendall, T. H. Dunning Jr. and R. J. Harrison, *J. Chem. Phys.* 1992, **96**, 6796–6806.

22 Y. Shao, E. Gan, A. Epifanovsky, A. T. B. Gilbert, M. Wormit, J. Kussmann, A. W. Lange, A. Behn, J. Deng, X. Feng, D. Ghosh, M. Goldey, P. R. Horn, L. D. Jacobson, I. Kaliman, R. Z. Khaliullin, T. Kús, A. Landau, J. Liu, E. I. Proynov, Y. M. Rhee, R. M. Richard, M. A. Rohrdanz, R. P. Steele, E. J. Sundstrom, H. L. Woodcock III, P. M. Zimmerman, D. Zuev, B. Albrecht, E. Alguire, B. Austin, G. J. O. Beran, Y. A. Bernard, E. Berquist, K. Brandhorst, K. B. Bravaya, S. T. Brown, D. Casanova, C.-M. Chang, Y. Chen, S. H. Chien, K. D. Closser, D. L. Crittenden, M. Diedenhofen, R. A. DiStasio Jr., H. Dop, A. D. Dutoi, R. G. Edgar, S. Fatehi, L. Frusti-Molnar, A. Ghysels, A. Golubeva-Zadorozhnaya, J. Gomes, M. W. D. Hanson-Heine, P. H. P. Harbach, A. W. Hauser, E. G. Hohenstein, Z. C. Holden, T.-C. Jagau, H. Ji, B. Kaduk, K. Khistyaev, J. Kim, J. Kim,

R. A. King, P. Klunzinger, D. Kosenkov, T. Kowalczyk, C. M. Krauter, K. U. Lao, A. Laurent, K. V. Lawler, S. V. Levchenko, C. Y. Lin, F. Liu, E. Livshits, R. C. Lochan, A. Luenser, P. Manohar, S. F. Manzer, S.-P. Mao, N. Mardirossian, A. V. Marenich, S. A. Maurer, N. J. Mayhall, C. M. Oana, R. Olivares-Amaya, D. P. O'Neill, J. A. Parkhill, T. M. Perrine, R. Peverati, P. A. Pieniazek, A. Prociuk, D. R. Rehn, E. Rosta, N. J. Russ, N. Sergueev, S. M. Sharada, S. Sharmaa, D. W. Small, A. Sodt, T. Stein, D. Stück, Y.-C. Su, A. J. W. Thom, T. Tsuchimochi, L. Vogt, O. Vydrov, T. Wang, M. A. Watson, J. Wenzel, A. White, C. F. Williams, V. Vanovschi, S. Yeganeh, S. R. Yost, Z.-Q. You, Y. Zhang, X. Zhang, Y. Zhou, B. R. Brooks, G. K. L. Chan, D. M. Chipman, C. J. Cramer, W. A. Goddard III, M. S. Gordon, W. J. Hehre, A. Klamt, H. F. Schaefer III, M. W. Schmidt, C. D. Sherrill, D. G. Truhlar, A. Warshel, X. Xua, A. Aspuru-Guzik, R. Baer, A. T. Bell, N. A. Besley, J.-D. Chai, A. Dreuw, B. D. Dunietz, T. R. Furlani, S. R. Gwaltney, C.-P. Hsu, Y. Jung, J. Kong, D. S. Lambrecht, W. Liang, C. Ochsenfeld, V. A. Rassolov, L. V. Slipchenko, J. E. Subotnik, T. Van Voorhis, J. M. Herbert, A. I. Krylov, P. M. W. Gill and M. Head-Gordon, *Mol. Phys.*, 2015, **113**, 184–215.

23 NBO 7.0. E. D. Glendening, J. K. Badenhoop, A. E. Reed, J. E. Carpenter, J. A. Bohmann, C. M. Morales, P. Karafiloglou, C. R. Landis and F. Weinhold, Theoretical Chemistry Institute, University of Wisconsin, Madison, **2018**.

24 Chemcraft - graphical software for visualization of quantum chemistry computations. https://www.chemcraftprog.com

25 T. Lu and F. Chen, *J. Comp. Chem.*, 2012, **33**, 580–592.

26 Humphrey, W., Dalke, A. and Schulten, K. "VMD - Visual Molecular Dynamics", *J. Molec. Graphics*, 1996, **14**, 33–38.

27 S. Grimme, J. Antony, S. Ehrlich and H. Krieg, *J. Chem. Phys.*, 2010, **132**, 154104.

28 Y. Zhao and D. G. Truhlar, *J. Phys. Chem. A*, 2005, **109**, 5656–5667.

29 F. Weigend and R. Ahlrichs, *Phys. Chem. Chem. Phys.*, 2005, **7**, 3297–3305.

Go back to table of contents

30 J. L. Pascual-Ahuir, E. Silla and I. Tuñón, *J. Comp. Chem.*, 1994, **15**, 1127–1138.

31 S. Miertuš and J. Tomasi, *Chem. Phys.,* 1982, **65**, 239–245.

32 S. Miertuš, E. Scrocco and J. Tomasi, *Chem. Phys.,* 1981, **55**, 117–129.

33 B. Allard, A. Casadevall, G. Casadevall and C. Largeau, *Nouv. J. Chim.,* 1979, **3**, 335–342.

34 Derived as a square of the refractive index *n* = 1.275 at 25 °C: E. E. Drott, *Liquid Chromatography of Polymers and Related Materials,* ed. J. Cazes, Marcel Dekker, New York, 1977, vol. 8.

35 A. Hansen, C. Bannwarth, S. Grimme, P. Petrović, C. Werlé and J.-P. Djukic, *ChemistryOpen*, 2014, **3**, 177–189.

36 (*a*) G. Luchini, J. V. Alegre-Requena, Y. Guan, I. Funes-Ardoiz and R. S. Paton, GoodVibes: GoodVibes v3.0.1 (2019); (*b*) G. Luchini, J. V. Alegre-Requena, Y. Guan, I. Funes-Ardoiz and R. S. Paton, *F1000Research* 2020, **9**, 291.

37 J.-D. Chai and M. Head-Gordon, *Phys. Chem. Chem. Phys.*, 2008, **10**, 6615–6620.

38 F. Weigend and R. Ahlrichs, *Phys. Chem. Chem. Phys.*, 2005, **7**, 3297–3305.

39 L. Goerigk and S. Grimme, *J. Chem. Theory Comput.,* 2011, **7**, 291–309.

40 L. Goerigk, ccA. Hansen, C. Bauer, S. Ehrlich, A. Najibi and S. Grimme, *Phys. Chem. Chem. Phys.,* 2017, **19**, 32184.

41 (*a*) K. Morokuma, *J. Chem. Phys.,* 1971, **55**, 1236–1244; (*b*) L. P. Wolters and F. M. Bickelhaupt, *WIREs Comput. Mol. Sci*., 2015, **5**, 324–343; (*c*) F. M. Bickelhaupt and K. N. Houk, *Angew. Chem., Int. Ed.*, 2017, **56**, 10070–10086; (*d*) D. H. Ess and K. N. Houk, *J. Am. Chem. Soc.,* 2007, **129**, 10646–10647.

42 (*a*) R. Z. Khaliullin, E. A. Cobar, R. C. Lochan, A. T. Bell and M. Head-Gordon, *J. Phys. Chem. A,* 2007, **111**, 8753–8765; (*b*) P. R. Horn, E. J. Sundstrom, T. A. Baker and M. Head-Gordon, *J. Chem. Phys.,* 2013, **138**, 134119; (*c*) P. R. Horn, Y. Mao and M. Head-Gordon, *Phys. Chem. Chem. Phys.,* 2016, **18**, 23067.

Go back to table of contents

43 R. Z. Khaliullin, A. T. Bell and M. Head-Gordon, *J. Chem. Phys*. 2008, **128**, 184112.

44 A. A. Isse and A. Gennaro, *J. Phys. Chem. B,* 2010, **114**, 7894–7899.

45 (*a*) M. Sparta and F. Neese, *Chem. Soc. Rev.,* 2014, **43**, 5032–5041; (*b*) I. Sandler, J. Chen, M. Taylor, S. Sharma and J. Ho, *J. Phys. Chem.,* 2021, **125**, 1553–1563.

46 D. G. Liakos, M. Sparta, M. K. Kesharwani, J. M. L. Martin and F. Neese, *J. Chem. Theory. Comput.,* 2015, **11**, 1525–1539.

Go back to table of contents
